# Synthesis of Benzopyran–Phenylpropanoid Hybrids via Matsuda–Heck-Arylation and Allylic Oxidation

**DOI:** 10.1021/acs.joc.4c02520

**Published:** 2024-12-07

**Authors:** Fabian Otte, Julia Greese, Stefan Foß, Mandy Krüger, Eric Sperlich, George Kwesiga, Bernd Schmidt

**Affiliations:** † Universitaet Potsdam, Institut fuer Chemie, Karl-Liebknecht-Straße 24-25, D-14476 Potsdam-Golm, Germany; ‡ Kabale University, Department of Chemistry, P.O. Box 317, Kabale, Uganda

## Abstract

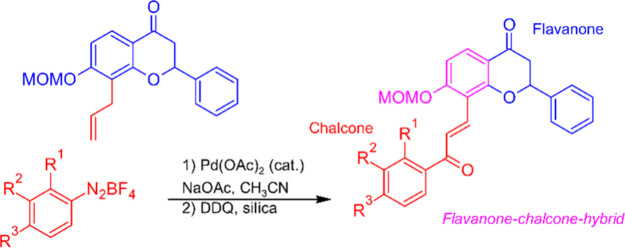

The synthesis of coumarin– and flavonoid–chalcone hybrids via Pd-catalyzed Heck-type coupling of arene diazonium salts and 8-allylcoumarins and −flavonoids is reported. The β-hydride elimination step proceeds with high regioselectivity if an OMOM-substituent is present at the position C7, adjacent to the allyl group. A selective allylic oxidation of the coupling products was accomplished using DDQ in the presence of silica to furnish the chalcones.

## Introduction

**Figure 1:**
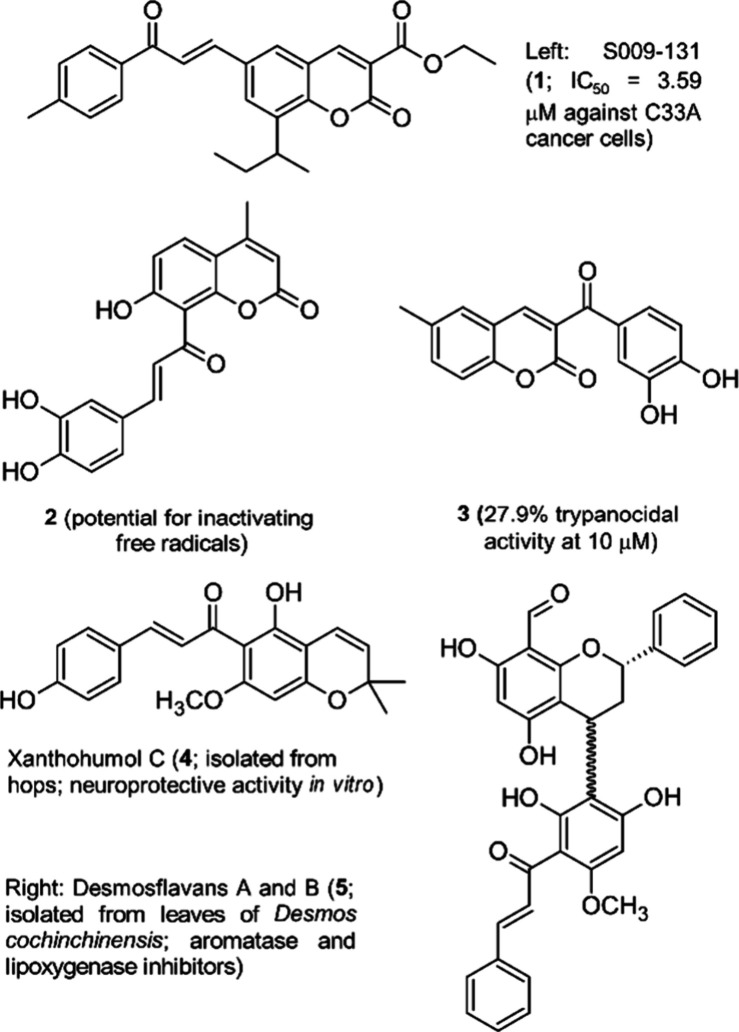
Benzopyran–chalcone hybrids and their bioactivities.

In medicinal chemistry hybrid molecules are defined as compounds with two or more pharmacologically active molecular entities that are linked through covalent bonds. The different pharmacophores can either bind to the same target, independently to different targets or simultaneously to two or more targets in close proximity.^1^ For example, trioxaquines are antimalarial drugs in which a 1,2,4-trioxane inspired by artemisinin is linked to a chloroquine moiety. The activity of these hybrids against chloroquine resistant *Plasmodium falciparum* strains is significantly higher than the activity of the single molecules.^1^ Many hybrid molecules based on both natural product and synthetic pharmacophores have been developed since the original disclosure of the concept and investigated for numerous anti-infective activities, anti-inflammatory activity and anticancer activity.^2^ Although some natural products are already hybrid molecules in themselves, it has been reasoned that the synthetic linkage of natural products can further broaden the chemical space of pharmacologically active compounds.^3^ A natural product scaffold that has been incorporated in many synthetic hybrid structures is the chalcone moiety. Chalcones are naturally occurring 1,3-diarylpropenones, a subgroup of diarylpropanoids, that are mostly found in plants.^4^ They are biosynthetically derived from coumaryl-CoA and three units of malonyl-CoA and are biosynthetic precursors of flavanones, flavones and isoflavones (Figure 1). For this reason, chalcones accumulate only in a limited number of plants, which, however, play a prominent role in traditional medicine.^4^

Bioactivities that have been reported for naturally occurring chalcones range from anti-infective activities (antibiotic, antiviral, antifungal, antiprotozoal activity) over cytotoxic^5^ to enzyme inhibiting activity (e.g., inhibition of tyrosinase^6^) and cardio- and neuroprotective effects.^4,7^ By using strategies such as bioisosterism, pro-drug development and molecular hybridization chalcones have been identified as attractive entry points for the design and development of new lead structures and drugs.^8^ Hybrids of chalcones and other natural product (e.g., quinolines, artemisinin and coumarins) and synthetic (e.g., azoles and ferrocenes) scaffolds have inter alia been tested as anticancer^9−12^ and antimalarial^13^ agents. An example is the synthetic coumarin–chalcone hybrid **1**,^14^ which was named S009-131 and proposed as a promising candidate for the treatment of cervical cancer as it inhibits proliferation of HeLa and C33A cancer cells by apoptosis.^15^ Compound S009-131 (**1**) triggers DNA damage through binding to the minor groove via hydrogen bonds and stabilizes the tumor suppressor protein p53.^16^ Other coumarin–chalcone hybrids were successfully tested for their radical scavenging ability (e.g., **2**)^17^ toward reactive oxygen species (ROS),^17−20^ for their antibacterial^21^ and trypanocidal activities (e.g., **3**),^22^ and as selective inhibitors of monoamine oxidase B (MAO-B).^23^ The pyranochalcone xanthohumol C (**4**) is a naturally occurring chromene–chalcone hybrid isolated from hops that shows neuroprotective activity,^24^ and desmosflavans A and B (**5**)^25^ are diastereomeric plant metabolites with a flavane–chalcone hybrid structure (Figure 1).

Most syntheses of coumarin–chalcone hybrids^8^ rely on classical carbonyl condensation methods, such as Claisen–Schmidt-condensation,^14^ Knoevenagel-condensation/cyclization sequences^18,22^ or the Friedel–Crafts acylation of coumarins and subsequent Claisen–Schmidt-condensation.^17^ The development of alternative approaches would open up the opportunity to use other starting materials and reaction conditions and access substitution patterns that can not, or only with difficulties, be realized through conventional carbonyl condensation chemistry.

**Scheme 1:**
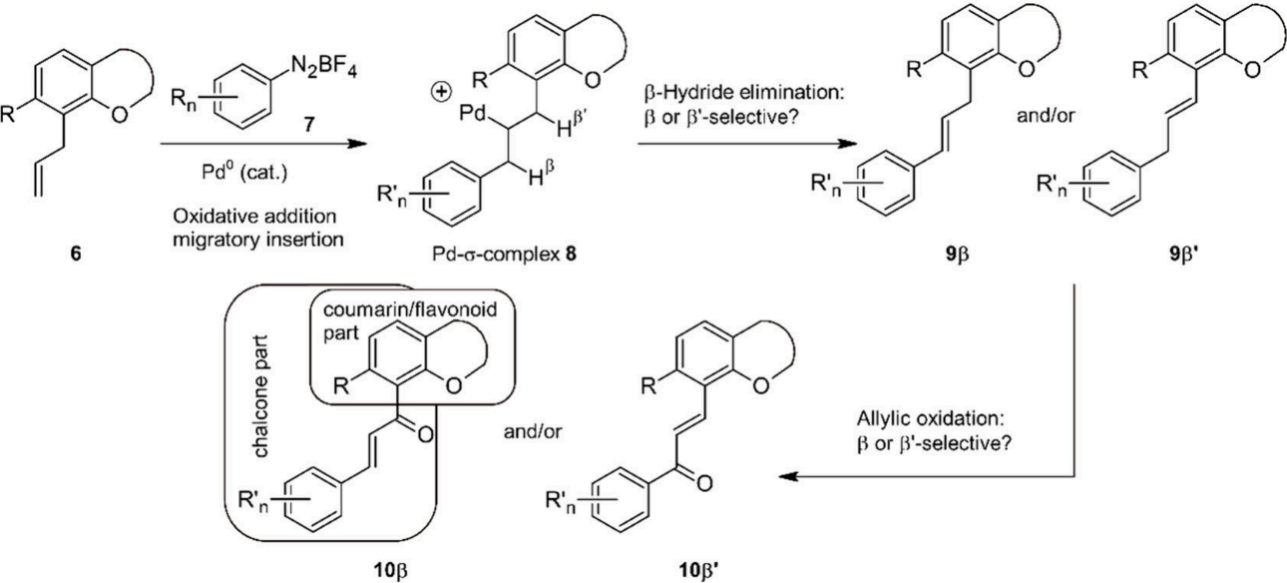
Possible Products of Matsuda–Heck Reactions for 8-Allylbenzopyranones **6**

We^26−28^ and others^29−34^ have investigated the Pd-catalyzed Heck-type arylation using arene diazonium salts as electrophilic coupling partners, commonly referred to as Matsuda–Heck reaction,^35^ for several years.^36−38^ This reaction allows the regio- and stereoselective arylation of olefins at ambient temperature under ligand-free conditions in the absence of strong bases or even under base-free conditions. A remarkable feature is the possibility to introduce electron-rich arenes, including unprotected phenols,^39^ which is particularly important for the synthesis of chalcones and other diarylpropanoids. With a view to the synthesis of diarylpropanoid–coumarin and diarylpropanoid–flavonoid hybrids with the general structure **9** we investigated the Matsuda–Heck-arylation of 8-allylbenzopyranones **6** (i.e., coumarins, flavanones, flavones and isoflavones) and arene diazonium salts **7**. After oxidative addition of the electrophilic coupling partner **7** to the Pd(0) catalyst and migratory insertion into the C–C-double bond a cationic Pd–II-σ-complex **8** is formed, which undergoes β-hydride elimination either with H^β^ to the product **9β** or with H^β′^ to the product **9β′**. The Heck-coupling products **9** will then be converted to chalcone–coumarin or chalcone–flavonoid hybrids **10** through allylic oxidation, a reaction that might also be associated with a regioselectivity issue (Scheme 1).

In this work, we investigate whether the regioselectivity issues described above can be controlled, either by choosing appropriate reaction conditions or by varying the substituent R adjacent to the olefin, and whether the sequence of Pd-catalyzed arylation and allylic oxidation opens up a synthetically useful route to chalcone hybrids.

## Results and Discussion

### Optimization of Conditions for the Coupling of Arene Diazonium Salts with Allylarenes

As an entry point toward benzopyrane–chalcone hybrids we identified the MOM-protected 8-allylcoumarin **11a** as an olefinic coupling partner. This compound was available from previous projects in our group directed at the synthesis of prenylated coumarin natural products from plants.^40^ We knew from our earlier experience with Matsuda–Heck reactions that different types of olefins require a specific optimization of reaction conditions, but that the number of variables is fortunately limited: most Matsuda–Heck reactions with electron-deficient alkenes (e.g., acrylates) work well in alcohols (preferably methanol),^27^ whereas for electron-rich alkenes, such as enolethers, better results are obtained in acetonitrile.^28^ For related arene substituted alkenes (styrenes, allylbenzene) THF^41^ and *N*,*N*-dimethylacetamide^42^ have been used as solvents, which were therefore also included in this study. The second important variable is the presence or absence of a base. In those cases where basic conditions are preferable sodium acetate is normally added to the reaction mixture.

For optimization purposes we have used in this and earlier studies the 4-methoxyphenyl diazonium salt **7f**, because the symmetry of the 4-methoxyphenyl substituent simplifies NMR-spectroscopic analysis of crude reaction mixtures and therefore allows rates of conversion to be estimated. The experiments summarized in Table 1 revealed that for electron-neutral allylbenzene-type olefins such as **11a**, basic conditions are mandatory in all solvents. In the absence of a base (entries 2, 4, 6 and 8) ^1^H NMR spectra of the reaction solutions pointed at a decomposition of the starting material without formation of any defined product. In these cases, no characteristic signals of either **11a** or **12af** were observed, and in all cases the MOM-ether was cleaved. Under basic conditions (addition of 3 equiv of NaOAc to the reaction mixture) in DMA (entry 7) only unreacted starting material **11a** was observed in the ^1^H NMR spectrum, whereas in THF (entry 5) conversion to the coupling product **12af** was ca. 30%. Synthetically useful rates of conversion were only observed in methanol (entry 1) and in acetonitrile (entry 3). In the latter solvent conversion was quantitative.

**Table 1:**
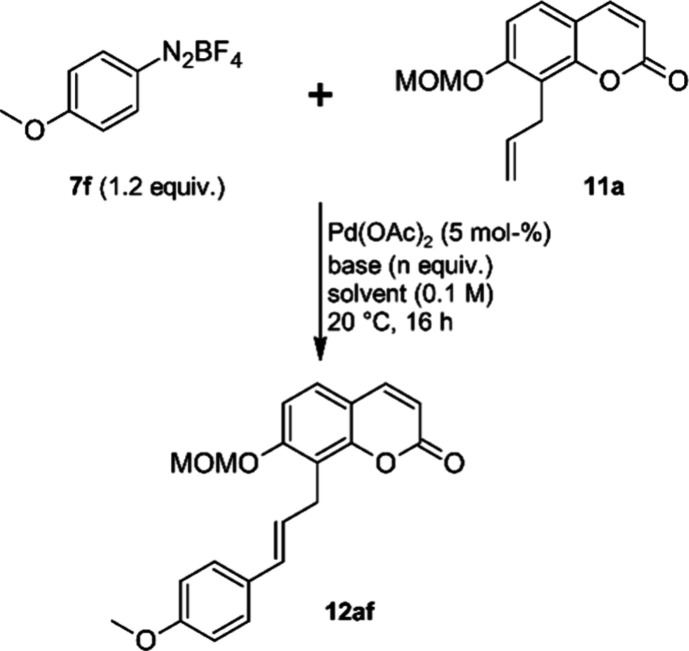
Screening of Conditions for the Pd-Catalyzed Arylation of **11a**

entry	solvent	Base (*n* equiv)	Conversion to **12af** (isolated yield)^a^
1	methanol	NaOAc (3.0)	>80^b^ (50)
2	methanol	–	dec.^c^
3	acetonitrile	NaOAc (3.0)	quant. (79)
4	acetonitrile	–	dec.^c^
5	THF	NaOAc (3.0)	<30%^b^
6	THF	–	<10%^d^
7	DMA^e^	NaOAc (3.0)	<5^b^
8	DMA^e^	–	dec.^c^

a Conversion was determined by TLC and ^1^H NMR spectroscopy of the reaction solution.

b Starting material **11** detected in ^1^H NMR spectrum.

c dec: decomposition; no signals of **12af** or **11a** visible in ^1^H NMR spectrum.

d Signals for **11a** with MOM-ether cleavage observed in ^1^H NMR spectrum.

e DMA: *N*,*N*-dimethylacetamide.

Regardless of the solvent used, we found that this particular Matsuda–Heck reaction is rather slow compared to many other Pd-catalyzed couplings with arene diazonium salts that were previously investigated in our group. For this reason we routinely used a reaction time of 16 h and employed the diazonium salt in slight excess. Under the optimized conditions (entry 3; acetonitrile as solvent, addition of NaOAc as a base) we obtained the coumarin–phenylpropanoid hybrid **12af** after chromatographic purification as a single isomer in 79% yield.

### Representative Structure Elucidation of Coupling Product **12af**

**Figure 2:**
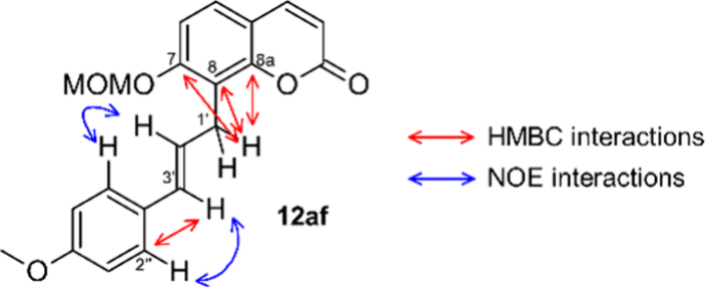
HMBC and NOE interactions in **12af**.

^1^H NMR-spectra of the crude reaction mixtures of **11a** and **7f** (Table 1, entries 1 and 3) revealed that **12af** was the predominant product, but that small amounts of other products were also formed. These may arise from decomposition reactions of the diazonium salt, which is used in excess, because additional signals are mainly observed in the aromatic region. The NMR spectra of the reaction mixtures did not point at the formation of any regio- or stereoisomers of the predominant coupling product **12af**, because we observed only the signals for the *E*-configured C=C-double bond of **12af** in the olefinic region of the ^1^H NMR spectrum. Chromatographic purification led to the isolation of the main product as a single isomer with an *E*-configured double bond, based on a ^3^*J* value of 15.8 Hz. It was, however, not possible to assign the position of the double bond based on standard 1D-NMR-spectra alone. Therefore, a full signal assignment based on the 2D-experiments H,H–COSY, HSQC, HMBC and NOESY was performed. Indicative for the assigned structure of **12af**, pointing at a β- rather than a β′-selective hydride elimination, are four HMBC interactions. The HMBC experiment is most sensitive for C–H-couplings across three, and to some extent across two bonds. We observed strong cross peaks between the allylic hydrogen atoms (position H1′) and quaternary carbons C7, C8 and C8a located in the coumarin part of the hybrid molecule. A fourth HMBC interaction was observed between the olefinic hydrogen H3′ and carbon C2″ of the 4-methoxyphenyl ring that was introduced by the Pd-catalyzed arylation. Other HMBC interactions (not shown in Figure 2) between the central position of the propanoid chain (H2′) and C8 of the coumarin part and C1″ of the 4-methoxyphenyl substituent are visible but are not suitable to distinguish between the possible regioisomers. This structural assignment was corroborated by two NOE-interactions between the H2″-protons of the 4-methoxyphenyl ring and both olefinic protons in the propene bridge (H2′ and H3′) (Figure 2).

### Scope and Limitations of the Matsuda–Heck Coupling Reactions with 8-Allylcoumarin **11a**

We applied the optimized conditions for the coupling of **11a** with **7f** to 21 other *ortho*-, *meta*-, and *para*-substituted arene diazonium salts **7** (Table 2). With few exceptions the coupling products **12** were obtained in synthetically useful yields and in all cases as single isomers. We repeated the structure elucidation and full signal assignment process based on 2D-NMR experiments, as described in the preceding paragraph for **12af**, for coupling products **12ah**, **12ai**, **12ap** and **12at**. With this selection a broad range of electronic and steric substituent effects at the electrophilic coupling partners **7** was covered, from strongly electron donating (*para*-OCH_3_, **12af**) over moderately electron donating (*para*-CH_3_, **12ah**) to strongly electron withdrawing (*para*-CO_2_Et, **12ai**). Compounds **12ap** (*meta*-OCH_3_), **12at** (*ortho*-OCH_3_) and **12au** (*ortho*-CO_2_CH_3_) result from arene diazonium salts with varying degrees of steric hindrance and electron densities at the reactive site. We reasoned that both electronic and steric effects of the substituents might affect the course of the β-hydride elimination step. This would remain undetected by routine ^1^H and ^13^C NMR spectra, as the expected chemical shift values, multiplicities and coupling constants for the propene bridges in **9β** and **9β′** are expected to be very similar. For coupling products **12ah**, **12ai**, **12ap** and **12at** the same HMBC- and NOE-interactions were observed as for **12af**, which indicates that the β-hydride elimination step is not affected by steric or electronic substituent effects, but gives mainly **9β**-isomers irrespective of the arene diazonium salt used. Only in the cases of **7j** (entry 10; *para*-NO_2_, complex mixture of products), **7s** (entry 19; *ortho*-Br, incomplete and sluggish conversion), and **7v** (entry 22; *ortho*-CH_3_, no conversion) no coupling products **12** could be isolated. In the cases of **7s** and **7v** significant amounts of unreacted starting material **11a** were recovered. The failure of the coupling reaction with most *ortho*-substituted arene diazonium salts might be explained by steric hindrance.

**Table 2:**
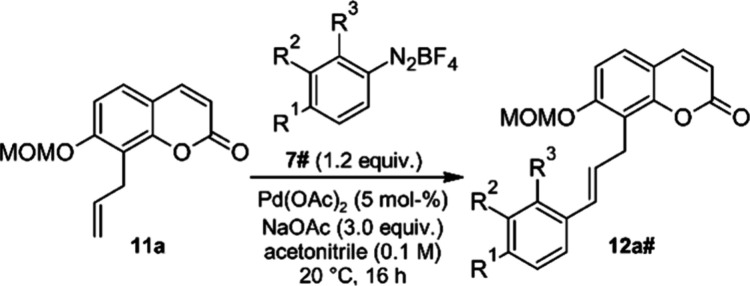
Synthesis of Coupling Products **12**: Scope and Limitations

entry	**7**	R^1^	R^2^	R^3^	**12**	yield (%)
1	**7a**	H	H	H	**12aa**	74
2	**7b**	F	H	H	**12ab**	67
3	**7c**	Cl	H	H	**12ac**	73
4	**7d**	Br	H	H	**12ad**	75
5	**7e**	I	H	H	**12ae**	61
6	**7f**	OCH_3_	H	H	**12af**	79
7	**7g**	OH	H	H	**12ag**	53
8	**7h**	CH_3_	H	H	**12ah**	71
9	**7i**	CO_2_Et	H	H	**12ai**	79
10	**7j**	NO_2_	H	H	**12aj**	<5^a^
11	**7k**	CF_3_	H	H	**12ak**	49
12	**7l**	H	F	H	**12al**	73
13	**7m**	H	Cl	H	**12am**	72
14	**7n**	H	Br	H	**12an**	50
15	**7o**	H	I	H	**12ao**	24
16	**7p**	H	OCH_3_	H	**12ap**	93
17	**7q**	H	CH_3_	H	**12aq**	75
18	**7r**	H	CO_2_CH_3_	H	**12ar**	69
19	**7s**	H	H	Br	**12as**	<5^b^
20	**7t**	H	H	OCH_3_	**12at**	64
21	**7u**	H	H	CO_2_CH_3_	**12au**	75
22	**7v**	H	H	CH_3_	**12av**	<5^b^

a Complex mixture of products.

b Low conversion (<5%); unreacted starting material recovered.

### Mechanistic Rationale for the Observed Regioselectivity

Electronically nonbiased olefins, such as allylarene **11a**, normally give mixtures of regioisomeric coupling products in Heck-type arylations, because most catalyst systems are unable to distinguish between the electronically very similar β-hydrogens (designated as H^β^ and H^β′^ in Scheme 1).^43^ A solution to this problem was proposed by Sigman and co-workers, who found that a cationic bimetallic Pd–Cu-catalyst system equipped with a strong σ-donor ligand yields styrene-type coupling products in oxidative Heck-coupling reactions with very high regioselectivity. In this study, arene boronic acids were used as arylating agents.^44^ The authors reasoned that this catalyst system is sensitive to subtle differences in C–H^β^ and C–H^β′^ bond strengths, which are caused by the electronic nature of the substituents at the arene boronic acid. In a follow-up study, Sigman and co-workers investigated the Heck-type coupling of the nonbiased olefin allylbenzene with various arene diazonium salts bearing electron-donating or electron-withdrawing substituents.^42^ It was found that only with the donor solvent *N*,*N*-dimethylacetamide (DMA) high selectivity in the β-hydride elimination step could be accomplished, and that strongly electron-donating substituents at the arene diazonium salt favor the formation of the double bond in conjugation to the newly introduced arene. This selectivity can be explained by a relatively higher hydridic character of the β-hydrogen adjacent to the aryl substituent coming from the diazonium salt.

Our results obtained for the Pd-catalyzed arylation of **11** with arene diazonium salts show some notable differences: a) contrary to the results from the Sigman group, DMA is an unsuitable solvent, but acetonitrile in combination with a base results in high conversions, irrespective of the electronic nature of the diazonium salt (Table 1). This solvent, however, gave poor yields and selectivity in the arylation of allylbenzene, as described by Sigman and co-workers;^42^ b) as outlined in the previous section, no significant and coherent effects of the electronic nature of the substituents at the diazonium salt on yield and selectivity were observed for the Pd-catalyzed couplings of **11**. In all cases, the same regioisomers **12** were obtained, which points at a strongly preferred elimination of H^β^ irrespective of the electronic nature of the arylating agent.

**Figure 3:**
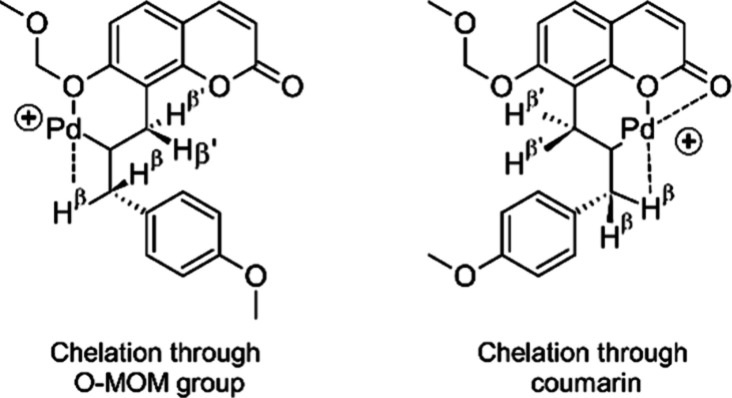
Proposed Pd-σ-alkyl chelates.

The observed selectivity can be explained if a coordination of the cationic and electrophilic Pd atom in Pd-σ-complex **8** (Scheme 1) is assumed. Selectivity control of the β-hydride elimination step through chelation with O- and N-donor groups of the substrate has previously been suggested by White and co-workers to rationalize unusually high levels of regioselectivity in oxidative Heck reactions.^45^ In our case, chelation might occur through the O-MOM group or through the ring oxygen and/or carbonyl oxygen of the coumarin heterocycle. In both cases the β-hydrogens H^β^ will be located at an *exo*-CH_2_-group that is connected to the chelate complex via a freely rotatable C–C-bond, which will allow a synperiplanar orientation of Pd and H^β^. On the other hand, for both chelate complexes the β-hydrogens H^β′^ will be located at an endocyclic C atom. This should result in limited conformational flexibility, which will make synperiplanar conformations with the Pd atom less likely (Figure 3).

**Scheme 2:**
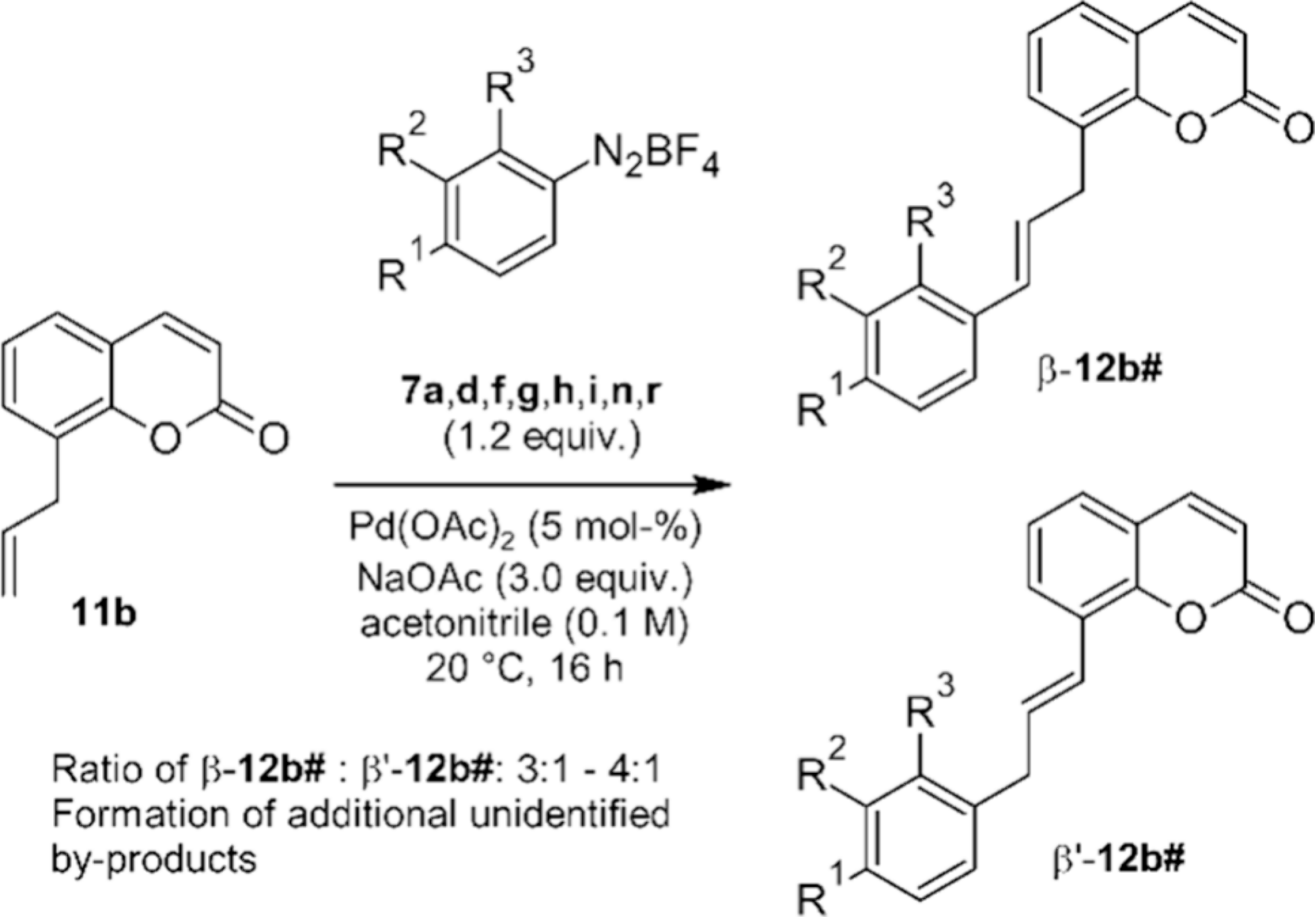
Arylation Reactions without a Chelating OMOM Group

To test the hypothesis that chelation of the cationic Pd-center contributes to the regioselectivity of the β-hydride elimination step, we first investigated the Matsuda–Heck coupling of 8-allylcoumarin (**11b**),^46^ that lacks a coordinating substituent at C-7, with various diazonium salts (**7a**,**d**,**f**,**g**,**h**,**i**,**n**,**r**) under our standard conditions (Scheme 2).

For all coupling reactions of **11b** the regioisomer resulting from hydride elimination of H^β^ (β-**12b#**) is the major product. However, in contrast to the coupling products of **11a**, notable amounts of the β′-isomer, in which the double bond is in conjugation with the coumarin part, were detected. The β- and β′-isomers were obtained as inseparable mixtures after chromatography, and the product ratio was determined from the ^1^H NMR spectrum by integration of the signals for the CH_2_-group. The ratio of regioisomers (β:β′) is in the range of 3:1 to 4:1 in all cases. All coupling reactions of **11b** proceeded rather sluggishly with the formation of several inseparable and unidentified byproducts, which prevented the isolation of pure coupling products and determining reliable yields. We conclude that coordination of the OMOM-group to the electrophilic Pd substantially contributes to the observed selectivity of the β-hydride elimination, but that a coordination to the ring-oxygen and probably the carbonyl oxygen is also a notable contributor to the observed regioselectivity.

**Scheme 3:**
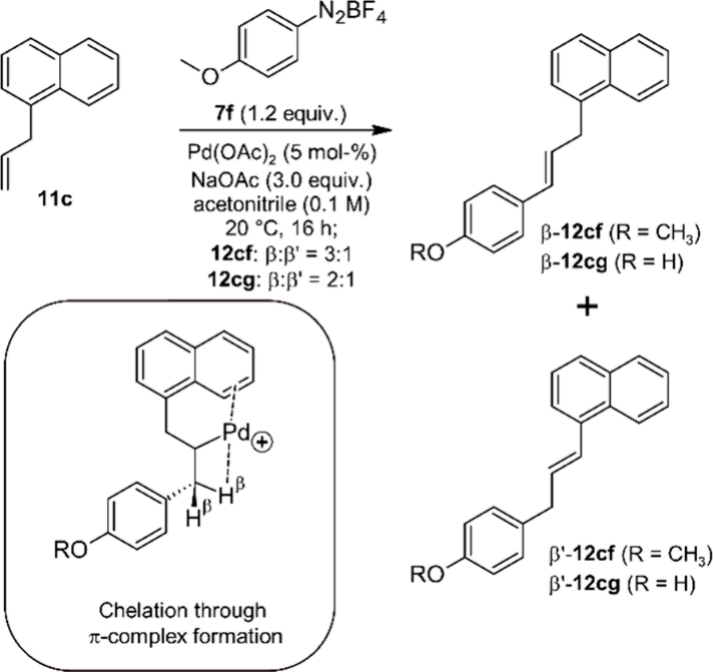
Arylation of 1-Allylnaphthalene (**11c**) with **7f** and **7g**

In order to further reduce possible coordinating sites at the allylarene substrate while maintaining the general steric situation of a coumarin, we investigated the regioselectivity of the coupling reaction of 1-allylnaphthalene (**11c**) and diazonium salts **7f** and **7g** (Scheme 3). The two regioisomers **β-12cf** and **β′-12-cf** were isolated as an inseparable mixture in a ratio (β:β′ = 3:1) very similar to that observed for the coupling reaction of 8-allylcoumarin (**11b**) with the same diazonium salt **7f**. A similar result was obtained for the coupling reaction of 1-allylnaphthalene (**11c**) with 4-phenoldiazonium salt **7g** (ratio β-**12cg**: β′-**12cg** = 2:1). These observations suggest that the π-system of the annulated benzene ring coordinates to the electrophilic Pd similar to a ring- and/or carbonyl-oxygen of a coumarin. The major product β-**12cf** was identified by comparing its NMR data with literature data reported for the same compound, but synthesized independently.^47^ As for 8-allylcoumarin (**11b**), both coupling reactions investigated with **11c** proceed sluggishly and with incomplete conversion, and with formation of other byproducts that could not be separated from products **12cf** and **12cg**, respectively. Therefore, a yield can again not be reported for these coupling products, but the ratio of β- and β′-isomer could be determined from the ^1^H NMR-spectrum through integration of the signals for the CH_2_-protons. In the particular case of **12cf** we also observed a doublet (*J* = 15.5 Hz) of the C=C-double bond of **β′-12cf**, which is shifted by 0.5 ppm downfield from the corresponding proton of **β-12cf**. Integration of these protons confirms the ratio obtained through integration of the CH_2_-groups.

**Scheme 4:**
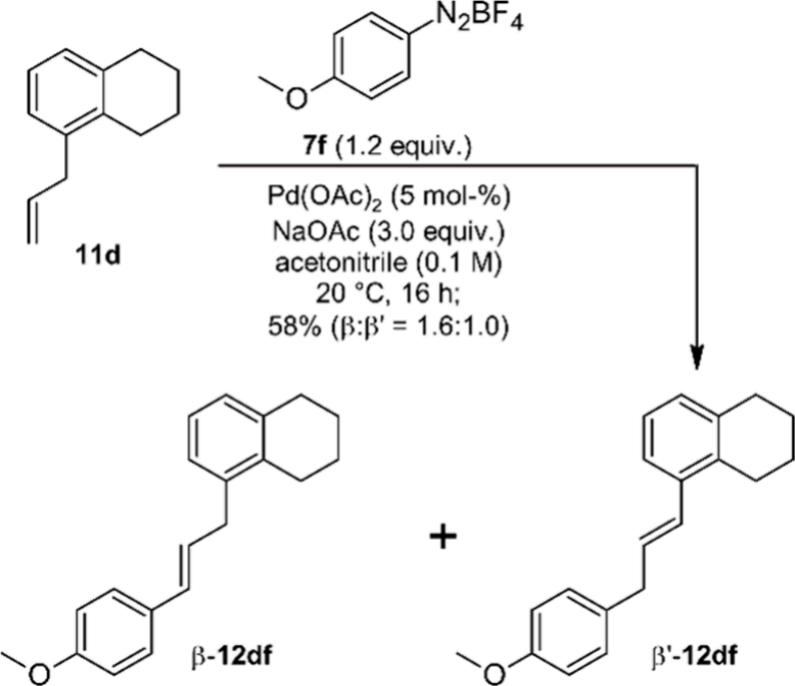
Arylation of 5-Allyl-1,2,3,4-tetrahydronaphthalene (**11d**) and **7f**

With the aim to rule out any bias of the β-hydride elimination step caused by chelation, we investigated the Heck coupling of 5-allyl-1,2,3,4-tetrahydronaphthalene **11d** under our standard conditions. Model substrate **11d** was synthesized in four steps from commercially available naphthalene-1-yl-acetic acid (see Supporting Information for details). We assume that the steric situation in test substrate **11d** is similar to that of **11b** and **11c**, but that a chelation of the electrophilic Pd in the transition state of the β-hydride elimination step is not possible (Scheme 4).

We noticed that under the standard conditions conversion of **11d** remained incomplete. Analysis of the product mixture by GC-MS revealed that five isomeric coupling products were formed, and ^1^H NMR spectroscopy indicates that β-**12df** and β′-**12df** are the main products. These isomers are present in a ratio of 1.6:1.0. The slight preference of β-**12df** is most likely caused by steric effects.

### Allylic Oxidation of Heck-Coupling Products **12**

Numerous stoichiometric and catalytic methods for allylic and benzylic oxidation reactions are available. These methods^48^ and their applications in natural product synthesis^49^ have been reviewed. To identify a suitable method for the envisaged allylic oxidation and optimize the reaction conditions for the task at hand, Heck coupling product **12af** was chosen as a substrate (Table 3). We first investigated Ru-catalyzed protocols. This chemistry, pioneered by Murahashi and co-workers,^50^ relies on the use of catalytic amounts of a Ru source, e.g. [Ru(PPh_3_)_2_Cl_2_],^51^ in combination with an oxidant such as *tert*-butyl hydroperoxide. During our investigations into a tandem ring-closing metathesis/allylic oxidation sequence^52^ we discovered that Ru-based metathesis catalysts, such as the first generation Grubbs’ catalyst **A**,^53^ are also capable of catalyzing allylic oxidations if *tert*-butyl hydroperoxide is used as an oxidant. For the present task, however, Ru-catalyzed protocols were unsuitable as conversion to the chalcone remained below 5%, regardless of the precatalyst and the reaction time and temperatures (entries 1–6). A similar protocol that uses catalytic amounts of CrO_3_ was also unsuccessful (entry 7),^54^ as was the use of pyridinium chloro chromate (PCC) in large excess (entry 8).^55^ With a catalytic amount of PCC and *tert*-BuOOH as oxidant, however, some conversion to the desired chalcone oxidation product could be detected (entry 9).^54^ No product formation was detected with SeO_2_- (entry 10)^56,57^ and CuI-catalyzed (entry 11)^58^ methods, whereas CuBr as a catalyst led to some conversion (entry 12).^58^ Eventually, a suitable method was identified when the reagent combination of dichlorodicyano benzoquinone (DDQ) and silica under microwave irradiation was tested (entry 13).^59^ This method had been developed by Sinha and co-workers, who originally used ultrasonication to promote the reaction,^60,61^ but later found that microwave irradiation gives higher yields of the oxidation products.^59^ The authors found that the addition of silica notably increases the yield and they reason that this is due to a protonation of the oxidant DDQ.^60^

**Table 3:**
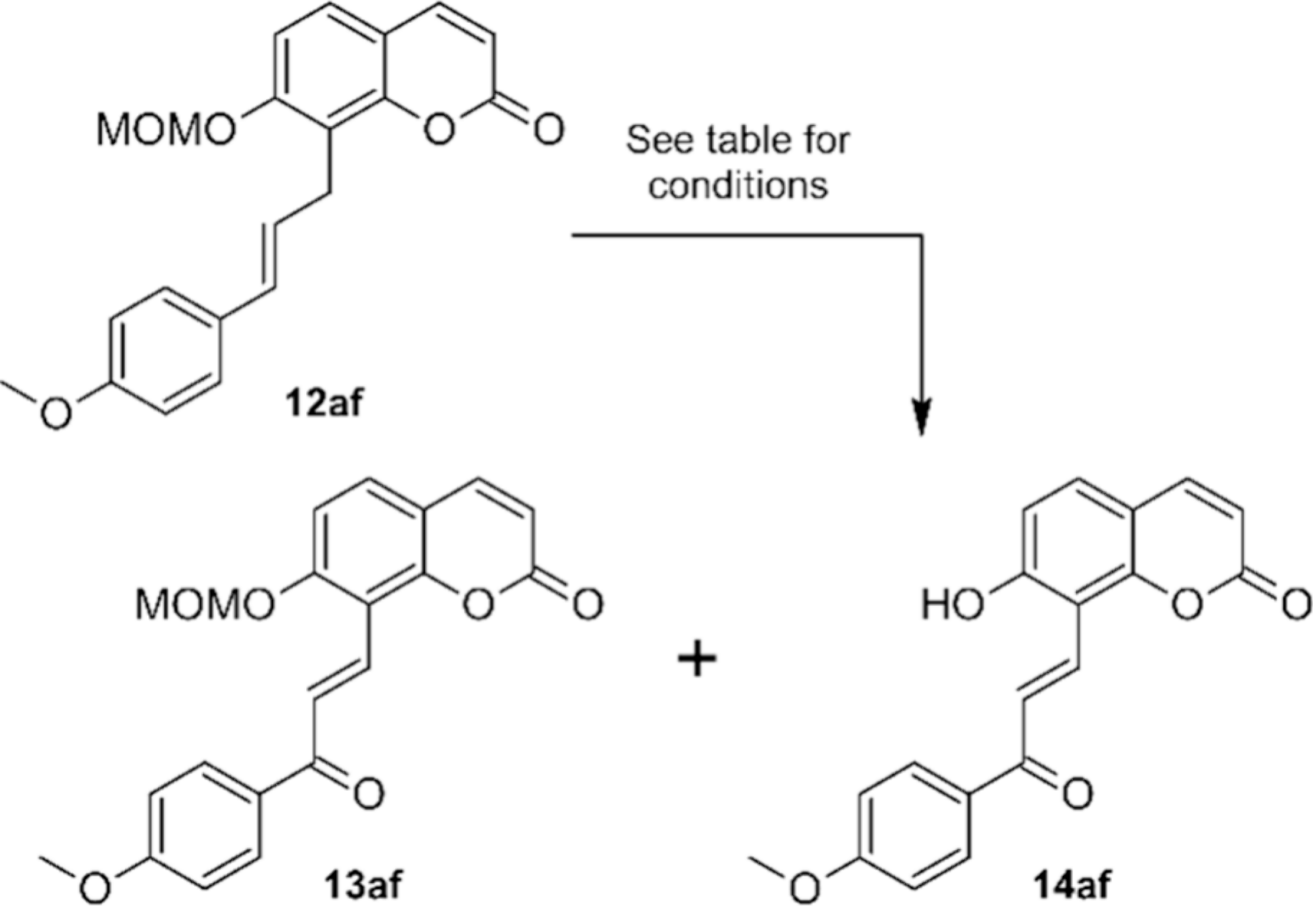
Optimization of Allylic Oxidation Conditions

entry	Conditions^a^	Yield of **13af** (%)
1	[RuPPh_3_)_2_Cl_2_] (5 mol %); *tert*-BuOOH (4 equiv), benzene, 40 °C, 3 h^51^	<5^b^
2	**A** (5 mol %); *tert*-BuOOH (4 equiv), benzene, 40 °C, 3 h^52^	<5^b^
3	[RuPPh_3_)_2_Cl_2_] (5 mol %); *tert*-BuOOH (8 equiv), benzene, 40 °C, 16 h^51^	<5^b^
4	**A** (5 mol %); *tert*-BuOOH (8 equiv), benzene, 40 °C, 16 h^52^	<5^b^
5	[RuPPh_3_)_2_Cl_2_] (5 mol %); *tert*-BuOOH (8 equiv), benzene, 60 °C, 16 h^51^	<5^b^
6	**A** (5 mol %); *tert*-BuOOH (8 equiv), benzene, 60 °C, 16 h^52^	<5^b^
7	CrO_3_ (5 mol %); *tert*-BuOOH (7 equiv), C_6_H_5_CF_3_, 20 °C, 16 h^54^	<5^b^
8	Pyridinium chlorochromate (25 equiv), 4 Å-molecular sieves, benzene, 80 °C, 16 h^55^	<5^b^
9	Pyridinium chlorochromate (10 mol %), *tert*-BuOOH (4 equiv), C_6_H_5_CF_3_, 20 °C, 96 h^54^	<20^c^
10	SeO_2_ (5 mol %), PIDA (5 equiv), dioxane, 100 °C, 16 h^56,57^	<5^b^
11	CuI (2.6 mol %), *tert*-BuOOH (6 equiv), benzene, 70 °C, 24 h.^58^	<5^b^
12	CuBr (2.0 mol %), *tert*-BuOOH (6 equiv), acetonitrile, 55 °C, 24 h^58^	<20^c^
13	DDQ, silica, dioxane, microwave irradiation @90 °C, 25 min^59^	78^d^

a Method and conditions adapted from this reference.

b Conversion <5% (TLC), unreacted starting material recovered.

c Conversion <20% (^1^H NMR-spectroscopy).

d Isolated along with ca. 20% of **14af**.

With these conditions we were able to isolate the chalcone–coumarin hybrid **13af** in 78% yield. Minor quantities of a second, slightly more polar product were obtained in impure form, contaminated with small amounts of **13af**. This side product was identified as the deprotected oxidation product **14af**. Cleavage of acetals, e.g. THP ethers, in the presence of catalytic amounts of DDQ has been reported,^62,63^ although in this case the acidic silica might also be responsible for the observed partial MOM-ether cleavage.

**Figure 4:**
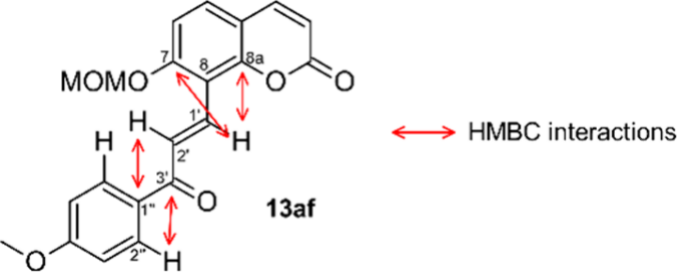
HMBC interactions in **13af**.

The allylic oxidation step can proceed either through a radical or a hydride abstraction. In both cases a resonance stabilized intermediate (either an allyl radical or a carbenium ion) will be formed, which can then react with or without net-transposition of the C–C-double bond to the oxidation product. Thus, not only **13af**, but also its regioisomer, with the carbonyl group bound to the coumarin part of the hybrid structure, are possible oxidation products. We obtained just one regioisomer with very high selectivity, but similar to the Heck coupling products **12** the structure could not be unambiguously assigned solely based on 1D-NMR spectra. For this reason, a full signal assignment based on the 2D-NMR methods H,H–COSY, HSQC, HMBC and NOESY was performed. While no significant NOE-interactions could be observed for protons H1′ and H2′, HMBC interactions between H1′ and C7 and C8a, between H2′ and C1″, and between H2″ and C3′ (the chalcone carbonyl carbon) clearly point at the structure shown for **13af** in Figure 4.

The optimized allylic oxidation conditions (Table 3, entry 13) were first applied to the other C7-OMOM-substituted Matsuda–Heck coupling products **12a#**. In most cases the allylic oxidation proceeds with high regioselectivity (Table 4). For the oxidation products of **12aa** (enry 1), **12am** (entry 12), **12aq** (entry 16), **12ar** (entry 17) and **12au** (entry 19) the complete signal assignment process outlined above was repeated to check whether the regioselectivity of the allylic oxidation is affected by the substitution pattern of the aromatic substituent bound at C3′. In all cases single regioisomers with a C1′=C2′-double bond and a carbonyl group at C3′-position, as for **13af** (entry 6), were obtained. While we observed cleavage of the MOM-group only to a small extent for this example and for the *para*-iodo-substituted derivative **13ae**, concomitant deprotection occurred quantitatively during the oxidation of the *meta*-bromo-substituted compound **12an**, the *meta*-carbomethoxy-substituted starting material **12ar**, and the *ortho*-carbomethoxy-substituted precursor **12au**. These reactions furnished exclusively phenols **14an** (entry 13), **14ar** (entry 17) and **14au** (entry 19). Quantitative decomposition was observed during oxidation of phenol-substituted compound **12ag** (entry 7), and partial decomposition for iodo-substituted coupling product **12ao** (entry 14) and *para*-methyl-substituted coupling product **12ah** (entry 8). In these cases the coumarin–chalcone hybrids were obtained as inseparable mixtures with other unidentified reaction products.

**Table 4:**
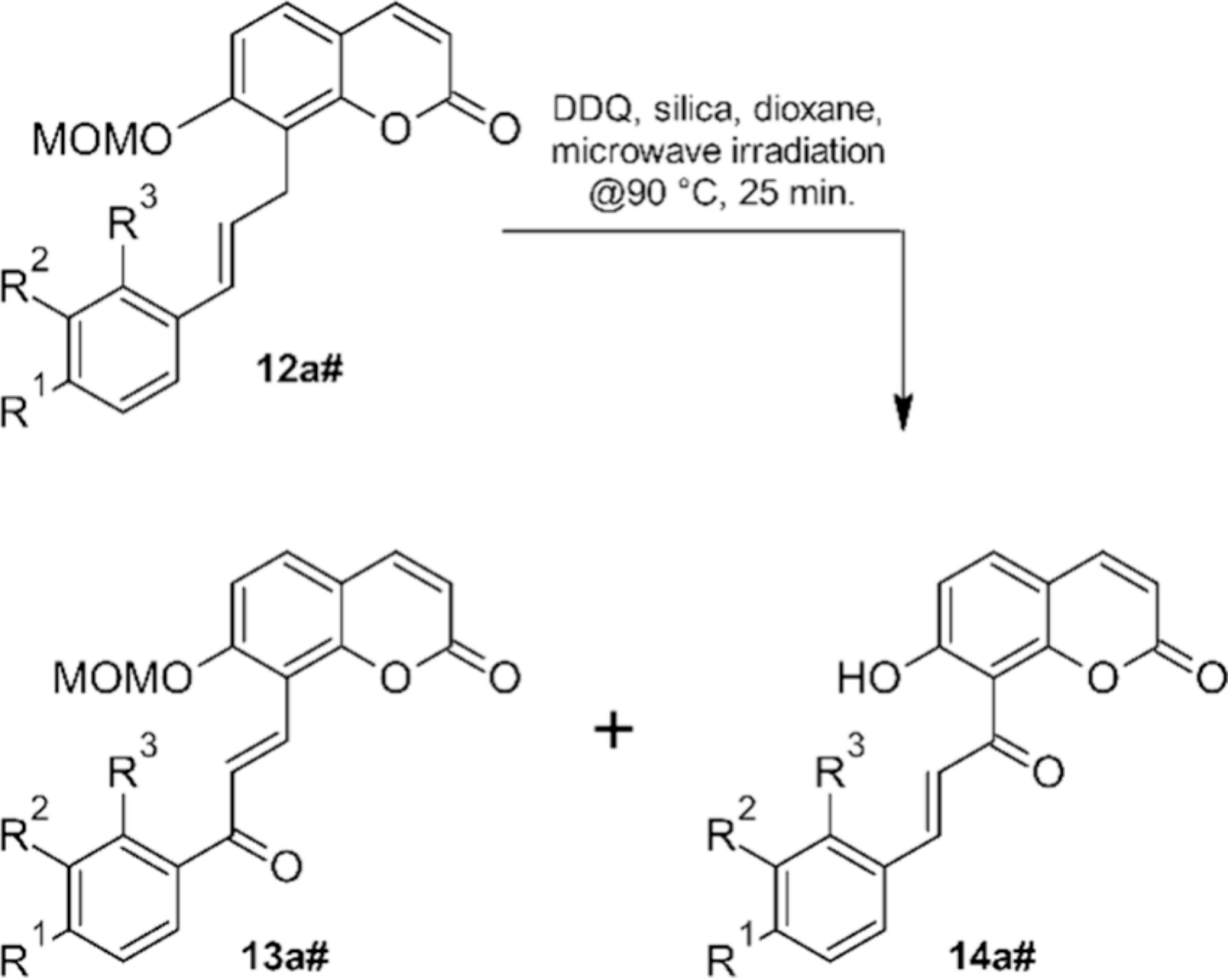
Allylic Oxidation of **12a#**: Scope and Limitations

entry	**12**	R^1^	R^2^	R^3^	Product	yield (%)
1	**12aa**	H	H	H	**13aa**	56
2	**12ab**	F	H	H	**13ab**	85
3	**12ac**	Cl	H	H	**13ac**	47
4	**12ad**	Br	H	H	**13ad**	43
5	**12ae**	I	H	H	**13ae**	29^a^
6	**12af**	OCH_3_	H	H	**13af**	78^a^
7	**12ag**	OH	H	H	**13ag**	<5^b^
8	**12ah**	CH_3_	H	H	**13ah**	n.d.^c^
9	**12ai**	CO_2_Et	H	H	**13ai**	36
10	**12ak**	CF_3_	H	H	**13ak**	22
11	**12al**	H	F	H	**13al**	33
12	**12am**	H	Cl	H	**13am**	60
13	**12an**	H	Br	H	**14an**	26
14	**12ao**	H	I	H	**13ao**	n.d.^c^
15	**12ap**	H	OCH_3_	H	**13ap**	24
16	**12aq**	H	CH_3_	H	**13aq**	37
17	**12ar**	H	CO_2_CH_3_	H	**14ar**	48
18	**12at**	H	H	OCH_3_	**13at**	56
19	**12au**	H	H	CO_2_CH_3_	**14au**	92

a Unprotected phenols **14ae** (16%) and **14af** (ca. 10%) were isolated as byproducts.

b Decomposition of starting material.

c n.d.: not determined; oxidation products **13** were detected by NMR but could not be isolated in pure form due to inseparable byproducts.

**Figure 5:**
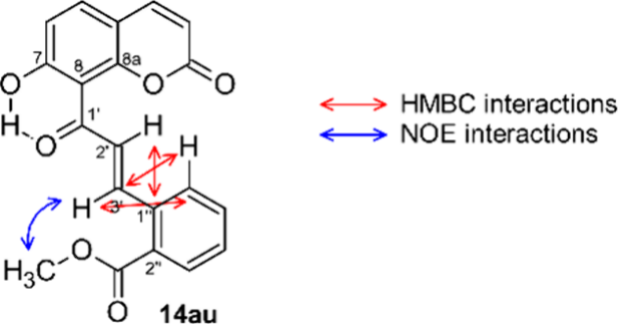
HMBC interactions in **14au**.

Upon careful inspection of the NMR-spectra of oxidation products **14** (free phenols) we noted some significant differences compared to the spectra of chalcones **13** (MOM-ethers). In particular, an unusually high δ(^13^C) value of ca. 167 ppm was detected for carbon atoms C7 in all oxidation products **14**. This prompted us to double check the assigned chalcone structure through a full signal assignment based on 2D-NMR-experiments. To our surprise, we did not detect the characteristic HMBC-correlations that are indicative for the C1′=C2′-double bond present in MOM-ethers **13**, but found instead HMBC-interactions that point at a double bond located between positions C2′ and C3′, as shown in Figure 5 for the representative example **14au**. It is not clear why the regioselectivity of the allylic oxidation is reversed in these cases, but it appears likely that cleavage of the MOM-ether occurs prior to the allylic oxidation. The phenol thus generated at C7 then directs the oxidant to the C1′-position, probably through hydrogen bond formation. In contrast, the selective C3′-oxidation observed in MOM-ethers **13** could originate from steric hindrance.

### Application to Flavanone–Chalcone Hybrids

**Figure 6:**
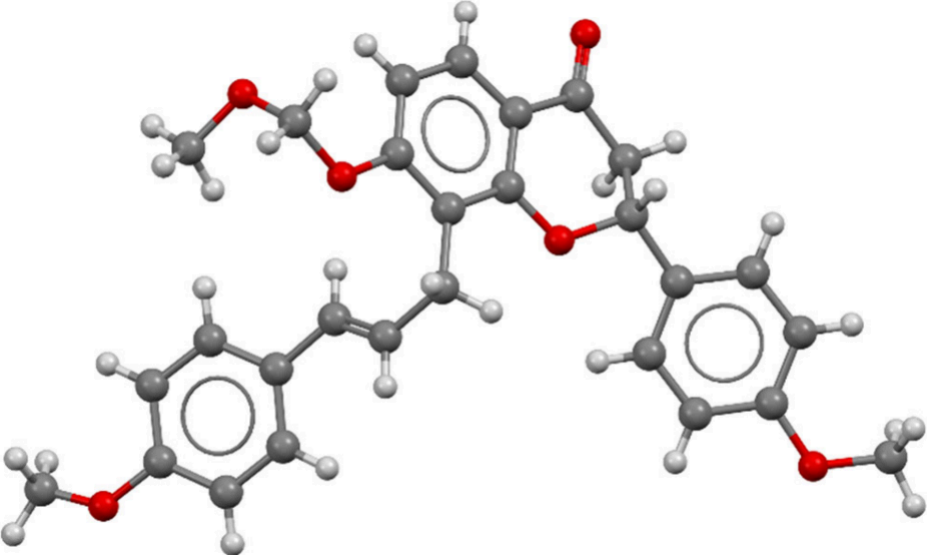
Molecular structure of coupling product **16cf**. Displacement ellipsoids are shown at the 50% probability level.

**Scheme 5:**
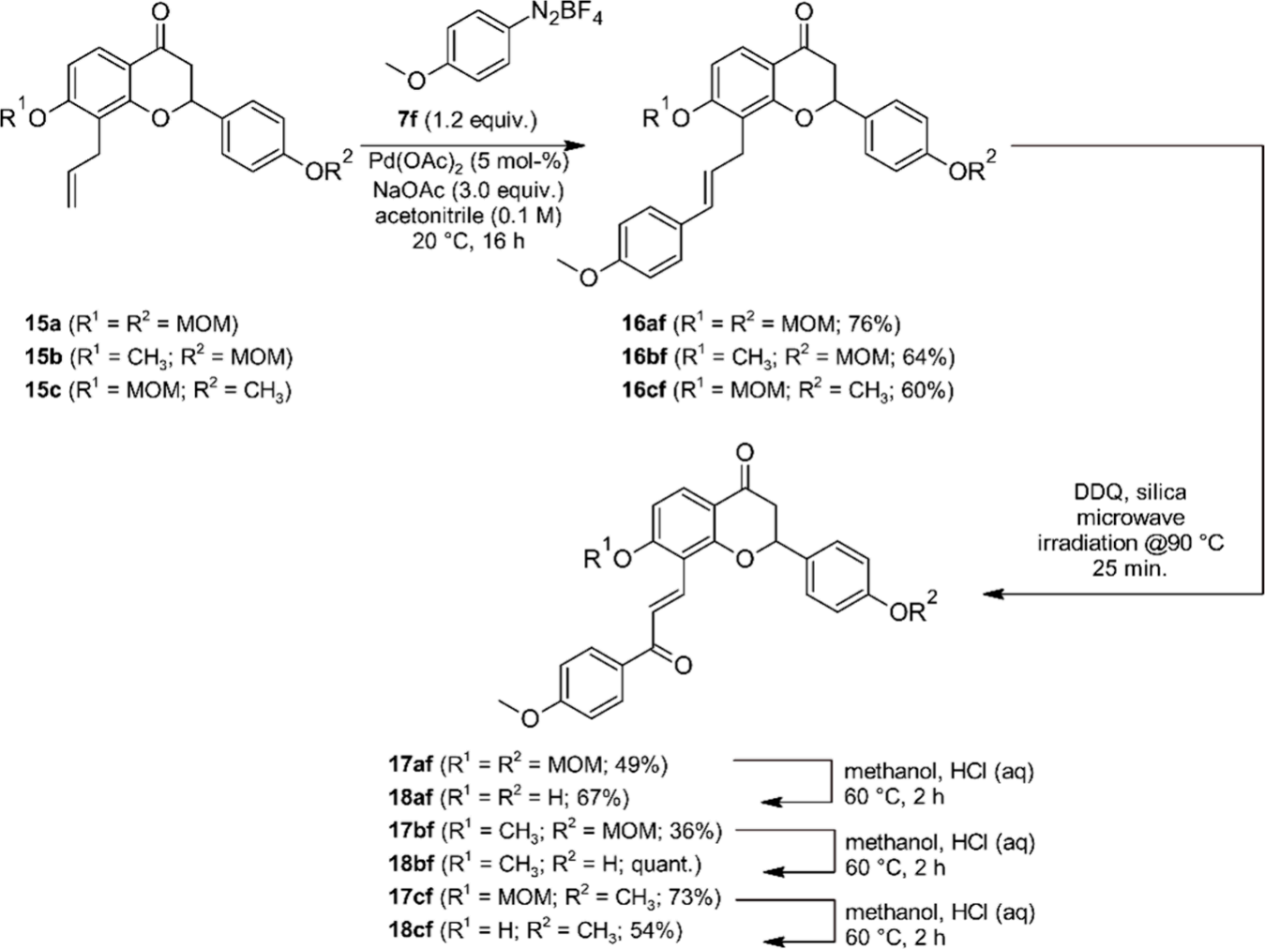
Synthesis of Flavanone–Chalcone Hybrids **18**

The Matsuda–Heck-arylation/allylic oxidation sequence for the synthesis of coumarin–chalcone hybrids can be applied to other natural product scaffolds. We first investigated the synthesis of flavanone–chalcone hybrids **18**, which started from 8-allylflavanones **15**. Flavanones **15a** and **15c** are known compounds that were previously used by us for the synthesis of prenylated flavanones.^64^ Flavanone **15b** was synthesized through an analogous sequence of steps (see Supporting Information for details). By using the standard conditions from Table 1, entry 3, three coupling products **16af**, **16bf** and **16cf** were obtained from 8-allylflavanones **15a**-**c** and diazonium salt **7f** in yields comparable to those obtained for the coupling reactions of 8-allylcoumarin **11a** (Scheme 5). All products **16** were obtained as single regioisomers with a C2′=C3′-double bond, which was proven by 2D-NMR methods via a line of reasoning analogous to that described for the coumarin coupling products **12** (see Supporting Information for a detailed chart). In the case of **16cf** the assigned structure (and hence the selectivity of the Matsuda–Heck-reaction) was independently corroborated by single-crystal X-ray analysis (Figure 6).

All coupling products **16** underwent the allylic oxidation, using the DDQ-silica reagent under microwave irradiation, in fair yields and high regioselectivity to furnish MOM-protected flavanone-chalcone hybrids **17**. Cleavage of the MOM-ether was accomplished with methanol in the presence of acid to furnish the phenolic hybrid compounds **18** (Scheme 5).

### Application to Isoflavone–Chalcone Hybrids

**Scheme 6:**
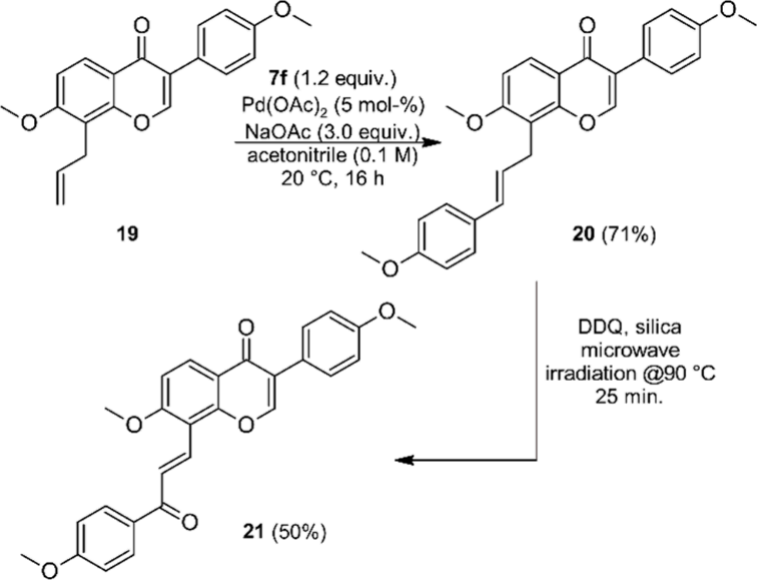
Synthesis of Isoflavone-A-Ring-Chalcone-Hybrid **21**

For the synthesis of an isoflavone–chalcone hybrid with the chalcone connected to the isoflavone-A-ring we started from 8-allyl isoflavone **19**. This is an intermediate en route to the prenylated isoflavone natural product 7-methoxyebenosin and was previously synthesized by us via an oxidative flavanone rearrangement.^65^ Matsuda–Heck arylation under the established standard conditions furnished **20** as a single regioisomer, and oxidation with DDQ-silica gave the isoflavone–chalcone hybrid **21** (Scheme 6). As discussed above for the coumarin– and flavanone–chalcone hybrids, structure elucidation is based on 2D-NMR-experiments, in particular HMBC (see Supporting Information for details).

**Scheme 7:**
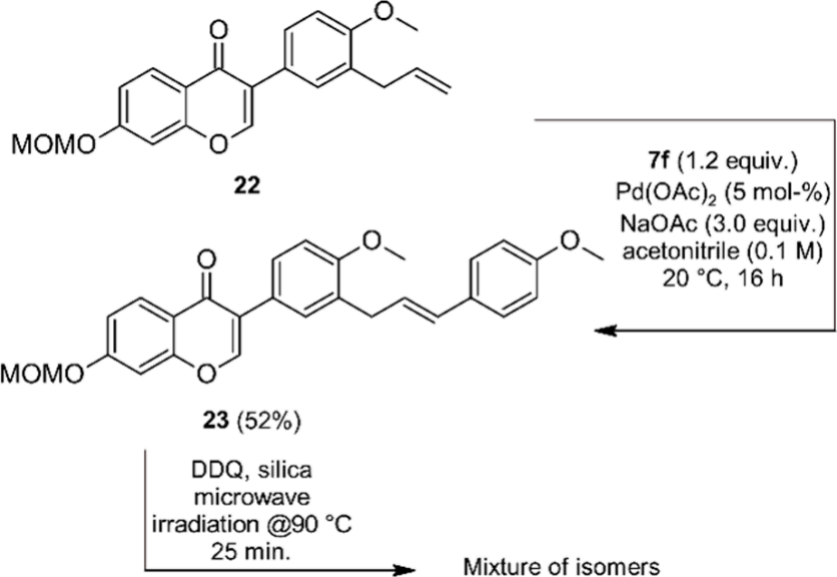
Attempted Synthesis of an Isoflavone-B-Ring-Chalcone-Hybrid **23**

We also investigated the application of our approach to the synthesis of an isoflavone-chalcone hybrid with the chalcone moiety attached to the B-ring (Scheme 7). To this end isoflavone **22**, an intermediate in our synthesis of the natural product 5-deoxy-3′-prenylbiochanin A^66^ was used as a starting material and reacted with diazonium salt **7f** under the standard conditions. Compared to other allylarenes from this study that underwent coupling reactions with high β-selectivity, **22** is sterically less congested and lacks a second coordinating group adjacent to the allyl chain. Nevertheless, coupling product **23** was obtained in fair yield and high β-selectivity. Allylic oxidation under the established standard conditions proceeded with full conversion of the starting material, but resulted in the formation of an inseparable mixture of isomers. This observation suggests that a synthetically useful regioselectivity of the allylic oxidation step requires a strong steric and/or chelating bias, and that the presence of just one adjacent substituent is not sufficient in this regard.

## Conclusions

In summary, we showed that electronically nonbiased olefins, such as allyl benzenes, undergo Pd-catalyzed arylation reactions in high regioselectivity if two potentially coordinating substituents are present at the positions adjacent to the allyl group. We reason that the regioselectivity of the β-hydride elimination step arises from a chelation of the Pd-σ-complex, that forces one β-hydrogen into an orientation that is geometrically unfavorable for the β-H-elimination. The subsequent allylic oxidation also occurs in high selectivity, but with transposition of the C=C-double bond. Most likely steric reasons are responsible for the observed regioselectivity of this step. The sequence of Pd-catalyzed arylation with arene diazonium salts and allylic oxidation was applied in the synthesis of various chalcone hybrids.

## Experimental Section

### General Methods

All experiments were conducted in dry reaction vessels under an atmosphere of dry nitrogen. Solvents were purified by standard procedures. Unless otherwise stated, reaction mixtures were heated with silicon oil baths. Microwave reactions were carried out in sealed vials in an Anton-Paar-monowave 300 or Anton-Paar-monowave 400 reactor (monowave, maximum power 850 W, temperature of reaction mixture and temperature–time profile were monitored by IR-sensor, vial volume 10 mL). ^1^H NMR spectra were obtained at 400 MHz in CDCl_3_ with C*H*Cl_3_ (δ = 7.26 ppm) as an internal standard. Coupling constants are given in Hz. ^13^C{^1^H} NMR spectra were recorded at 100 MHz in CDCl_3_ with CDCl_3_ (δ = 77.1 ppm) as an internal standard. Whenever the solubility of the sample was insufficient in CDCl_3_, it was replaced by either acetone-*d*_6_ (acetone-*d*_5_ as internal standard for ^1^H NMR spectroscopy, δ = 2.05 ppm, acetone-*d*_6_ as internal standard for ^13^C{^1^H} NMR spectroscopy, δ = 29.9 ppm) or DMSO-*d*_6_ (DMSO-*d*_5_ as internal standard for ^1^H NMR spectroscopy, δ = 2.50 ppm, DMSO-*d*_6_ as internal standard for ^13^C{^1^H} NMR spectroscopy, δ = 39.5 ppm). Structural assignments were made with additional information from gNOESY, gCOSY, gHSQC, and gHMBC experiments. Signal assignments refer to the numbering scheme shown in Figure 1. IR spectra were recorded as ATR-FTIR spectra. Wavenumbers are given in cm^–1^. The peak intensities are defined as strong (s), medium (m) or weak (w). Low and high resolution mass spectra were obtained by EI-TOF or ESI-TOF. Chromatographic purification of compounds was accomplished using the dry column vacuum chromatography (DCVC) method as described in the literature.^67^

***Caution***: arene diazonium salts with tetrafluoroborate counterions are in most cases very stable. We have never experienced any safety incidents while working with these reagents, but there have been reports describing explosions or violent decomposition reactions caused by some arene diazonium tetrafluoroborates. We urgently recommend that operators familiarize themselves with the potential hazards and recommended safety measures as e.g. suggested by Firth and Fairlamb^68^ and by Correia and co-workers.^69^

### General Procedure for the Coupling of Allylbenzopyranones and Arene Diazonium Salts

To a solution of the respective arene diazonium salt **7** (1.20 mmol, 1.20 equiv) in acetonitrile (10 mL, 0.1 M in olefin **11**) was added Pd(OAc)_2_ (11.2 mg, 5.0 mol %). The mixture was stirred at 20 °C for 5 min, and NaOAc (246 mg, 3.00 mmol, 3.00 equiv) and the respective allylbenzopyranone **11** (1.00 mmol, 1.00 equiv) were added. The mixture was stirred at 20 °C for 16 h, filtered through a short silica-Celite pad which was washed with MTBE (20 mL). All volatiles were evaporated, and the residue was purified by flash chromatography on silica, using hexanes–ethyl acetate mixtures of increasing polarity as eluent, to furnish the coupling products.

#### 7-(Methoxymethoxy)-8-[(2*E*)-3-phenylprop-2-en-1-yl]-2*H*-chromen-2-one (**12aa**)

Obtained from **7a** (58 mg, 0.30 mmol) and **11a** (61 mg, 0.25 mmol) as an off-white solid; eluent for chromatography: hexanes–ethyl acetate mixtures of increasing polarity (9:1 (v/v) to 5:1 (v/v)); yield: 60 mg (0.19 mmol, 74%); mp 105–106 °C; IR (ATR) ν 2906 (w), 1714 (s), 1601 (s), 1492 (m), 1246 (s), 1018 (s) cm^–1^; ^1^H NMR (400 MHz, CDCl_3_) δ 7.63 (d, *J* = 9.5 Hz, 1H), 7.33–7.29 (m, 3H), 7.25 (t, *J* = 7.4 Hz, 2H), 7.16 (tt, *J* = 7.3, 1.3 Hz, 1H), 7.07 (d, *J* = 8.7 Hz, 1H), 6.51 (d, *J* = 15.8 Hz, 1H), 6.34 (dt, *J* = 15.8, 6.6 Hz, 1H), 6.27 (d, *J* = 9.5 Hz, 1H), 5.31 (s, 2H), 3.78 (d, *J* = 6.5 Hz, 2H), 3.49 (s, 3H); ^13^C{^1^H} NMR (100 MHz, CDCl_3_) δ 161.2, 158.0, 153.1, 143.8, 137.6, 131.0, 128.5, 127.1, 127.0, 126.7, 126.1, 117.0, 113.7, 113.6, 110.6, 94.4, 56.5, 26.3; HRMS (EI) *m*/*z* [M]^+^ calcd for C_20_H_18_O_4_ 322.1205, found 322.1215.

#### 8-[(2*E*)-3-(4-Fluorophenyl)prop-2-en-1-yl]-7-(methoxymethoxy)-2*H*-chromen-2-one (**12ab**)

Obtained from **7b** (63 mg, 0.30 mmol) and **11a** (61 mg, 0.25 mmol) as an off-white solid; eluent for chromatography: hexanes–ethyl acetate mixtures of increasing polarity (9:1 (v/v) to 5:1 (v/v)); yield: 57 mg (0.17 mmol, 67%); mp 110–112 °C; IR (ATR) ν 2930 (w), 1722 (s), 1605 (s), 1508 (m), 1116 (m), 1058 (m) cm^–1^; ^1^H NMR (400 MHz, CDCl_3_) δ 7.64 (d, *J* = 9.5 Hz, 1H), 7.31 (d, *J* = 8.7 Hz, 1H), 7.26 (dd, *J* = 8.5, 5.9 Hz, 2H), 7.07 (d, *J* = 8.7 Hz, 1H), 6.93 (t, *J* = 8.5 Hz, 2H), 6.47 (d, *J* = 15.8 Hz, 1H), 6.28 (d, *J* = 9.5 Hz, 1H), 6.25 (dt, *J* = 15.9, 6.6 Hz, 1H), 5.31 (s, 2H), 3.76 (dd, *J* = 6.6, 0.8 Hz, 2H), 3.48 (s, 3H); ^13^C{^1^H} NMR (100 MHz, CDCl_3_) δ 162.0 (d, *J* = 245.9 Hz), 161.1, 158.0, 153.1, 143.9, 133.8 (d, *J* = 3.4 Hz), 129.8, 127.6 (d, *J* = 7.9 Hz), 126.8, 126.6 (d, *J* = 2.3 Hz), 117.0, 115.4 (d, *J* = 21.5 Hz), 113.8, 113.7, 110.6, 94.5, 56.5, 26.3; HRMS (EI) *m*/*z* [M^+^] calcd for C_20_H_17_O_4_F 340.1111, found 340.1114.

#### 8-[(2*E*)-3-(4-Chlorophenyl)prop-2-en-1-yl]-7-(methoxymethoxy)-2*H*-chromen-2-one (**12ac**)

Obtained from **7c** (68 mg, 0.30 mmol) and **11a** (61 mg, 0.25 mmol) as a yellowish solid; eluent for chromatography: hexanes–ethyl acetate mixtures of increasing polarity (9:1 (v/v) to 5:1 (v/v)); yield: 65 mg (0.18 mmol, 73%); mp 108–110 °C; IR (ATR) ν 2957 (w), 1723 (s), 1606 (s), 1491 (m), 1116 (m), 1058 (m) cm^–1^; ^1^H NMR (400 MHz, CDCl_3_) δ 7.64 (d, *J* = 9.5 Hz, 1H), 7.31 (d, *J* = 8.7 Hz, 1H), 7.25–7.18 (4H), 7.08 (d, *J* = 8.6 Hz, 1H), 6.44 (d, *J* = 15.8 Hz, 1H), 6.31 (dt, *J* = 15.8, 6.4 Hz, 1H), 6.28 (d, *J* = 9.5 Hz, 1H), 5.31 (s, 2H), 3.77 (dd, *J* = 6.4, 0.7 Hz, 2H), 3.48 (s, 3H); ^13^C{^1^H} NMR (100 MHz, CDCl_3_) δ 161.3, 158.0, 153.1, 143.8, 136.2, 132.7, 129.8, 128.7, 127.8, 127.4, 126.9, 116.8, 113.8, 113.8, 110.6, 94.4, 56.5, 26.4; HRMS (EI) *m*/*z* [M^+^] calcd for C_20_H_17_O_4_^35^Cl 356.0815, found 356.0805.

#### 8-[(2*E*)-3-(4-Bromophenyl)prop-2-en-1-yl]-7-(methoxymethoxy)-2*H*-chromen-2-one (**12ad**)

Obtained from **7d** (81 mg, 0.30 mmol) and **11a** (61 mg, 0.25 mmol) as a yellowish solid; eluent for chromatography: hexanes–ethyl acetate mixtures of increasing polarity (9:1 (v/v) to 5:1 (v/v)); yield: 76 mg (0.19 mmol, 75%); mp 100–101 °C; IR (ATR) ν 2956 (w), 1722 (s), 1606 (s), 1488 (m), 1115 (m), 1054 (m) cm^–1^; ^1^H NMR (400 MHz, CDCl_3_) δ 7.64 (d, *J* = 9.5 Hz, 1H), 7.36 (d, *J* = 8.5 Hz, 2H), 7.32 (d, *J* = 8.7 Hz, 1H), 7.16 (d, *J* = 8.5 Hz, 2H), 7.08 (d, *J* = 8.7 Hz, 1H), 6.43 (d, *J* = 15.9 Hz, 1H), 6.33 (dt, *J* = 15.7, 6.1 Hz, 1H), 6.28 (d, *J* = 9.5 Hz, 1H), 5.31 (s, 2H), 3.76 (d, *J* = 6.0 Hz, 2H), 3.47 (s, 3H); ^13^C{^1^H} NMR (100 MHz, CDCl_3_) δ 161.2, 158.0, 153.1, 143.8, 136.6, 131.6, 129.8, 127.9, 127.7, 126.9, 120.7, 116.7, 113.8, 113.7, 110.6, 94.4, 56.5, 26.4; HRMS (EI) *m*/*z* [M^+^] calcd for C_20_H_17_O_4_^79^Br 400.0310, found 400.0311.

#### 8-[(2*E*)-3-(4-Iodophenyl)prop-2-en-1-yl]-7-(methoxymethoxy)-2*H*-chromen-2-one (**12ae**)

Obtained from **7e** (95 mg, 0.30 mmol) and **11a** (61 mg, 0.25 mmol) as a yellowish solid; eluent for chromatography: hexanes–ethyl acetate mixtures of increasing polarity (9:1 (v/v) to 5:1 (v/v)); yield: 68 mg (0.15 mmol, 61%); mp 122–124 °C; IR (ATR) ν 2955 (w), 1720 (s), 1605 (s), 1483 (m), 1115 (m), 1059 (m) cm^–1^; ^1^H NMR (400 MHz, CDCl_3_) δ 7.64 (d, *J* = 9.5 Hz, 1H), 7.56 (d, *J* = 8.4 Hz, 2H), 7.32 (d, *J* = 8.7 Hz, 1H), 7.07 (d, *J* = 8.7 Hz, 1H), 7.04 (d, *J* = 8.4 Hz, 2H), 6.41 (d, *J* = 15.9 Hz, 1H), 6.34 (dt, *J* = 15.9, 5.5 Hz, 1H), 6.28 (d, *J* = 9.5 Hz, 1H), 5.31 (s, 2H), 3.76 (d, *J* = 5.5 Hz, 1H), 3.47 (s, 3H); ^13^C{^1^H} NMR (100 MHz, CDCl_3_) δ 161.3, 158.0, 153.1, 143.9, 137.6, 137.2, 130.0, 128.1, 128.0, 126.9, 116.7, 113.9, 113.8, 110.6, 94.4, 92.2, 56.5, 26.4; HRMS (EI) *m*/*z* [M^+^] calcd for C_20_H_17_O_4_^127^I 448.0172, found 448.0158.

#### 7-(Methoxymethoxy)-8-[(2*E*)-3-(4-methoxyphenyl)prop-2-en-1-yl]-2*H*-chromen-2-one (**12af**)

Obtained from **7f** (67 mg, 0.30 mmol) and **11a** (61 mg, 0.25 mmol) as a yellowish solid; eluent for chromatography: hexanes–ethyl acetate mixtures of increasing polarity (9:1 (v/v) to 5:1 (v/v)); yield: 70 mg (0.20 mmol, 79%); mp 100–105 °C; IR (ATR) ν 2956 (w), 1727 (s), 1607 (s), 1511 (m), 1247 (s), 1060 (m) cm^–1^; ^1^H NMR (400 MHz, CDCl_3_) δ 7.62 (d, *J* = 9.5 Hz, 1H, H4), 7.29 (d, *J* = 8.7 Hz, 1H, H5), 7.23 (d, *J* = 8.7 Hz, 2H, H2″/H6″), 7.23 (d, *J* = 8.7 Hz, 2H), 7.06 (d, *J* = 8.7 Hz, 1H, H6), 6.78 (d, *J* = 8.8 Hz, 2H), 6.45 (d, *J* = 15.8 Hz, 1H, H3′), 6.26 (d, *J* = 9.5 Hz, 1H, H3), 6.20 (dt, *J* = 15.7, 6.7 Hz, 1H, H2′), 5.30 (s, 2H, −OC*H*_2_O−), 3.77 (s, 3H), 3.76 (dd, *J* = 6.6, 1.2 Hz, 1H, H1′), 3.48 (s, 3H, −OCH_2_OC*H*_3_); ^13^C{^1^H} NMR (100 MHz, CDCl_3_) δ 161.3 (C2), 158.8 (C4″), 158.0 (C7), 153.0 (C8a), 143.8 (C4), 130.5 (C1″), 130.3 (C3′), 127.2 (C2″/C6″), 126.6 (C5), 124.8 (C2′), 117.3 (C8), 113.9 (C3″/C5″), 113.7 (C4a), 113.6 (C3), 110.6 (C6), 94.4 (*−OC*H_2_OCH_3_), 56.5 (−OCH_2_O*C*H_3_), 55.3 (C4″*–OC*H_3_), 26.3 (C1′); HRMS (EI) *m*/*z* [M^+^] calcd for C_21_H_20_O_5_ 352.1311, found 352.1325.

#### 8-[(2*E*)-3-(4-Hydroxyphenyl)prop-2-en-1-yl]-7-(methoxymethoxy)-2*H*-chromen-2-one (**12ag**)

Obtained from **7g** (250 mg, 1.20 mmol) and **11a** (246 mg, 1.00 mmol) as a yellowish solid; eluent for chromatography: hexanes–ethyl acetate mixtures of increasing polarity (9:1 (v/v) to 5:1 (v/v)); yield: 179 mg (0.53 mmol, 53%); mp 105–107 °C; IR (ATR) ν 3346 (w), 2932 (w), 1698 (s), 1604 (s), 1512 (m), 1247 (m), 1065 (m), 835 (m) cm^–1^; ^1^H NMR (400 MHz, CDCl_3_) δ 7.64 (d, *J* = 9.5 Hz, 1H), 7.38 (d, *J* = 8.7 Hz, 1H), 7.17 (d, *J* = 8.5 Hz, 2H), 7.08 (d, *J* = 8.6 Hz, 1H), 6.73 (d, *J* = 8.5 Hz, 2H), 6.42 (d, *J* = 15.8 Hz, 1H), 6.28 (d, *J* = 9.5 Hz, 1H), 6.17 (dt, *J* = 15.8, 6.7 Hz, 1H), 5.31 (s, 2H), 3.72 (dd, *J* = 6.7, 1.0 Hz, 2H), 3.48 (s, 3H); ^13^C{^1^H} NMR (100 MHz, CDCl_3_) δ 162.2, 158.1, 155.5, 153.0, 144.4, 130.5, 130.1, 127.4, 126.8, 124.2, 117.5, 115.5, 113.8, 113.4, 110.8, 94.4, 56.6, 26.3; HRMS (EI) *m*/*z* [M^+^] calcd for C_20_H_18_O_5_ 338.1154, found 338.1132.

#### 7-(Methoxymethoxy)-8-[(2*E*)-3-(4-methylphenyl)prop-2-en-1-yl]-2*H*-chromen-2-one (**12ah**)

Obtained from **7h** (62 mg, 0.30 mmol) and **11a** (61 mg, 0.25 mmol) as a yellowish solid; eluent for chromatography: hexanes–ethyl acetate mixtures of increasing polarity (9:1 (v/v) to 5:1 (v/v)); yield: 60 mg (0.18 mmol, 71%); mp 119–121 °C; IR (ATR) ν 2920 (w), 1715 (s), 1601 (s), 1492 (m), 1246 (m) cm^–1^; ^1^H NMR (400 MHz, CDCl_3_) δ 7.63 (d, *J* = 9.5 Hz, 1H, H4), 7.30 (d, *J* = 8.7 Hz, 1H, H5), 7.20 (d, *J* = 8.1 Hz, 2H, H2″), 7.07 (d, *J* = 8.8 Hz, 1H, H6), 7.05 (d, *J* = 8.1 Hz, 2H, H3″), 6.47 (d, *J* = 15.8 Hz, 1H, H3′), 6.29 (dt, *J* = 15.7, 6.6 Hz, 1H, H2′), 6.27 (d, *J* = 9.5 Hz, 1H, H3), 5.31 (s, 2H, −OC*H*_2_O−), 3.77 (dd, *J* = 6.7, 0.8 Hz, 2H, H1′), 3.48 (s, 3H, −OC*H*_3_), 2.29 (s, 3H, −C4″C*H*_3_); ^13^C{^1^H} NMR (100 MHz, CDCl_3_) δ 161.2 (C2), 158.0 (C7), 153.1 (C8a), 143.8 C4), 136.8 (C4″), 134.9 (C1″), 130.9 (C3′), 129.2 (C3″), 126.7 (C5), 126.0 (C2″), 125.9 (C2′), 117.3 (C8), 113.7 (C4a), 113.6 (C3), 110.6 (C6), 94.5 (*−OC*H_2_OCH_3_), 56.5 (−OCH_2_O*C*H_3_), 26.3 (C1′), 21.2 (C4″–*C*H_3_); HRMS (EI) *m*/*z* [M^+^] calcd for C_21_H_20_O_4_ 336.1362, found 336.1354.

#### Ethyl 4-{(1*E*)-3-[7-(Methoxymethoxy)-2-oxo-2*H*-chromen-8-yl]prop-1-en-1-yl}benzoate (**12ai**)

Obtained from **7i** (79 mg, 0.30 mmol) and **11a** (61 mg, 0.25 mmol) as a yellowish solid; eluent for chromatography: hexanes–ethyl acetate mixtures of increasing polarity (9:1 (v/v) to 5:1 (v/v)); yield: 78 mg (0.20 mmol, 79%); mp 75–77 °C; IR (ATR) ν 2980 (w), 1710 (s), 1605 (s), 1272 (s), 1107 (s) cm^–1^; ^1^H NMR (400 MHz, CDCl_3_) δ 7.92 (d, *J* = 8.4 Hz, 2H, H3″) 7.64 (d, *J* = 9.5 Hz, 1H, H4), 7.34 (d, *J* = 8.3 Hz, 2H, H2″) 7.32 (d, *J* = 8.6 Hz, 1H, H5), 7.08 (d, *J* = 8.6 Hz, 1H, H6), 6.52 (d, *J* = 15.8 Hz, 1H, H3′), 6.46 (dt, *J* = 15.8, 5.6 Hz, 1H, H2′), 6.28 (d, *J* = 9.5 Hz, 1H, H3), 5.31 (s, 2H, −OC*H*_2_O−), 4.34 (q, *J* = 7.1 Hz, 2H, −CO_2_C*H*_2_CH_3_), 3.80 (d, *J* = 5.5 Hz, 2H, H1′), 3.47 (s, 3H, −OC*H*_3_), 1.37 (t, *J* = 7.1 Hz, 3H, −CO_2_CH_2_C*H*_3_); ^13^C{^1^H} NMR (100 MHz, CDCl_3_): δ = 166.6 (−*C*O_2_CH_2_CH_3_), 161.2 (C2), 158.0 (C7), 153.1 (C8a), 143.8 (C4), 142.1 (C1″), 130.2 (C3′), 130.0 (C3″), 130.0 (C2′), 128.9 (C4″), 126.9 (C5), 126.0 (C2″), 116.5 (C8), 113.8 (C4a), 113.7 (C3), 110.6 (C6), 94.4 (*−OC*H_2_OCH_3_), 60.9 (−CO_2_*C*H_2_CH_3_), 56.5 (−OCH_2_O*C*H_3_), 26.5 (C1′), 14.4 (−CO_2_CH_2_*C*H_3_); HRMS (EI) *m*/*z* [M^+^] calcd for C_23_H_22_O_6_ 394.1416, found 394.1408.

#### 7-(Methoxymethoxy)-8-{(2*E*)-3-[4-(trifluoromethyl)phenyl]prop-2-en-1-yl}-2*H*-chromen-2-one (**12ak**)

Obtained from **7k** (78 mg, 0.30 mmol) and **11a** (61 mg, 0.25 mmol) as a yellowish solid; eluent for chromatography: hexanes–ethyl acetate mixtures of increasing polarity (9:1 (v/v) to 5:1 (v/v)); yield: 48 mg (0.12 mmol, 49%); mp 128–130 °C; IR (ATR) ν 2934 (w), 1725 (s), 1607 (s), 1323 (s), 1066 (s) cm^–1^; ^1^H NMR (400 MHz, CDCl_3_) δ 7.65 (d, *J* = 9.5 Hz, 1H), 7.49 (d, *J* = 8.2 Hz, 2H), 7.39 (d, *J* = 8.2 Hz, 2H), 7.33 (d, *J* = 8.7 Hz, 1H), 7.09 (d, *J* = 8.7 Hz, 1H), 6.52 (d, *J* = 15.9 Hz, 1 H), 6.44 (dt, *J* = 15.9, 6.0 Hz, 1H), 6.29 (d, *J* = 9.5 Hz, 1H), 5.32 (s, 2H), 3.80 (d, *J* = 6.0 Hz, 2H), 3.48 (s, 3H); ^13^C{^1^H} NMR (100 MHz, CDCl_3_) δ 161.2, 158.0, 153.2, 143.8, 141.2, 129.9, 129.8, 128.9 (q, *J* = 32.0 Hz), 127.0, 126.3, 125.5 (q, *J* = 3.9 Hz), 124.4 (q, *J* = 271.0 Hz), 116.5, 113.8, 113.8, 110.6, 94.5, 56.5, 26.4; HRMS (EI) *m*/*z* [M^+^] calcd for C_21_H_17_O_4_F_3_ 390.1079, found 390.1072.

#### 8-[(2*E*)-3-(3-Fluorophenyl)prop-2-en-1-yl]-7-(methoxymethoxy)-2*H*-chromen-2-one (**12al**)

Obtained from **7l** (63 mg, 0.30 mmol) and **11a** (61 mg, 0.25 mmol) as a yellowish solid; eluent for chromatography: hexanes–ethyl acetate mixtures of increasing polarity (9:1 (v/v) to 5:1 (v/v)); yield: 62 mg (0.18 mmol, 73%); mp 105–107 °C; IR (ATR) ν 2906 (w), 1715 (s), 1602 (s), 1490 (m), 1246 (s), 1019 (s) cm^–1^; ^1^H NMR (400 MHz, CDCl_3_) δ 7.64 (d, *J* = 9.5 Hz, 1H), 7.32 (d, *J* = 8.7 Hz, 1H), 7.20 (td, *J* = 7.9, 6.1 Hz, 1H), 7.08 (d, *J* = 8.6 Hz, 1H), 7.05 (d, *J* = 7.2 Hz, 1H), 6.99 (dm, *J* = 9.5 Hz, 1H), 6.84 (td, *J* = 8.5, 2.5 Hz, 1H), 6.46 (d, *J* = 15.9 Hz, 1H), 6.35 (dt, *J* = 15.9, 6.2 Hz, 1H), 6.28 (d, *J* = 9.5 Hz, 1H), 5.31 (s, 2H), 3.78 (d, *J* = 6.2 Hz, 1H), 3.48 (s, 3H); ^13^C{^1^H} NMR (100 MHz, CDCl_3_) δ 163.2 (d, *J* = 244.9 Hz), 161.2, 158.0, 153.1, 143.8, 140.1 (d, *J* = 7.8 Hz), 130.0, 129.9 (d, *J* = 7.5 Hz), 128.5, 126.9, 122.1 (d, *J* = 2.6 Hz), 116.7, 113.9 (d, *J* = 21.2 Hz), 113.8, 113.7, 112.6 (d, *J* = 21.7 Hz), 110.6, 94.5, 56.5, 26.3; HRMS (EI) *m*/*z* [M^+^] calcd for C_20_H_17_O_4_F 340.1111, found 340.1103.

#### 8-[(2*E*)-3-(3-Chlorophenyl)prop-2-en-1-yl]-7-(methoxymethoxy)-2*H*-chromen-2-one (**12am**)

Obtained from **7m** (226 mg, 1.20 mmol) and **11a** (246 mg, 1.00 mmol) as a yellowish solid; eluent for chromatography: hexanes–ethyl acetate mixtures of increasing polarity (9:1 (v/v) to 5:1 (v/v)); yield: 258 mg (0.72 mmol, 72%); mp 83–85 °C; IR (ATR) ν 1714 (s), 1603 (s), 1247 (s), 1119 (s), 1066 (s), 1022 (s), 966 (s), 836 (s) cm^–1^; ^1^H NMR (400 MHz, CDCl_3_) δ 7.64 (d, *J* = 9.5 Hz, 1H), 7.32 (d, *J* = 8.7 Hz, 1H), 7.29–7.27 (m, 1H), 7.19–7.10 (m, 3H), 7.08 (d, *J* = 8.7 Hz, 1H), 6.44 (d, *J* = 15.9 Hz, 1H), 6.34 (dt, *J* = 15.9, 6.2 Hz, 1H), 6.28 (d, *J* = 9.5 Hz, 1H), 5.31 (s, 2H), 3.78 (d, *J* = 6.2 Hz, 1H), 3.48 (s, 3H); ^13^C{^1^H} NMR (100 MHz, CDCl_3_) δ 161.3, 158.0, 153.1, 143.8, 139.6, 134.5, 129.8, 129.7, 128.7, 127.1, 126.9, 126.1, 124.4, 116.7, 113.8, 113.8, 110.6, 94.5, 56.6, 26.3; HRMS (ESI) *m*/*z* [M + H]^+^ calcd for C_20_H_18_O_4_Cl 357.0894, found 357.0902.

#### 8-[(2*E*)-3-(3-Bromophenyl)prop-2-en-1-yl]-7-(methoxymethoxy)-2*H*-chromen-2-one (**12an**)

Obtained from **7n** (81 mg, 0.30 mmol) and **11a** (61 mg, 0.25 mmol) as a red-brown solid; eluent for chromatography: hexanes–ethyl acetate mixtures of increasing polarity (9:1 (v/v) to 5:1 (v/v)); yield: 50 mg (0.12 mmol, 50%); mp 106–108 °C; IR (ATR) ν 2907 (w), 1710 (s), 1603 (s), 1494 (m), 1245 (s), 1058 (s) cm^–1^; ^1^H NMR (400 MHz, CDCl_3_) δ 7.63 (d, *J* = 9.5 Hz, 1H), 7.43 (s(br), 1H), 7.32 (d, *J* = 8.7 Hz, 1H), 7.27 (d, *J* = 7.2 Hz, 1H), 7.21 (d, *J* = 8.1 Hz, 1H), 7.12 (d, *J* = 7.8 Hz, 1H), 7.08 (d, *J* = 8.7 Hz, 1H), 6.42 (d, *J* = 15.9 Hz, 1H), 6.33 (dt, *J* = 15.8, 5.7 Hz, 1H), 6.28 (d, *J* = 9.5 Hz, 1H), 5.31 (s, 2H), 3.77 (d, *J* = 5.7 Hz, 2H), 3.48 (s, 3H); ^13^C{^1^H} NMR (100 MHz, CDCl_3_) δ 161.2, 158.0, 153.1, 143.8, 139.8, 130.0, 129.9, 129.6, 129.0, 128.7, 126.9, 124.8, 122.7, 116.6, 113.7, 113.7, 110.6, 94.4, 56.5, 26.3; HRMS (EI) *m*/*z* [M^+^] calcd for C_20_H_17_O_4_^[79]^Br 400.0310, found 400.0314.

#### 8-[(2*E*)-3-(3-Iodophenyl)prop-2-en-1-yl]-7-(methoxymethoxy)-2*H*-chromen-2-one (**12ao**)

Obtained from **7o** (318 mg, 1.20 mmol) and **11a** (246 mg, 1.00 mmol) as a red-brown solid; eluent for chromatography: hexanes–ethyl acetate mixtures of increasing polarity (9:1 (v/v) to 5:1 (v/v)); yield: 108 mg (0.24 mmol, 24%); mp 129–132 °C; IR (ATR) ν 2961 (w), 1709 (s), 1603 (s), 1244 (s), 1115 (s), 1056 (s), 1036 (s), 964 (m) cm^–1^; ^1^H NMR (400 MHz, CDCl_3_) δ 7.64 (d, *J* = 9.5 Hz, 1H), 7.64 (t, *J* = 1.5 Hz, 1H), 7.48 (dm, *J* = 7.8 Hz, 1H), 7.31 (d, *J* = 8.7 Hz, 1H), 7.25 (dm, *J* = 7.8 Hz, 1H), 7.08 (d, *J* = 8.7 Hz, 1H), 6.97 (t, *J* = 7.8 Hz, 1H), 6.38 (d, *J* = 15.9 Hz, 1H), 6.32 (dt, *J* = 15.8, 5.7 Hz, 1H), 6.28 (d, *J* = 9.5 Hz, 1H), 5.31 (s, 2H), 3.77 (d, *J* = 5.7 Hz, 1H), 3.48 (s, 3H); ^13^C{^1^H} NMR (100 MHz, CDCl_3_) δ 161.2, 158.0, 153.1, 143.8, 139.9, 135.9, 135.0, 130.2, 129.5, 128.6, 126.9, 125.4, 116.6, 113.8, 113.8, 110.6, 94.7, 94.4, 56.6, 26.3; HRMS (ESI) *m*/*z* [M + H]^+^ calcd for C_20_H_18_O_4_I 449.0244, found 449.0241.

#### 7-(Methoxymethoxy)-8-[(2*E*)-3-(3-methoxyphenyl)prop-2-en-1-yl]-2*H*-chromen-2-one (**12ap**)

Obtained from **7p** (67 mg, 0.30 mmol) and **11a** (61 mg, 0.25 mmol) as a yellowish solid; eluent for chromatography: hexanes–ethyl acetate mixtures of increasing polarity (9:1 (v/v) to 5:1 (v/v)); yield: 82 mg (0.23 mmol, 93%); mp 73–75 °C; IR (ATR) ν 2936 (w), 1721 (s), 1604 (s), 1491 (m), 1245 (s), 1152 (s), 1115 (s), 1040 (s), 832 (s) cm^–1^; ^1^H NMR (400 MHz, CDCl_3_) δ 7.63 (d, *J* = 9.5 Hz, 1H, H4), 7.31 (d, *J* = 8.6 Hz, 1H, H5), 7.16 (dd, *J* = 8.2, 7.7 Hz, 1H, H5″), 7.07 (d, *J* = 8.7 Hz, 1H, H6), 6.90 (d, *J* = 7.7 Hz, 1H, H6″), 6.85–6.83 (m, 1H, H2″), 6.72 (dd, *J* = 8.2, 1.6 Hz, 1H, H4″), 6.47 (d, *J* = 15.8 Hz, 1H, H3′), 6.33 (dt, *J* = 15.8, 6.5 Hz, 1H, H2′), 6.28 (d, *J* = 9.5 Hz, 1H, H3), 5.31 (s, 2H, −OC*H*_2_O−), 3.78 (d, *J* = 6.5 Hz, 2H, H1′), 3.77 (s, 3H, −OC*H*_3_), 3.48 (s, 3H, −OCH_2_OC*H*_3_); ^13^C{^1^H} NMR (100 MHz, CDCl_3_) δ 161.3 (C2), 159.8 (C3″), 158.0 (C7), 153.1 (C8a), 143.8 (C4), 139.1 (C1″), 130.9 (C3′), 129.5 (C5″), 127.4 (C2′), 126.8 (C5), 118.8 (C6″), 117.0 (C8), 113.8 (C4a), 113.7 (C3), 112.9 (C4″), 111.4 (C2″), 110.6 (C6), 94.4 (*−OC*H_2_OCH_3_), 56.5 (*−OC*H_3_), 55.3 (−OCH_2_O*C*H_3_), 26.3 (C1′); HRMS (EI): *m*/*z* [M^+^] calcd for C_21_H_20_O_5_ 352.1311, found 352.1309.

#### 7-(Methoxymethoxy)-8-[(2*E*)-3-(3-methylphenyl)prop-2-en-1-yl]-2*H*-chromen-2-one (**12aq**)

Obtained from **7q** (62 mg, 0.30 mmol) and **11a** (61 mg, 0.25 mmol) as a yellowish solid; eluent for chromatography: hexanes–ethyl acetate mixtures of increasing polarity (9:1 (v/v) to 5:1 (v/v)); yield: 63 mg (0.19 mmol, 75%); mp 95–96 °C; IR (ATR) ν 2907 (w), 1714 (s), 1602 (s), 1413 (m), 1246 (s), 1024 (s) cm^–1^; ^1^H NMR (400 MHz, CDCl_3_) δ 7.64 (d, *J* = 9.5 Hz, 1H), 7.31 (d, *J* = 8.7 Hz, 1H), 7.16–7.08 (3H), 7.07 (d, *J* = 8.7 Hz, 1H), 6.98 (d, *J* = 7.0 Hz, 1H), 6.47 (d, *J* = 15.8 Hz, 1H), 6.32 (dt, *J* = 15.7, 6.5 Hz, 1H), 6.28 (d, *J* = 9.5 Hz, 1H), 5.31 (s, 2H), 3.77 (d, *J* = 6.5 Hz, 2H), 3.49 (s, 3H), 2.29 (s, 3H); ^13^C{^1^H} NMR (100 MHz, CDCl_3_) δ 161.3, 158.0, 153.1, 143.8, 138.1, 137.6, 131.1, 128.5, 127.9, 126.9, 126.8, 126.7, 123.3, 117.2, 113.8, 113.7, 110.6, 94.5, 56.5, 26.4, 21.5; HRMS (EI) *m*/*z* [M^+^] calcd for C_21_H_20_O_4_ 336.1362, found 336.1365.

#### Methyl 3-{(1*E*)-3-[7-(Methoxymethoxy)-2-oxo-2*H*-chromen-8-yl]prop-1-en-1-yl}benzoate (**12ar**)

Obtained from **7r** (79 mg, 0.30 mmol) and **11a** (61 mg, 0.25 mmol) as an off-white solid; eluent for chromatography: hexanes–ethyl acetate mixtures of increasing polarity (9:1 (v/v) to 5:1 (v/v)); yield: 66 mg (0.17 mmol, 69%); mp 110–112 °C; IR (ATR) ν 2955 (w), 1714 (s), 1604 (s), 1432 (m), 1246 (s), 1025 (s) cm^–1^; ^1^H NMR (400 MHz, CDCl_3_) δ 7.95 (t, *J* = 1.7 Hz, 1H), 7.82 (dt, *J* = 7.7, 1.2 Hz, 1H), 7.64 (d, *J* = 9.5 Hz, 1H), 7.49 (dt, *J* = 7.8, 1.4 Hz, 1H), 7.32 (t, *J* = 7.8 Hz, 1H), 7.31 (d, *J* = 8.6 Hz, 1H), 7.09 (d, *J* = 8.7 Hz, 1H), 6.52 (d, *J* = 15.9 Hz, 1H), 6.42 (dt, *J* = 15.8, 6.3 Hz, 1H), 6.29 (d, *J* = 9.5 Hz, 1H), 5.32 (s, 2H), 3.89 (s, 3H), 3.80 (d, *J* = 6.3 Hz, 2H), 3.48 (s, 3H); ^13^C{^1^H} NMR (100 MHz, CDCl_3_) δ 167.2, 161.3, 158.0, 153.2, 143.9, 138.0, 130.6, 130.5, 130.0, 128.6, 128.5, 128.2, 127.3, 126.9, 116.8, 113.8, 113.8, 110.6, 94.5, 56.6, 52.2, 26.4; HRMS (EI) *m*/*z* [M^+^] calcd for C_22_H_20_O_6_ 380.1260, found 380.1264.

#### 7-(Methoxymethoxy)-8-[(2*E*)-3-(2-methoxyphenyl)prop-2-en-1-yl]-2*H*-chromen-2-one (**12at**)

Obtained from **7t** (67 mg, 0.30 mmol) and **11a** (61 mg, 0.25 mmol) as a yellowish oil; eluent for chromatography: hexanes–ethyl acetate mixtures of increasing polarity (9:1 (v/v) to 5:1 (v/v)); yield: 56 mg (0.16 mmol, 64%); IR (ATR) ν 2961 (w), 1721 (s), 1605 (s), 1488 (m), 1243 (s), 1053 (s), 1019 (s) cm^–1^; ^1^H NMR (400 MHz, CDCl_3_) δ 7.61 (d, *J* = 9.6 Hz, 1H, H4), 7.35 (dd, *J* = 7.6, 1.8 Hz, 1H, H6″), 7.28 (d, *J* = 8.6 Hz, 1H, H5), 7.14 (td, *J* = 7.6, 1.8 Hz, 1H, H4″), 7.06 (d, *J* = 8.6 Hz, 1H, H6), 6.86 (d, *J* = 15.9 Hz, 1H, H3′), 6.84 (t, *J* = 7.5 Hz, 1H, H5″), 6.80 (d, *J* = 8.1 Hz, 1H, H3″), 6.34 (dt, *J* = 15.8, 6.9 Hz, 1H, H2′), 6.25 (d, *J* = 9.5 Hz, 1H, H3), 5.30 (s, 2H, −OC*H*_2_O−), 3.80 (d, *J* = 6.9 Hz, 2H, H1′), 3.79 (s, 3H, −OC*H*_3_), 3.49 (s, 3H, −OCH_2_OC*H*_3_); ^13^C{^1^H} NMR (100 MHz, CDCl_3_) δ 161.3 (C2), 158.0 (C7), 156.4 (C2″), 153.0 (C8a), 143.8 (C4), 128.1 (C4″), 127.5 (C2′), 126.6 (C1″), 126.6 (C6″), 126.5 (C5), 125.9 (C3′), 120.6 (C5″), 117.3 (C8), 113.7 (C4a), 113.5 (C3), 110.8 (C3″), 110.6 (C6), 94.3 (*−OC*H_2_OCH_3_), 56.4 (−OCH_2_O*C*H_3_), 55.4 (*−OC*H_3_), 26.8 (C1′); HRMS (EI) *m*/*z* [M^+^] calcd for C_21_H_20_O_5_ 352.1311, found 352.1302.

#### Methyl 2-{(1*E*)-3-[7-(Methoxymethoxy)-2-oxo-2*H*-chromen-8-yl]prop-1-en-1-yl}benzoate (**12au**)

Obtained from **7u** (75 mg, 0.30 mmol) and **11a** (61 mg, 0.25 mmol) as a colorless oil; eluent for chromatography: hexanes–ethyl acetate mixtures of increasing polarity (9:1 (v/v) to 5:1 (v/v)); yield: 71 mg (0.19 mmol, 75%); IR (ATR) ν 2951 (w), 1716 (s), 1605 (s), 1433 (w), 1245 (s), 1115 (s), 1058 (s), 832 (s) cm^–1^; ^1^H NMR (400 MHz, CDCl_3_) δ 7.79 (dd, *J* = 7.9, 1.1 Hz, 1H), 7.64 (d, *J* = 9.5 Hz, 1H), 7.49 (d, *J* = 7.8 Hz, 1H), 7.38 (td, *J* = 7.6, 1.1 Hz, 1H), 7.31 (d, *J* = 8.6 Hz, 1H), 7.24–7.17 (m, 2H), 7.09 (d, *J* = 8.6 Hz, 1H), 6.30–6.21 (m, 3H), 5.31 (s, 2H), 3.83 (s, 3H), 3.82 (dd, *J* = 6.3, 1.3 Hz, 2H), 3.48 (s, 3H); ^13^C{^1^H} NMR (100 MHz, CDCl_3_) δ 168.1, 161.3, 158.3, 153.1, 143.8, 139.2, 132.0, 130.4, 129.9, 129.7, 128.5, 127.3, 126.8, 126.8, 117.0, 113.7, 113.7, 110.8, 94.6, 56.5, 52.0, 26.6; HRMS (EI) *m*/*z* [M^+^] calcd for C_22_H_20_O_6_ 380.1260, found 380.1271.

#### (*E*)-7-(Methoxymethoxy)-2-(4-(methoxymethoxy)phenyl)-8-(3-(4-methoxyphenyl)allyl) chroman-4-one (**16af**)

Obtained from **7f** (270 mg, 1.20 mmol) and **15a** (390 mg, 1.00 mmol) as a yellow solid; eluent for chromatography: hexanes–ethyl acetate mixtures of increasing polarity (9:1 (v/v) to 5:1 (v/v)); yield: 372 mg (0.76 mmol, 76%); mp 105–107 °C; IR (ATR) ν 2886 (w), 1689 (s), 1595 (m), 1510 (s), 1244 (s), 1150 (s) cm^–1^; ^1^H NMR (400 MHz, CDCl_3_) δ 7.83 (d, *J* = 8.9 Hz, 1H, H5), 7.40 (d, *J* = 8.6 Hz, 2H, H2‴), 7.21 (d, *J* = 8.7 Hz, 2H, H2″), 7.08 (d, *J* = 8.6 Hz, 2H, H3‴), 6.84 (d, *J* = 8.9 Hz, 1H, H6), 6.81 (d, *J* = 8.7 Hz, 2H, H3″), 6.34 (d, *J* = 15.8 Hz, 1H, H3′), 6.13 (dt, *J* = 15.8, 6.7 Hz, 1H, H2′), 5.44 (dd, *J* = 12.8, 3.0 Hz, 1H, H2), 5.29 (s, 2H, C7–OC*H*_2_–OCH_3_), 5.21 (s, 2H, C4‴–OC*H*_2_–OCH_3_), 3.79 (s, 3H, −OC*H*_3_), 3.59–3.53 (m, 2H, H1′), 3.50 (s, 3H, C4‴–OCH_2_–OC*H*_3_), 3.48 (s, 3H, C7–OCH_2_–OC*H*_3_), 3.02 (dd, *J* = 16.8, 12.8 Hz, 1H, H3^ax^), 2.85 (dd, *J* = 16.8, 3.0 Hz, 1H, H3^eq^); ^13^C{^1^H} NMR (100 MHz, CDCl_3_) δ 191.6 (C4), 161.0 (C7), 160.7 (C8a), 158.8 (C4″), 157.5 (C4‴), 132.6 (C1‴), 130.7 (C1″), 129.9 (C3′), 127.6 (C2‴), 127.2 (C2″), 126.6 (C5), 125.5 (C2′), 117.2 (C8), 116.5 (C3‴), 116.0 (C4a), 114.0 (C3″), 107.9 (C6), 94.5 (C4‴*–OC*H_2_–OCH_3_), 94.2 (C7–O*C*H_2_–OCH_3_), 79.3 (C1), 56.5 (C7–OCH_2_–O*C*H_3_), 56.2 (C7–OCH_2_–O*C*H_3_), 55.4 (C4″*–OC*H_3_), 44.3 (C3), 26.7 (C2′); HRMS (EI) *m*/*z* [M^+^] calcd for C_29_H_30_O_7_ 490.1992, found 490.1984.

#### (*E*)-7-Methoxy-2-(4-(methoxymethoxy)phenyl)-8-(3-(4-methoxyphenyl)allyl)chroman-4-one (**16bf**)

Obtained from **7f** (270 mg, 1.20 mmol) and **15b** (355 mg, 1.00 mmol) as a yellow paste; eluent for chromatography: hexanes–ethyl acetate mixtures of increasing polarity (9:1 (v/v) to 5:1 (v/v)); yield: 295 mg (0.64 mmol, 64%); IR (ATR) ν 2902 (w), 2837 (w), 1681 (s), 1595 (s), 1510 (s), 1243 (s), 1108 (s) cm^–1^; ^1^H NMR (400 MHz, CDCl_3_) δ 7.86 (d, *J* = 8.8 Hz, 1H), 7.40 (d, *J* = 8.7 Hz, 2H), 7.22 (d, *J* = 8.7 Hz, 2H), 7.08 (d, *J* = 8.7 Hz, 2H), 6.81 (d, *J* = 8.7 Hz, 2H), 6.65 (d, *J* = 8.8 Hz, 1H), 6.33 (d, *J* = 15.9 Hz, 1H), 6.12 (dt, *J* = 15.9, 6.7 Hz, 1H), 5.43 (dd, *J* = 12.8, 3.1 Hz, 1H), 5.20 (s, 2H), 3.92 (s, 3H), 3.79 (s, 3H), 3.55–3.51 (m, 2H), 3.50 (s, 3H), 3.01 (dd, *J* = 16.9, 12.8 Hz, 1H), 2.84 (dd, *J* = 16.9, 3.1 Hz, 1H); ^13^C{^1^H} NMR (100 MHz, CDCl_3_) δ 191.6, 163.4, 160.5, 158.8, 157.4, 132.7, 130.7, 129.9, 127.6, 127.2, 126.8, 125.6, 116.5, 116.4, 115.5, 114.0, 105.0, 94.5, 79.2, 56.2, 56.1, 55.4, 44.3, 26.5; HRMS (EI) *m*/*z* [M^+^] calcd for C_28_H_28_O_6_ 460.1886, found 460.1870.

#### (*E*)-7-(Methoxymethoxy)-2-(4-methoxyphenyl)-8-(3-(4-methoxyphenyl)allyl)chroman-4-one (**16cf**)

Obtained from **7f** (270 mg, 1.20 mmol) and **15c** (355 mg, 1.00 mmol) as a yellow solid; eluent for chromatography: hexanes–ethyl acetate mixtures of increasing polarity (9:1 (v/v) to 5:1 (v/v)); yield: 275 mg (0.60 mmol, 60%). Suitable crystals for single crystal X-ray diffraction analysis were obtained by slowly evaporating the solvent from a solution of **16cf** in a hexanes–ethyl acetate mixture: mp 101–102 °C; IR (ATR) ν 2892 (w), 1688 (m), 1588 (s), 1510 (s), 1250 (s), 1032 (s) cm^–1^; ^1^H NMR (400 MHz, CDCl_3_) δ 7.83 (d, *J* = 8.9 Hz, 1H), 7.40 (d, *J* = 8.5 Hz, 2H), 7.21 (d, *J* = 8.7 Hz, 2H), 6.94 (d, *J* = 8.7 Hz, 2H), 6.83 (d, *J* = 8.9 Hz, 1H), 6.81 (d, *J* = 8.5 Hz, 2H), 6.33 (d, *J* = 15.7 Hz, 1H), 6.13 (dt, *J* = 15.7, 6.7 Hz, 1H), 5.44 (dd, *J* = 12.7, 3.1 Hz, 1H), 5.28 (s, 2H), 3.83 (s, 3H), 3.79 (s, 3H), 3.58–3.54 (m, 2H), 3.47 (s, 3H), 3.03 (dd, *J* = 16.9, 12.7 Hz, 1H), 2.86 (dd, *J* = 16.9, 3.1 Hz, 1H); ^13^C{^1^H} NMR (100 MHz, CDCl_3_) δ 191.7, 161.0, 160.7, 159.9, 158.8, 131.4, 130.7, 129.9, 127.7, 127.2, 126.6, 125.5, 117.2, 116.0, 114.2, 114.0, 107.8, 94.2, 79.3, 56.5, 55.5, 55.4, 44.2, 26.7; HRMS (EI): *m*/*z* [M^+^] calcd for C_28_H_28_O_6_ 460.1886, found 460.1893.

#### 7-Methoxy-3-(4-methoxyphenyl)-8-[(2*E*)-3-(4-methoxyphenyl)prop-2-en-1-yl]-4*H*-chromen-4-one (**20**)

Obtained from **7f** (270 mg, 1.20 mmol) and **19** (324 mg, 1.00 mmol) as a colorless solid; eluent for chromatography: hexanes–ethyl acetate mixtures of increasing polarity (9:1 (v/v) to 5:1 (v/v)); yield: 305 mg (0.71 mmol, 71%); mp 123–124 °C; IR (ATR) ν 1651 (s), 1605 (s), 1588 (s), 1511 (s), 1250 (s), 1176 (s) cm^–1^; ^1^H NMR (400 MHz, CDCl_3_) δ 8.22 (d, *J* = 8.9 Hz, 1H, H5), 8.00 (s, 1H, H2), 7.51 (d, *J* = 8.7 Hz, 2H, H2‴), 7.26 (d, *J* = 8.7 Hz, 2H, H2″), 7.05 (d, *J* = 8.9 Hz, 1H, H6), 6.97 (d, *J* = 8.7 Hz, 2H, H3‴), 6.81 (d, *J* = 8.7 Hz, 2H, H3″), 6.38 (d, *J* = 15.8 Hz, 1H, H3′), 6.20 (dt, *J* = 15.8, 6.5 Hz, 1H, H2′), 3.98 (s, 3H, C7–OC*H*_3_), 3.84 (s, 3H, C4‴–OC*H*_3_), 3.78 (s, 3H, C4″–OCH_3_), 3.75 (dd, *J* = 6.5, 1.0 Hz, 2H, H1′); ^13^C{^1^H} NMR (100 MHz, CDCl_3_) δ 176.5 (C4), 161.2 (C7), 159.6 (C4‴), 158.9 (C4″), 155.3 (C8a), 152.5 (C2), 130.4 (C1″), 130.2 (C2‴), 130.1 (C3′), 127.3 (C2″), 125.9 (C5), 125.0 (C2′), 124.5 (C1‴), 124.3 (C3), 118.7 (C4a), 116.1 (C8), 114.1 (C3‴), 114.0 (C3″), 109.2 (C6), 56.4 (C7–OCH_3_), 55.4 (C4‴–OCH_3_), 55.4 (C4″–OCH_3_), 26.3 (C1′); HRMS (EI) *m*/*z* [M^+^] calcd for C_27_H_24_O_5_ 428.1624, found 428.1628.

#### 7-(Methoxymethoxy)-3-{4-methoxy-3-[(2*E*)-3-(4-methoxyphenyl)-prop-2-en-1-yl]phenyl}-4*H*-chromen-4-one (**23**)

Obtained from **7f** (270 mg, 1.20 mmol) and **22** (353 mg, 1.00 mmol) as an amorphous colorless solid; eluent for chromatography: hexanes–ethyl acetate mixtures of increasing polarity (9:1 (v/v) to 5:1 (v/v)); yield: 224 mg (0.52 mmol, 52%); IR (ATR) ν 2922 (w), 1641 (s), 1606 (s), 1241 (s) cm^–1^; ^1^H NMR (400 MHz, CDCl_3_) δ 8.21 (d, *J* = 9.5 Hz, 1H, H5), 7.91 (s, 1H, H2), 7.44 (dd, *J* = 8.6, 2.0 Hz, 1H, H6′), 7.35 (d, *J* = 2.0 Hz, 1H, H2′), 7.28 (d, *J* = 8.6 Hz, 2H, H2‴), 7.09–7.04 (m, 2H, H6, H8), 6.94 (d, *J* = 8.6 Hz, 1H, H5′), 6.82 (d, *J* = 8.6 Hz, 2H, H3‴), 6.40 (d, *J* = 15.8 Hz, 1H, H3″), 6.25 (dt, *J* = 15.8, 6.8 Hz, 1H, H2″), 5.27 (s, 2H, −OC*H*_2_O−), 3.88 (s, 3H, C4′–OC*H*_3_), 3.78 (s, 3H, C4‴–OC*H*_3_), 3.55 (d, *J* = 6.8 Hz, 2H, H1″), 3.51 (s, 3H, −OCH_2_OC*H*_3_); ^13^C{^1^H} NMR (100 MHz, CDCl_3_) δ 176.1 (C4), 161.5 (C7), 158.8 (C4‴), 157.8 (C8a), 157.5 (C4′), 152.4 (C2), 130.7 (C1‴), 130.5 (C2′), 130.3 (C3″), 129.2 (C3′), 128.3 (C6′), 128.0 (C5), 127.3 (C2‴), 126.7 (C2″), 125.2 (C3), 124.1 (C1′), 119.4 (C4a), 115.5 (C6 or C8), 114.0 (C3‴), 110.6 (C5′), 103.2 (C6 or C8), 94.5 (*−OC*H_2_OCH_3_), 56.5 (−OCH_2_O*C*H_3_), 55.7 (C4′*–OC*H_3_) 55.4 (C4‴*–OC*H_3_), 33.6 (C1″); HRMS (ESI): *m*/*z* [M + H]^+^ calcd for C_28_H_27_O_6_ 459.1808, found 459.1814.

### General Procedure for the Allylic Oxidation of Matsuda–Heck Coupling Products

Arylallyl chromanones **12** (0.25 mmol, 1.00 equiv), DDQ (136 mg, 0.60 mmol, 2.40 equiv) and silica (100 mg) were suspended in 1,4-dioxane (5 mL, 0.05 M in **12**) in a vessel suitable for microwave irradiation. The vessel was sealed, and the mixture was heated to 90 °C under microwave irradiation for 25 min. After cooling to ambient temperature, it was filtered through a silica-Celite pad and washed with MTBE (150 mL). The solvent was removed under reduced pressure. The residue was purified by flash chromatography on silica, using hexanes–MTBE mixtures of increasing polarity as eluent. Attempts for upscaling to a 1 mmol scale were unsuccessful due to limitations in vial size, and because the initial substrate concentration of 0.05 M could not be increased.

#### 7-(Methoxymethoxy)-8-[(1*E*)-3-oxo-3-phenylprop-1-en-1-yl]-2*H*-chromen-2-one (**13aa**)

Obtained from **12aa** (81 mg, 0.25 mmol) as an off-white solid; eluent for chromatography: hexanes–ethyl acetate mixtures of increasing polarity (9:1 (v/v) to 5:1 (v/v)); yield: 47 mg (0.14 mmol, 56%); mp 141–144 °C; IR (ATR) ν 2909 (w), 1726 (s), 1661 (m), 1588 (s), 1115 (m), 1044 (s) cm^–1^; ^1^H NMR (400 MHz, CDCl_3_) δ 8.31 (d, *J* = 16.2 Hz, 1H), 8.22 (d, *J* = 16.1 Hz, 1H), 8.10 (dm, *J* = 8.0 Hz, 2H, H2″), 7.67 (d, *J* = 9.5 Hz, 1H, H4), 7.59 (tm, *J* = 7.3 Hz, 1H, H4″), 7.51 (tm, *J* = 7.5 Hz, 2H, H3″), 7.43 (d, *J* = 8.7 Hz, 1H, H5), 7.15 (d, *J* = 8.7 Hz, 1H, H6), 6.34 (d, *J* = 9.5 Hz, 1H, H3), 5.37 (s, 2H, −OC*H*_2_O−), 3.53 (s, 3H, −OC*H*_3_); ^13^C{^1^H} NMR (100 MHz, CDCl_3_) δ 191.2 (C3′), 160.1 (C2), 159.6 (C7), 154.2 (C8a), 143.8 (C4), 138.3 (C1″), 133.0 (C4″), 132.5 (C1′), 130.1 (C5), 128.8 (C2″), 128.8 (C3″), 127.8 (C2′), 114.0 (C3), 113.8 (C4a), 113.1 (C8), 111.0 (C6), 95.0 (*−OC*H_2_OCH_3_), 56.9 (−OCH_2_O*C*H_3_); HRMS (ESI) *m*/*z* [M + H]^+^ calcd for C_20_H_17_O_5_ 337.1071, found 337.1068.

#### 8-[(1*E*)-3-(4-Fluorophenyl)-3-oxoprop-1-en-1-yl]-7-(methoxymethoxy)-2*H*-chromen-2-one (**13ab**)

Obtained from **12ab** (85 mg, 0.25 mmol) as a colorless solid; eluent for chromatography: hexanes–ethyl acetate mixtures of increasing polarity (9:1 (v/v) to 5:1 (v/v)); yield: 75 mg (0.21 mmol, 85%); mp 158–160 °C; IR (ATR) ν 1729 (s), 1663 (m), 1596 (s), 1276 (m), 1240 (m), 1154 (s), 1116 (s), 1045 (s), 835 (s) cm^–1^; ^1^H NMR (400 MHz, CDCl_3_) δ 8.30 (d, *J* = 16.1 Hz, 1H), 8.22 (d, *J* = 16.1 Hz, 1H), 8.17–8.10 (m, 2H), 7.68 (d, *J* = 9.5 Hz, 1H), 7.44 (d, *J* = 8.8 Hz, 1H), 7.23–7.14 (m, 2H), 6.35 (d, *J* = 9.5 Hz, 1H), 5.37 (s, 2H), 3.53 (s, 3H); ^13^C{^1^H} NMR (100 MHz, CDCl_3_) δ 189.5, 165.8 (d, *J* = 254.3 Hz), 160.1, 159.6, 154.1, 143.8, 134.6 (d, *J* = 3.0 Hz), 132.6, 131.4 (d, *J* = 9.7 Hz), 130.2, 127.2, 115.9 (d, *J* = 21.8 Hz), 113.9, 113.7, 112.8, 111.0, 95.0, 56.9; HRMS (ESI) *m*/*z* [M + H]^+^ calcd for C_20_H_16_O_5_F 355.0976, found 355.0970.

#### 8-[(1*E*)-3-(4-Chlorophenyl)-3-oxoprop-1-en-1-yl]-7-(methoxymethoxy)-2*H*-chromen-2-one (**13ac**)

Obtained from **12ac** (91 mg, 0.25 mmol) as an off-white solid; eluent for chromatography: hexanes–ethyl acetate mixtures of increasing polarity (9:1 (v/v) to 5:1 (v/v)); yield: 44 mg (0.12 mmol, 47%); mp 156–160 °C; IR (ATR) ν 1720 (s), 1662 (s), 1593 (s), 1554 (s), 1082 (s) cm^–1^; ^1^H NMR (400 MHz, CDCl_3_) δ 8.27 (d, *J* = 16.1 Hz, 1H), 8.21 (d, *J* = 16.1 Hz, 1H), 8.03 (d, *J* = 8.6 Hz, 2H), 7.67 (d, *J* = 9.5 Hz, 1H), 7.49 (d, *J* = 8.6 Hz, 2H), 7.44 (d, *J* = 8.7 Hz, 1H), 7.16 (d, *J* = 8.7 Hz, 1H), 6.34 (d, *J* = 9.5 Hz, 1H), 5.37 (s, 2H), 3.53 (s, 3H); ^13^C{^1^H} NMR (100 MHz, CDCl_3_) δ 190.0, 160.1, 159.6, 154.2, 143.8, 139.4, 136.6, 133.0, 130.3, 130.2, 129.1, 127.2, 114.0, 113.8, 112.9, 111.1, 95.0, 56.9; HRMS (ESI) *m*/*z* [M + H]^+^ calcd for C_20_H_16_O_5_Cl 371.0681, found 371.0674.

#### 8-[(1*E*)-3-(4-Bromophenyl)-3-oxoprop-1-en-1-yl]-7-(methoxymethoxy)-2*H*-chromen-2-one (**13ad**)

Obtained from **12ad** (100 mg, 0.25 mmol) as an off-white solid; eluent for chromatography: hexanes–ethyl acetate mixtures of increasing polarity (9:1 (v/v) to 5:1 (v/v)); yield: 45 mg (0.11 mmol, 43%); mp 166–169 °C; IR (ATR) ν 2973 (w), 1713 (s), 1592 (m), 1361 (m), 1221 (s), 1166 (w) cm^–1^; ^1^H NMR (400 MHz, CDCl_3_) δ 8.27 (d, *J* = 16.3 Hz, 1H), 8.22 (d, *J* = 16.3 Hz, 1H), 7.96 (dm, *J* = 8.5 Hz, 2H), 7.67 (d, *J* = 9.5 Hz, 1H), 7.66 (dm, *J* = 8.6 Hz, 2H), 7.44 (d, *J* = 8.8 Hz, 1H), 7.16 (d, *J* = 8.8 Hz, 1H), 6.34 (d, *J* = 9.5 Hz, 1H), 5.37 (s, 2H), 3.53 (s, 3H); ^13^C{^1^H} NMR (100 MHz, CDCl_3_) δ 190.1, 160.1, 159.6, 154.2, 143.8, 137.1, 133.0, 132.1, 130.4, 130.3, 128.2, 127.0, 114.0, 113.8, 112.9, 111.1, 95.0, 56.9; HRMS (ESI) *m*/*z* [M + H]^+^ calcd for C_20_H_16_O_5_Br 415.0181, found 415.0158.

#### 8-[(1*E*)-3-(4-Iodophenyl)-3-oxoprop-1-en-1-yl]-7-(methoxymethoxy)-2*H*-chromen-2-one (**13ae**)

Obtained from **12ae** (112 mg, 0.25 mmol) as an off white solid; eluent for chromatography: hexanes–ethyl acetate mixtures of increasing polarity (9:1 (v/v) to 5:1 (v/v)); yield: 34 mg (0.07 mmol, 29%); mp 150–154 °C. A second fraction contained 7-hydroxy-8-[(2*E*)-3-(4-iodophenyl)prop-2-enoyl]-2*H*-chromen-2-one (**14ae**): off-white solid; yield: (17 mg, 0.04 mmol, 16%); mp 220–222 °C. *Analytical data for**13ae***: IR (ATR) ν 2926 (w), 1730 (s), 1661 (m), 1591 (s), 1557 (m), 1277 (m), 1245 (m), 1116 (m), 1047 (s), 1005 (m), 830 (m) cm^–1^; ^1^H NMR (400 MHz, CDCl_3_) δ 8.26 (d, *J* = 16.2 Hz, 1H, H1′), 8.21 (d, *J* = 16.2 Hz, 1H, H2′), 7.88 (dm, *J* = 8.6 Hz, 2H, H3″), 7.80 (dm, *J* = 8.6 Hz, 2H, H2″), 7.67 (d, *J* = 9.5 Hz, 1H, H4), 7.44 (d, *J* = 8.7 Hz, 1H, H5), 7.15 (d, *J* = 8.7 Hz, 1H, H6), 6.34 (d, *J* = 9.5 Hz, 1H, H3), 5.37 (s, 2H, −OC*H*_2_O−), 3.53 (s, 3H, −OC*H*_3_); ^13^C{^1^H} NMR (100 MHz, CDCl_3_) δ 190.5 (C3′), 160.1 (C2), 159.6 (C7), 154.2 (C8a), 143.8 (C4), 138.1 (C3″), 137.6 (C1″), 133.0 (C2′), 130.3 (C5), 130.2 (C2″), 127.1 (C1′), 114.0 (C3), 113.8 (C4a), 112.9 (C8), 111.1 (C6), 101.0 (C4″), 95.0 (*−OC*H_2_OCH_3_), 56.9 (−OCH_2_O*C*H_3_); HRMS (ESI) *m*/*z* [M + H]^+^ calcd for C_20_H_16_O_5_I 463.0037, found 463.0043. *Analytical data for**14ae***: IR (ATR) ν 1730 (s), 1631 (s), 1599 (s), 1544 (s), 1584 (s), 1351 (s), 1193 (s), 1117 (m), 833 (s) cm^–1^; ^1^H NMR (400 MHz, CDCl_3_) δ 13.88 (s, 1H, -O*H*), 8.28 (d, *J* = 15.5 Hz, 1H, H2′), 7.87 (d, *J* = 15.5 Hz, 1H, H3′), 7.79 (dm, *J* = 8.4 Hz, 2H, H3″), 7.66 (d, *J* = 9.5 Hz, 1H, H4), 7.54 (d, *J* = 8.8 Hz, 1H, H5), 7.46 (dm, *J* = 8.4 Hz, 2H, H2″), 6.94 (d, *J* = 8.8 Hz, 1H, H6), 6.31 (d, *J* = 9.5 Hz, 1H, H3); ^13^C{^1^H} NMR (100 MHz, CDCl_3_): δ = 193.2 (C1′), 167.9 (C7), 159.5 (C2), 155.7 (C8a), 144.9 (C3′), 144.4 (C4), 138.5 (C3″), 134.8 (C5), 134.4 (C1″), 130.5 (C2″), 126.8 (C2′), 115.9 (C6), 112.3 (C3), 111.3 (C4a), 109.8 (C8), 97.8 (C4″); HRMS (ESI) *m*/*z* [M + H]^+^ calcd for C_18_H_12_O_4_^127^I 418.9775, found 418.9781.

#### 7-(Methoxymethoxy)-8-[(1*E*)-3-(4-methoxyphenyl)-3-oxoprop-1-en-1-yl]-2*H*-chromen-2-one (**13af**)

Obtained from **12af** (88 mg, 0.25 mmol) as an off-white solid; eluent for chromatography: hexanes–ethyl acetate mixtures of increasing polarity (9:1 (v/v) to 5:1 (v/v)); yield: 71 mg (0.19 mmol, 78%); mp 140–143 °C. A second, slightly more polar fraction consisted of 7-hydroxy-8-[(1*E*)-3-(4-methoxyphenyl)-3-oxoprop-1-en-1-yl]-2*H*-chromen-2-one (**14af**), contaminated with ca. 10% of **13af**. *Analytical data for****13af***: IR (ATR) ν 1726 (s), 1655 (m), 1588 (s), 1453 (m), 1243 (m), 1167 (s), 1045 (s) cm^–1^; ^1^H NMR (400 MHz, CDCl_3_) δ 8.32 (d, *J* = 16.0 Hz, 1H, H2′), 8.19 (d, *J* = 16.1 Hz, 1H, H1′), 8.11 (dm, *J* = 8.5 Hz, 2H, H2″), 7.67 (d, *J* = 9.5 Hz, 1H, H4), 7.42 (d, *J* = 8.7 Hz, 1H, H5), 7.14 (d, *J* = 8.8 Hz, 1H, H6), 6.99 (dm, *J* = 8.5 Hz, 2H, H3″), 6.33 (d, *J* = 9.5 Hz, 1H, H3), 5.36 (s, 2H, −OC*H*_2_O−), 3.89 (s, 3H, −OC*H*_3_), 3.52 (s, 3H, −OCH_2_OC*H*_3_); ^13^C{^1^H} NMR (100 MHz, CDCl_3_) δ 189.6 (C3′), 163.6 (C4″), 160.2 (C2), 159.5 (C7), 154.1 (C8a), 143.8 (C4), 131.6 (C1′), 131.3 (C1″), 131.2 (C2″), 129.9 (C5), 127.8 (C2′), 114.1 (C3″), 114.0 (C3), 113.8 (C4a), 113.3 (C8), 111.1 (C6), 95.0 (*−OC*H_2_OCH_3_), 56.9 (−OCH_2_O*C*H_3_), 55.6 (*−OC*H_3_); HRMS (ESI) *m*/*z* [M + H]^+^ calcd for C_21_H_19_O_6_ 367.1176, found 367.1171. *Selected analytical data of the minor byproduct**14af**(obtained from a sample contaminated with**13af**)*: ^1^H NMR (400 MHz, acetone-*d*_6_) δ 8.41 (d, *J* = 16.0 Hz, 1H), 8.28 (d, *J* = 16.1 Hz, 1H), 8.09 (dm, *J* = 8.5 Hz, 2H), 7.93 (d, *J* = 9.5 Hz, 1H), 7.58 (d, *J* = 9.5 Hz, 1H), 7.09 (dm, *J* = 8.6 Hz, 2H), 7.06 (d, *J* = 8.6 Hz, 1H), 6.27 (d, *J* = 9.5 Hz, 1H), 3.92 (s, 3H).

#### Ethyl 4-{(2*E*)-3-[7-(Methoxymethoxy)-2-oxo-2*H*-chromen-8-yl]prop-2-enoyl}benzoate (**13ai**)

Obtained from **12ai** (99 mg, 0.25 mmol) as an off-white solid; eluent for chromatography: hexanes–ethyl acetate mixtures of increasing polarity (9:1 (v/v) to 5:1 (v/v)); yield: 36 mg (0.09 mmol, 36%); mp 115–118 °C; IR (ATR) ν 2925 (w), 1713 (m), 1663 (m), 1608 (m), 1583 (s), 1275 (s), 1045 (s) cm^–1^; ^1^H NMR (400 MHz, CDCl_3_) δ 8.29 (d, *J* = 16.4 Hz, 1H), 8.23 (d, *J* = 16.4 Hz, 1H), 8.19 (dm, *J* = 8.4 Hz, 2H), 8.12 (dm, *J* = 8.4 Hz, 2H), 7.67 (d, *J* = 9.5 Hz, 1H), 7.44 (d, *J* = 8.7 Hz, 1H), 7.15 (d, *J* = 8.7 Hz, 1H), 6.34 (d, *J* = 9.5 Hz, 1H), 5.37 (s, 2H), 4.41 (q, *J* = 7.1 Hz, 2H), 3.53 (s, 3H), 1.42 (t, *J* = 7.1 Hz, 3H); ^13^C{^1^H} NMR (100 MHz, CDCl_3_) δ 190.8, 166.0, 160.0, 159.7, 154.2, 143.7, 141.6, 134.1, 133.3, 130.4, 130.0, 128.6, 127.5, 114.1, 113.8, 112.9, 111.0, 95.0, 61.5, 56.9, 14.4; HRMS (ESI) *m*/*z* [M + H]^+^ calcd for C_23_H_21_O_7_ 409.1287, found 409.1263.

#### 7-(Methoxymethoxy)-8-{(1*E*)-3-oxo-3-[4-(trifluoromethyl)phenyl]prop-1-en-1-yl}-2*H*-chromen-2-one (**13ak**)

Obtained from **12ak** (98 mg, 0.25 mmol) as an off-white solid; eluent for chromatography: hexanes–ethyl acetate mixtures of increasing polarity (9:1 (v/v) to 5:1 (v/v)); yield: 22 mg (0.05 mmol, 22%); mp 100–104 °C; IR (ATR) ν 1725 (s), 1666 (m), 1591 (s), 1554 (m), 1325 (m), 1287 (m), 1165 (m), 1106 (s), 1066 (s) cm^–1^; ^1^H NMR (400 MHz, CDCl_3_) δ 8.31 (d, *J* = 16.1 Hz, 1H), 8.26 (d, *J* = 16.1 Hz, 1H), 8.19 (d, *J* = 8.5 Hz, 2H), 7.79 (d, *J* = 8.4 Hz, 2H), 7.68 (d, *J* = 9.5 Hz, 1H), 7.46 (d, *J* = 8.8 Hz, 1H), 7.17 (d, *J* = 8.8 Hz, 1H), 6.36 (d, *J* = 9.5 Hz, 1H), 5.38 (s, 2H), 3.54 (s, 3H); ^13^C{^1^H} NMR (100 MHz, CDCl_3_) δ 190.3, 160.0, 159.7, 154.3, 143.8, 141.2, 134.4, 133.7, 130.5, 129.1, 127.1, 125.9 (q, *J* = 3.9 Hz), 124.0 (d, *J* = 272.3 Hz), 114.1, 113.8, 112.8, 111.1, 95.1, 56.9; HRMS (ESI) *m*/*z* [M + H]^+^ calcd for C_21_H_16_O_5_F_3_ 405.0944, found 405.0947.

#### 8-[(1*E*)-3-(3-Fluorophenyl)-3-oxoprop-1-en-1-yl]-7-(methoxymethoxy)-2*H*-chromen-2-one (**13al**)

Obtained from **12al** (85 mg, 0.25 mmol) as an off-white solid; eluent for chromatography: hexanes–ethyl acetate mixtures of increasing polarity (9:1 (v/v) to 5:1 (v/v)); yield: 29 mg (0.08 mmol, 33%); mp 92–95 °C; IR (ATR) ν 1732 (s), 1664 (m), 1583 (s), 1246 (s), 1115 (m), 1047 (s), 728 (m) cm^–1^; ^1^H NMR (400 MHz, CDCl_3_) δ 8.27–8.22 (m, 2H), 7.88 (dm, *J* = 7.6 Hz, 1H), 7.76 (dm, *J* = 9.5 Hz, 1H), 7.67 (d, *J* = 9.5 Hz, 1H), 7.50 (td, *J* = 8.0, 5.6 Hz, 1H), 7.45 (d, *J* = 8.7 Hz, 1H), 7.28 (td, *J* = 8.3, 2.7 Hz, 1H), 7.16 (d, *J* = 8.6 Hz, 1H), 6.35 (d, *J* = 9.6 Hz, 1H), 5.38 (s, 2H), 3.54 (s, 3H); ^13^C{^1^H} NMR (100 MHz, CDCl_3_) δ 190.0, 163.1 (d, *J* = 248.0 Hz), 160.1, 159.6, 154.2, 143.8, 140.5 (d, *J* = 6.4 Hz), 133.2, 130.5 (d, *J* = 8.1 Hz), 130.4, 127.2, 124.6 (d, *J* = 3.1 Hz), 120.0 (d, *J* = 21.7 Hz), 115.5 (d, *J* = 23.0 Hz), 114.0, 113.8, 112.9, 111.0, 95.0, 56.9; HRMS (ESI) *m*/*z* [M + H]^+^ calcd for C_20_H_16_O_5_F 355.0976, found 355.0969.

#### 8-[(1*E*)-3-(3-Chlorophenyl)-3-oxoprop-1-en-1-yl]-7-(methoxymethoxy)-2*H*-chromen-2-one (**13am**)

Obtained from **12am** (89 mg, 0.25 mmol) as an off-white solid; eluent for chromatography: hexanes–ethyl acetate mixtures of increasing polarity (9:1 (v/v) to 5:1 (v/v)); yield: 56 mg (0.15 mmol, 60%); mp 127–130 °C; IR (ATR) ν 1726 (s), 1663 (m), 1596 (s), 1585 (s), 1291 (s), 1204 (s), 1048 (s) cm^–1^; ^1^H NMR (400 MHz, CDCl_3_) δ 8.24–8.21 (m, 2H, H1′, H2′), 8.04 (t, *J* = 1.8 Hz, 1H, H2″), 7.96 (dt, *J* = 7.7, 1.2 Hz, 1H, H6″), 7.66 (d, *J* = 9.5 Hz, 1H, H4), 7.55 (dm, *J* = 7.9 Hz, 1H, H4″), 7.46 (t, *J* = 7.8 Hz, 1H, H5″), 7.44 (d, *J* = 8.8 Hz, 1H, H5), 7.16 (d, *J* = 8.8 Hz, 1H, H6), 6.34 (d, *J* = 9.5 Hz, 1H, H3), 5.37 (s, 2H, −OC*H*_2_O−), 3.53 (s, 3H, −OCH_2_OC*H*_3_); ^13^C{^1^H} NMR (101 MHz, CDCl_3_) δ 190.0 (C3′), 160.0 (C2), 159.6 (C7), 154.2 (C8a), 143.7 (C4), 139.9, 135.1, 133.3 (C1′), 132.9 (C4″), 130.4 (C5), 130.2 (C5″), 128.9 (C2″), 127.3 (C2′), 126.9 (C6″), 114.1 (C3), 113.8 (C4a), 112.9 (C8), 111.0 (C6), 95.0 (−OCH_2_O*C*H_3_), 56.9 (*−OC*H_3_); HRMS (ESI) *m*/*z* [M + H]^+^ calcd for C_20_H_16_O_5_Cl 371.0681, found 371.0677.

#### 8-[(2*E*)-3-(3-Bromophenyl)prop-2-enoyl]-7-hydroxy-2*H*-chromen-2-one (**14an**)

Obtained from **12an** (100 mg, 0.25 mmol) as an off-white solid; eluent for chromatography: hexanes–ethyl acetate mixtures of increasing polarity (9:1 (v/v) to 5:1 (v/v)); yield: 24 mg (0.06 mmol, 26%); mp 178–181 °C; IR (ATR) ν 1740 (s), 1631 (s), 1599 (s), 1548 (s), 1242 (s), 1190 (s), 1110 (s) cm^–1^; ^1^H NMR (400 MHz, CDCl_3_) δ 13.78 (s, 1H, -O*H*), 8.24 (d, *J* = 15.5 Hz, 1H, H2′), 7.85 (d, *J* = 15.5 Hz, 1H, H3′), 7.83 (s, 1H, H2″), 7.70 (dm, *J* = 7.8 Hz, 1H, H6″), 7.66 (d, *J* = 9.5 Hz, 1H, H4), 7.56 (dm, *J* = 7.7 Hz, 1H, H4″), 7.54 (d, *J* = 8.7 Hz, 1H, H5), 7.33 (t, *J* = 7.9 Hz, 1H, H5″), 6.94 (d, *J* = 8.7 Hz, 1H, H6), 6.31 (d, *J* = 9.5 Hz, 1H, H3); ^13^C{^1^H} NMR (101 MHz, CDCl_3_) δ 193.2 (C1′), 167.8 (C7), 159.4 (C2), 155.7 (C8a), 144.4 (C4), 144.1 (C3′), 137.1 (C1″), 134.9 (C5), 133.8 (C4″), 132.3 (C2″), 130.8 (C5″), 127.6 (C2′), 127.1 (C6″), 123.3 (C3″), 115.9 (C6), 112.3 (C3), 111.3 (C4a), 109.8 (C8); HRMS (ESI) *m*/*z* [M + H]^+^ calcd for C_18_H_11_O_4_^79^Br 370.9913, found 370.9916.

#### 7-(Methoxymethoxy)-8-[(1*E*)-3-(3-methoxyphenyl)-3-oxoprop-1-en-1-yl]-2*H*-chromen-2-one (**13ap**)

Obtained from **12ap** (88 mg, 0.25 mmol) as an off-white solid; eluent for chromatography: hexanes–ethyl acetate mixtures of increasing polarity (9:1 (v/v) to 5:1 (v/v)); yield: 21 mg (0.06 mmol, 24%); mp 107–110 °C; IR (ATR) ν 2943 (w), 1723 (s), 1661 (m), 1604 (m), 1581 (s), 1446 (m), 1286 (s) cm^–1^; ^1^H NMR (400 MHz, CDCl_3_) δ 8.30 (d, *J* = 16.2 Hz, 1H), 8.20 (d, *J* = 16.2 Hz, 1H), 7.73–7.55 (m, 3H), 7.47–7.37 (m, 2H), 7.17–7.10 (m, 2H), 6.33 (d, *J* = 9.5 Hz, 1H), 5.36 (s, 2H), 3.90 (s, 3H), 3.53 (s, 3H); ^13^C{^1^H} NMR (100 MHz, CDCl_3_) δ 191.0, 160.1, 160.0, 159.7, 154.2, 143.8, 139.7, 132.5, 130.1, 129.8, 127.9, 121.4, 120.0, 114.0, 113.8, 113.1, 112.7, 111.0, 95.0, 56.9, 55.6; HRMS (ESI) *m*/*z* [M + H]^+^ calcd for C_21_H_19_O_6_ 367.1182, found 367.1183.

#### 7-(Methoxymethoxy)-8-[(1*E*)-3-(3-methylphenyl)-3-oxoprop-1-en-1-yl]-2*H*-chromen-2-one (**13aq**)

Obtained from **12aq** (84 mg, 0.25 mmol) as an off-white solid; eluent for chromatography: hexanes–ethyl acetate mixtures of increasing polarity (9:1 (v/v) to 5:1 (v/v)); yield: 32 mg (0.09 mmol, 37%); mp 151–154 °C; IR (ATR) ν 2922 (w), 1730 (m), 1661 (m), 1591 (s), 1277 (m), 1245 (s), 1116 (s), 1046 (m) cm^–1^; ^1^H NMR (400 MHz, CDCl_3_) δ 8.29 (d, *J* = 16.2 Hz, 1H, H2′), 8.20 (d, *J* = 16.2 Hz, 1H, H1′), 7.93–7.86 (m, 2H, H2″-H6″), 7.67 (d, *J* = 9.5 Hz, 1H, H4), 7.43 (d, *J* = 8.7 Hz, 1H, H5), 7.44–7.38 (m, 2H, H2″-H6″), 7.16 (d, *J* = 8.8 Hz, 1H, H6), 6.34 (d, *J* = 9.6 Hz, 1H, H3), 5.37 (s, 2H, −OC*H*_2_O−), 3.54 (s, 3H, −OCH_2_OC*H*_3_), 2.46 (s, 3H, −C*H*_3_); ^13^C{^1^H} NMR (100 MHz, CDCl_3_) δ 191.6 (C3′), 160.2 (C2), 159.6 (C7), 154.1 (C8a), 143.8 (C4), 138.6 (C3″), 138.4 (C1″), 133.8 (C2″, C4″-C6″), 132.3 (C1′), 130.0 (C5), 129.4 (C2″, C4″-C6″), 128.7 (C2″, C4″-C6″), 128.3 (C2′), 126.1 (C2″, C4″-C6″), 114.1 (C3), 113.8 (C4a), 113.3 (C8), 111.0 (C6), 95.0 (*−OC*H_2_OCH_3_), 56.9 (−OCH_2_O*C*H_3_), 21.6 (−*C*H_3_); HRMS (ESI) *m*/*z* [M + H]^+^ calcd for C_21_H_19_O_5_ 351.1227, found 351.1220.

#### Methyl 3-[(1*E*)-3-(7-hydroxy-2-oxo-2*H*-chromen-8-yl)-3-oxoprop-1-en-1-yl]benzoate (**14ar**)

Obtained from **12ar** (95 mg, 0.25 mmol) as an off-white solid; eluent for chromatography: hexanes–ethyl acetate mixtures of increasing polarity (9:1 (v/v) to 5:1 (v/v)); yield: 42 mg (0.12 mmol, 48%); mp 187–190 °C; IR (ATR) ν 2954 (w), 2924 (w), 1723 (s), 1634 (m), 1598 (s), 1346 (m), 1190 (m), 1113 (m) cm^–1^; ^1^H NMR (400 MHz, CDCl_3_) δ 13.82 (s, 1H, – O*H*), 8.35 (t, *J* = 1.7 Hz, 1H, H2″), 8.32 (d, *J* = 15.6 Hz, 1H, H2′), 8.09 (dt, *J* = 7.8, 1.4 Hz, 1H, H4″), 7.97 (d, *J* = 15.4 Hz, 1H, H3′), 7.97 (dm, *J* = 7.9 Hz, 1H, H6″), 7.66 (d, *J* = 9.5 Hz, 1H, H4), 7.54 (t, *J* = 7.7 Hz, 1H, H5″), 7.54 (d, *J* = 8.7 Hz, 1H, H5), 6.94 (d, *J* = 8.7 Hz, 1H, H6), 6.31 (d, *J* = 9.5 Hz, 1H, H3), 3.96 (s, 3H, −OC*H*_3_); ^13^C{^1^H} NMR (100 MHz, CDCl_3_) δ 193.2 (C1′), 167.9 (C7), 166.6 (−*C*O_2_CH_3_), 159.4 (C2), 155.7 (C8a), 144.7 (C3′), 144.4 (C4), 135.3 (C1″), 134.8 (C5), 132.5 (C6″), 131.9 (C4″), 131.2 (C3″), 130.8 (C2″), 129.4 (C5″), 127.4 (C2′), 115.9 (C6), 112.3 (C3), 111.3 (C4a), 109.8 (C8), 52.5 (−O*C*H_3_); HRMS (ESI) *m*/*z* [M + H]^+^ calcd for C_20_H_14_O_6_ 351.0863, found 351.0898.

#### 7-(Methoxymethoxy)-8-[(1*E*)-3-(3-methylphenyl)-3-oxoprop-1-en-1-yl]-2*H*-chromen-2-one (**13at**)

Obtained from **12at** (88 mg, 0.25 mmol) as an off-white solid; eluent for chromatography: hexanes–ethyl acetate mixtures of increasing polarity (9:1 (v/v) to 5:1 (v/v)); yield: 50 mg (0.14 mmol, 56%); mp 146–148 °C; IR (ATR) ν 2839 (w), 1725 (s), 1653 (m), 1597 (m), 1581 (s), 1438 (m), 1245 (s), 1045 (s) cm^–1^; ^1^H NMR (400 MHz, CDCl_3_) δ 8.21 (d, *J* = 16.3 Hz, 1H, H2′), 8.12 (d, *J* = 16.2 Hz, 1H, H1′), 7.73 (dd, *J* = 7.7, 1.8 Hz, 1H, H6″), 7.64 (d, *J* = 9.5 Hz, 1H, H4), 7.49 (ddd, *J* = 8.5, 7.5, 1.7 Hz, 1H, H4″), 7.39 (d, *J* = 8.7 Hz, 1H, H5), 7.13 (d, *J* = 8.7 Hz, 1H, H6), 7.06–7.00 (m, 2H, H3″, H5″), 6.29 (d, *J* = 9.5 Hz, 1H, H3), 5.34 (s, 2H, −OC*H*_2_O−), 3.99 (s, 3H, −OCH_2_OC*H*_3_), 3.51 (s, 3H, −OC*H*_3_); ^13^C{^1^H} NMR (100 MHz, CDCl_3_) δ 193.1 (C3′), 160.2 (C2), 159.5 (C7), 158.9 (C2″), 154.1 (C8a), 143.8 (C4), 133.4 (C4″), 132.5 (C2′), 131.0 (C6″), 130.7 (C1′), 129.8 (C5), 129.2 (C1″), 120.8 (C5″), 113.9 (C3), 113.8 (C4a), 113.4 (C8), 111.9 (C3″), 111.0 (C6), 94.9 (*−OC*H_2_O−), 56.8 (−OCH_2_O*C*H_3_), 56.0 (*−OC*H_3_); HRMS (ESI) *m*/*z* [M + H]^+^ calcd for C_21_H_19_O_6_ 367.1182, found 367.1175.

#### Methyl 2-[(1*E*)-3-(7-Hydroxy-2-oxo-2*H*-chromen-8-yl)-3-oxoprop-1-en-1-yl]benzoate (**14au**)

Obtained from **12au** (96 mg, 0.25 mmol) as a yellow solid; eluent for chromatography: hexanes–ethyl acetate mixtures of increasing polarity (9:1 (v/v) to 5:1 (v/v)); yield: 80 mg (0.23 mmol, 92%); mp 214–218 °C; IR (ATR) ν 2959 (w), 1723 (s), 1713 (s), 1599 (s), 1235 (m), 1266 (m), 1195 (m), 1116 (s), 1031 (m) cm^–1^; ^1^H NMR (400 MHz, CDCl_3_) δ 13.78 (s, 1H, -O*H*), 8.73 (d, *J* = 15.5 Hz, 1H, H3′), 8.05 (d, *J* = 15.5 Hz, 1H, H2′), 8.01–7.97 (m, 2H, H3″, H6″), 7.66 (d, *J* = 9.5 Hz, 1H, H4), 7.64 (t, *J* = 7.5 Hz, 1H, H5″), 7.53 (d, *J* = 8.9 Hz, 1H, H5), 7.48 (t, *J* = 7.8 Hz, 1H, H4″), 6.94 (d, *J* = 8.8 Hz, 1H, H6), 6.29 (d, *J* = 9.5 Hz, 1H, H3), 3.96 (s, 3H, −OC*H*_3_); ^13^C{^1^H} NMR (101 MHz, CDCl_3_) δ 193.6 (C1′), 167.7 (C7), 167.3 (-*C*O_2_CH_3_), 159.6 (C2), 155.7 (C8a), 144.8 (C3′), 144.4 (C4), 136.8 (C1″), 134.7 (C5), 133.0 (C5″), 130.9 (C3″), 130.5 (C2″), 130.0 (C4″), 128.9 (C2′), 128.7 (C6″), 115.9 (C6), 112.2 (C3), 111.2 (C4a), 109.8 (C8), 52.6 (*−OC*H_3_); HRMS (ESI) *m*/*z* [M + H]^+^ calcd for C_20_H_15_O_6_ 351.0863, found 351.0862.

#### (*E*)-7-(Methoxymethoxy)-2-(4-(methoxymethoxy)phenyl)-8-(3-(4-methoxyphenyl)-3-oxoprop-1-en-1-yl)chroman-4-one (**17af**)

Obtained from **16af** (240 mg, 0.49 mmol) as a yellow solid; eluent for chromatography: hexanes–ethyl acetate mixtures of increasing polarity (9:1 (v/v) to 5:1 (v/v)); yield: 120 mg, (0.24 mmol, 49%); mp 143–144 °C; IR (ATR) ν 2839 (w), 1673 (m), 1654 (m), 1578 (s), 1256 (m), 1026 (s) cm^–1^; ^1^H NMR (400 MHz, CDCl_3_) δ 8.18 (d, *J* = 16.0 Hz, 1H, H1′), 8.06 (d, *J* = 16.0 Hz, 1H, H2′), 7.96 (d, *J* = 8.9 Hz, 1H, H5), 7.75 (d, *J* = 9.0 Hz, 2H, H2″), 7.51 (d, *J* = 8.6 Hz, 2H, H2‴), 7.16 (d, *J* = 8.6 Hz, 2H, H3‴), 6.91 (d, *J* = 8.9 Hz, 1H, H6), 6.84 (d, *J* = 9.0 Hz, 2H, H3″), 5.53 (dd, *J* = 13.6, 2.8 Hz, 1H, H2), 5.35 (s, 2H, C7–OC*H*_2_OCH_3_), 5.24 (s, 2H, C4‴–OC*H*_2_OCH_3_), 3.86 (s, 3H, C4″–OC*H*_3_), 3.52 (s, 3H, C7–OCH_2_OC*H*_3_), 3.52 (s, 3H, C4‴–OCH_2_OC*H*_3_), 3.16 (dd, *J* = 16.8, 13.6 Hz, 1H, H3^ax^), 2.89 (dd, *J* = 16.8, 2.8 Hz, 1H, H3^eq^); ^13^C{^1^H} NMR (100 MHz, CDCl_3_) δ 190.8 (C4), 189.3 (C3′), 163.4 (C4″), 162.7 (C7), 162.4 (C8a), 158.0 (C4‴), 133.0 (C1′), 131.8 (C1‴), 131.4 (C1″), 130.9 (C2″), 130.1 (C5), 128.2 (C2‴), 125.8 (C2′), 116.8 (C3‴), 116.0 (C4a), 113.8 (C3″), 113.5 (8), 108.2 (C6), 94.7 (C7–O*C*H_2_OCH_3_) 94.5 (C4‴*–OC*H_2_OCH_3_), 80.4 (C2), 56.9 (C7–OCH_2_O*C*H_3_), 56.3 (C4‴–OCH_2_O*C*H_3_), 55.5 (C4″*–OC*H_3_), 43.8 (C3); HRMS (EI) *m*/*z* [M^+^] calcd for C_29_H_28_O_8_ 504.1784, found 504.1782.

#### (*E*)-7-Methoxy-2-(4-(methoxymethoxy)phenyl)-8-(3-(4-methoxyphenyl)-3-oxoprop-1-en-1-yl)chroman-4-one (**17bf**)

Obtained from **16bf** (230 mg, 0.50 mmol) as a yellow solid; eluent for chromatography: hexanes–ethyl acetate mixtures of increasing polarity (9:1 (v/v) to 5:1 (v/v)); yield: 85 mg (0.18 mmol, 36%); mp 198–199 °C; IR (ATR) ν 2936 (w), 2837 (w), 1676 (m), 1650 (m), 1601 (s), 1580 (s), 1230 (s), 1081 (s) cm^–1^; ^1^H NMR (400 MHz, CDCl_3_) δ 8.16 (d, *J* = 15.9 Hz, 1H), 8.02 (d, *J* = 15.9 Hz, 1H), 7.98 (d, *J* = 8.8 Hz, 1H), 7.76 (d, *J* = 8.8 Hz, 2H), 7.50 (d, *J* = 8.6 Hz, 2H), 7.15 (d, *J* = 8.6 Hz, 2H), 6.83 (d, *J* = 8.8 Hz, 2H), 6.68 (d, *J* = 8.8 Hz, 1H), 5.52 (dd, *J* = 13.5, 2.8 Hz, 1H), 5.24 (s, 2H), 3.98 (s, 3H), 3.85 (s, 3H), 3.52 (s, 3H), 3.15 (dd, *J* = 16.9, 13.5 Hz, 1H), 2.88 (dd, *J* = 16.9, 2.8 Hz, 1H); ^13^C{^1^H} NMR (100 MHz, CDCl_3_) δ 190.9, 189.4, 165.0, 163.3, 162.3, 157.9, 133.0, 131.9, 131.5, 130.9, 130.3, 128.1, 125.6, 116.7, 115.4, 113.7, 112.7, 105.1, 94.5, 80.3, 56.4, 56.3, 55.5, 43.8; HRMS (EI) *m*/*z* [M^+^] calcd for C_28_H_26_O_7_ 474.1679, found 474.1676.

#### (*E*)-7-(Methoxymethoxy)-2-(4-methoxyphenyl)-8-(3-(4-methoxyphenyl)-3-oxoprop-1-en-1-yl)chroman-4-one (**17cf**)

Obtained from **16cf** (137 mg, 0.30 mmol) as a yellow solid; eluent for chromatography: hexanes–ethyl acetate mixtures of increasing polarity (9:1 (v/v) to 5:1 (v/v)); yield: 104 mg (0.22 mmol, 73%); mp 188–189 °C; IR (ATR) ν 2839 (w), 1674 (m), 1655 (m), 1578 (s), 1256 (m), 1027 (s) cm^–1^; ^1^H NMR (400 MHz, CDCl_3_) δ 8.16 (d, *J* = 16.0 Hz, 1H), 8.04 (d, *J* = 16.0 Hz, 1H), 7.95 (d, *J* = 8.9 Hz, 1H), 7.70 (d, *J* = 8.8 Hz, 2H), 7.51 (d, *J* = 8.6 Hz, 2H), 7.03 (d, *J* = 8.6 Hz, 2H), 6.91 (d, *J* = 8.9 Hz, 1H), 6.79 (d, *J* = 8.8 Hz, 2H), 5.52 (dd, *J* = 13.7, 2.7 Hz, 1H), 5.35 (s, 2H), 3.88 (s, 3H), 3.86 (s, 3H), 3.52 (s, 3H), 3.19 (dd, *J* = 16.9, 13.7 Hz, 1H), 2.89 (dd, *J* = 16.9, 2.7 Hz, 1H); ^13^C{^1^H} NMR (100 MHz, CDCl_3_) δ 190.9, 189.3, 163.3, 162.7, 162.4, 160.3, 132.9, 131.5, 130.9, 130.6, 130.1, 128.4, 125.8, 116.0, 114.5, 113.7, 113.5, 108.2, 94.7, 80.5, 56.9, 55.5, 55.5, 43.6; HRMS (EI) *m*/*z* [M^+^] calcd for C_28_H_26_O_7_ 474.1679, found 474.1692.

#### 7-Methoxy-3-(4-methoxyphenyl)-8-[(1*E*)-3-(4-methoxyphenyl)-3-oxoprop-1-en-1-yl]-4*H*-chromen-4-one (**21**)

Obtained from **20** (180 mg, 0.42 mmol) as a yellow solid; eluent for chromatography: hexanes–ethyl acetate mixtures of increasing polarity (9:1 (v/v) to 5:1 (v/v)); yield: 96 mg (0.21 mmol, 50%); mp 177–178 °C; IR (ATR) ν 1660 (s), 1594 (s), 1513 (m), 1417 (m), 1296 (s), 1172 (s) cm^–1^; ^1^H NMR (400 MHz, CDCl_3_) δ 8.29 (d, *J* = 9.0 Hz, 1H, H5), 8.24 (d, *J* = 16.0 Hz, 1H, H1′), 8.07 (d, *J* = 16.0 Hz, 1H, H2′), 8.06 (d, *J* = 8.7 Hz, 2H, H2″), 8.03 (s, 1H, H2), 7.52 (d, *J* = 8.7 Hz, 2H, H2‴), 7.06 (d, *J* = 9.0 Hz, 1H, H6), 6.98 (d, *J* = 8.7 Hz, 2H, H3″ or H3‴), 6.97 (d, *J* = 8.7 Hz, 2H, H3″ or H3‴), 4.06 (s, 3H, C7–OC*H*_3_), 3.88 (s, 3H, C4″–OC*H*_3_), 3.84 (s, 3H, C4‴–OC*H*_3_); ^13^C{^1^H} NMR (100 MHz, CDCl_3_) δ 189.6 (C3′), 175.9 (C4), 163.5 (C4″), 162.9 (C7), 159.8 (C4‴), 156.0 (C8a), 152.1 (C2), 132.1 (C1′), 131.3 (C1″), 131.0 (C2″), 130.2 (C2‴), 129.3 (C5), 126.8 (C2′), 124.8 (C3), 123.9 (C1‴), 118.7 (C4a), 114.1 (C3″ or C3‴), 114.0 (C3″ or C3‴), 112.4 (C8), 109.3 (C6), 56.6 (C7–OCH_3_), 55.6 (C4″–OCH_3_), 55.4 (C4‴–OCH_3_); HRMS (EI) *m*/*z* [M^+^] calcd for C_27_H_22_O_6_ 442.1416, found 442.1410.

### General Procedure for the Cleavage of MOM-Protecting Groups

To a solution of the respective MOM-ether **17** (0.5 mmol, 1.0 equiv) in methanol (10 mL, 0.05 M in **17**) was added aqueous HCl (4 M, 1.5 mmol, 3.0 equiv per MOM-group) and the mixture was heated at 60 °C for 2 h. It was then cooled to ambient temperature, diluted with water (20 mL), and extracted with ethyl acetate (three times 20 mL). The combined organic extracts were dried with MgSO_4_ and filtered, and all volatiles were evaporated in vacuo. The residue was purified by column chromatography on silica, using hexanes–ethyl acetate mixtures as eluent.

#### (*E*)-7-Hydroxy-2-(4-hydroxyphenyl)-8-(3-(4-methoxyphenyl)-3-oxoprop-1-en-1-yl)chroman-4-one (**18af**)

Obtained from **17af** (75 mg, 0.15 mmol) as a yellow solid; eluent for chromatography: hexanes–ethyl acetate mixtures of increasing polarity (3:1 (v/v) to 1:1 (v/v)); yield: 40 mg (0.10 mmol, 67%); mp 170–171 °C; IR (ATR) ν 3270 (w), 2972 (w), 1657 (m), 1601 (s), 1576 (s), 1548 (s), 1234 (s), 1165 (s) cm^–1^; ^1^H NMR (400 MHz, DMSO-*d*_6_) δ 11.52 (s,1H), 9.79 (s, 1H), 8.01 (d, *J* = 15.9 Hz, 1H), 7.96 (d, *J* = 15.9 Hz, 1H), 7.75 (d, *J* = 8.8 Hz, 1H), 7.60 (d, *J* = 8.7 Hz, 2H), 7.50 (d, *J* = 8.4 Hz, 2H), 6.94 (d, *J* = 8.7 Hz, 2H), 6.91 (d, *J* = 8.4 Hz, 2H), 6.71 (d, *J* = 8.8 Hz, 1H), 5.63 (dd, *J* = 13.8, 2.6 Hz, 1H), 3.85 (s, 3H), 3.35 (dd, *J* = 16.8, 13.8 Hz, 1H), 2.71 (dd, *J* = 16.8, 2.6 Hz, 1H); ^13^C{^1^H} NMR (100 MHz, DMSO-*d*_6_) δ 190.3, 187.7, 164.2, 163.0, 162.8, 158.1, 133.1, 130.7, 130.2, 129.7, 128.9, 128.9, 123.5, 115.4, 113.9, 113.7, 109.9, 109.9, 79.9, 55.5, 42.2; HRMS (ESI) *m*/*z* [M + H]^+^ calcd for C_25_H_21_O_6_ 417.1338, found 417.1348.

#### (*E*)-2-(4-Hydroxyphenyl)-7-methoxy-8-(3-(4-methoxyphenyl)-3-oxoprop-1-en-1-yl)chroman-4-one (**18bf**)

Obtained from **17bf** (40 mg, 0.08 mmol) as a yellow solid; eluent for chromatography: hexanes–ethyl acetate mixtures of increasing polarity (3:1 (v/v) to 1:1 (v/v)); yield: 34 mg (0.08 mmol, quant.); mp 214–215 °C; IR (ATR) ν 3279 (w), 2832 (w), 1682 (m), 1588 (s), 1552 (m), 1231 (s), 1162 (s) cm^–1^; ^1^H NMR (400 MHz, DMSO-*d*_6_) δ 9.82 (s, 1H), 7.98 (d, *J* = 16.0 Hz, 1H), 7.92 (d, *J* = 16.0 Hz, 1H), 7.88 (d, *J* = 8.9 Hz, 1H), 7.57 (d, *J* = 8.0 Hz, 2H), 7.48 (d, *J* = 8.0 Hz, 2H), 6.98–6.83 (m, 4H), 6.89 (d, *J* = 8.9 Hz, 1H), 5.62 (dd, *J* = 13.8, 2.7 Hz, 1H), 3.98 (s, 3H), 3.84 (s, 3H), 3.37 (dd, *J* = 16.7, 13.8 Hz, 1H), 2.74 (dd, *J* = 16.7, 2.7 Hz, 1H); ^13^C{^1^H} NMR (100 MHz, DMSO-*d*_6_) δ 190.7, 187.6, 164.2, 163.0, 161.9, 158.2, 132.3, 130.6, 130.3 (2C), 130.1, 128.9 (2C), 128.8, 124.4, 115.5 (2C), 115.0, 113.9 (2C), 111.2, 105.4, 80.1, 56.8, 55.5, 42.3; HRMS (ESI) *m*/*z* [M + H]^+^ calcd for C_26_H_23_O_6_ 431.1495, found 431.1475.

#### (*E*)-7-Hydroxy-2-(4-methoxyphenyl)-8-(3-(4-methoxyphenyl)-3-oxoprop-1-en-1-yl)chroman-4-one (**18cf**)

Obtained from **17cf** (70 mg, 0.15 mmol) as a yellow solid; eluent for chromatography: hexanes–ethyl acetate mixtures of increasing polarity (3:1 (v/v) to 1:1 (v/v)); yield: 35 mg (0.08 mmol, 54%); mp 186–187 °C; IR (ATR) ν 3120 (w), 2931 (w), 1687 (m), 1636 (m), 1595 (s), 1438 (s), 1254 (s), 1230 (s), 1160 (s) cm^–1^; ^1^H NMR (400 MHz, DMSO-*d*_6_) δ 7.98 (s, 1H), 7.74 (d, *J* = 8.6 Hz, 1H), 7.61 (d, *J* = 8.7 Hz, 2H), 7.60 (d, *J* = 8.5 Hz, 2H), 7.08 (d, *J* = 8.7 Hz, 2H), 6.89 (d, *J* = 8.5 Hz, 2H), 6.70 (d, *J* = 8.6 Hz, 1H), 5.67 (dd, *J* = 13.6, 2.8 Hz, 1H), 3.83 (s, 3H), 3.83 (s, 3H), 3.32 (dd, *J* = 16.8, 13.6 Hz, 1H), 2.72 (dd, *J* = 16.8, 2.8 Hz, 1H); ^13^C{^1^H} NMR (100 MHz, DMSO-*d*_6_) δ 190.3, 187.9, 164.3, 163.0, 162.7, 159.8, 133.2, 130.8, 130.8, 130.3, 129.7, 128.2, 123.6, 114.2, 113.9, 113.7, 110.1, 110.0, 79.8, 55.5, 55.4, 42.2; HRMS (ESI) *m*/*z* [M + H]^+^ calcd for C_26_H_23_O_6_ 431.1495, found 431.1500.

## Data Availability

The data underlying this study are available in the published article and its Supporting Information.

## Supporting Information Available

Crystallographic details and refinement data for compound **16cf**; synthesis procedures, characterization data and copies of spectra for test substrate **11d** and all intermediates; synthesis procedures, characterization data and copies of spectra for **15b** and its precursor; copies of spectra for all products **12**, **13**, **14**, **16**, **17**, **18**, **20**, **21** and **23** (PDF)
FAIR data, including the primary NMR FID files, for compounds **11d**, **12aa**–**12ai**, **12ak**–**12ar**, **12at**, **12au**, **13aa**–**13am**, **13ap**, **13aq**, **13at**, **14ae**, **14af**, **14an**, **14ar**, **14au**, **15b**, **16af**–**16cf**, **17af**–**17cf**, **18af**–**18cf**, **20**, **21**, **23**, **SI**-**2**, **SI**-**3** and **SI-7** (ZIP)
