# Supplementary material for: Synthesis of Benzopyran–Phenylpropanoid Hybrids via Matsuda–Heck-Arylation and Allylic Oxidation
Source: J Org Chem. 2024 Dec 7;89(24):18585–601. doi: 10.1021/acs.joc.4c02520 (PMC11667724; doi:10.1021/acs.joc.4c02520)
Supplement: Supplementary file 1 — jo4c02520_si_001.pdf [file jo4c02520_si_001.pdf]

Supporting Information

Synthesis of Benzopyran-Phenylpropanoid Hybrids via Matsuda-Heck-Arylation and Allylic Oxidation

Fabian Otte,[a] Julia Greese,[a] Stefan Foß,[a] Mandy Krüger,[a] Eric Sperlich,[a] George Kwesiga,[a,b*] Bernd Schmidt[a*]

[a] *Universität Potsdam, Institut für Chemie, Karl-Liebknecht-Straße 24-25, D-14476 Potsdam-Golm, Germany.*

[b] *Kabale University, Department of Chemistry, P.O. Box 317, Kabale, Uganda.*

E-mail: *gekwesiga@kab.ac.ug (George Kwesiga); bernd.schmidt@uni-potsdam.de (Bernd Schmidt)*

Contents:

**A**	**Single crystal X-ray structure analysis of compounds 16cf**	**S2**
**B**	**Syntheses of compounds 11d and 15b**	**S8**
**C**	**Copies of ^{1}H and ^{13}C NMR spectra**	**S25**
**D**	**References**	**S211**

A Single crystal X-ray structure analyses of compounds 16cf

1 General details of X-ray structure analysis

The crystal structures were determined by single crystal structure analysis. Suitable single crystals were selected using a Leica M205C light microscope and separated with oil. **Figure S1** shows a single crystal of **16cf**. The X-ray crystal structure analysis was performed on a Stadivari diffractometer (Stoe) with monochromated Mo-*K*α radiation (λ = 0.71073 Å). The data correction was performed using the program X-Area.[1] The structure was solved by direct methods and refined against F^2 on all data by full-matrix least-squares using the SHELX suite of programs.[2,3] All non-hydrogen atoms were refined anisotropically; the hydrogen atoms were placed on calculated positions. **Table S1** was created using FinalCif.[4] The crystal structure was visualized with Diamond.[5] The data (**16cf**: CCDC 2156920) can be obtained free of charge from The Cambridge Crystallographic Data Centre, http://www.ccdc.cam.ac.uk.

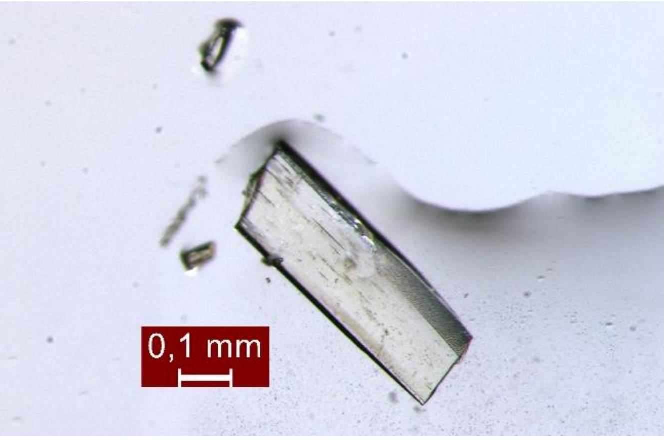

Figure S1: Microscope image of a single crystal of **16cf**.

2 Crystallographic Data:

Table S1. Crystal data and details of structure refinement for **16cf**.

Compound	**16cf**
CCDC number	2156920
Empirical formula	$C_{28}H_{28}O_6$
Formula weight	460.50
Temperature [K]	210
Crystal system	triclinic
Space group	$P\bar{1}$ (2)
a [Å]	10.0329(5)
b [Å]	12.4721(6)
c [Å]	20.1848(9)
α [°]	107.193(4)
β [°]	99.409(4)
γ [°]	91.739(4)
Volume [Å^3]	2372.1(2)
Z	4
ρ_{calc} [gcm^{-3}]	1.289
μ [mm^{-1}]	0.090
F(000)	976
Crystal size [mm^3]	0.400×0.245×0.095
Crystal color	colorless
Crystal shape	needle
Radiation	MoK_α (λ=0.71073 Å)
2θ range [°]	4.30 to 59.98 (0.71 Å)
Index ranges	−14 ≤ h ≤ 12<br>−17 ≤ k ≤ 17<br>−28 ≤ l ≤ 28
Reflections collected	59370
Independent reflections	13808<br>R_{int} = 0.0392<br>R_{sigma} = 0.0374
Completeness to θ = 25°	99.8 %
Data / Restraints / Parameters	13808/92/778
Goodness-of-fit on F^2	1.067
Final R indexes [I≥2σ(I)]	R_1 = 0.0556<br>wR_2 = 0.1528
Final R indexes [all data]	R_1 = 0.1043<br>wR_2 = 0.1735
Largest peak/hole [eÅ^{-3}]	0.43/-0.35

3 Visualization of the crystal structure and molecular structure for compound 16cf

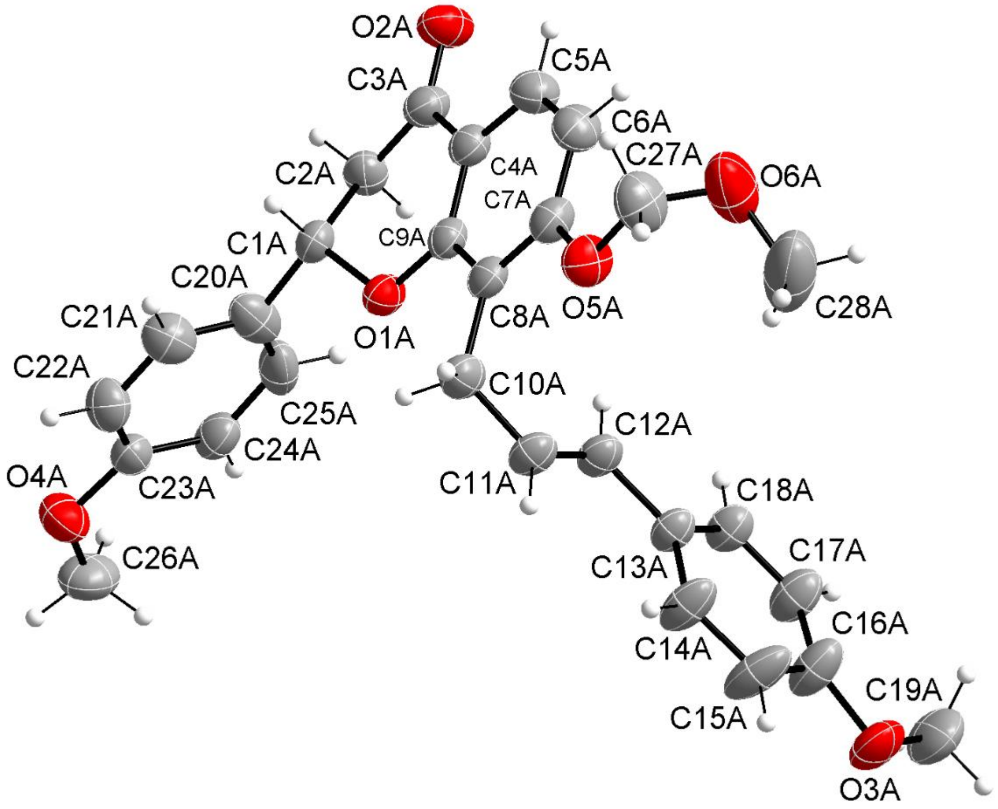

Figure S2: Molecular structure with atom labeling of the symmetry-independent molecule A in **16cf**. Displacement ellipsoids are shown at the 50% probability level.

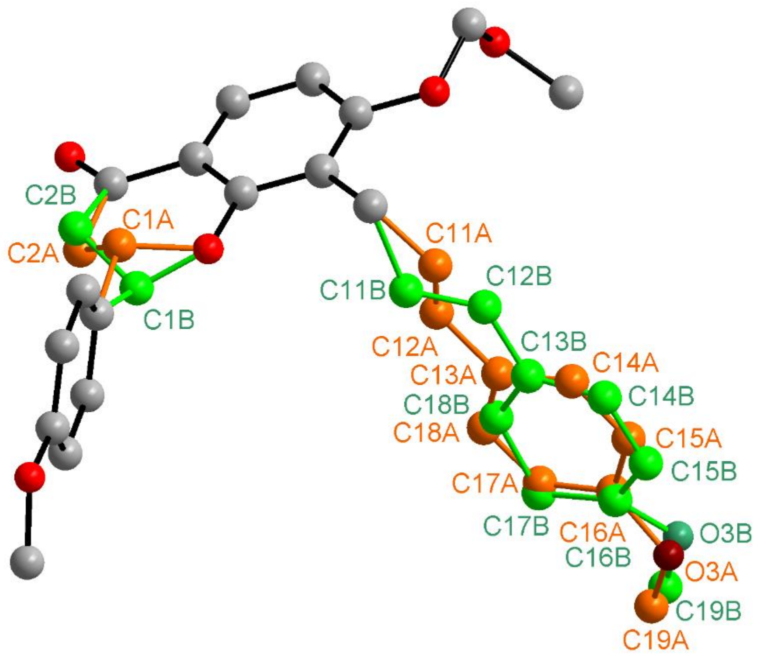

Figure S3: Disorder in the asymmetric molecule A in **16cf** with the main part shown in orange and the minor part shown in green color (hydrogens are omitted).

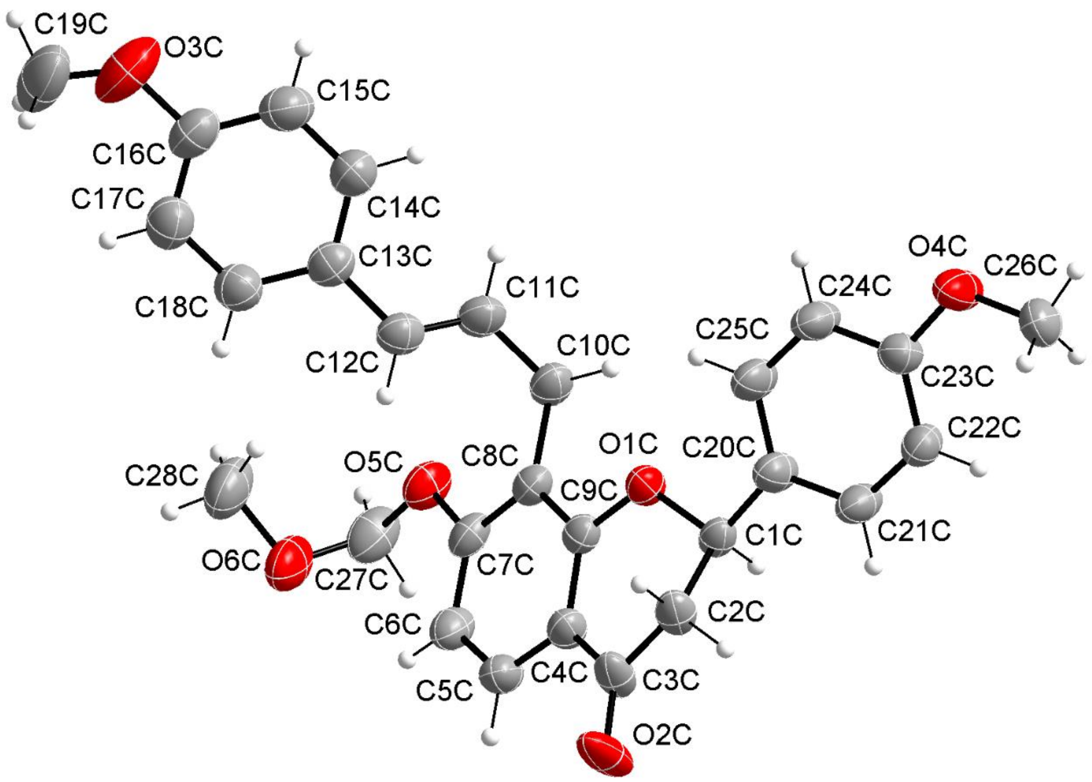

Figure S4: Molecular structure with atom labeling of the symmetry-independent molecule B in **16cf**. Displacement ellipsoids are shown at the 50% probability level.

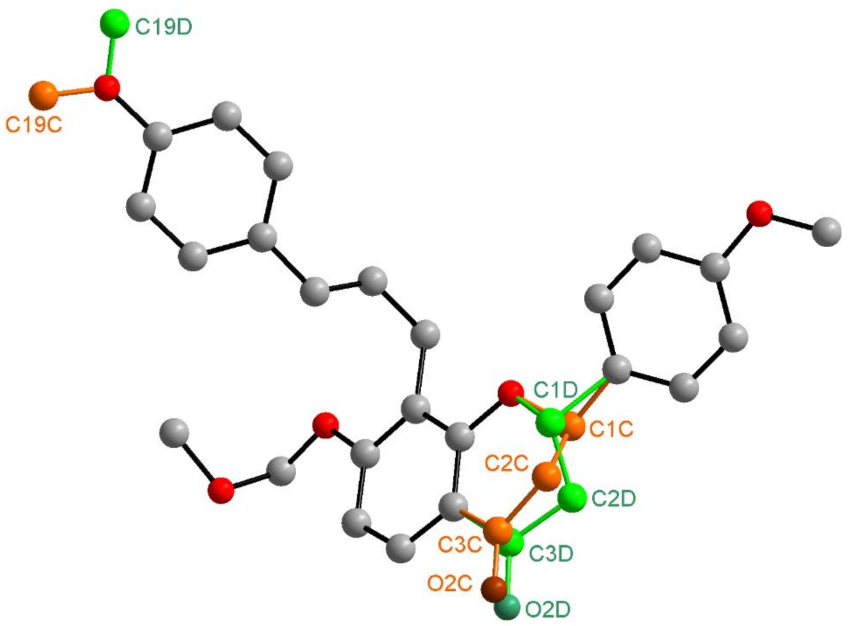

Figure S5: Disorder in the asymmetric molecule B in **16cf** with the main part shown in orange and the minor part shown in green color (hydrogens are omitted).

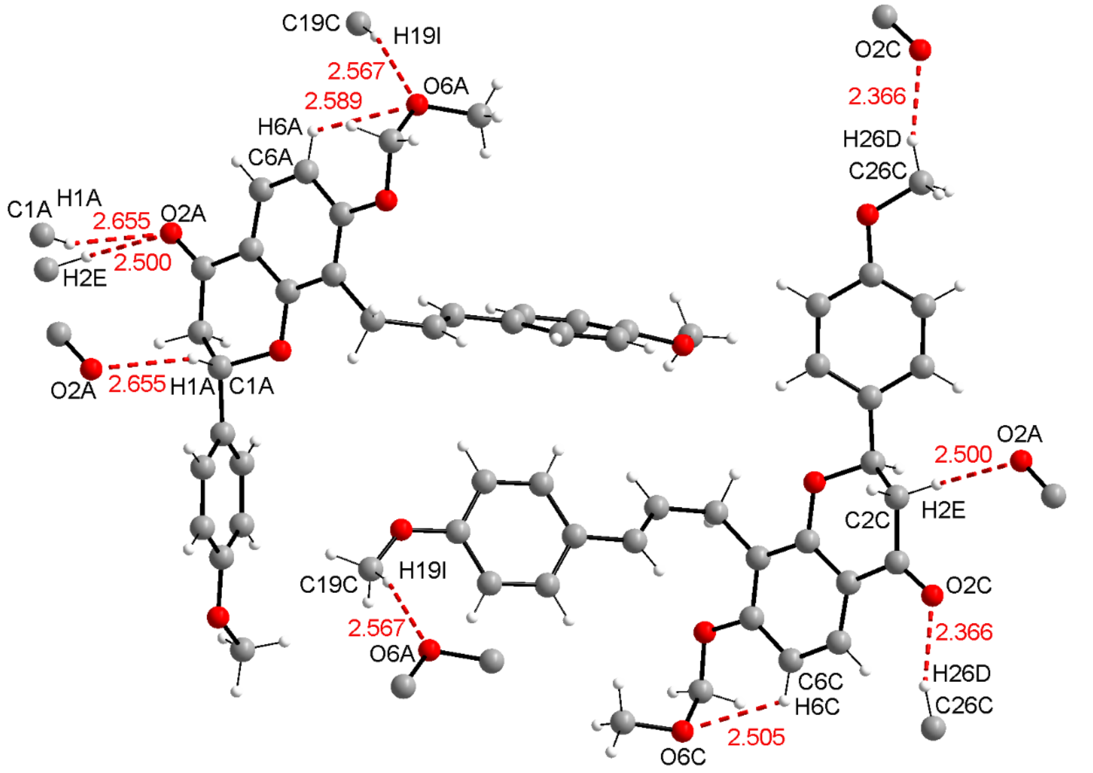

Figure S6: C-H···O hydrogen bonding (red dashed lines) in **16cf**.

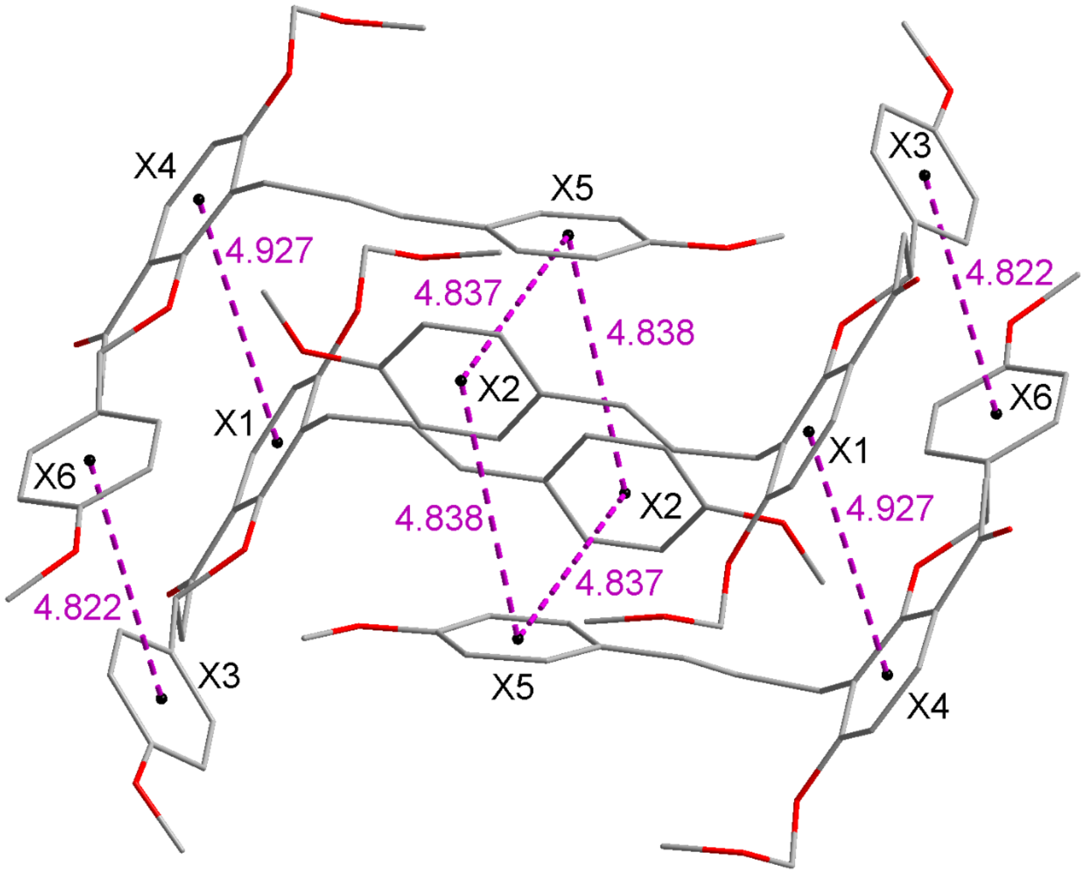

Figure S7: Stacking interactions (purple dotted lines) between the molecules in **16cf**.
(X1, X2 and X3 mark the centers of the aromatics in molecule A, X4, X5 and X6 mark the centers in molecule B, hydrogens are omitted).

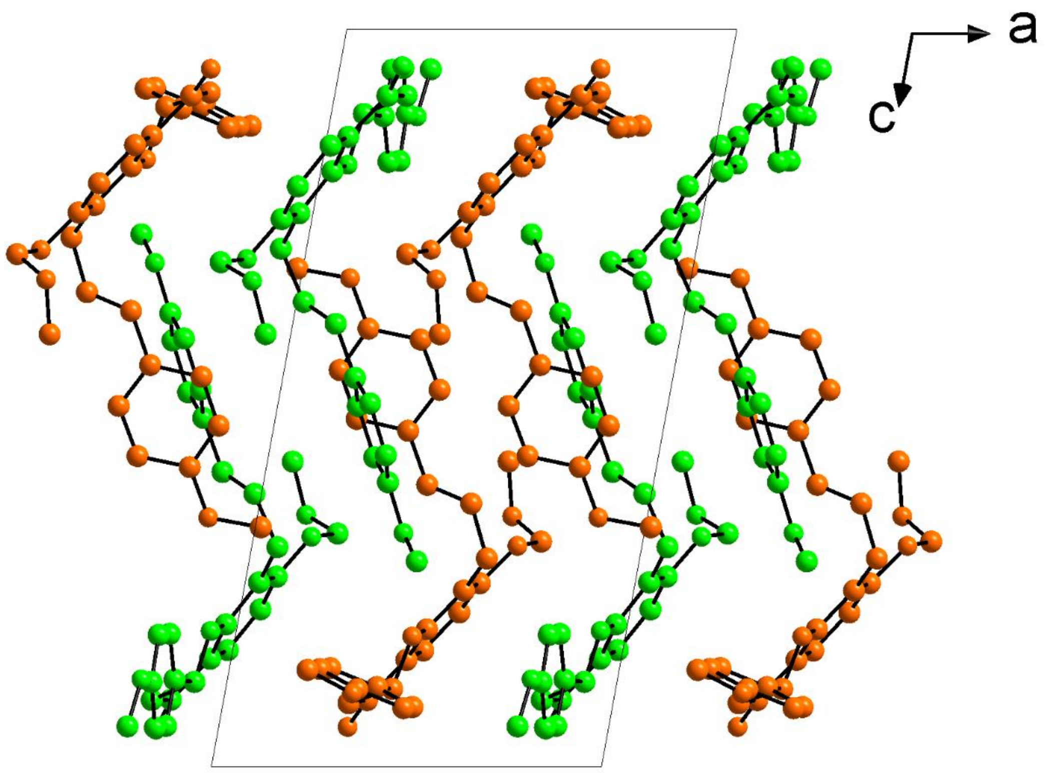

Figure S8: Cell view of **16cf** with the asymmetric molecule A colored in orange and the asymmetric molecule B colored in green looking along the crystallographic b axis (hydrogens are omitted).

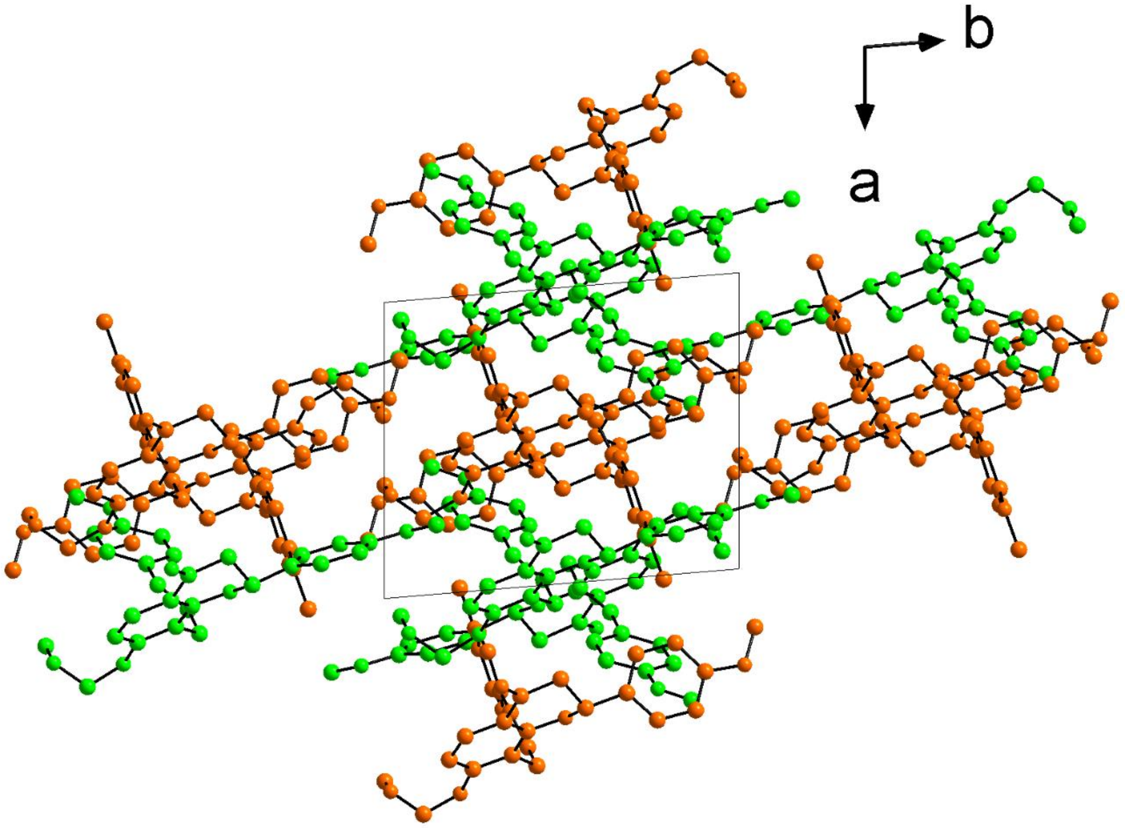

Figure S9: Cell view of **16cf** with the asymmetric molecule A colored in orange and the asymmetric molecule B colored in green looking along the crystallographic c axis (hydrogens are omitted).

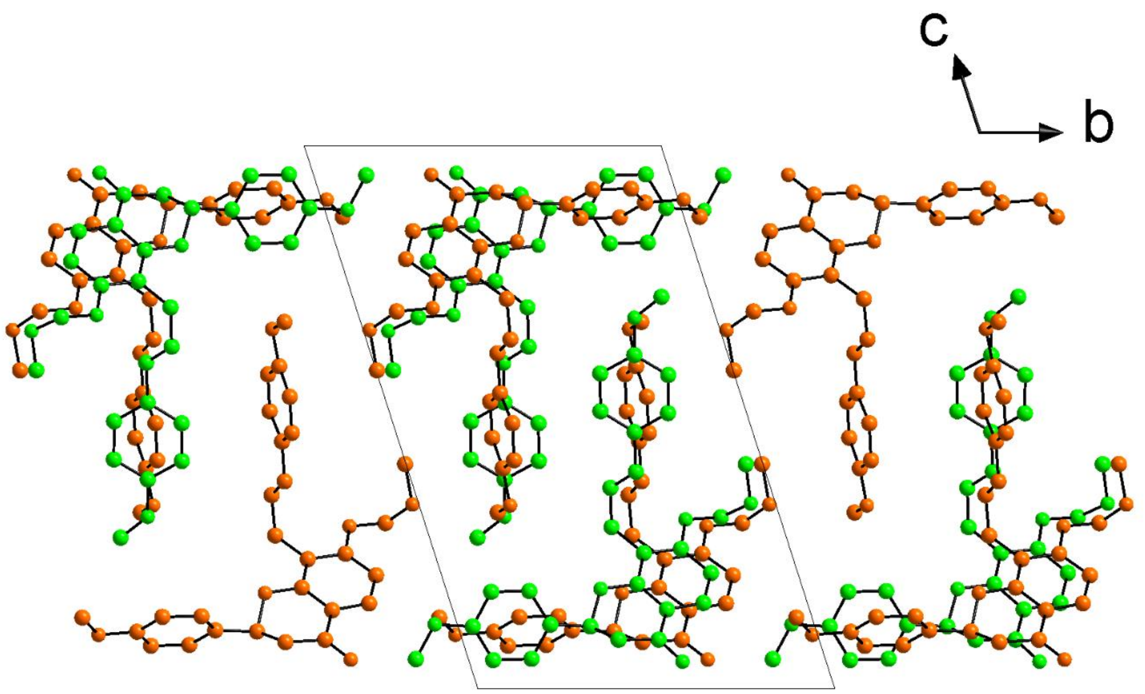

Figure S10: Cell view of **16cf** with the asymmetric molecule A colored in orange and the asymmetric molecule B colored in green looking along the crystallographic a axis (hydrogens are omitted).

B Syntheses of compounds 11d and 15b

1 Synthesis of 5-(prop-2-en-1-yl)-1,2,3,4-tetrahydronaphthalene (11d)

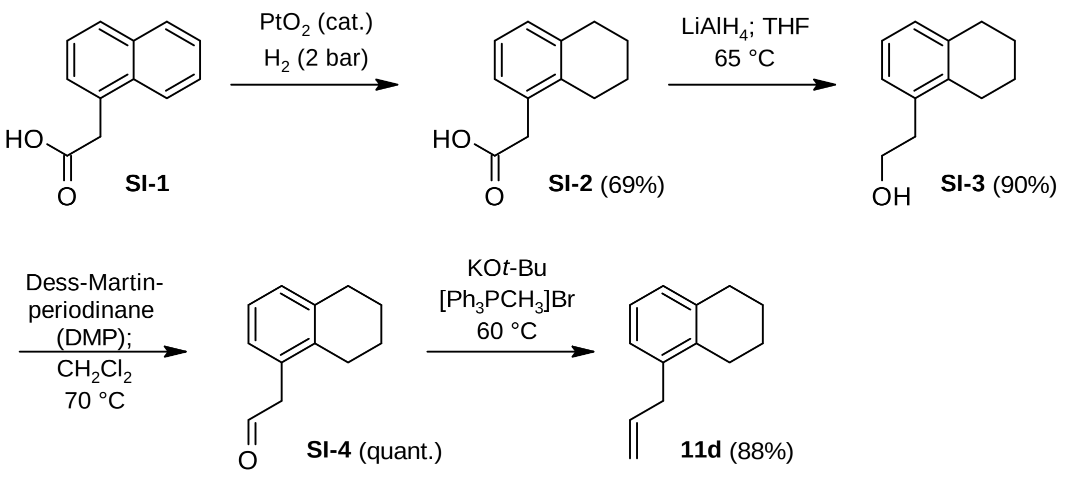

Scheme S1 Synthesis of test substrate **11d**

5,6,7,8-Tetrahydronaphthalen-1-ylacetic acid (SI-2).[6] The title compound was synthesized according to a modified literature procedure:[6] to a solution of **SI-1** (1.86 g, 10.0 mmol) in ethyl acetate (15 mL) was added PtO_2 (180 mg, 0.8 mmol, 8 mol-%) and the solution was purged with hydrogen. The mixture was then stirred under an atmosphere of hydrogen (2 bar) for 3

days. The mixture was filtered through a short pad of celite, washed with ethyl acetate (10 mL), and the combined ethyl acetate solution was evaporated. The residue was recrystallized from hexane to give **SI-2** as colourless crystals; yield: 1.31 g (6.9 mmol, 69%); mp 128 – 130 °C; IR (ATR) ν 2941 (w), 1705 (s), 1406 (m), 1257 (m), 1210 (s), 780 (m) cm^{-1}; ^{1}H NMR (400 MHz, $CDCl_3$) δ 7.12-7.02 (m, 3H), 3.65 (s, 2H), 2.81 (t, J = 6.0 Hz, 2H), 2.70 (t, J = 6.0 Hz, 2H), 1.89-1.74 (m, 4H); $^{13}C\{^{1}H\}$ NMR (100 MHz, $CDCl_3$) δ 178.4, 138.0, 136.0, 132.1, 129.0, 128.0, 125.6, 38.7, 30.2, 26.5, 23.4, 22.8; HRMS (EI) m/z $[M+H]^+$ calcd for $C_{12}H_{15}O_2$ 191.1067, found 191.1072.

2-(5,6,7,8-Tetrahydronaphthalen-1-yl)ethanol (SI-3). Conditions adapted from a literature procedure:[7] under an atmosphere of dry nitrogen, $LiAlH_4$ (0.19 g, 5.3 mmol) was suspended in dry THF (40 mL) and cooled to 0 °C. A solution of **SI-2** (1.00 g, 5.3 mmol) in dry THF (40 mL) was added dropwise, the mixture was warmed to ambient temperature and then heated at 65 °C for 24 h. It was then cooled to ambient temperature, poured onto ice-water mixture (35 mL), and the resulting precipitate was dissolved with aq. H_2SO_4 (1 M). MTBE (25 mL) was added to the mixture, the organic layer was separated, and the aqueous layer was extracted with MTBE (3 times 10 mL). The combined organic extracts were dried with $MgSO_4$, filtered and evaporated. The product was sufficiently pure to be used in the next step without further purification; colourless oil; yield: 0.84 g (4.8 mmol, 90%); IR (ATR) ν 3327 (s, br.), 2927 (s), 2837 (m), 1587 (m), 1458 (m), 1436 (m), 1036 (s), 771 (s), 719 (s) cm^{-1}; ^{1}H NMR (400 MHz, $CDCl_3$) δ 7.11 (t, J = 7.5 Hz, 1H), 7.04 (d, J = 7.5 Hz, 1H), 7.01 (d, J = 7.5 Hz), 3.85 (t, J = 7.0 Hz, 2H), 2.89 (t, J = 7.0 Hz, 2H), 2.83 (t, J = 6.2 Hz, 2H), 2.78 (t, J = 6.2 Hz, 2H), 2.37 (s(br.), 1H), 1.92-1.77 (m, 4H); $^{13}C\{^{1}H\}$ NMR (100 MHz, $CDCl_3$) δ 137.7, 136.4, 135.6, 127.9, 127.0, 125.4, 62.6, 35.9, 30.2, 26.3, 23.5, 22.9; HRMS (EI) m/z $[M^+]$ calcd for $C_{12}H_{16}O$ 176.1196, found 176.1193.

5,6,7,8-Tetrahydronaphthalen-1-ylacetaldehyde (SI-4). Conditions adapted from a literature procedure:[8] to a solution of **SI-3** (400 mg, 2.3 mmol) in CH_2Cl_2 (19 mL) was added Dess-Martin-periodinane (DMP, 1060 mg, 2.5 mmol) in portions at ambient temperature. After stirring for two hours, another portion of DMP (530 mg, 1.3 mmol) was added and stirring was continued for 12 h. The mixture was diluted with diethylether (20 mL), and aq. NaOH (1 M, 20 mL) was added. The organic layer was separated, washed with aq. NaOH (1 M, 10 mL) followed by water (20 mL), and then dried with $MgSO_4$. It was filtered and evaporated to furnish crude **SI-4** (400 mg, 2.3 mmol, quant.). All attempts to purify aldehyde **SI-4** resulted in decomposition; therefore, **SI-4** was used in the following reaction without further purification; ^{1}H NMR (400 MHz, $CDCl_3$) δ 9.72 (t, J = 2.3 Hz, 1H), 7.15 (t, J = 7.5 Hz, 1H), 7.09 (d, J = 7.5

Hz, 1H), 7.04 (d, J = 7.5 Hz, 1H), 3.69 (d, J = 2.3 Hz, 2H), 2.84 (t, J = 6.2 Hz, 2H), 2.64 (t, J = 6.2 Hz, 2H), 1.94-1.72 (m, 4H); $^{13}C\{^{1}H\}$ NMR (100 MHz, $CDCl_3$): δ = 199.7, 138.2, 136.1, 130.7, 129.0, 128.1, 125.8, 48.4, 30.1, 26.8, 23.3, 22.7.

5-(Prop-2-en-1-yl)-1,2,3,4-tetrahydronaphthalene (11d). Conditions adapted from a literature procedure:[9] a solution of methyltriphenylphosphonium bromide (986 mg, 2.8 mmol) in THF (7 mL) was cooled to 0 °C. A solution of BuLi in hexane (2.5 M, 1.01 mL, 2.5 mmol) was slowly added, and the mixture was warmed to ambient temperature and stirred for 0.5 h. The mixture was cooled to 0 °C, a solution of **SI-4** (400 mg, 2.3 mmol) in THF (2.5 mL) was slowly added, and it was warmed to ambient temperature and stirred at ambient temperature for 2 h. The reaction was quenched by addition of methanol (10 mL), and all volatiles were removed in vacuo. The residue was extracted with pentane (three times, 10 mL each), filtered, and evaporated. The residue was purified by column chromatography on silica, using hexanes as eluent; colourless liquid; yield: 350 mg (2.0 mmol, 88%); IR (ATR): 2926 (s), 1637 (m), 1586 (w), 1458 (s), 1436 (s), 993 (s), 909 (s), 772 (s), 728 (s) cm^{-1}; ^{1}H NMR (400 MHz, $CDCl_3$) δ 7.10 (t, J = 7.5 Hz, 1H), 7.05-6.98 (m, 2H), 5.99 (ddt, J = 16.9, 10.1 6.5 Hz, 1H), 5.10 (dq, J = 10.1, 1.6 Hz, 1H), 5.05 (dq, J = 16.9, 1.7 Hz, 1H), 3.77 (dm, J = 6.5 Hz, 2H), 2.83 (t, J = 6.3 Hz, 1H), 2.73 (t, J = 6.3 Hz, 1H), 1.91-1.76 (m, 4H); $^{13}C\{^{1}H\}$ NMR (100 MHz, $CDCl_3$) δ 138.3, 137.5, 136.9, 135.4, 127.6, 126.6, 125.4, 115.7, 37.4, 30.3, 26.2, 23.5, 23.0. No matching HRMS data could be obtained using either EI or ESI.

Figure S11: ^{1}H NMR (400 MHz, $CDCl_3$) of **SI-2**

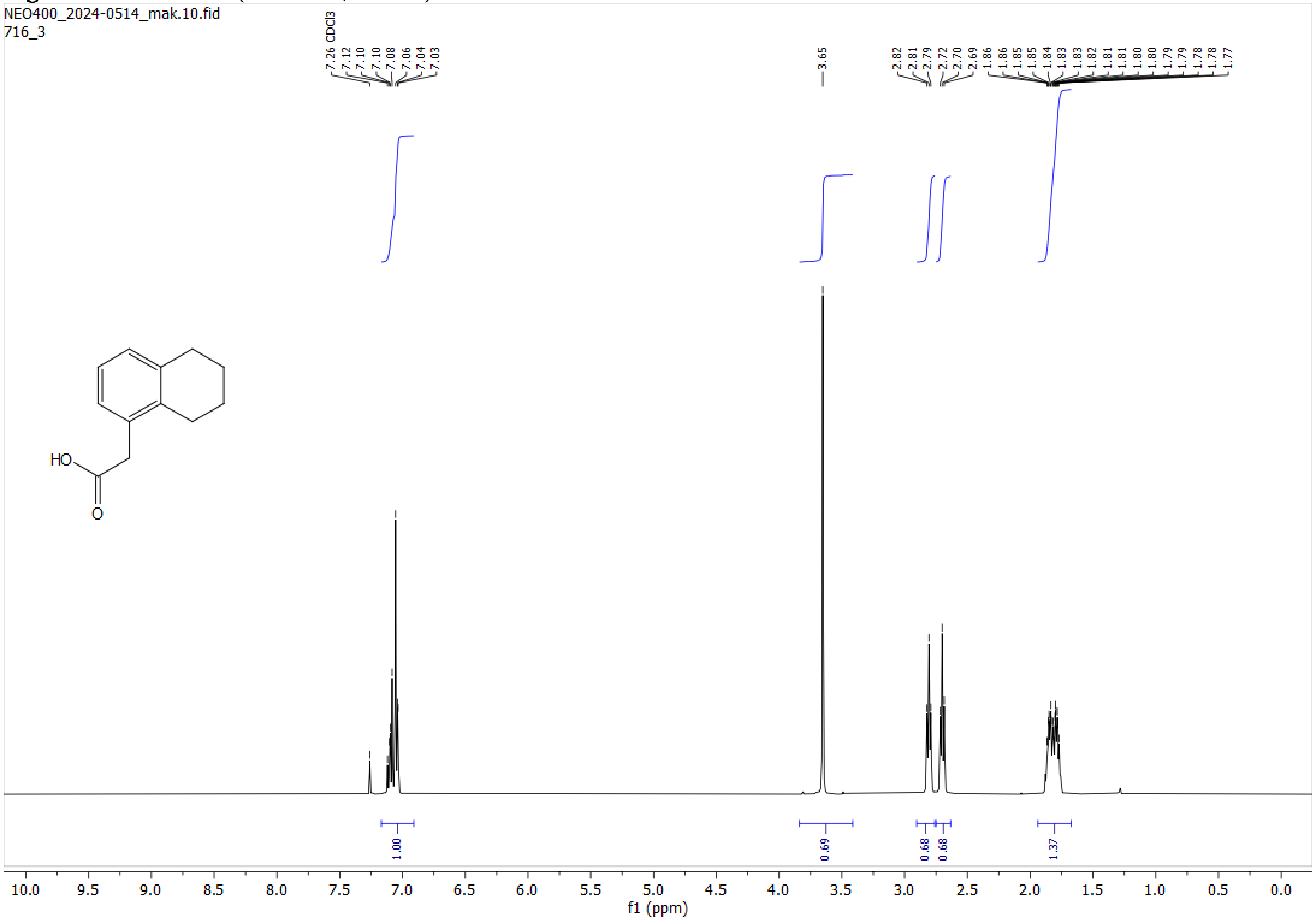

Figure S12: $^{13}C\{^1H\}$ NMR (100 MHz, $CDCl_3$) of **SI-2**

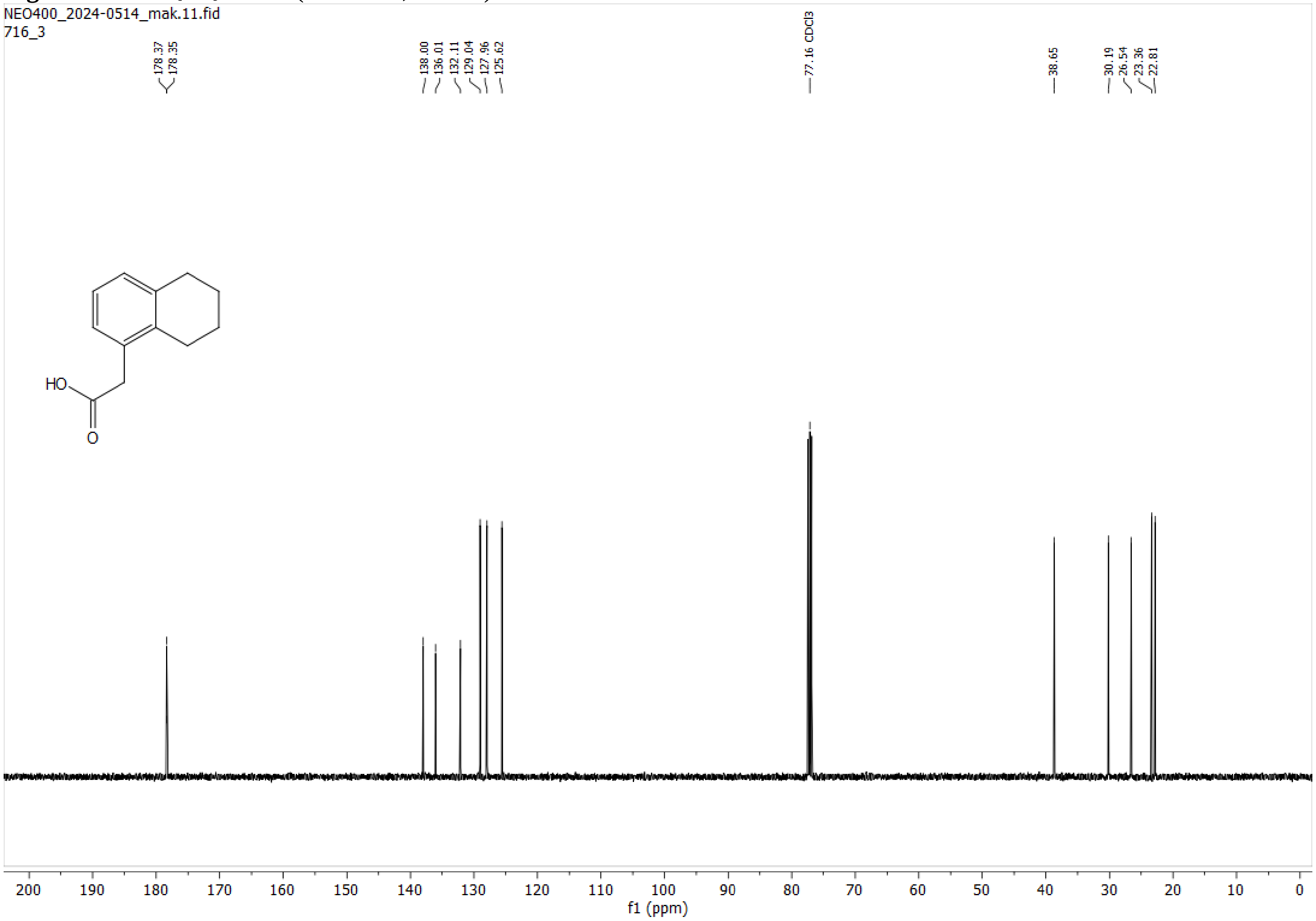

Figure S13: ^{1}H NMR (400 MHz, $CDCl_3$) of **SI-3**

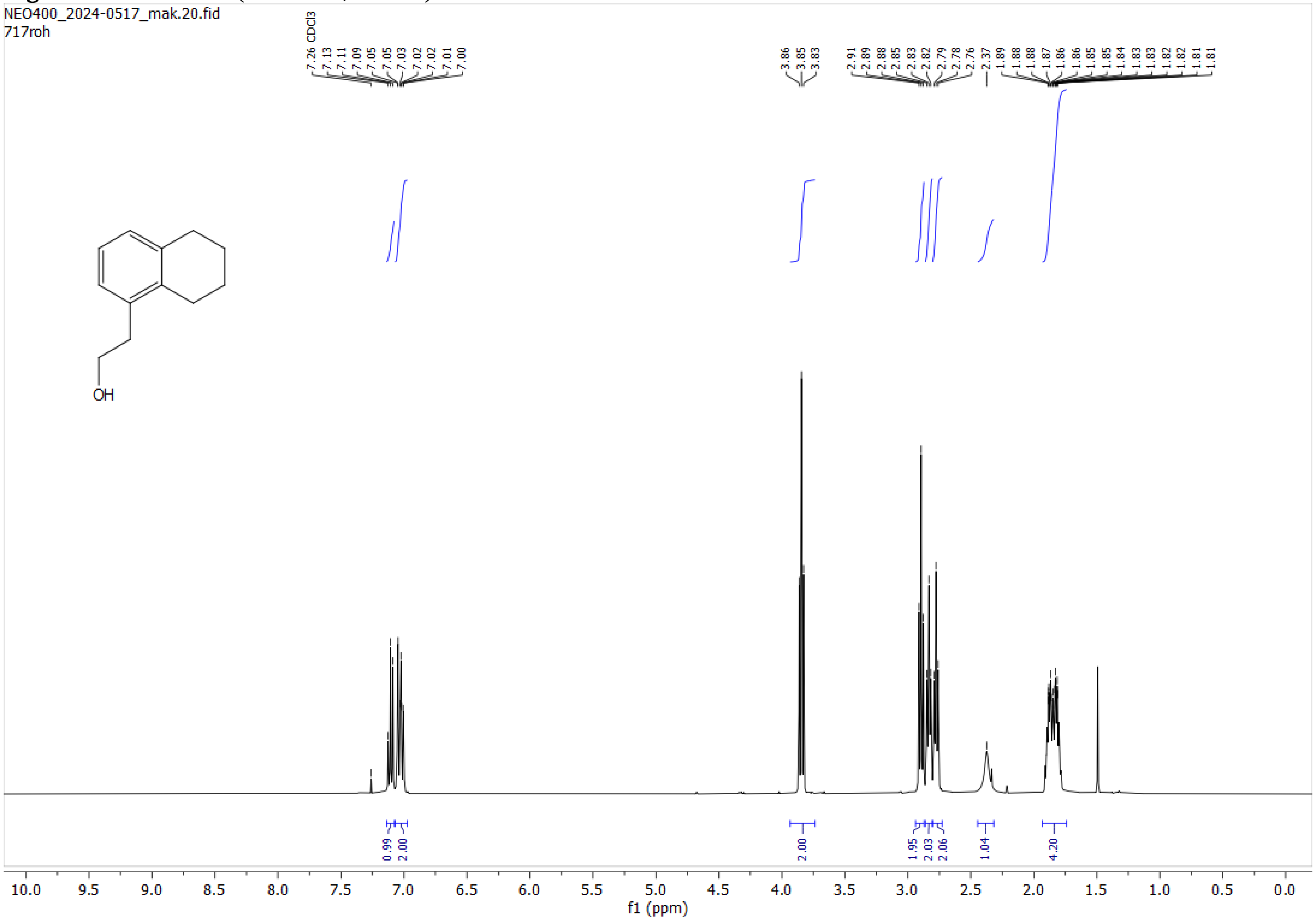

Figure S14: $^{13}C\{^{1}H\}$ NMR (100 MHz, $CDCl_3$) of **SI-3**

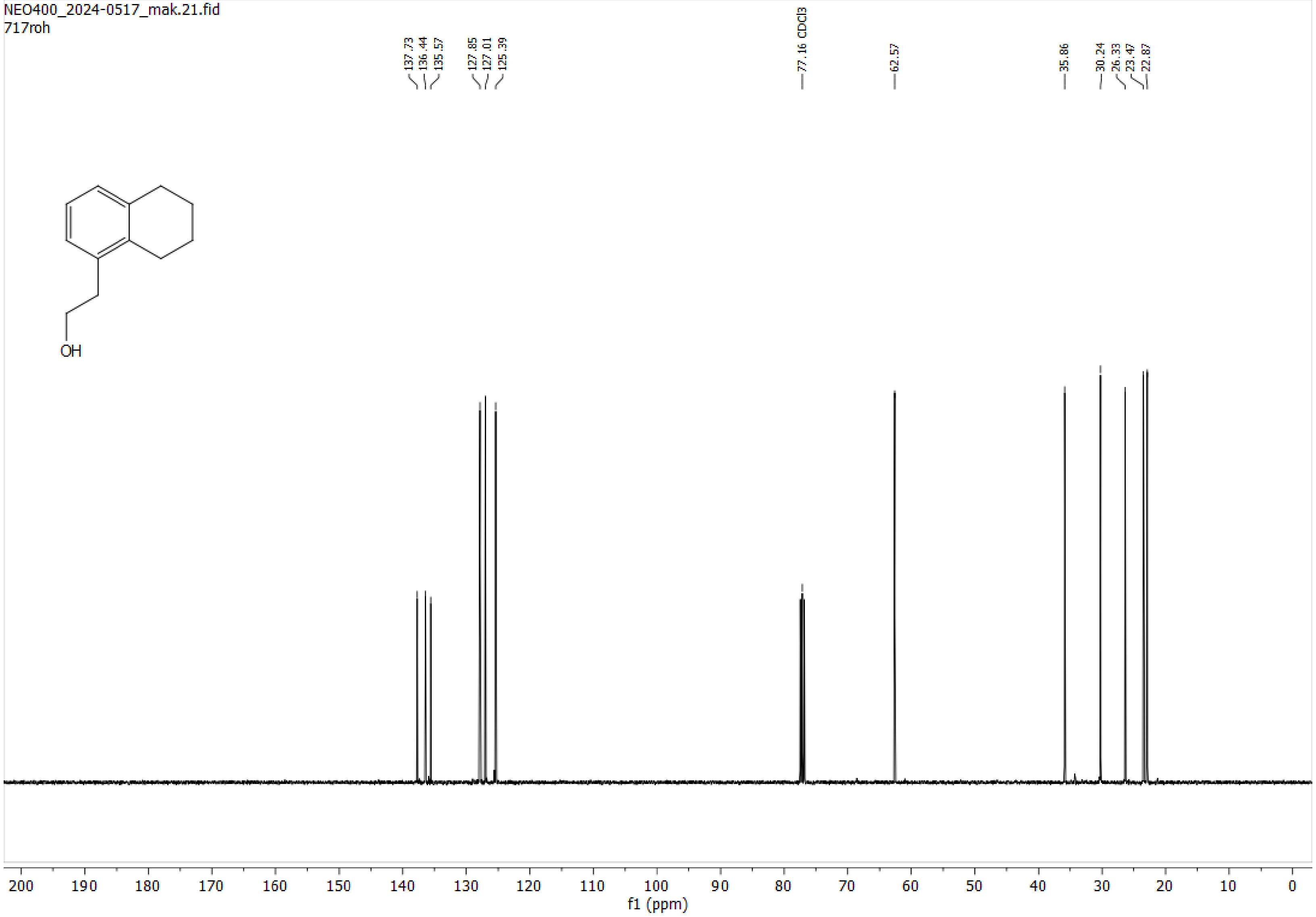

Figure S15: ^{1}H NMR (400 MHz, $CDCl_3$) of **SI-4** (crude reaction mixture, due to decomposition upon purification attempts)

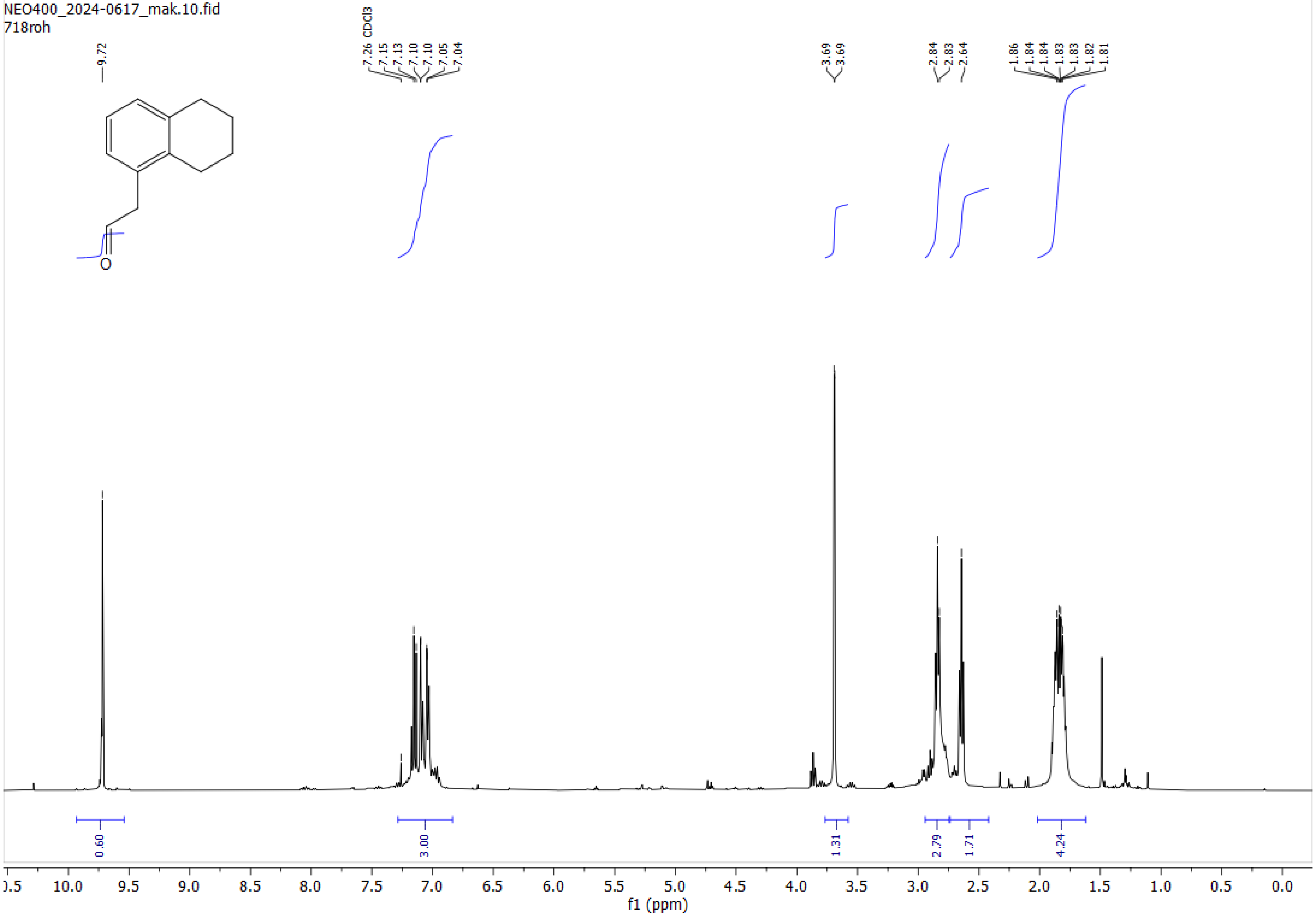

Figure S16: $^{13}C\{^{1}H\}$ NMR (100 MHz, $CDCl_3$) of **SI-4** (crude reaction mixture, due to decomposition upon purification attempts)

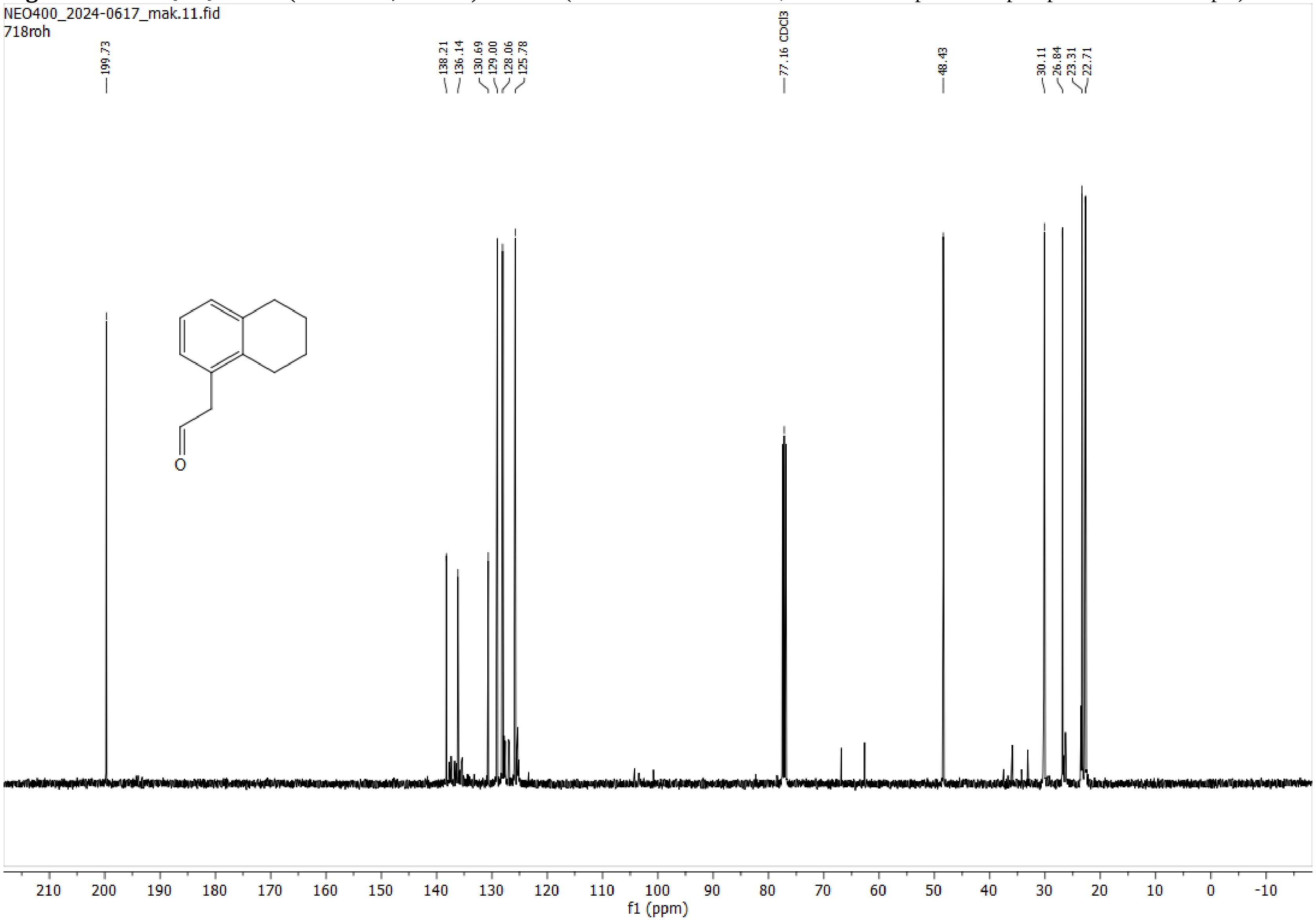

Figure S17: ^{1}H NMR (400 MHz, $CDCl_3$) of **11d**

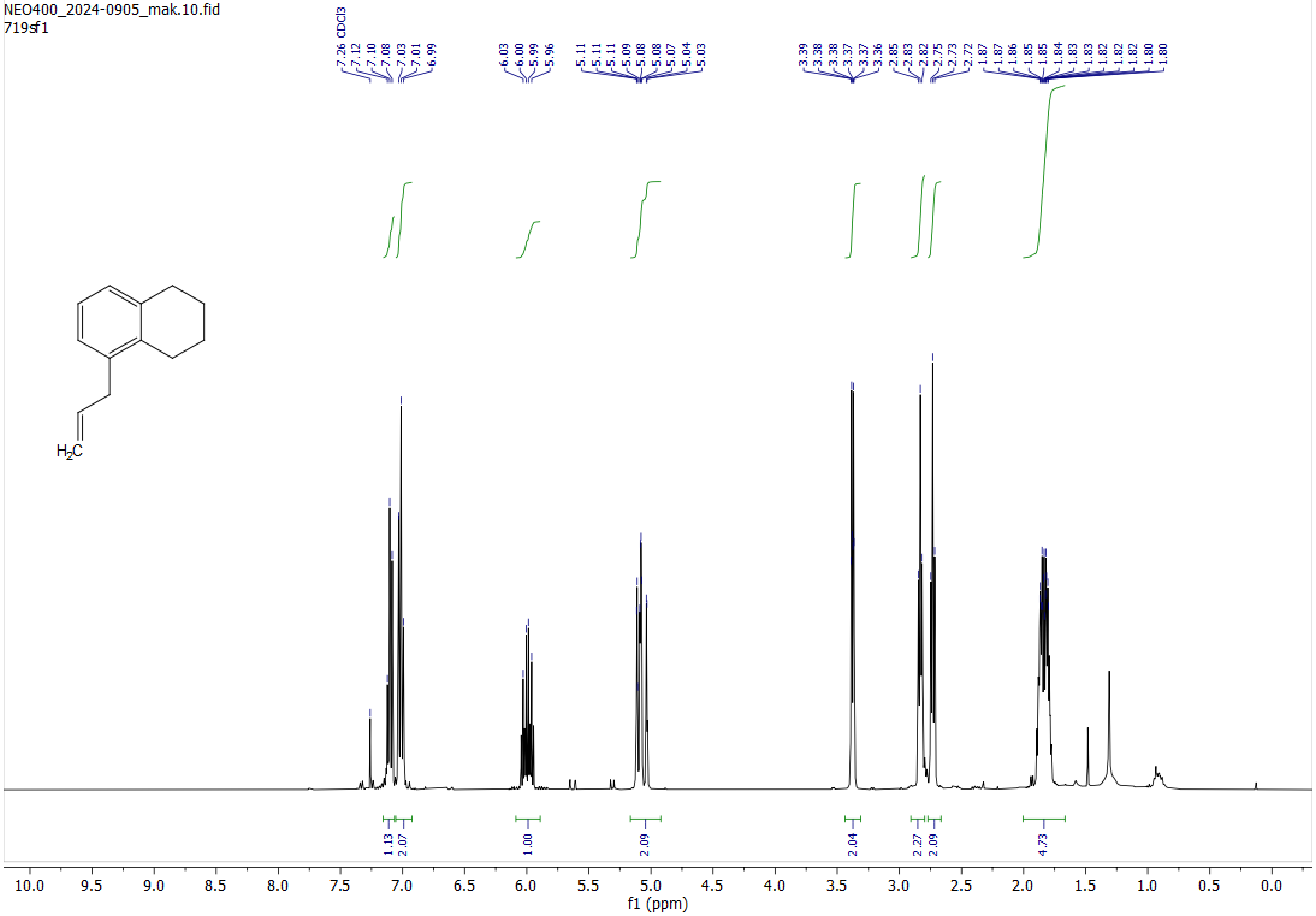

Figure S18: $^{13}C\{^{1}H\}$ NMR (100 MHz, $CDCl_3$) of **11d**

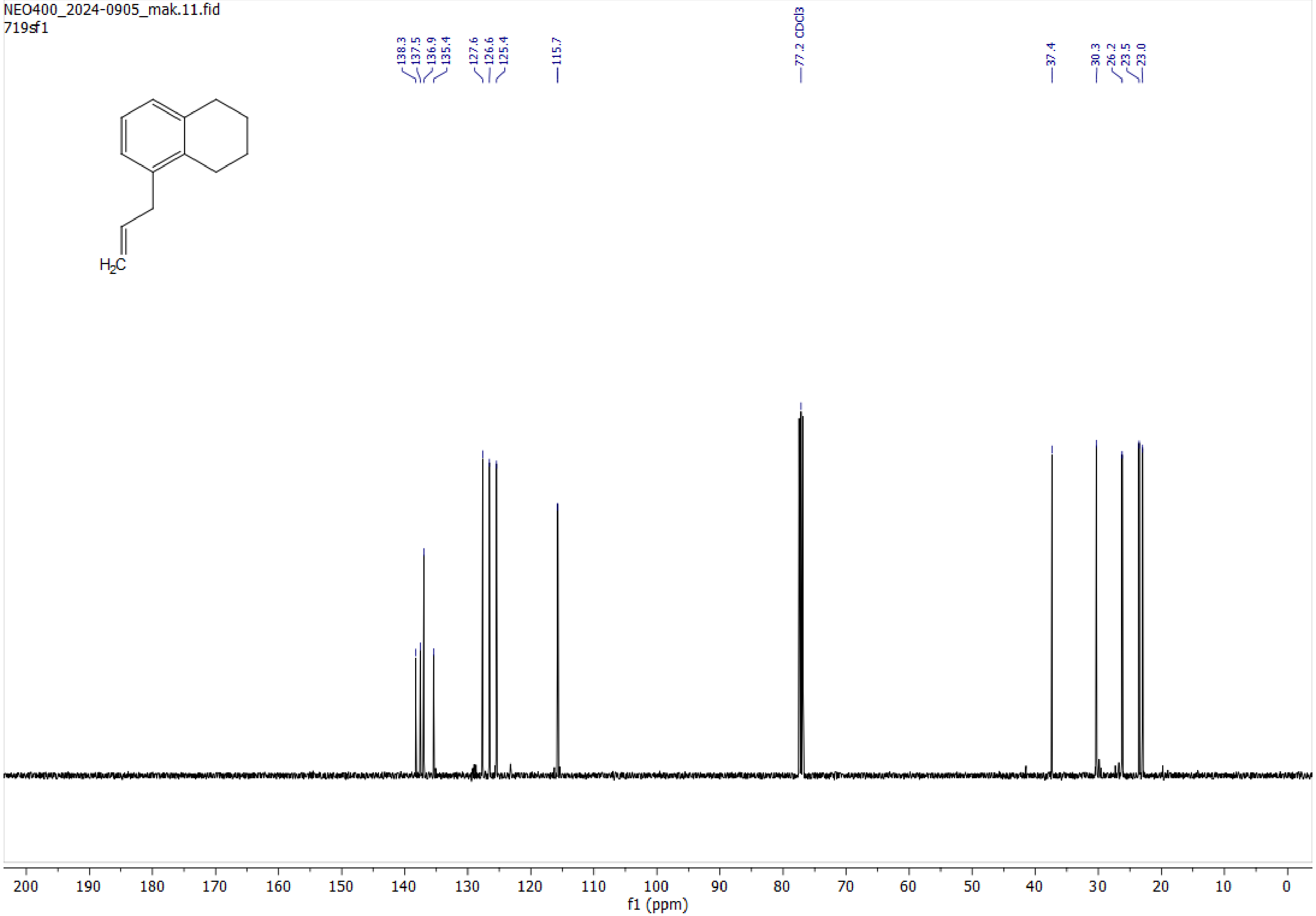

2 Synthesis of 7-methoxy-2-[4-(methoxymethoxy)phenyl]-8-(prop-2-en-1-yl)-2,3-dihydro-4*H*-chromen-4-one (15b)

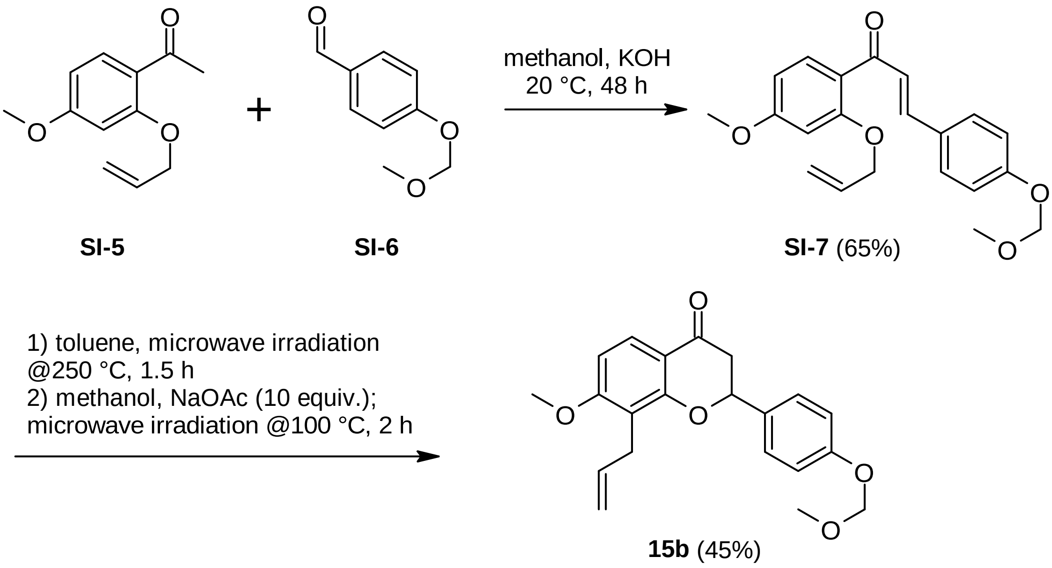

Scheme S2 Synthesis of allylflavanone **15b**

(2*E*)-3-[4-(Methoxymethoxy)phenyl]-1-[4-methoxy-2-(prop-2-en-1-yloxy)phenyl]prop-2-en-1-one (SI-7). A solution of KOH in methanol (60 wt %, 40 mL) was added dropwise to a well stirred solution of acetophenone **SI-5** (2.06 g, 10.0 mmol) and 4-OMOM-substituted benzaldehyde **SI-6** (1.66 g, 10.0 mmol) in methanol (20 mL) at 20 ºC. The reaction mixture was stirred at 20 ºC for 48 h. It was then poured into ice water (100 mL) and neutralized with aqueous HCl (4 M). The aqueous solution was extracted with EtOAc (three times 60 mL). The combined organic extracts were washed with brine, dried with anhydrous $MgSO_4$, and filtered. The solvent was evaporated under reduced pressure, and the residue was purified by column chromatography on silica using hexane − EtOAc mixture (5:1 (v/v)) as eluent to afford **SI-7** as a pale yellow solid; yield: 2.30 g (6.5 mmol, 65%); mp 81 – 83 °C; IR (ATR) ν 2903 (w), 1649 (m), 1584 (s), 1244 (m), 1149 (s) cm^{-1}; ^1H NMR (400 MHz, $CDCl_3$) δ 7.77 (d, *J* = 8.6 Hz, 1H), 7.66 (d, *J* = 15.7 Hz, 1H), 7.53 (d, *J* = 8.6 Hz, 2H), 7.50 (d, *J* = 15.7 Hz, 1H), 7.04 (d, *J* = 8.6 Hz, 2H), 6.57 (dd, *J* = 8.6, 2.3 Hz, 1H), 6.48 (d, *J* = 2.3 Hz, 1H), 6.06 (ddt, *J* = 17.2, 10.6, 5.1 Hz, 1H), 5.45 (dm, *J* = 17.2 Hz, 1H), 5.28 (dm, *J* = 10.6 Hz, 1H), 5.21 (s, 2H), 4.62 (dt, *J* = 5.1, 1.6 Hz, 2H), 3.86 (s, 3H), 3.49 (s, 3H); $^{13}C\{^1H\}$ NMR (100 MHz, $CDCl_3$) δ 190.7, 164.1, 159.4, 158.9, 141.7, 133.1, 132.6, 130.0, 129.4, 125.7, 122.8, 118.1, 116.5, 105.7, 99.9, 94.4, 69.5, 56.3, 55.7; HRMS (EI) *m/z* [M^+] calcd for $C_{21}H_{22}O_5$ 354.1467, found 354.1472.

7-Methoxy-2-[4-(methoxymethoxy)phenyl]-8-(prop-2-en-1-yl)-2,3-dihydro-4*H*-chromen-4-one (15b). A solution of the **SI-7** (710 mg, 2.0 mmol) in toluene (10 mL) was placed in a

vessel suited for microwave irradiation. The vessel was sealed and irradiated in a microwave reactor at 250 ºC for 1.5 h. The solvent was evaporated, and the residue was redissolved in methanol (20 mL) in a vessel suited for microwave irradiation. NaOAc (1.68 g, 20.00 mmol) was added to the solution, the vessel was sealed again, and the mixture was further irradiated in a microwave reactor at 100 ºC for 2 h. The solvent was then evaporated, and water (50 mL) was added to the residue. The mixture was extracted with EtOAc (three times 30 mL). The combined organic extracts were washed with brine, dried with anhydrous $MgSO_4$, and filtered. The solvent was evaporated under reduced pressure and the residue was purified by column chromatography on silica using hexanes − MTBE mixture (4:1 (v/v)) to afford the **15b** as a pale yellow solid; yield: 320 mg (0.90 mmol, 45%); mp 87 – 89 °C; IR (ATR) ν 2901 (w), 2884 (w), 1667 (s), 1601 (s), 1234 (m), 1022 (s) cm^{-1}; ^{1}H NMR (400 MHz, $CDCl_3$) δ 7.85 (d, *J* = 8.8 Hz, 1H), 7.39 (d, *J* = 8.7 Hz, 2H), 7.08 (d, *J* = 8.7 Hz, 2H), 6.63 (d, *J* = 8.8 Hz, 1H), 5.91 (ddt, *J* = 17.1, 10.0, 6.3 Hz, 1H), 5.39 (dd, *J* = 12.8, 3.0 Hz, 1H), 5.18 (s, 2H), 4.98 (dm, *J* = 17.1 Hz, 1H), 4.94 (dm, *J* = 10.0 Hz, 1H), 3.87 (s, 3H), 3.47 (s, 3H), 3.41 (d, *J* = 6.3, Hz, 2H), 2.97 (dd, *J* = 16.8, 12.8 Hz, 1H), 2.82 (dd, *J* = 16.8, 3.0 Hz, 1H); ^{13}C{^{1}H} NMR (100 MHz, $CDCl_3$) δ 191.5, 163.3, 160.3, 157.3, 135.8, 132.6, 127.3, 126.7, 116.3, 115.9, 115.3, 114.7, 104.9, 94.3, 79.0, 56.0, 55.9, 44.2, 27.1; HRMS (EI) *m*/*z* [M^+] calcd for $C_{21}H_{22}O_5$ 354.1467, found 354.1481.

Figure S19: ^{1}H NMR (400 MHz, $CDCl_3$) of **SI-7**

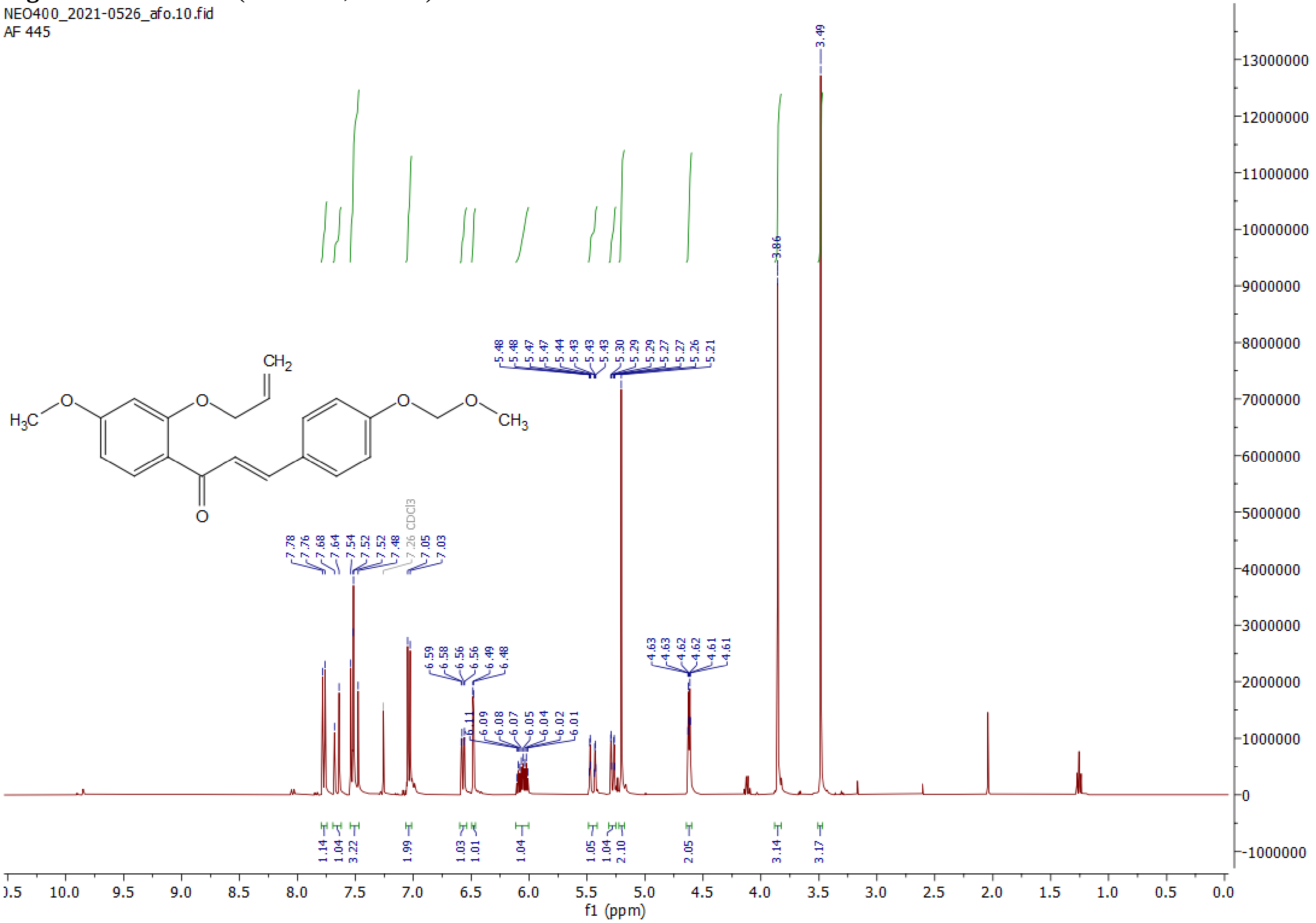

Figure S20: $^{13}C\{^{1}H\}$ NMR (100 MHz, $CDCl_3$) of **SI-7**

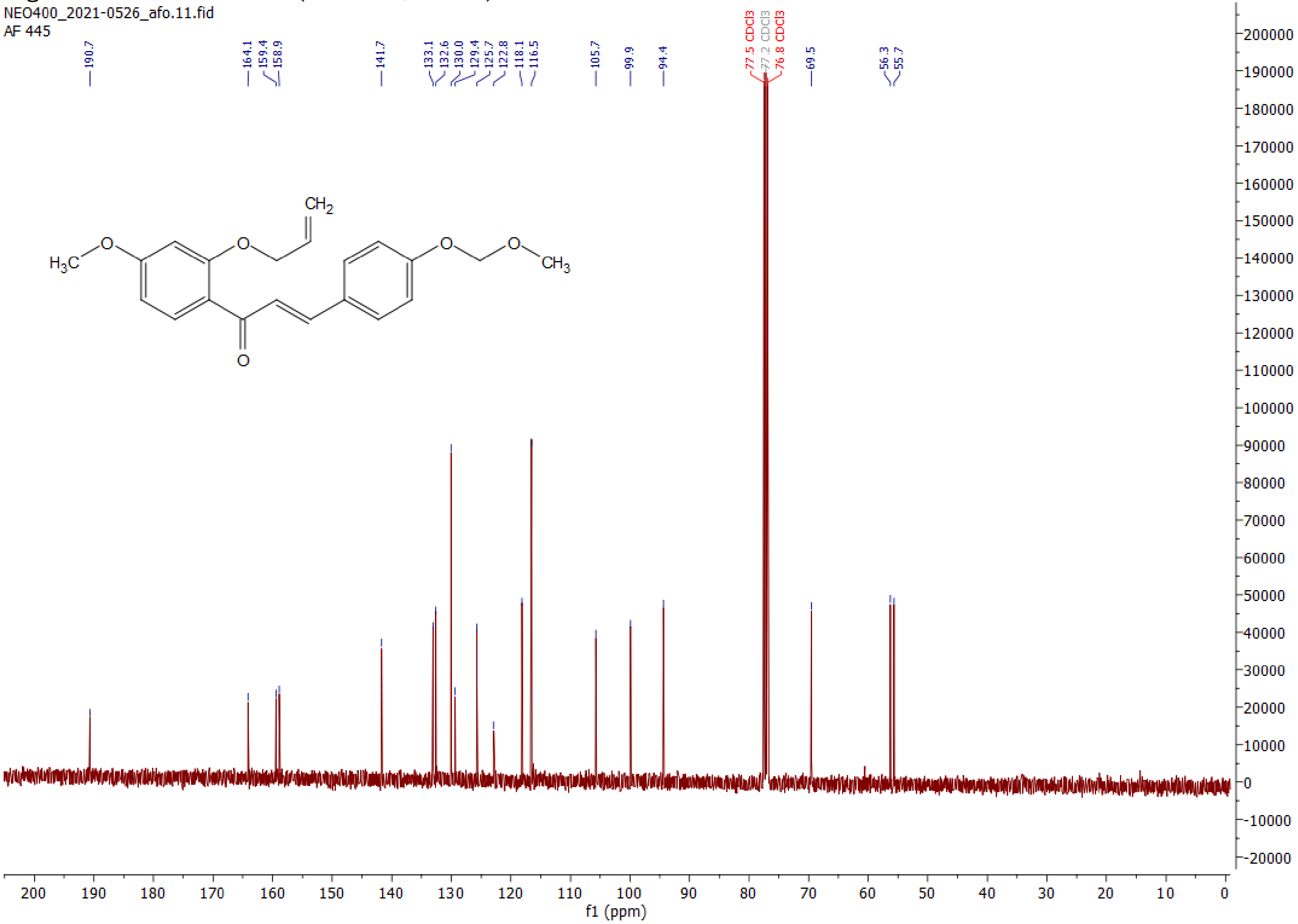

Figure S21: ^{1}H NMR (400 MHz, $CDCl_3$) of **15b**

NEO400_2021-0603_afo.40.fid
AF 447 F2

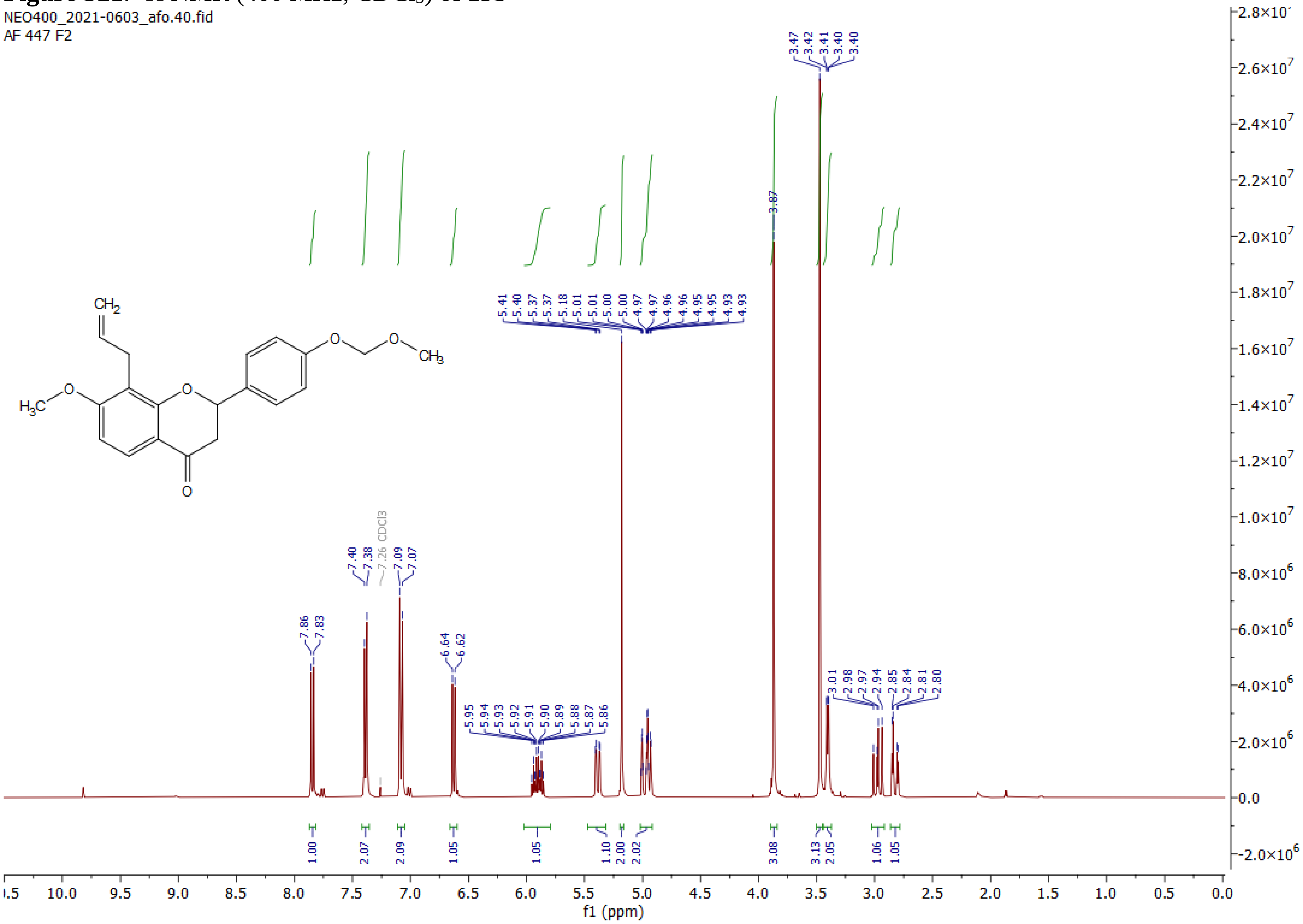

Figure S22: $^{13}C\{^1H\}$ NMR (100 MHz, $CDCl_3$) of **15b**

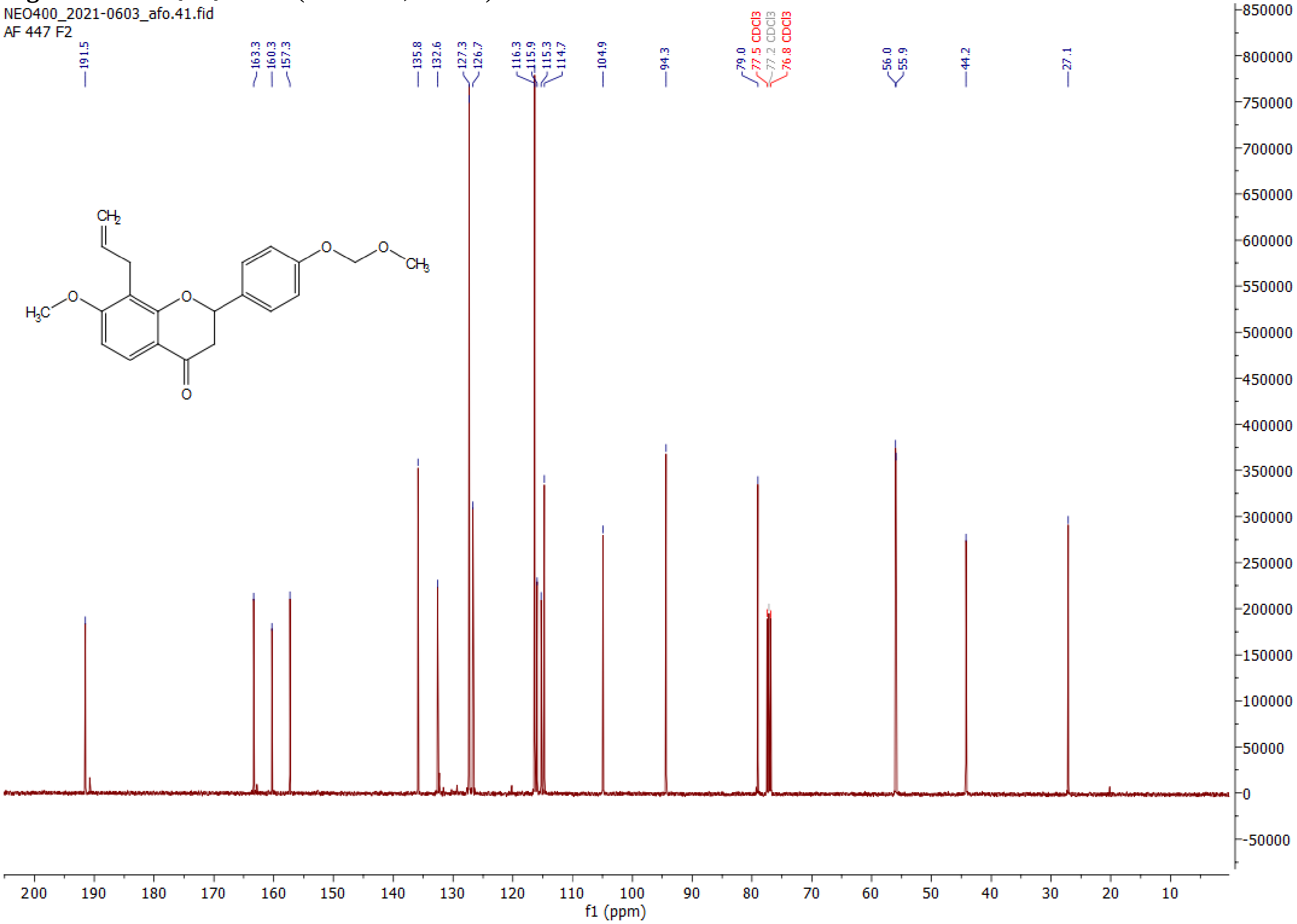

C Copies of NMR-spectra

compound	page	compound	page	compound	page
**12aa**	S26	**12ar**	S75	**13aq**	S135
**12ab**	S28	**12at**	S77	**14ar**	S141
**12ac**	S30	**12au**	S83	**13at**	S148
**12ad**	S32	**13aa**	S85	**14au**	S155
**12ae**	S34	**13ab**	S91	**16af**	S162
**12af**	S36	**13ac**	S93	**16bf**	S167
**12ag**	S43	**13ad**	S95	**16cf**	S171
**12ah**	S45	**13ae**	S97	**17af**	S175
**12ai**	S51	**14ae**	S103	**17bf**	S180
**12ak**	S57	**13af**	S109	**17cf**	S184
**12al**	S59	**13ai**	S116	**18af**	S188
**12am**	S61	**13ak**	S118	**18bf**	S190
**12an**	S63	**13al**	S120	**18cf**	S192
**12ao**	S65	**14am**	S126	**20**	S194
**12ap**	S67	**13an**	S131	**21**	S199
**12aq**	S73	**13ap**	S133	**23**	S204

Figure S23: ^{1}H NMR (400 MHz, $CDCl_3$) of **12aa**

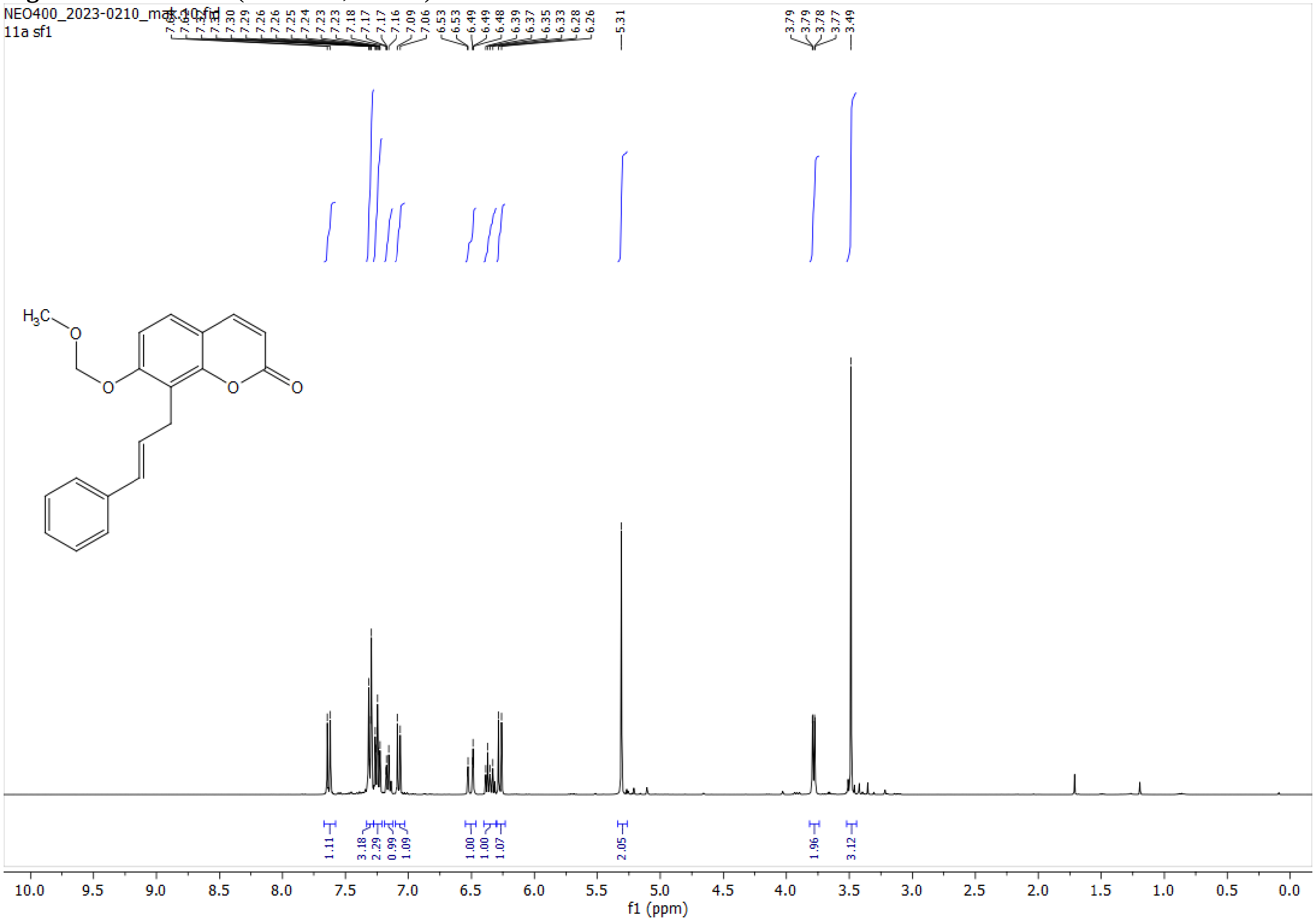

Figure S24: $^{13}C\{^{1}H\}$ NMR (100 MHz, $CDCl_3$) of **12aa**

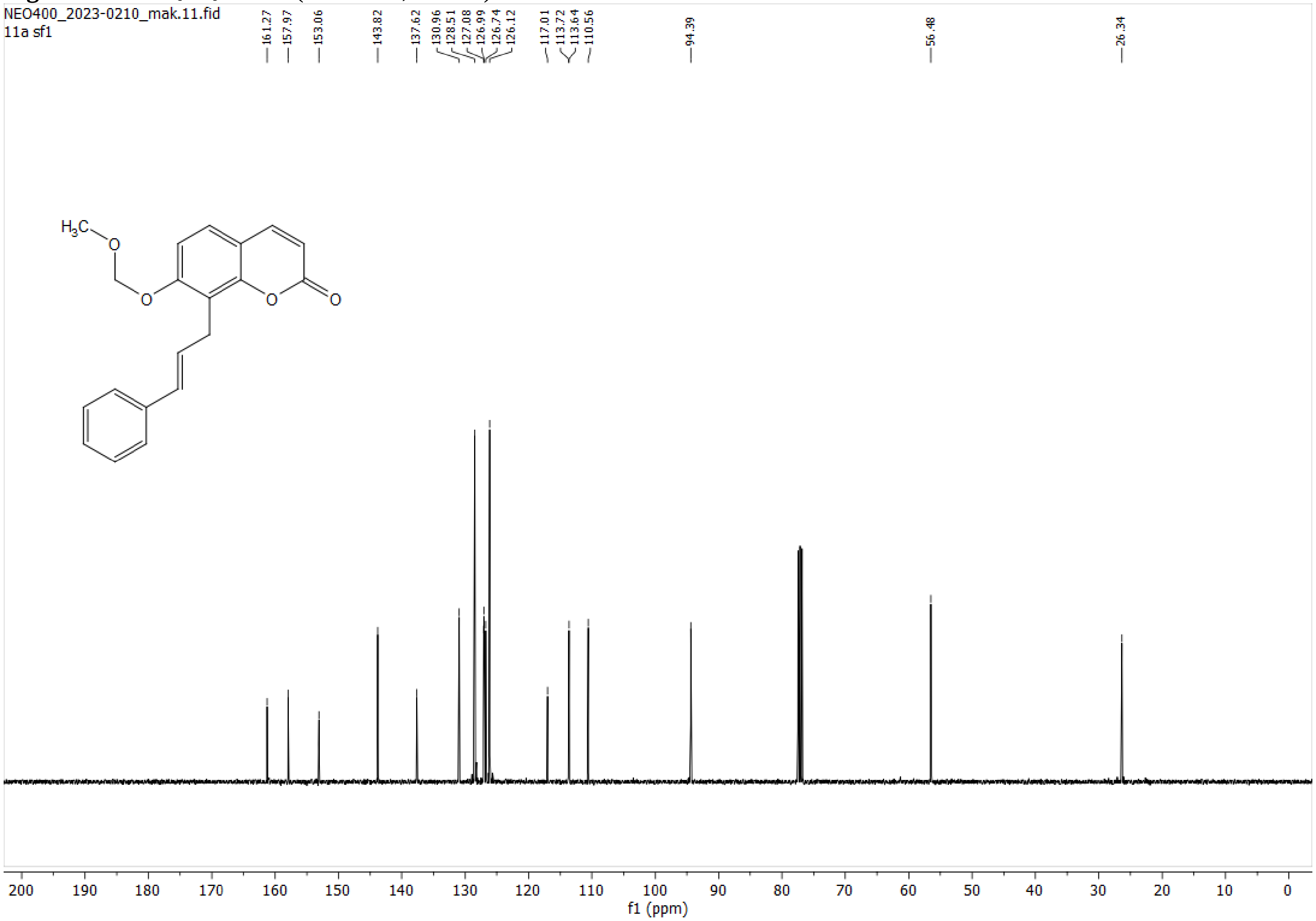

Figure S25: ^{1}H NMR (400 MHz, $CDCl_3$) of **12ab**

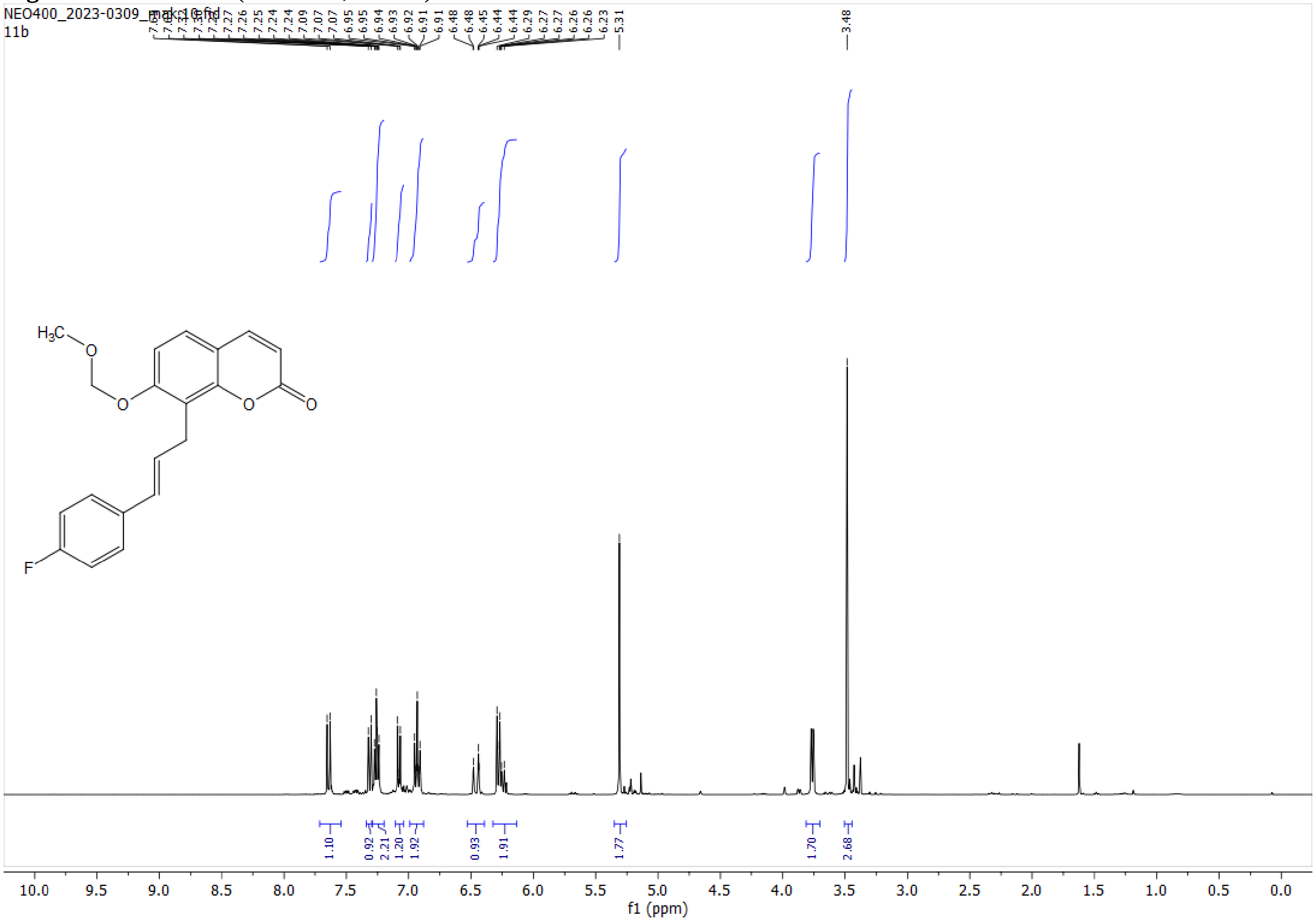

Figure S26: $^{13}C\{^{1}H\}$ NMR (100 MHz, $CDCl_3$) of **12ab**

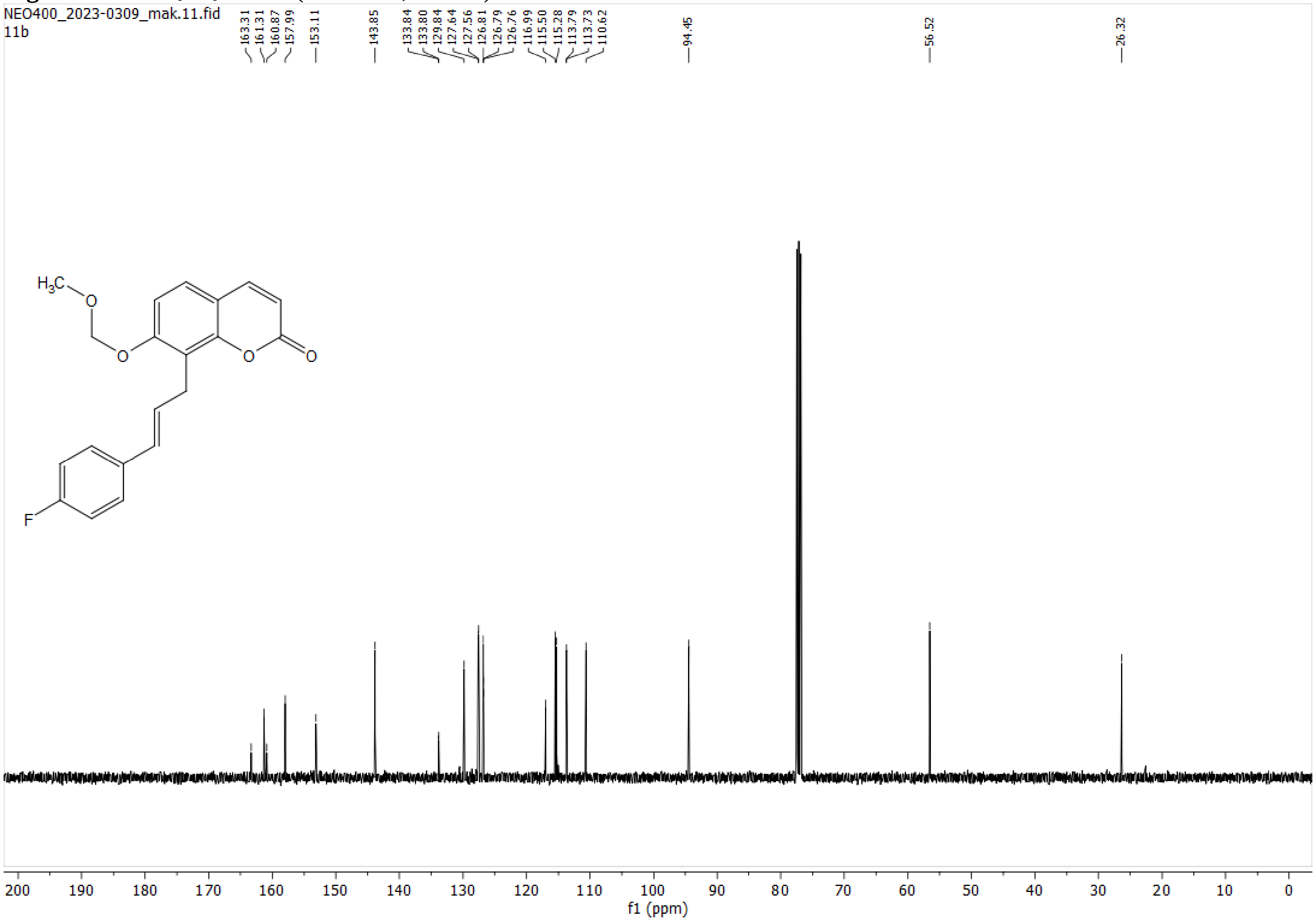

Figure S27: ^{1}H NMR (400 MHz, $CDCl_3$) of **12ac**

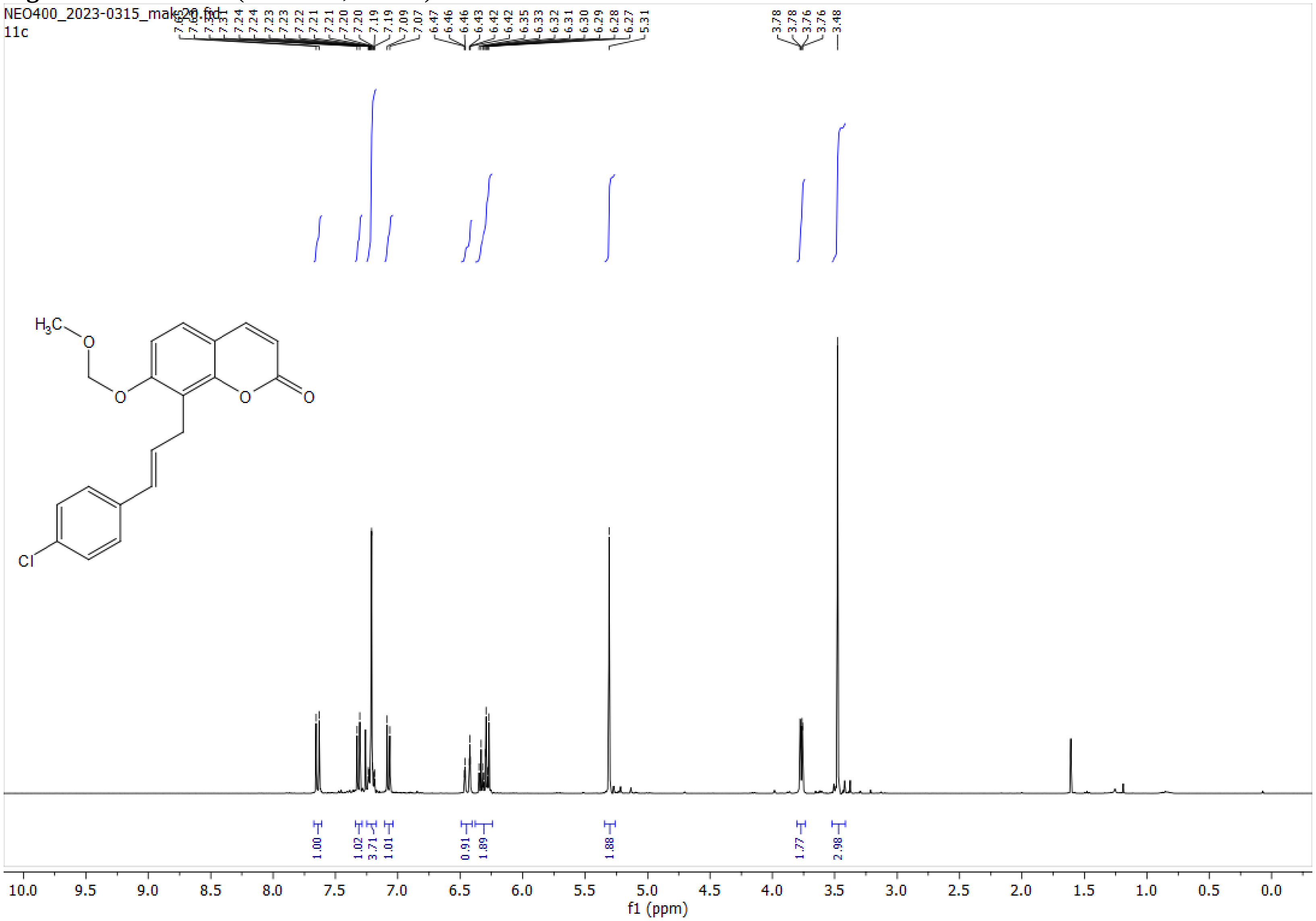

Figure S28: $^{13}C\{^{1}H\}$ NMR (100 MHz, $CDCl_3$) of **12ac**

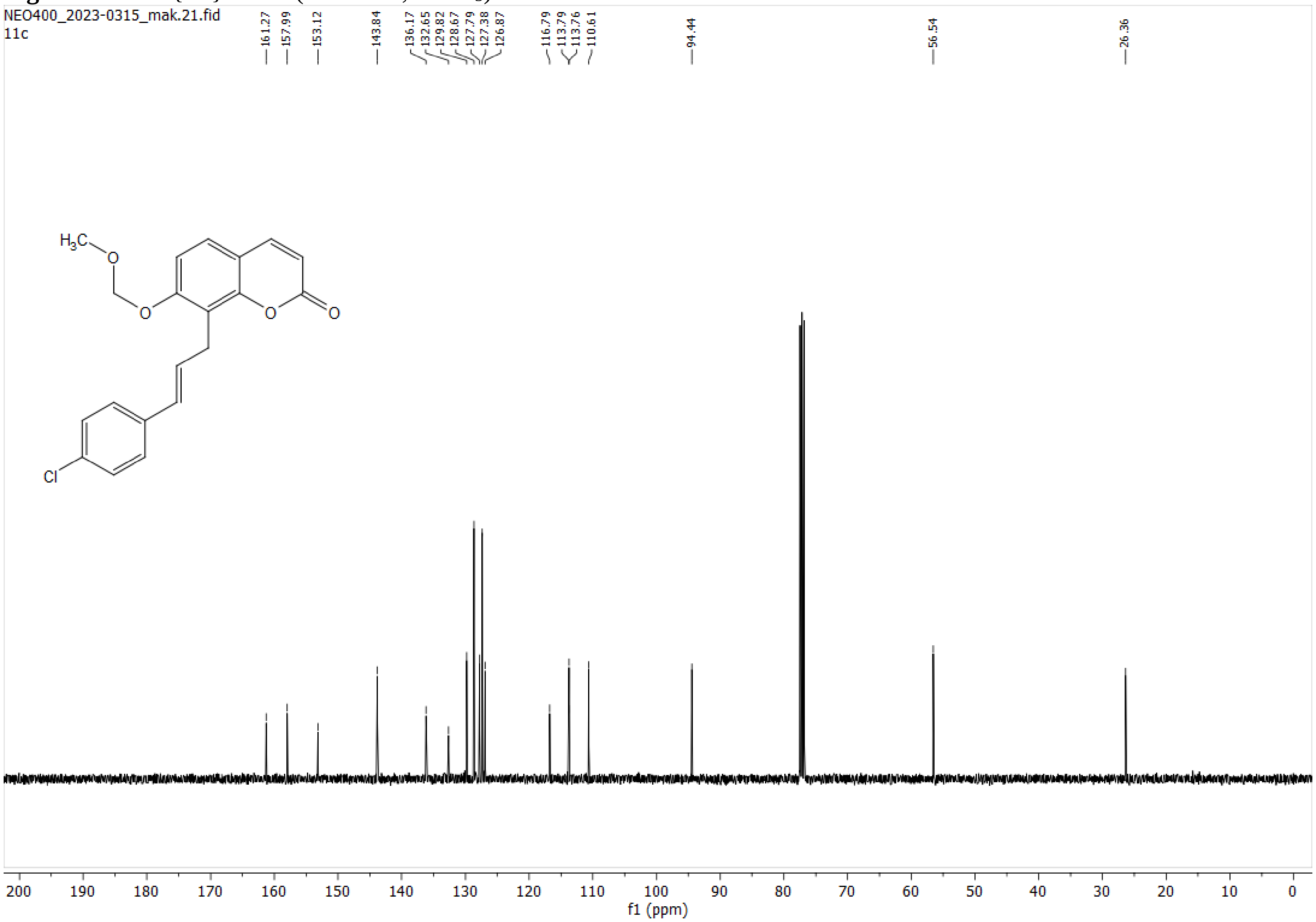

Figure S29: ^{1}H NMR (400 MHz, $CDCl_3$) of **12ad**

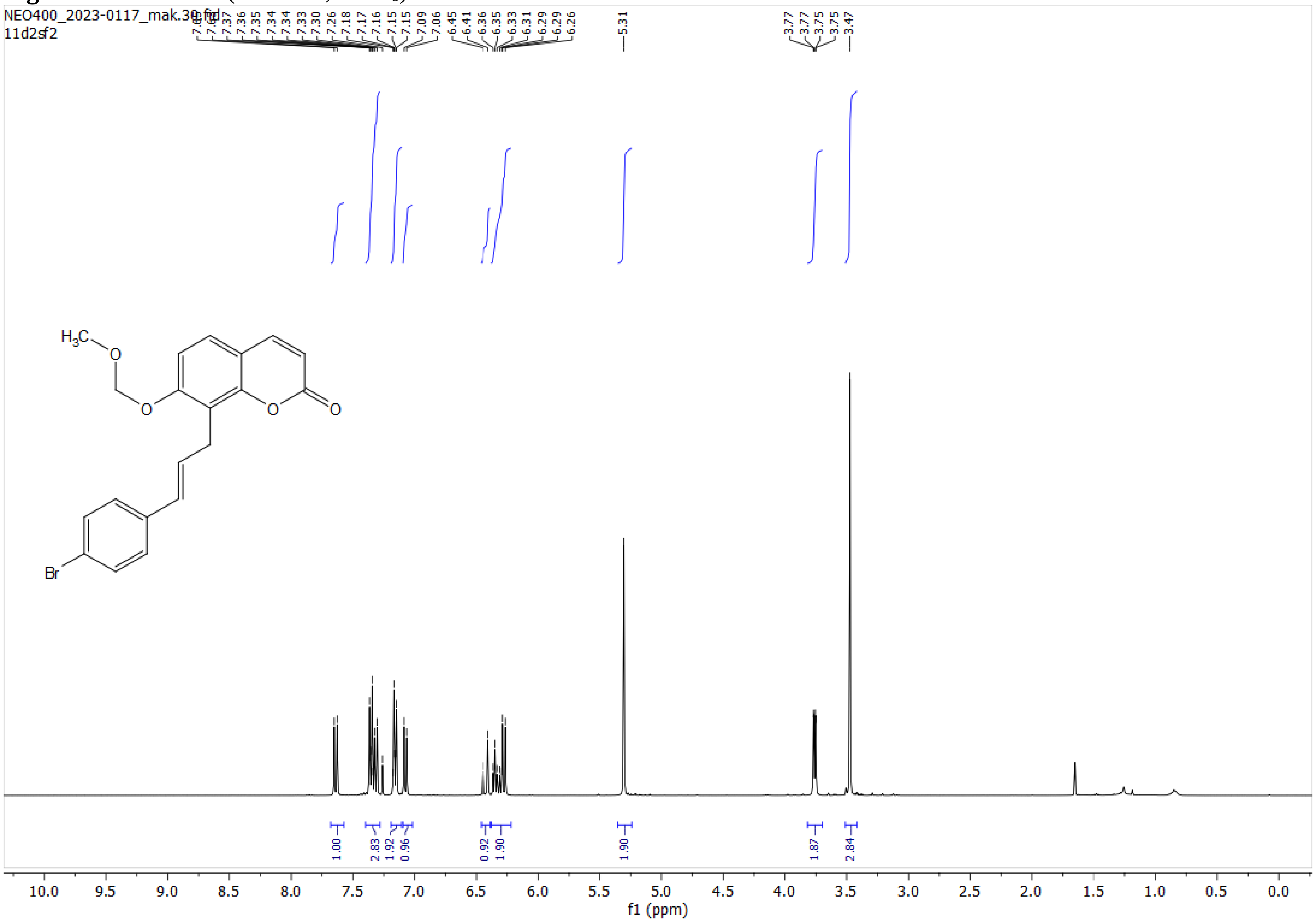

Figure S30: $^{13}C\{^{1}H\}$ NMR (100 MHz, $CDCl_3$) of **12ad**

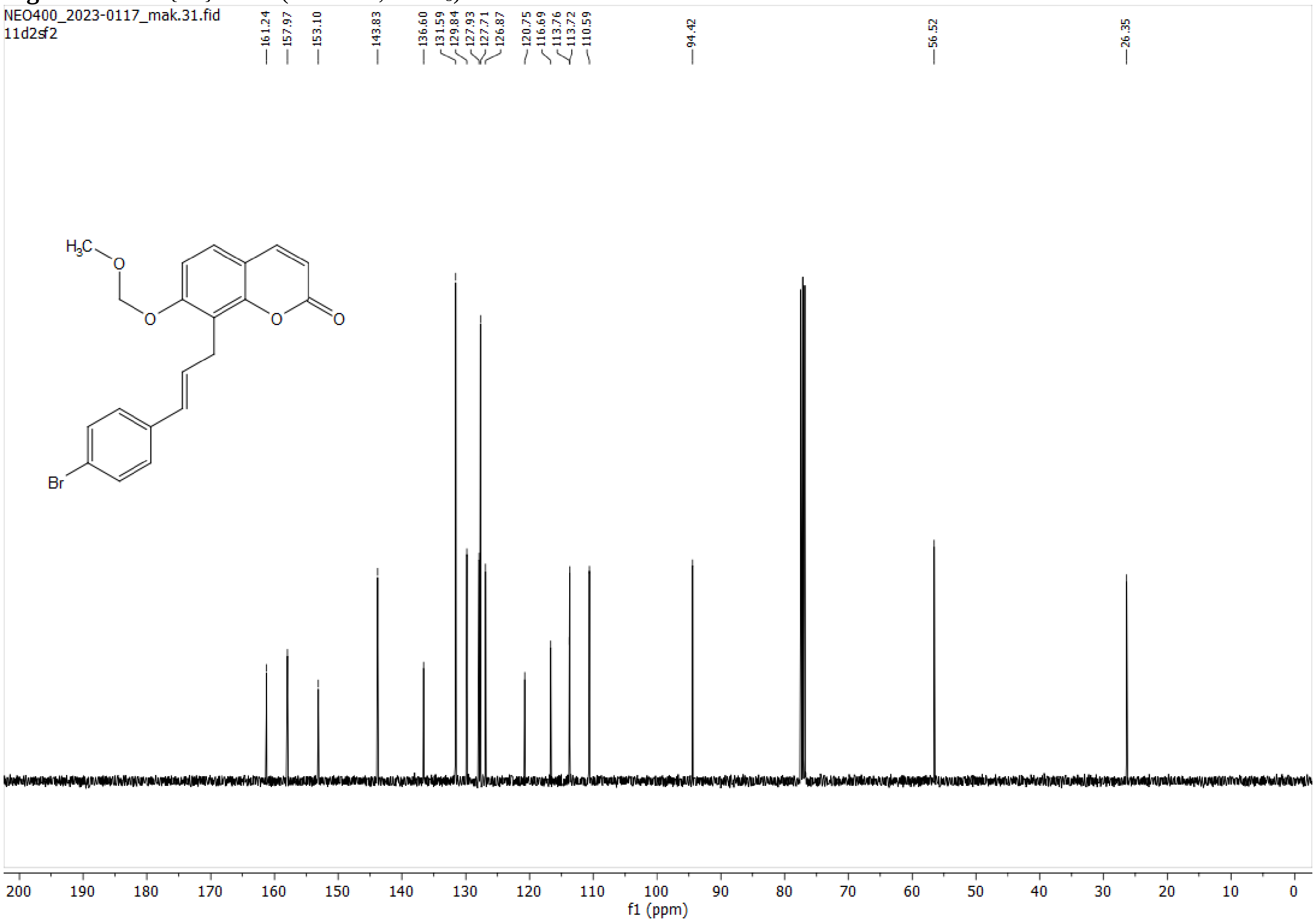

Figure S31: ^{1}H NMR (400 MHz, $CDCl_3$) of **12ae**

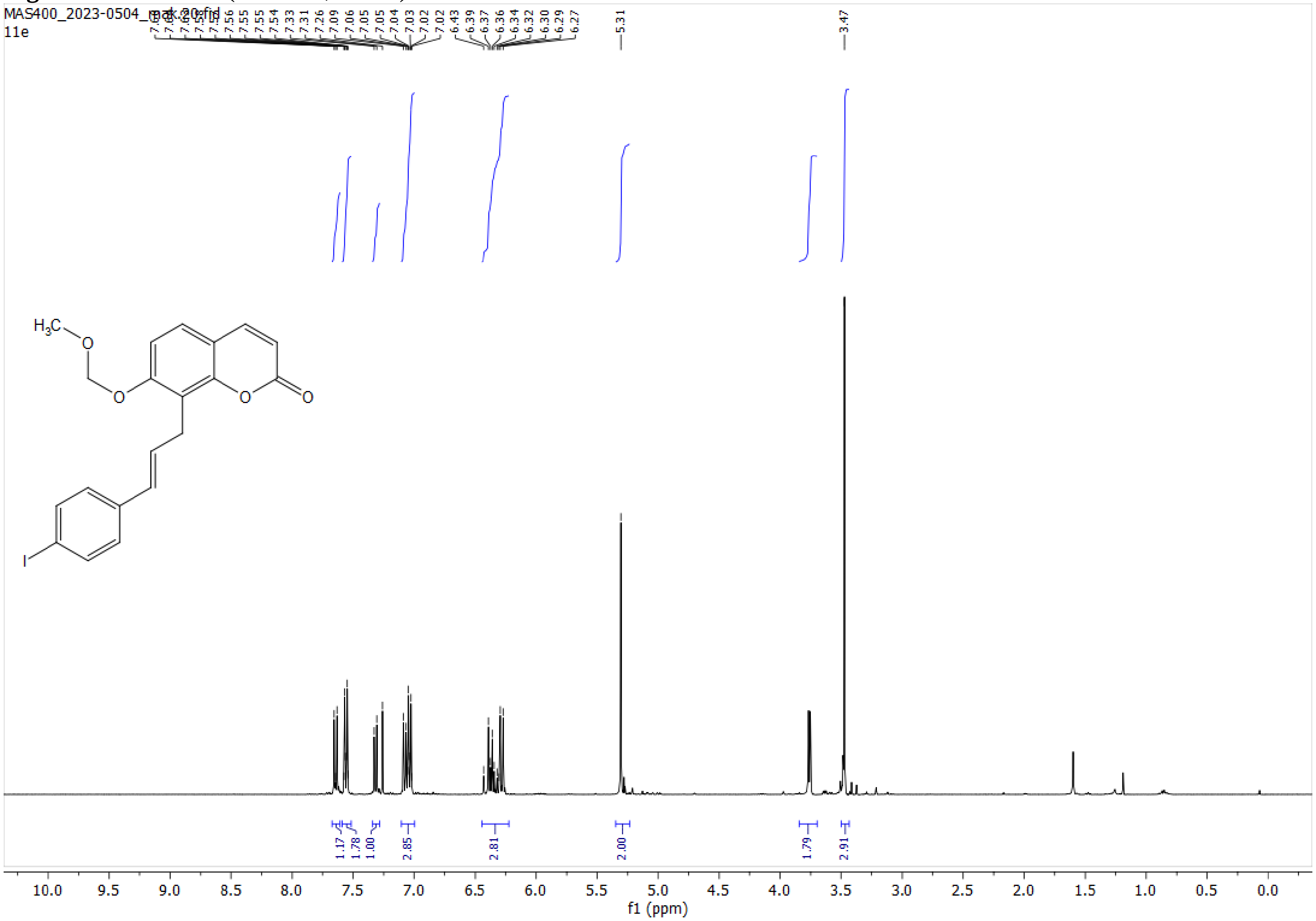

Figure S32: $^{13}C\{^{1}H\}$ NMR (100 MHz, $CDCl_3$) of **12ae**

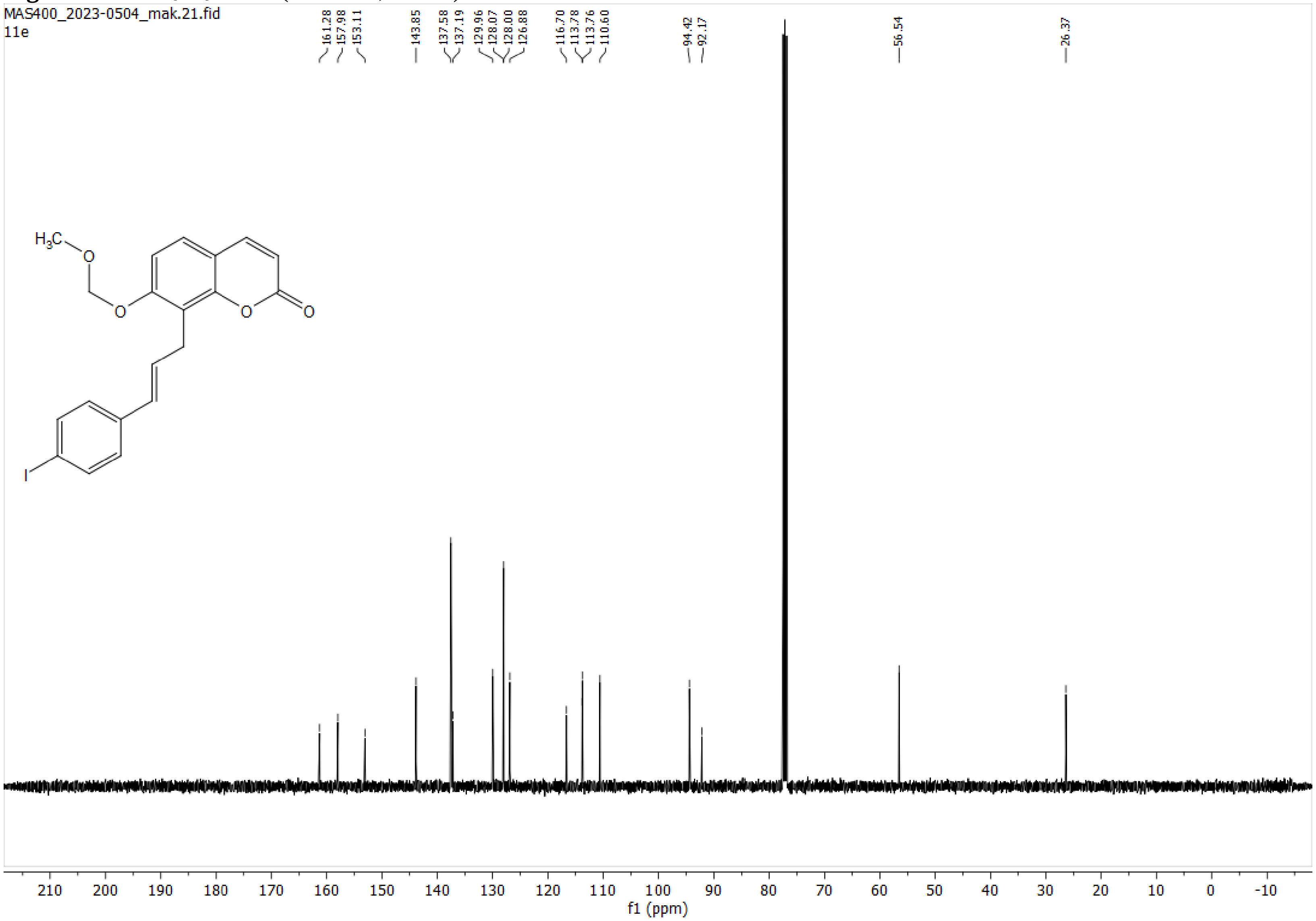

Figure S33: NMR-Signal assignment and selected HMBC-couplings for compound **12af**

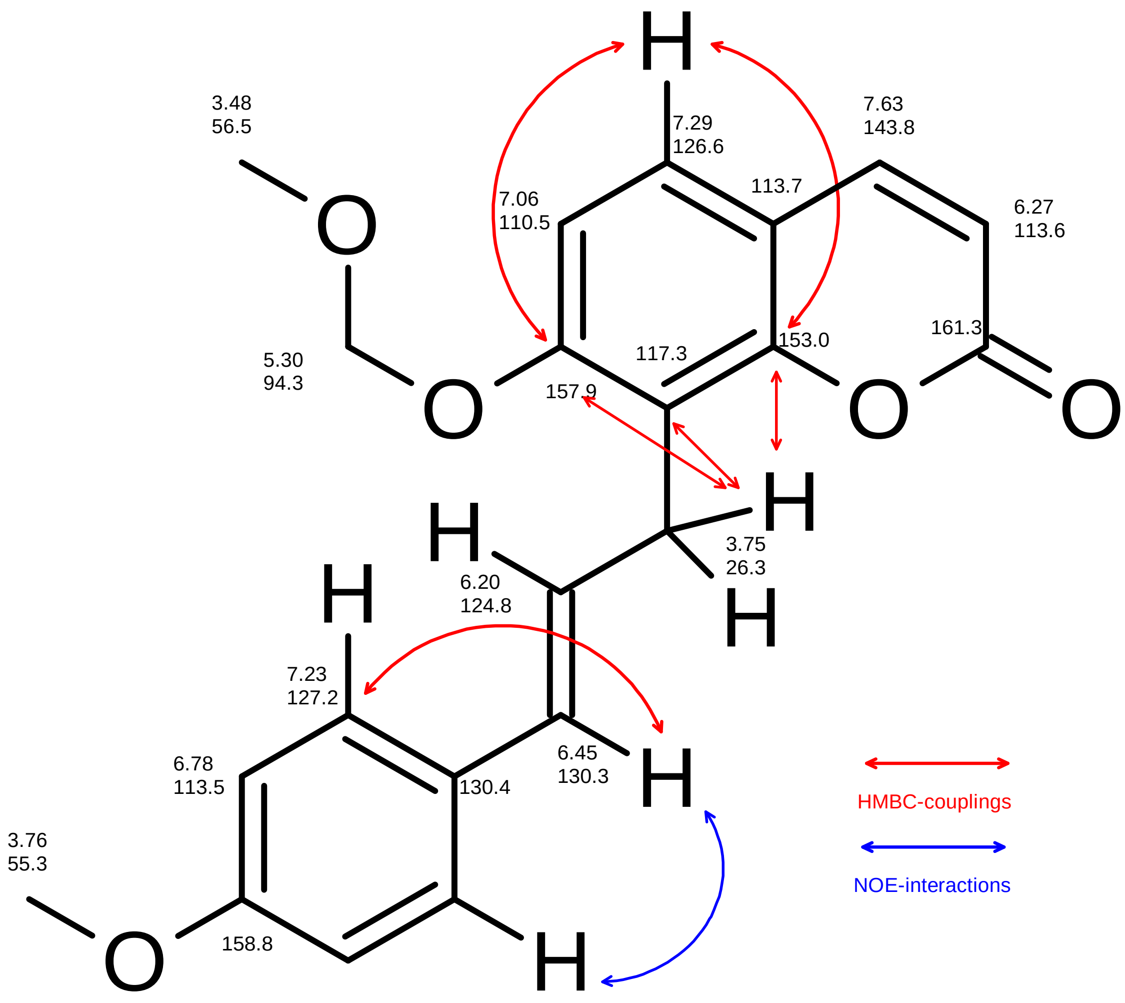

Figure S34: ^{1}H NMR (400 MHz, $CDCl_3$) of **12af**

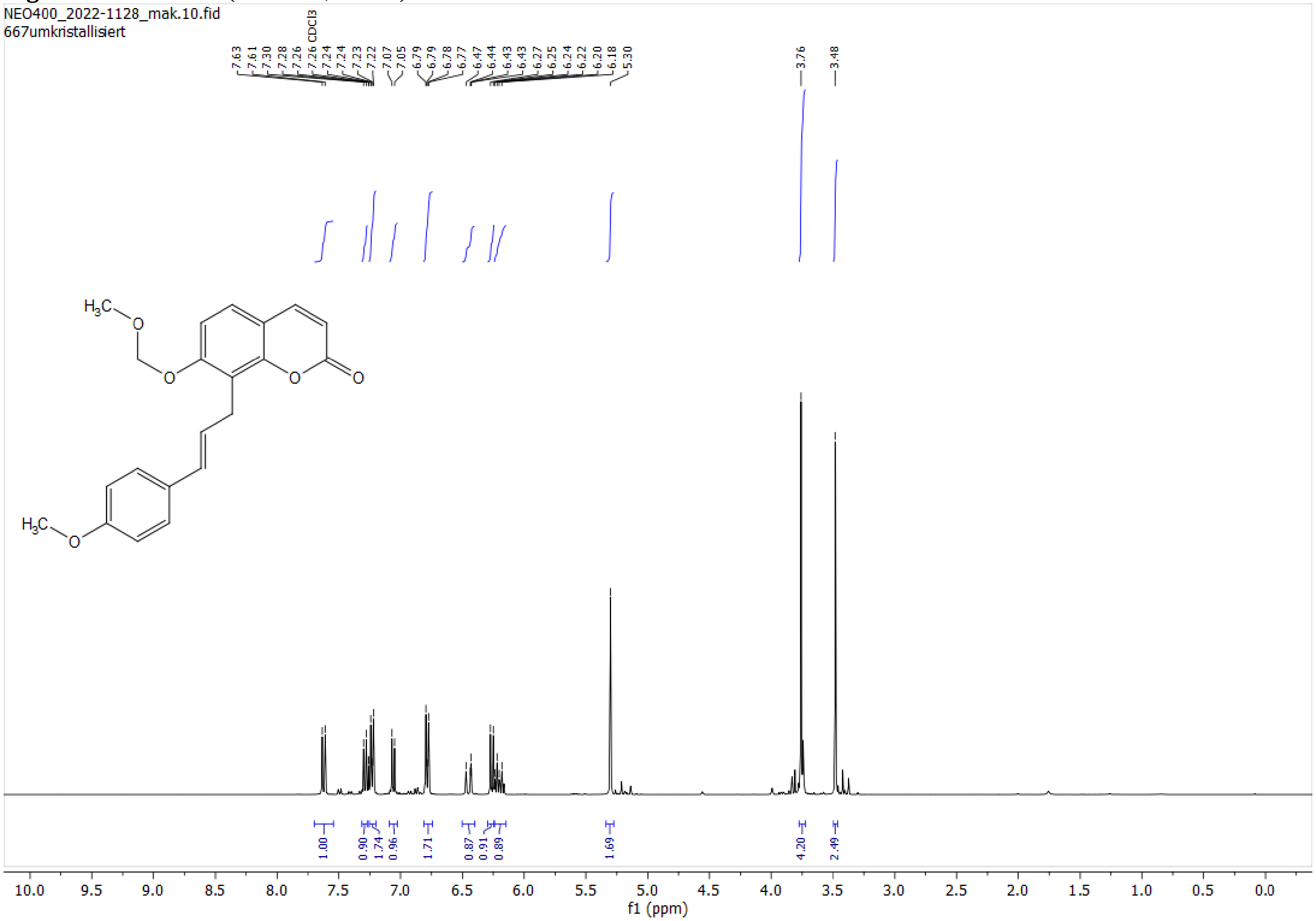

Figure S35: $^{13}C\{^{1}H\}$ NMR (100 MHz, $CDCl_3$) of **12af**

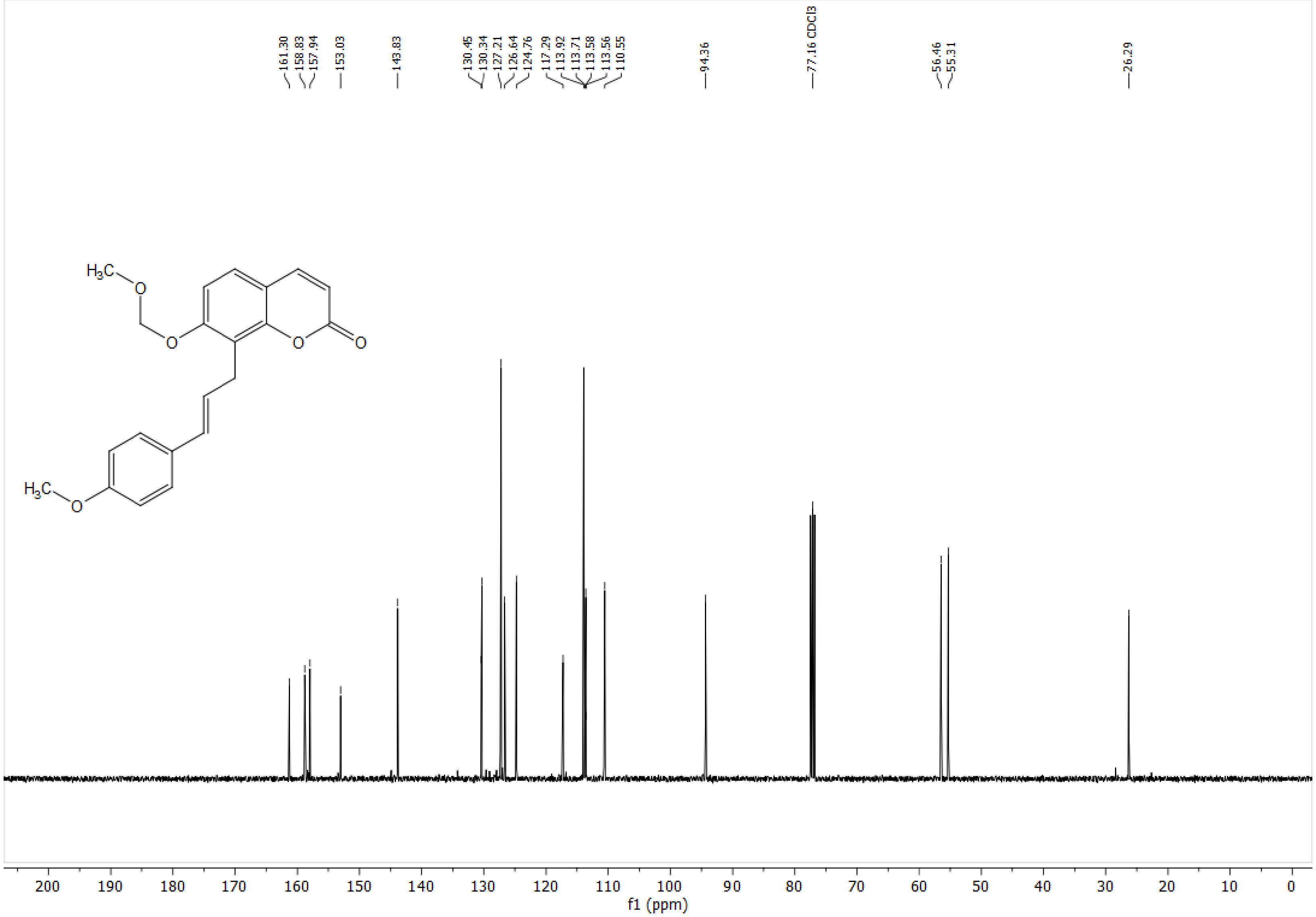

Figure S36: COSY (400 MHz, $CDCl_3$) of **12af**

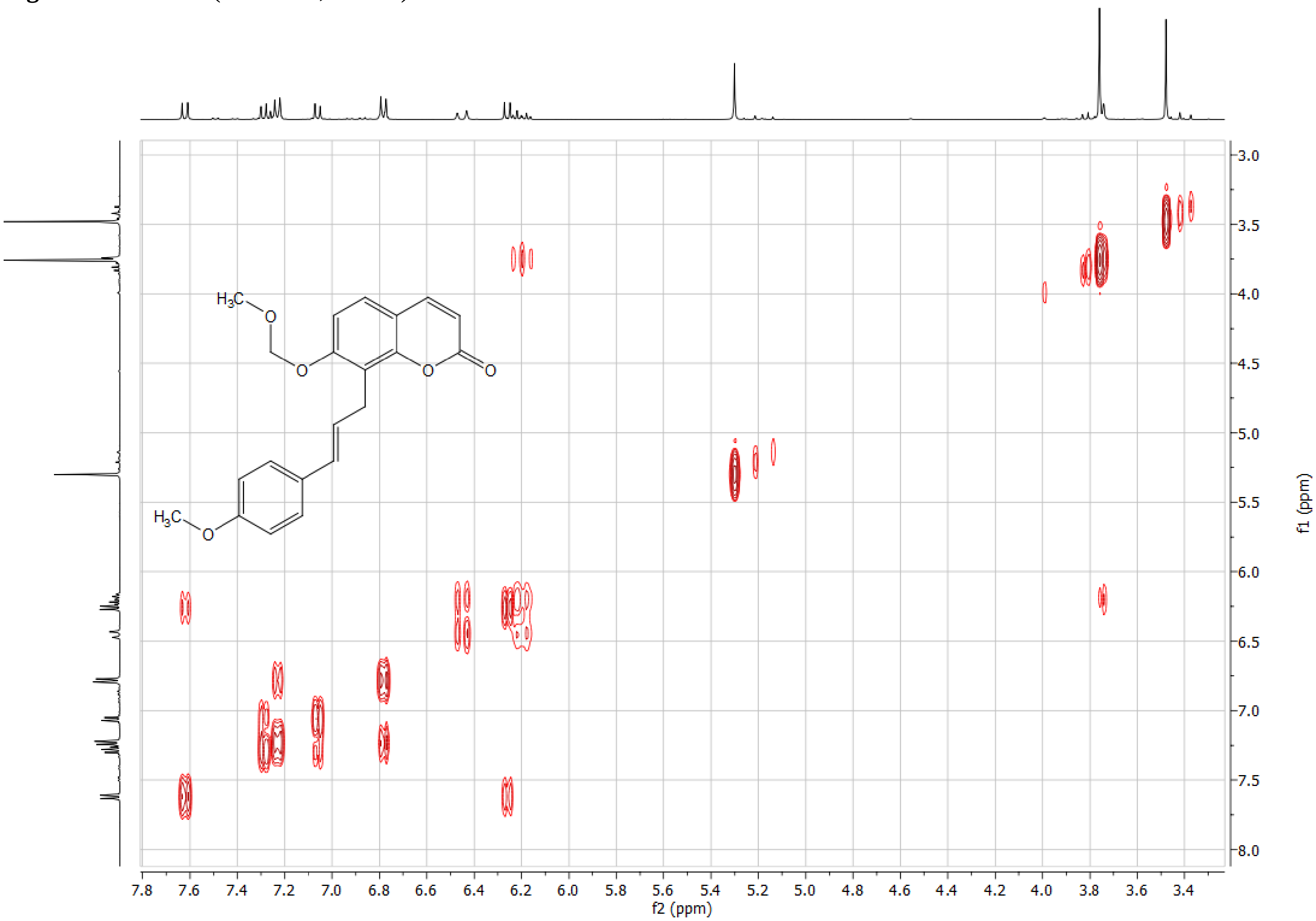

Figure S37: HSQC (400/100 MHz, $CDCl_3$) of **12af**

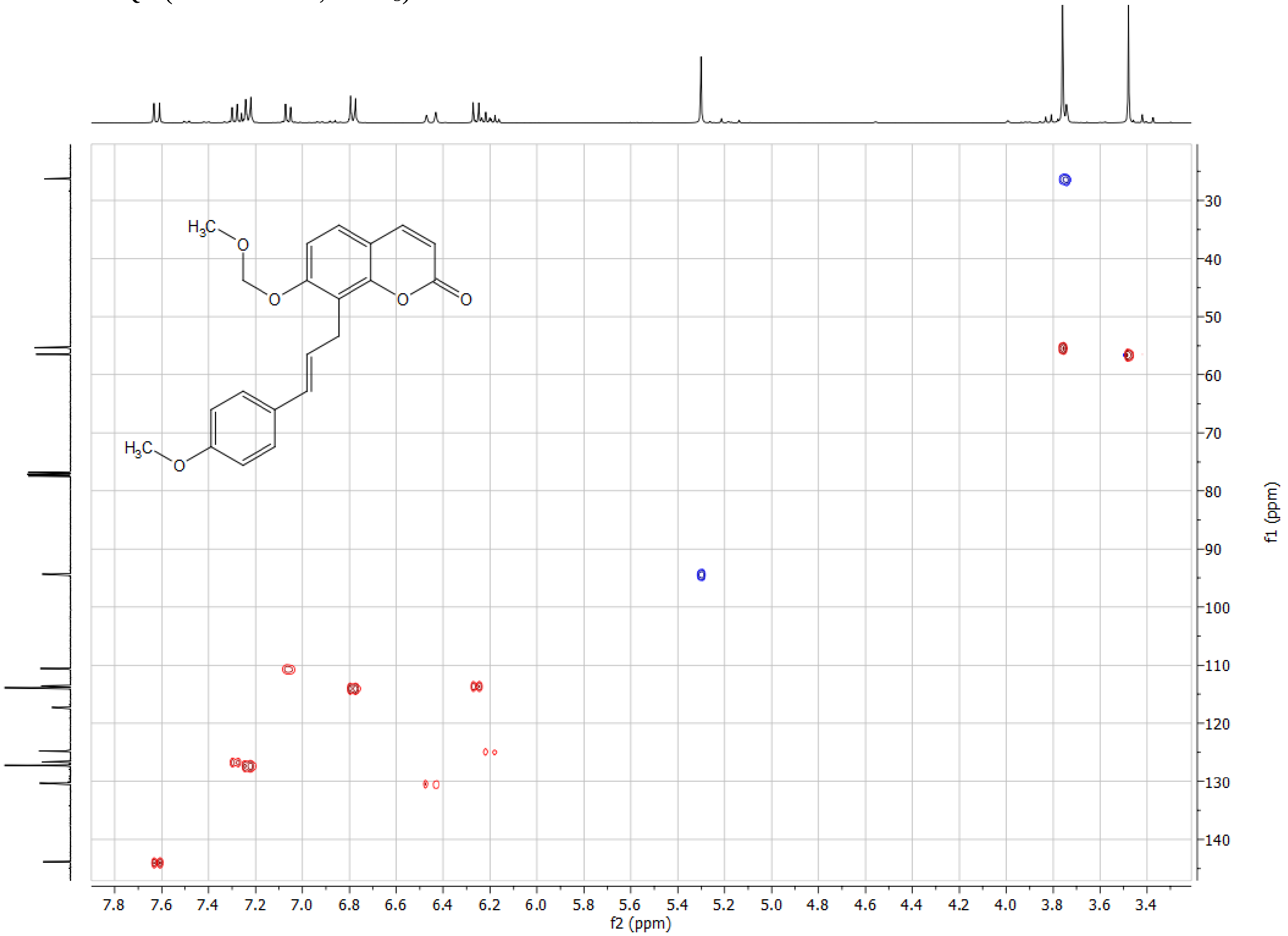

Figure S38: HMBC (400/100 MHz, $CDCl_3$) of **12af**

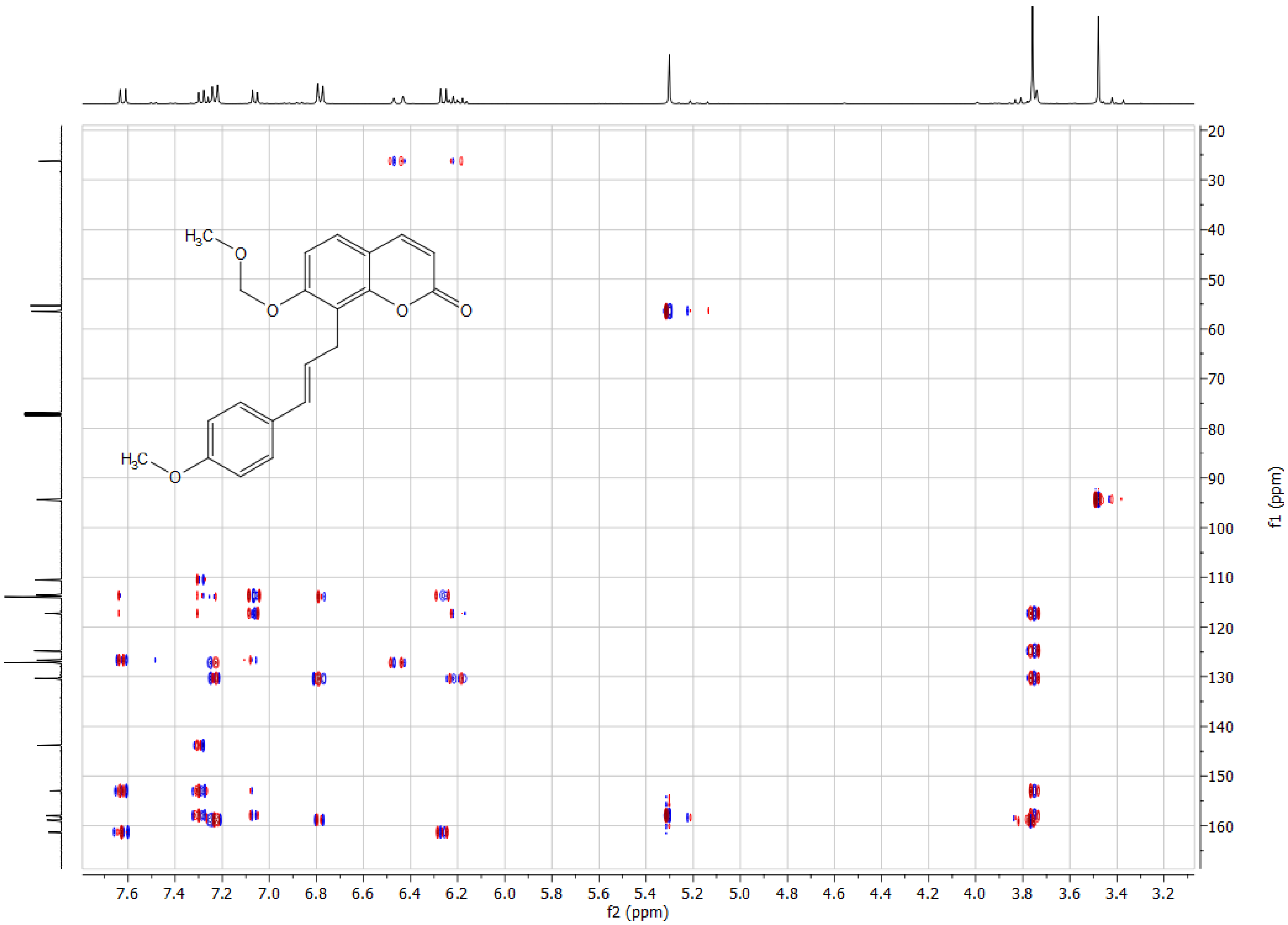

Figure S39: NOESY (400 MHz, $CDCl_3$) of **12af**

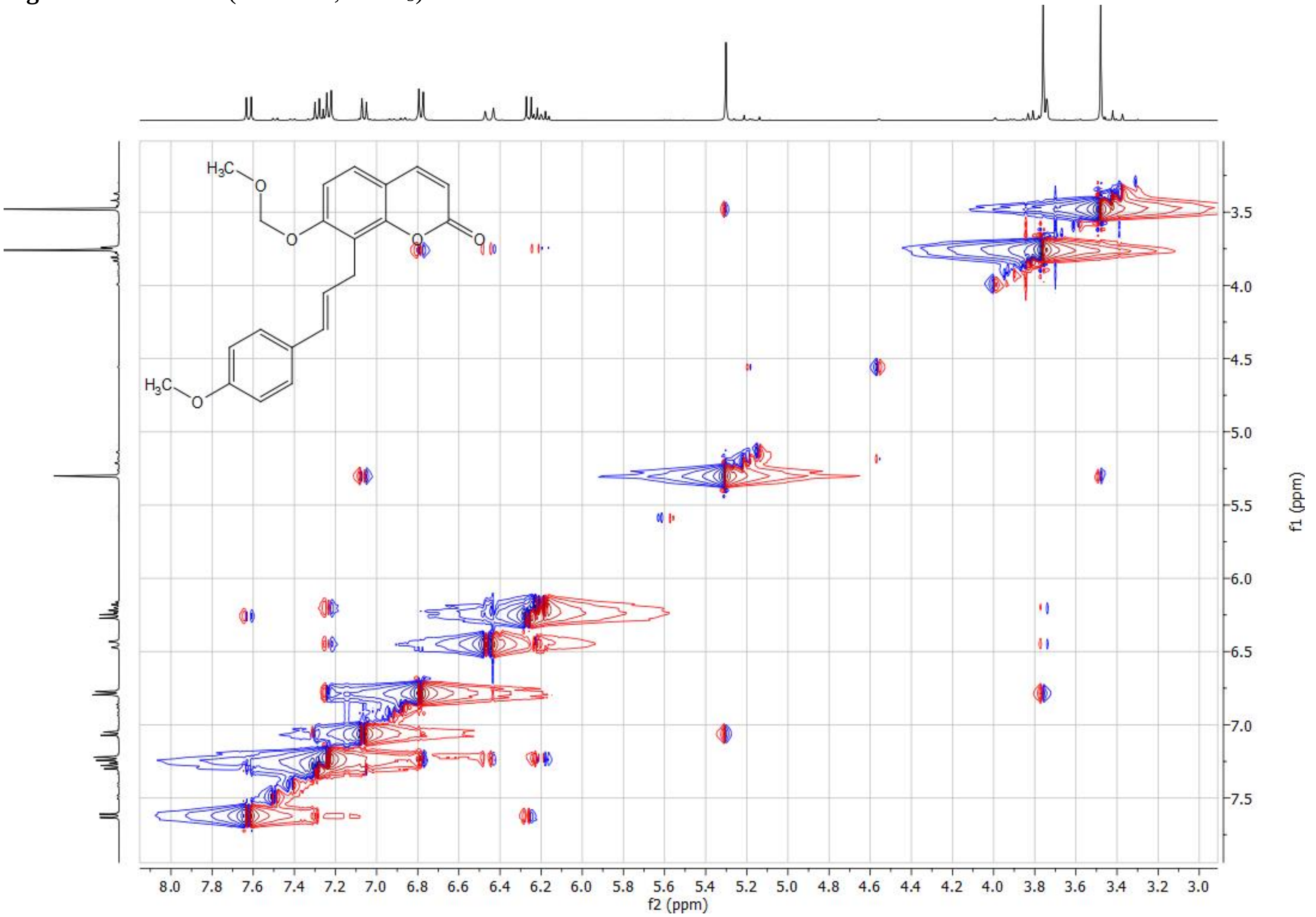

Figure S40: ^{1}H NMR (400 MHz, $CDCl_3$) of **12ag**

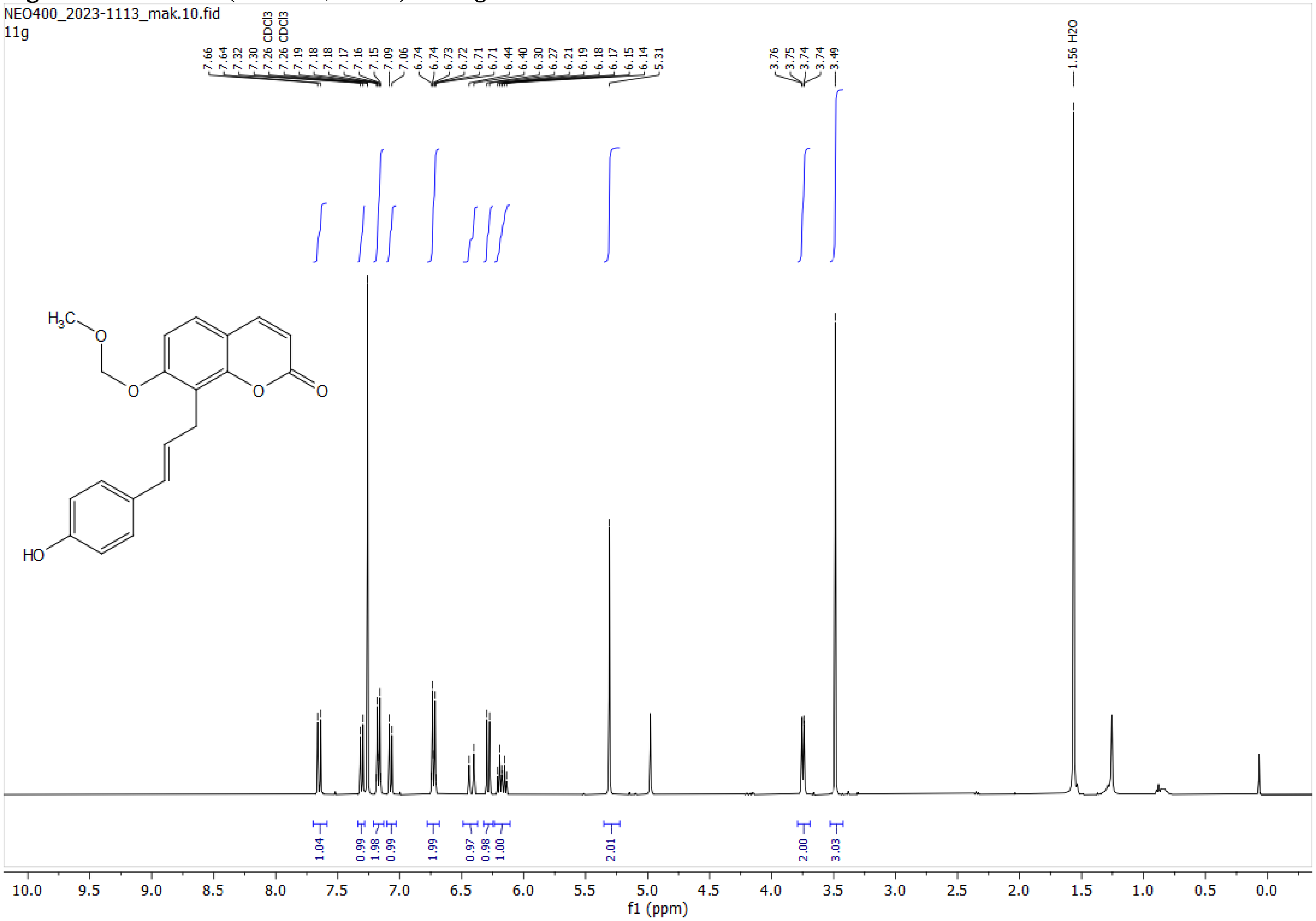

Figure S41: $^{13}C\{^{1}H\}$ NMR (100 MHz, $CDCl_3$) of **12ag**

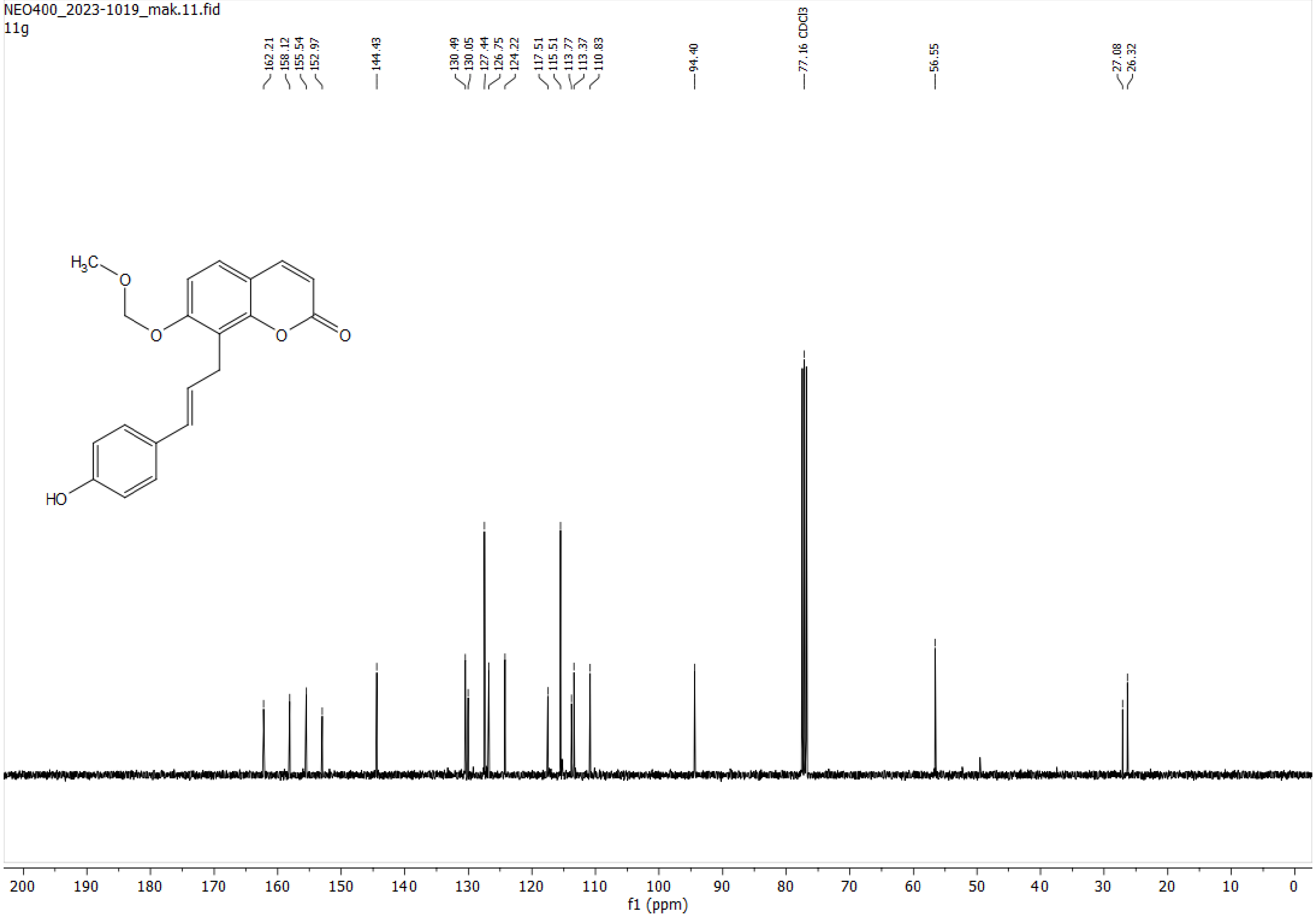

Figure S42: ^{1}H NMR (400 MHz, $CDCl_3$) of **12ah**

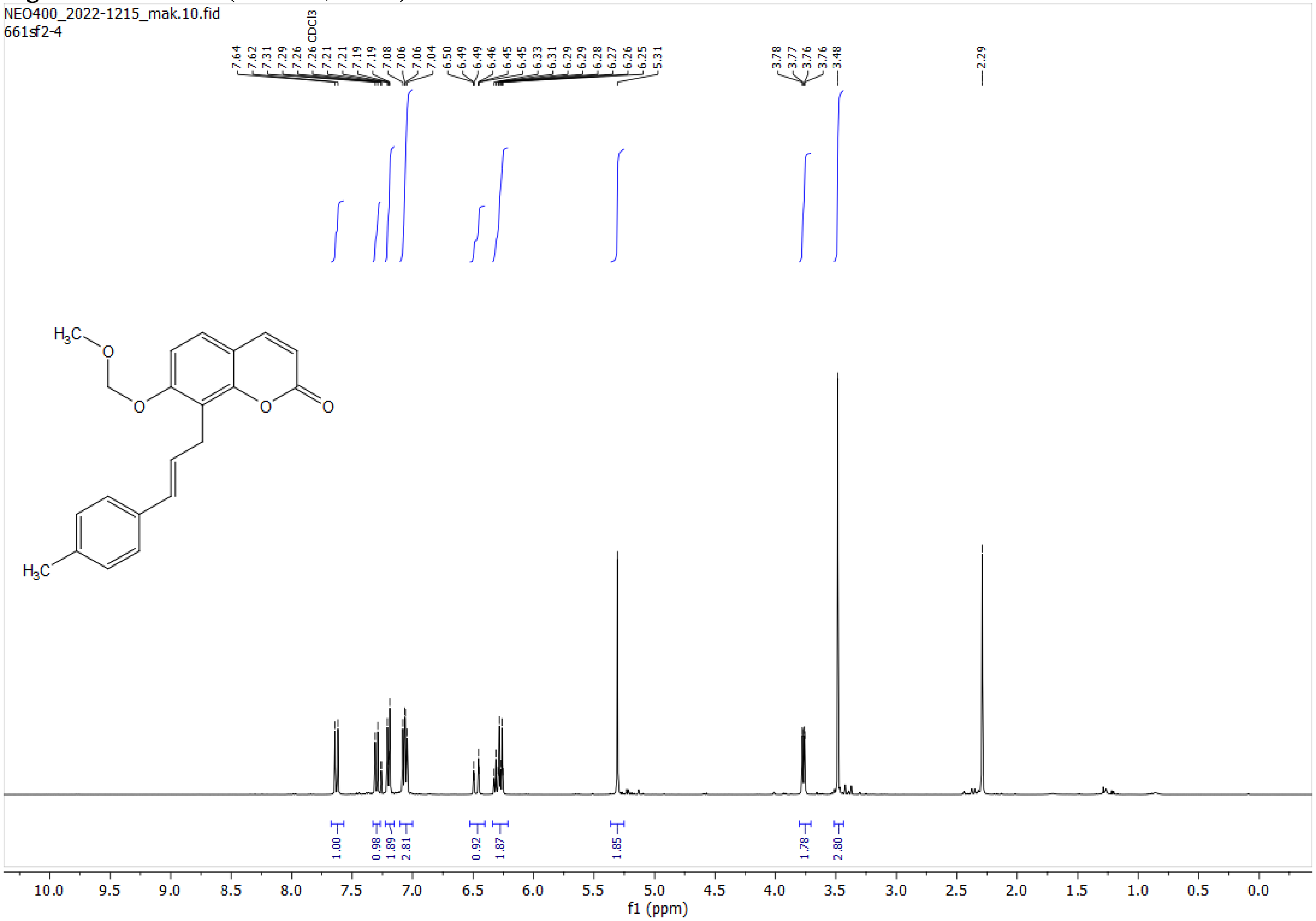

Figure S43: $^{13}C\{^{1}H\}$ NMR (100 MHz, $CDCl_3$) of **12ah**

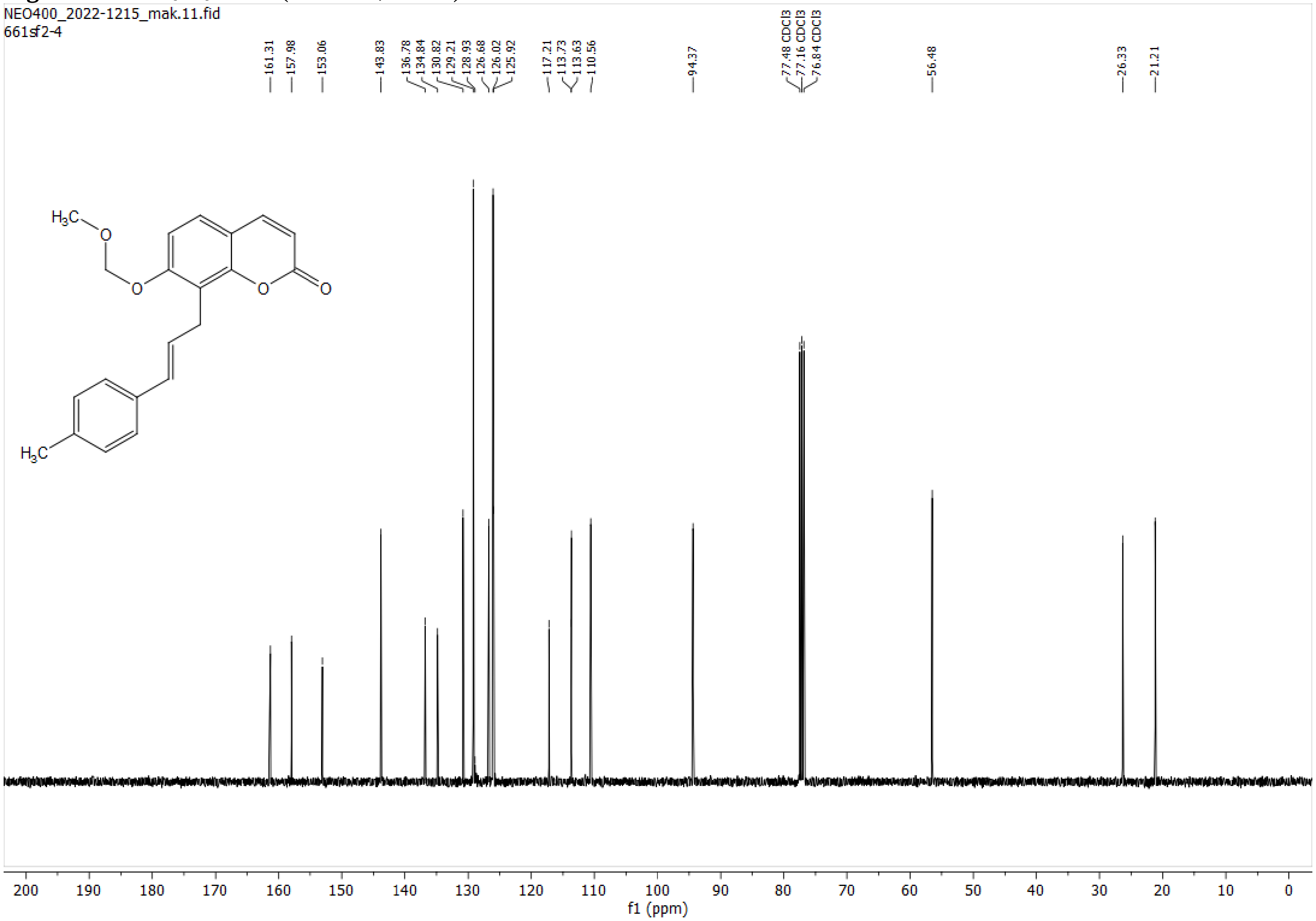

Figure S44: COSY (400 MHz, $CDCl_3$) of **12ah**

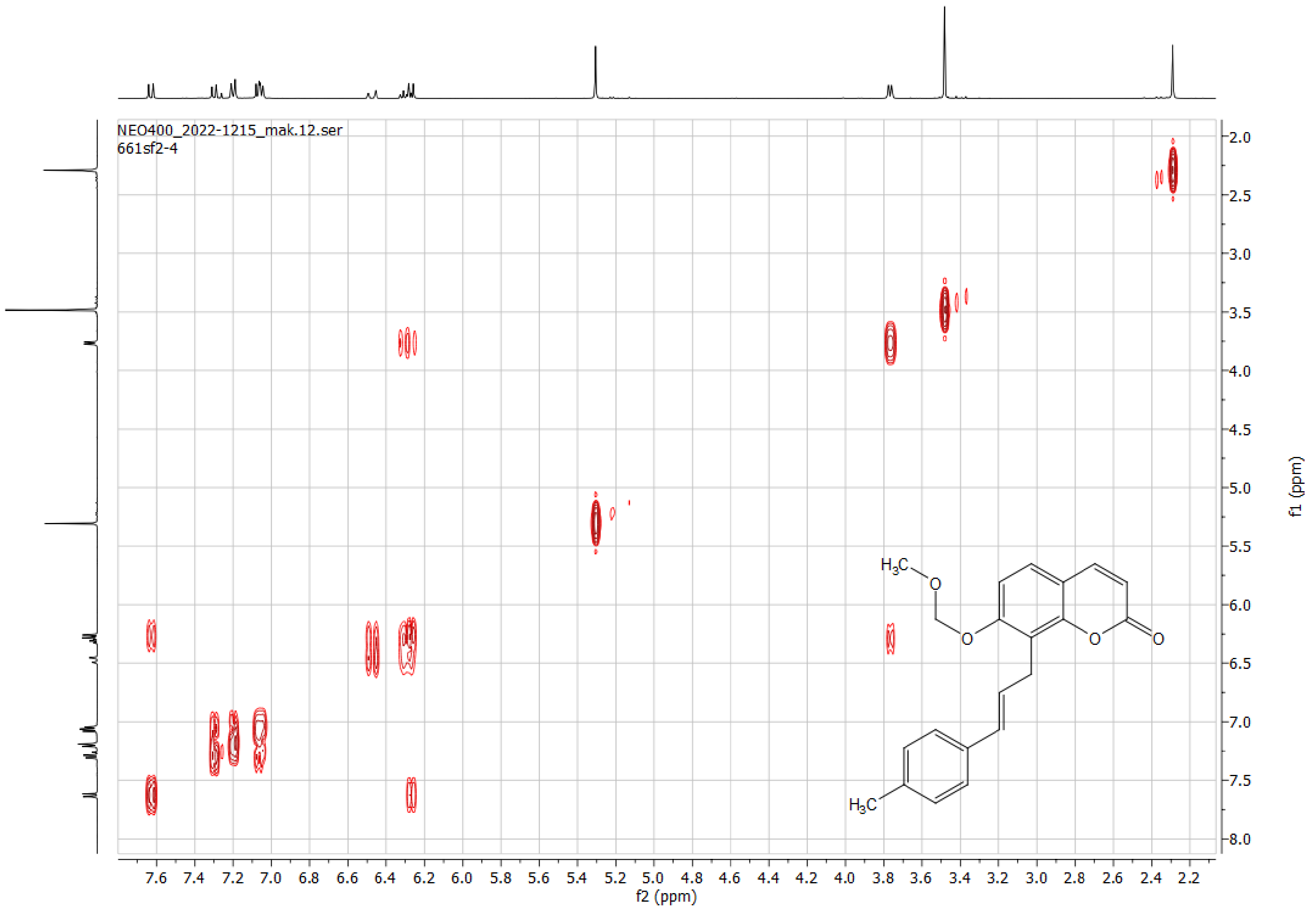

Figure S45: HSQC (400/100 MHz, $CDCl_3$) of **12ah**

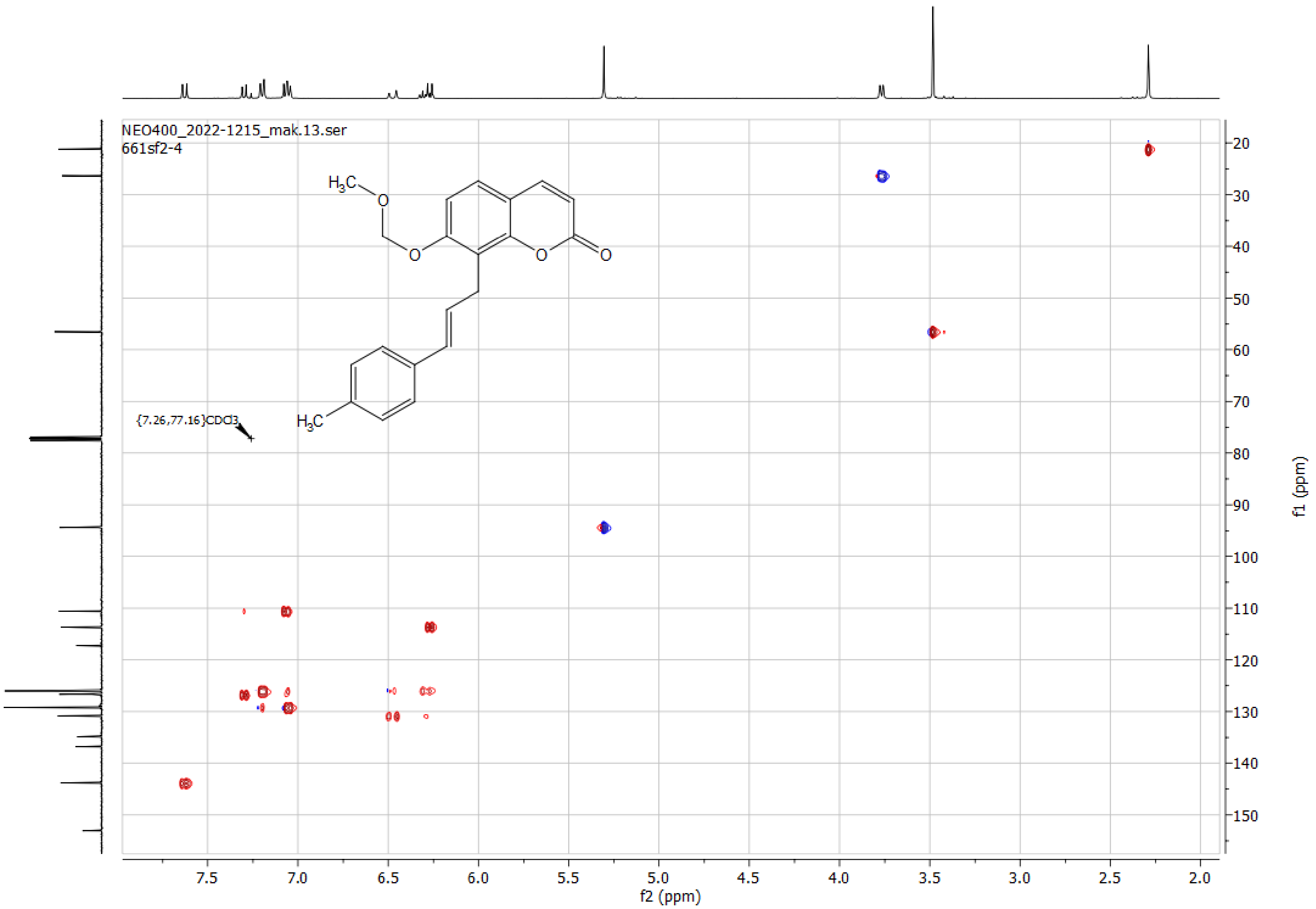

Figure S46: HMBC (400/100 MHz, $CDCl_3$) of **12ah**

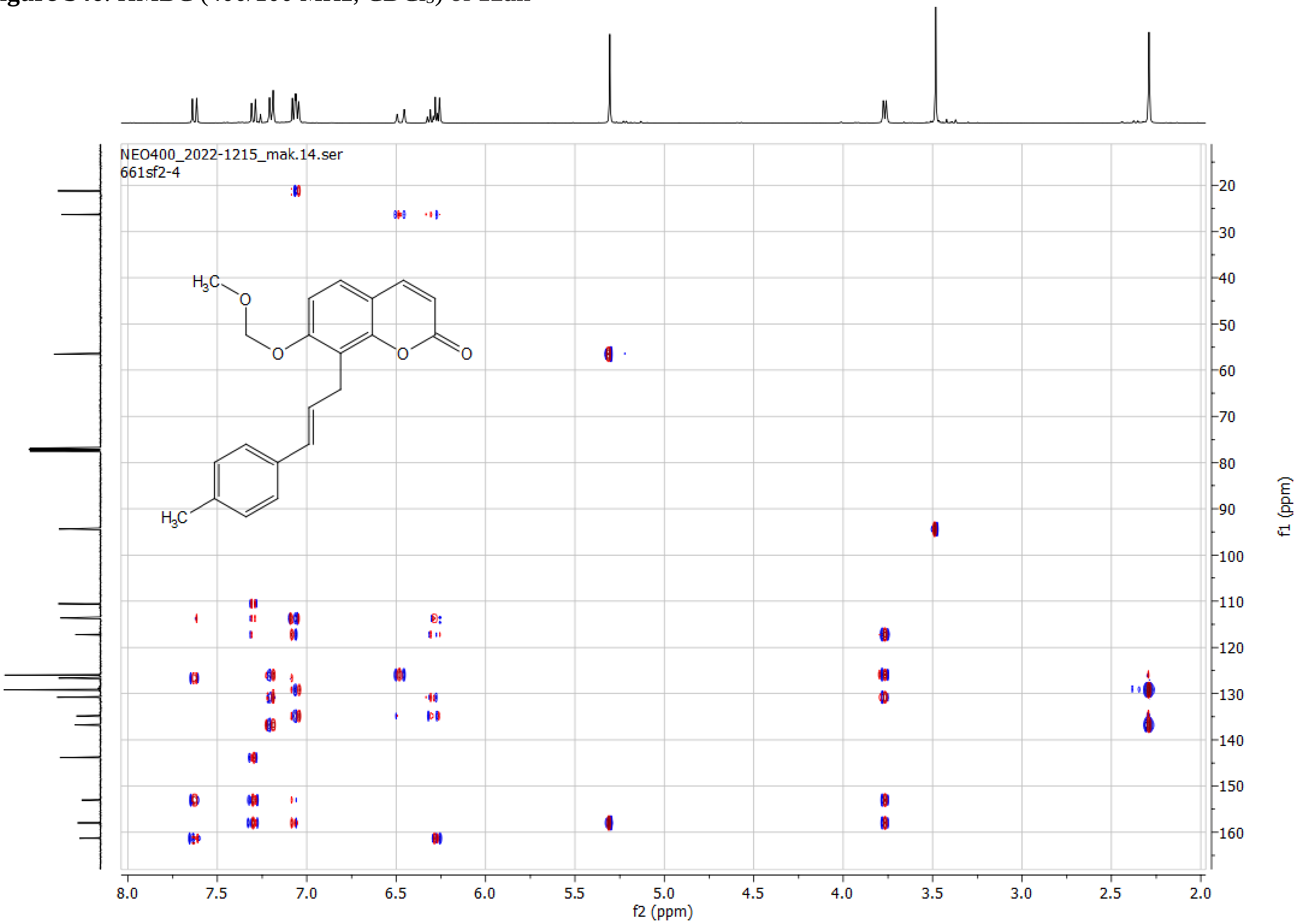

Figure S47: NOESY (400 MHz, $CDCl_3$) of **12ah**

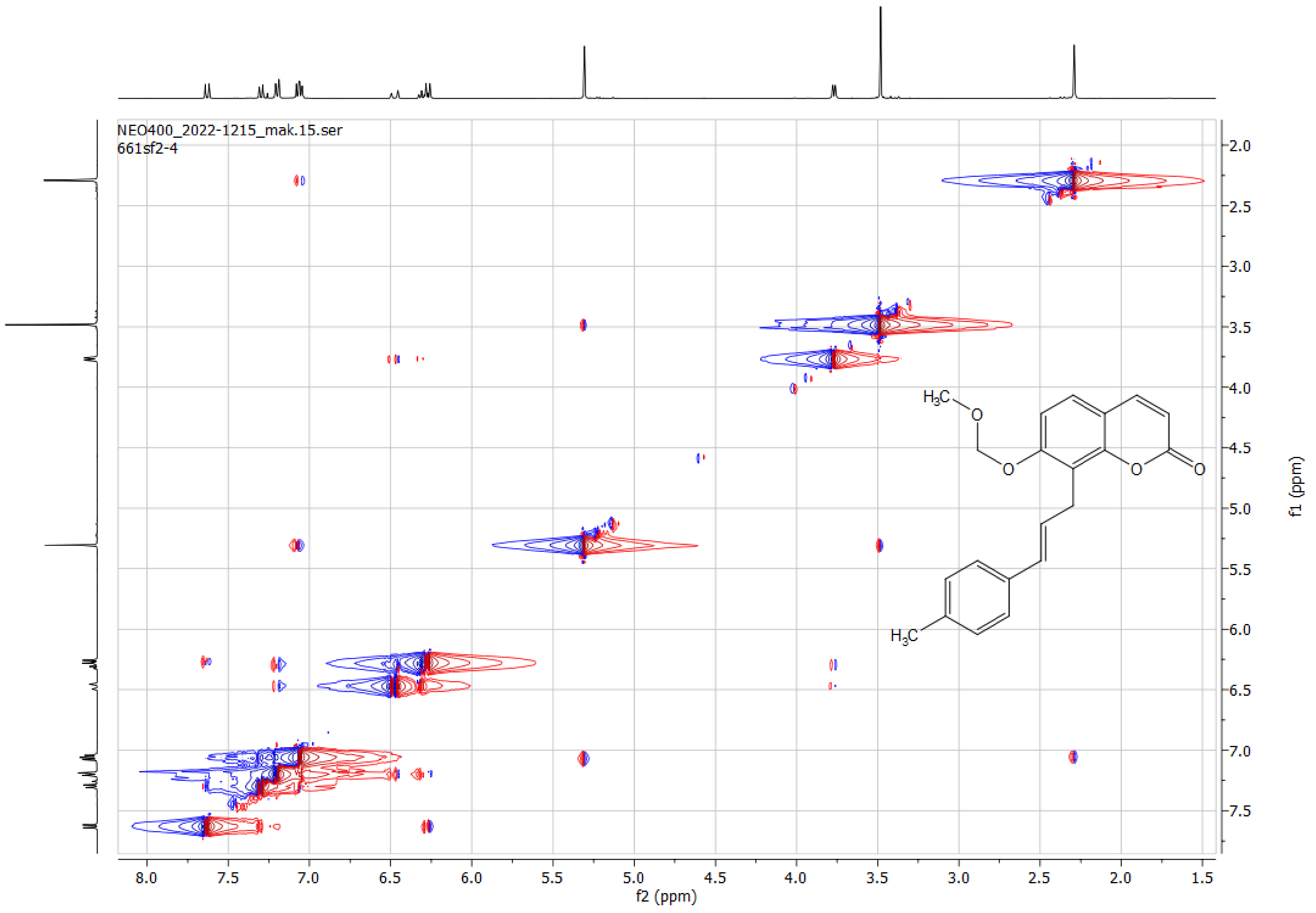

Figure S48: ^{1}H NMR (400 MHz, $CDCl_3$) of **12ai**

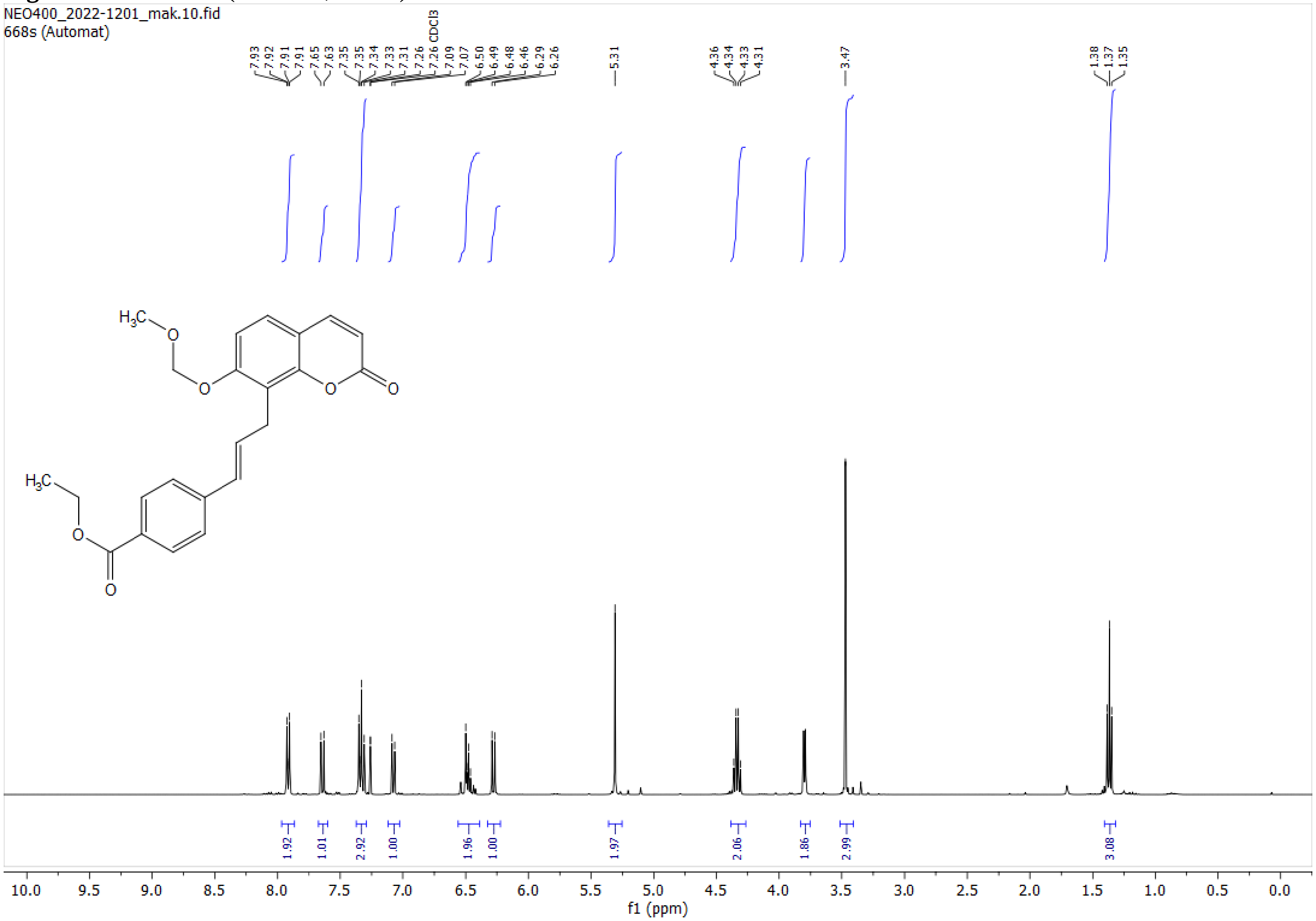

Figure S49: $^{13}C\{^{1}H\}$ NMR (100 MHz, $CDCl_3$) of **12ai**

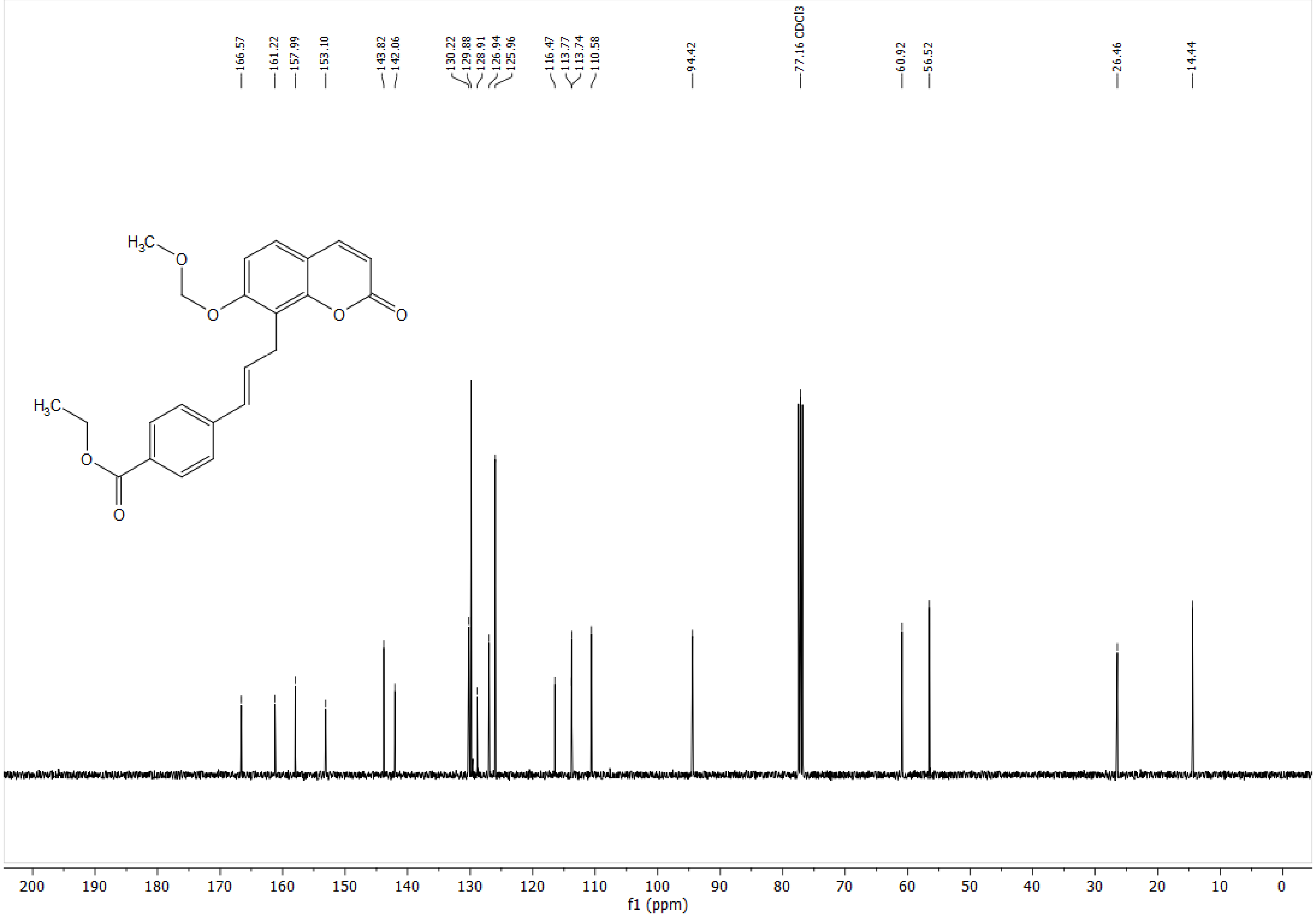

Figure S50: COSY (400 MHz, $CDCl_3$) of **12ai**

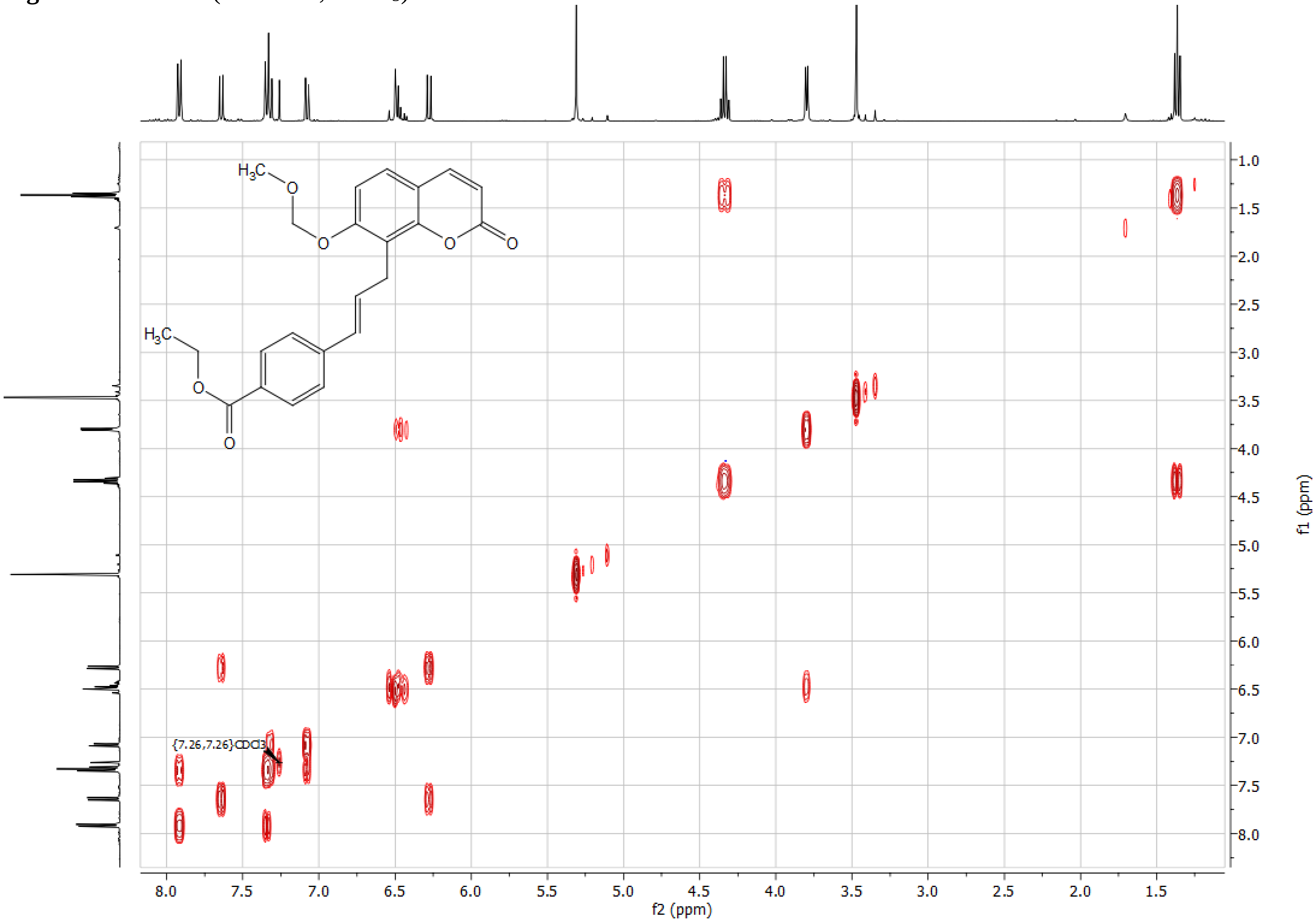

Figure S51: HSQC (400/100 MHz, $CDCl_3$) of **12ai**

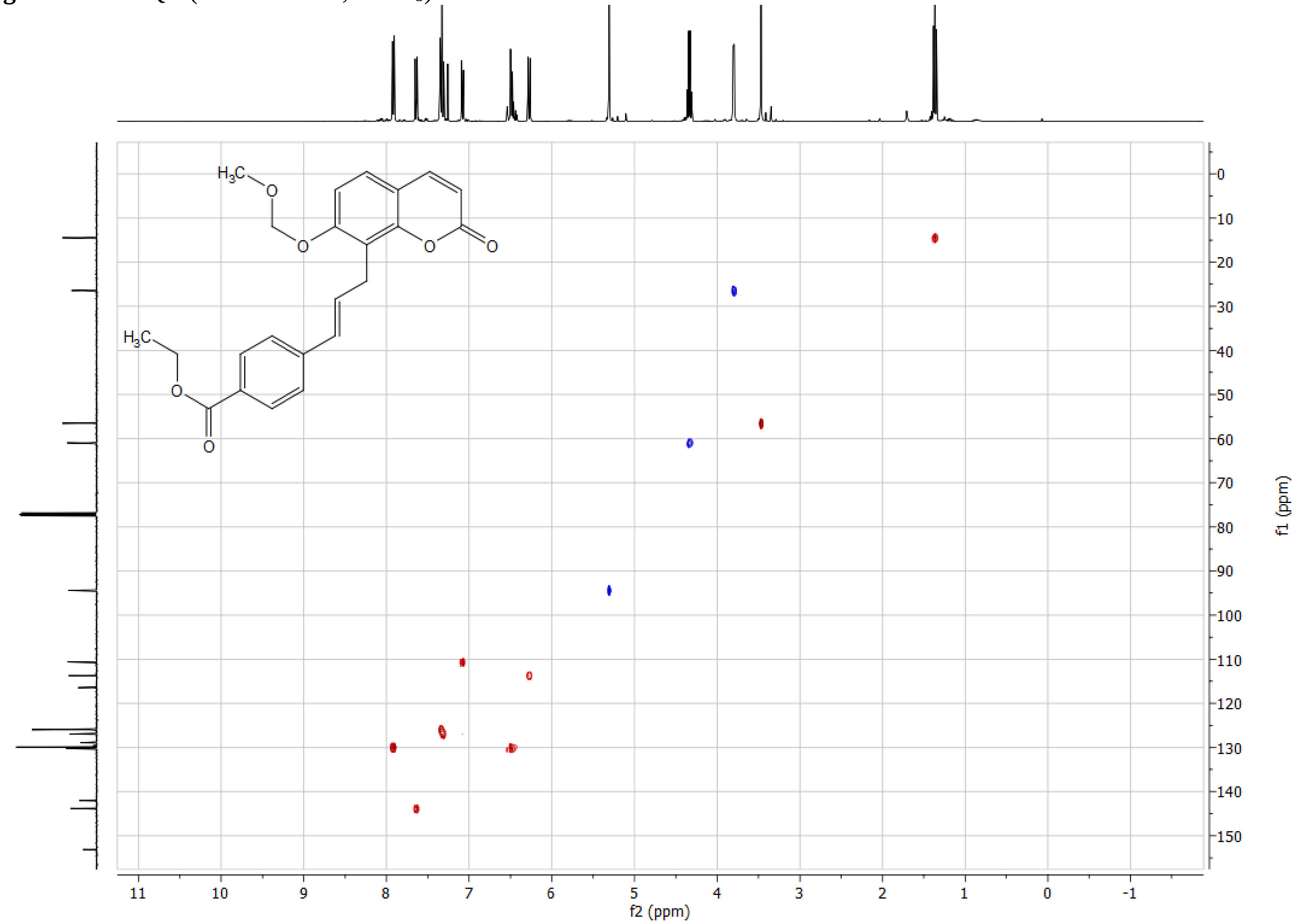

Figure S52: HMBC (400/100 MHz, $CDCl_3$) of **12ai**

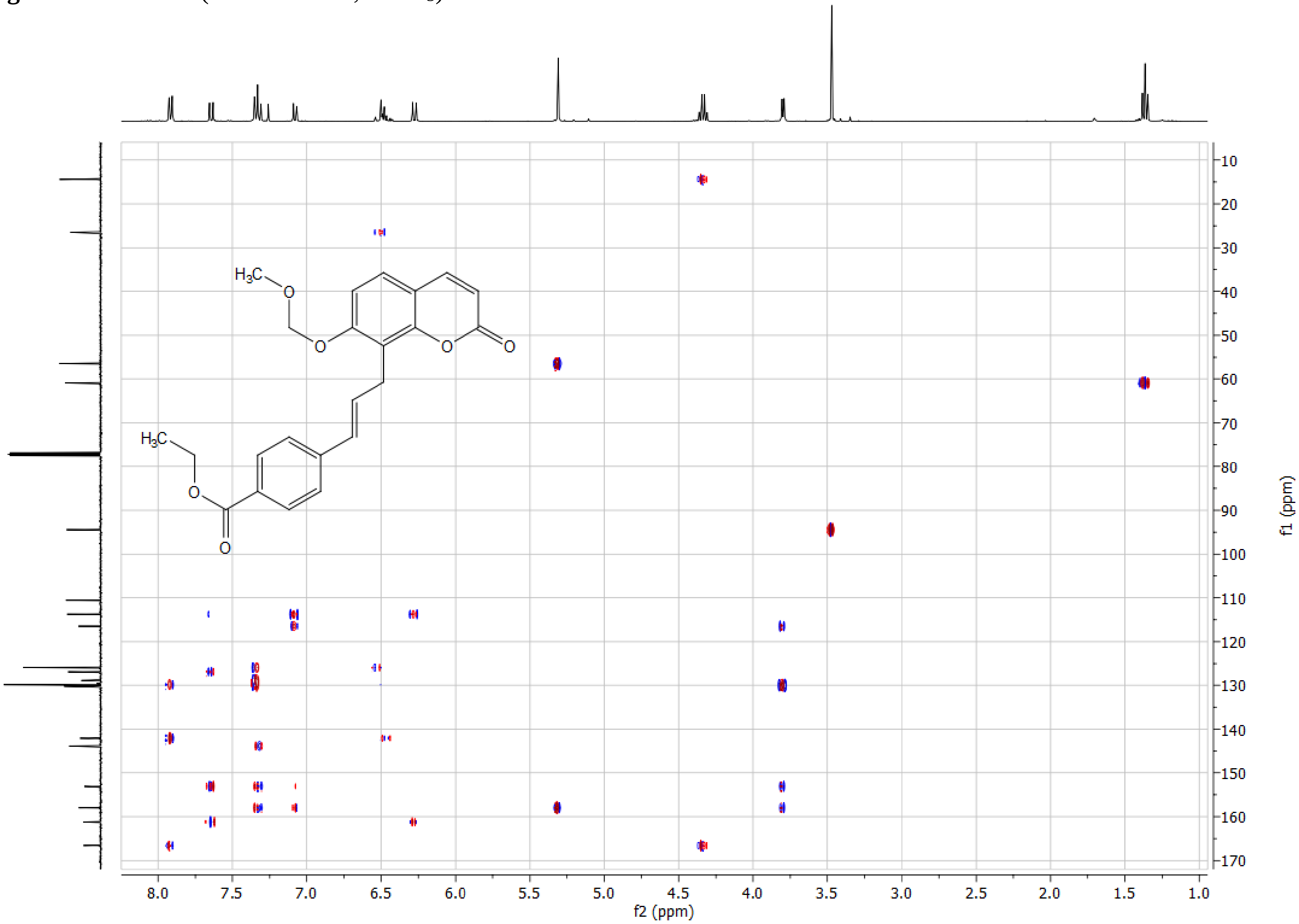

Figure S53: NOESY (400 MHz, $CDCl_3$) of **12ai**

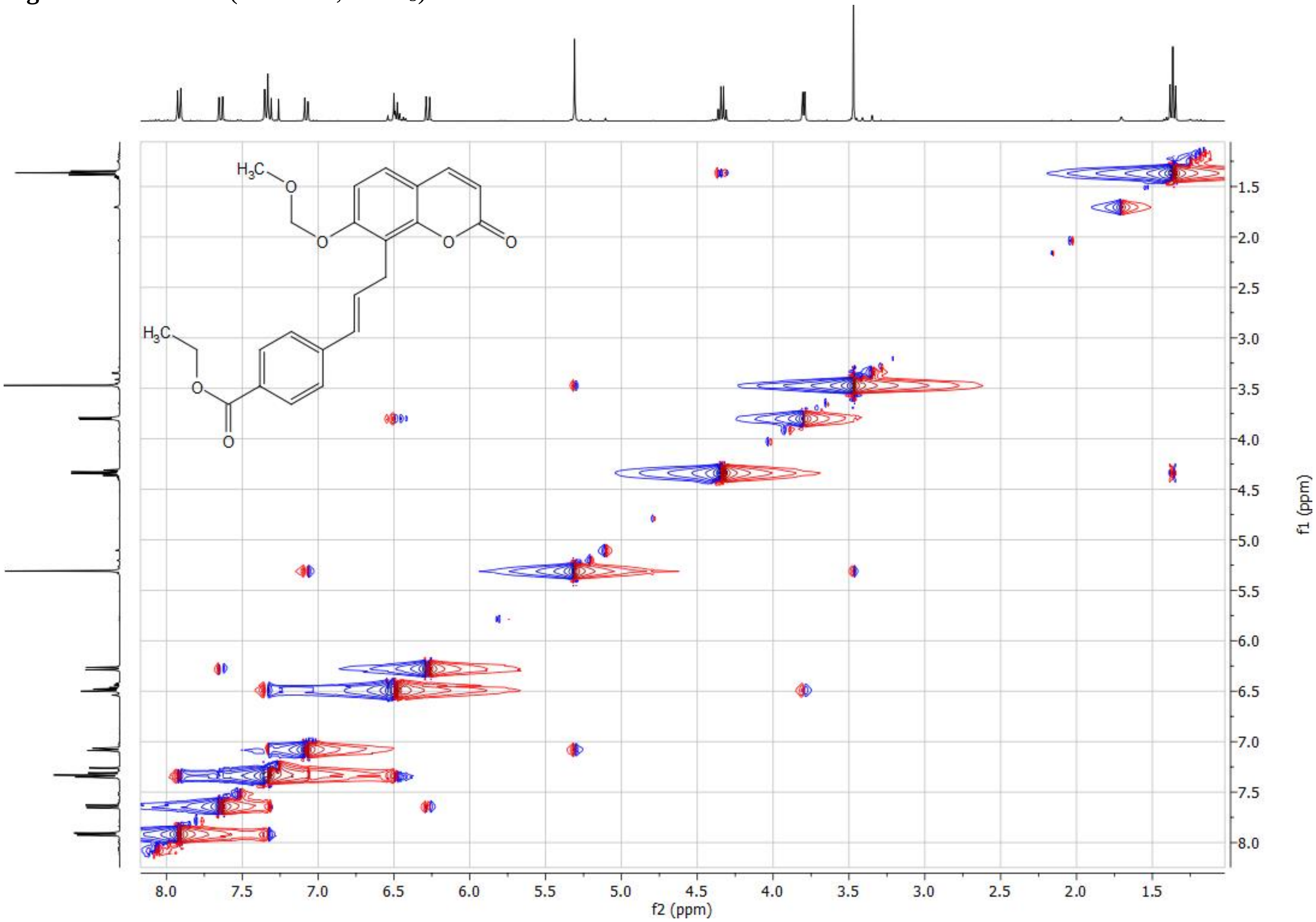

Figure S54: ^{1}H NMR (400 MHz, $CDCl_3$) of **12ak**

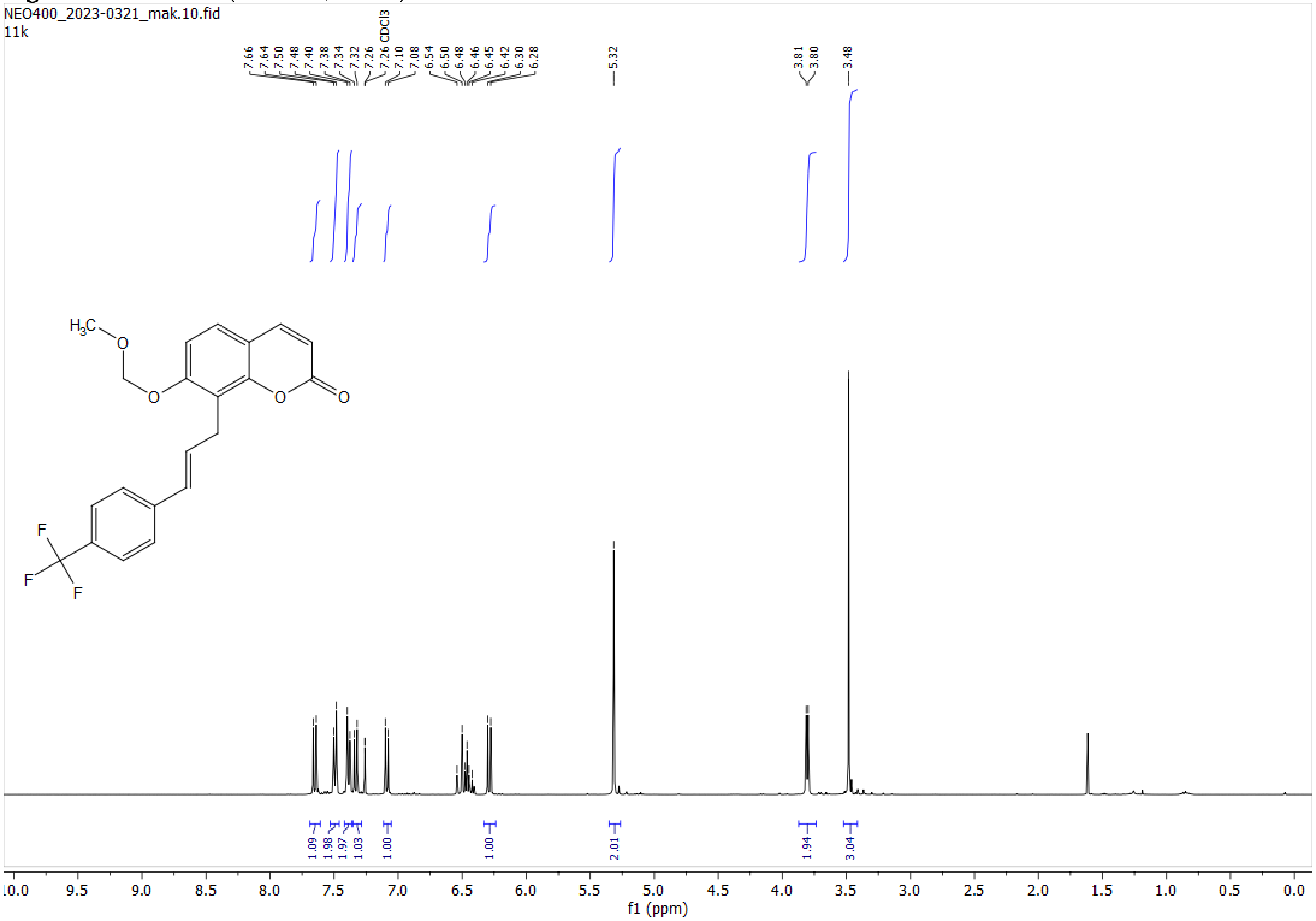

Figure S55: $^{13}C\{^{1}H\}$ NMR (100 MHz, $CDCl_3$) of **12ak**

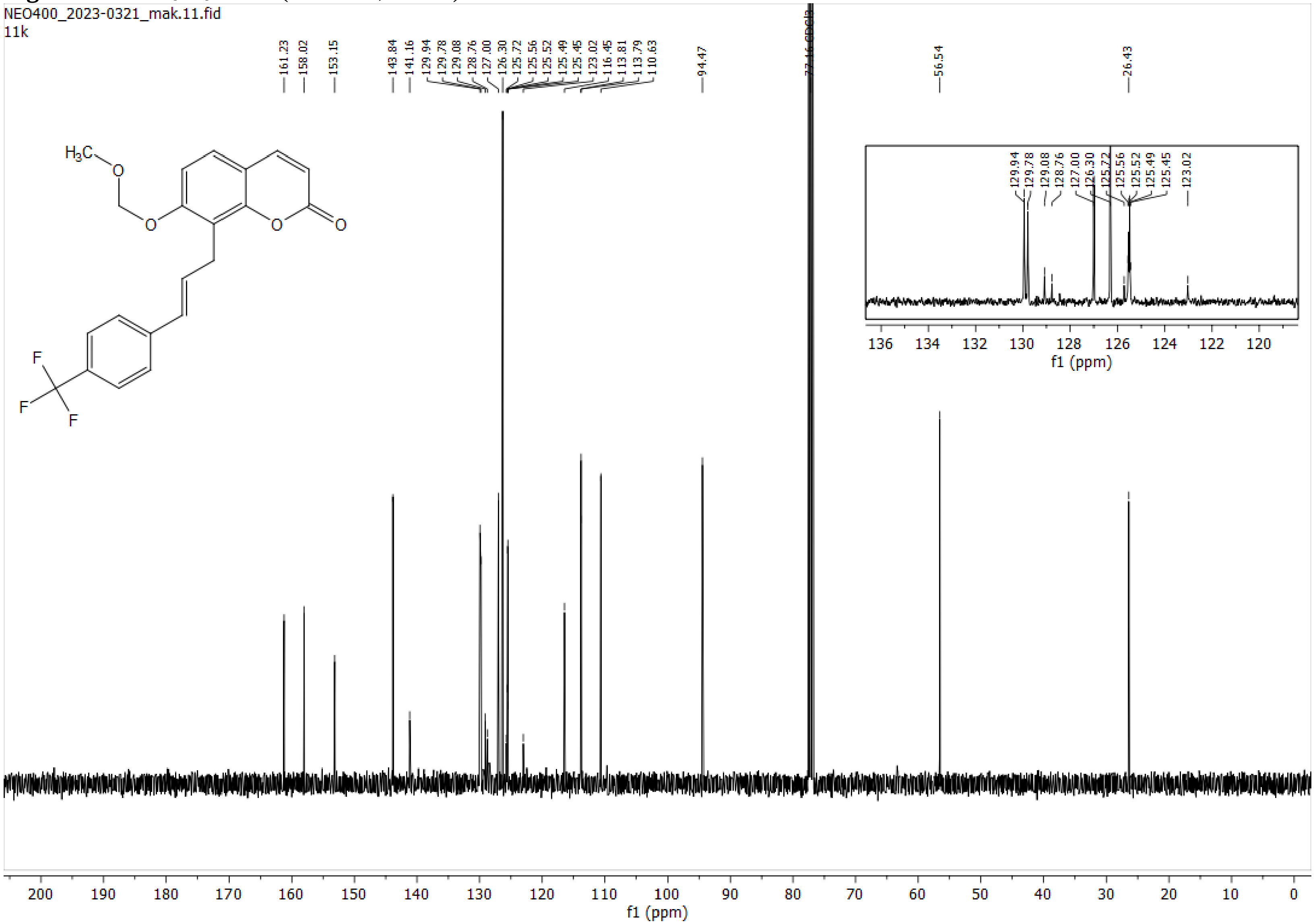

Figure S56: ^{1}H NMR (400 MHz, $CDCl_3$) of **12al**

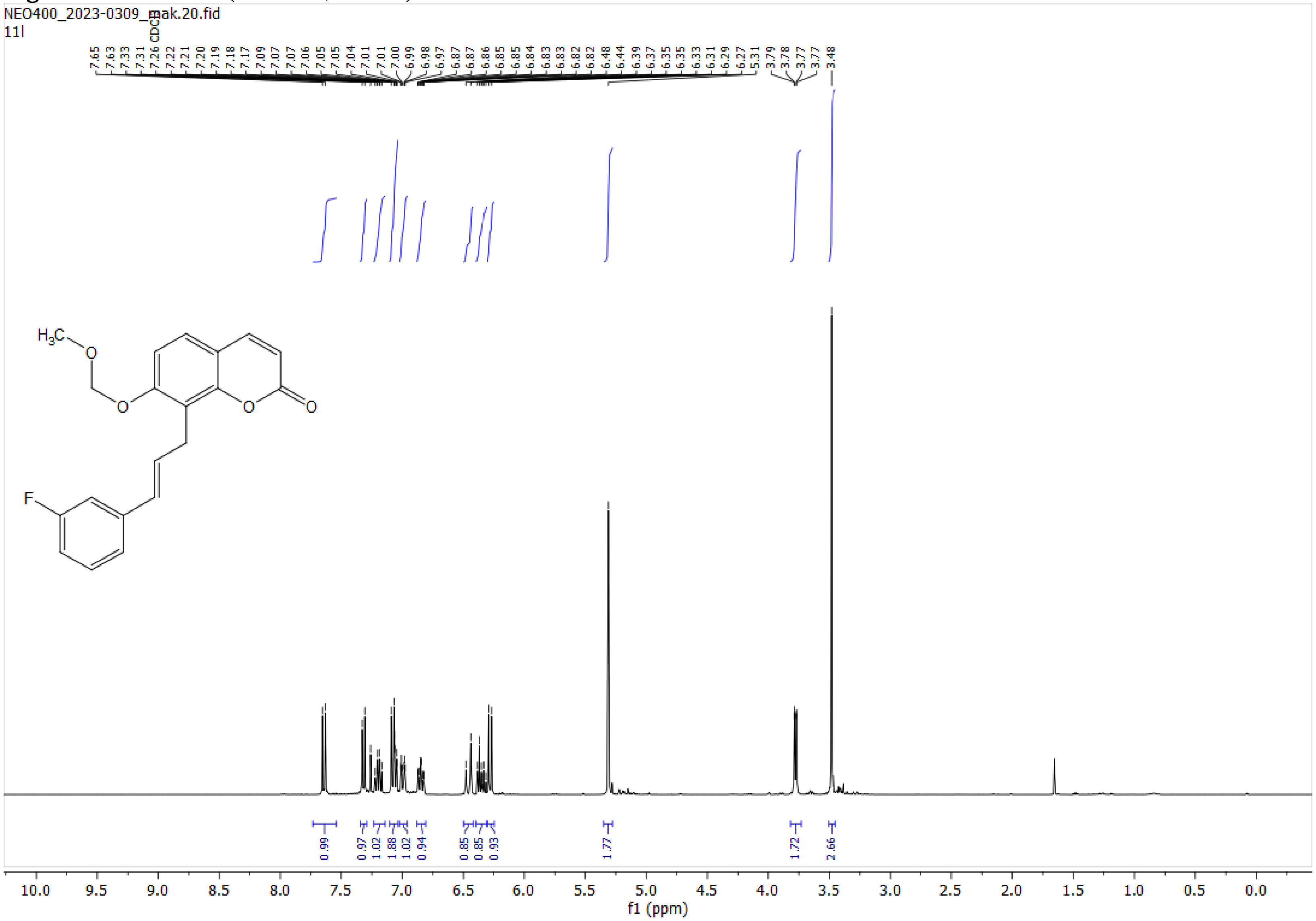

Figure S57: $^{13}C\{^{1}H\}$ NMR (100 MHz, $CDCl_3$) of **12al**

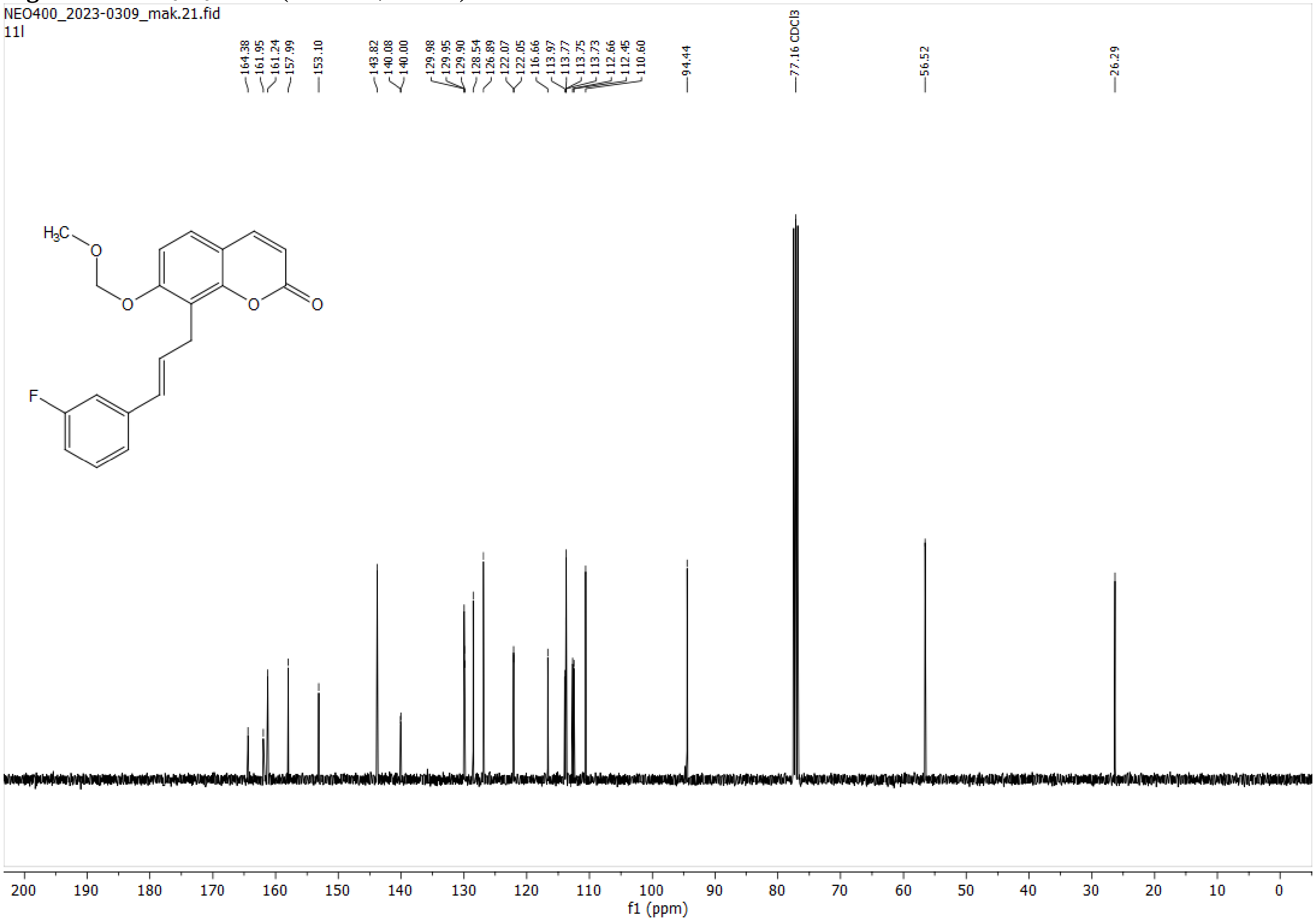

Figure S58: ^{1}H NMR (400 MHz, $CDCl_3$) of **12am**

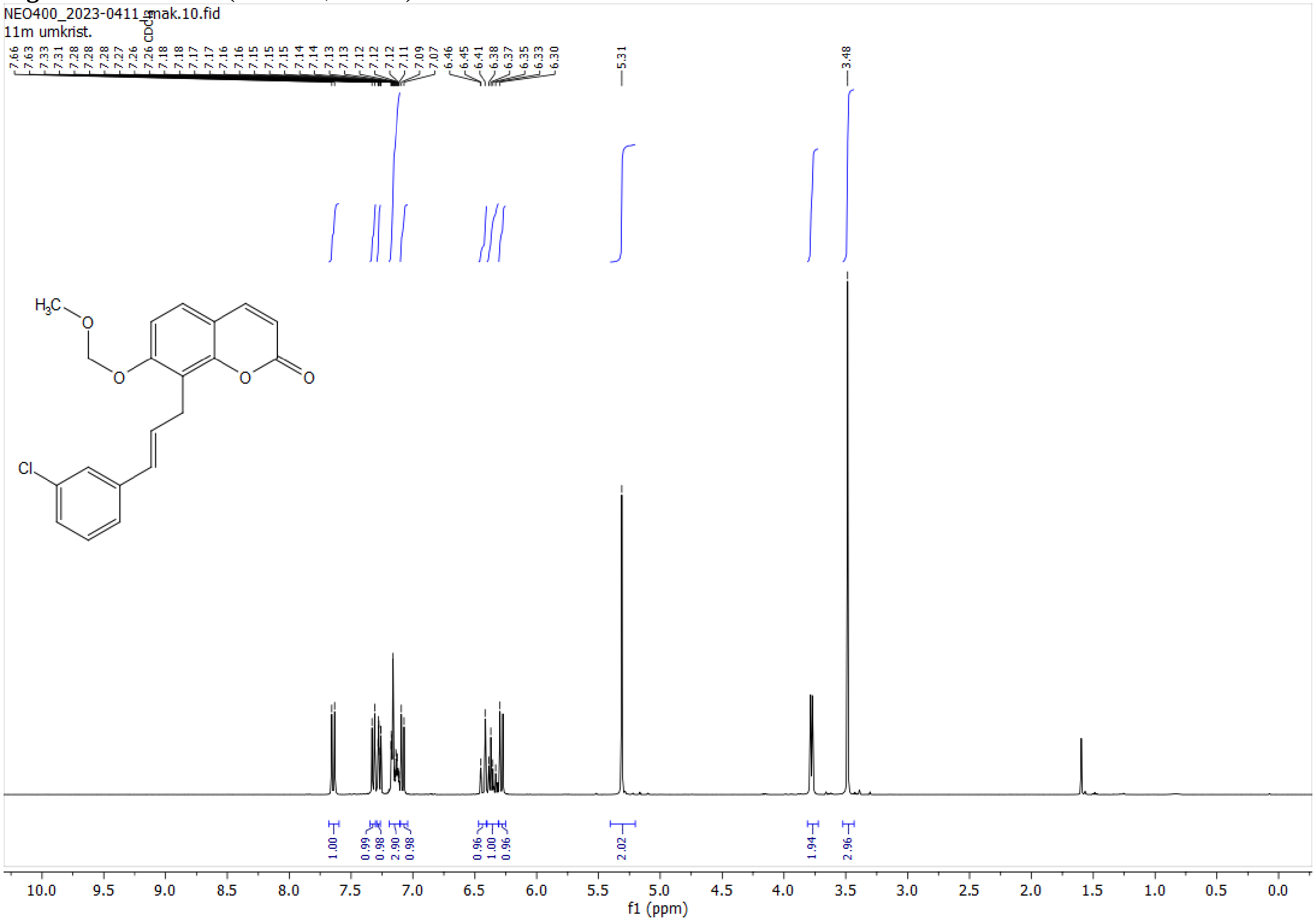

Figure S59: $^{13}C\{^{1}H\}$ NMR (100 MHz, $CDCl_3$) of **12am**

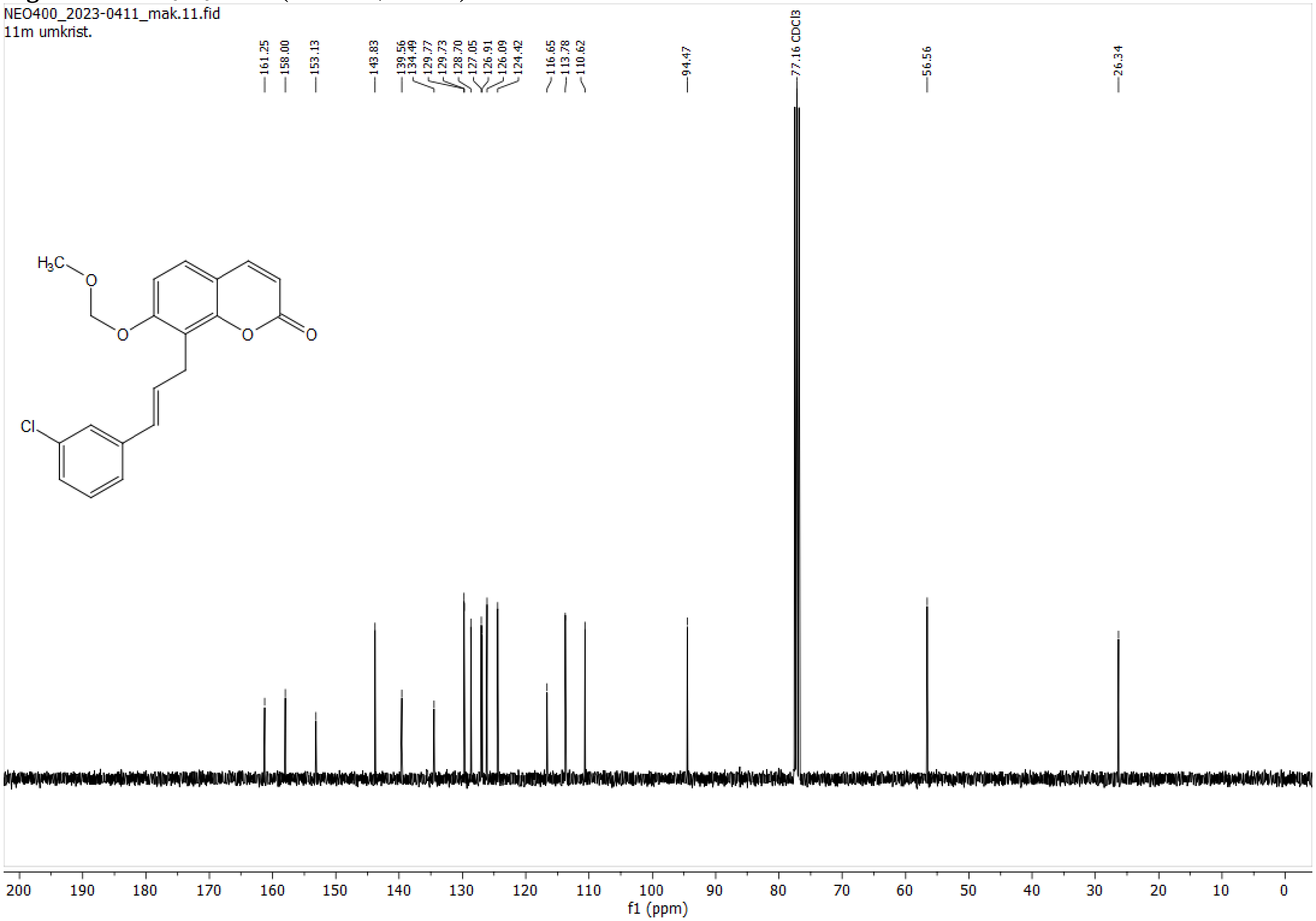

Figure S60: ^{1}H NMR (400 MHz, $CDCl_3$) of **12an**

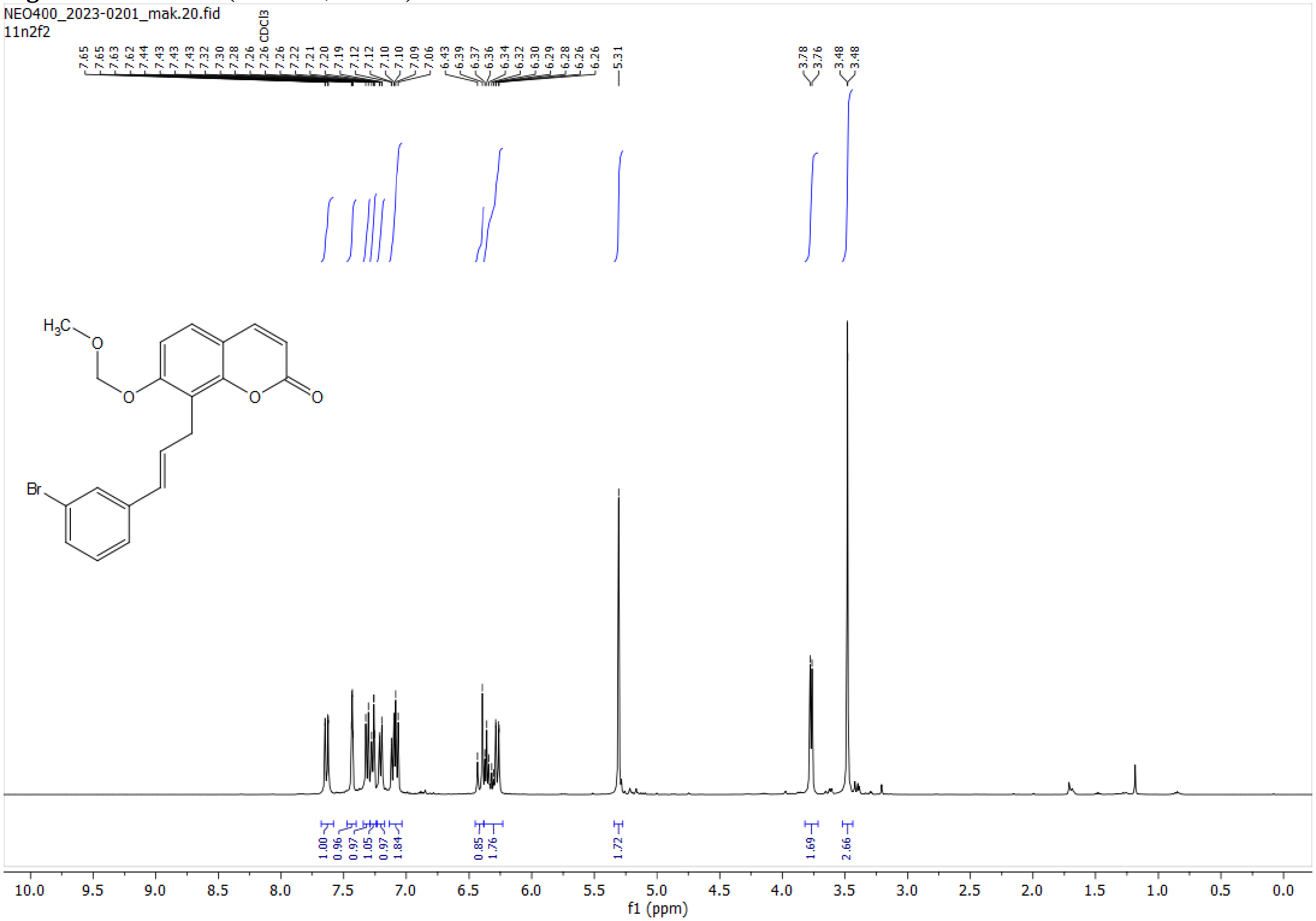

Figure S61: $^{13}C\{^{1}H\}$ NMR (100 MHz, $CDCl_3$) of **12an**

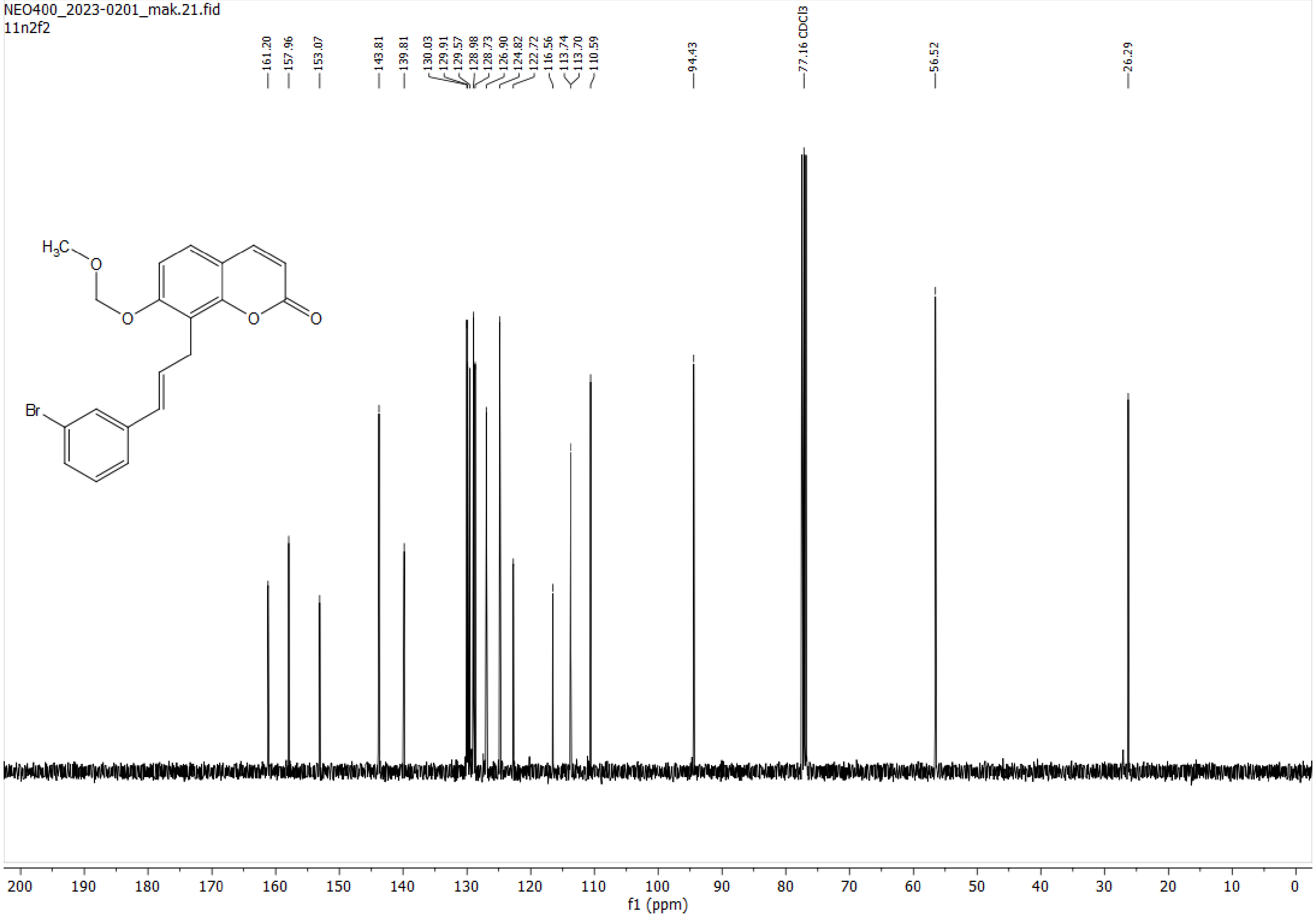

Figure S62: ^{1}H NMR (400 MHz, $CDCl_3$) of **12ao**

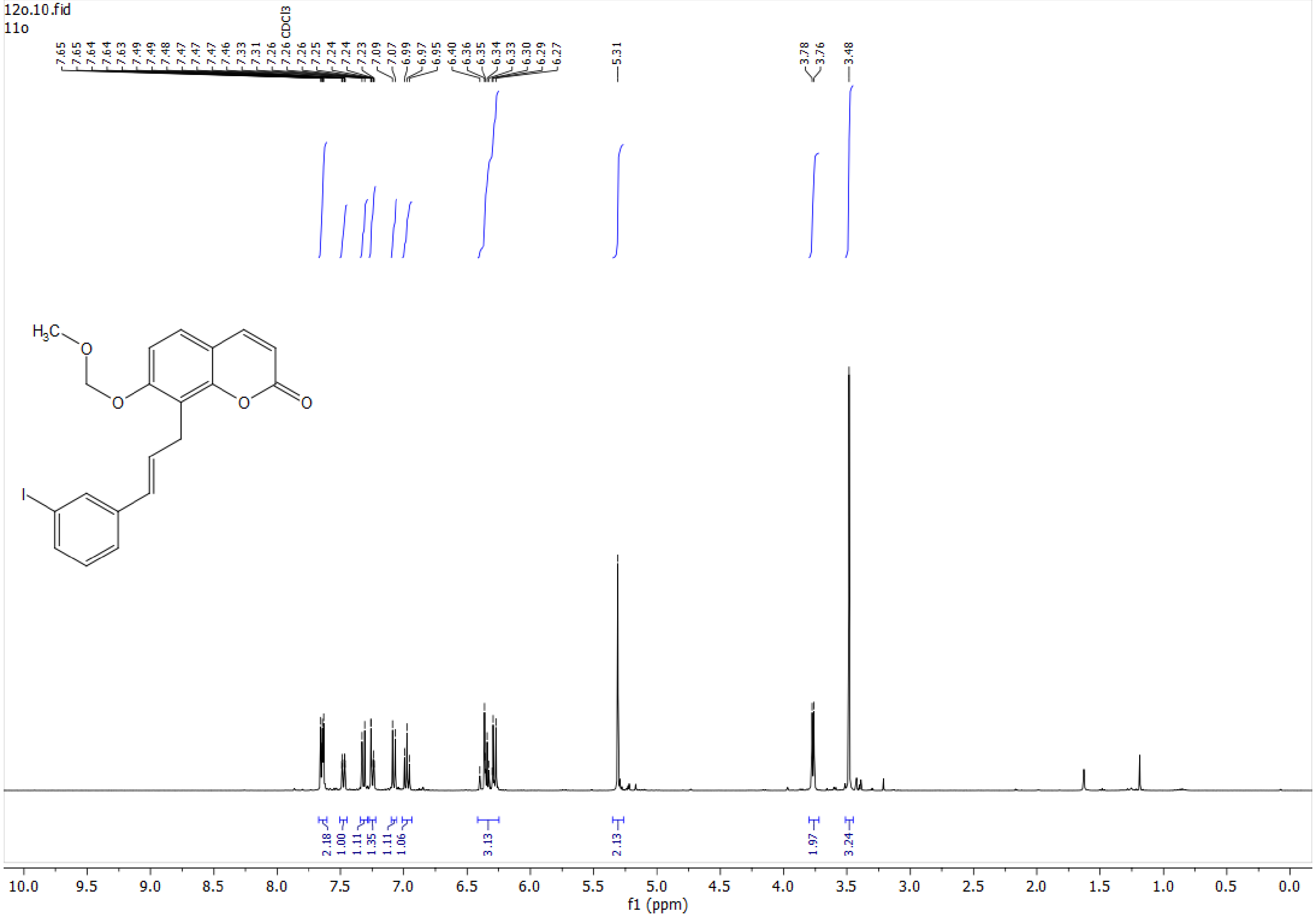

Figure S63: $^{13}C\{^{1}H\}$ NMR (100 MHz, $CDCl_3$) of **12ao**

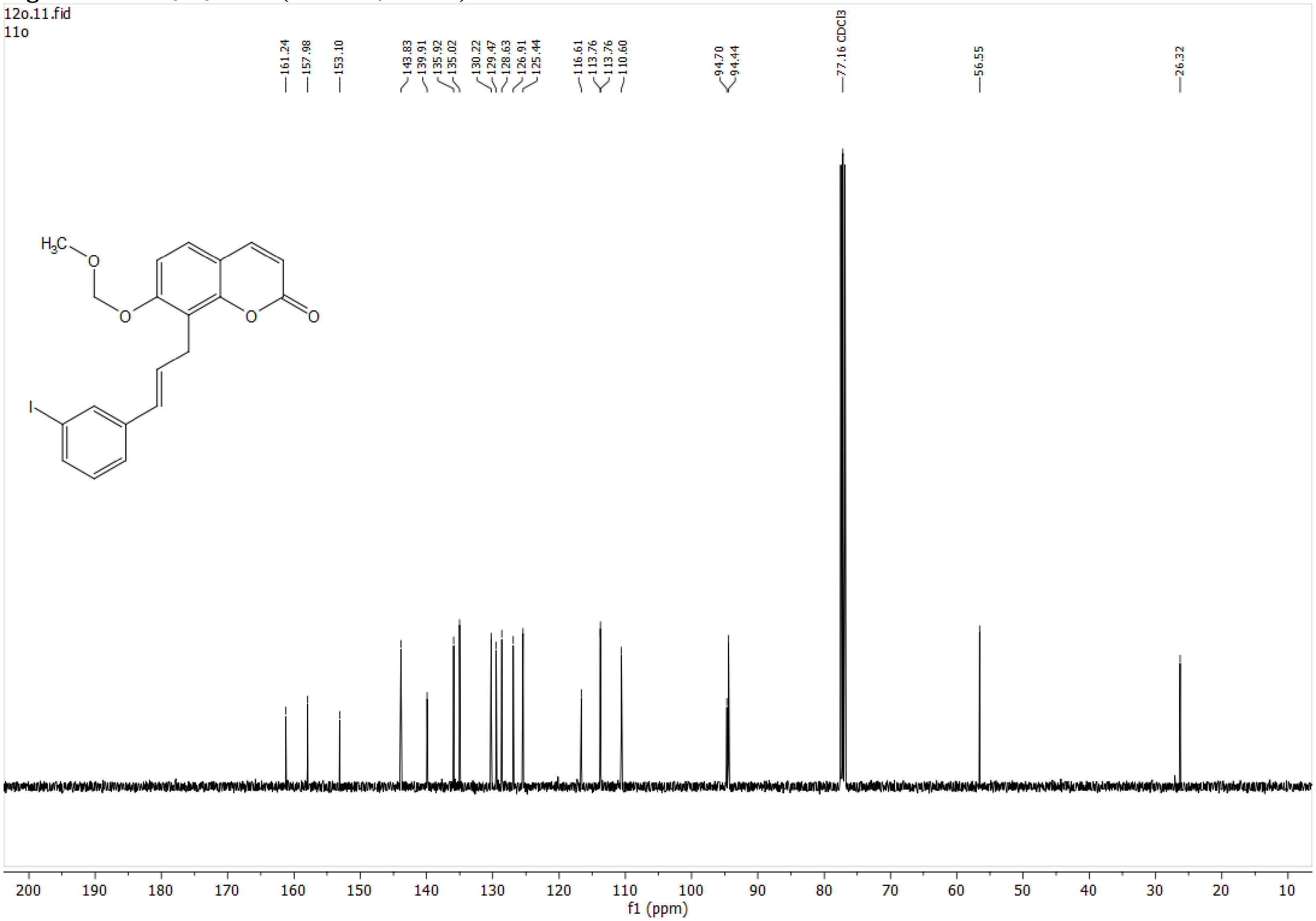

Figure S64: ^{1}H NMR (400 MHz, $CDCl_3$) of **12ap**

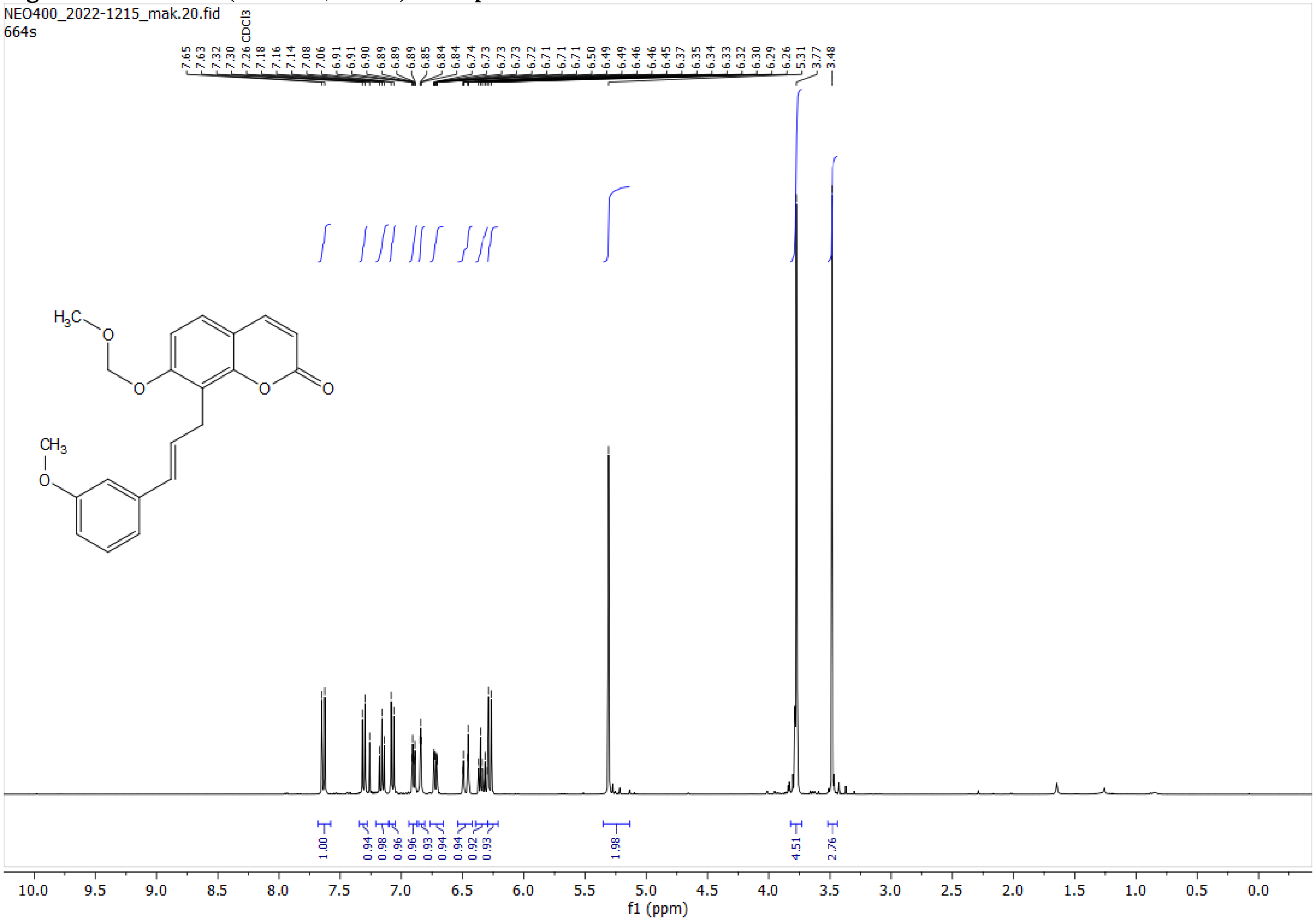

Figure S65: $^{13}C\{^{1}H\}$ NMR (100 MHz, $CDCl_3$) of **12ap**

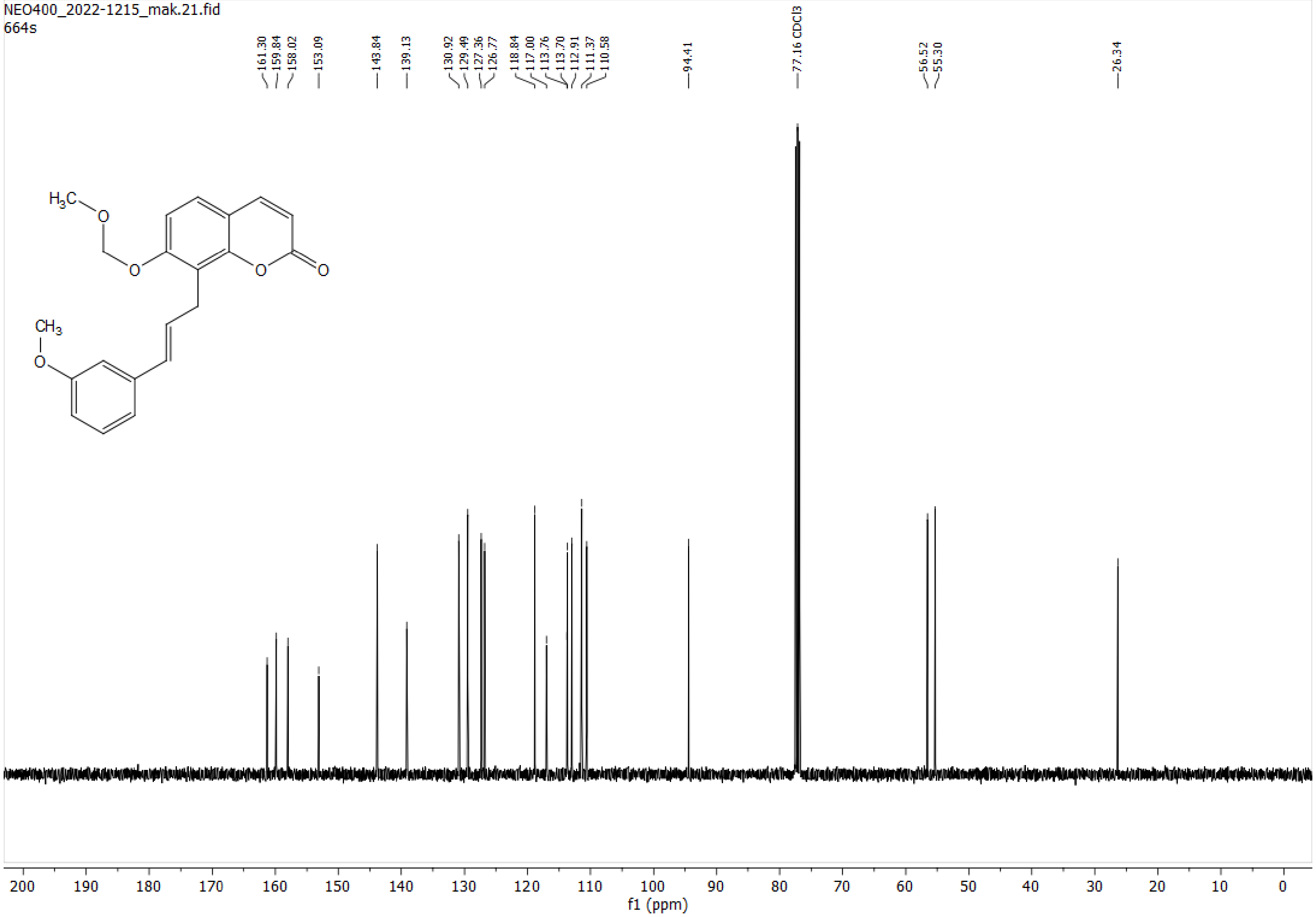

Figure S66: COSY (400 MHz, $CDCl_3$) of **12ap**

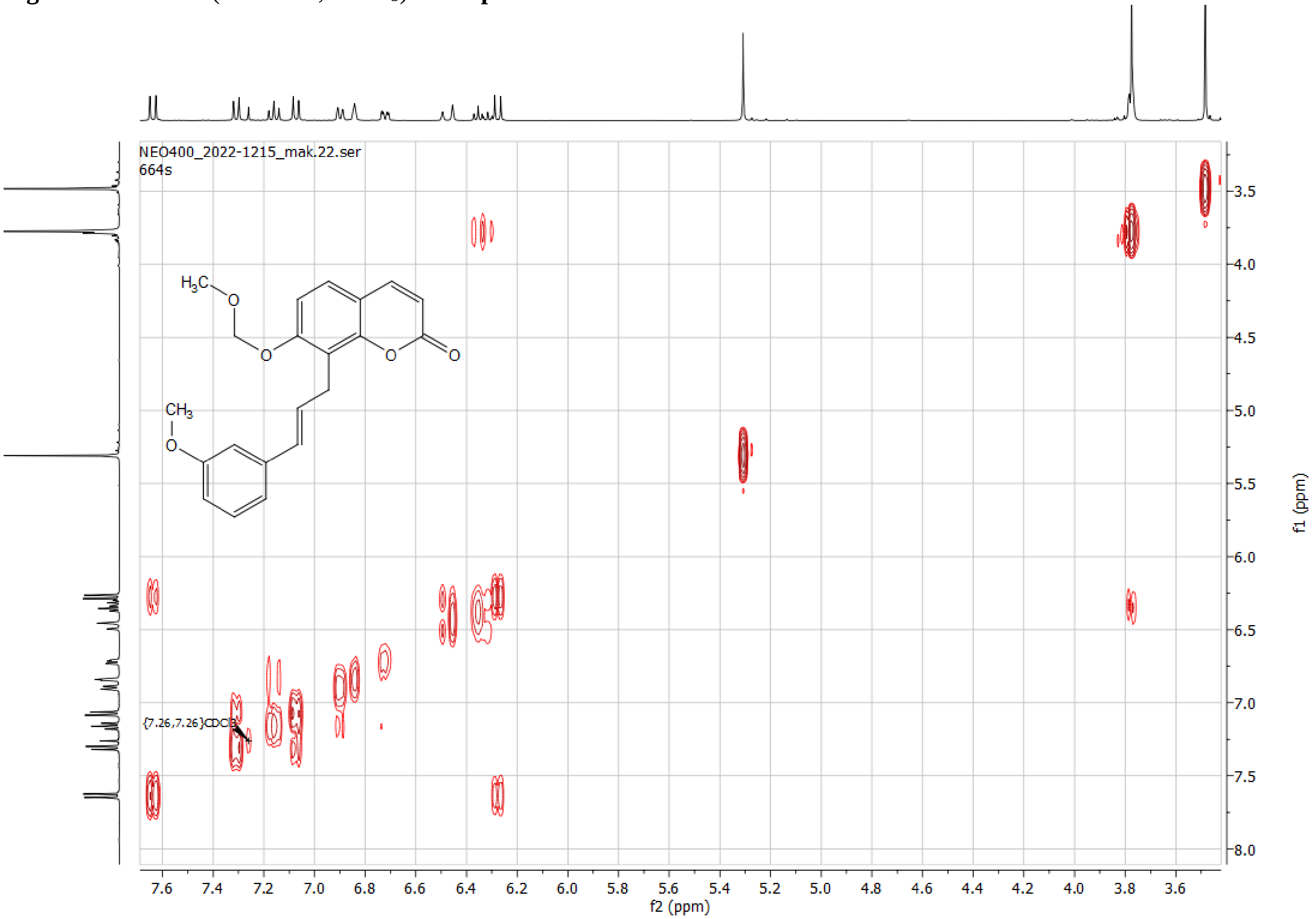

Figure S67: HSQC (400/100 MHz, $CDCl_3$) of **12ap**

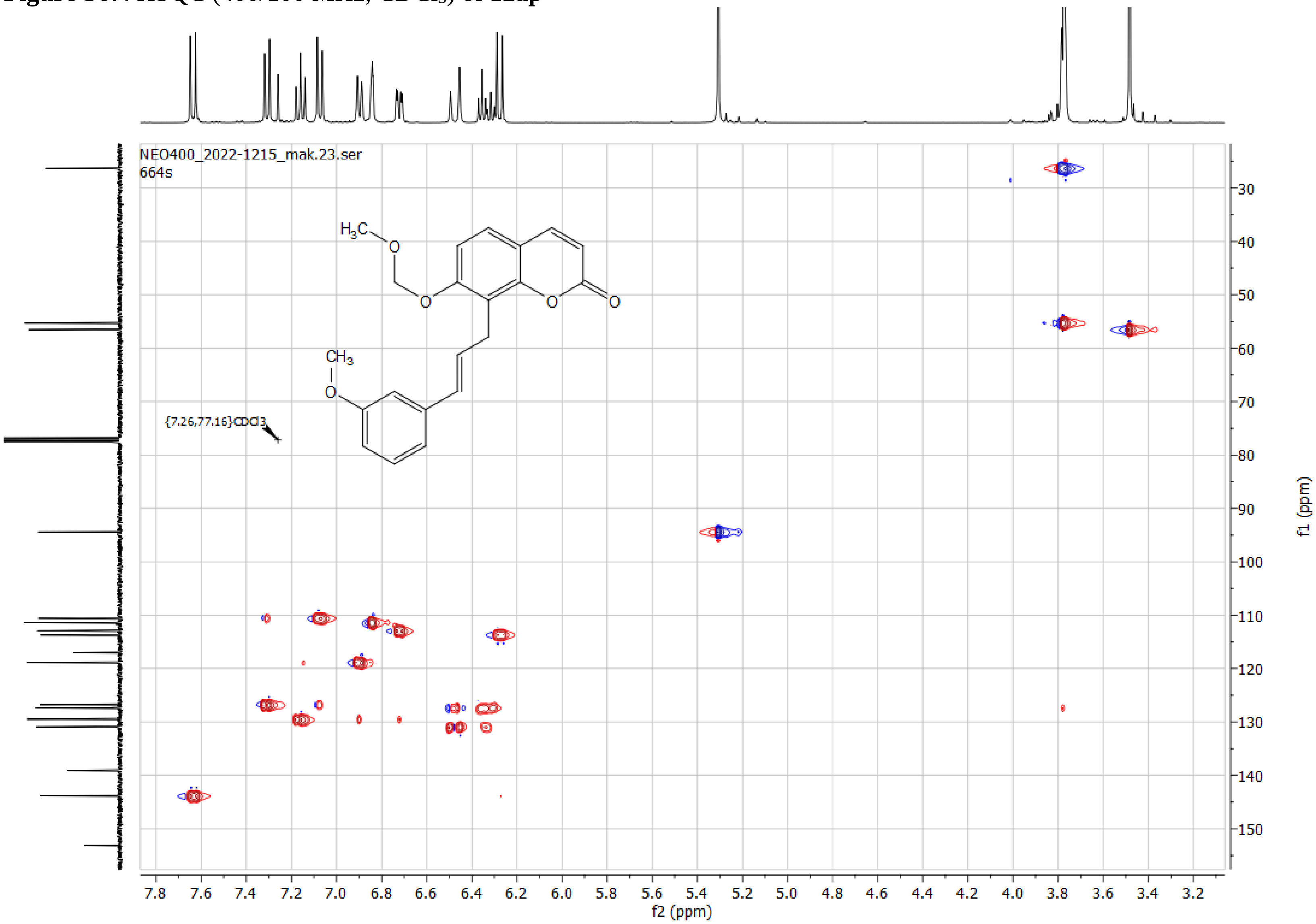

Figure S68: HMBC (400/100 MHz, $CDCl_3$) of **12ap**

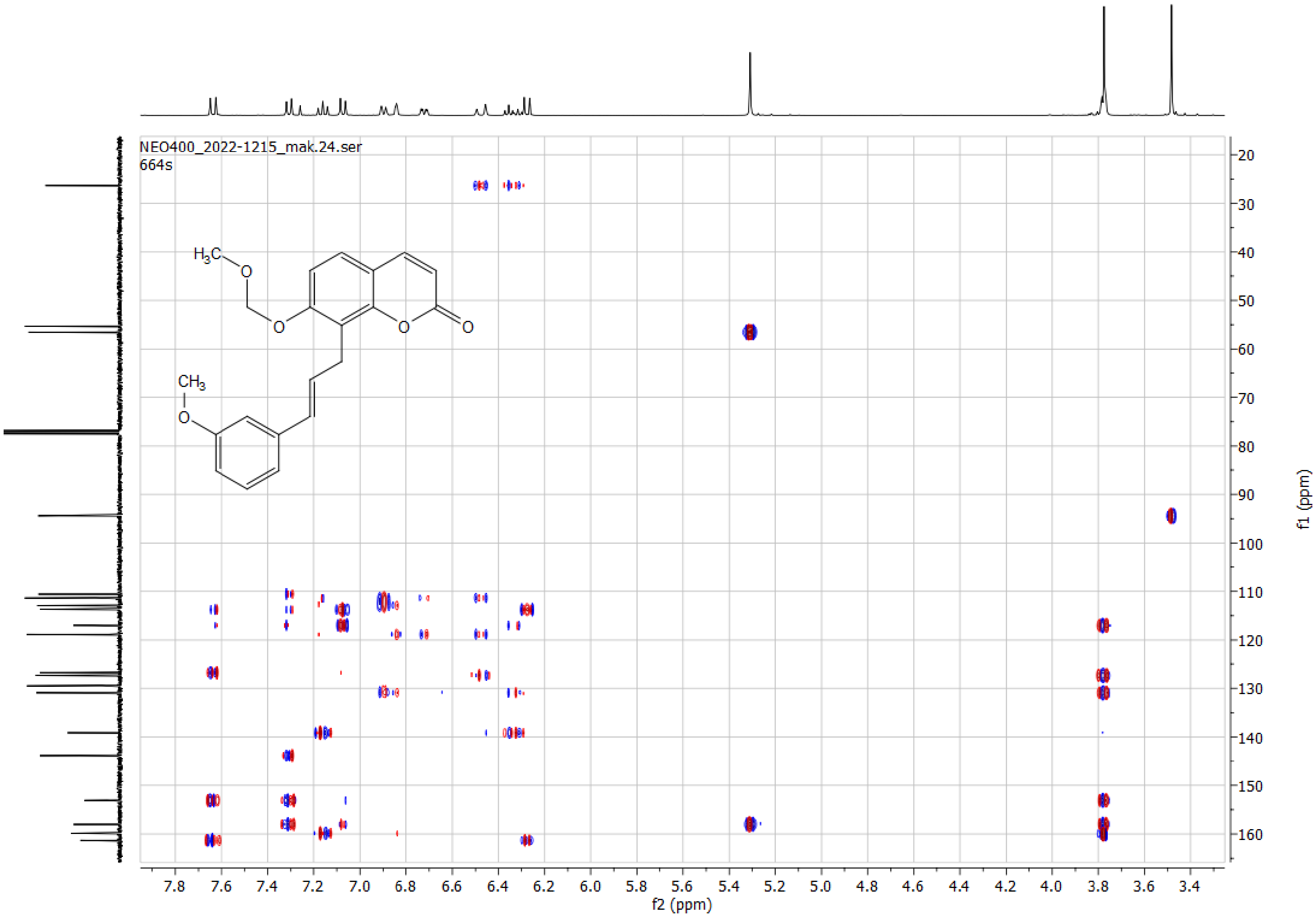

Figure S69: NOESY (400 MHz, $CDCl_3$) of **12ap**

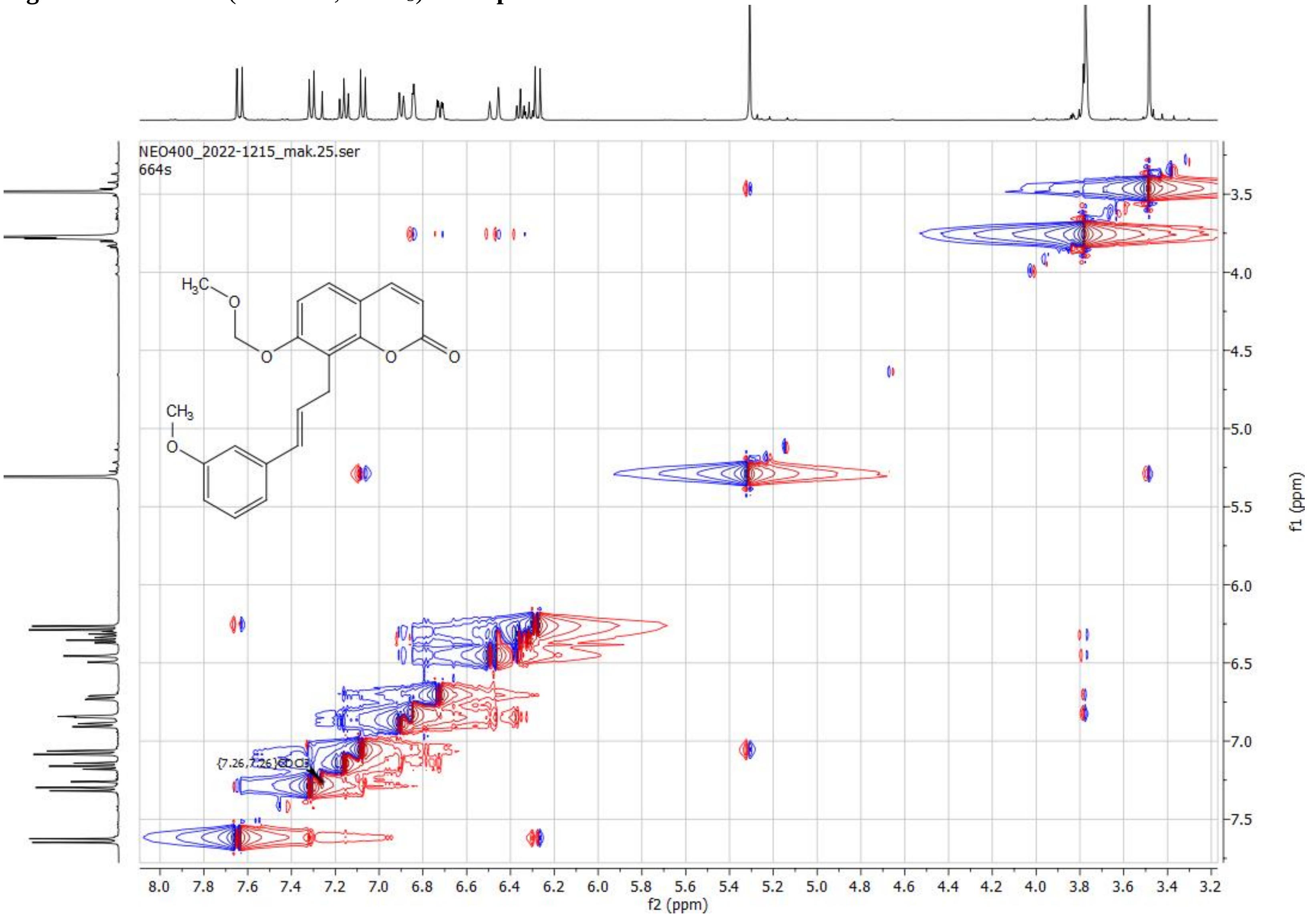

Figure S70: ^{1}H NMR (400 MHz, $CDCl_3$) of **12aq**

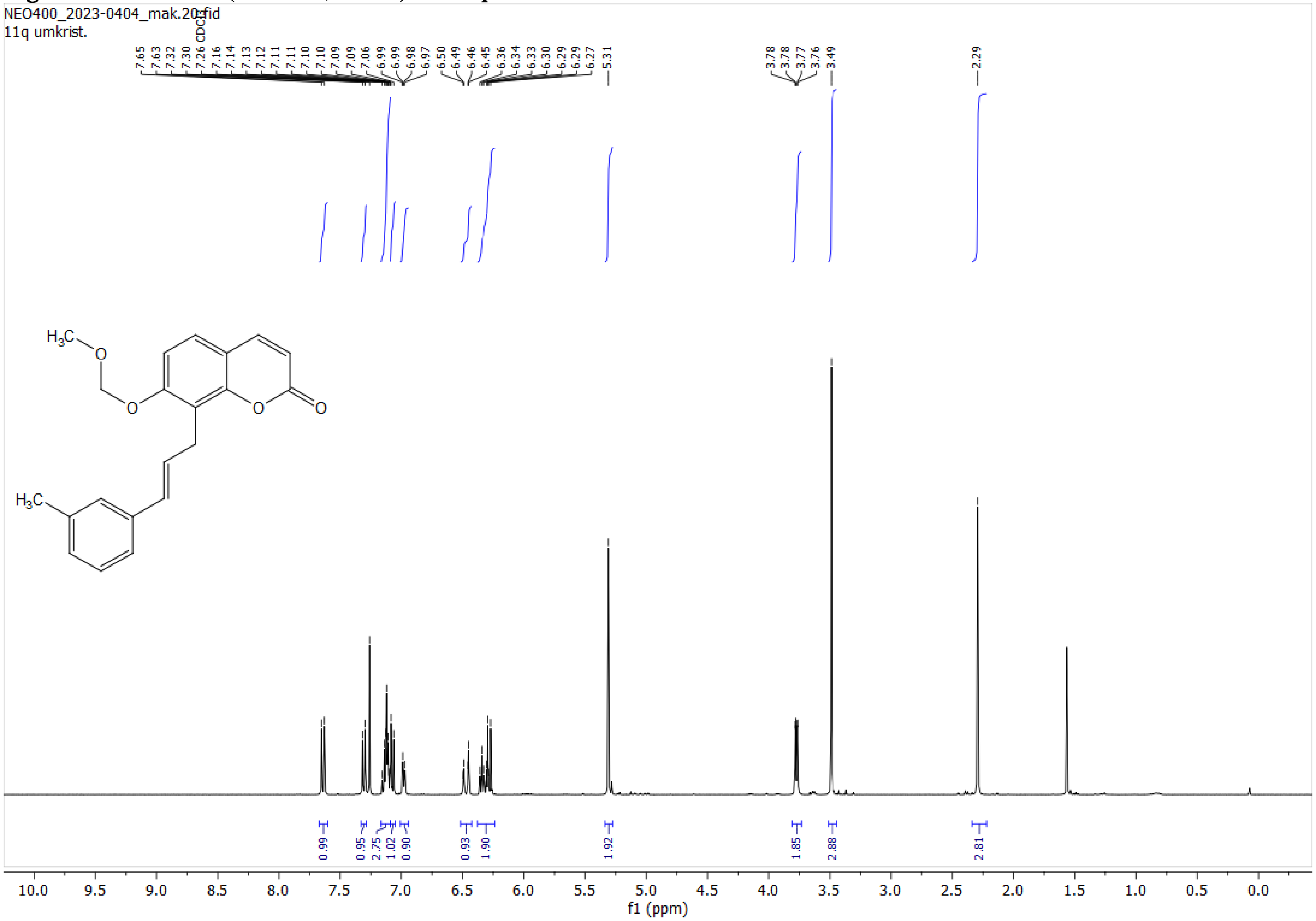

Figure S71: $^{13}C\{^{1}H\}$ NMR (100 MHz, $CDCl_3$) of **12aq**

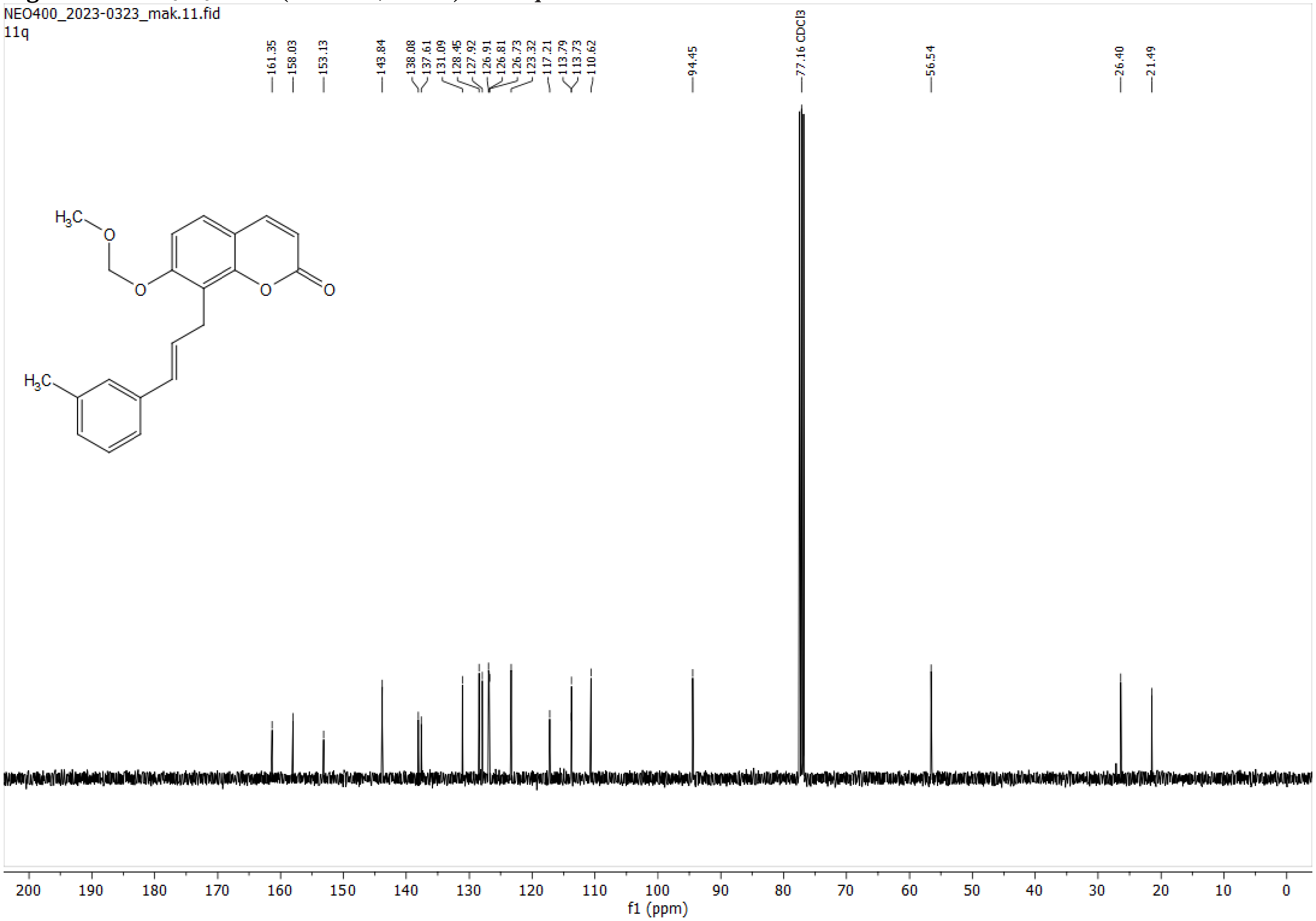

Figure S72: ^{1}H NMR (400 MHz, $CDCl_3$) of **12ar**

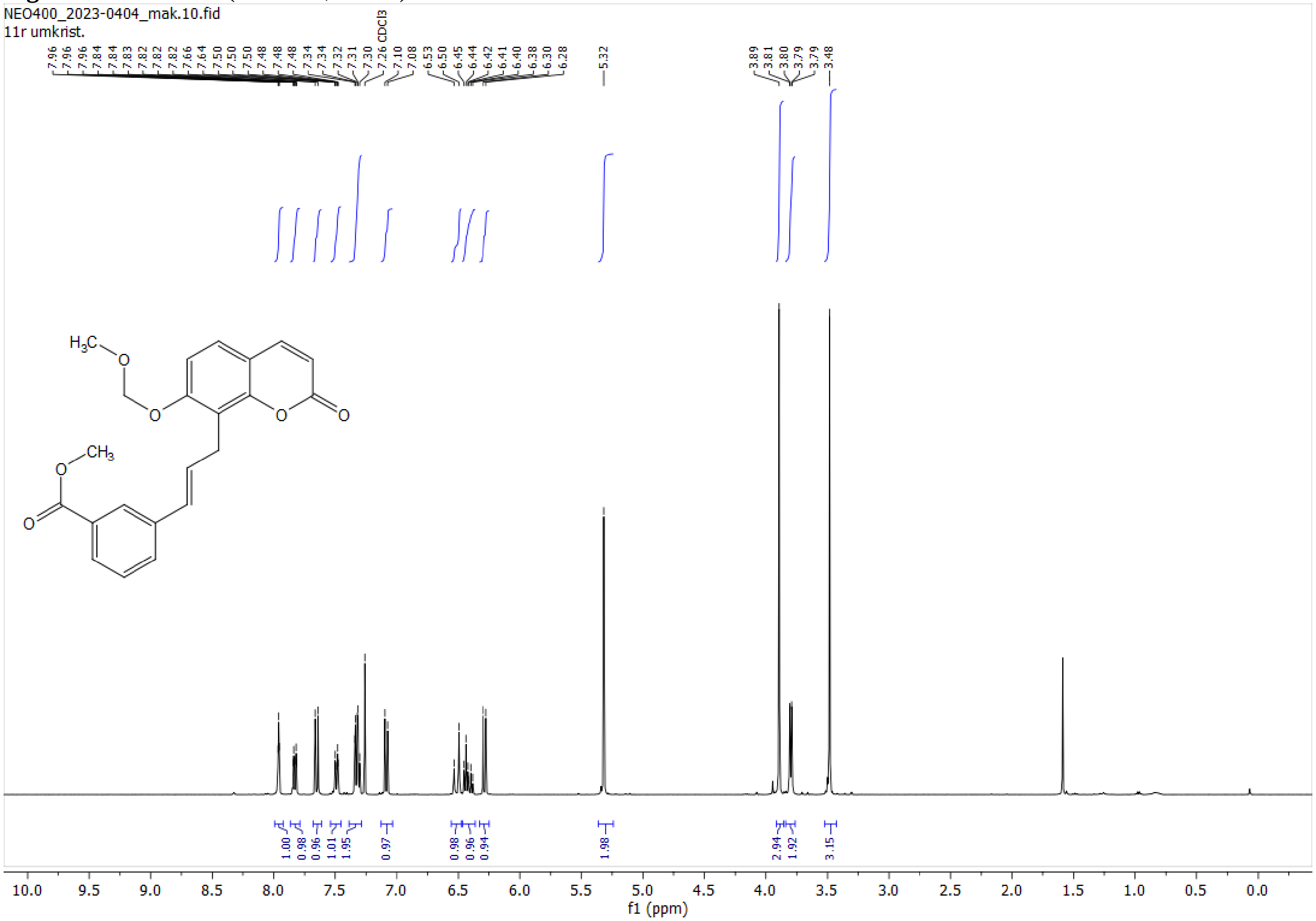

Figure S73: $^{13}C\{^{1}H\}$ NMR (100 MHz, $CDCl_3$) of **12ar**

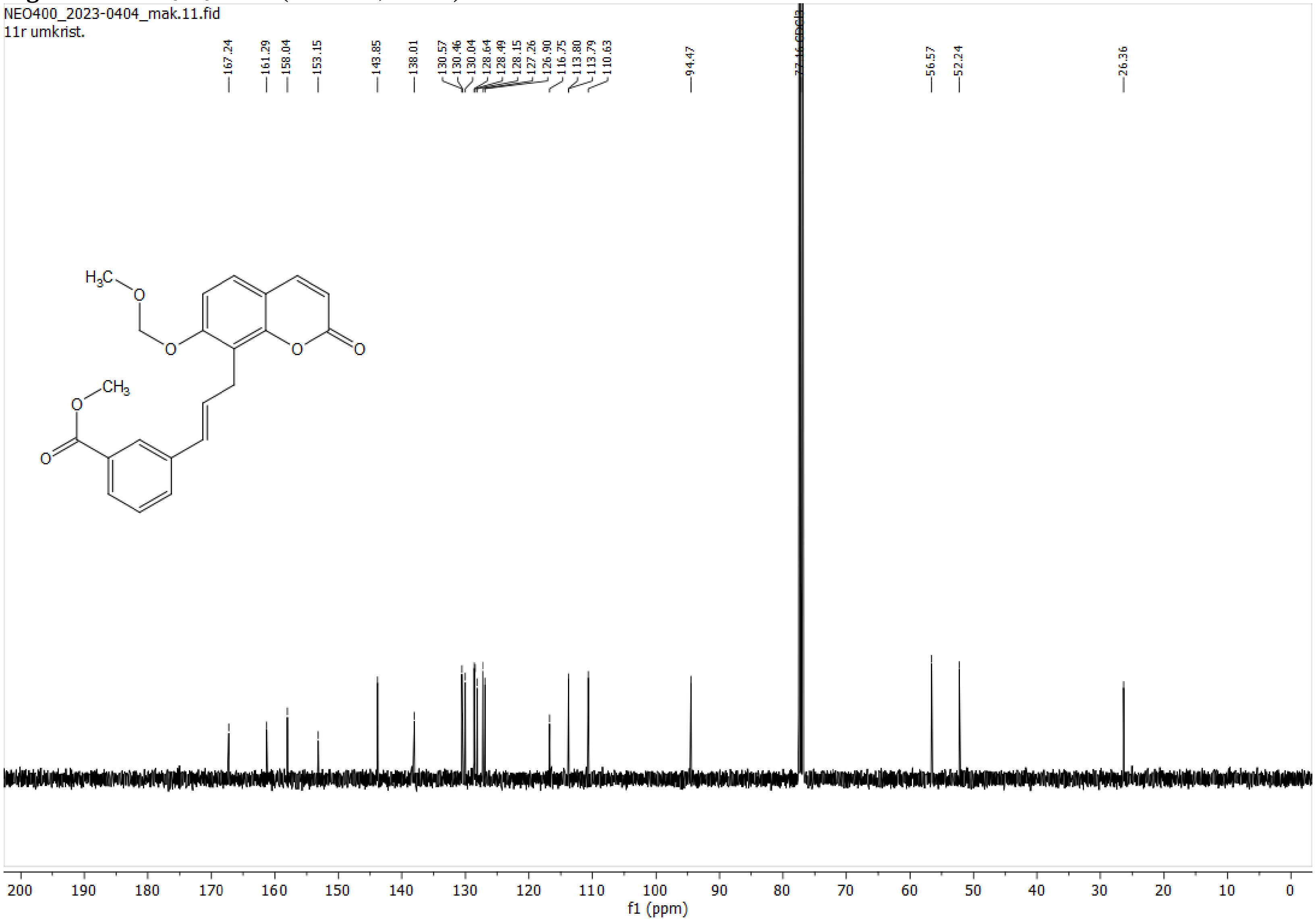

Figure S74: ^{1}H NMR (400 MHz, $CDCl_3$) of **12at**

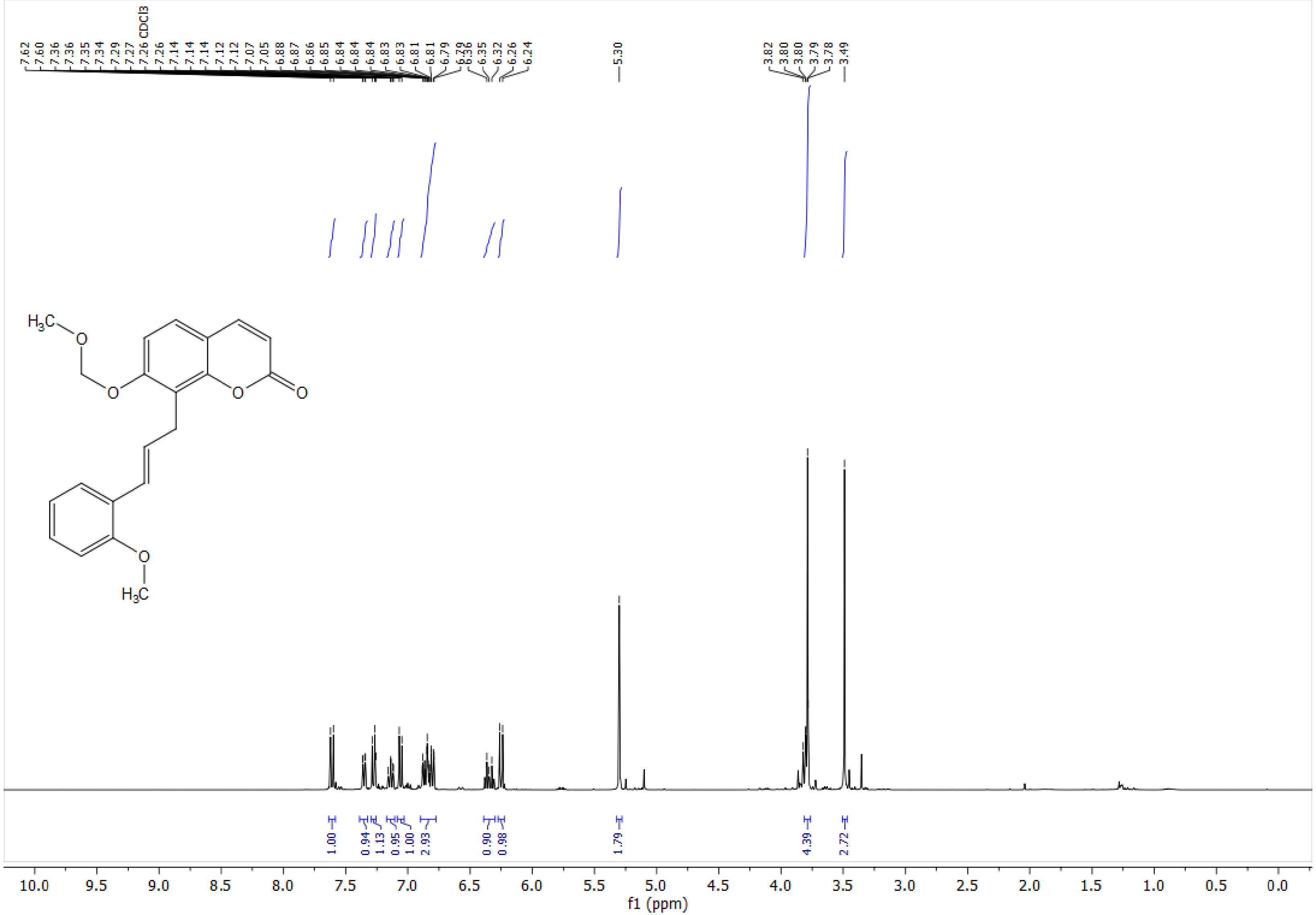

Figure S75: $^{13}C\{^1H\}$ NMR (100 MHz, $CDCl_3$) of **12at**

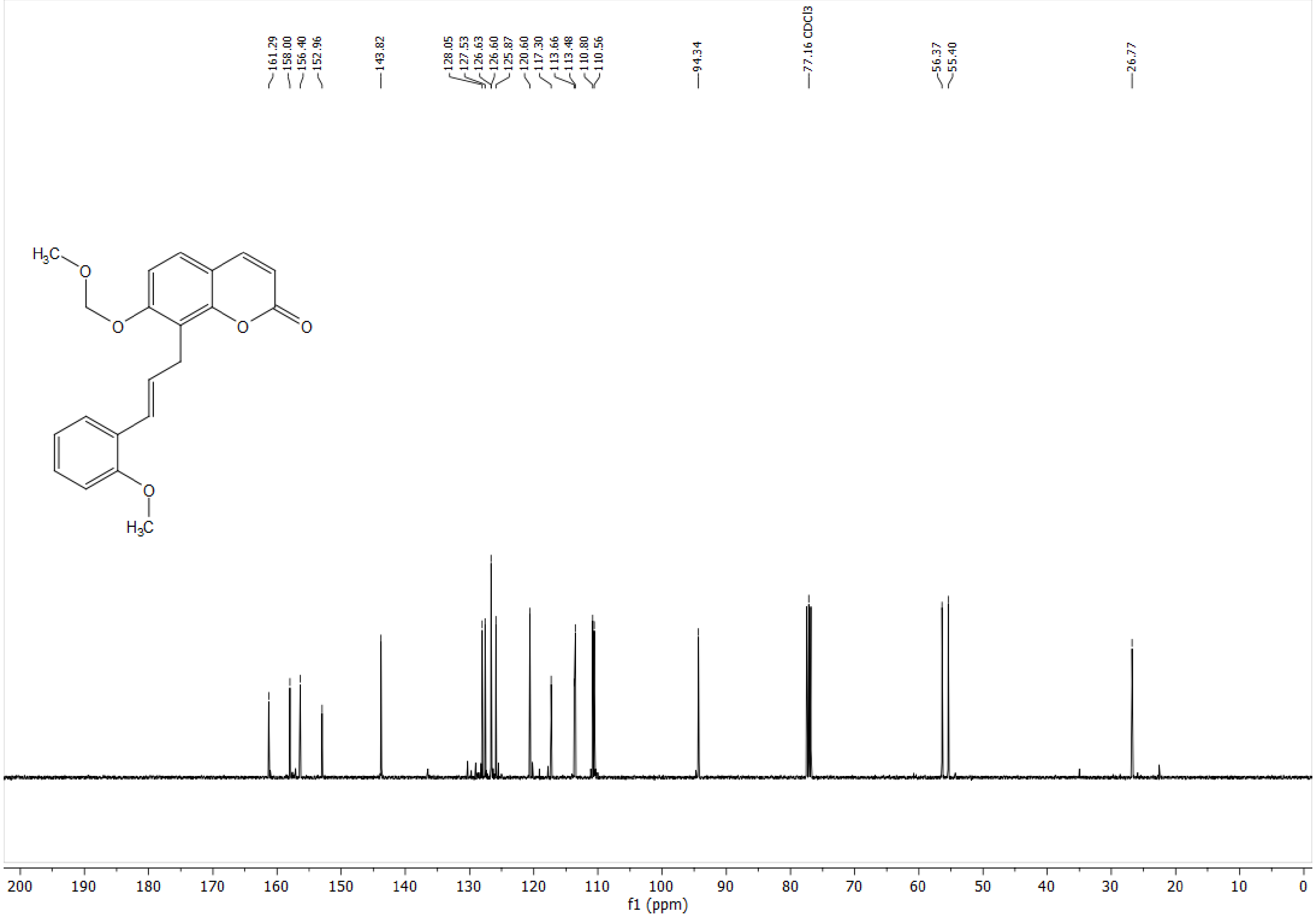

Figure S76: COSY (400 MHz, $CDCl_3$) of **12at**

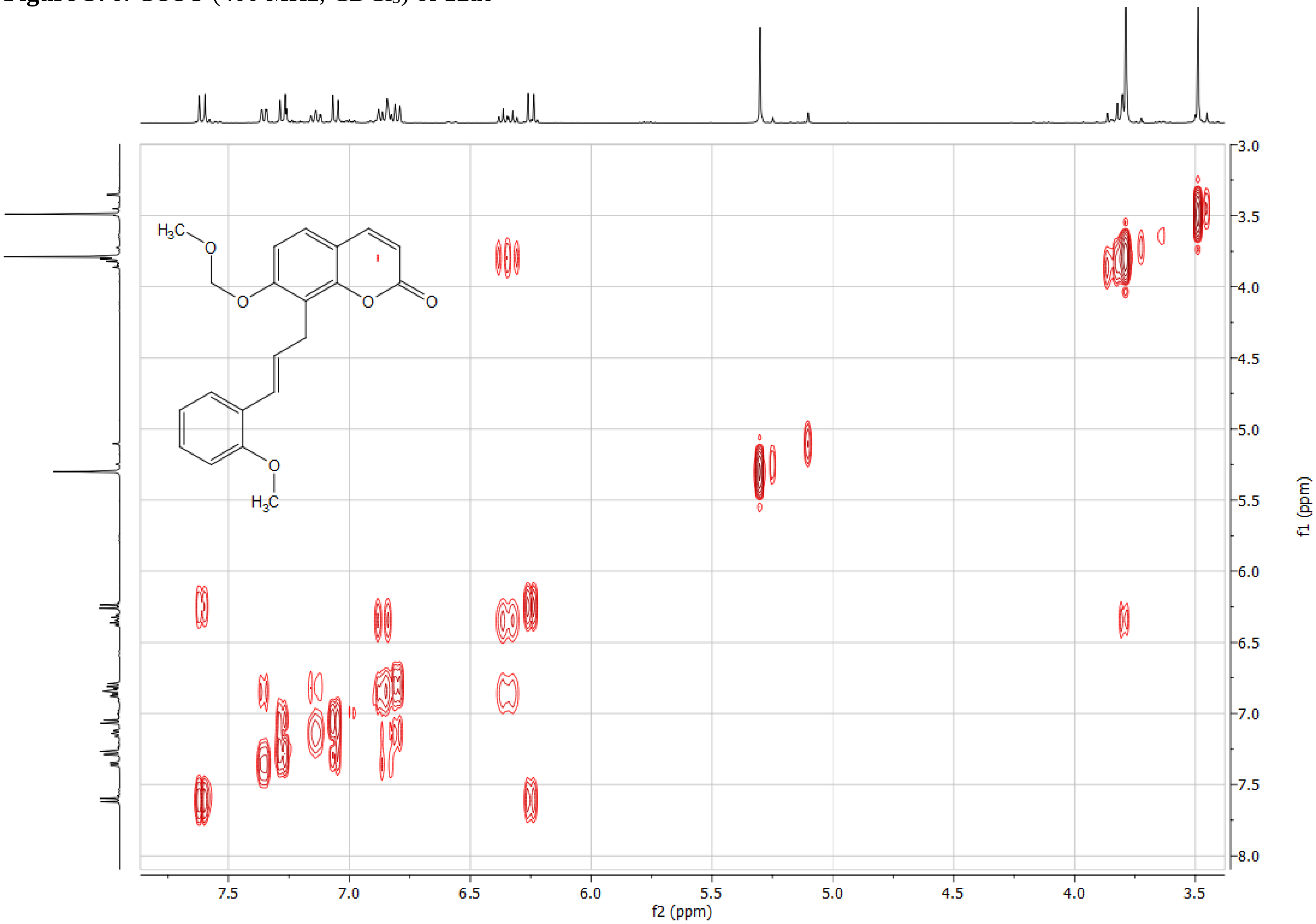

Figure S77: HSQC (400/100 MHz, $CDCl_3$) of **12at**

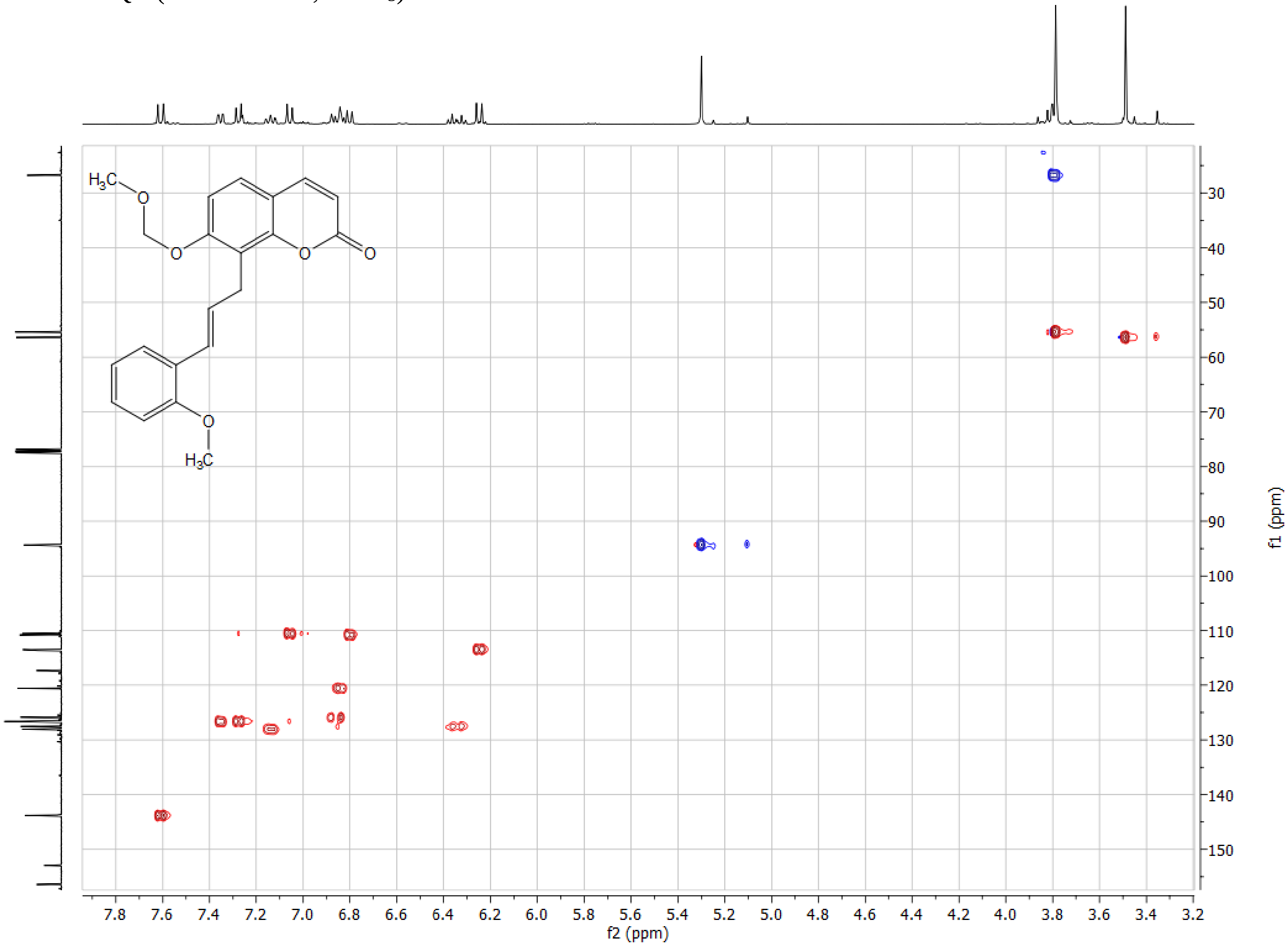

Figure S78: HMBC (400/100 MHz, $CDCl_3$) of **12at**

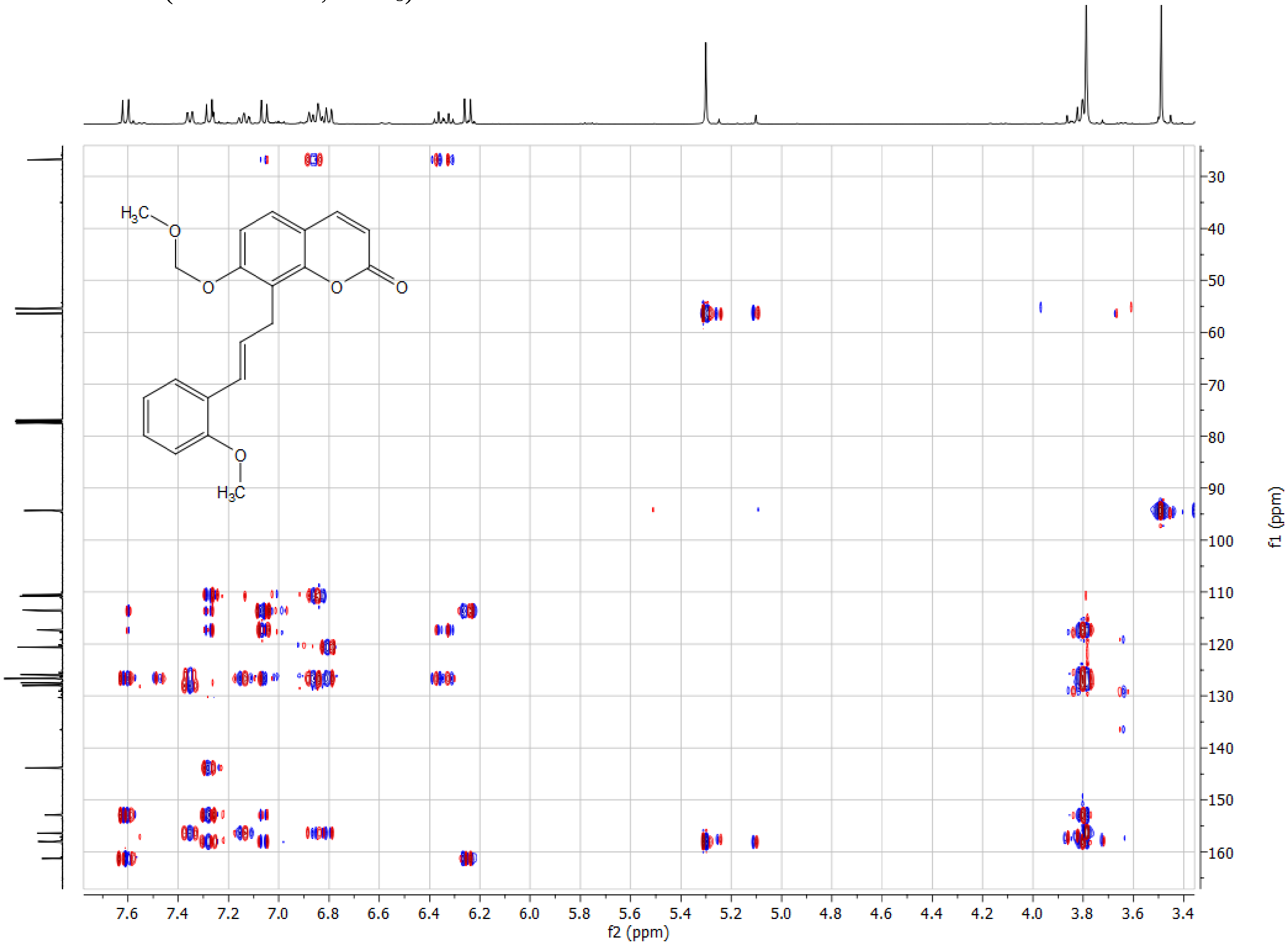

Figure S79: NOESY (400 MHz, $CDCl_3$) of **12at**

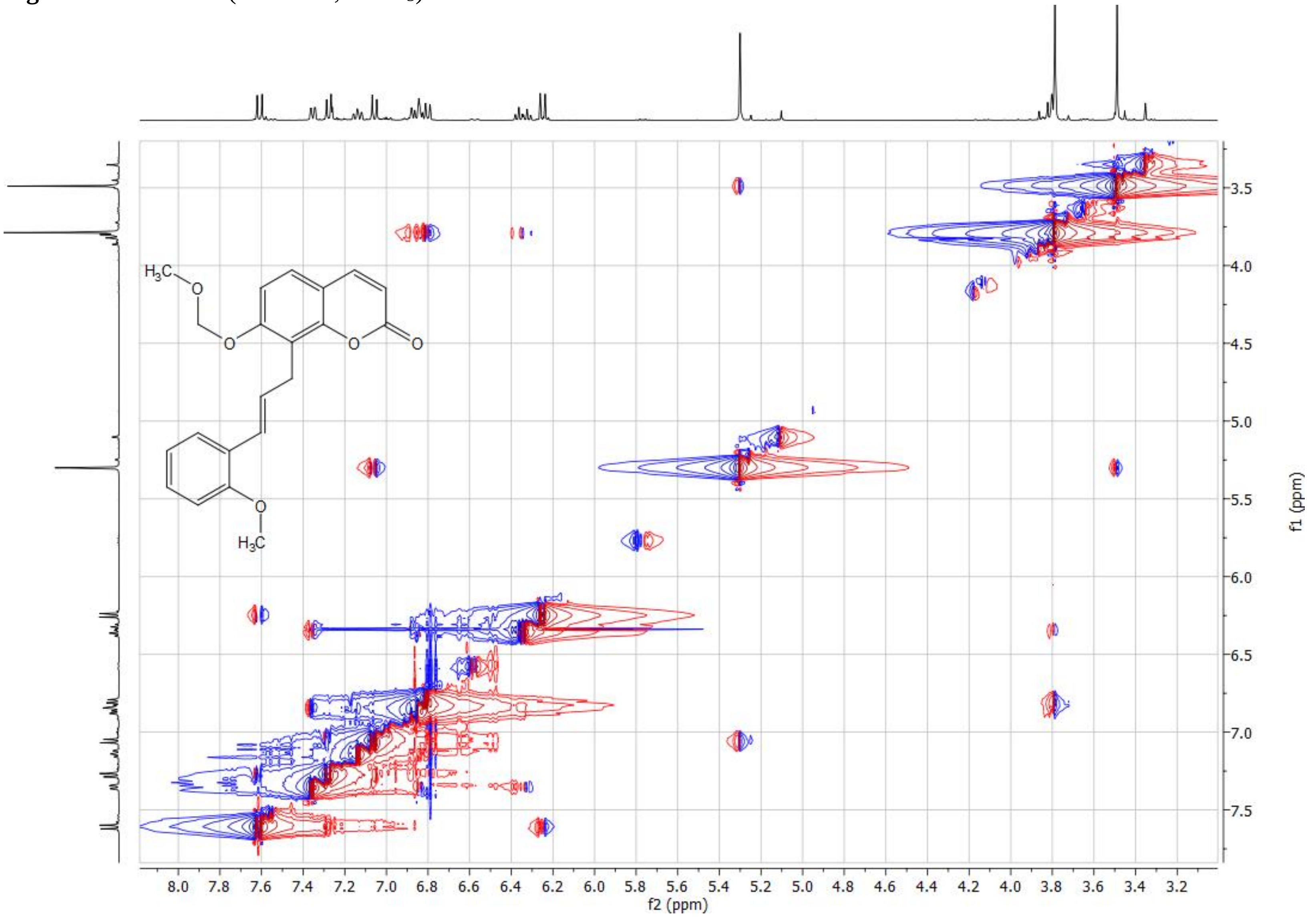

Figure S80: ^{1}H NMR (400 MHz, $CDCl_3$) of **12au**

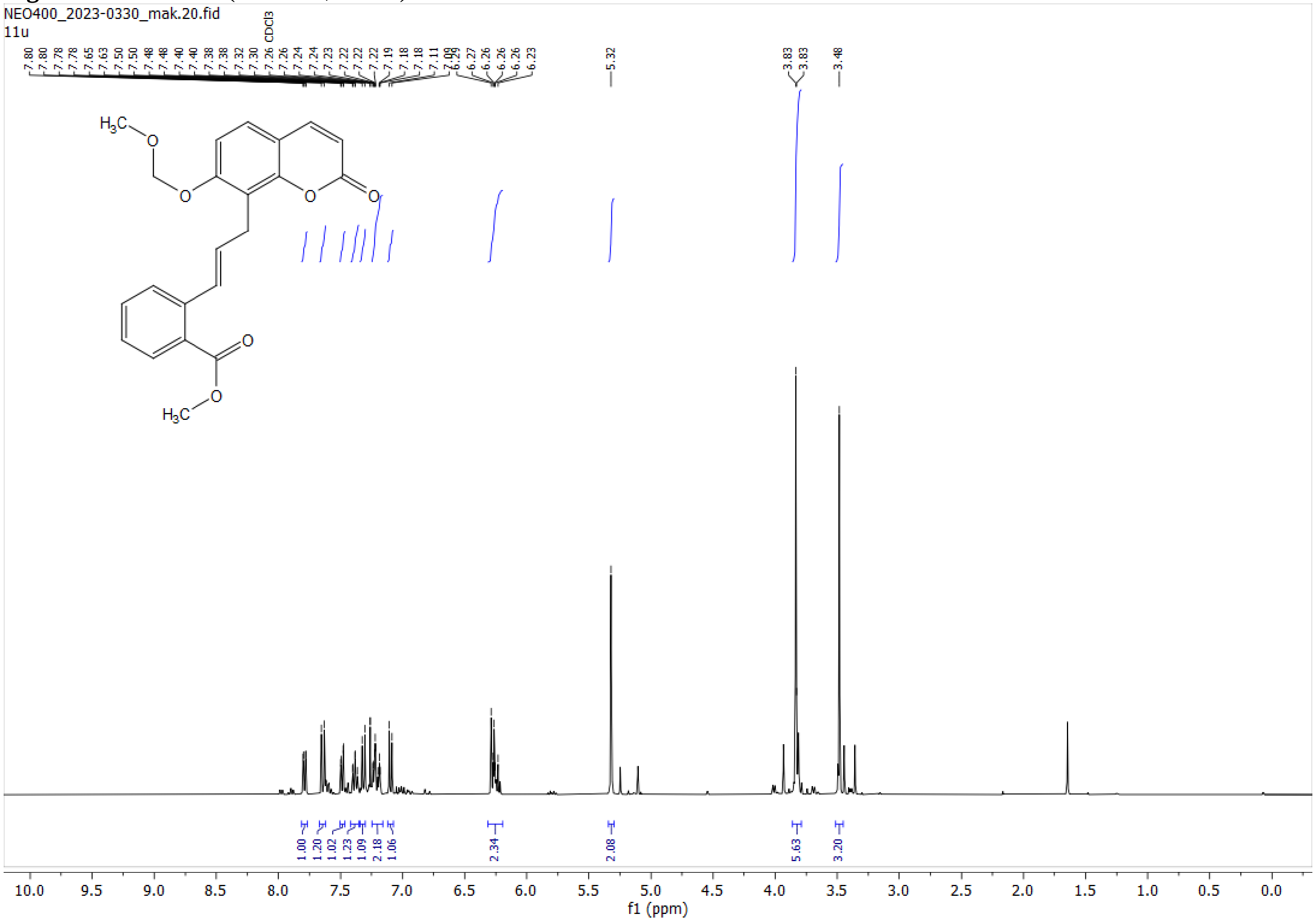

Figure S81: $^{13}C\{^{1}H\}$ NMR (100 MHz, $CDCl_3$) of **12au**

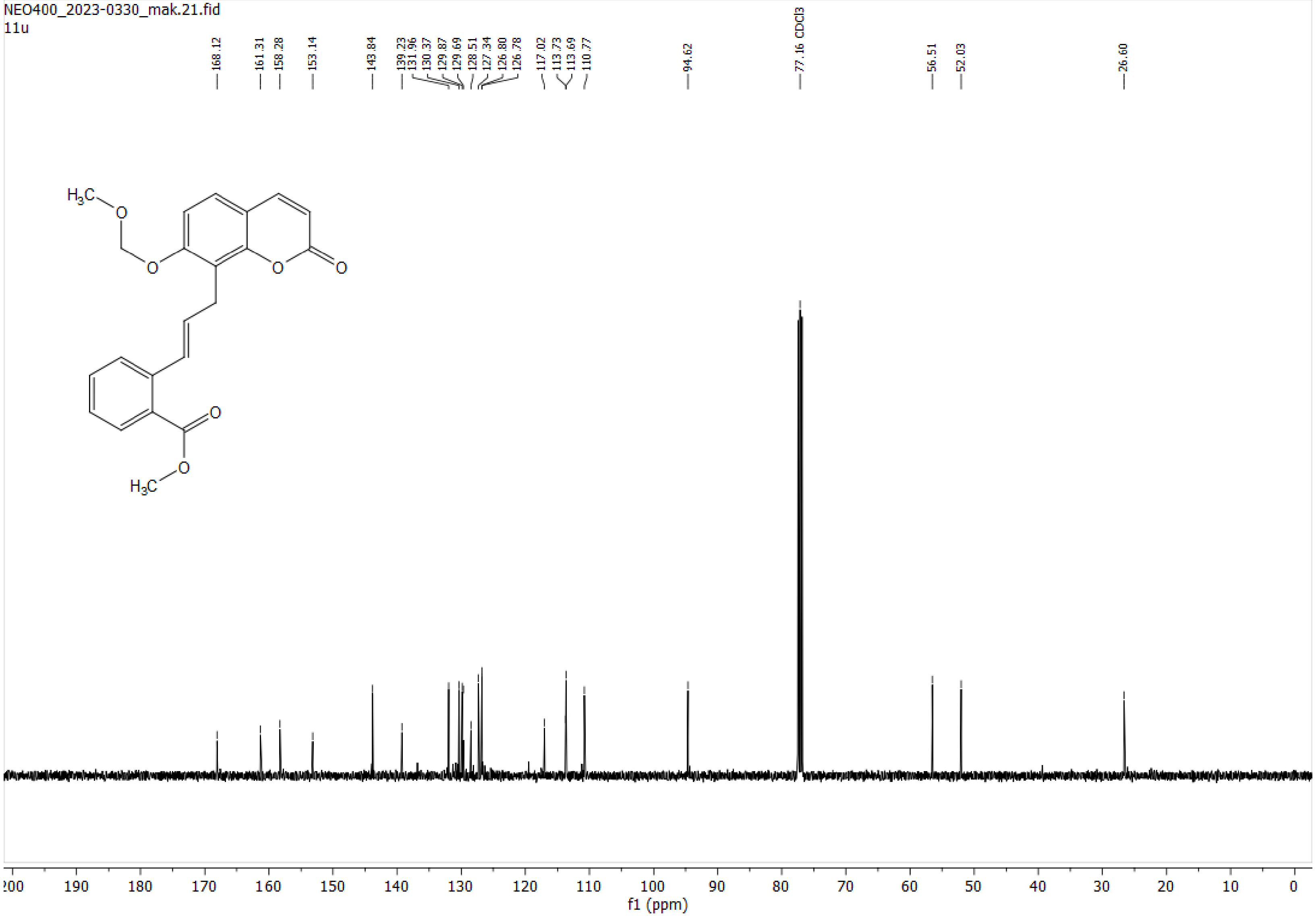

Figure S82: ^{1}H NMR (400 MHz, $CDCl_3$) of **13aa**

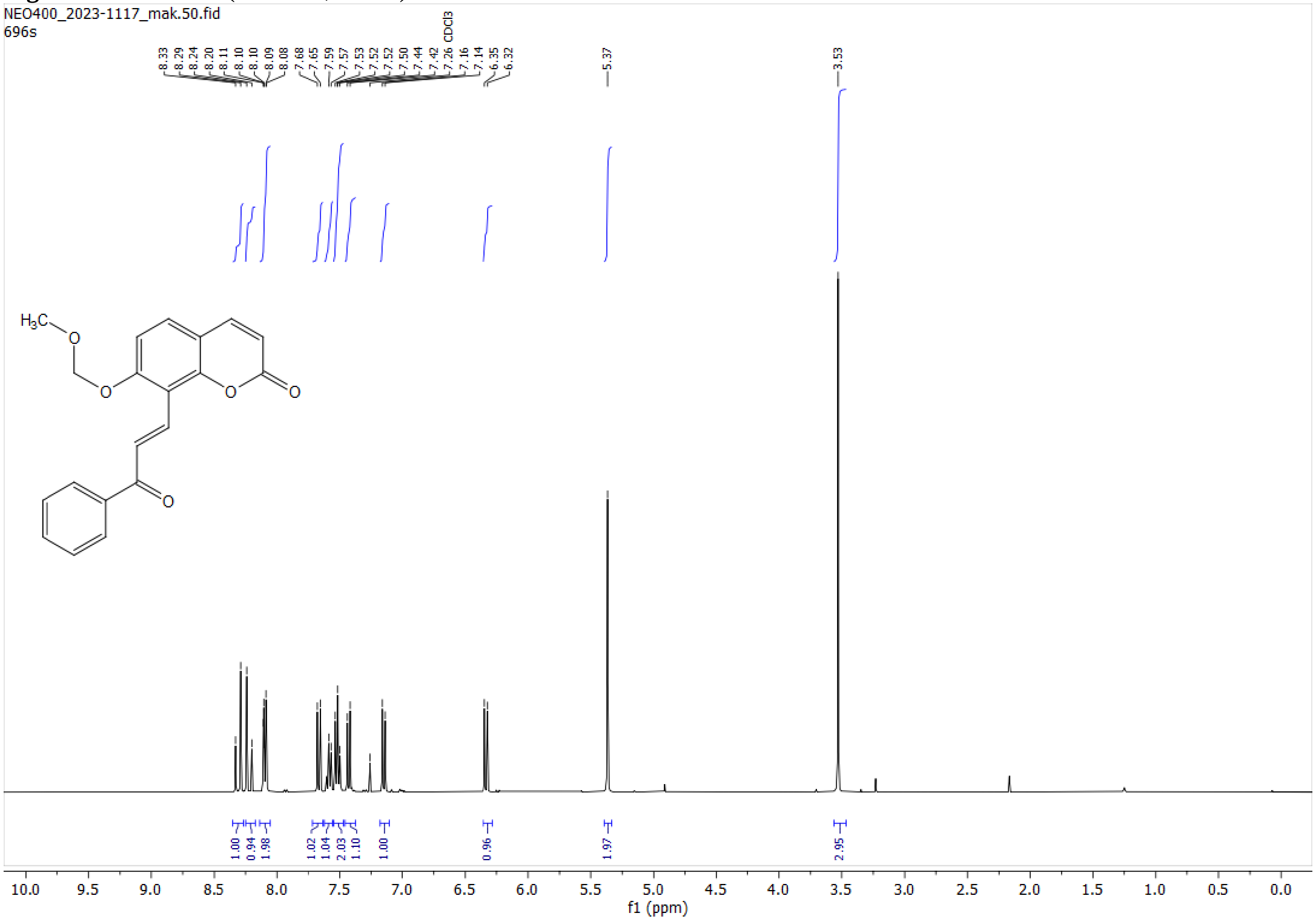

Figure S83: $^{13}C\{^{1}H\}$ NMR (100 MHz, $CDCl_3$) of **13aa**

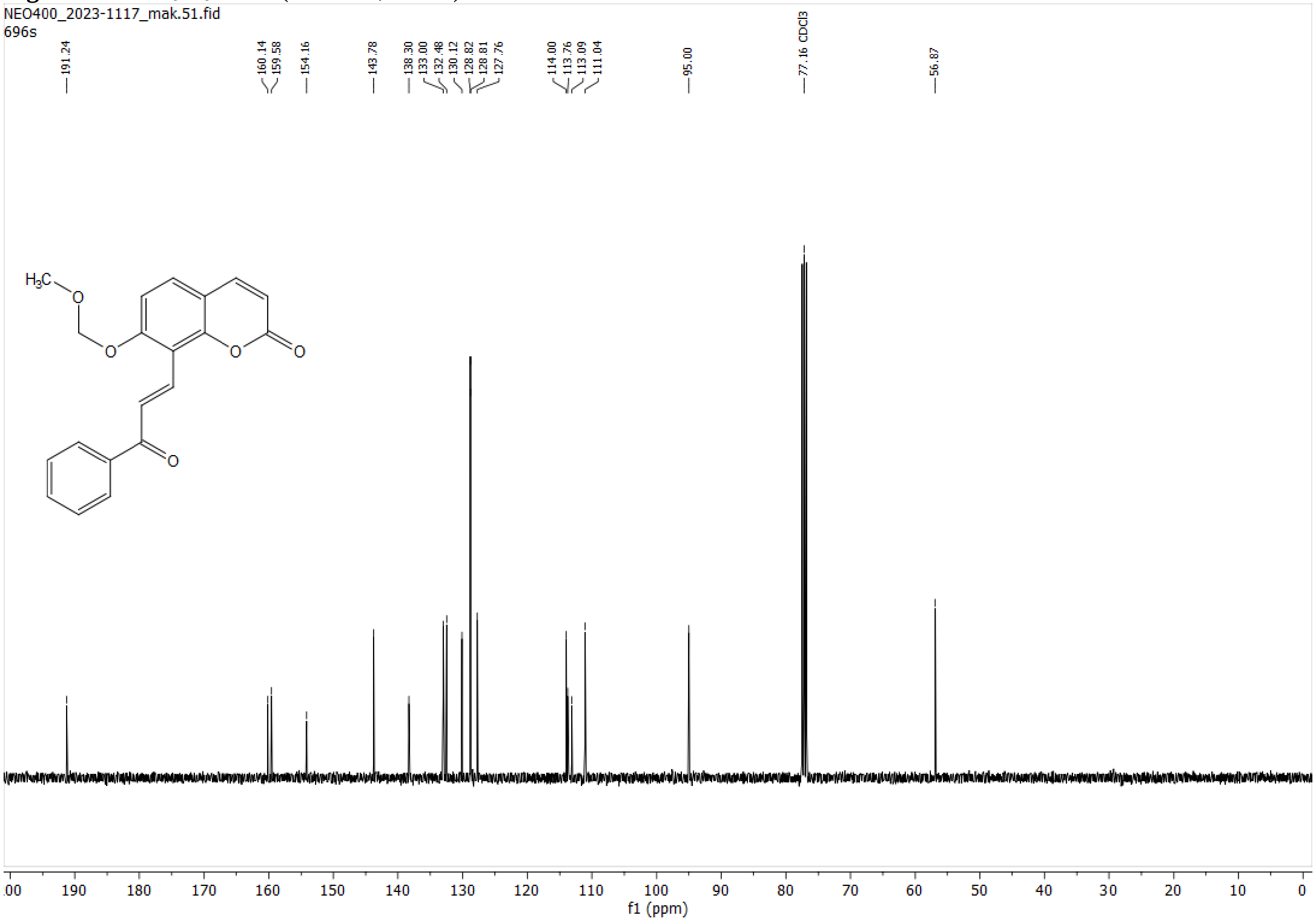

Figure S84: COSY (400 MHz, $CDCl_3$) of **13aa**

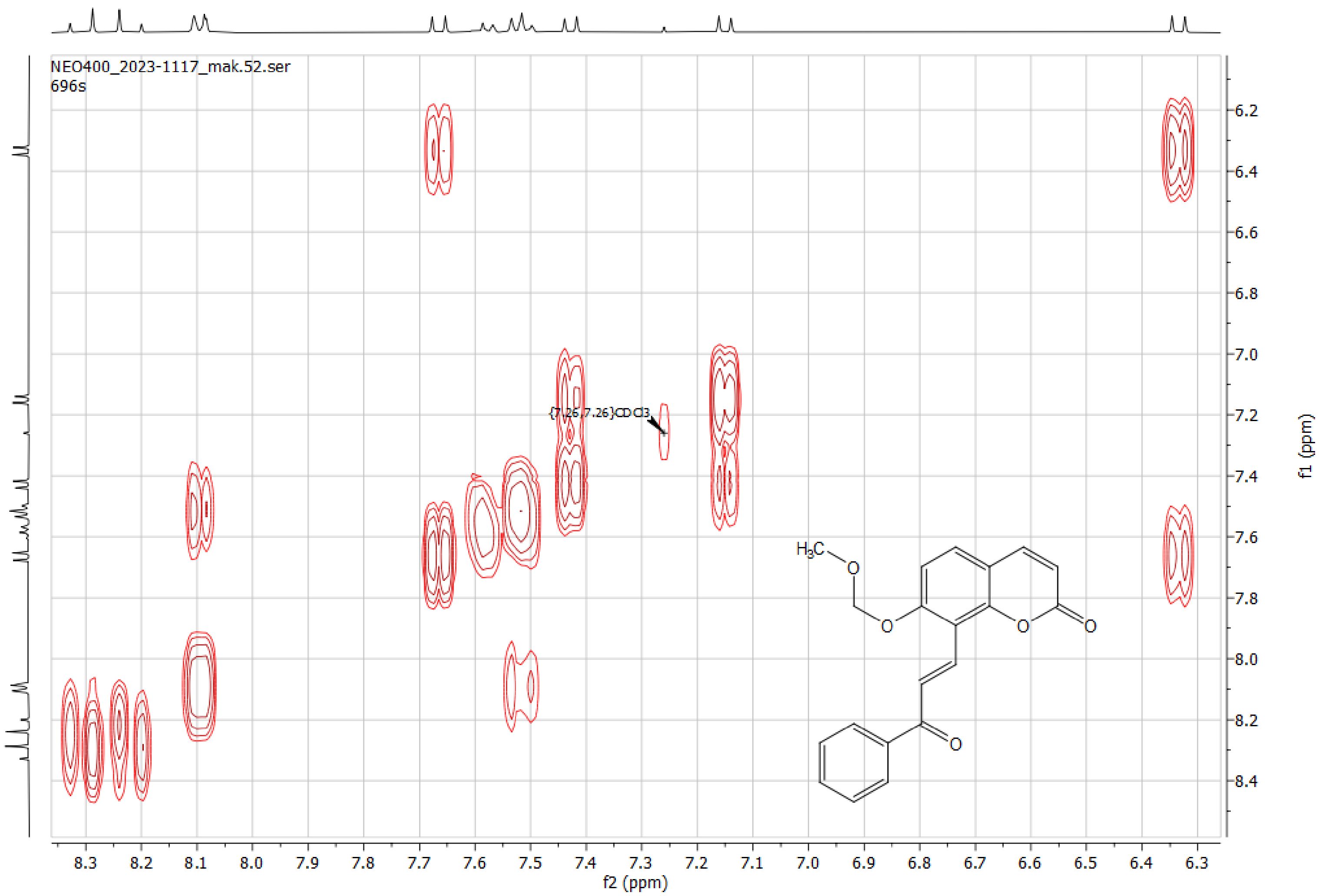

Figure S85: HSQC (400/100 MHz, $CDCl_3$) of **13aa**

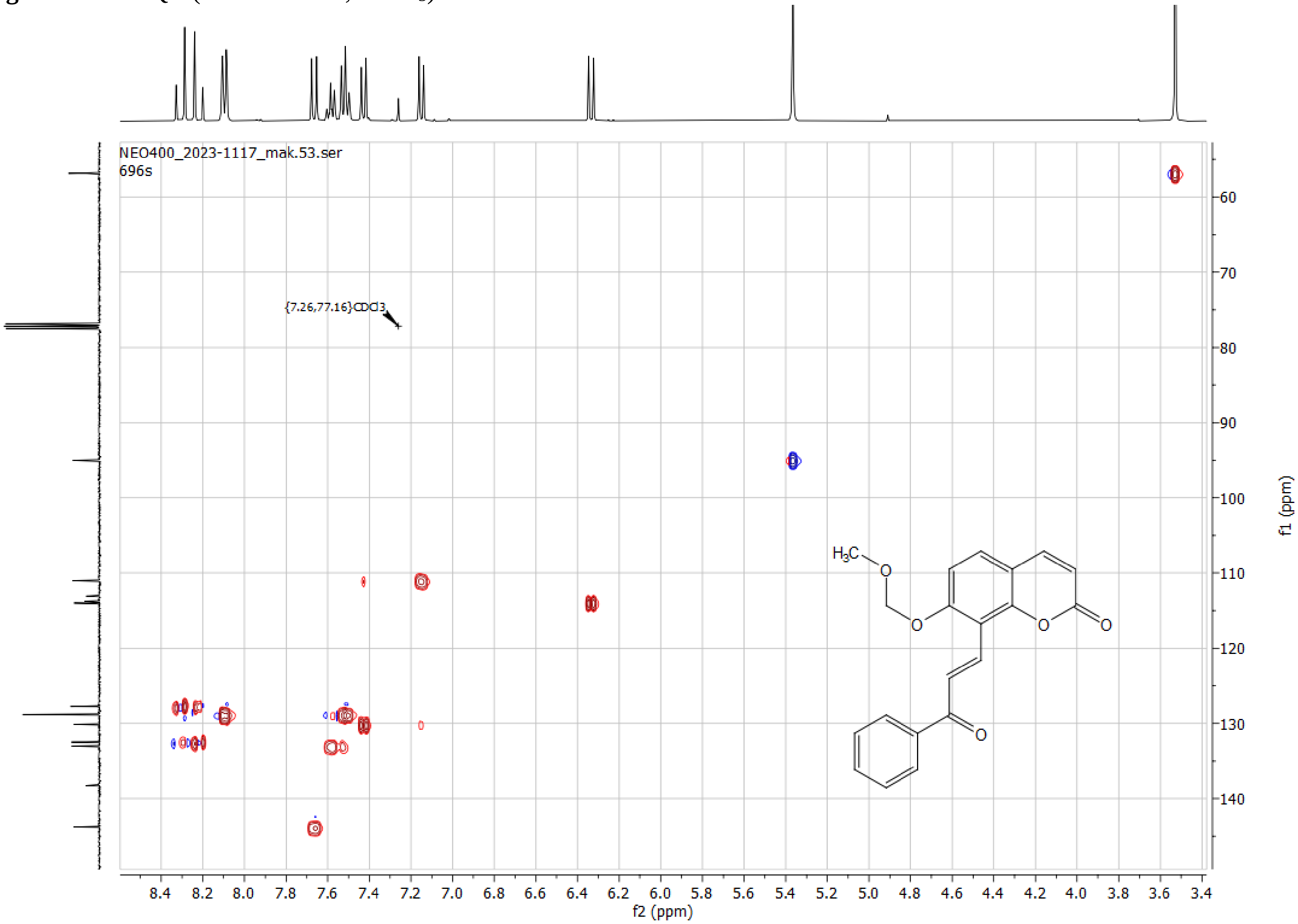

Figure S86: HMBC (400/100 MHz, $CDCl_3$) of **13aa**

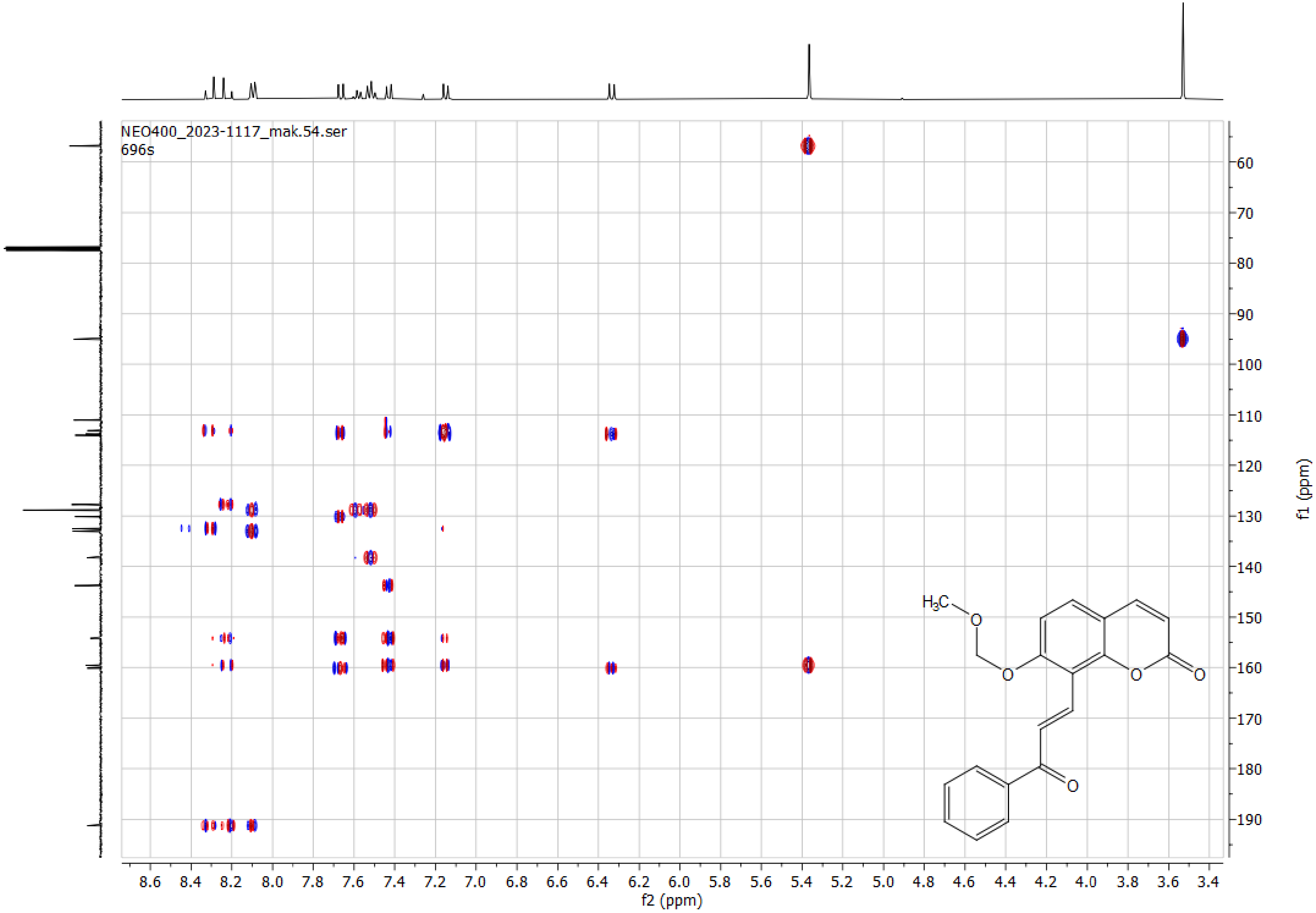

Figure S87: NOESY (400 MHz, $CDCl_3$) of **13aa**

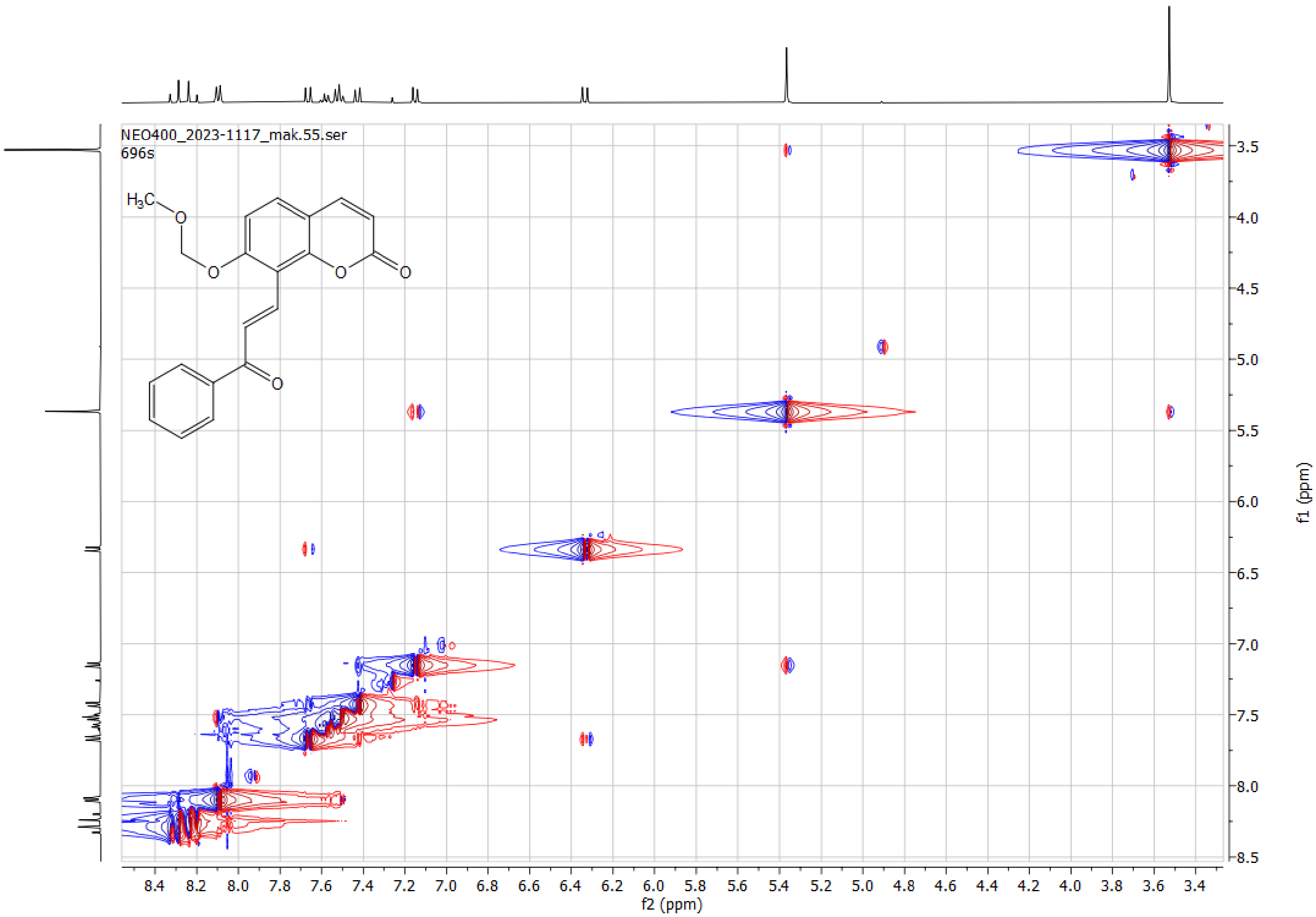

Figure S88: ^{1}H NMR (400 MHz, $CDCl_3$) of **13ab**

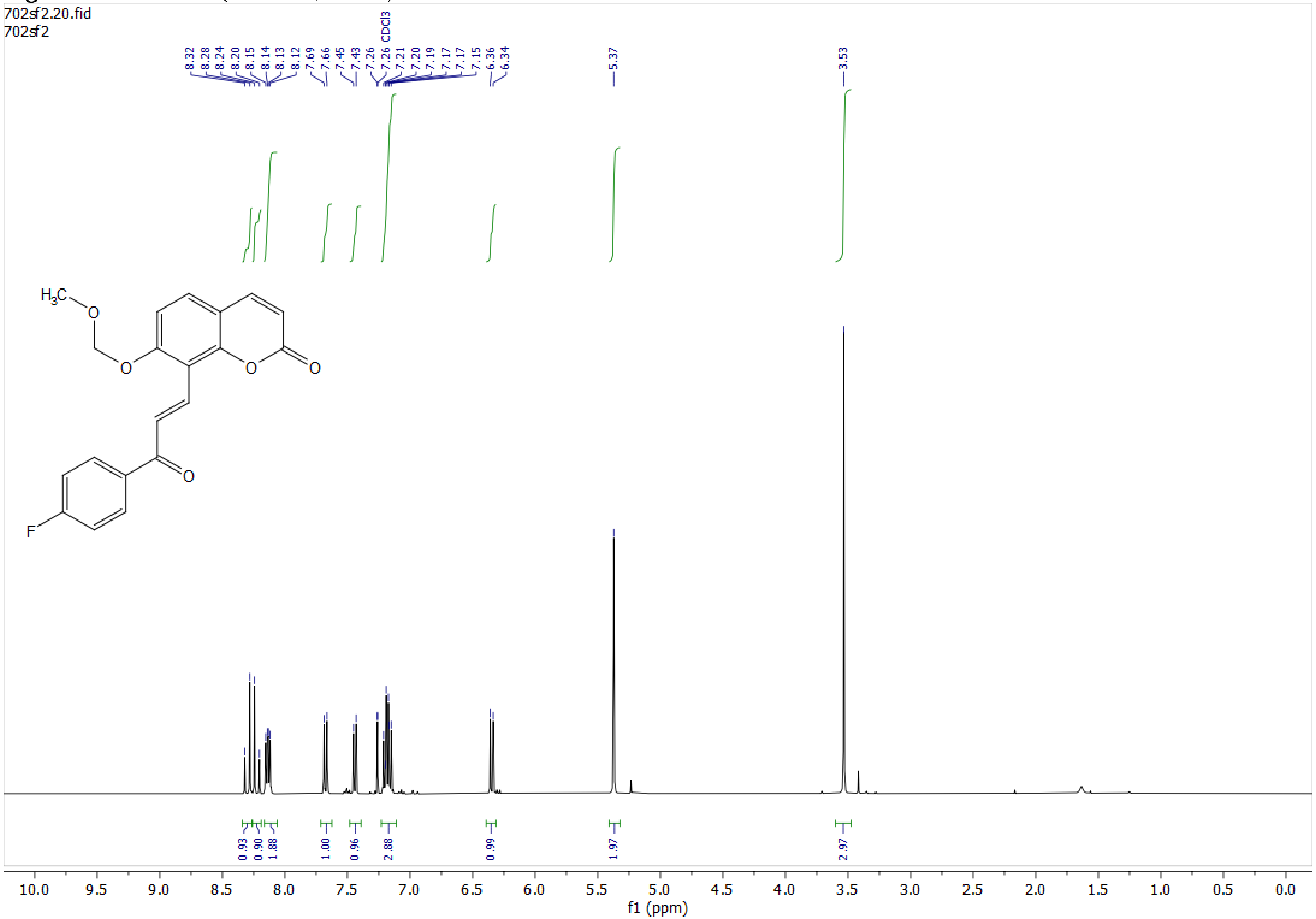

Figure S89: $^{13}C\{^{1}H\}$ NMR (100 MHz, $CDCl_3$) of **13ab**

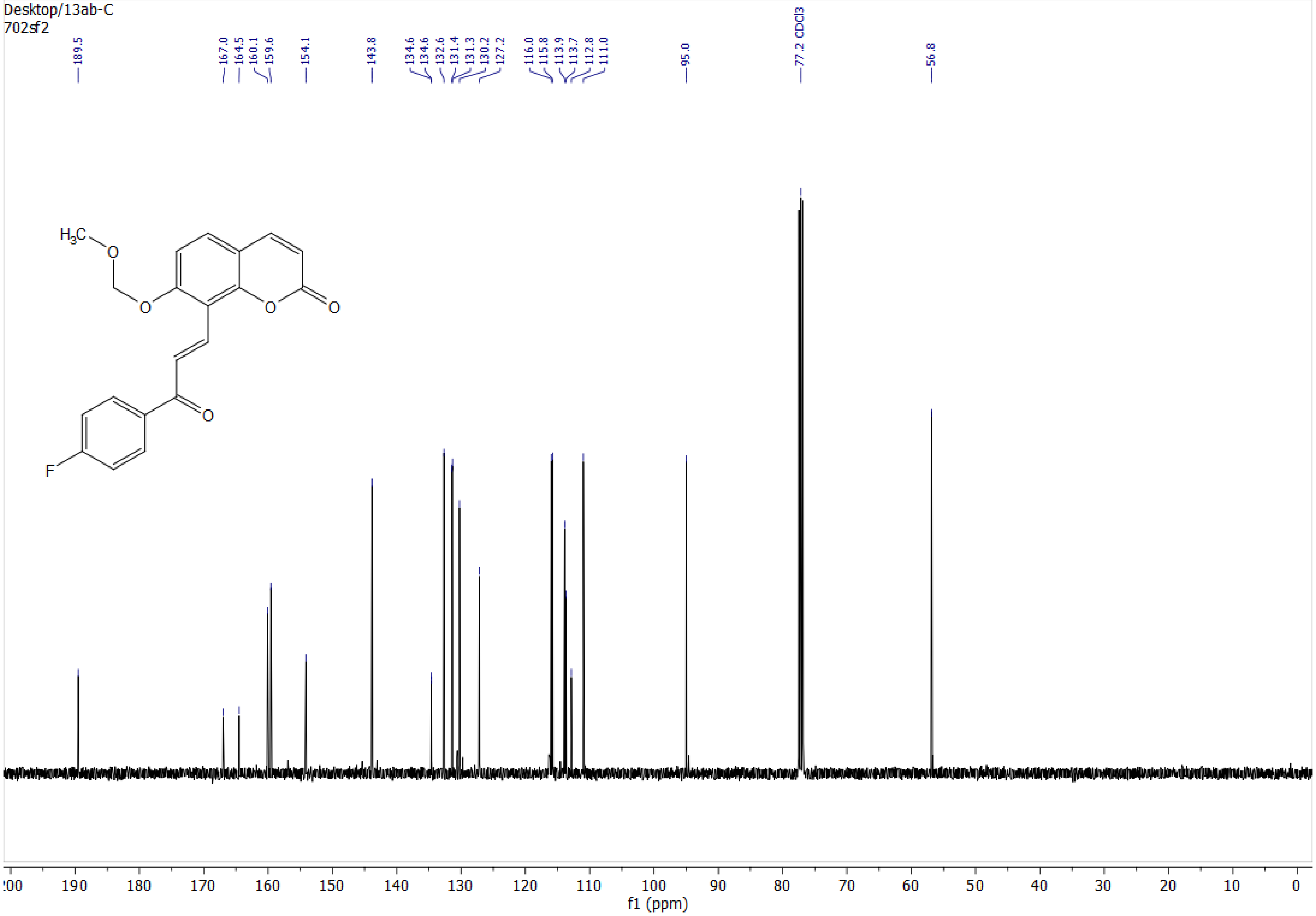

Figure S90: ^{1}H NMR (400 MHz, $CDCl_3$) of **13ac**

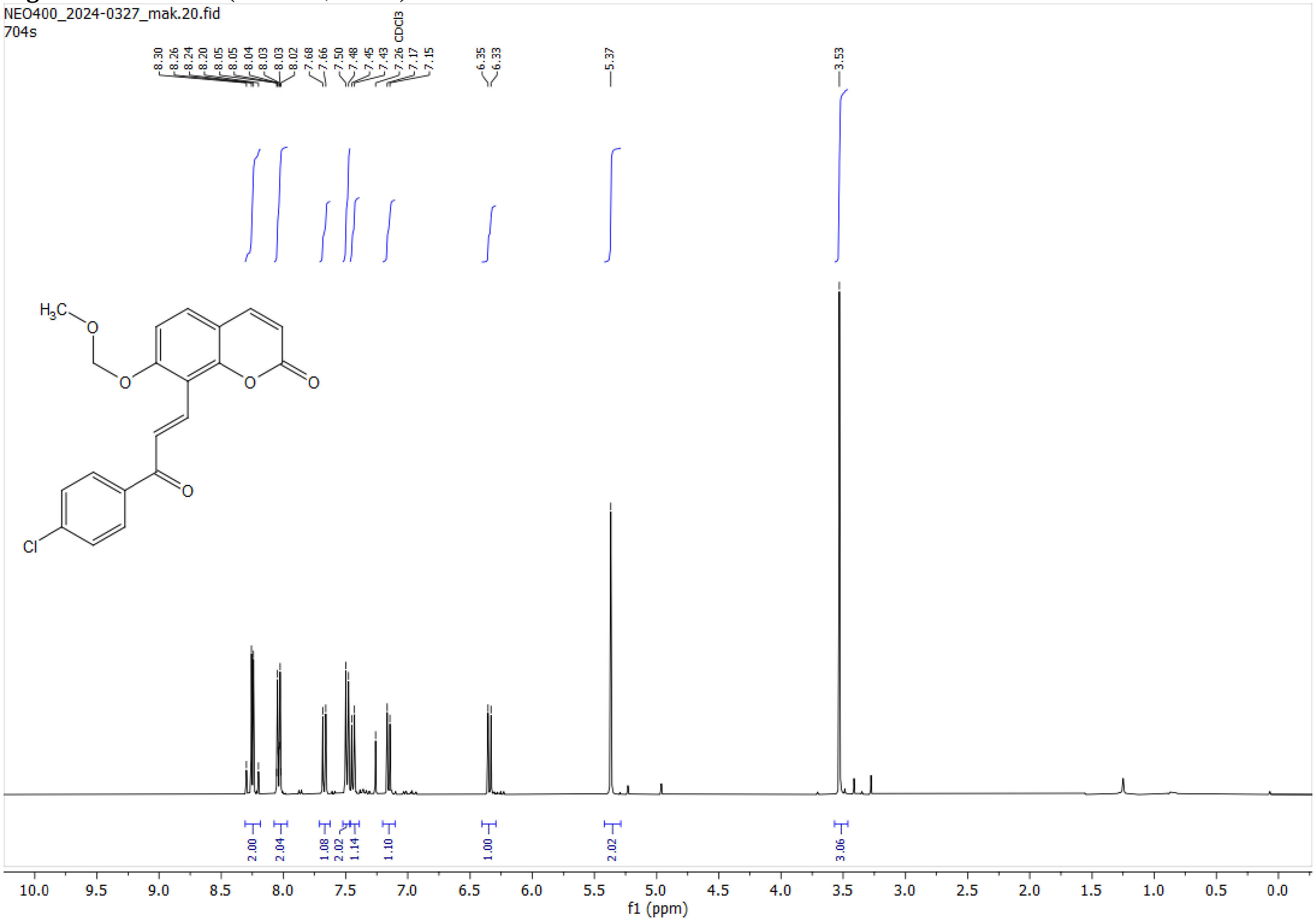

Figure S91: $^{13}C\{^{1}H\}$ NMR (100 MHz, $CDCl_3$) of **13ac**

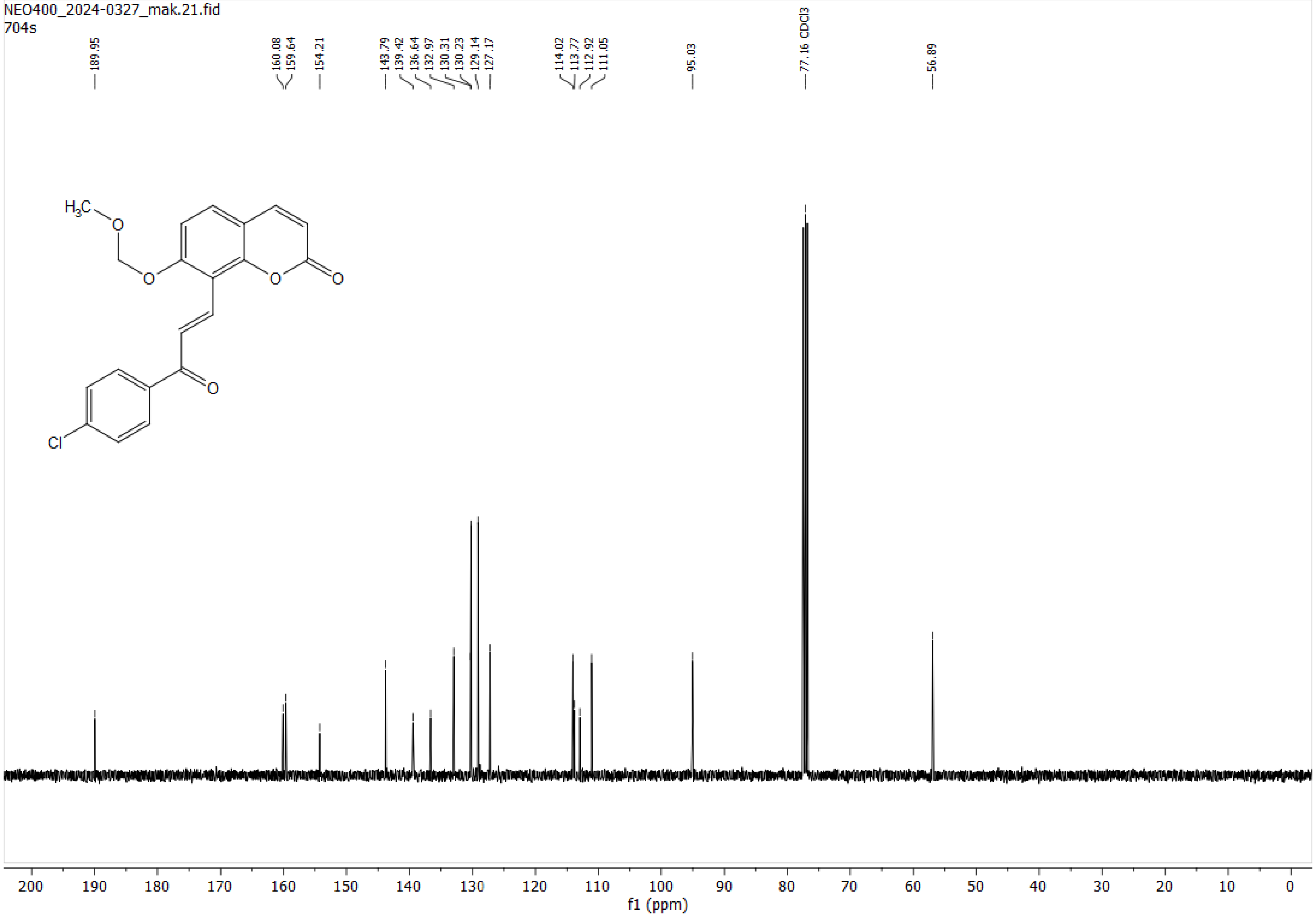

Figure S92: ^{1}H NMR (400 MHz, $CDCl_3$) of **13ad**

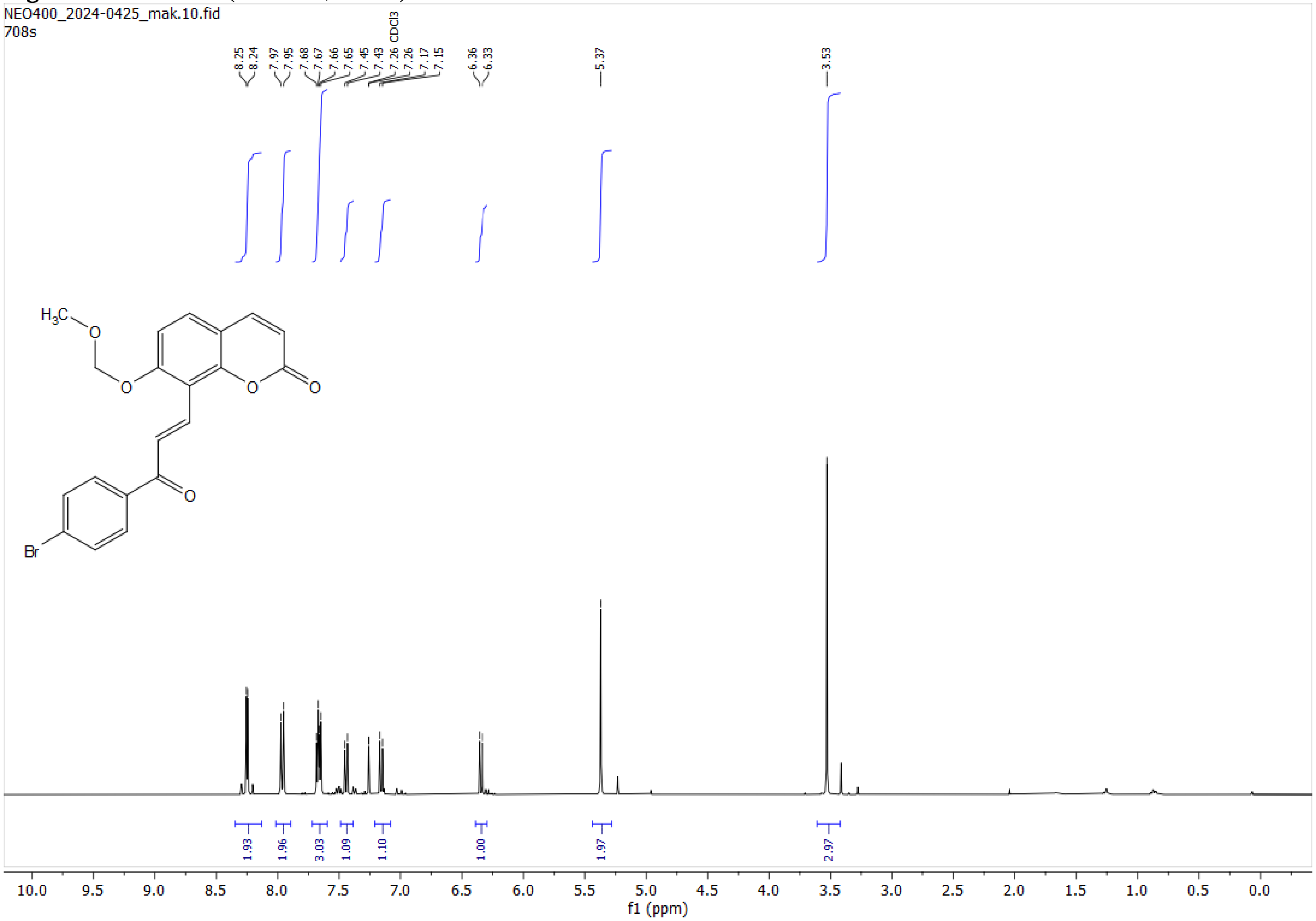

Figure S93: $^{13}C\{^1H\}$ NMR (100 MHz, $CDCl_3$) of **13ad**

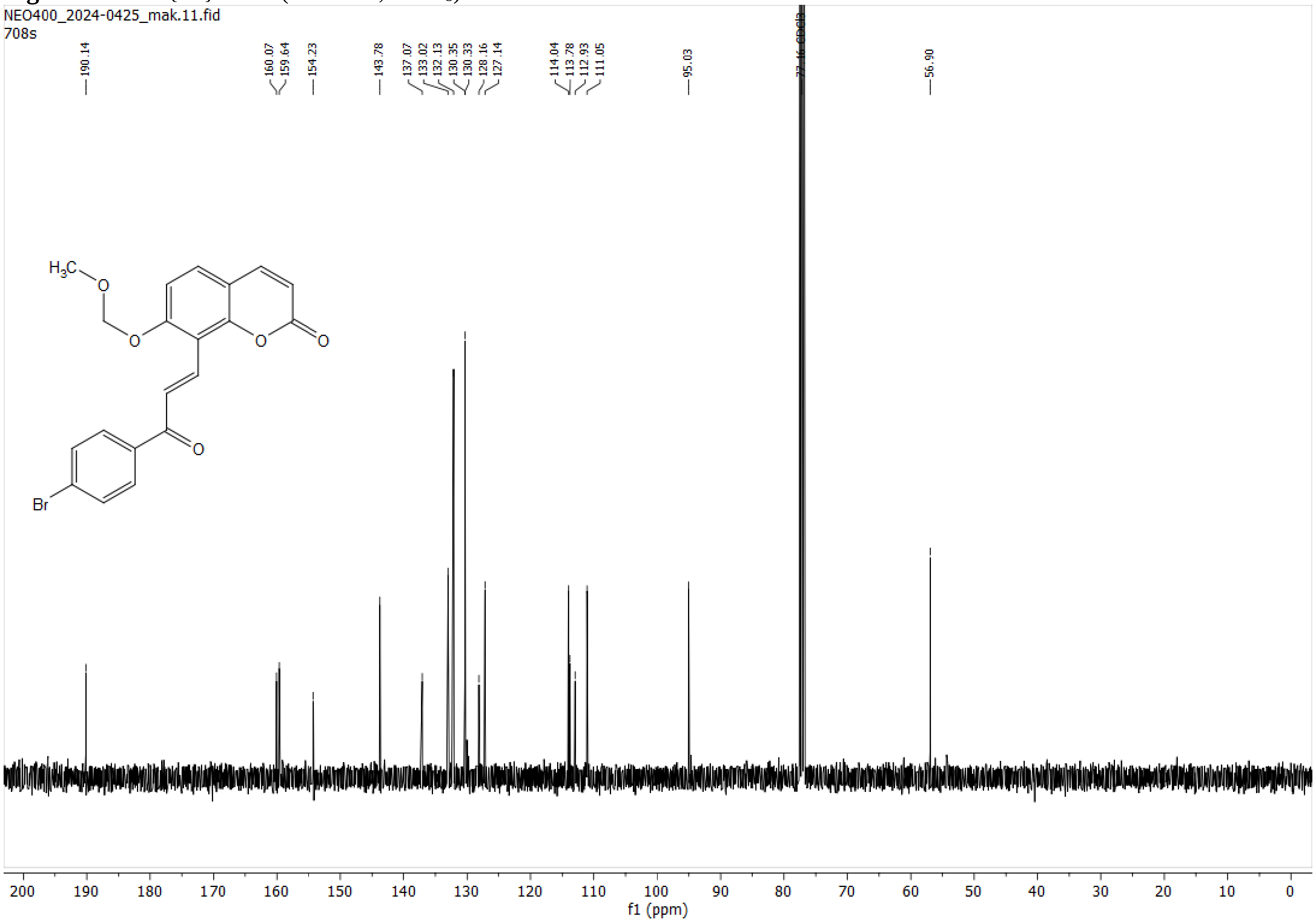

Figure S94: ^{1}H NMR (400 MHz, $CDCl_3$) of **13ae**

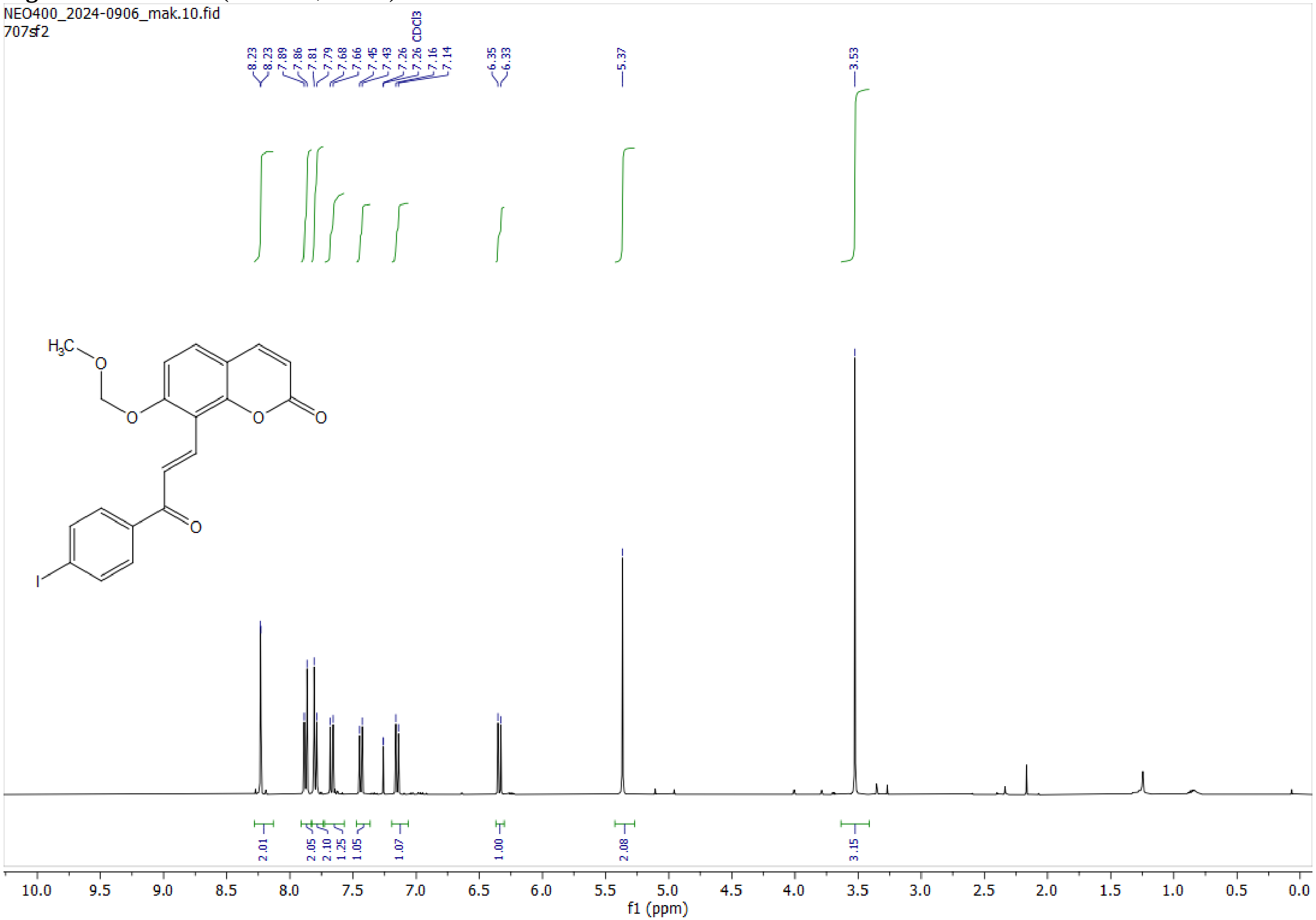

Figure S95: $^{13}C\{^1H\}$ NMR (100 MHz, $CDCl_3$) of **13ae**

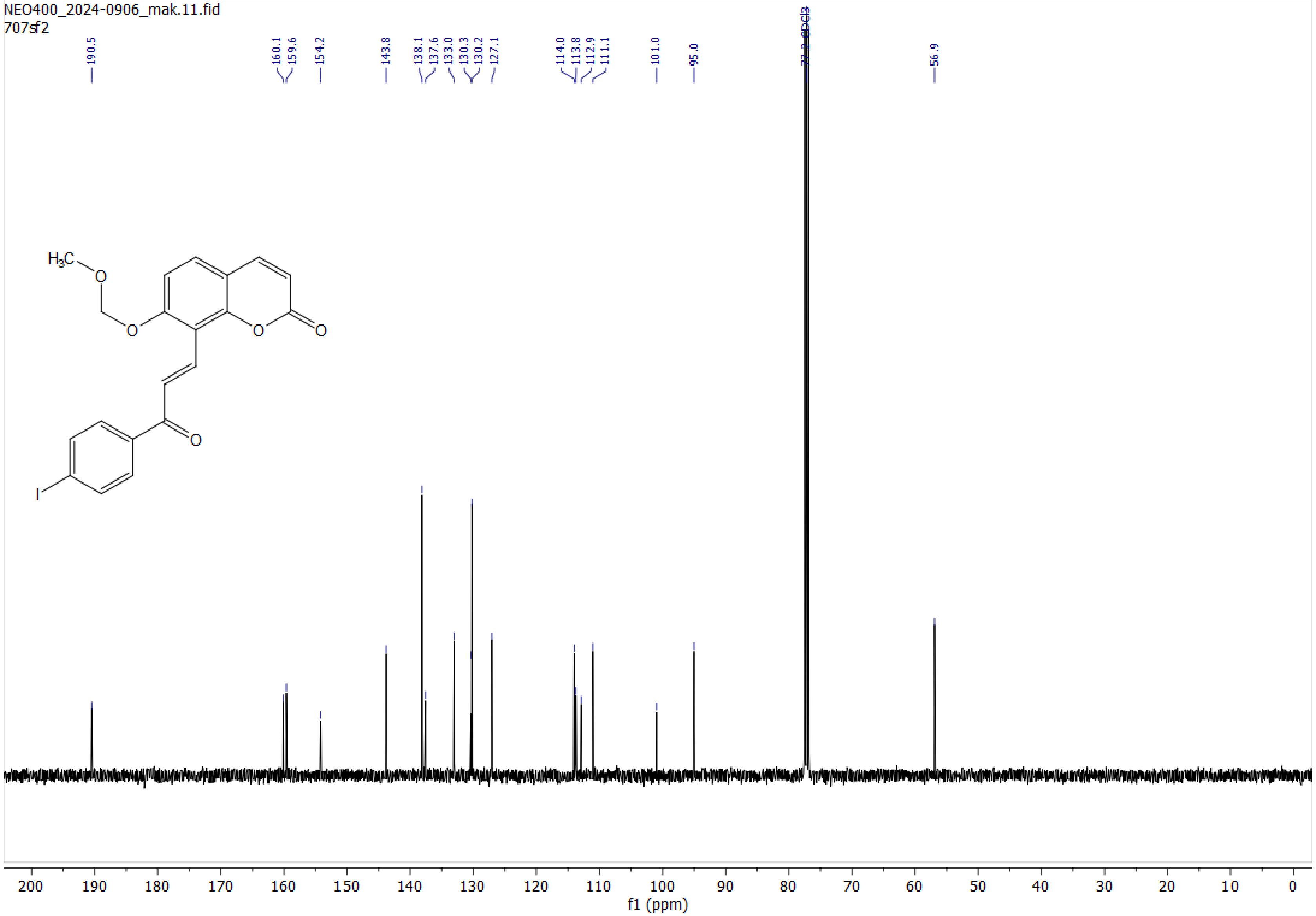

Figure S96: COSY (400 MHz, $CDCl_3$) of **13ae**

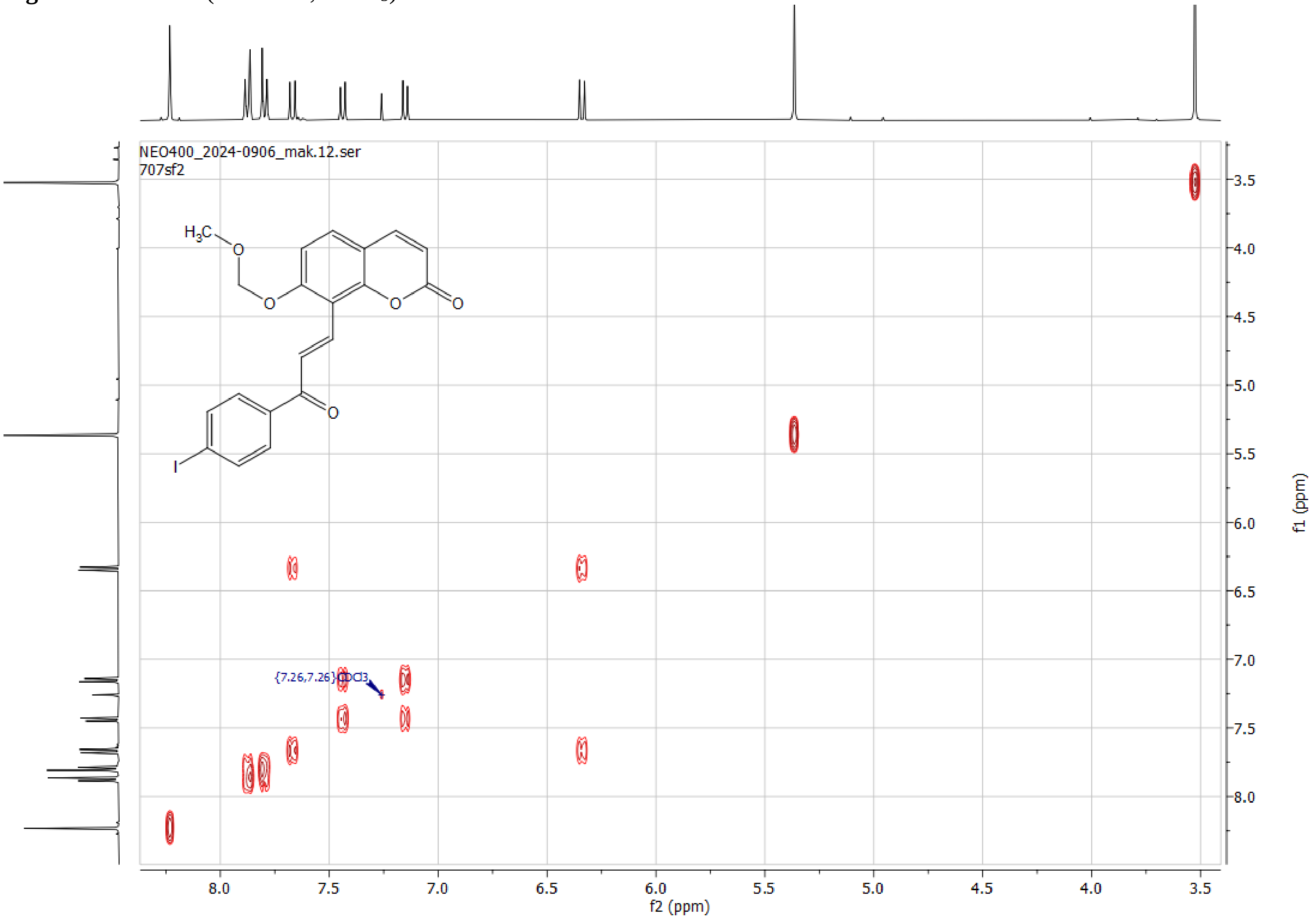

Figure S97: HSQC (400/100 MHz, $CDCl_3$) of **13ae**

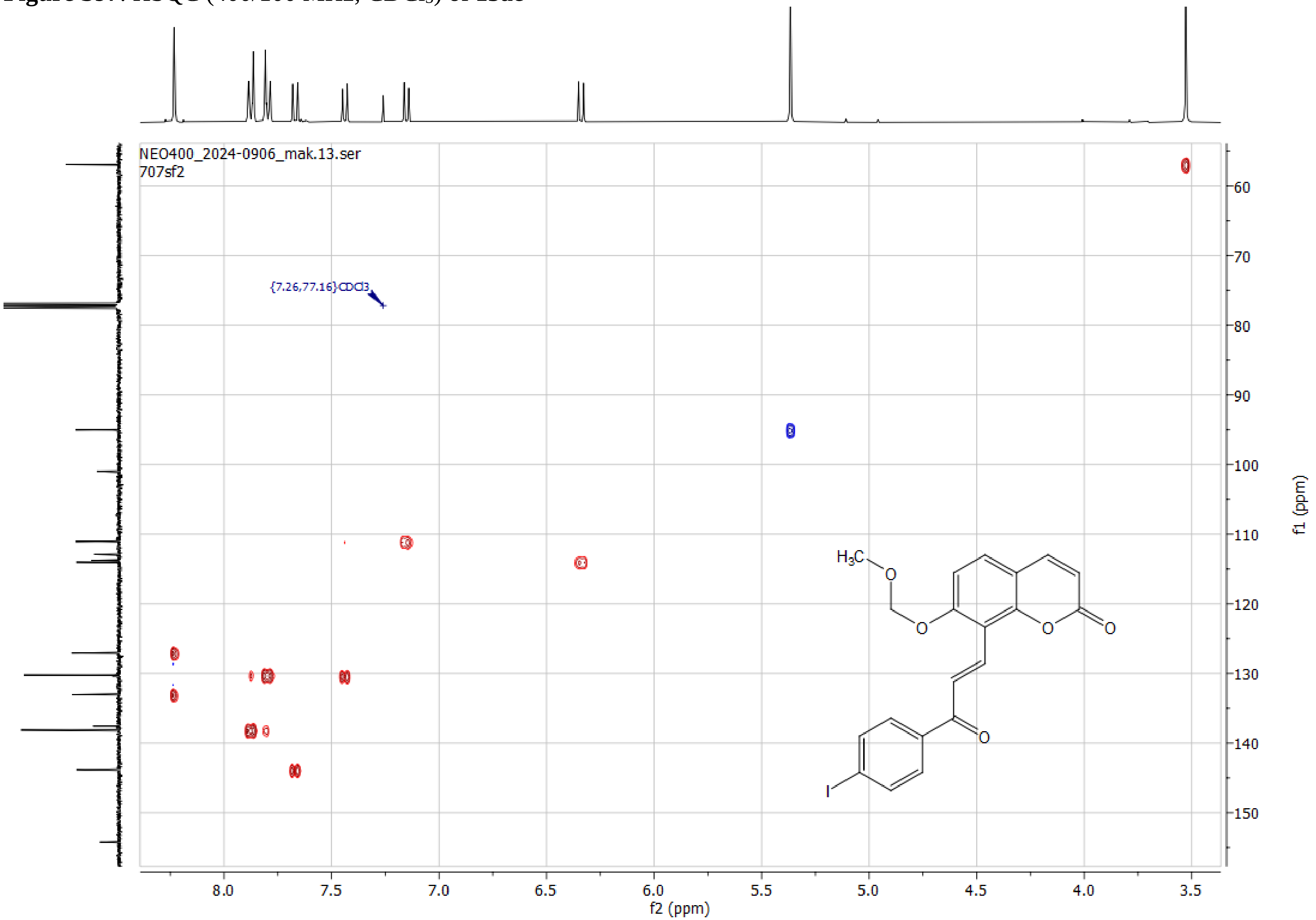

Figure S98: HMBC (400/100 MHz, $CDCl_3$) of **13ae**

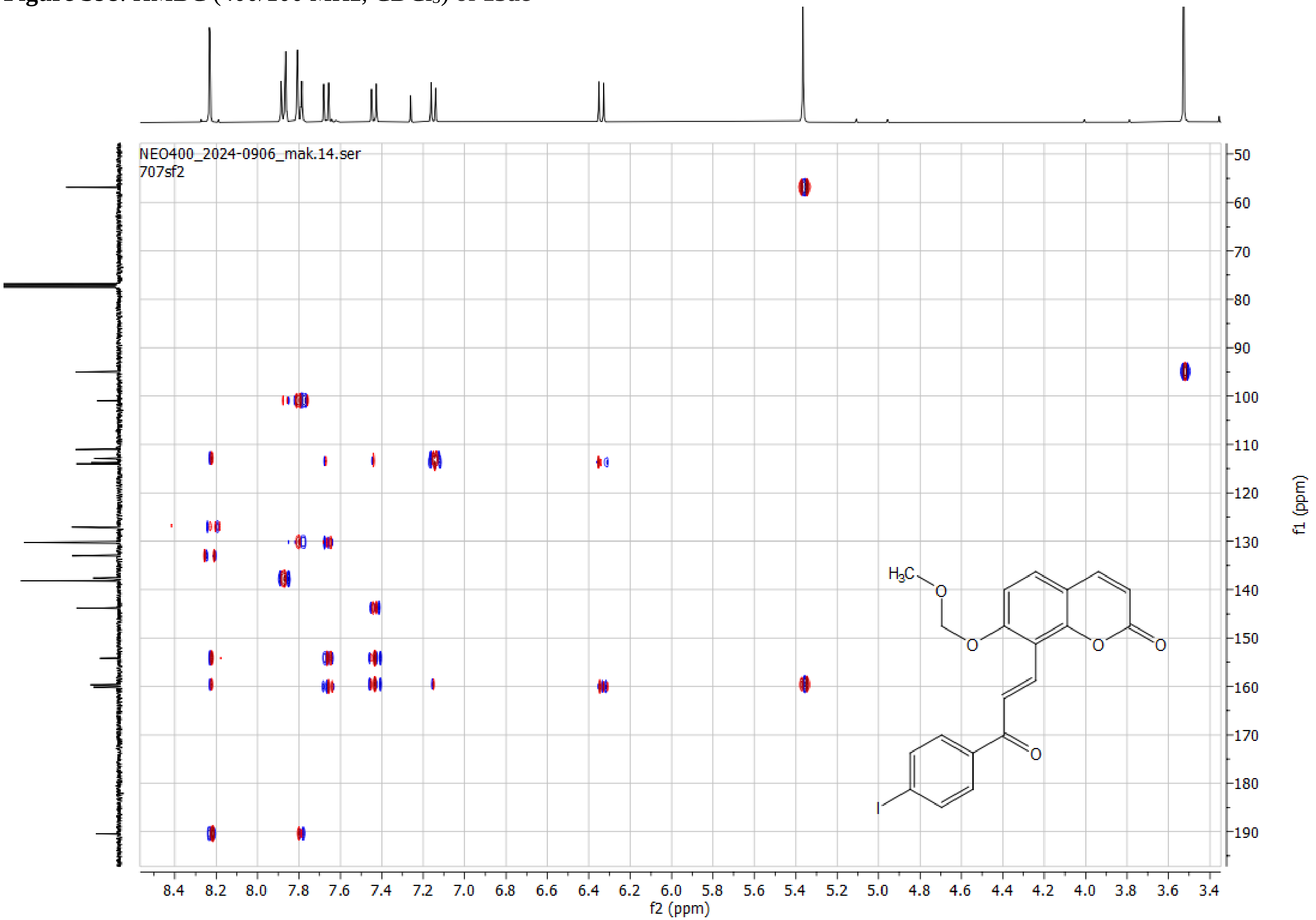

Figure S99: NOESY (400 MHz, $CDCl_3$) of **13ae**

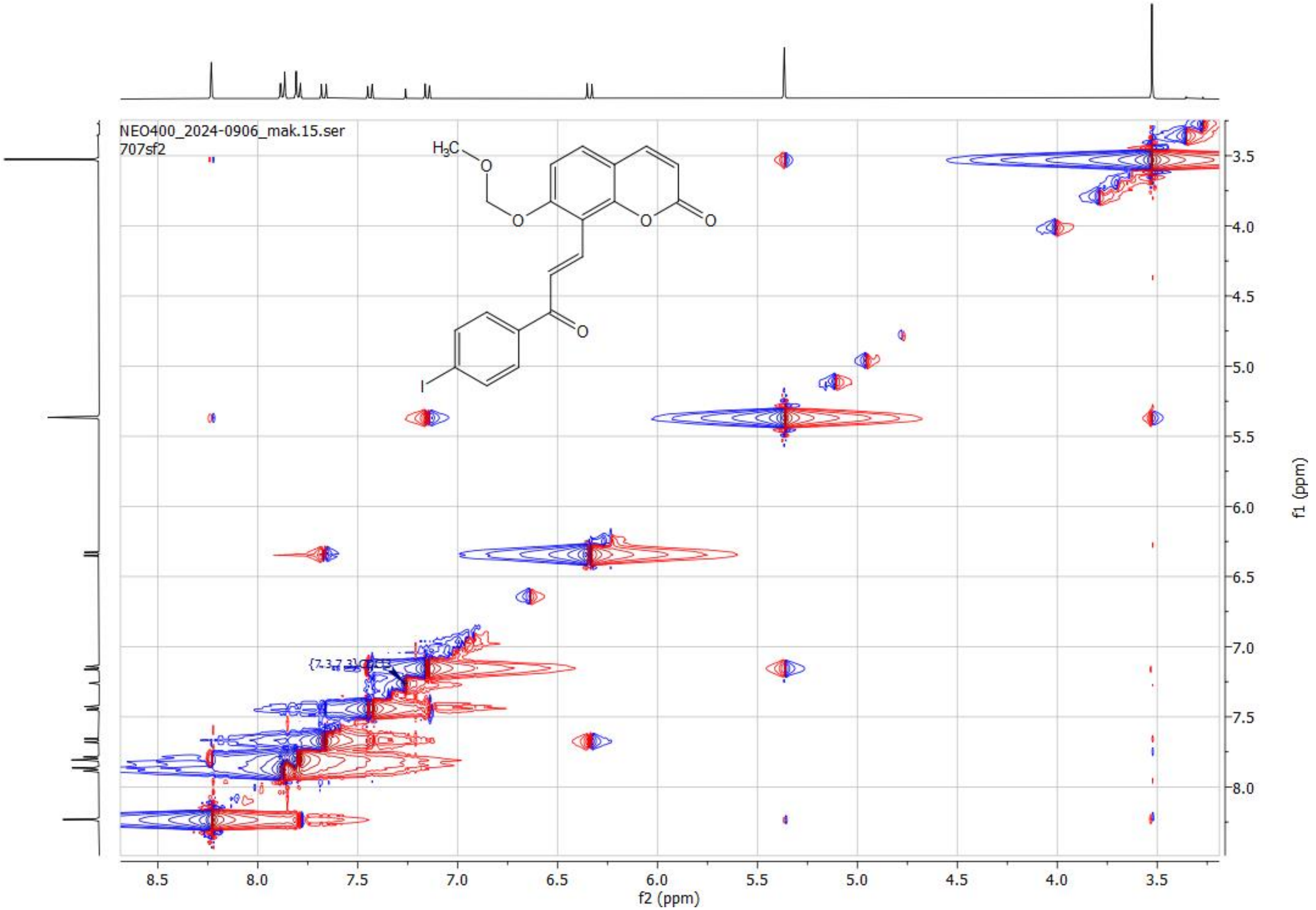

Figure S100: ^{1}H NMR (400 MHz, $CDCl_3$) of **14ae**

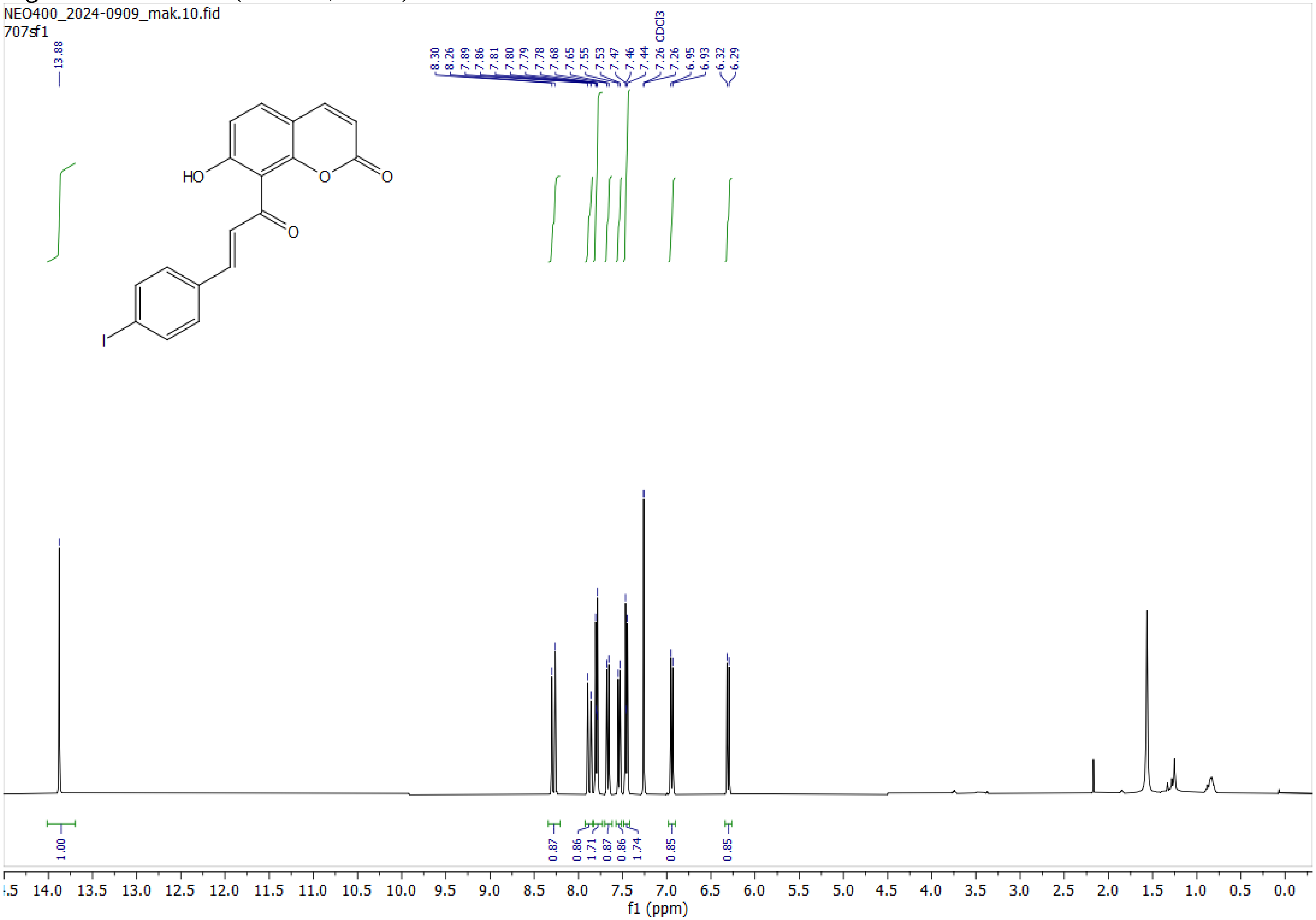

Figure S101: $^{13}C\{^{1}H\}$ NMR (100 MHz, $CDCl_3$) of **14ae**

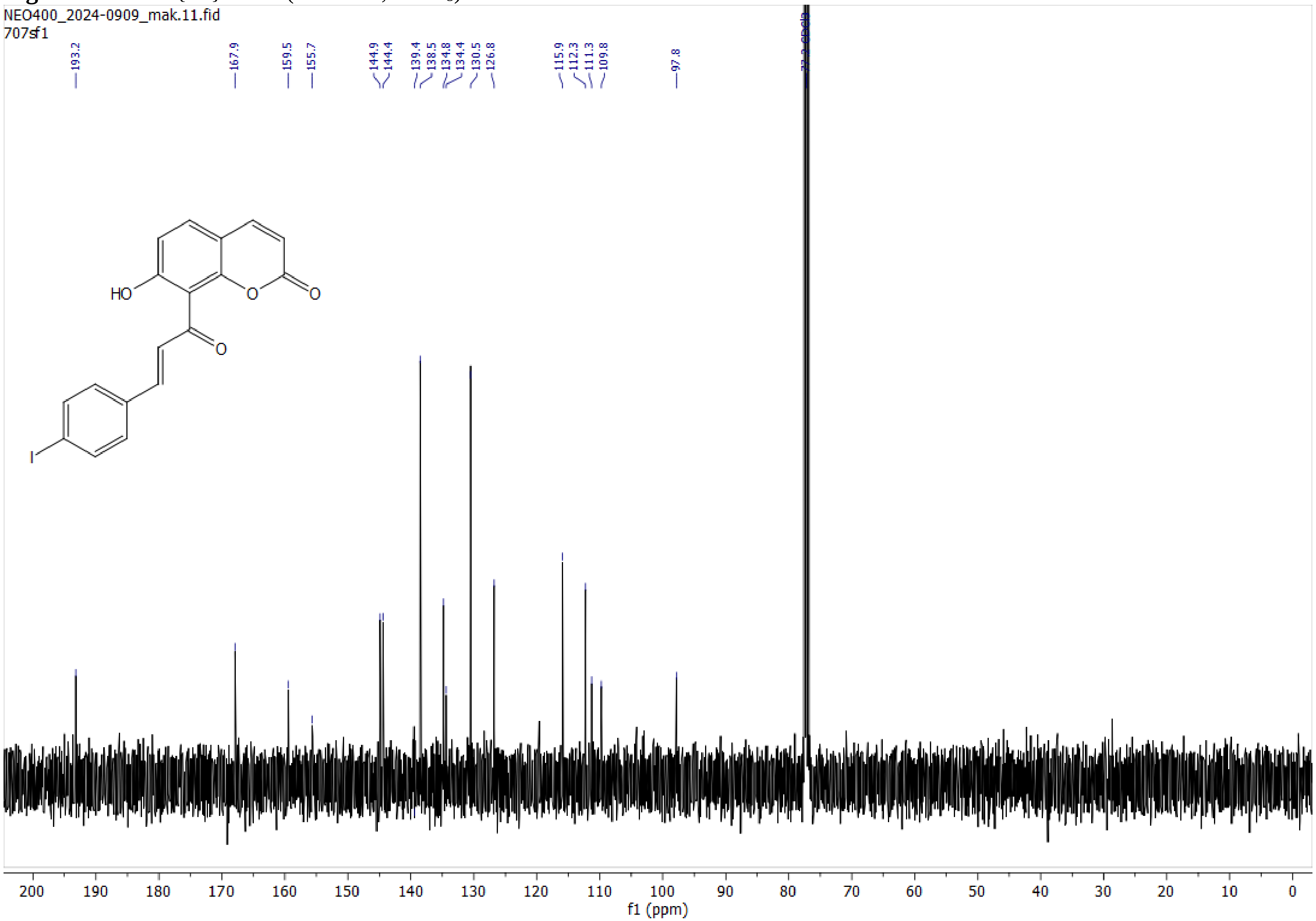

Figure S102: COSY (400 MHz, $CDCl_3$) of **14ae**

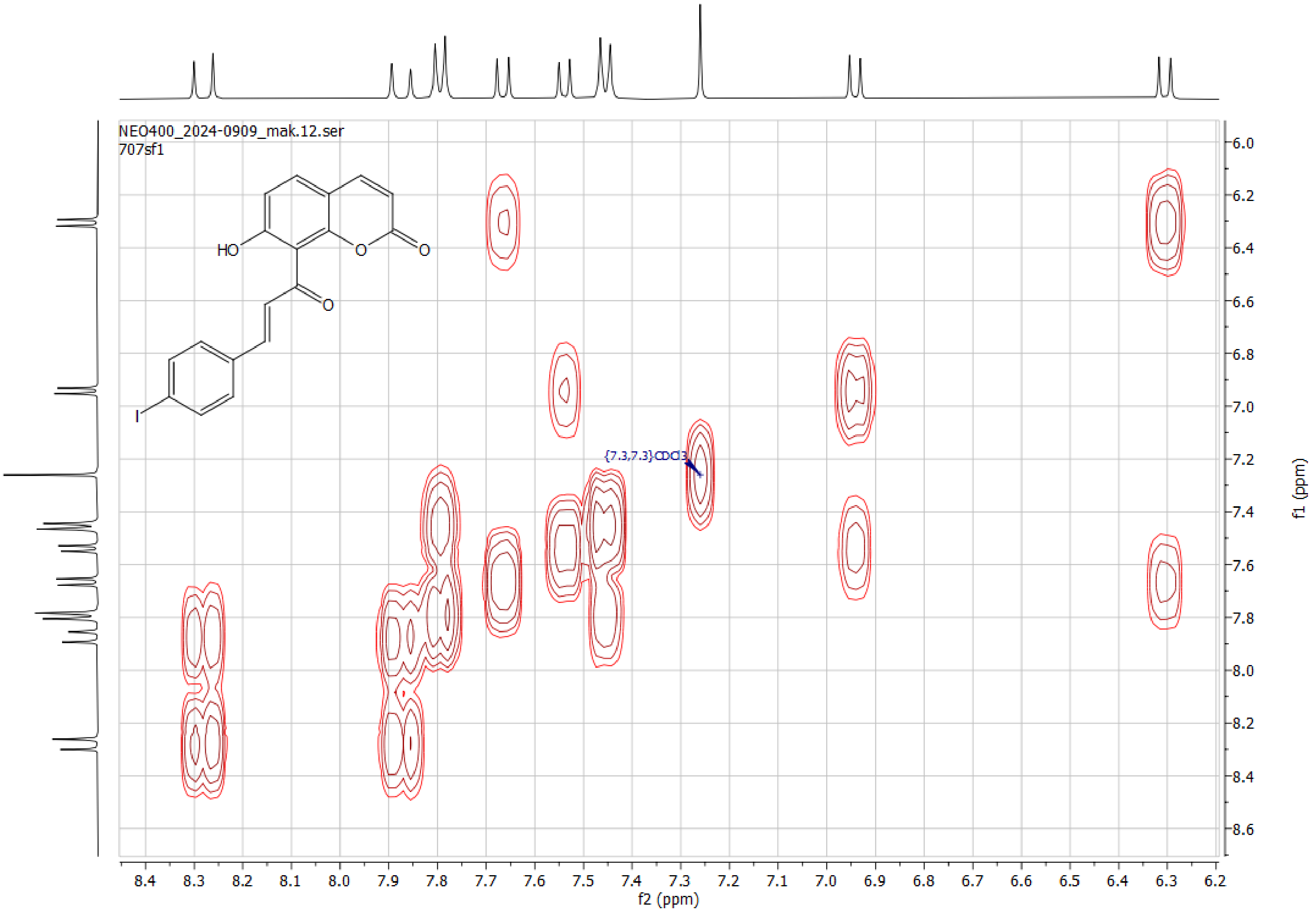

Figure S103: HSQC (400/100 MHz, $CDCl_3$) of **14ae**

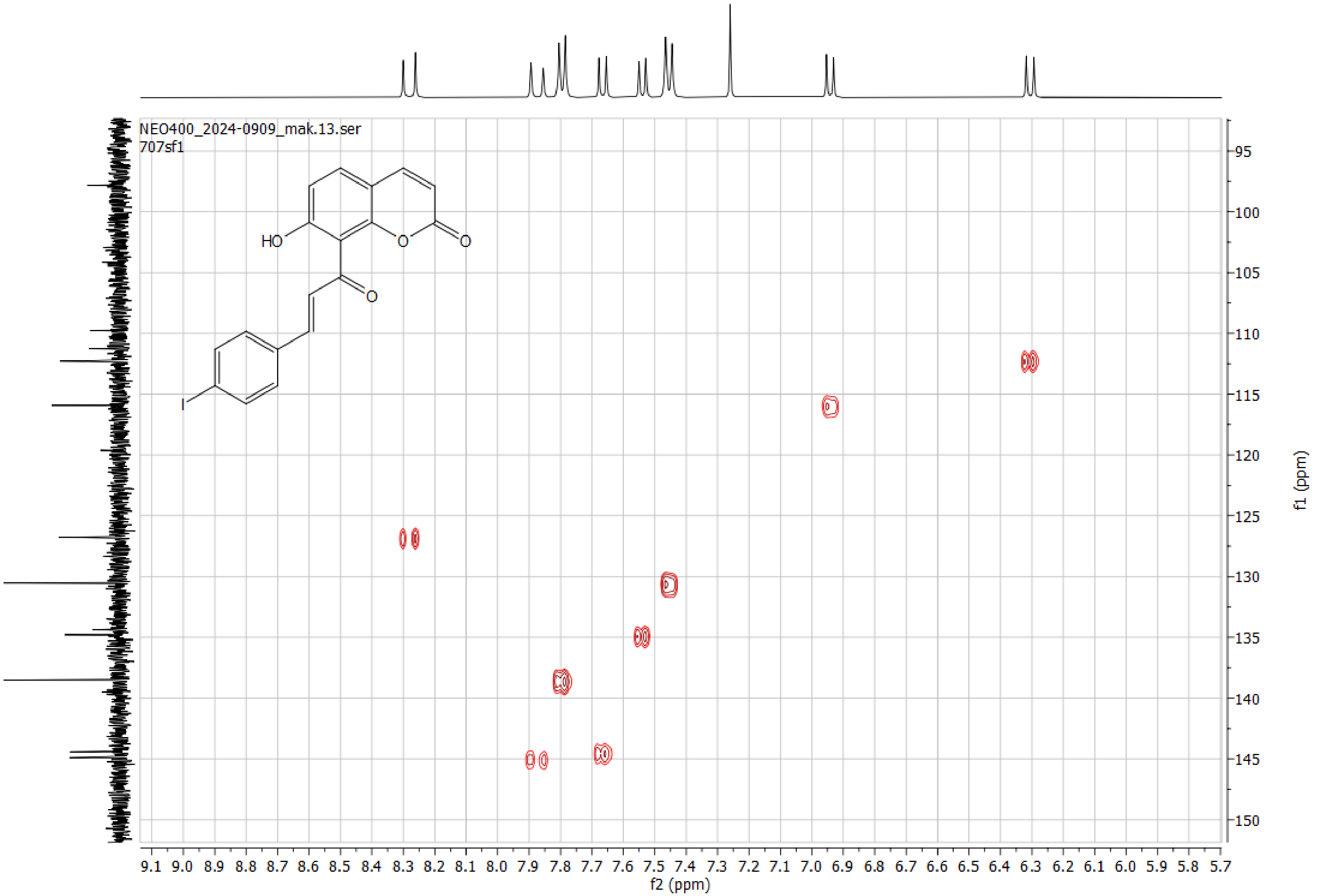

Figure S104: HMBC (400/100 MHz, $CDCl_3$) of **14ae**

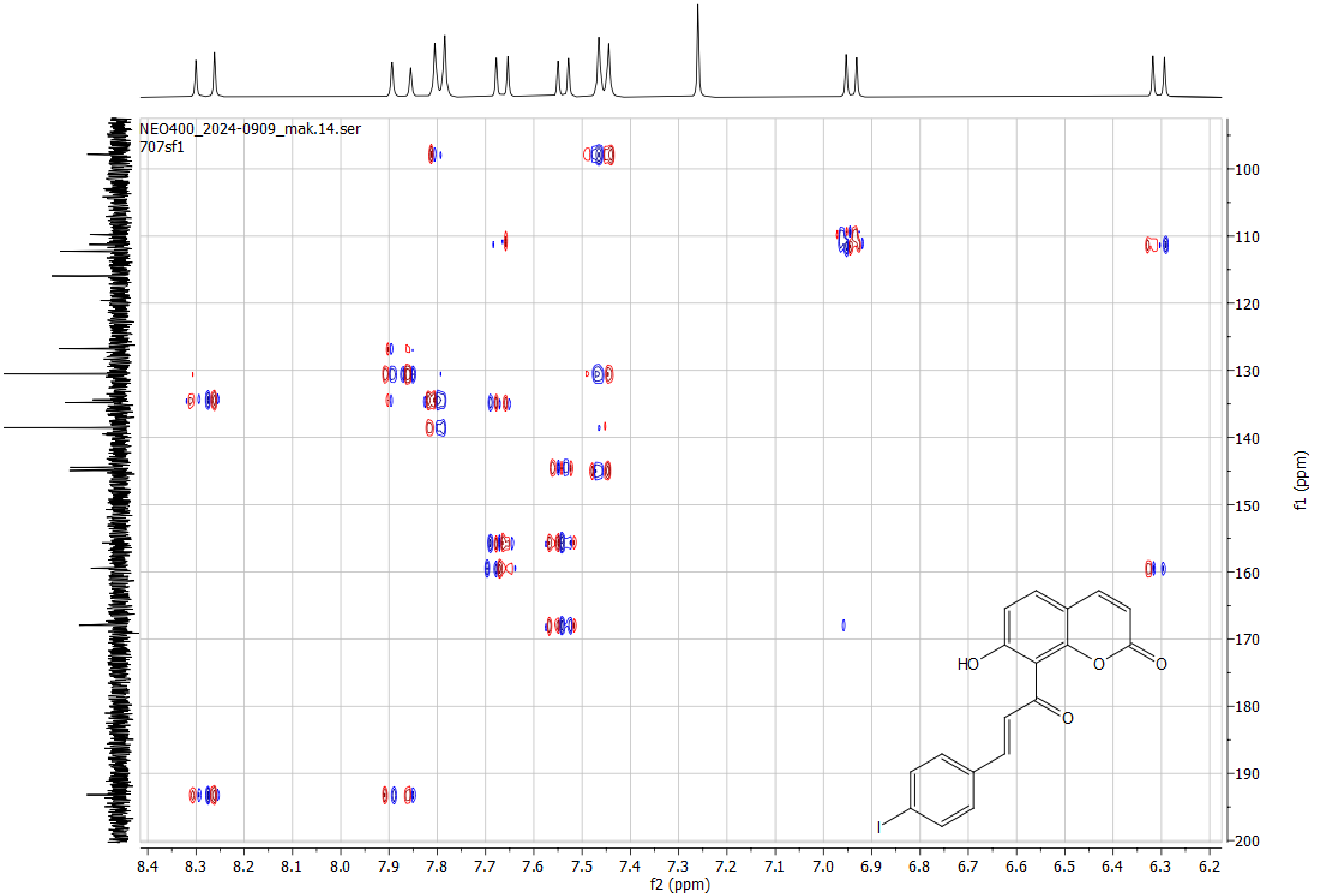

Figure S105: NOESY (400 MHz, $CDCl_3$) of **14ae**

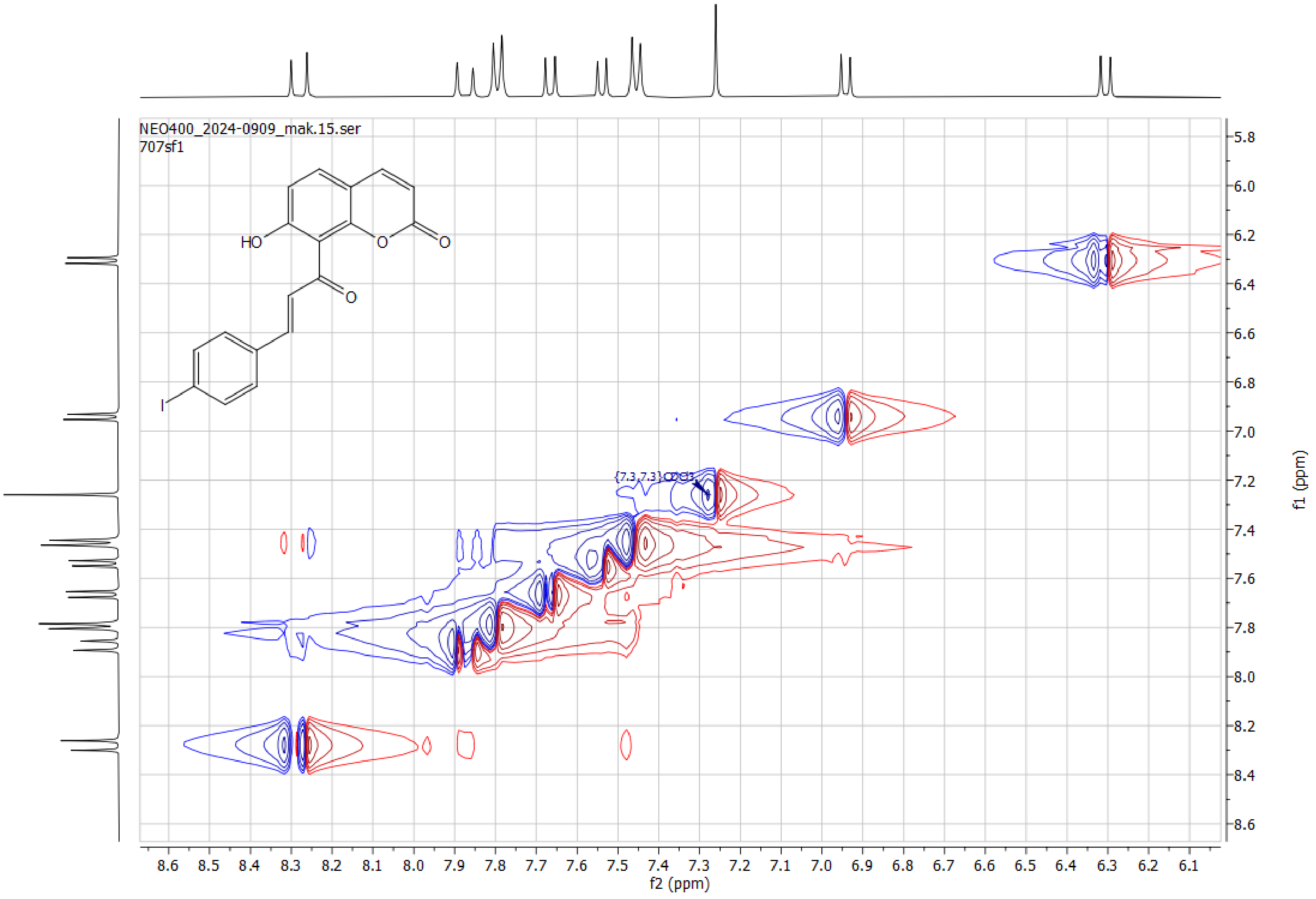

Figure S106: NMR-Signal assignment and selected HMBC-couplings for compound **13af**

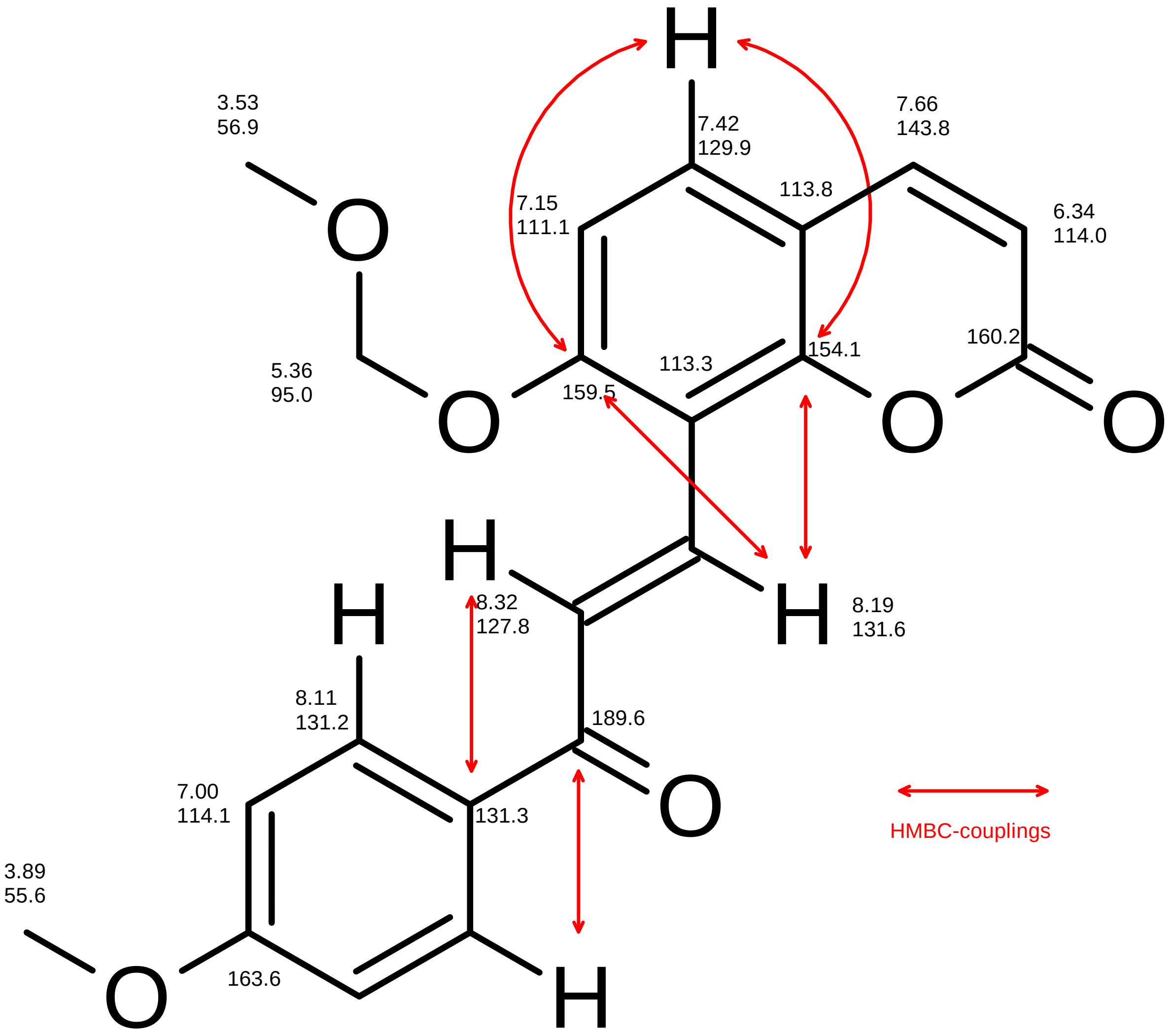

Figure S107: ^{1}H NMR (400 MHz, $CDCl_3$) of **13af**

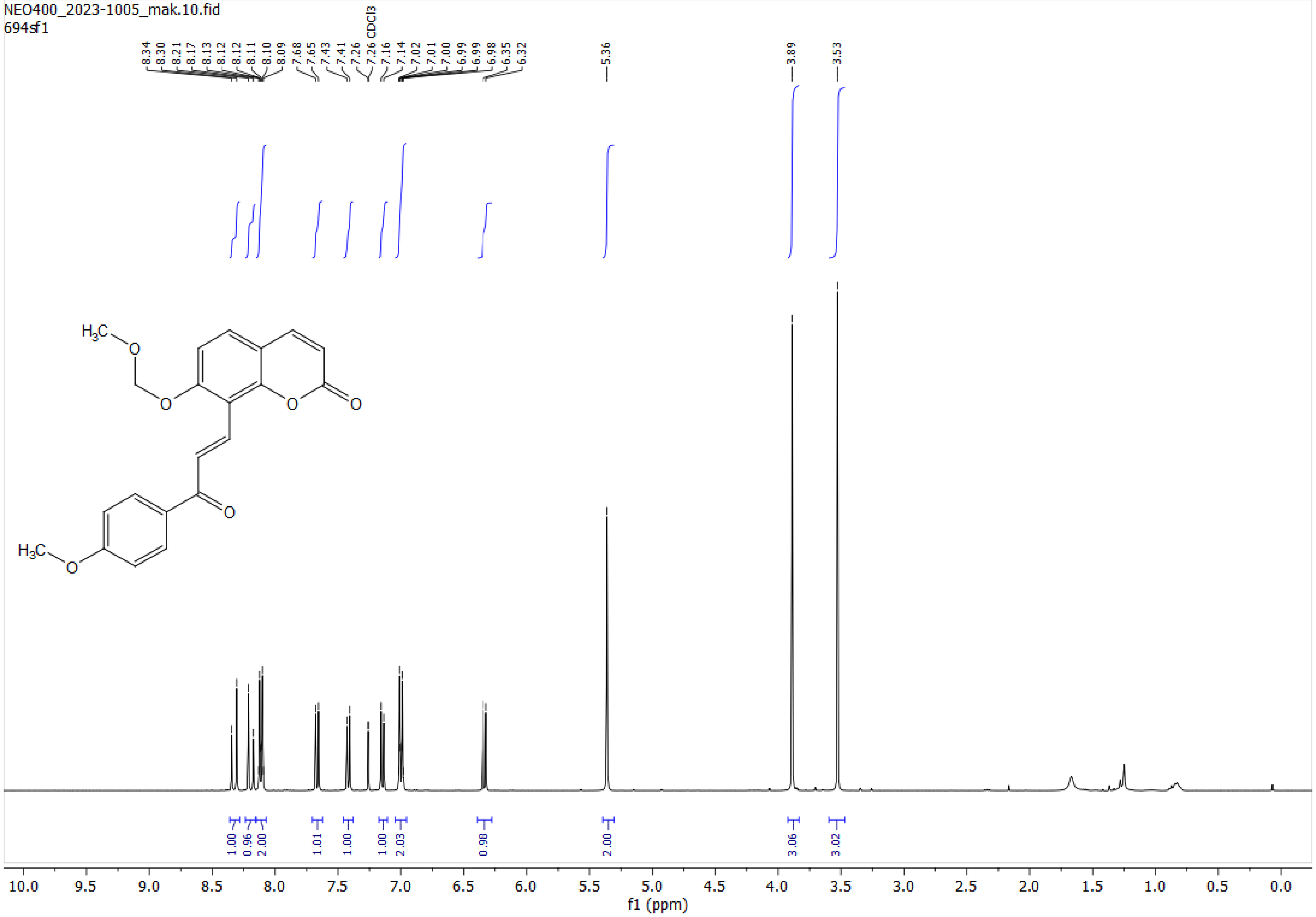

Figure S108: $^{13}C\{^{1}H\}$ NMR (100 MHz, $CDCl_3$) of **13af**

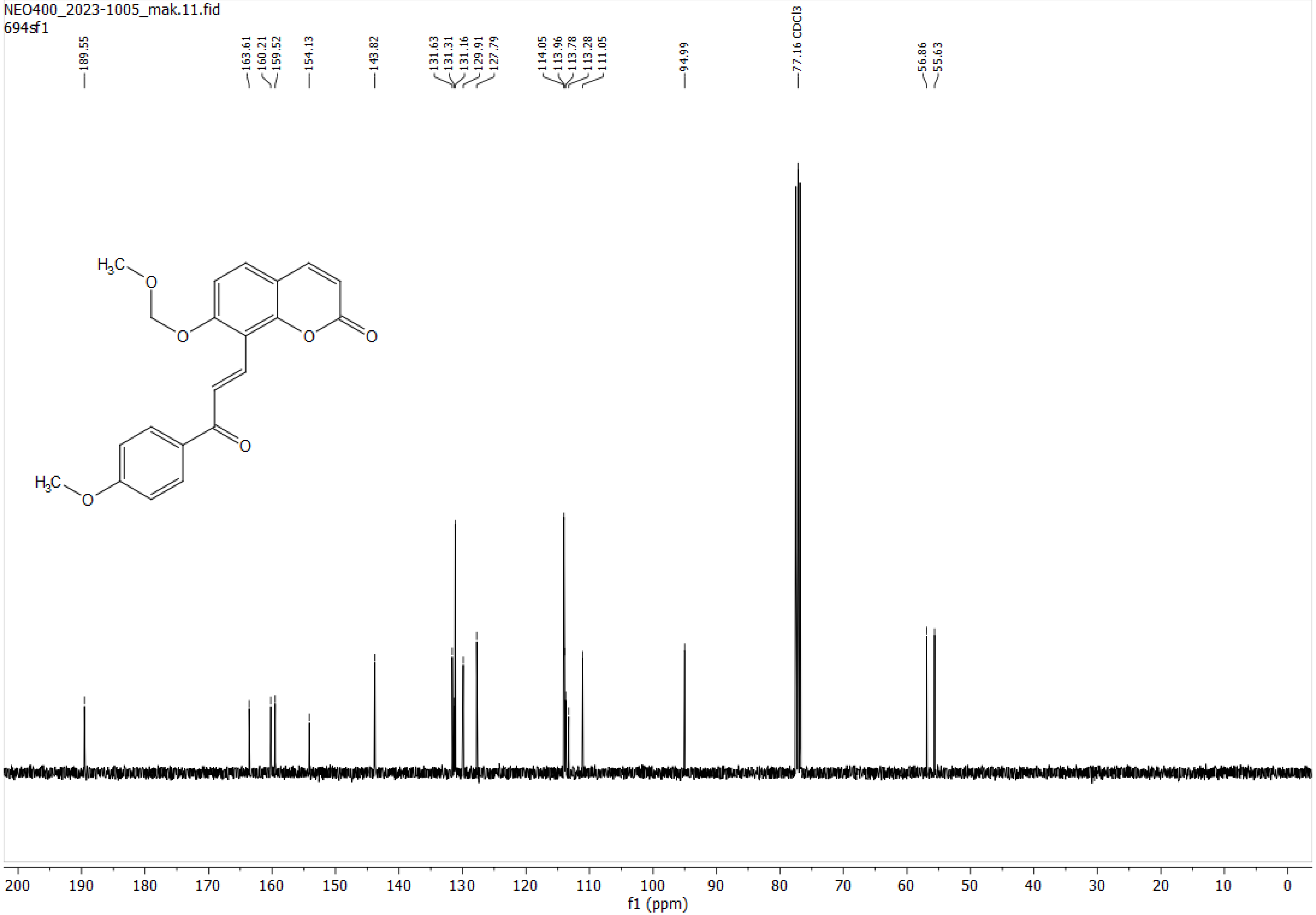

Figure S109: COSY (400 MHz, $CDCl_3$) of **13af**

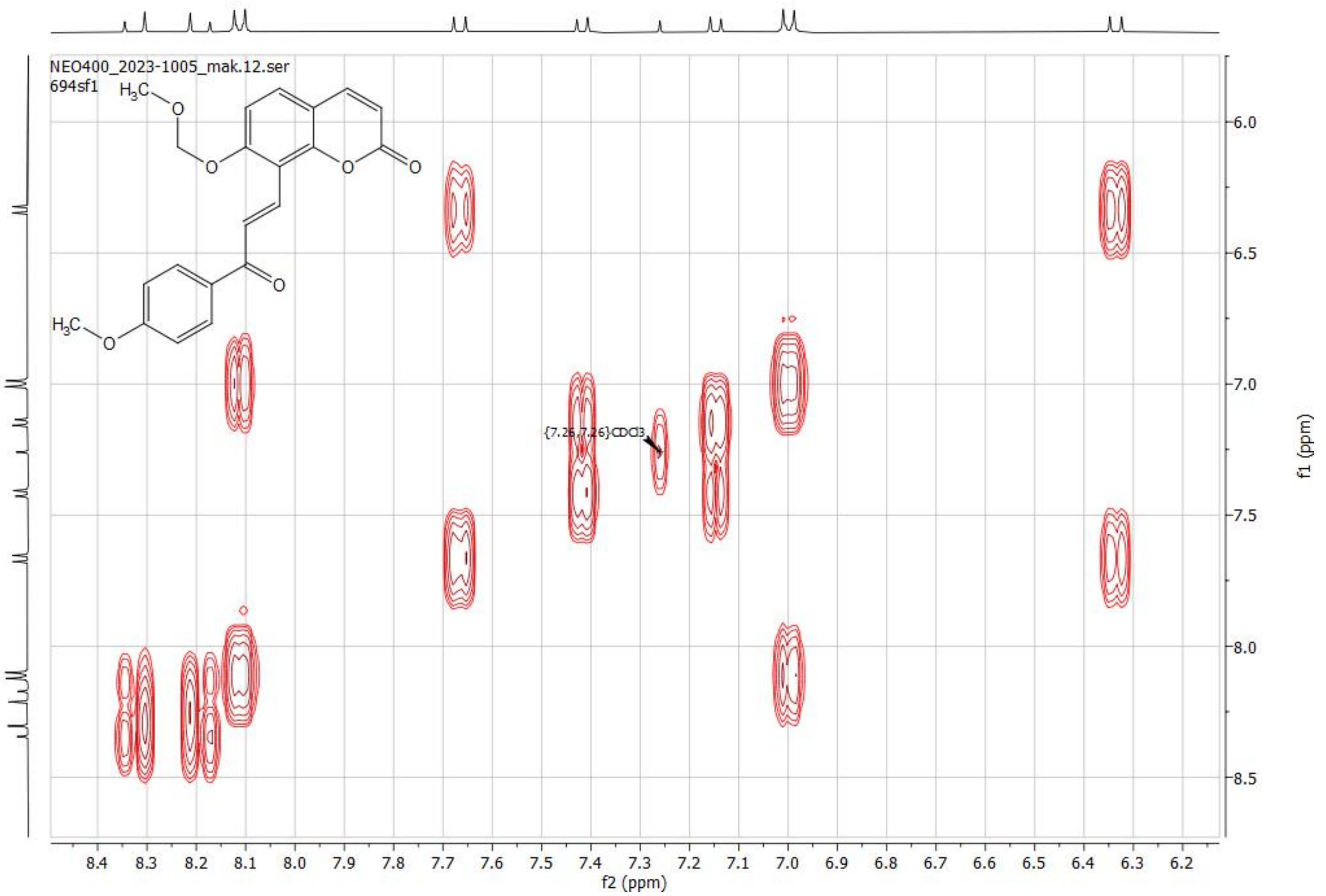

Figure S110: HSQC (400/100 MHz, $CDCl_3$) of **13af**

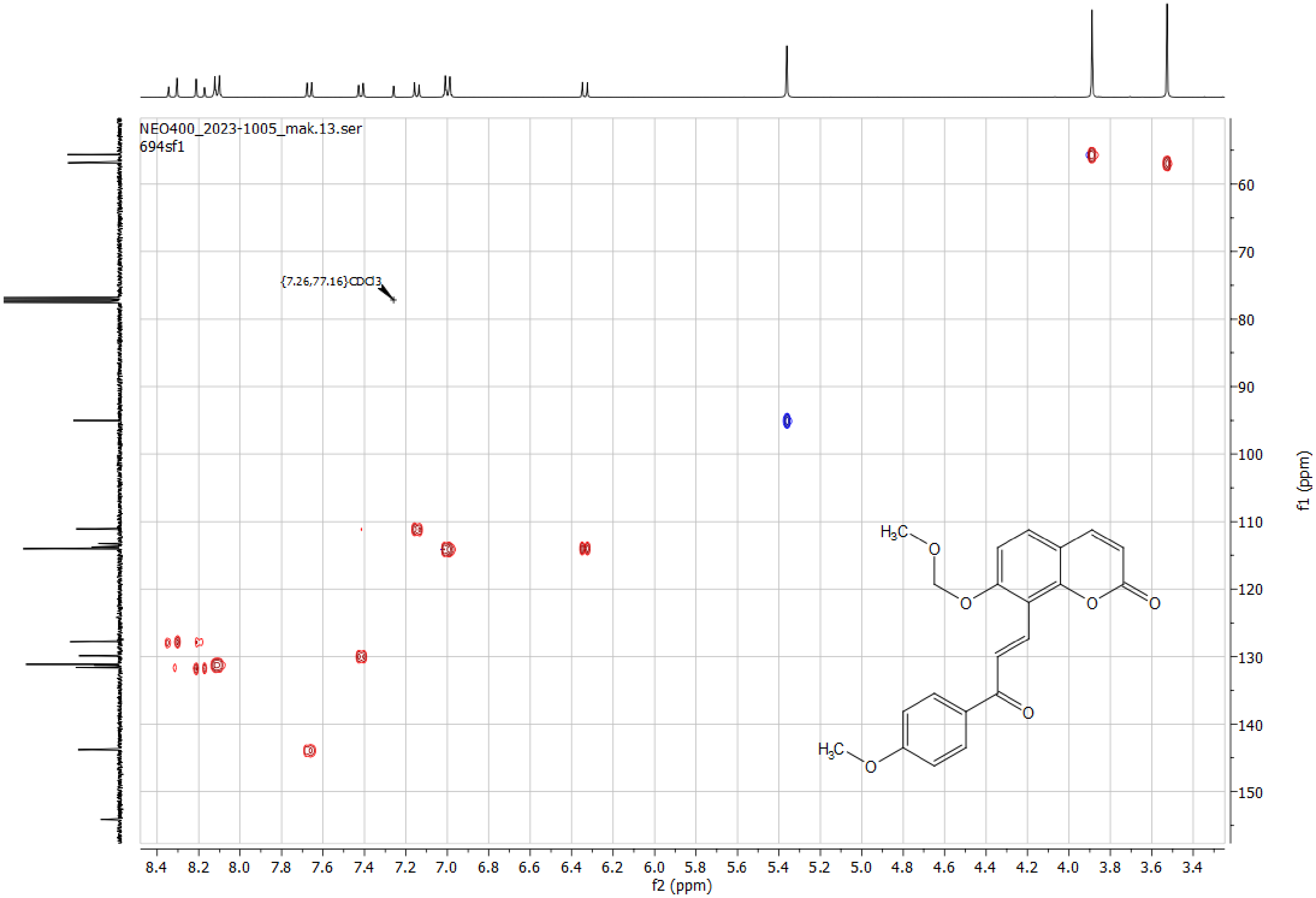

Figure S111: HMBC (400/100 MHz, $CDCl_3$) of **13af**

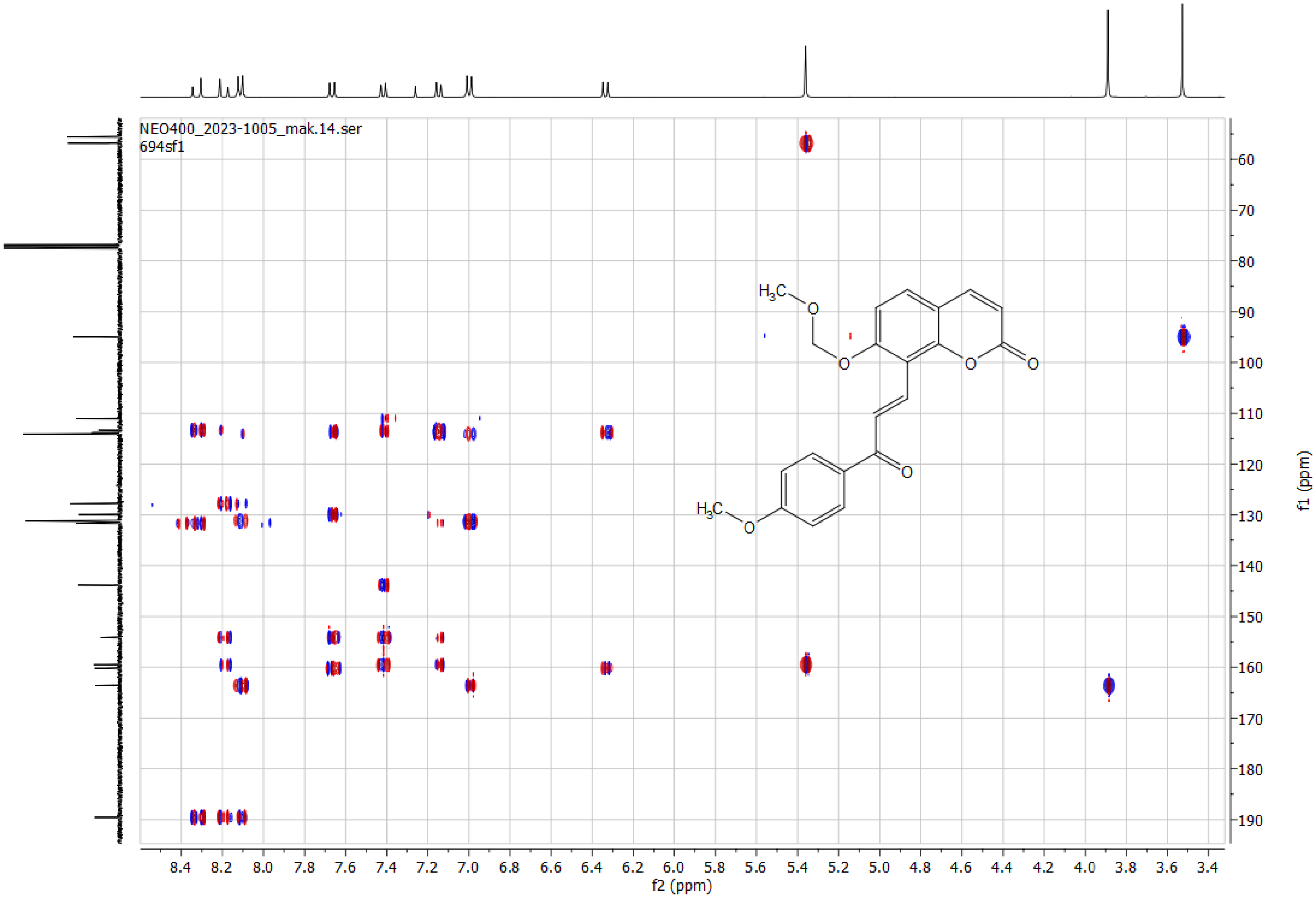

Figure S112: NOESY (400 MHz, $CDCl_3$) of **13af**

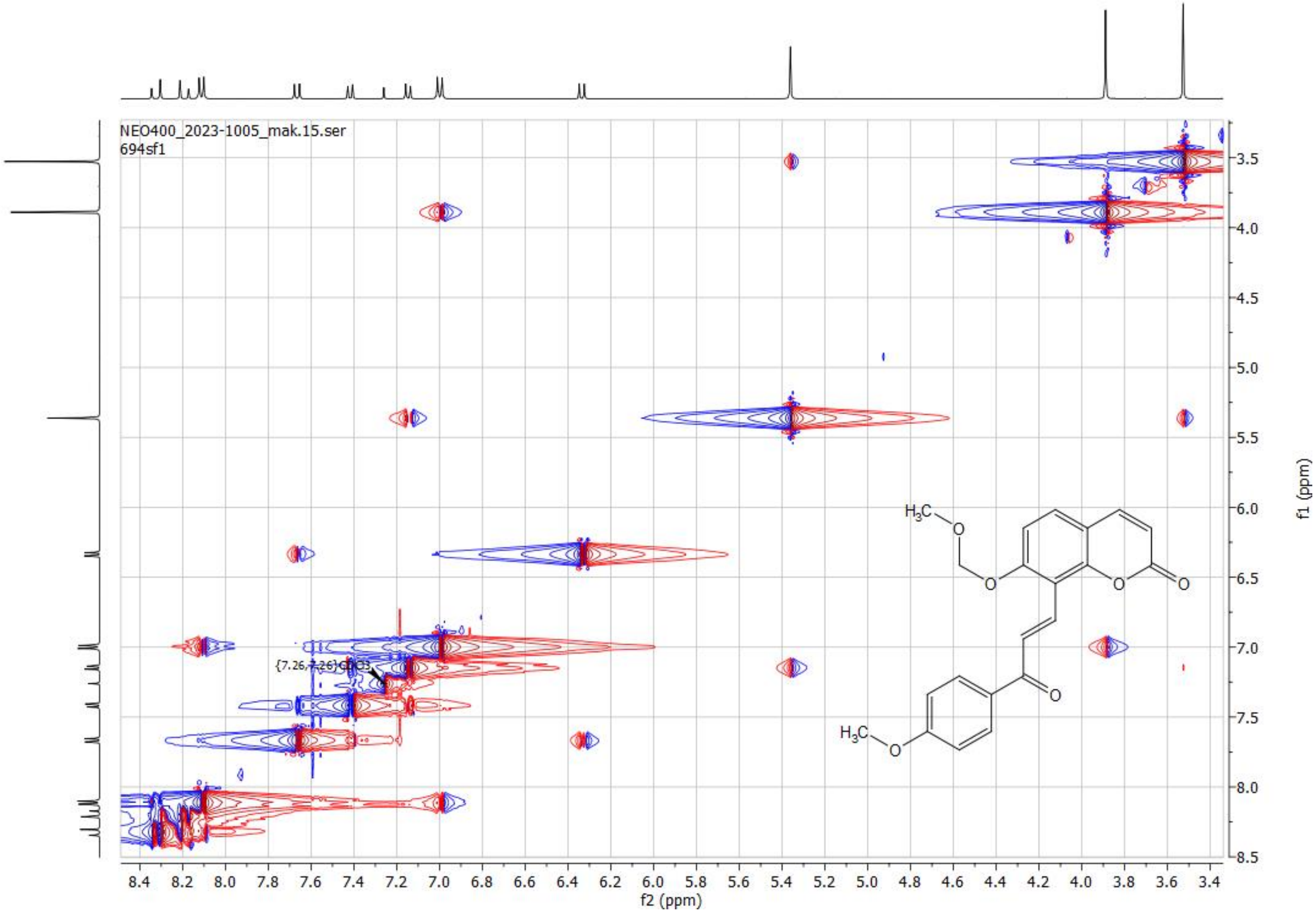

Figure S113: ^{1}H NMR (400 MHz, $CDCl_3$) of **13ai**

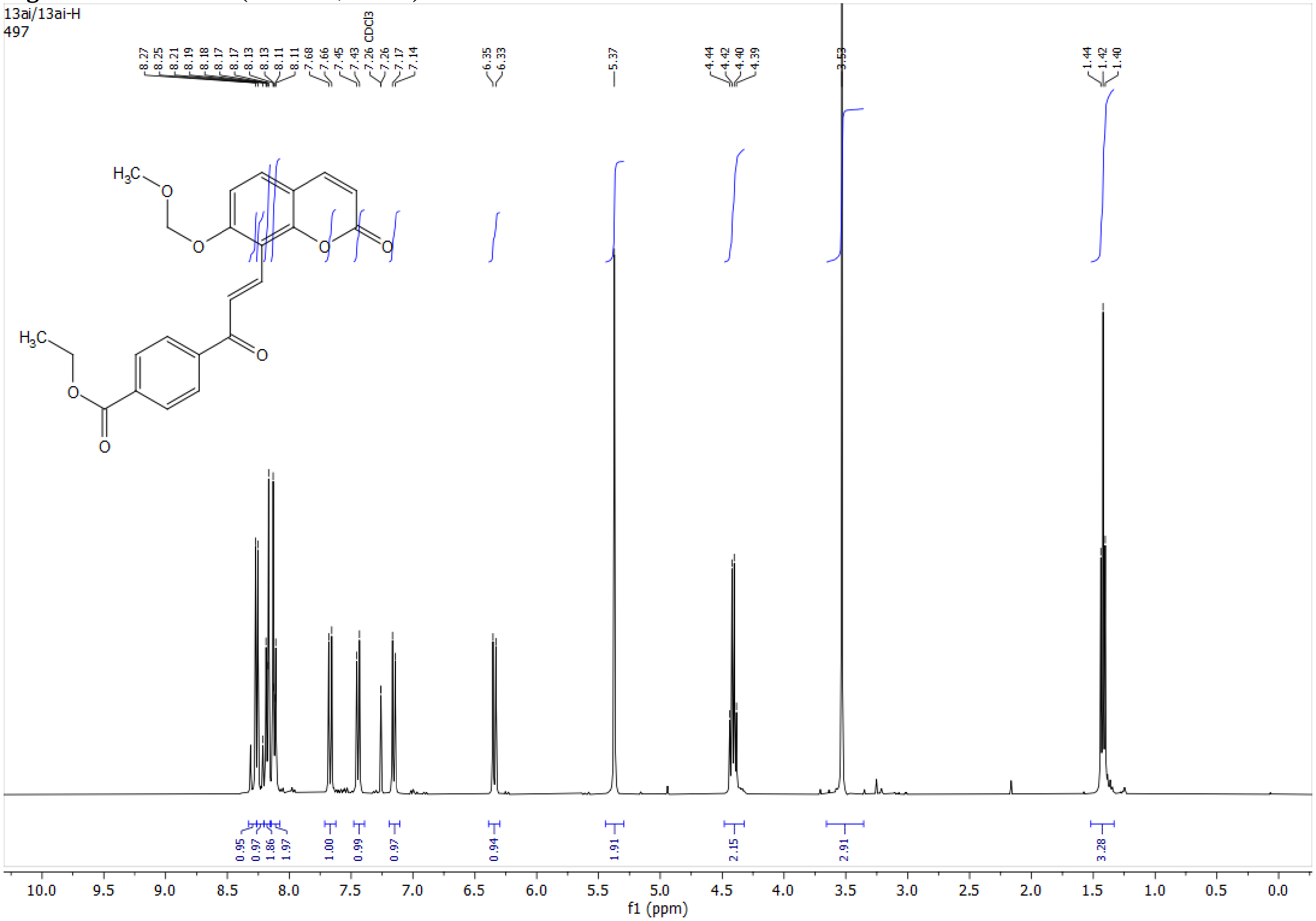

Figure S114: $^{13}C\{^{1}H\}$ NMR (100 MHz, $CDCl_3$) of **13ai**

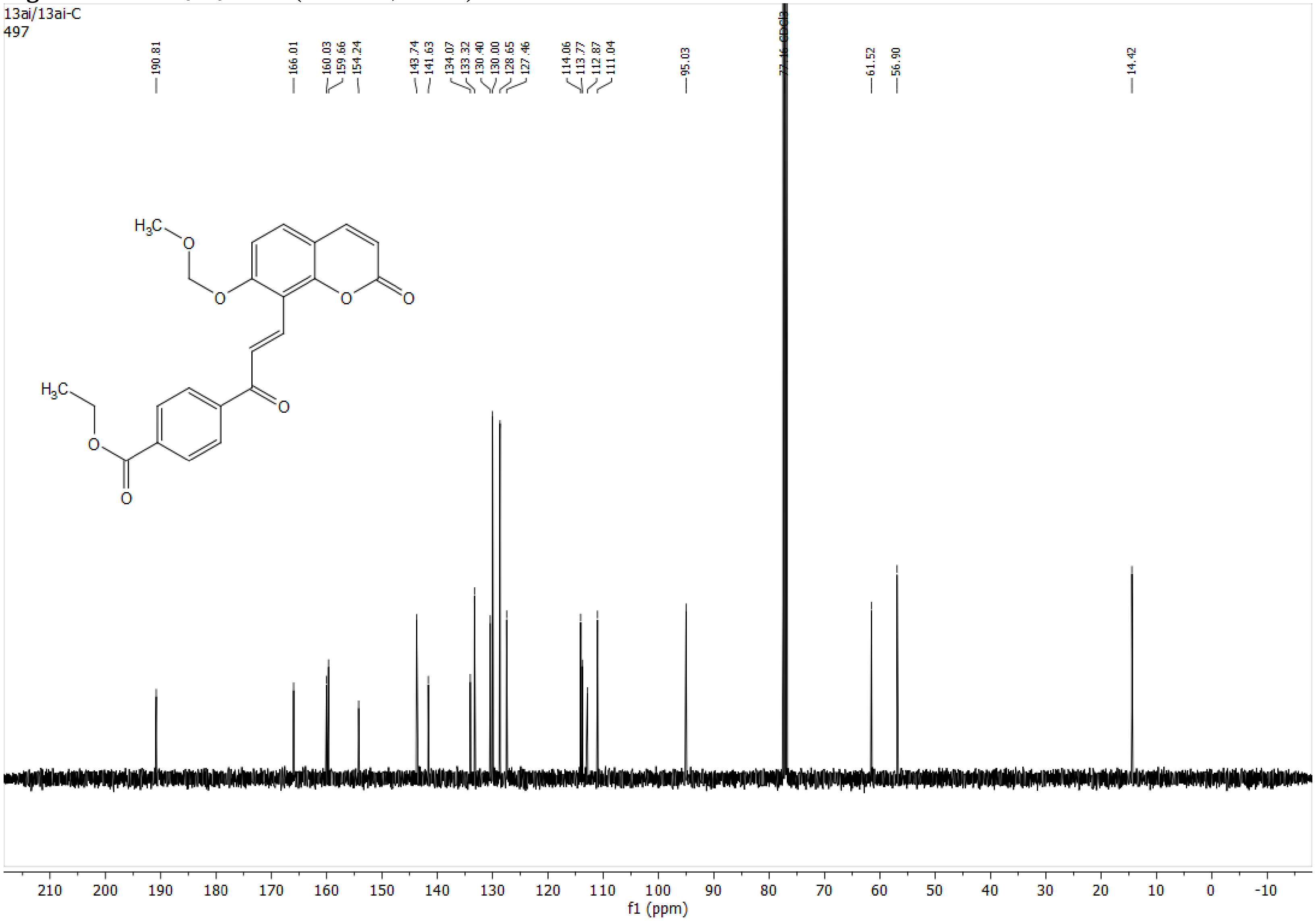

Figure S115: ^{1}H NMR (400 MHz, $CDCl_3$) of **13ak**

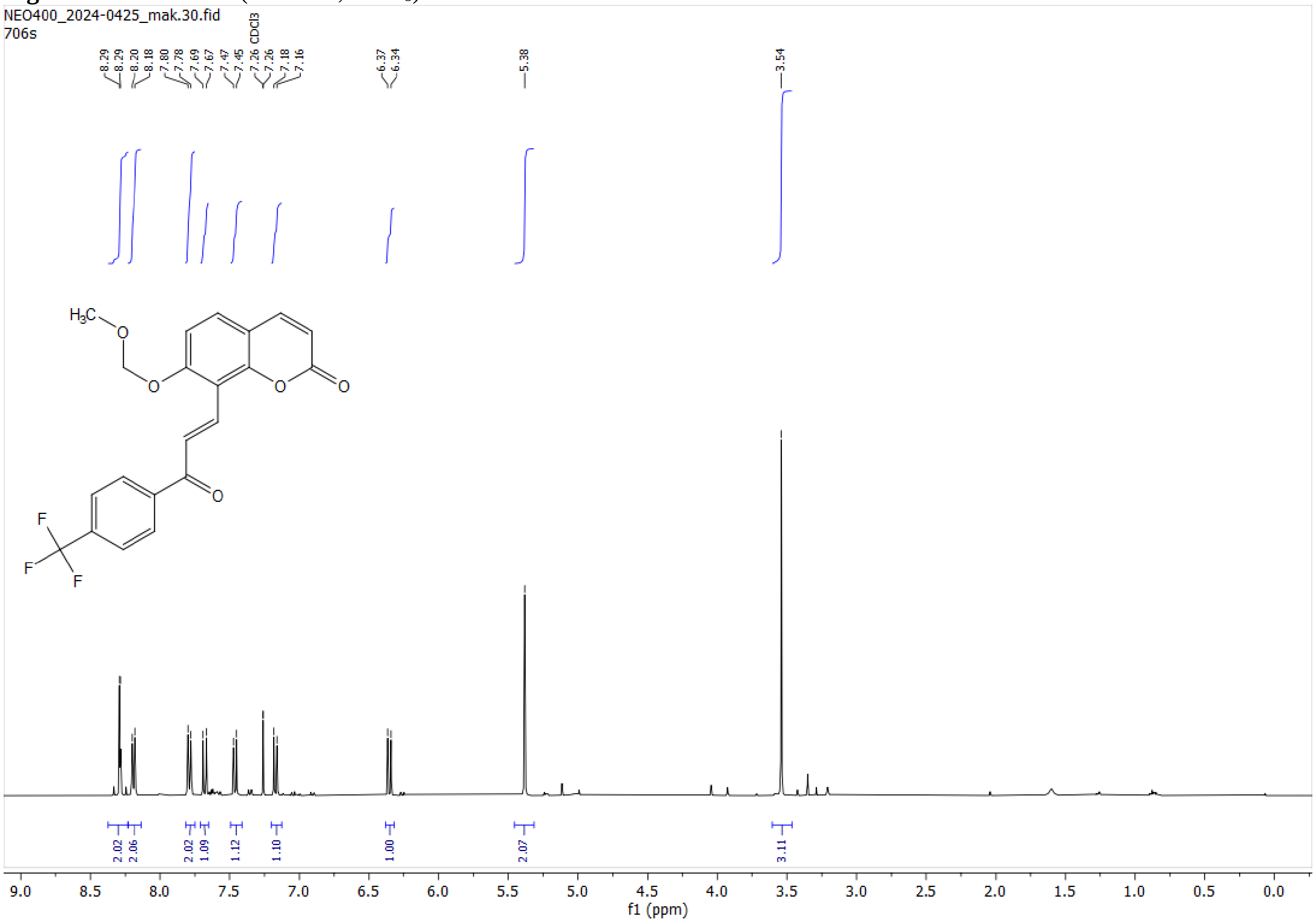

Figure S116: $^{13}C\{^{1}H\}$ NMR (100 MHz, $CDCl_3$) of **13ak**

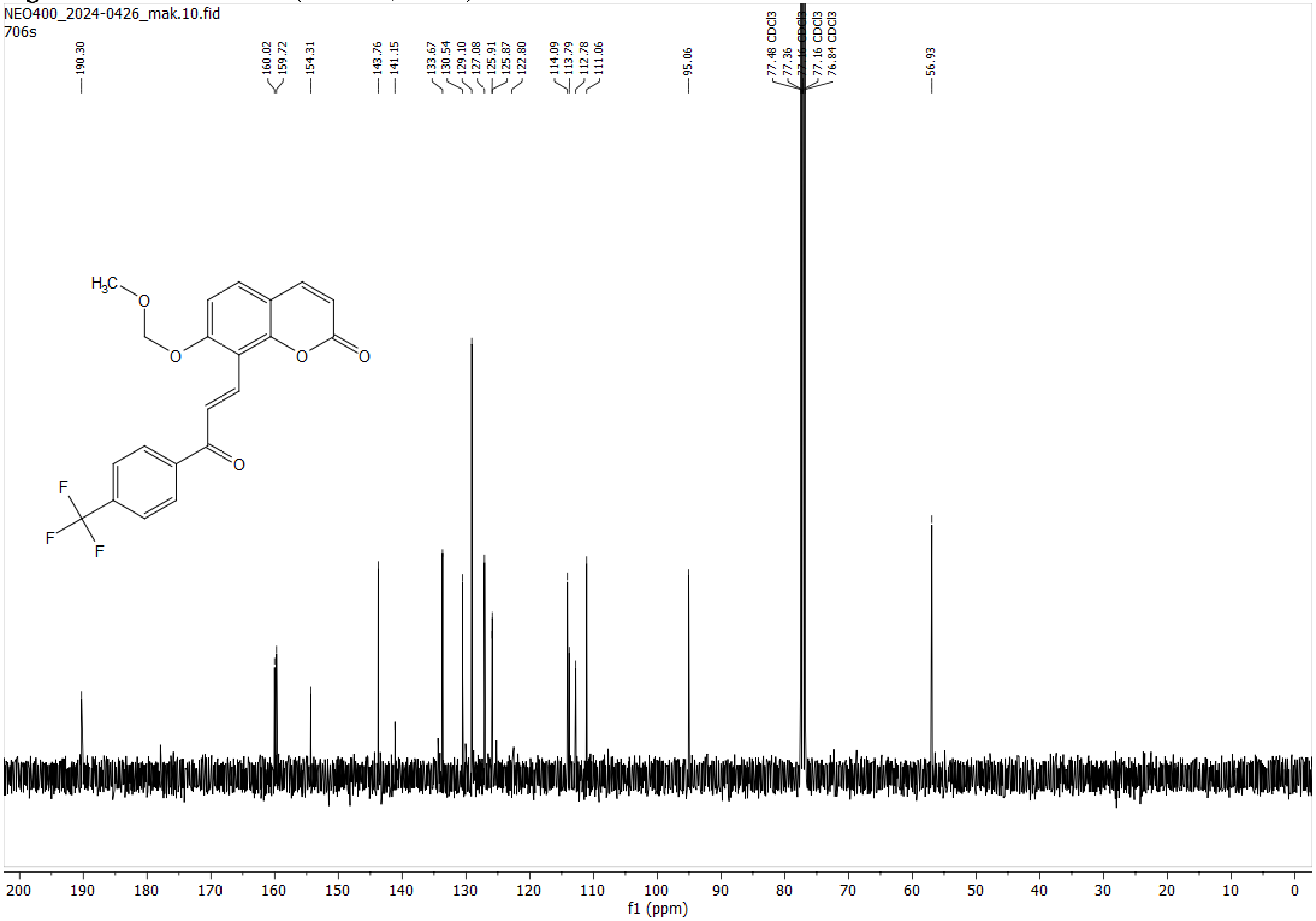

Figure S117: ^{1}H NMR (400 MHz, $CDCl_3$) of **13al**

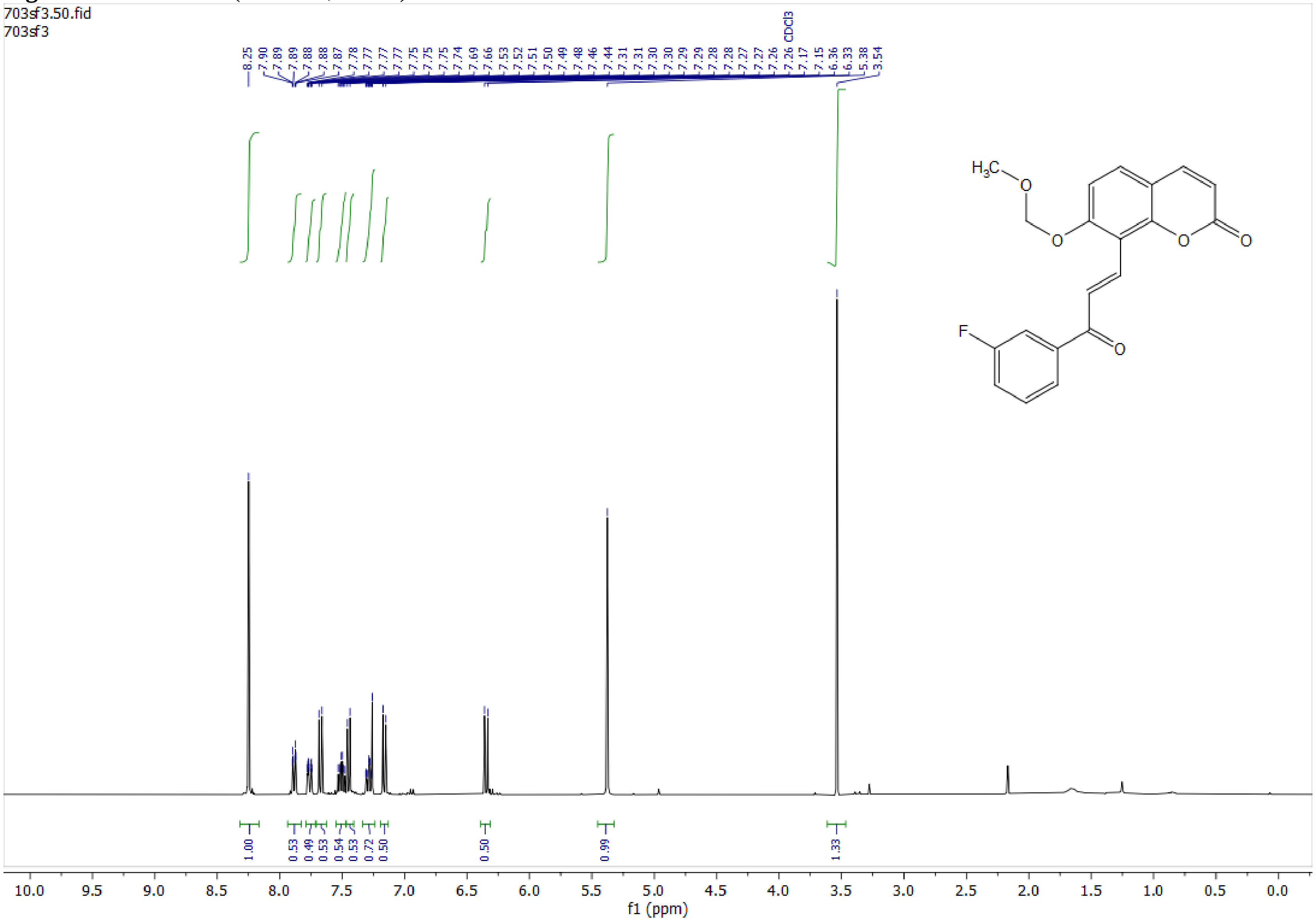

Figure S118: $^{13}C\{^{1}H\}$ NMR (100 MHz, $CDCl_3$) of **13al**

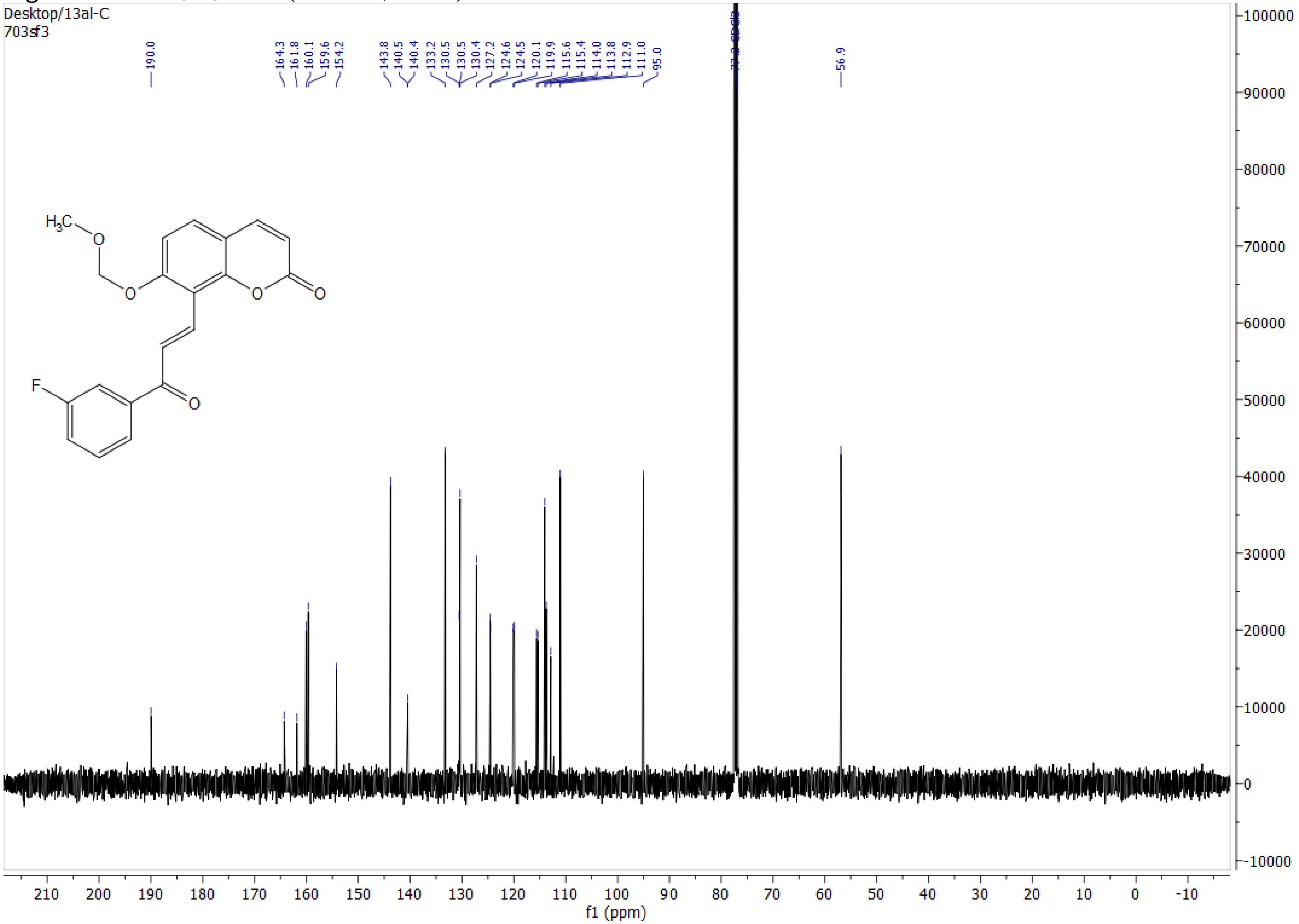

Figure S119: ^{1}H NMR (400 MHz, $CDCl_3$) of **13am**

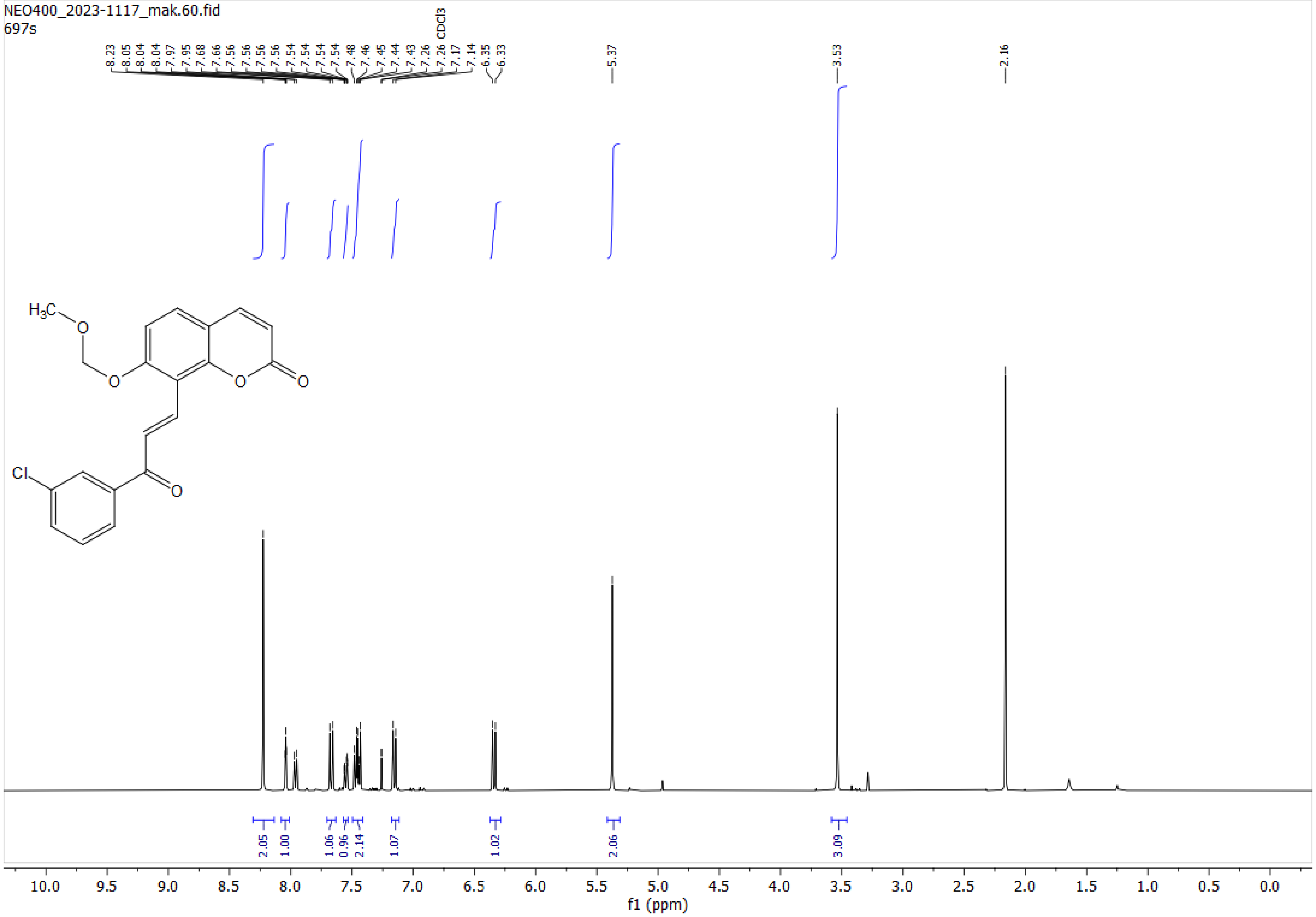

Figure S120: $^{13}C\{^{1}H\}$ NMR (100 MHz, $CDCl_3$) of **13am**

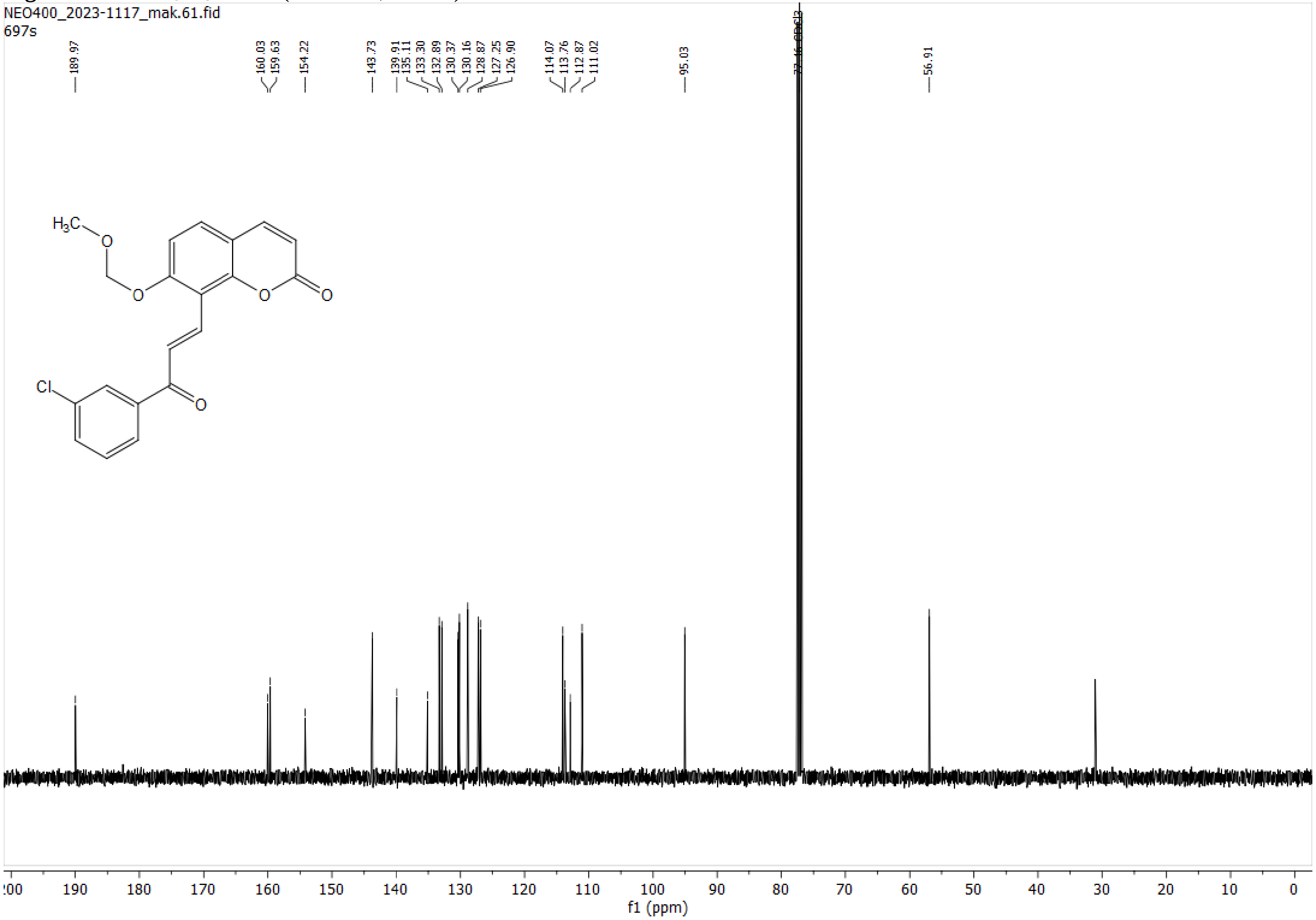

Figure S121: COSY (400 MHz, $CDCl_3$) of **13am**

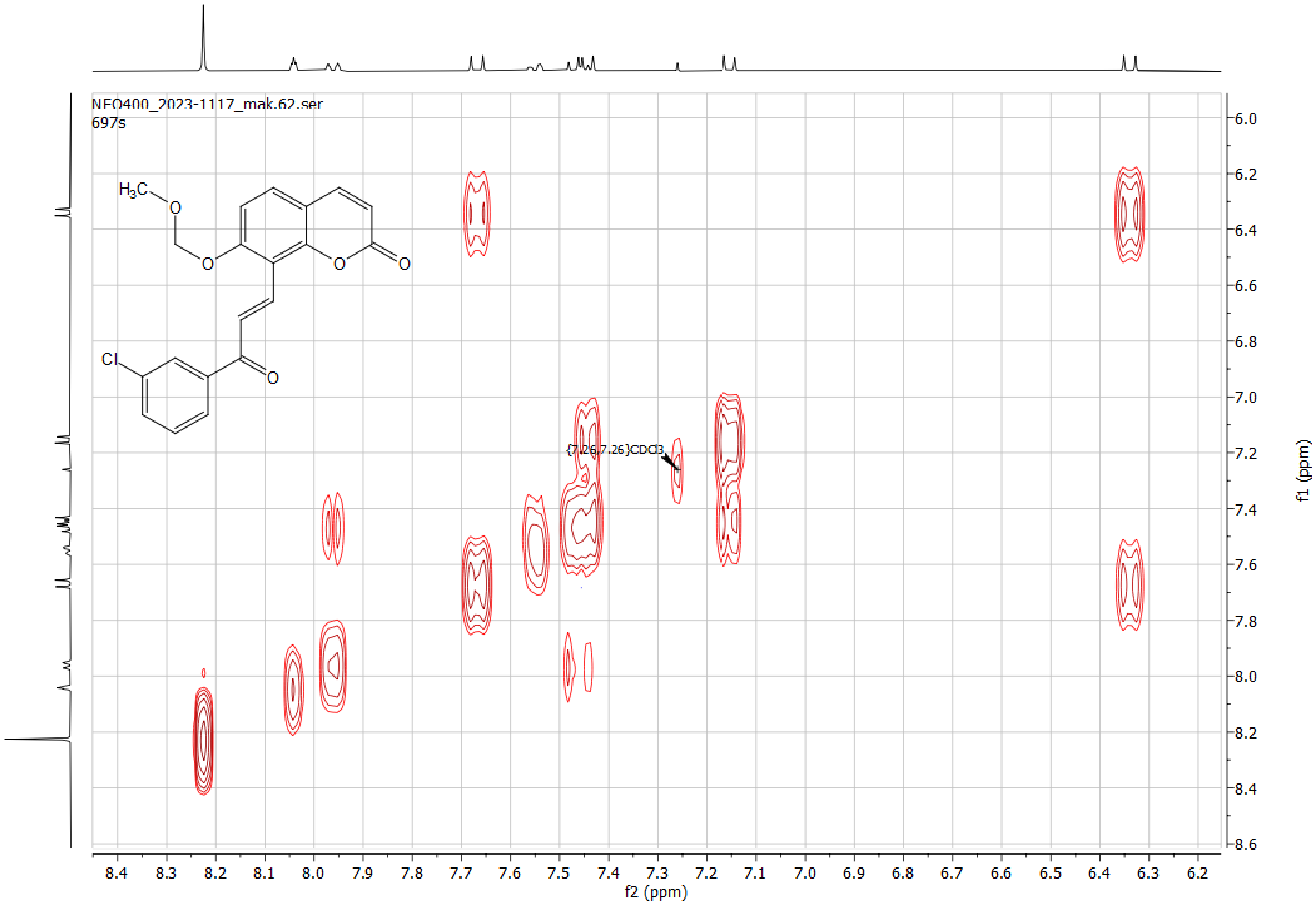

Figure S122: HSQC (400/100 MHz, $CDCl_3$) of **13am**

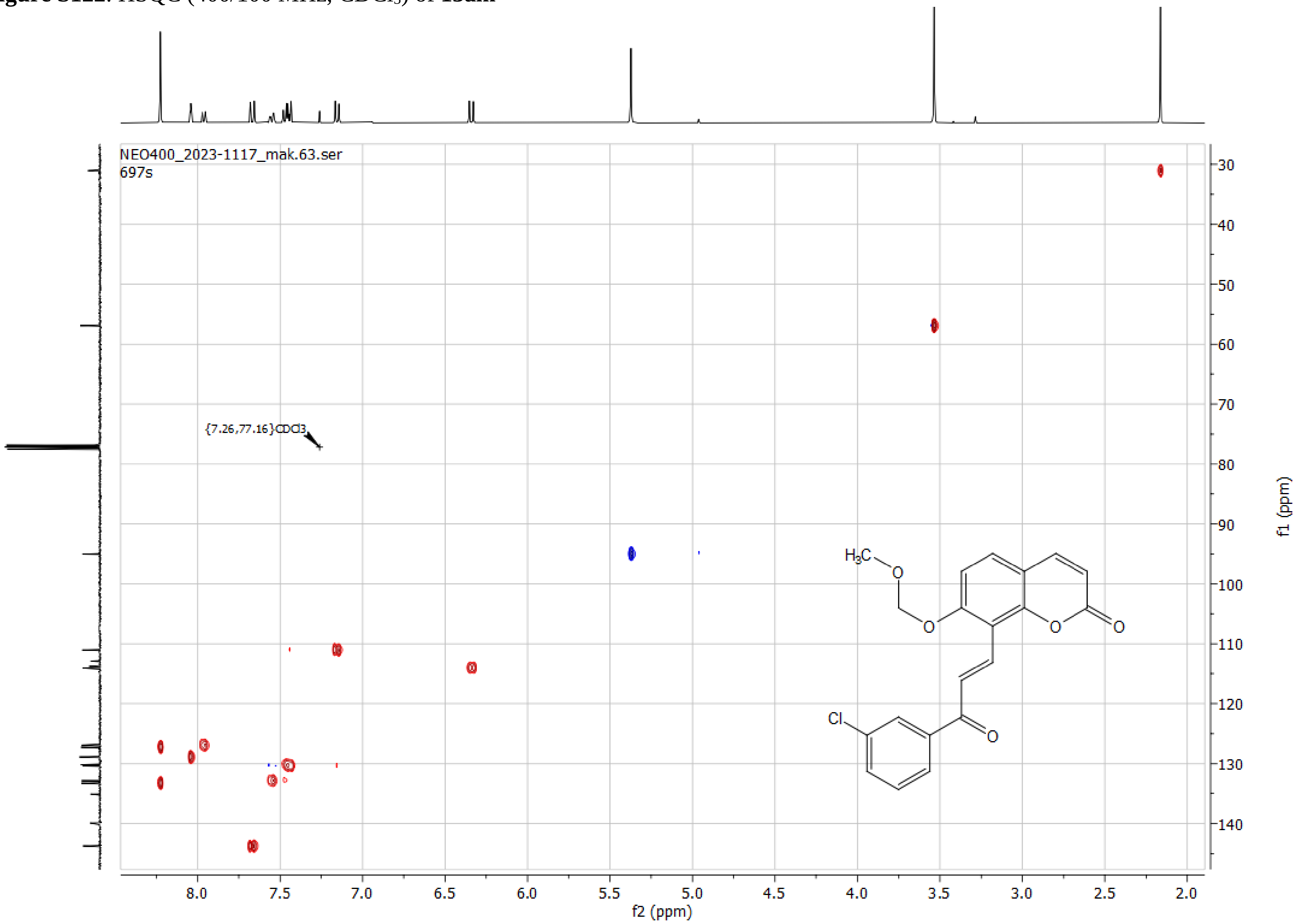

Figure S123: HMBC (400/100 MHz, $CDCl_3$) of **13am**

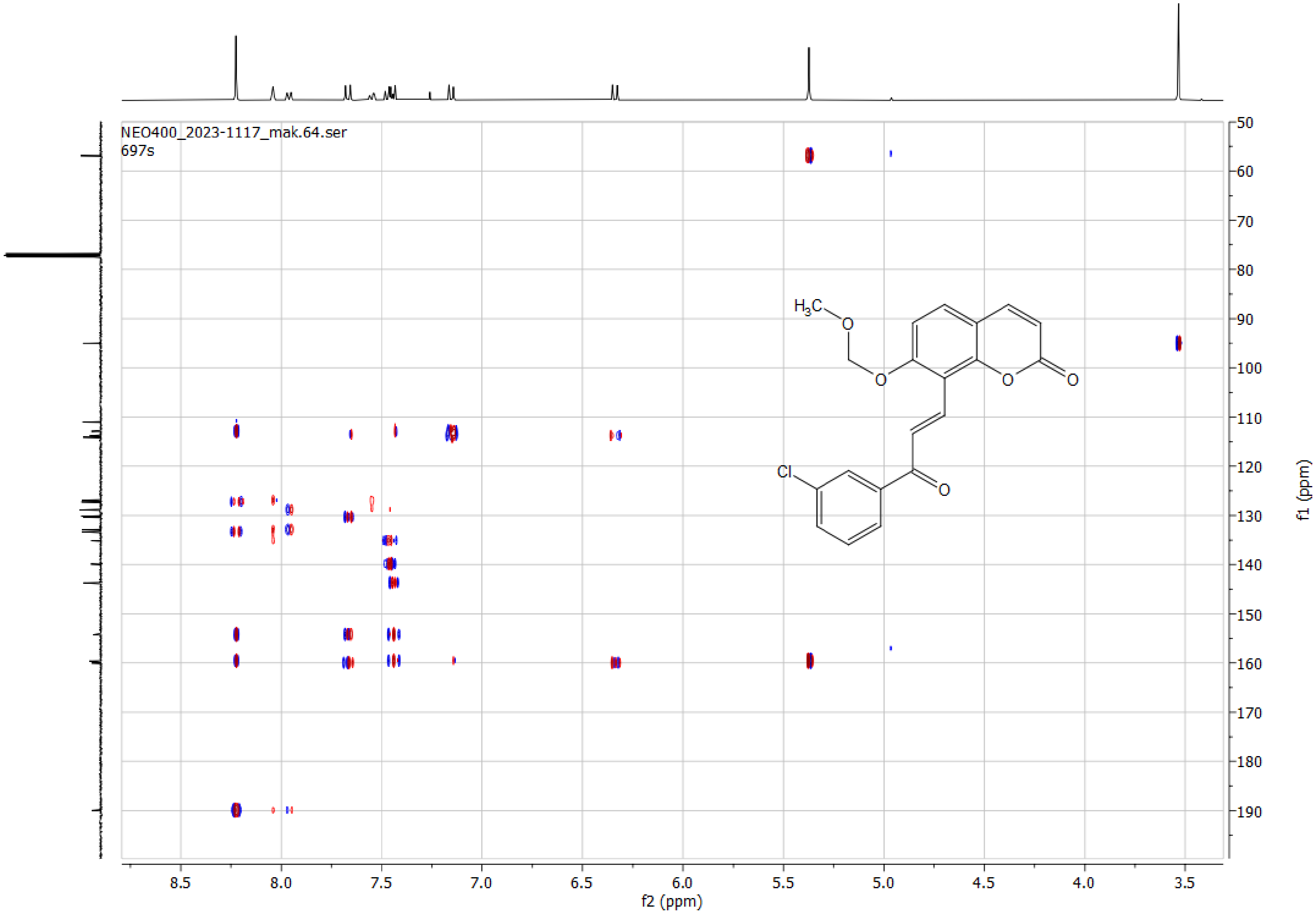

Figure S124: NOESY (400 MHz, $CDCl_3$) of **13am**

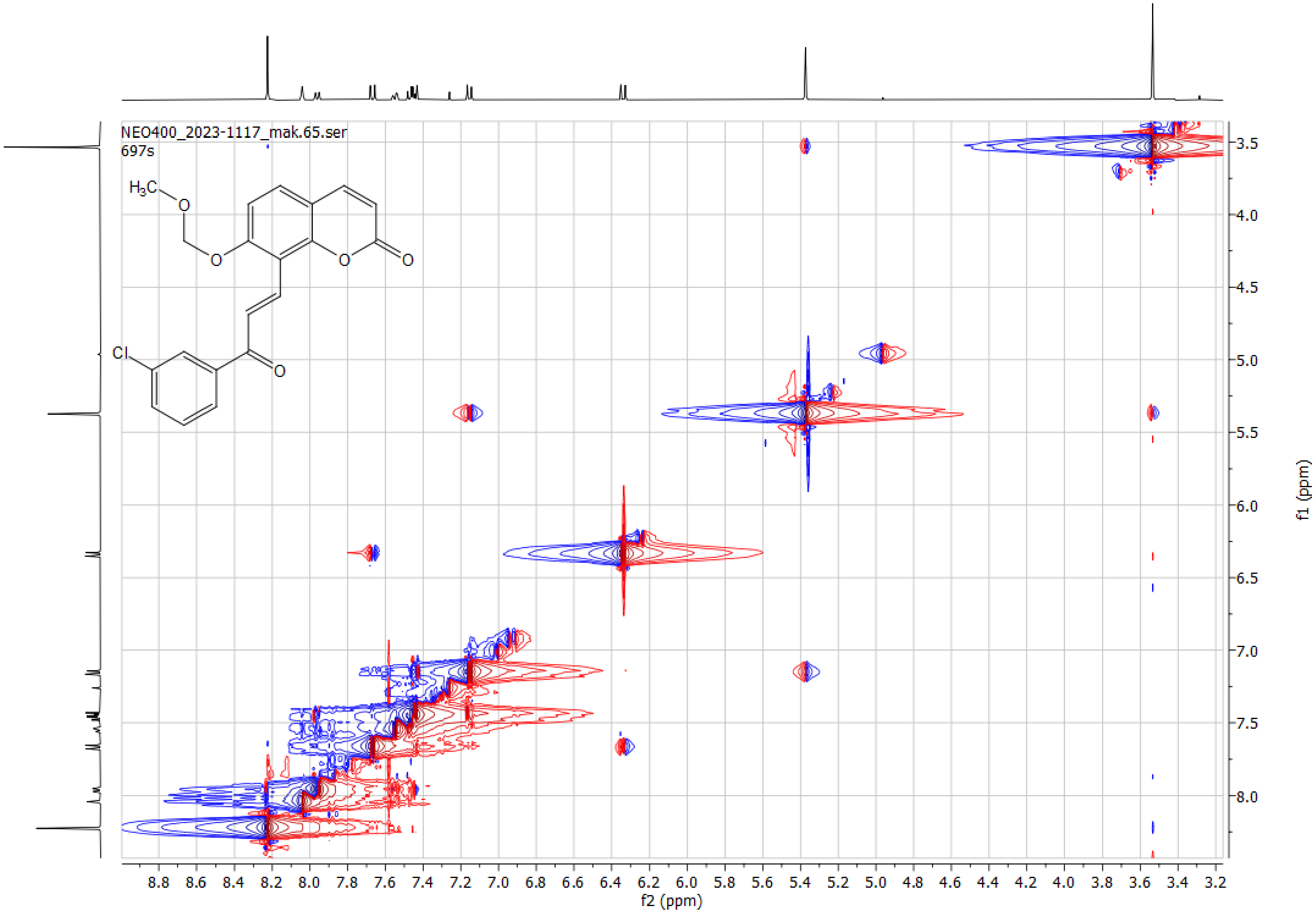

Figure S125: NMR-Signal assignment and selected HMBC-couplings for compound **14an**

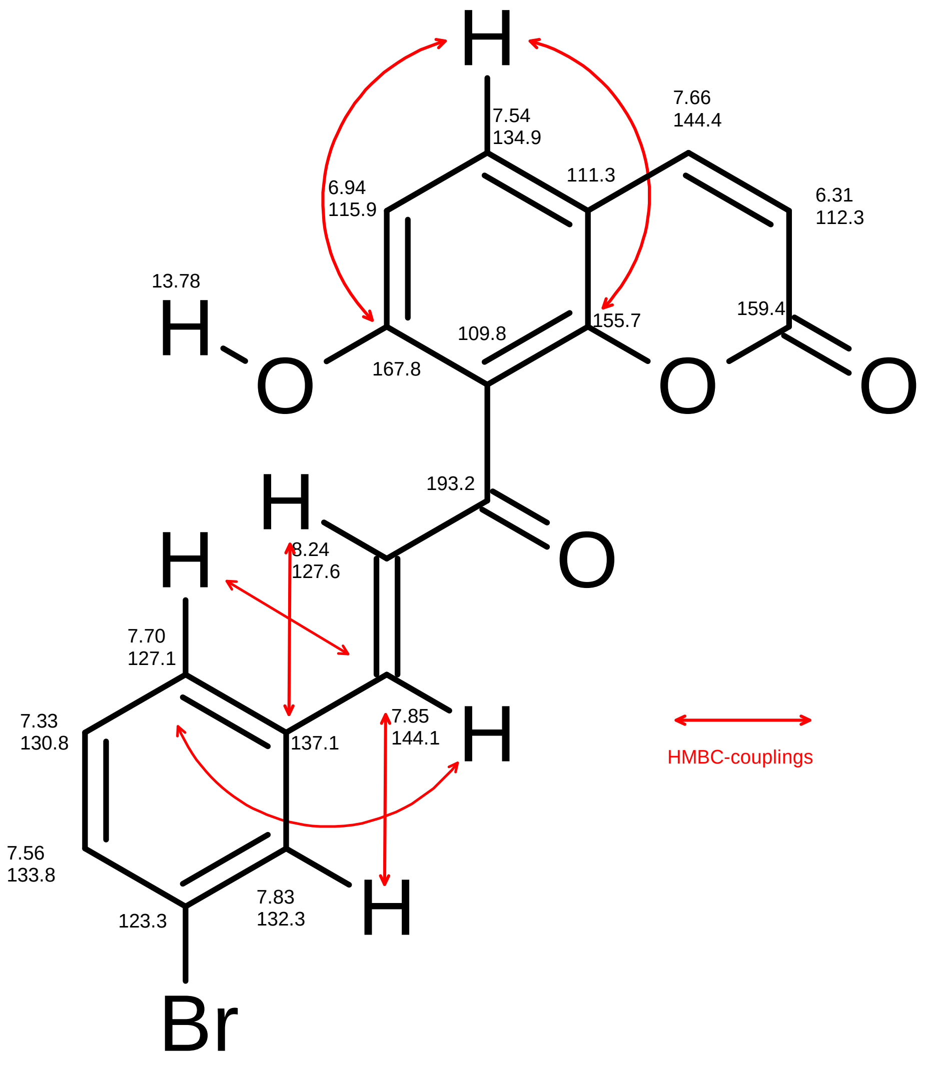

Figure S126: ^{1}H NMR (400 MHz, $CDCl_3$) of **14an**

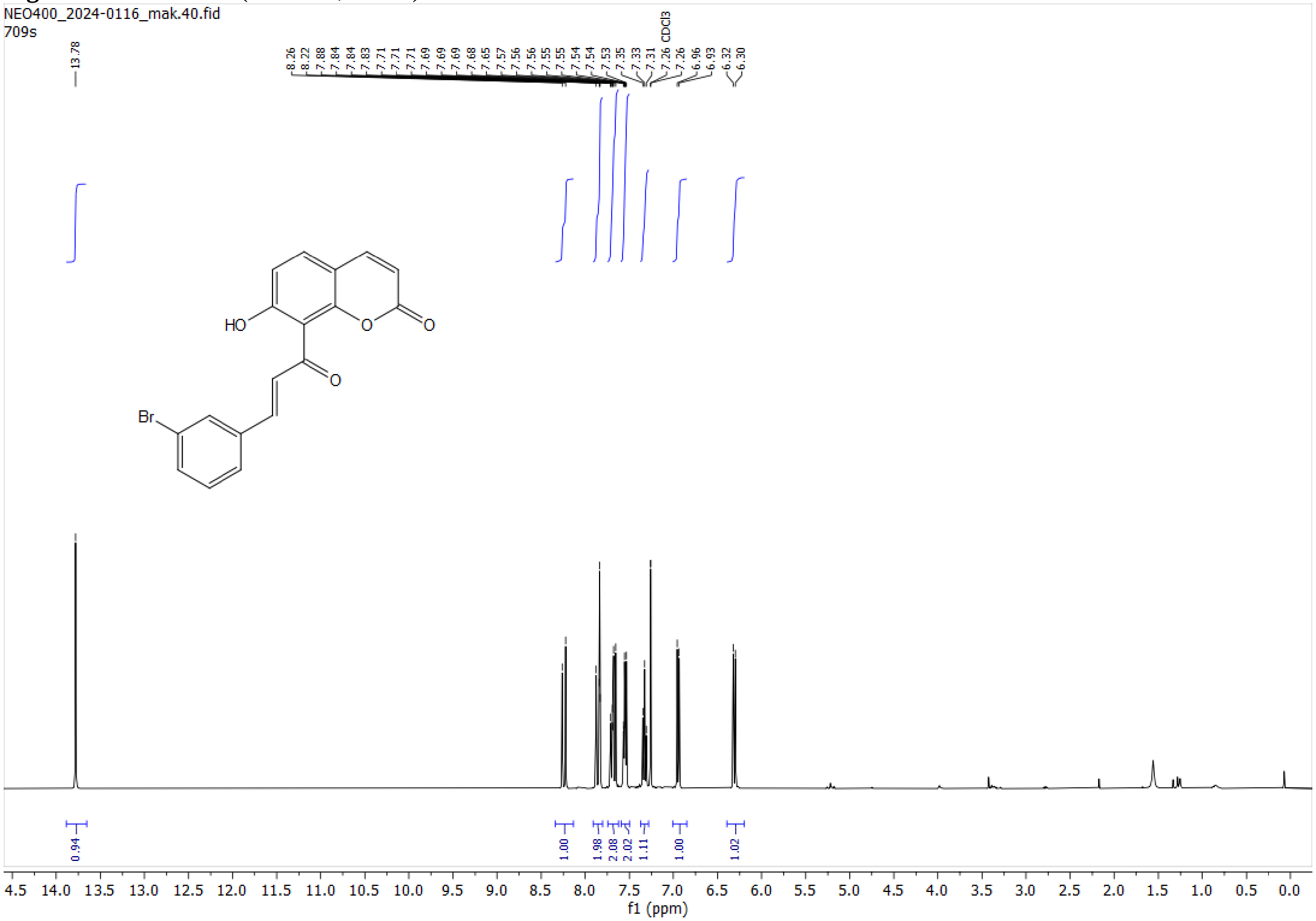

Figure S127: $^{13}C\{^{1}H\}$ NMR (100 MHz, $CDCl_3$) of **14an**

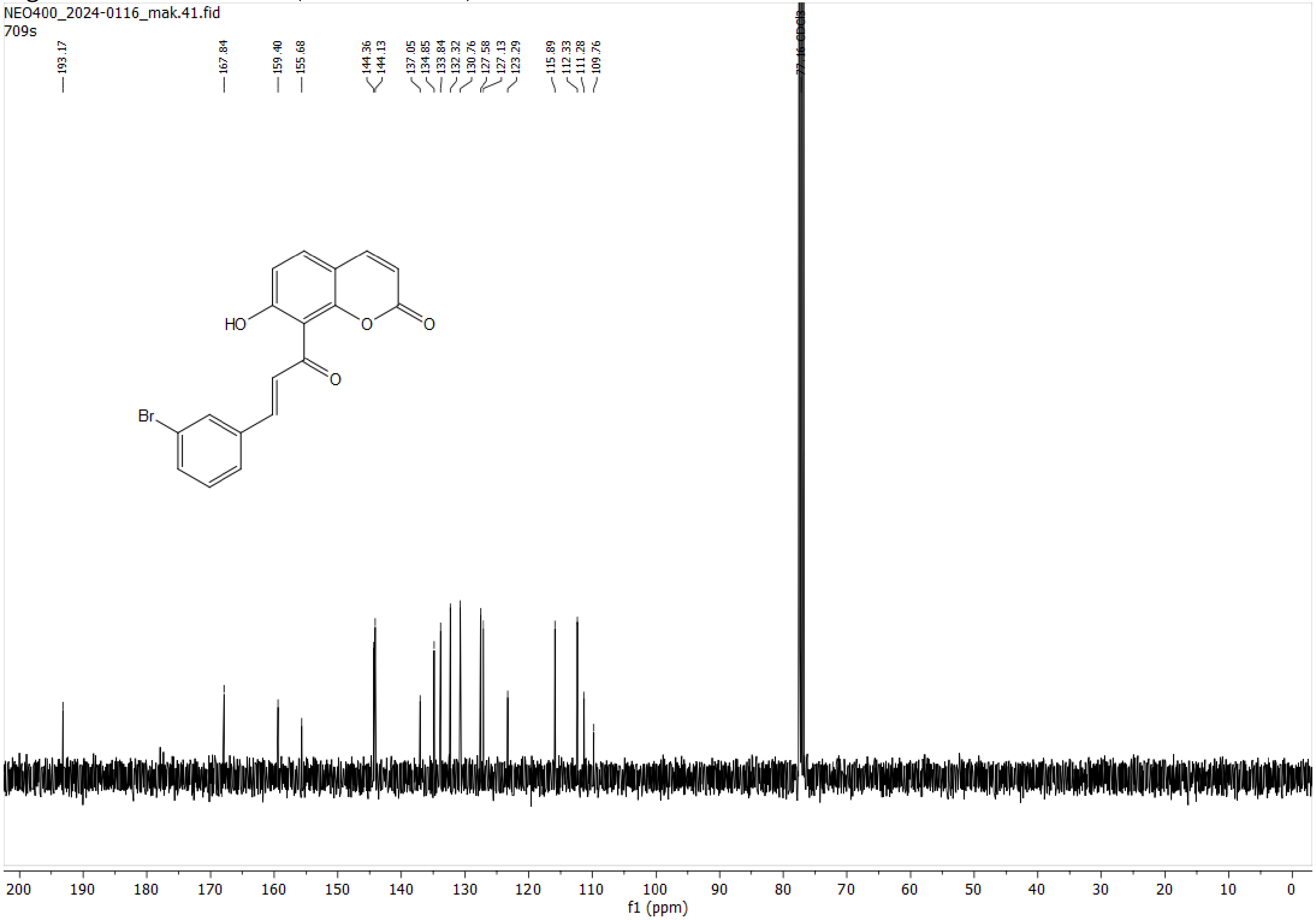

Figure S128: HSQC (400/100 MHz, $CDCl_3$) of **14an**

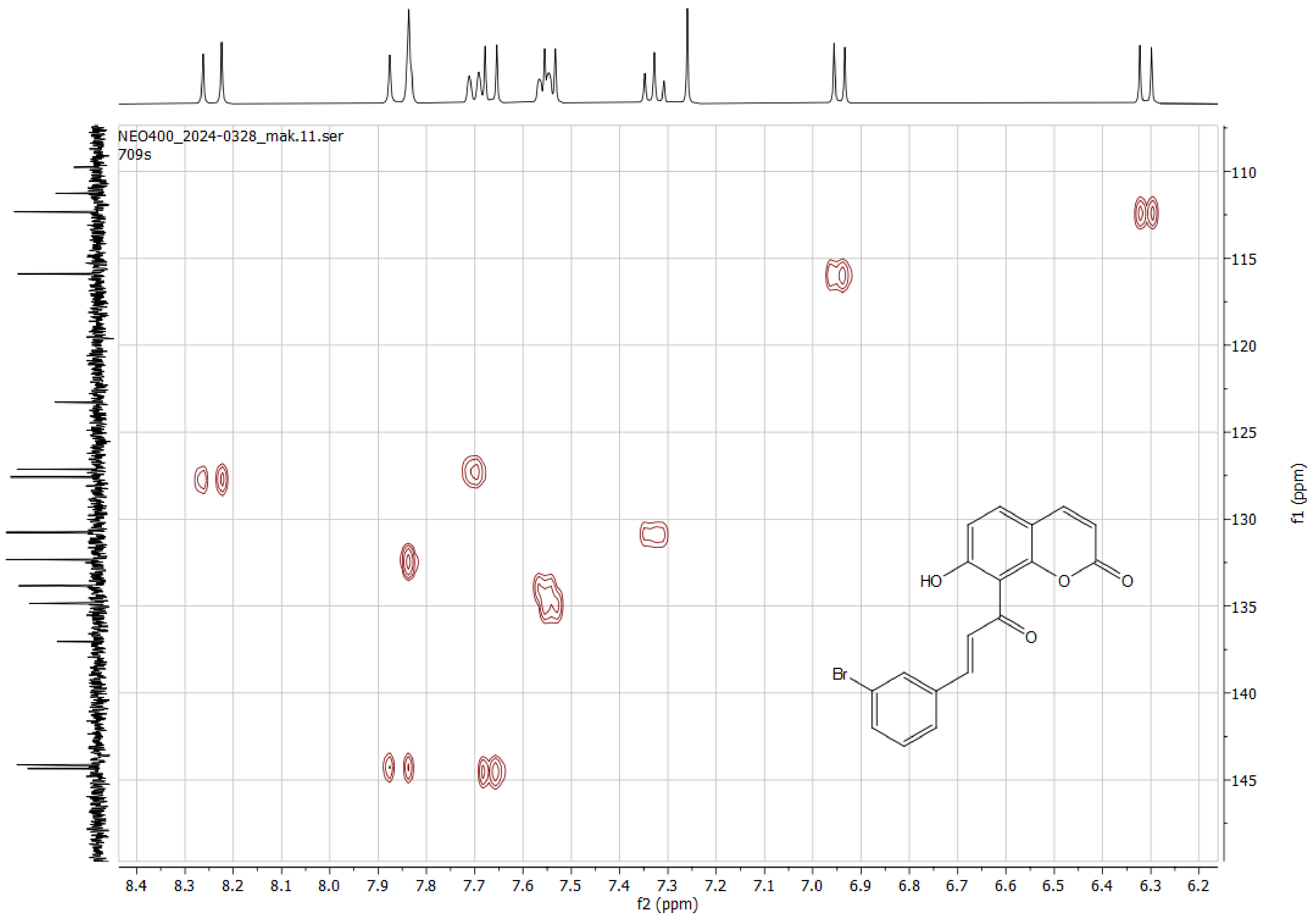

Figure S129: HMBC (400/100 MHz, $CDCl_3$) of **14an**

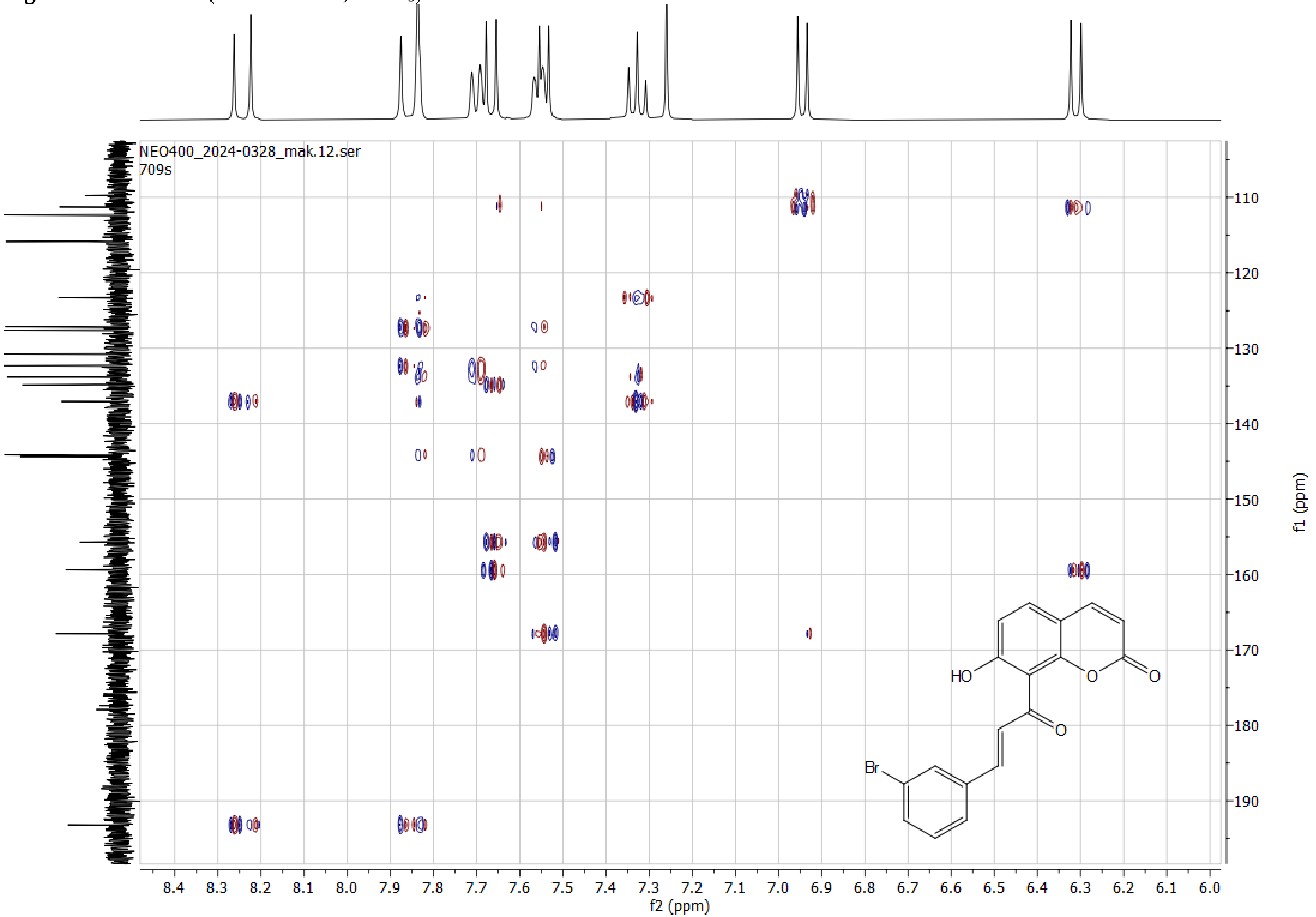

Figure S130: ^{1}H NMR (400 MHz, $CDCl_3$) of **13ap**

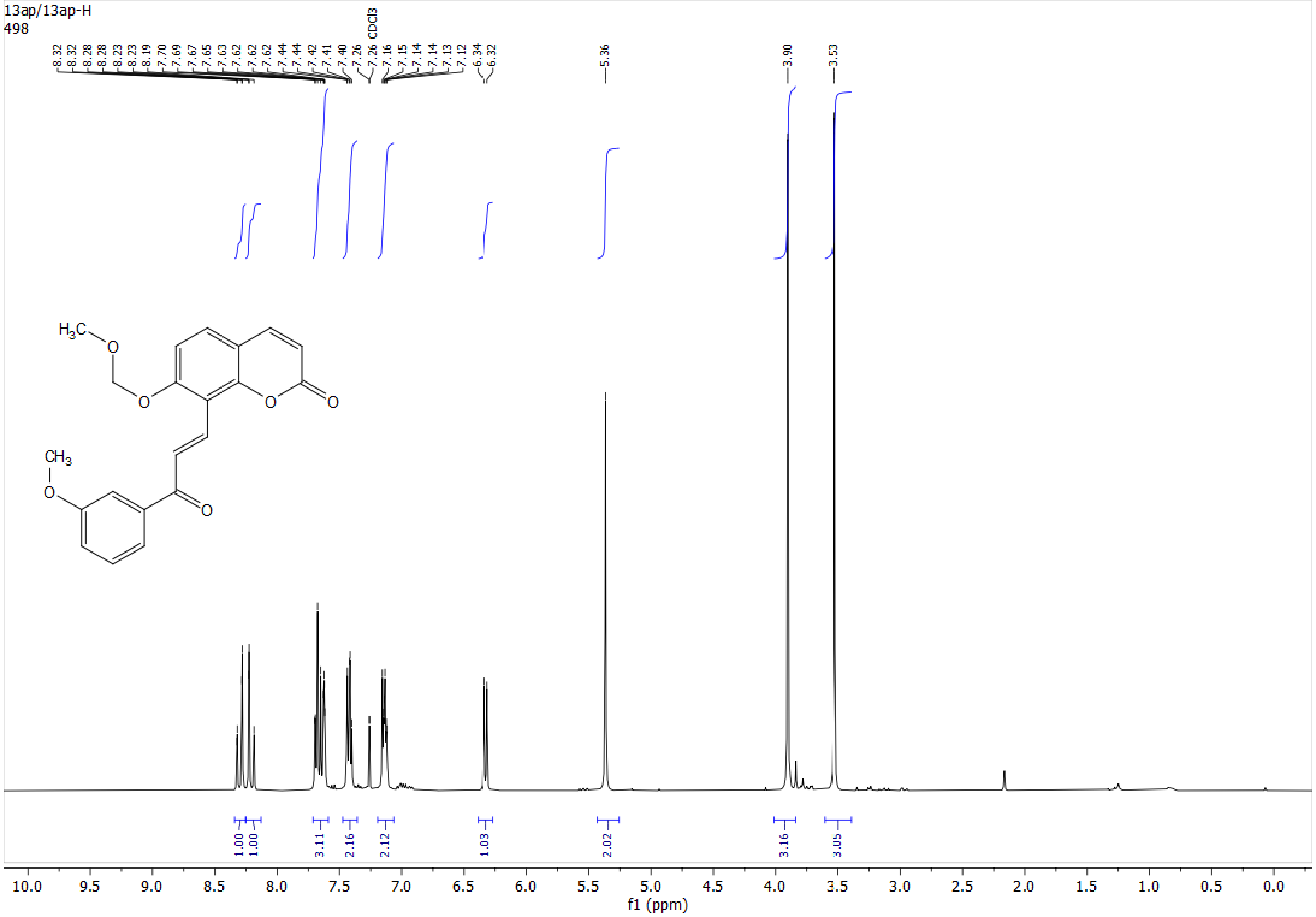

Figure S131: $^{13}C\{^1H\}$ NMR (100 MHz, $CDCl_3$) of **13ap**

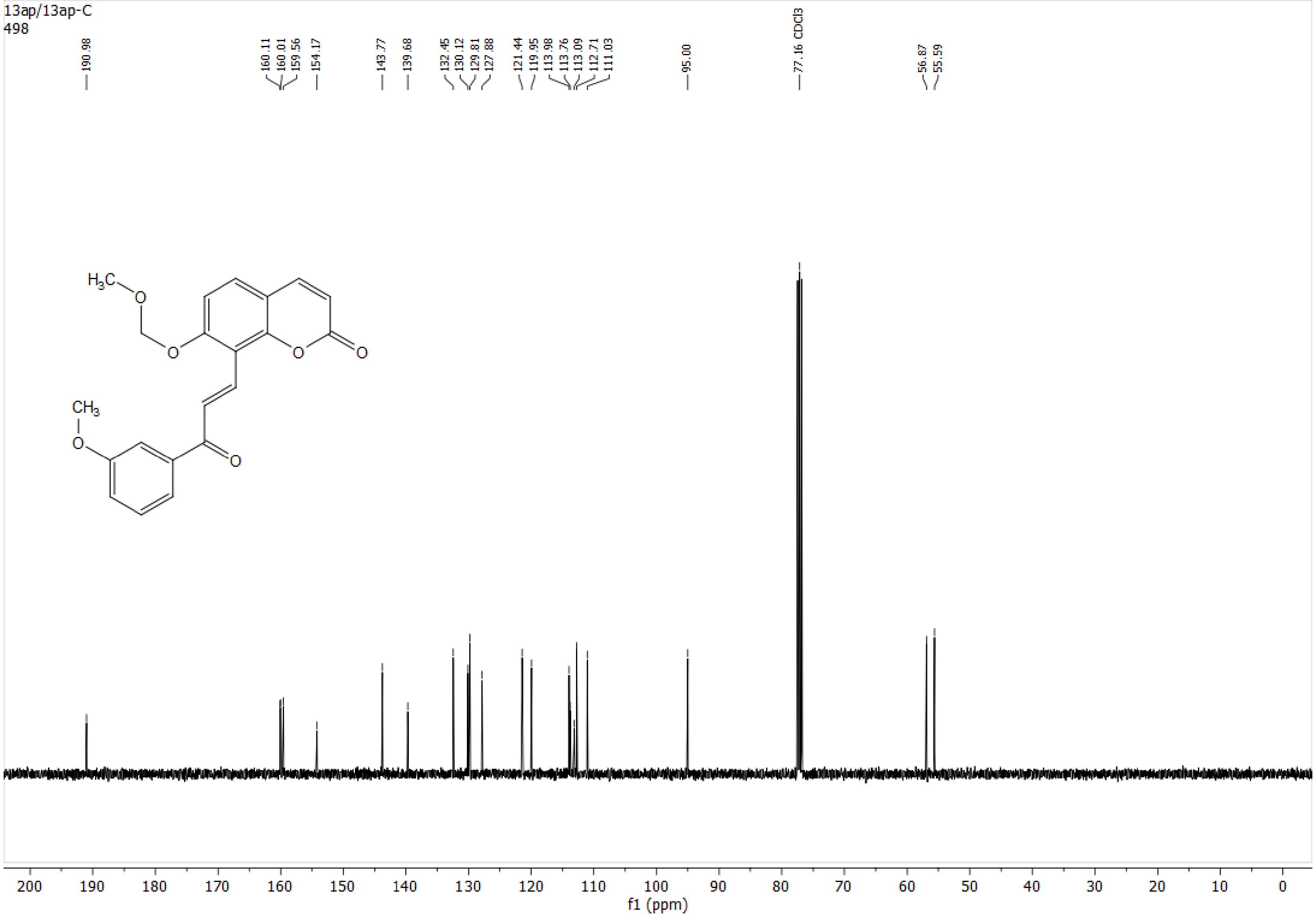

Figure S132: ^{1}H NMR (400 MHz, $CDCl_3$) of **13aq**

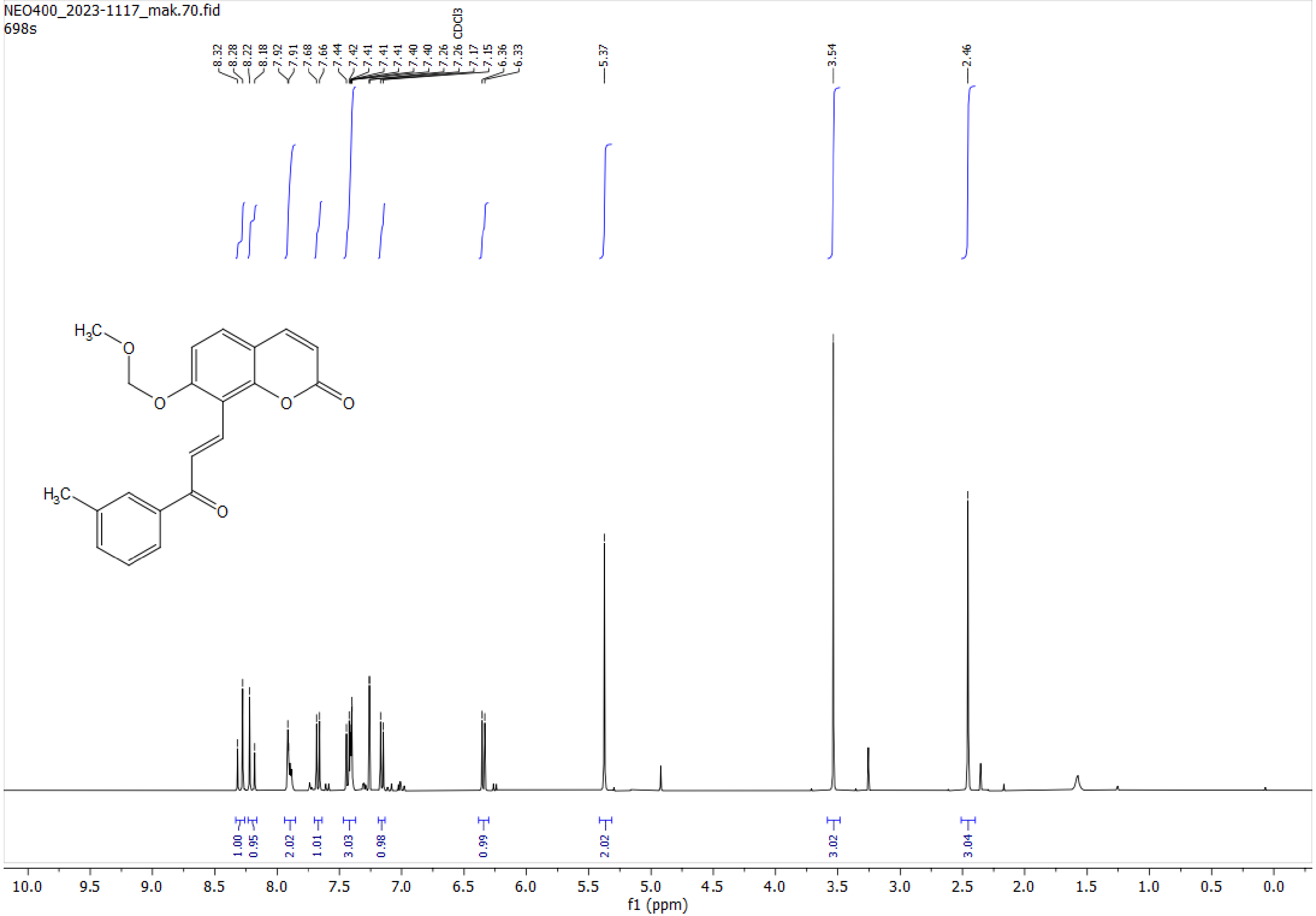

Figure S133: $^{13}C\{^1H\}$ NMR (100 MHz, $CDCl_3$) of **13aq**

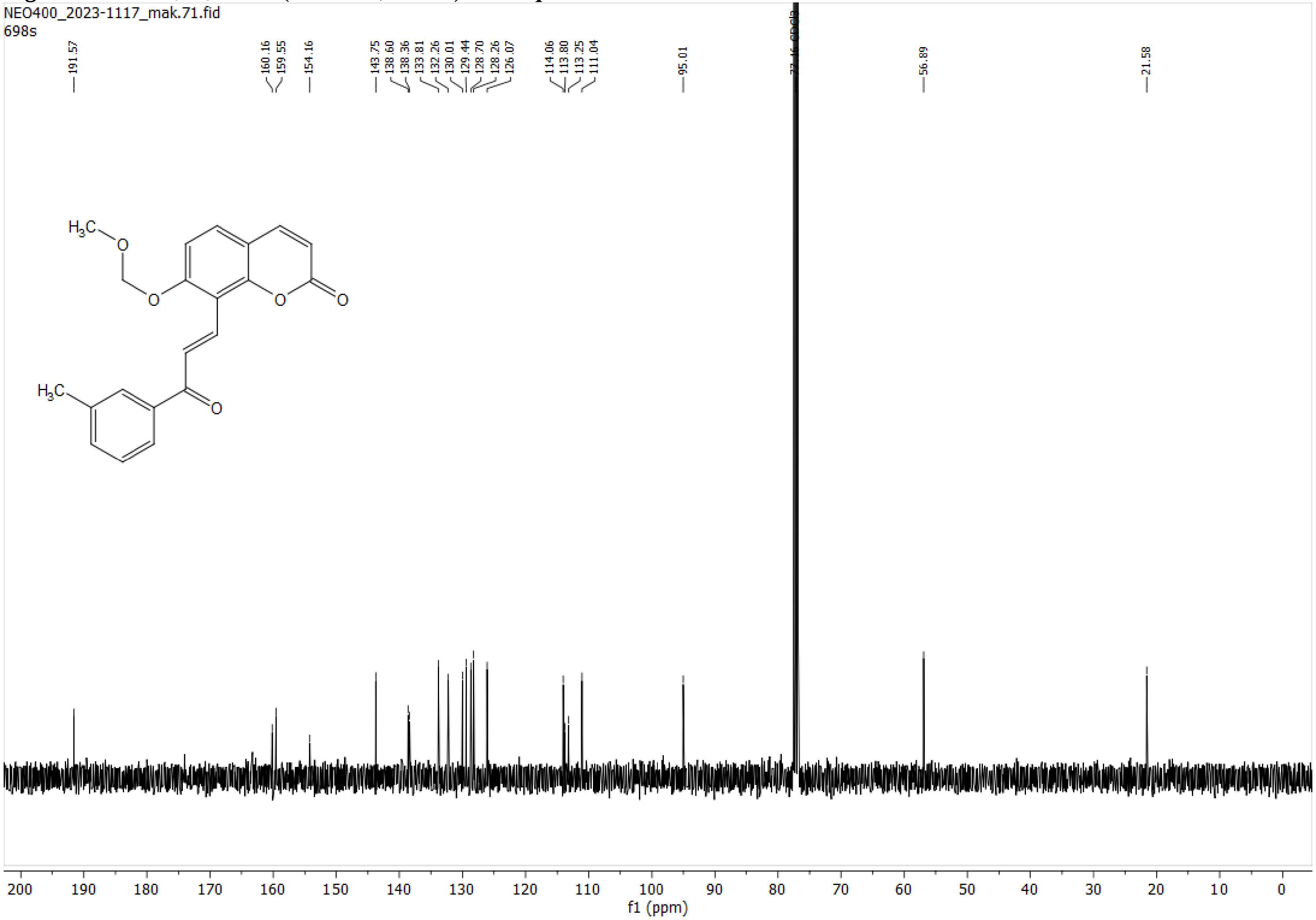

Figure S134: COSY (400 MHz, $CDCl_3$) of **13aq**

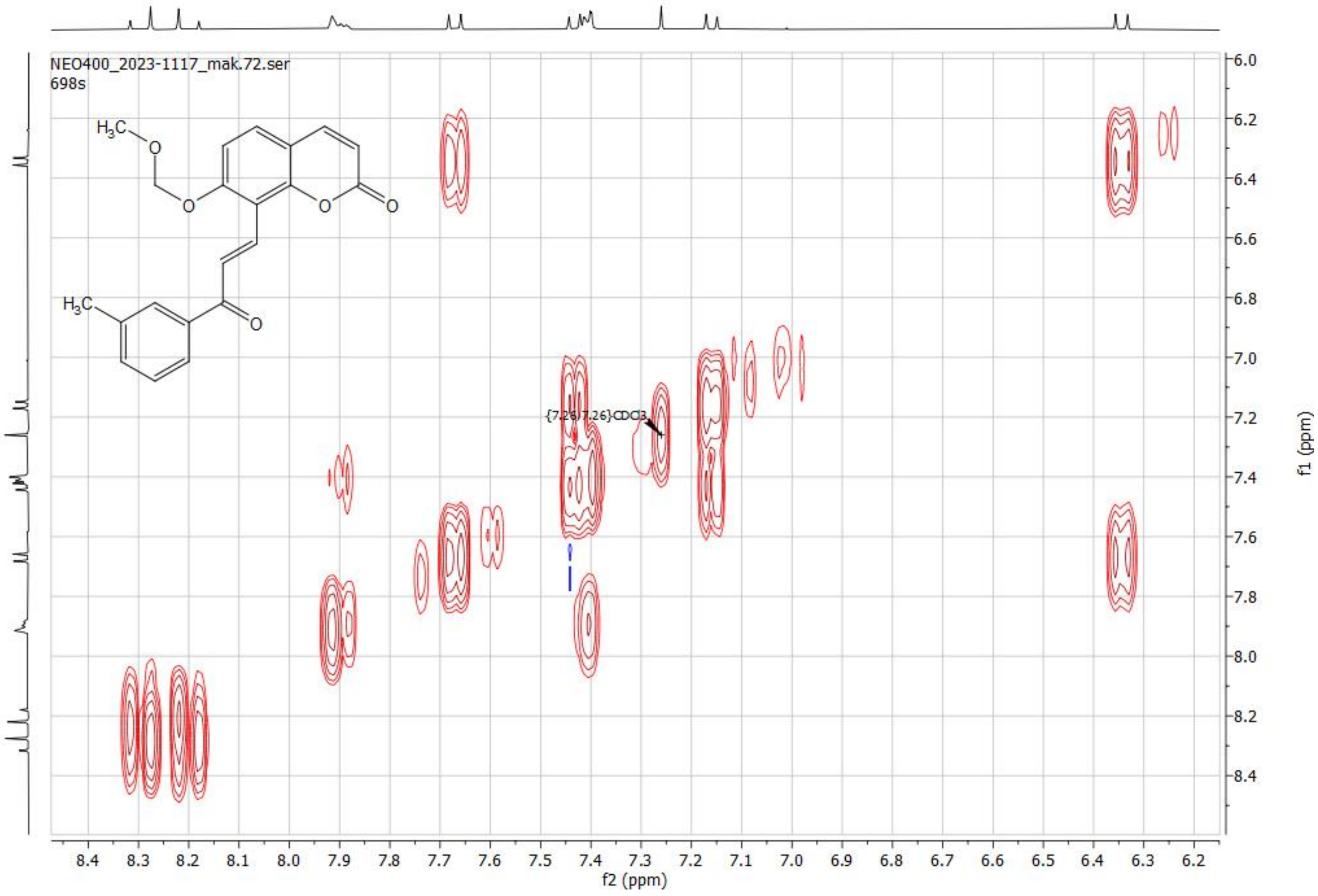

Figure S135: HSQC (400/100 MHz, $CDCl_3$) of **13aq**

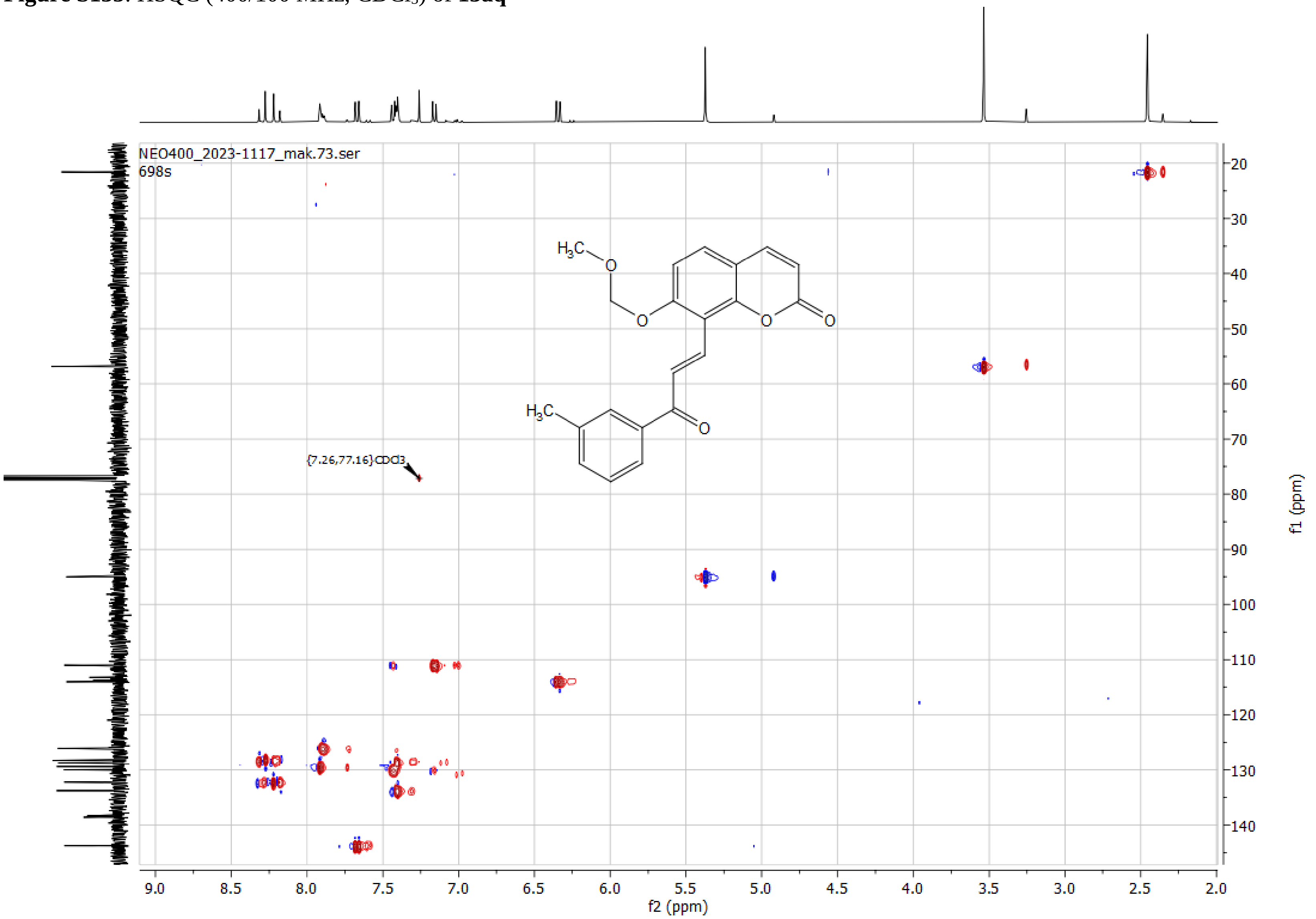

Figure S136: HMBC (400/100 MHz, $CDCl_3$) of **13aq**

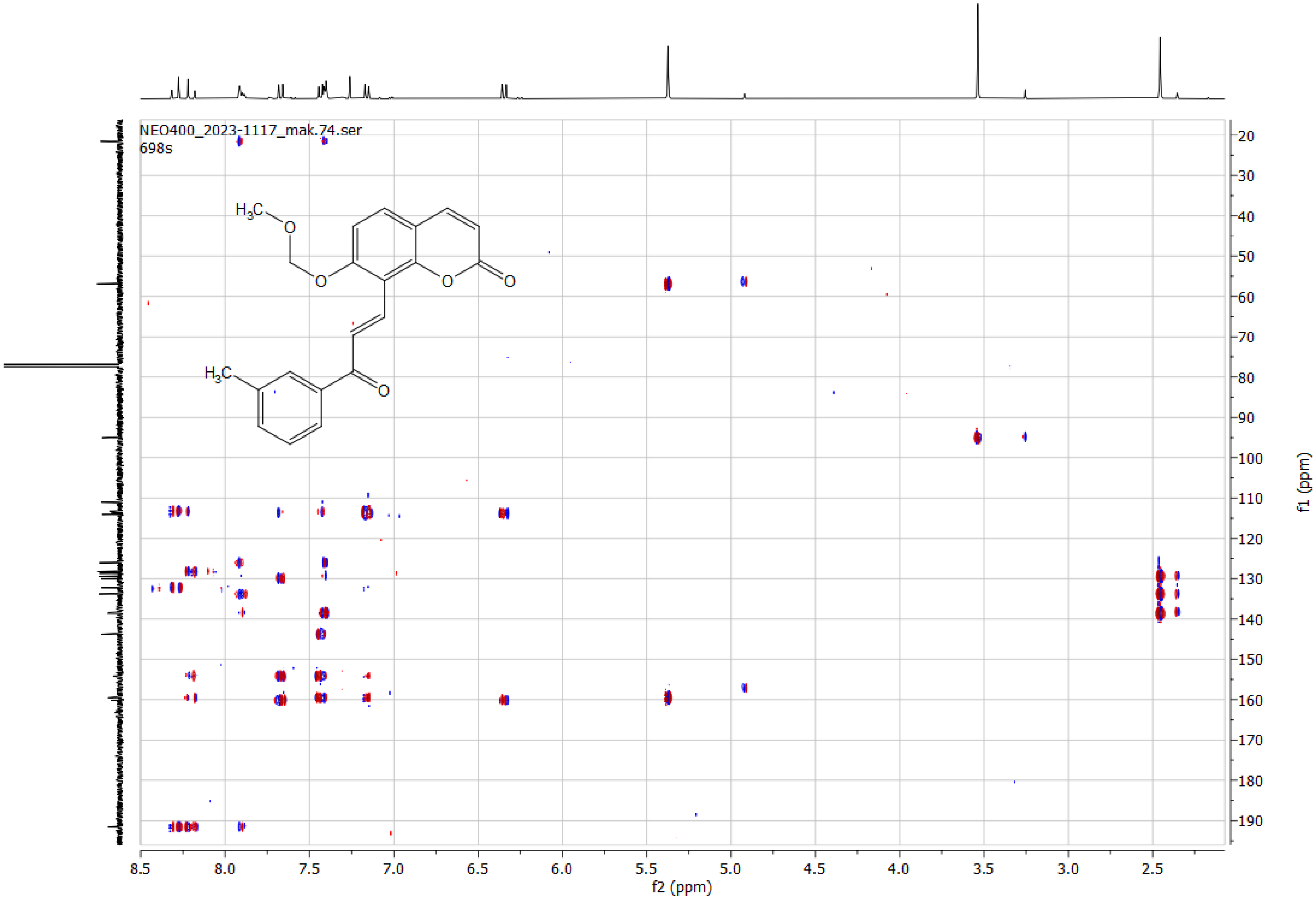

Figure S137: NOESY (400 MHz, $CDCl_3$) of **13aq**

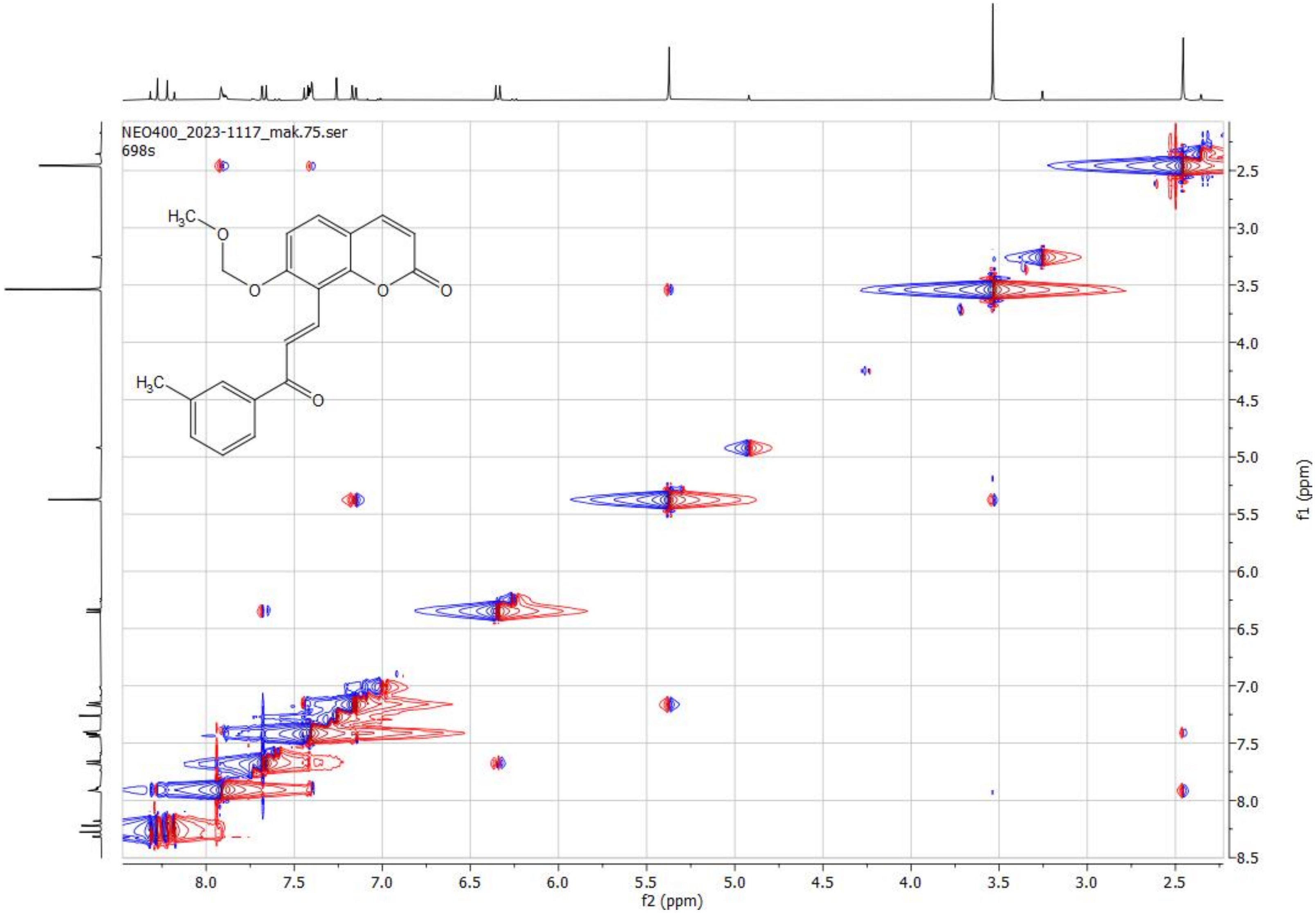

Figure S138: NMR-Signal assignment and selected HMBC-couplings for compound **14ar**

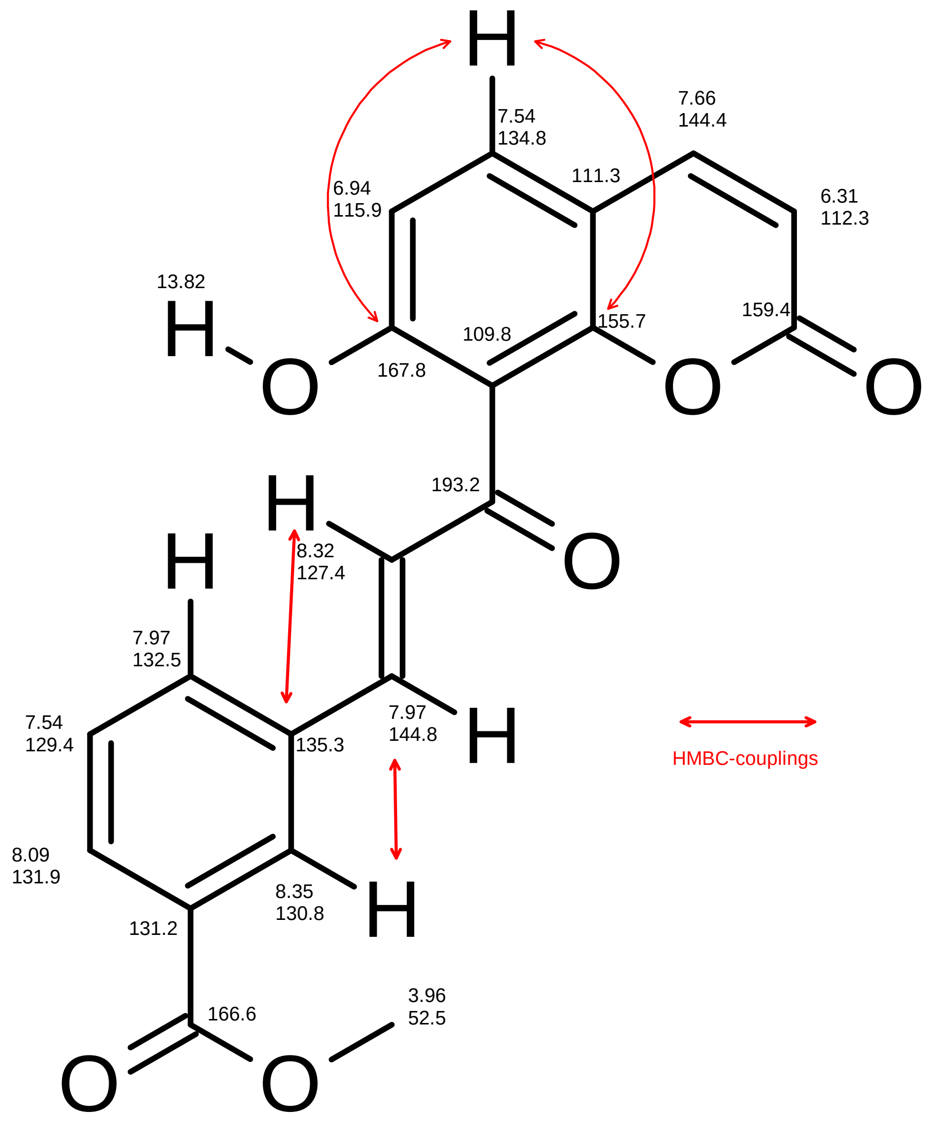

Figure S139: ^{1}H NMR (400 MHz, $CDCl_3$) of **14ar**

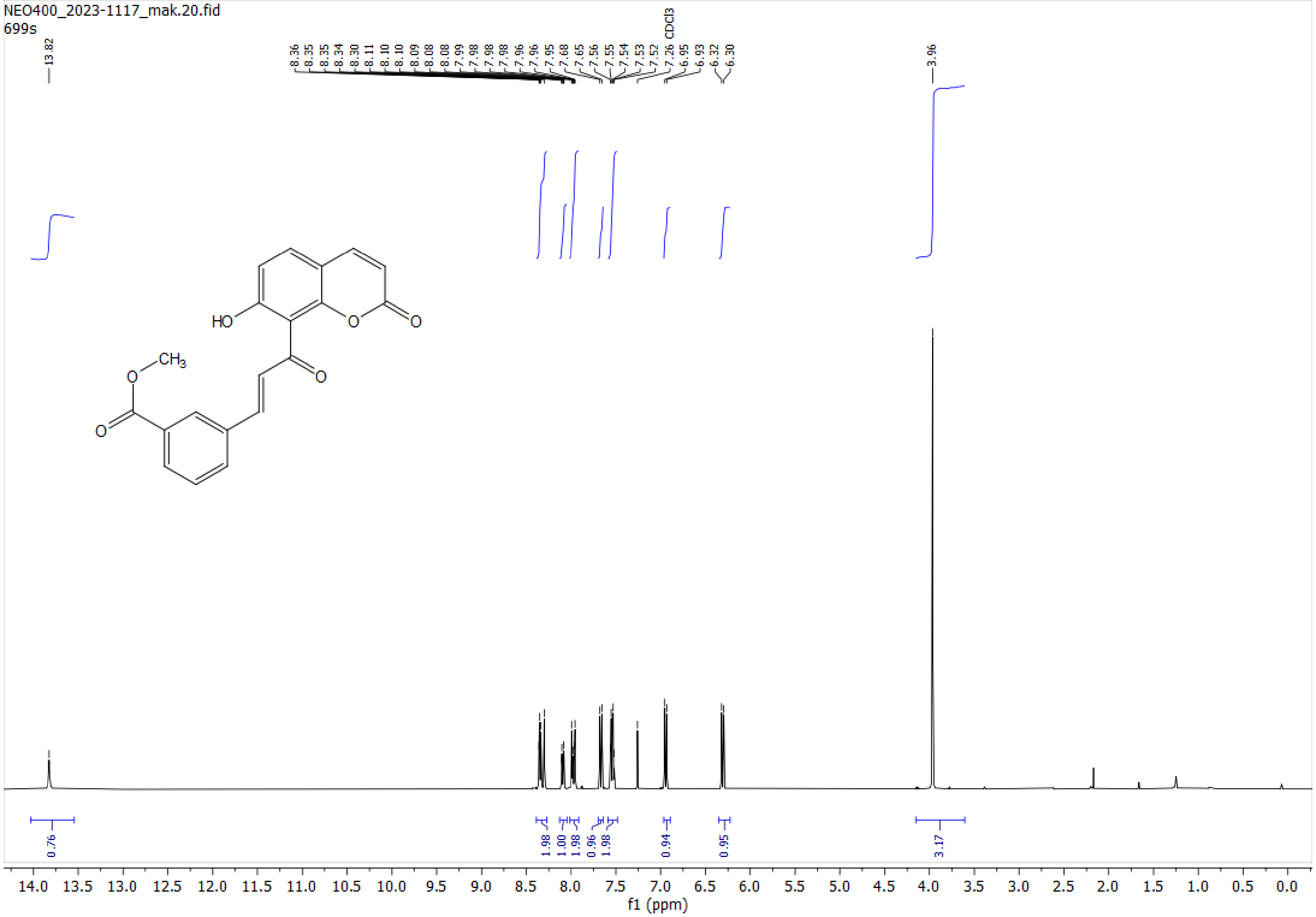

Figure S140: $^{13}C\{^1H\}$ NMR (100 MHz, $CDCl_3$) of **14ar**

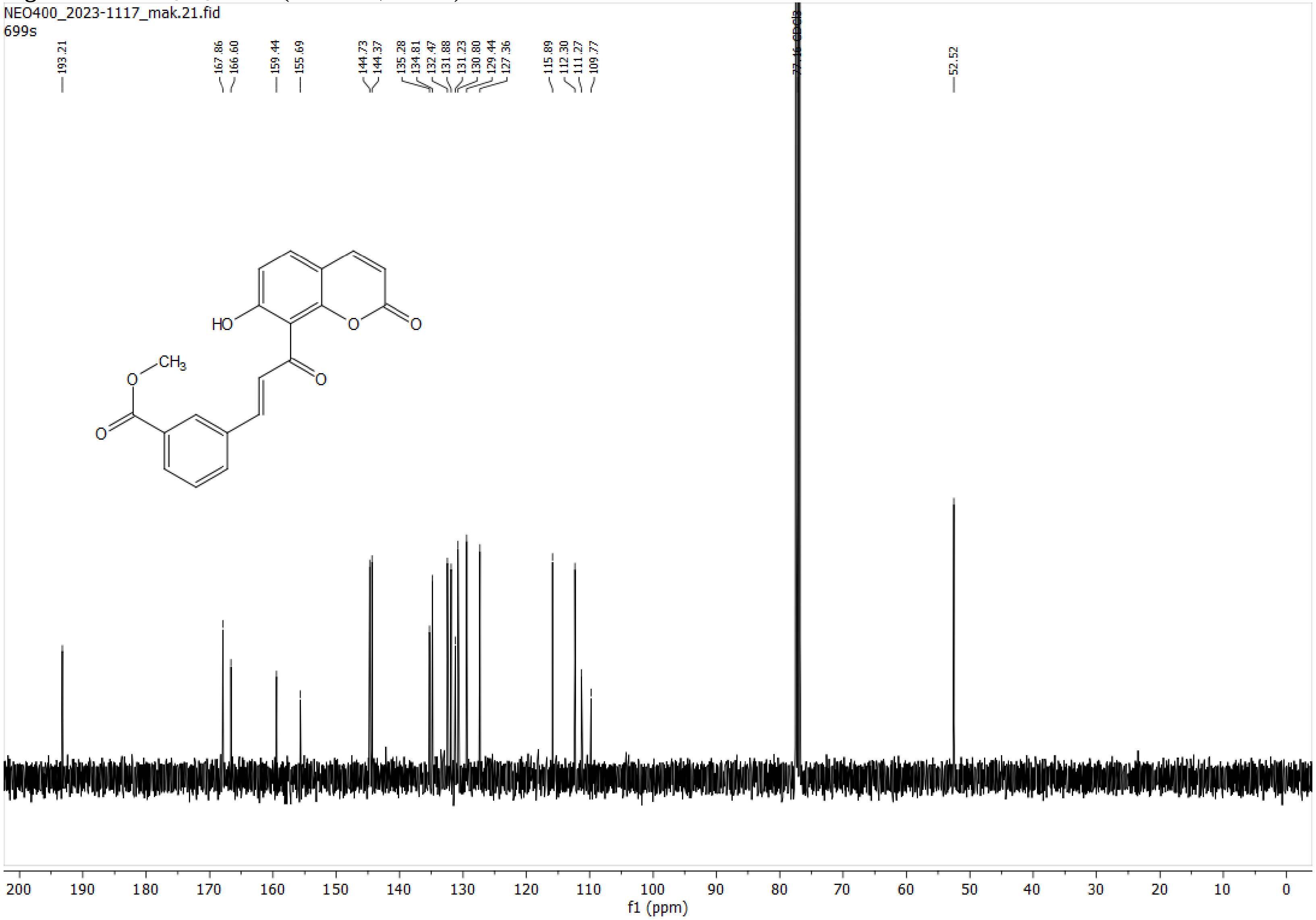

Figure S141: COSY (400 MHz, $CDCl_3$) of **14ar**

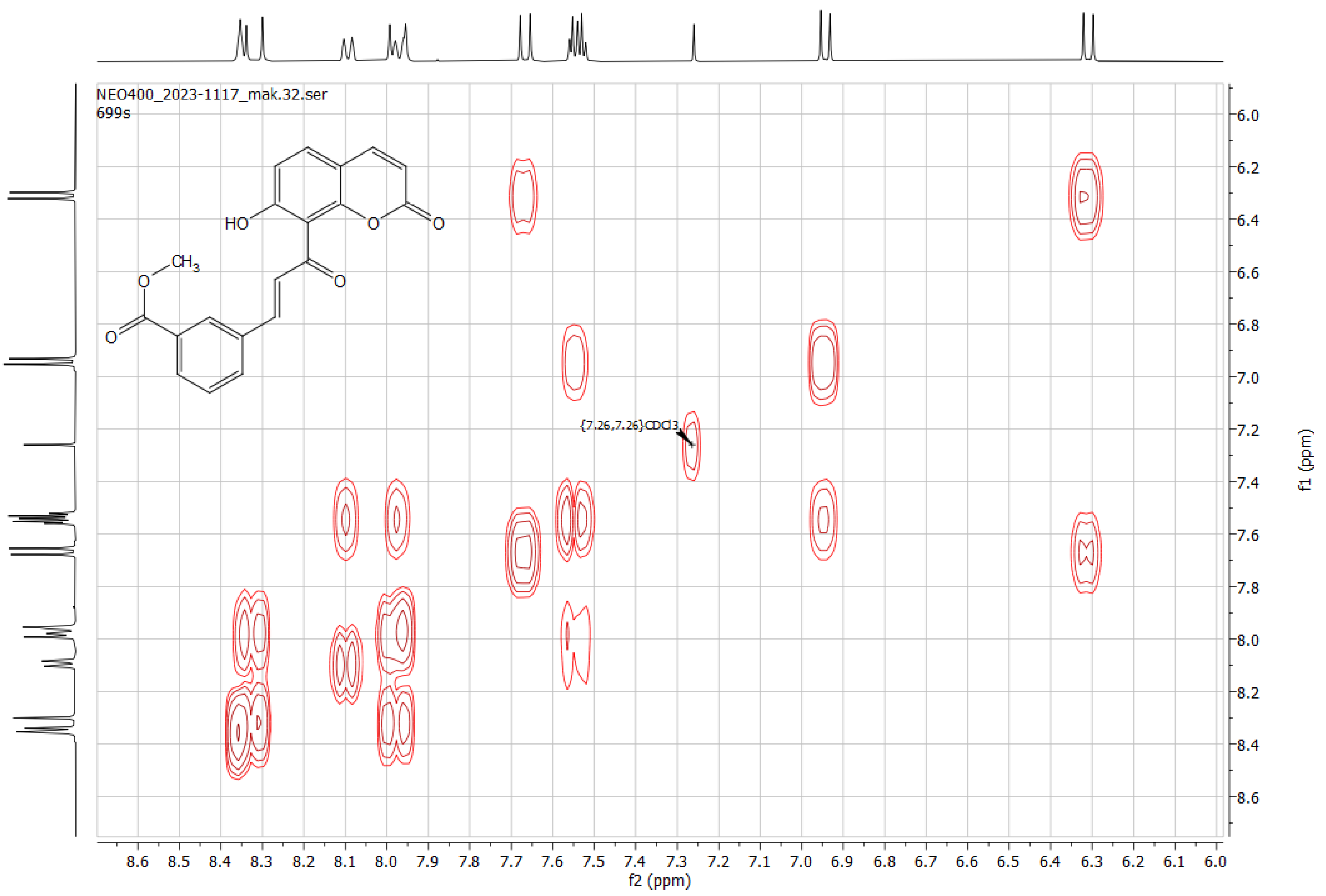

Figure S142: HSQC (400/100 MHz, $CDCl_3$) of **14ar**

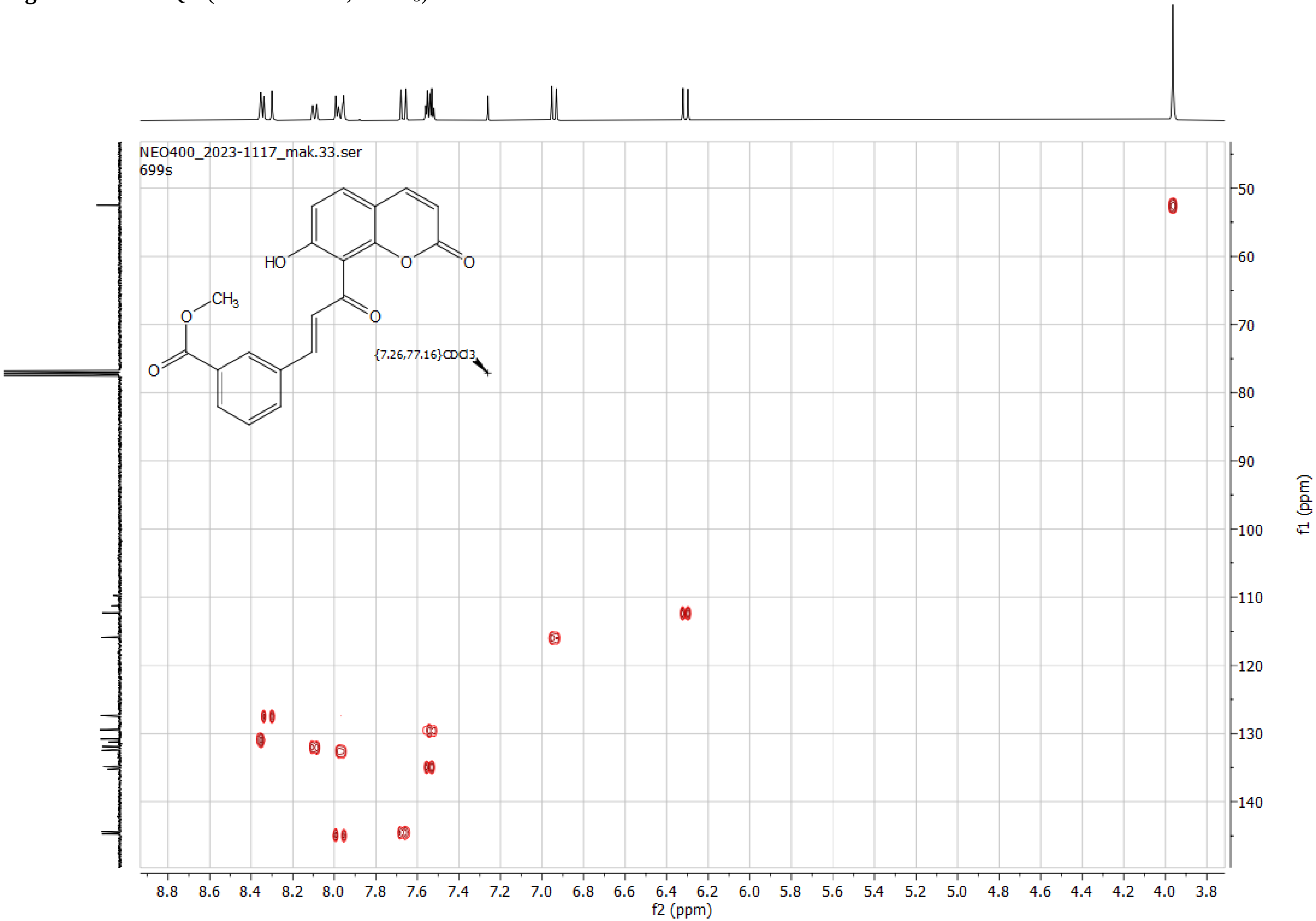

Figure S143: HMBC (400/100 MHz, $CDCl_3$) of **14ar**

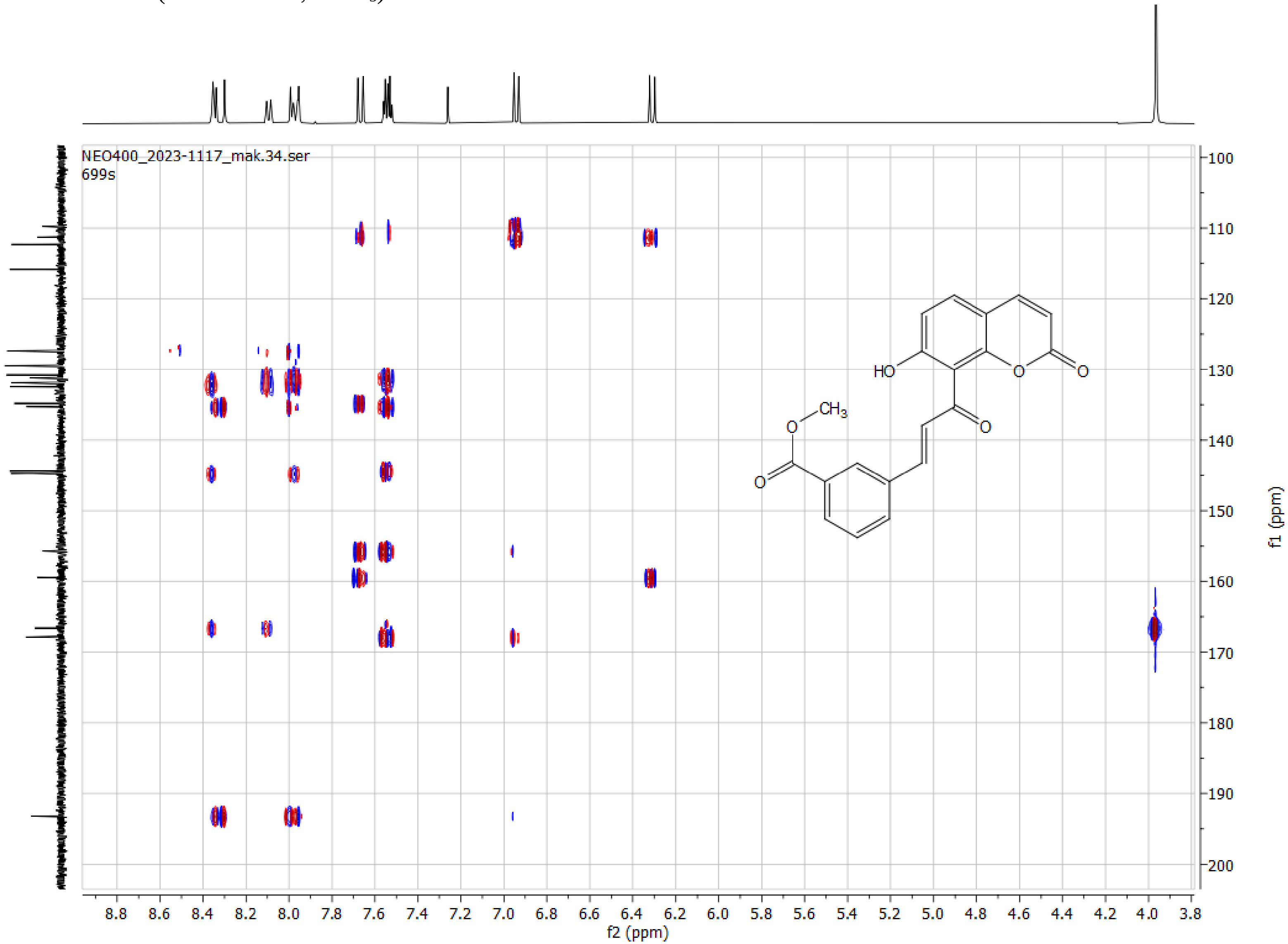

Figure S144: NOESY (400 MHz, $CDCl_3$) of **14ar**

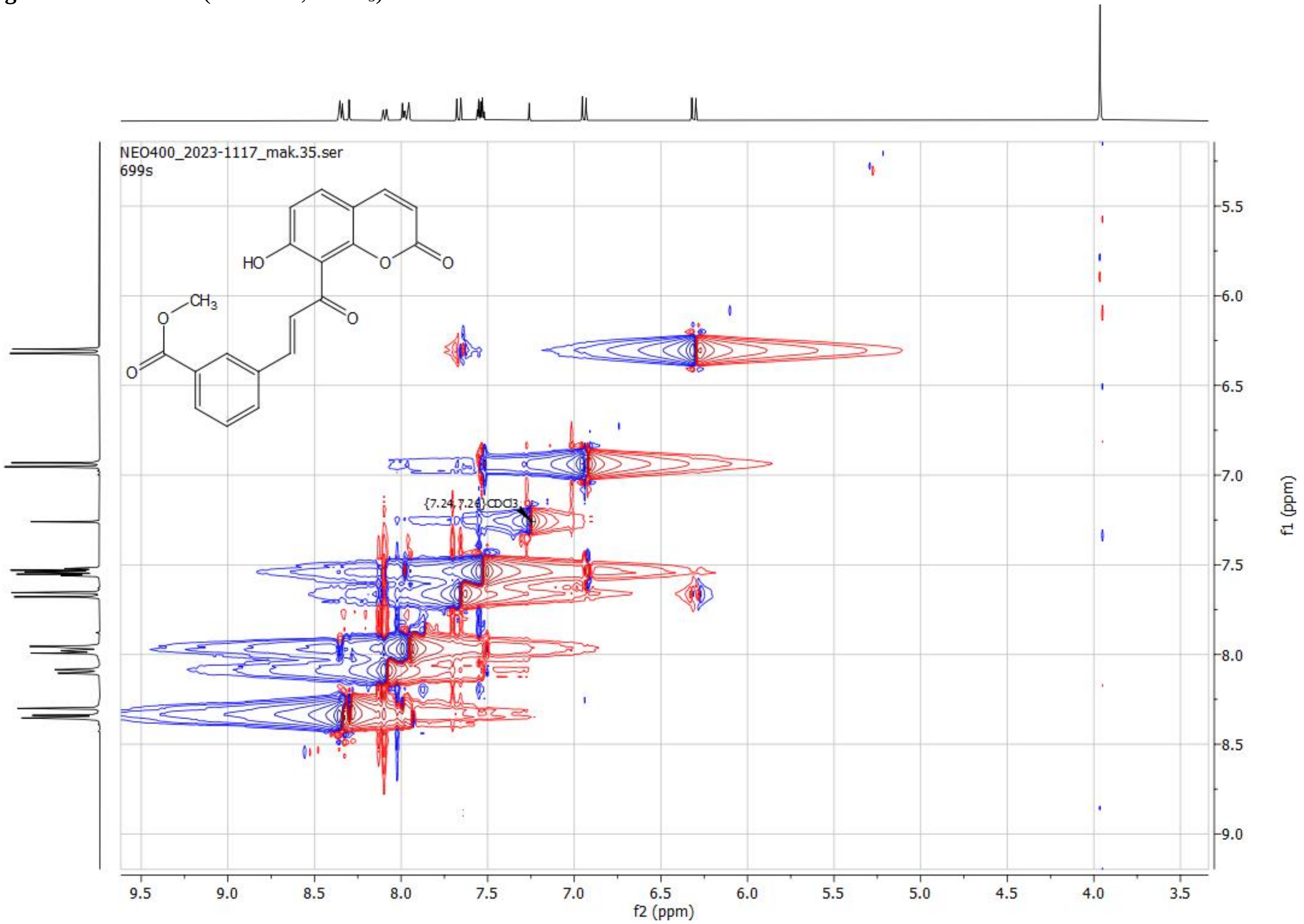

Figure S145: NMR-Signal assignment and selected HMBC-couplings for compound **13at**

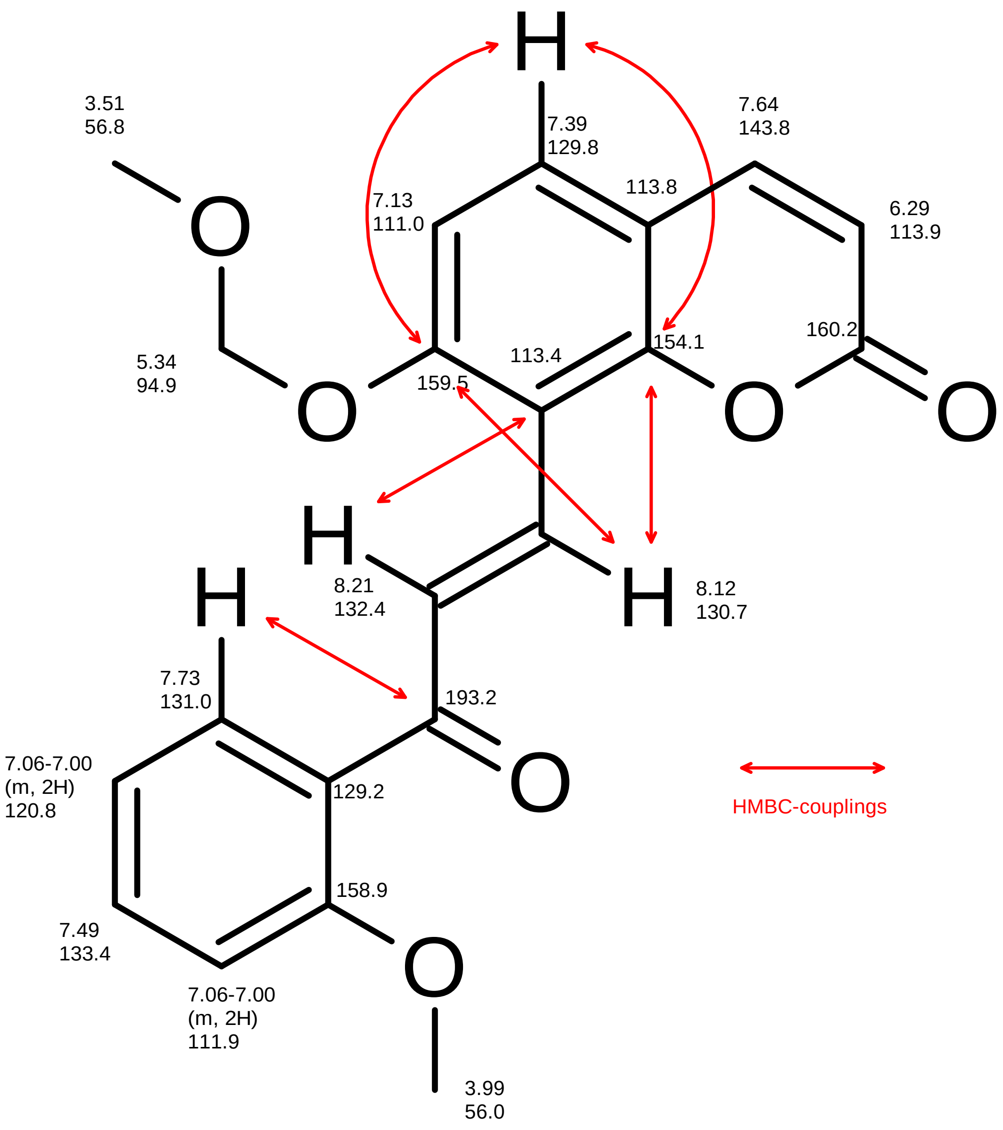

Figure S146: ^{1}H NMR (400 MHz, $CDCl_3$) of **13at**

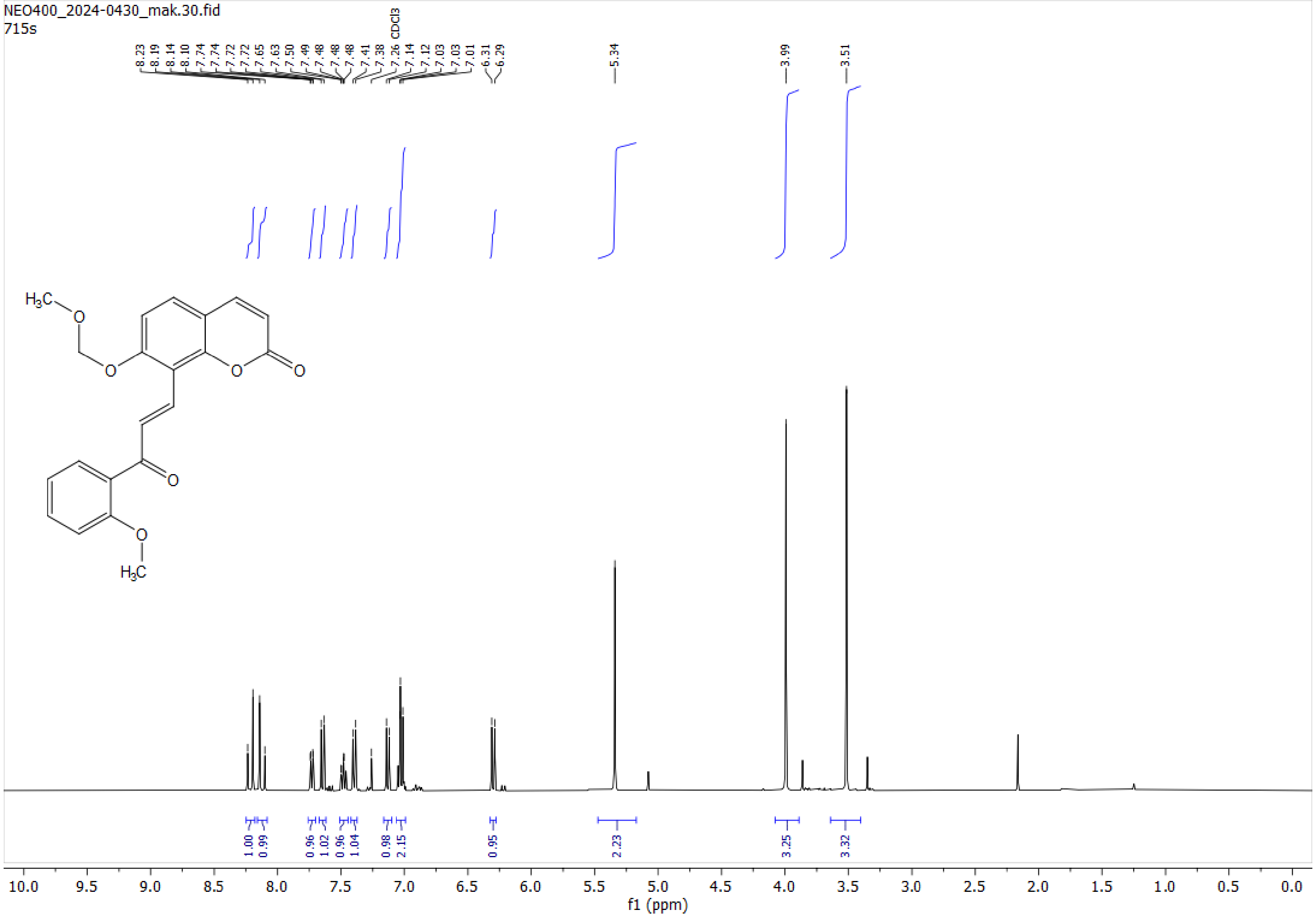

Figure S147: $^{13}C\{^{1}H\}$ NMR (100 MHz, $CDCl_3$) of **13at**

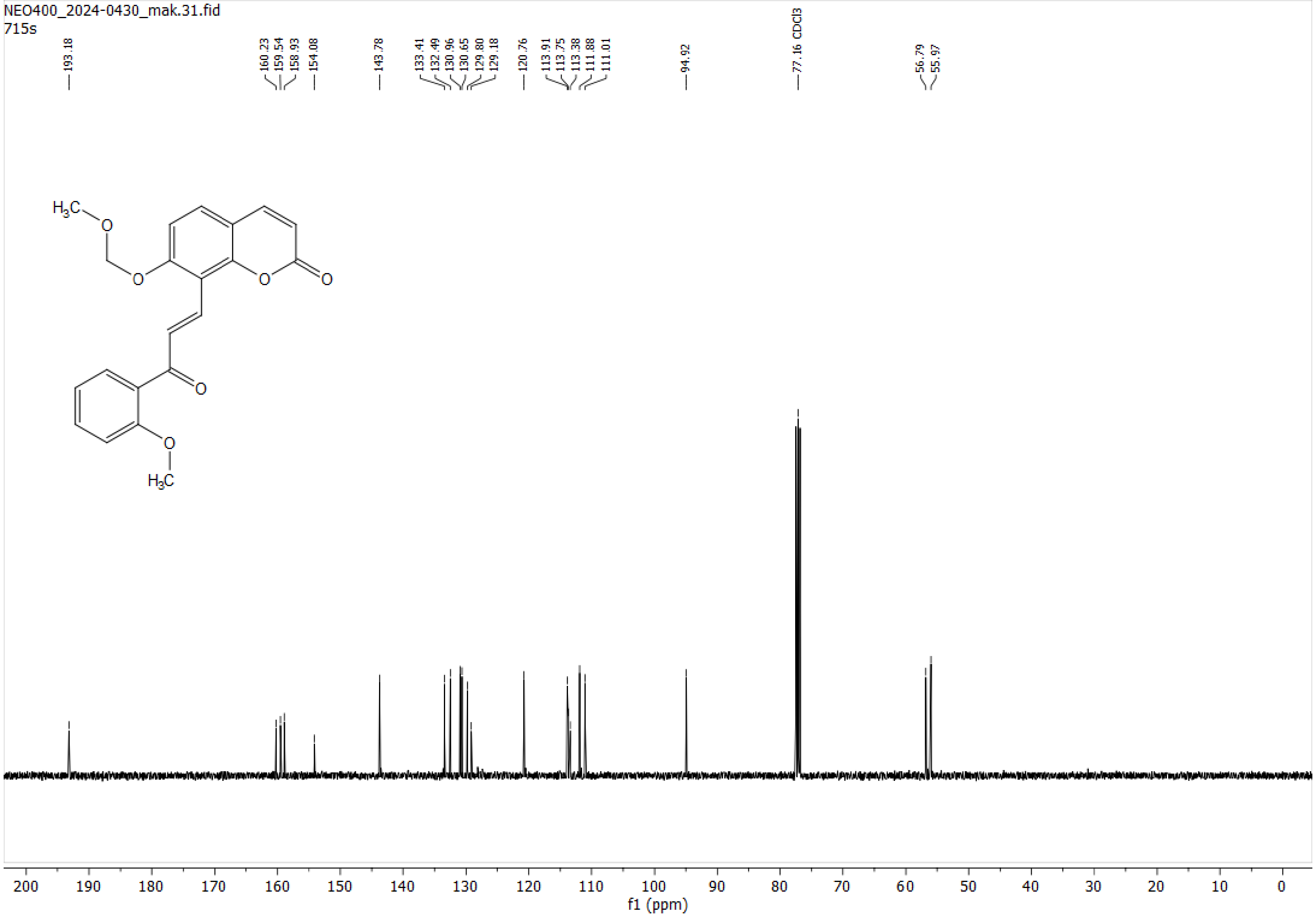

Figure S148: COSY (400 MHz, $CDCl_3$) of **13at**

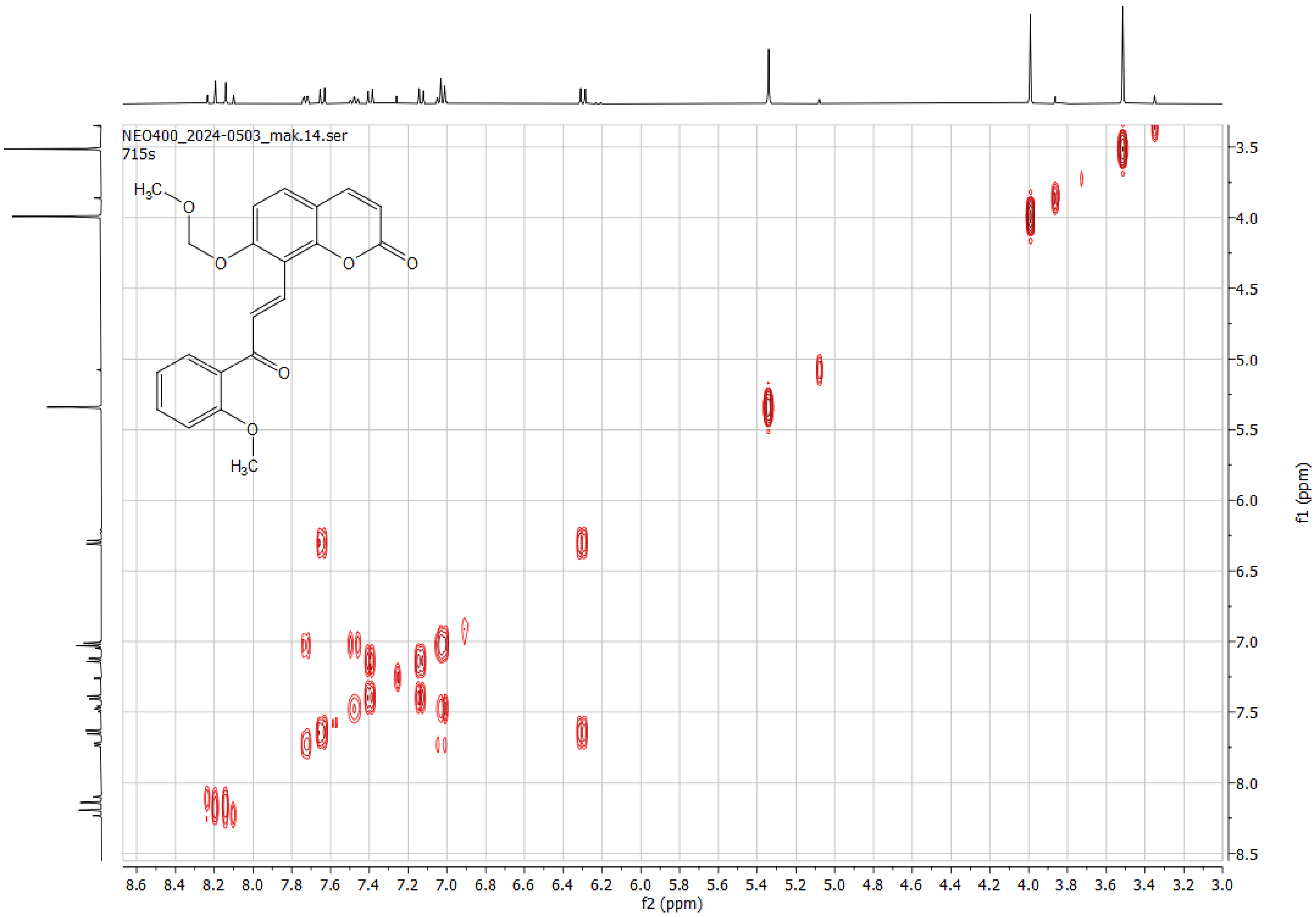

Figure S149: HSQC (400/100 MHz, $CDCl_3$) of **13at**

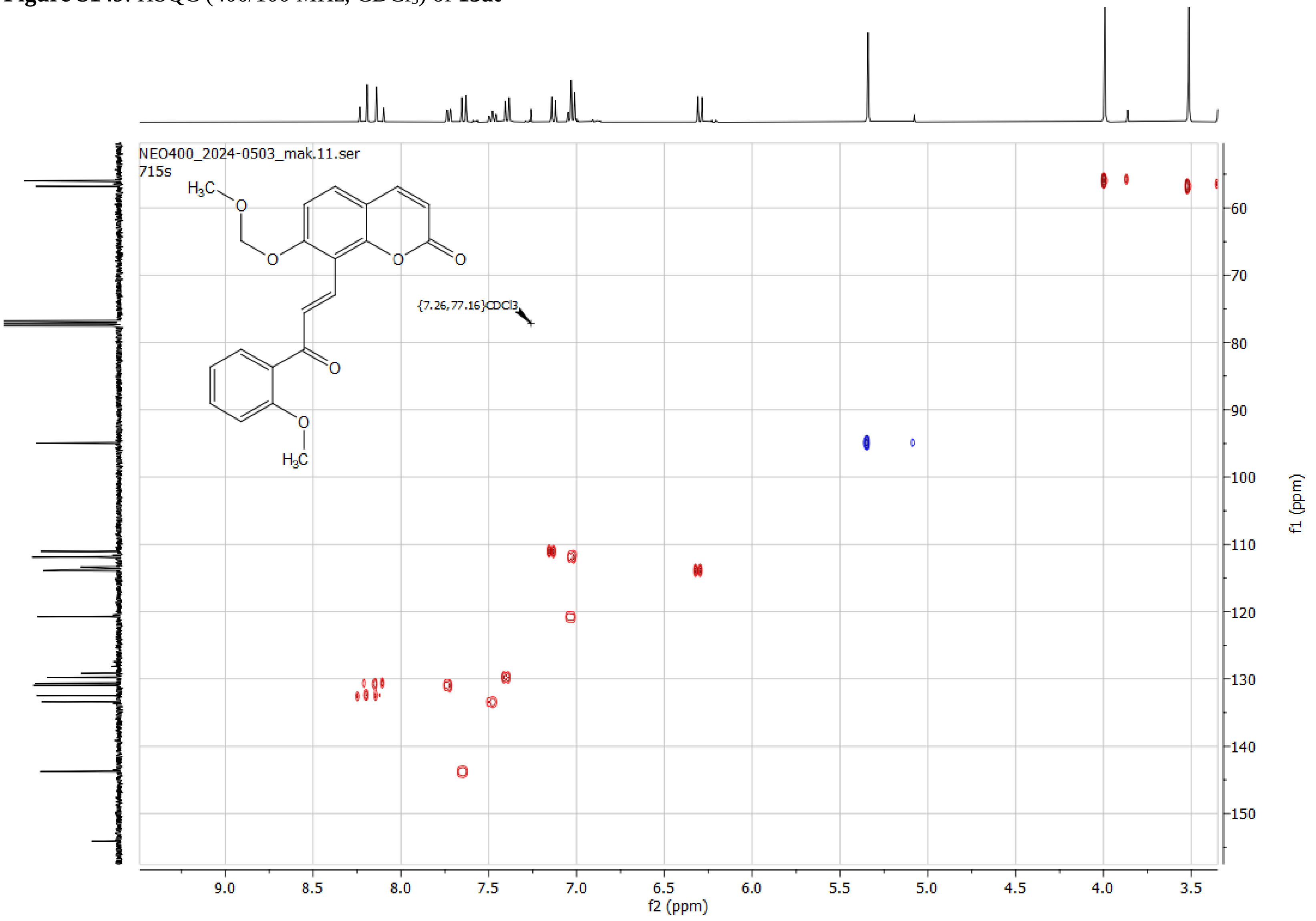

Figure S150: HMBC (400/100 MHz, $CDCl_3$) of **13at**

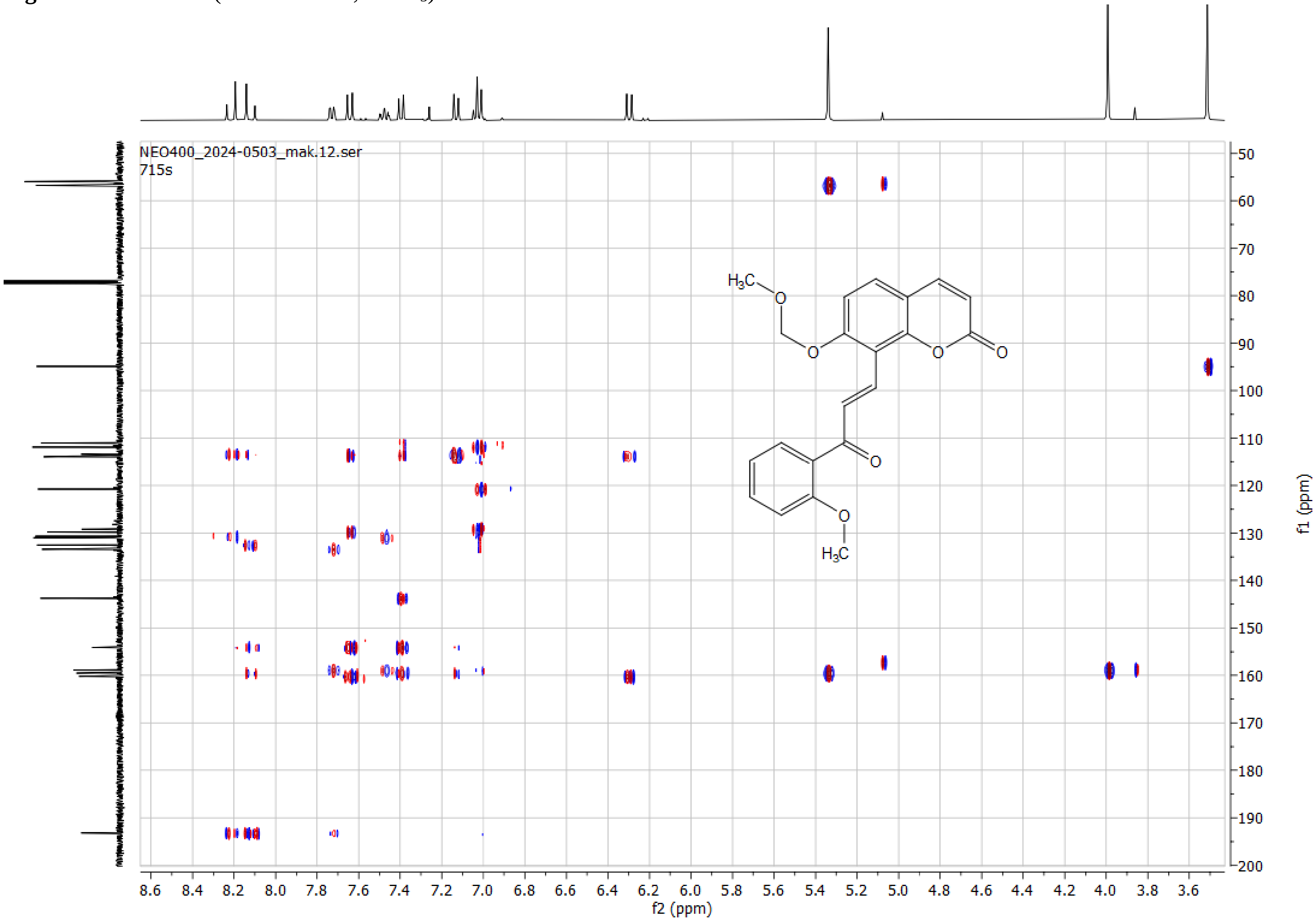

Figure S151: NOESY (400 MHz, $CDCl_3$) of **13at**

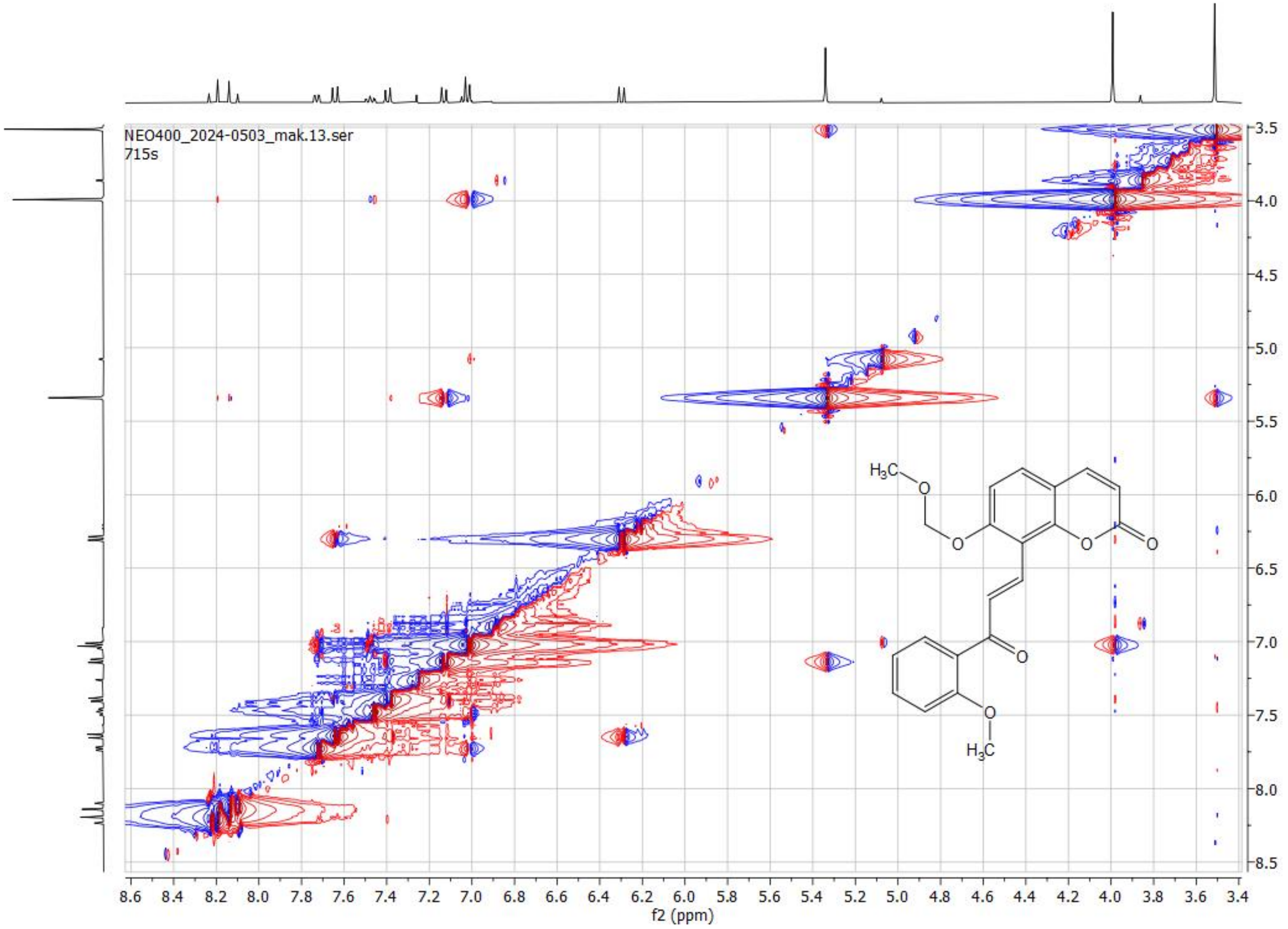

Figure S152: NMR-Signal assignment and selected HMBC-couplings and NOE-interaction for compound **14au**

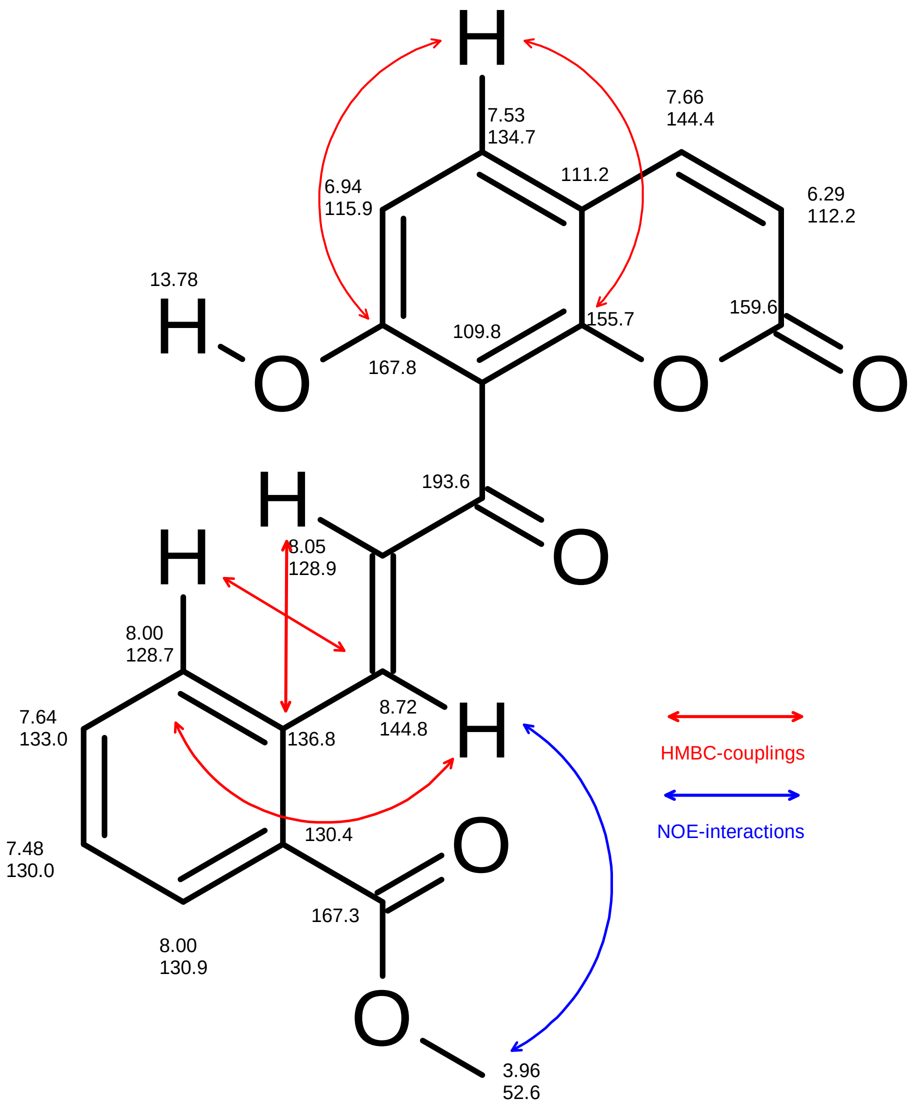

Figure S153: ^{1}H NMR (400 MHz, $CDCl_3$) of **14au**

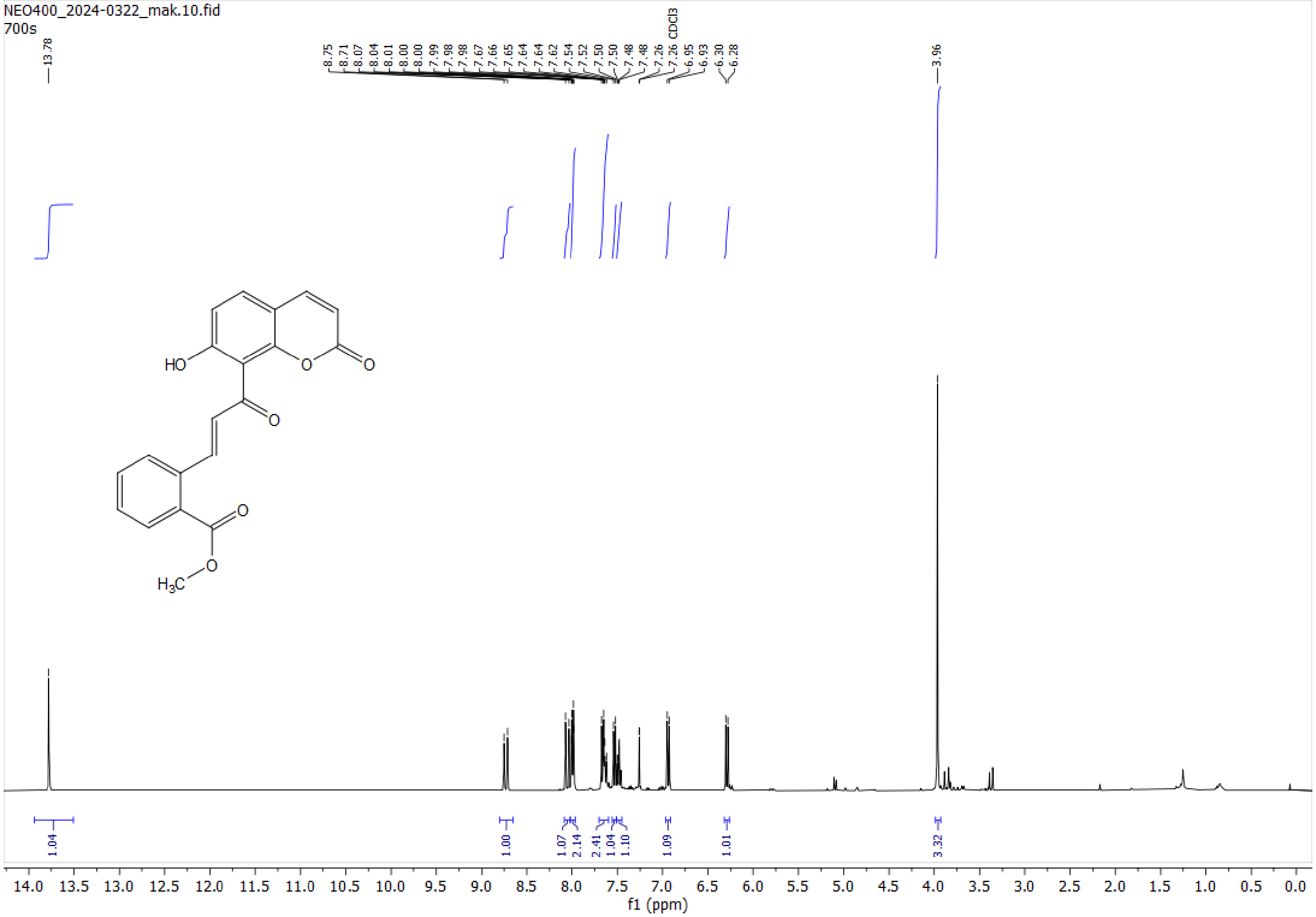

Figure S154: $^{13}C\{^{1}H\}$ NMR (100 MHz, $CDCl_3$) of **14au**

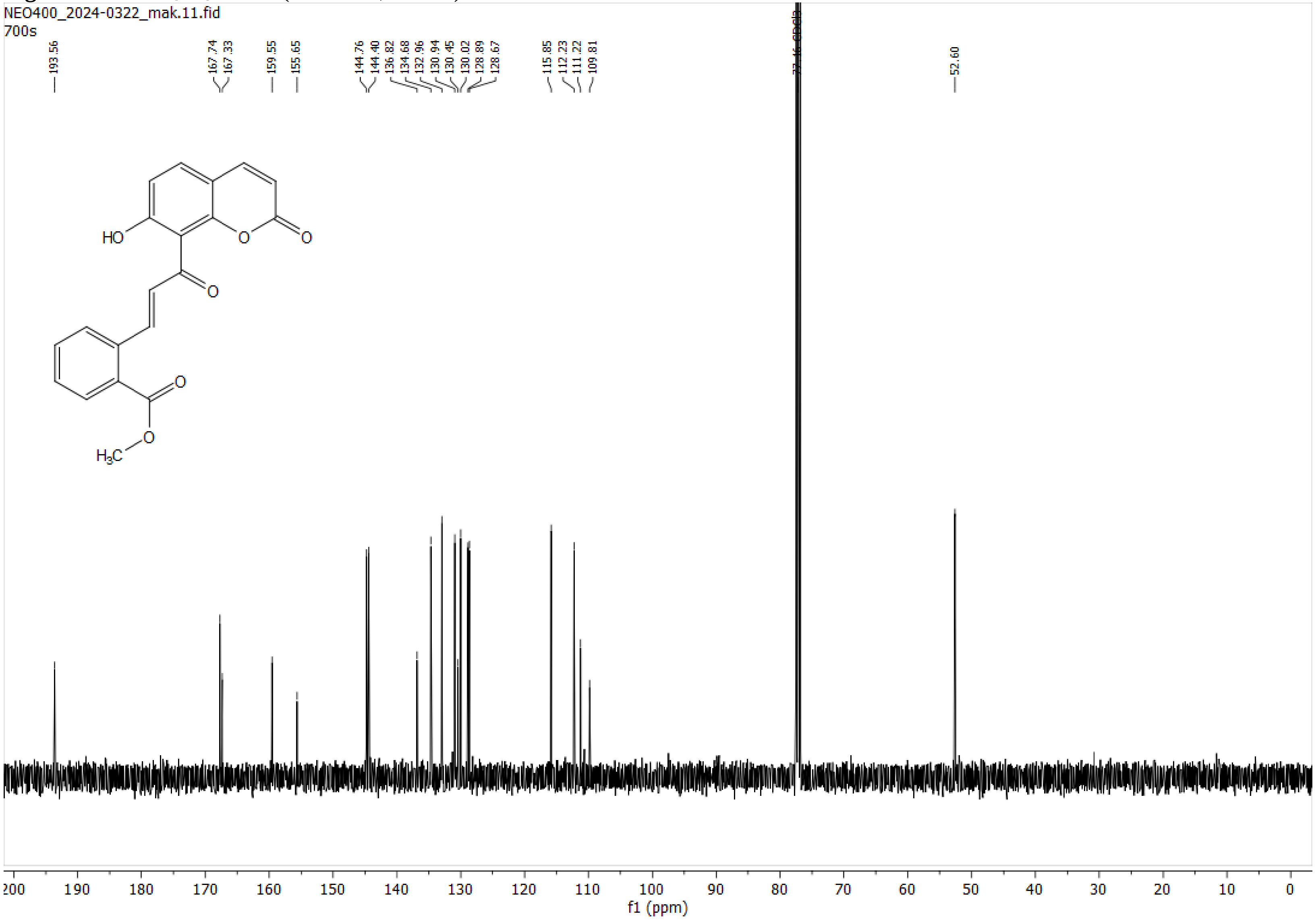

Figure S155: COSY (400 MHz, $CDCl_3$) of **14au**

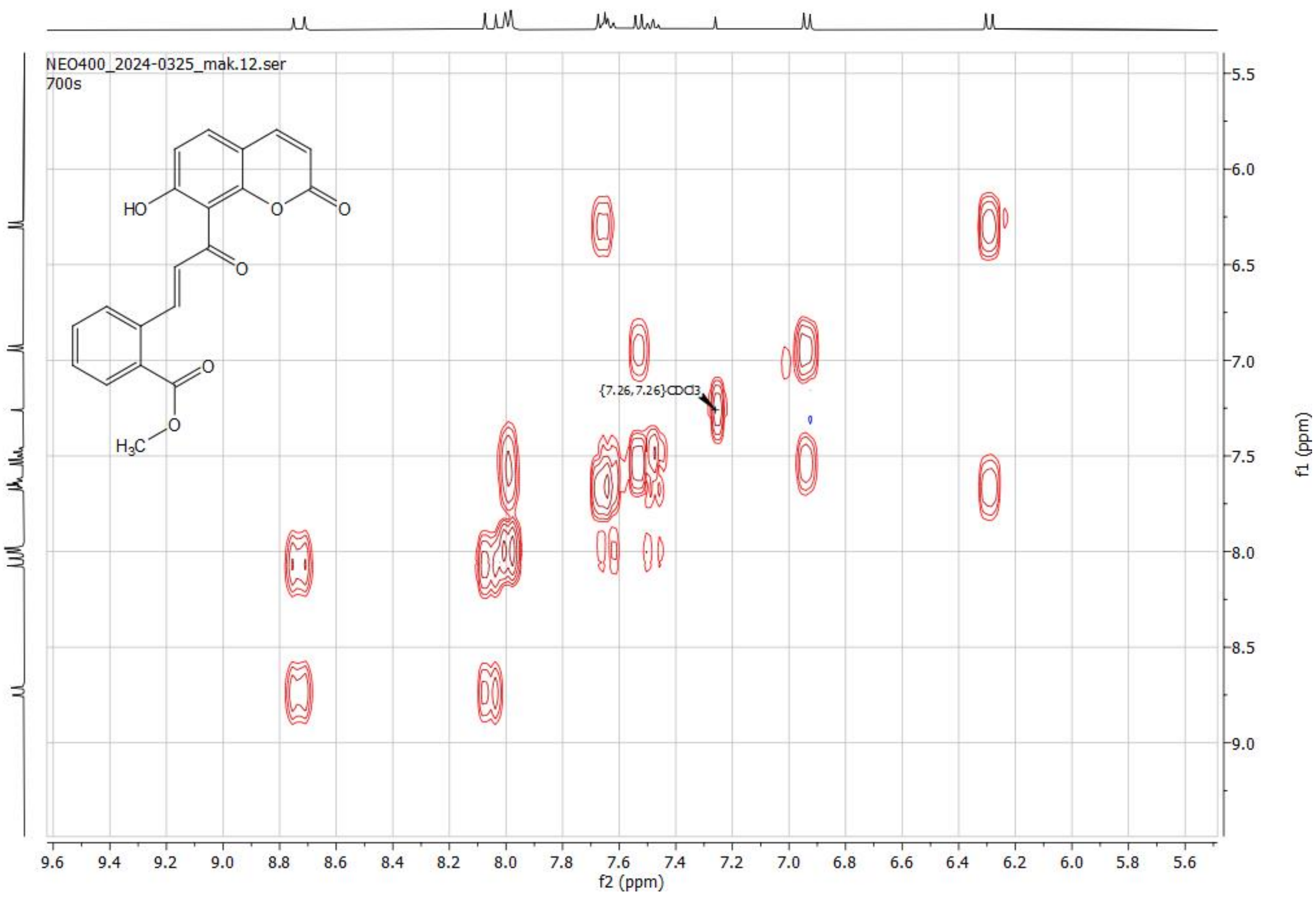

Figure S156: HSQC (400/100 MHz, $CDCl_3$) of **14au**

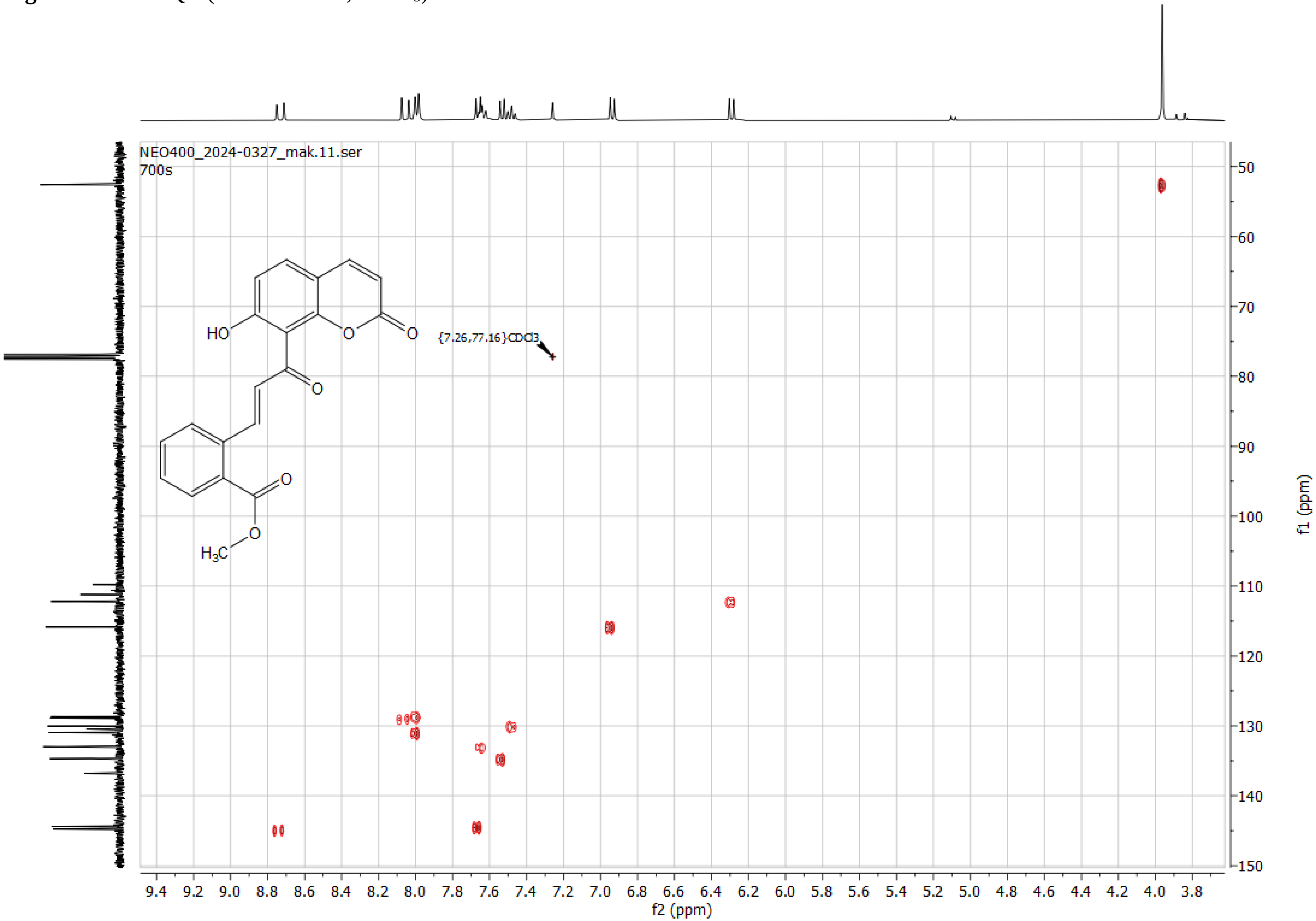

Figure S157: HMBC (400/100 MHz, $CDCl_3$) of **14au**

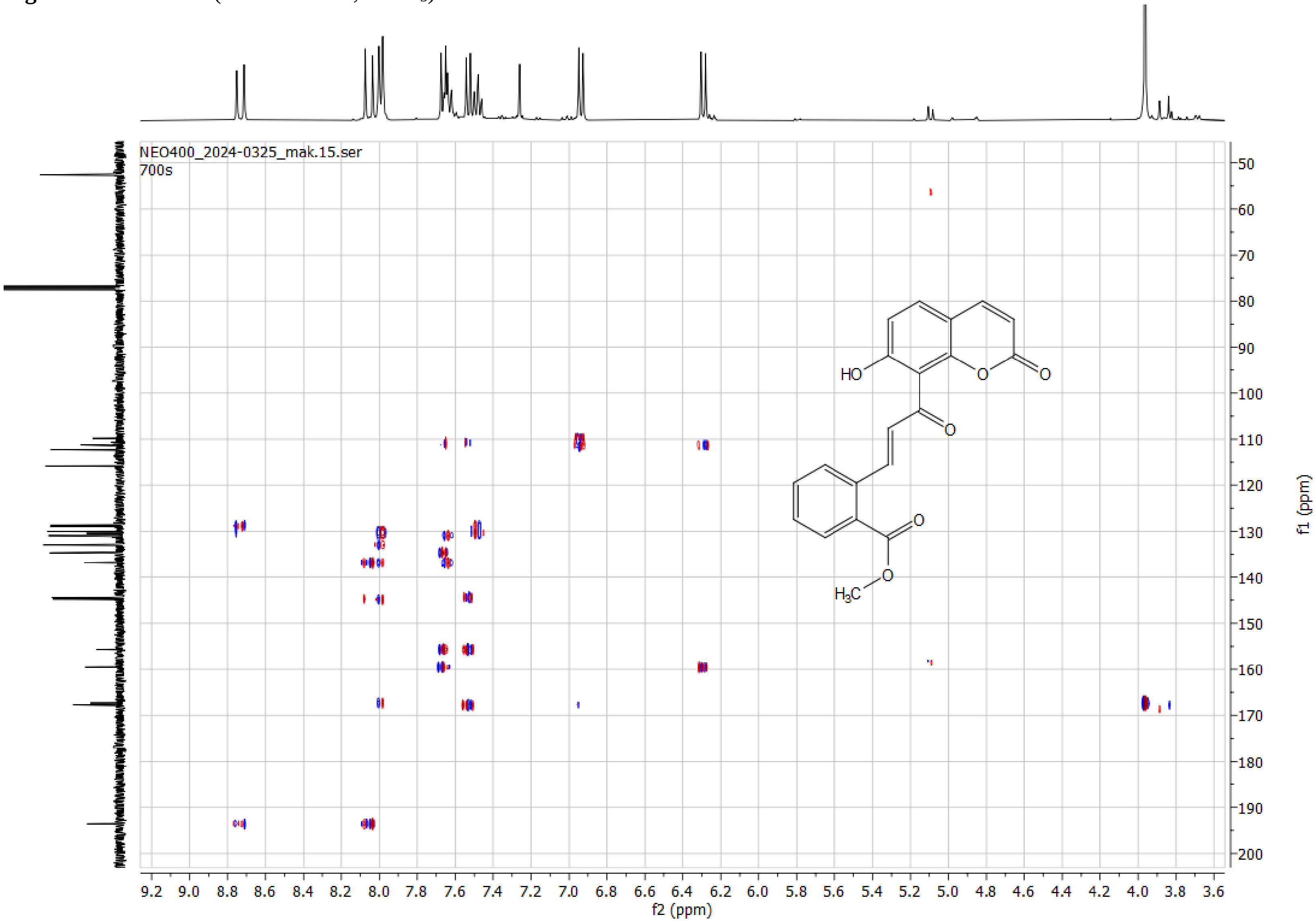

Figure S158: NOESY (400 MHz, $CDCl_3$) of **14au**

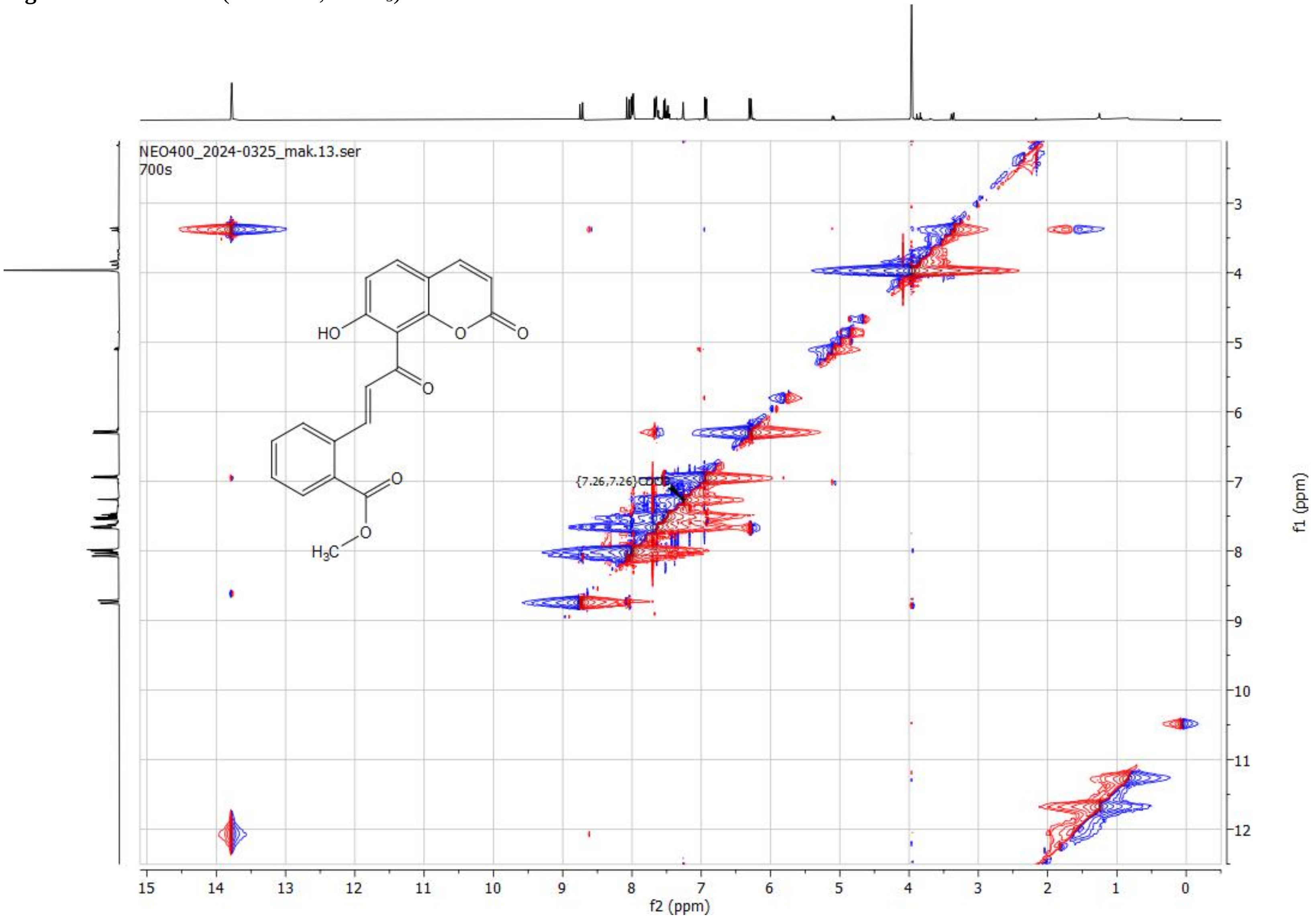

Figure S159: NMR-Signal assignment and selected HMBC-couplings for compound **16af**

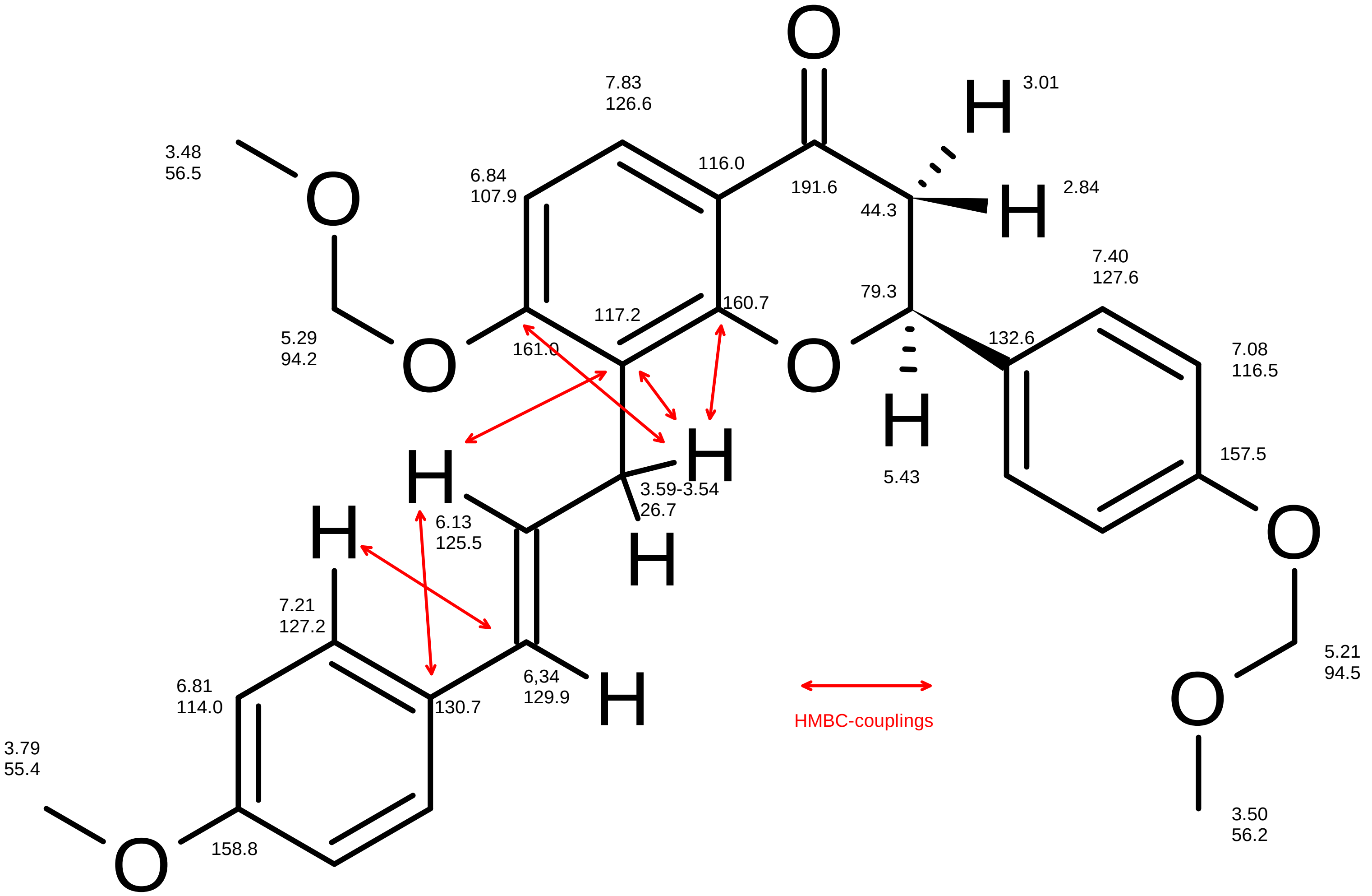

Figure S160: ^{1}H NMR (400 MHz, $CDCl_3$) of **16af**

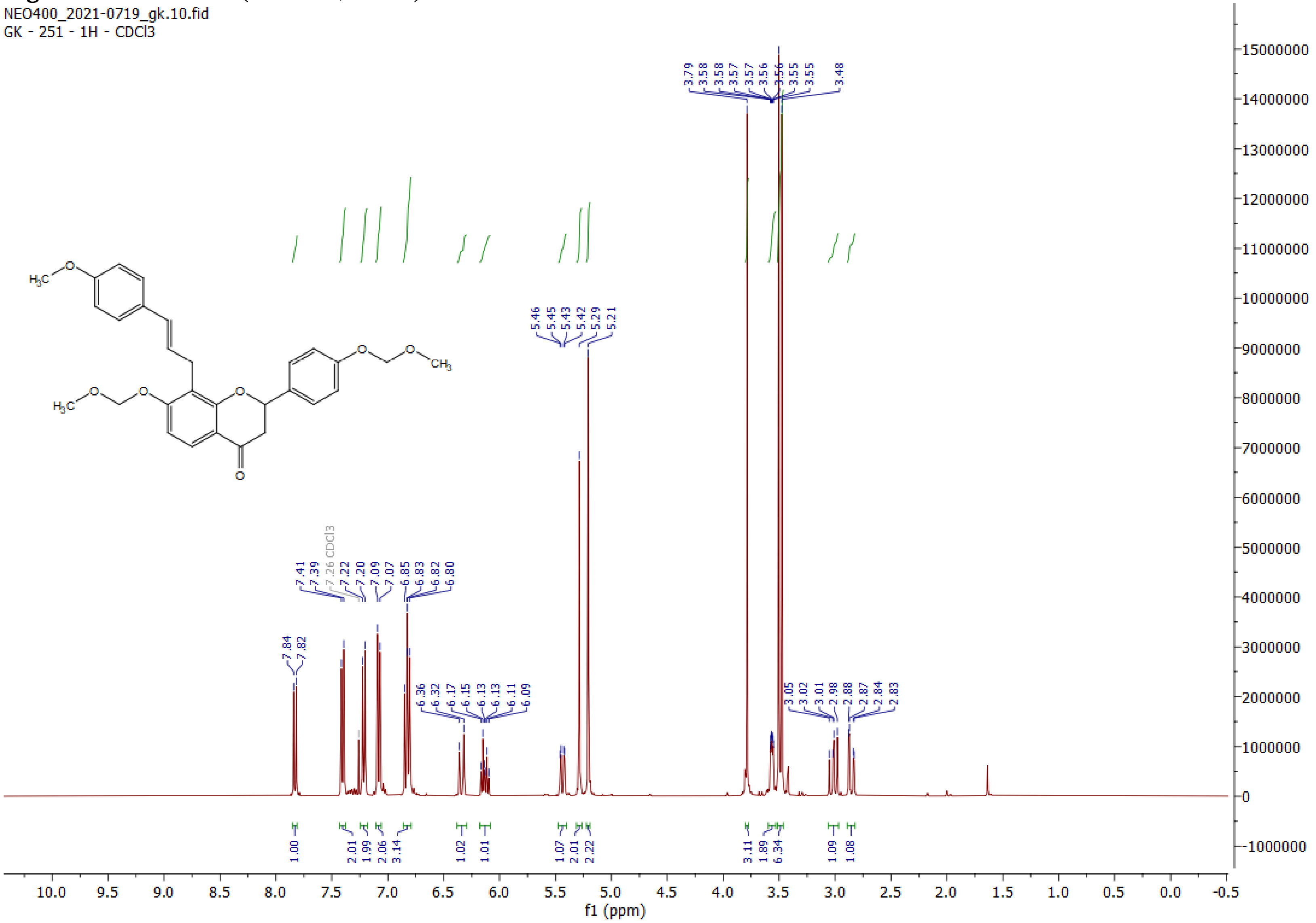

Figure S161: $^{13}C\{^{1}H\}$ NMR (100 MHz, $CDCl_3$) of **16af**

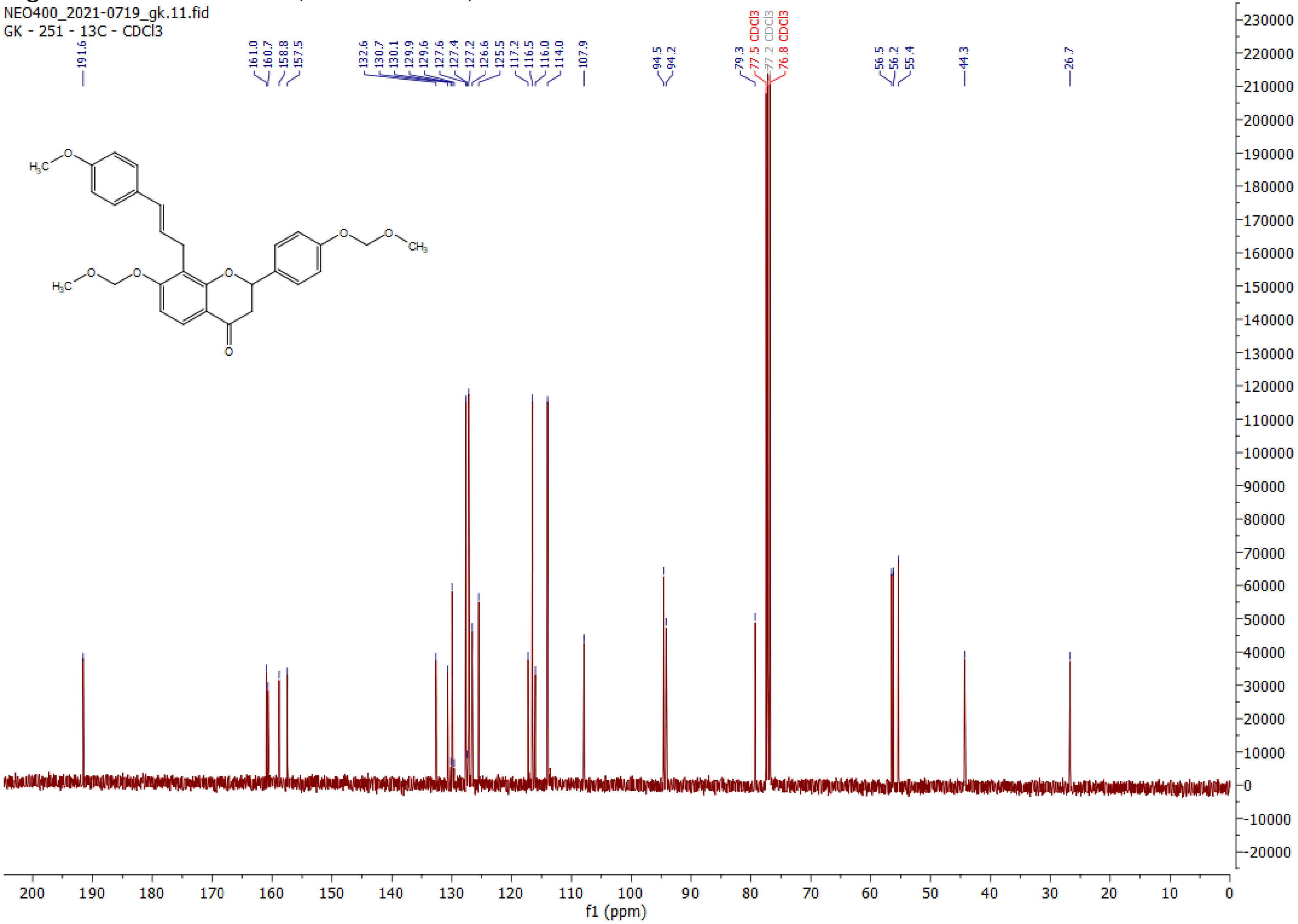

Figure S162: HSQC (400/100 MHz, $CDCl_3$) of **16af**

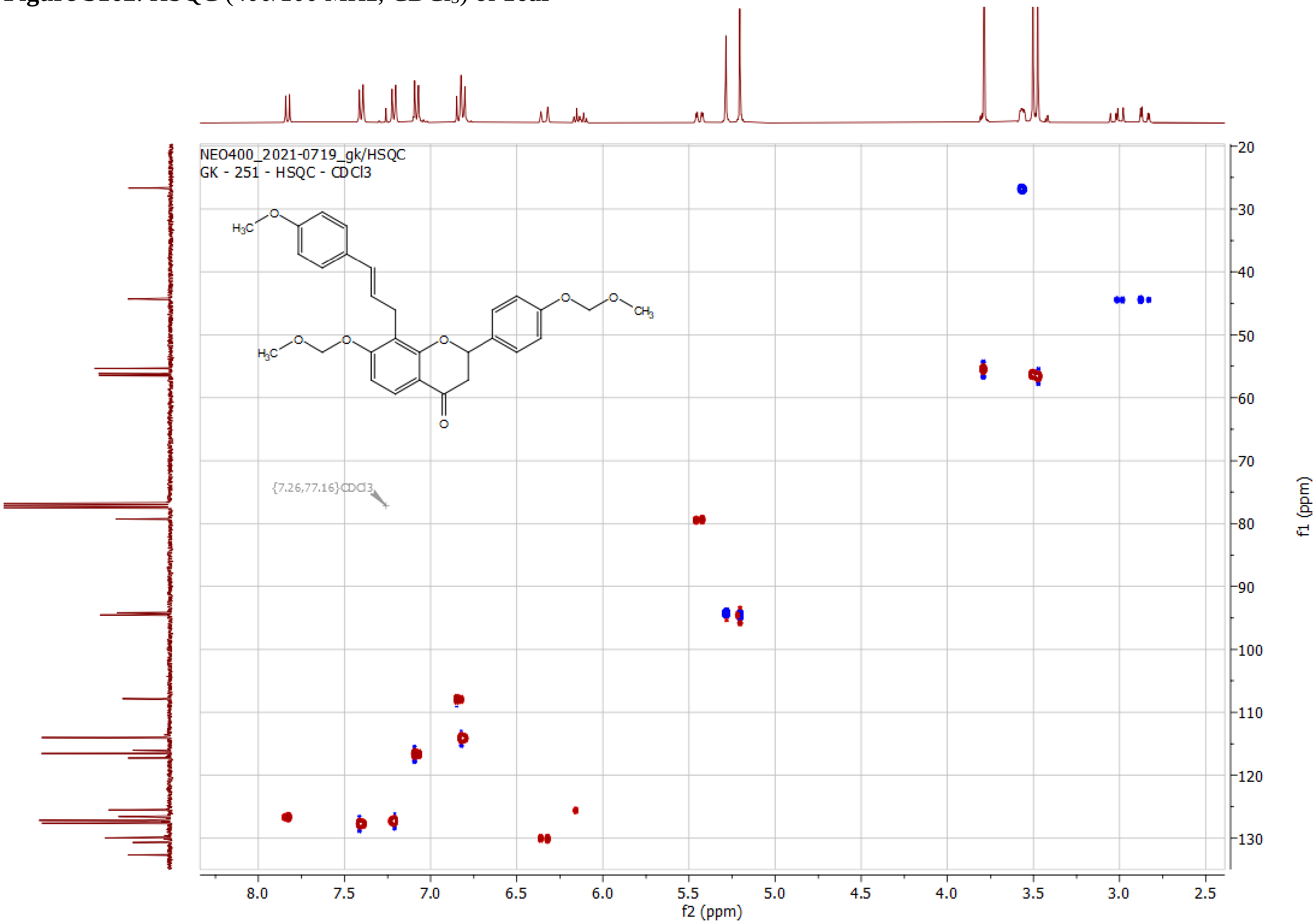

Figure S163: HMBC (400/100 MHz, $CDCl_3$) of **16af**

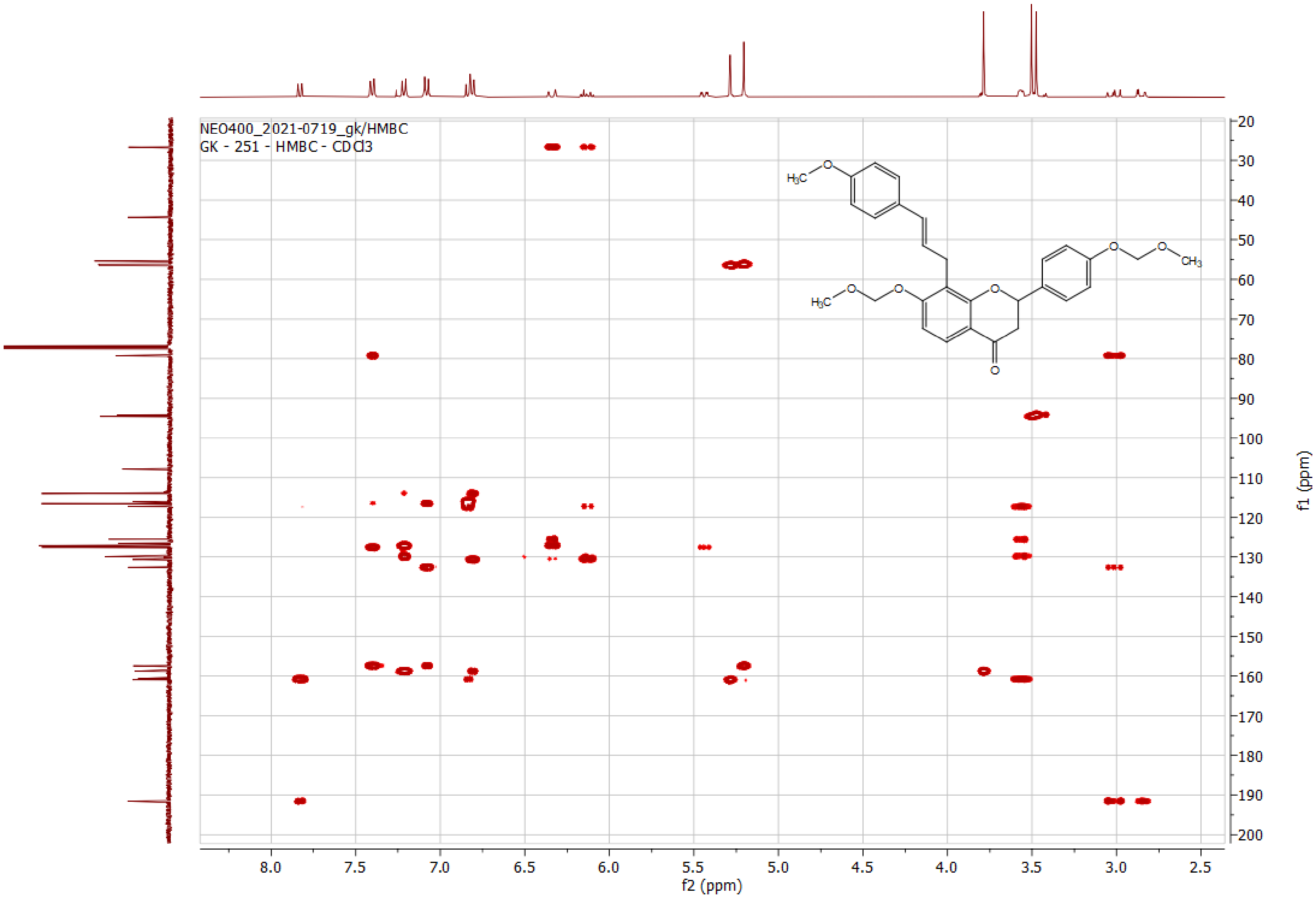

Figure S164: ^{1}H NMR (400 MHz, $CDCl_3$) of **16bf**

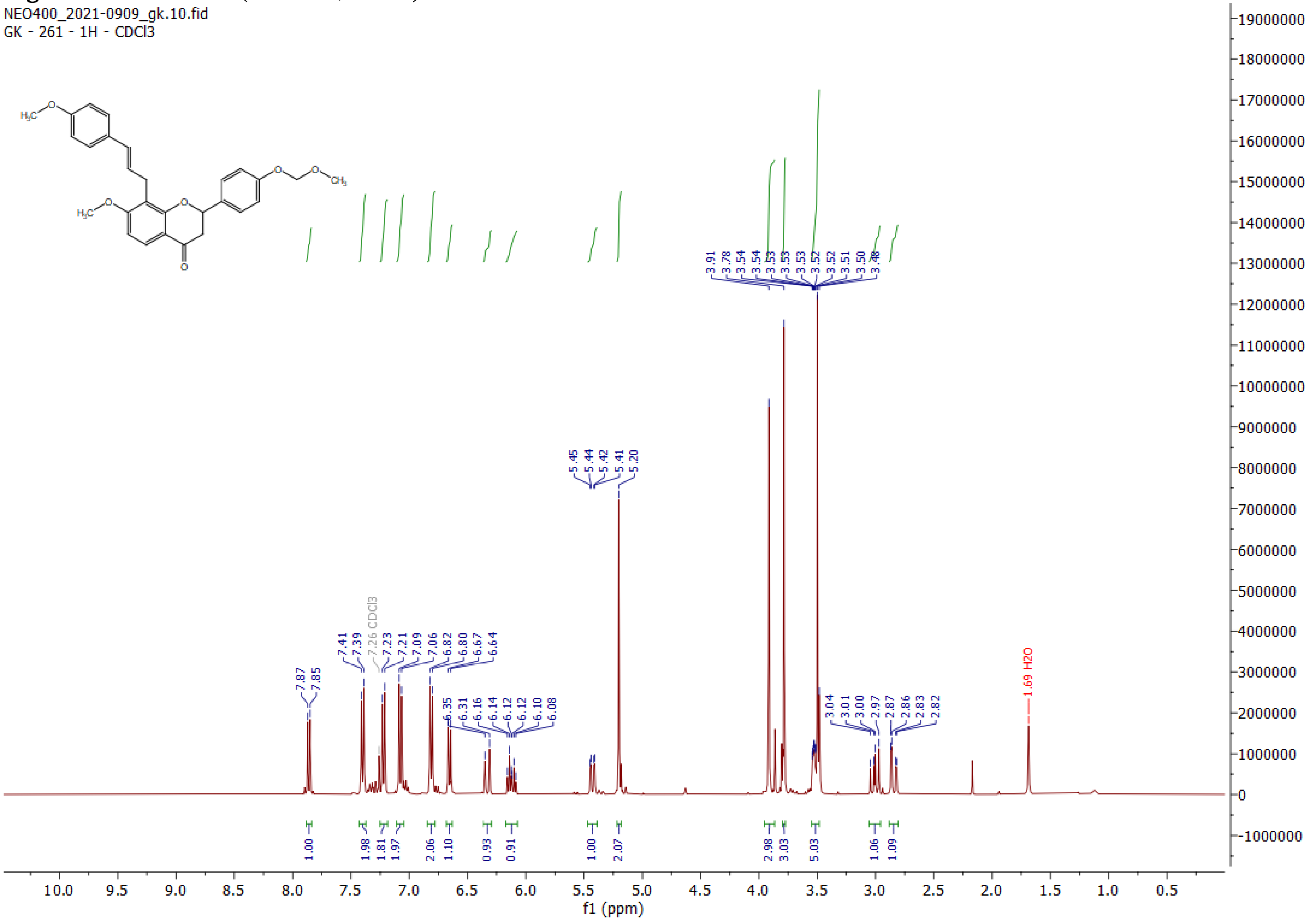

Figure S165: $^{13}C\{^{1}H\}$ NMR (100 MHz, $CDCl_3$) of **16bf**

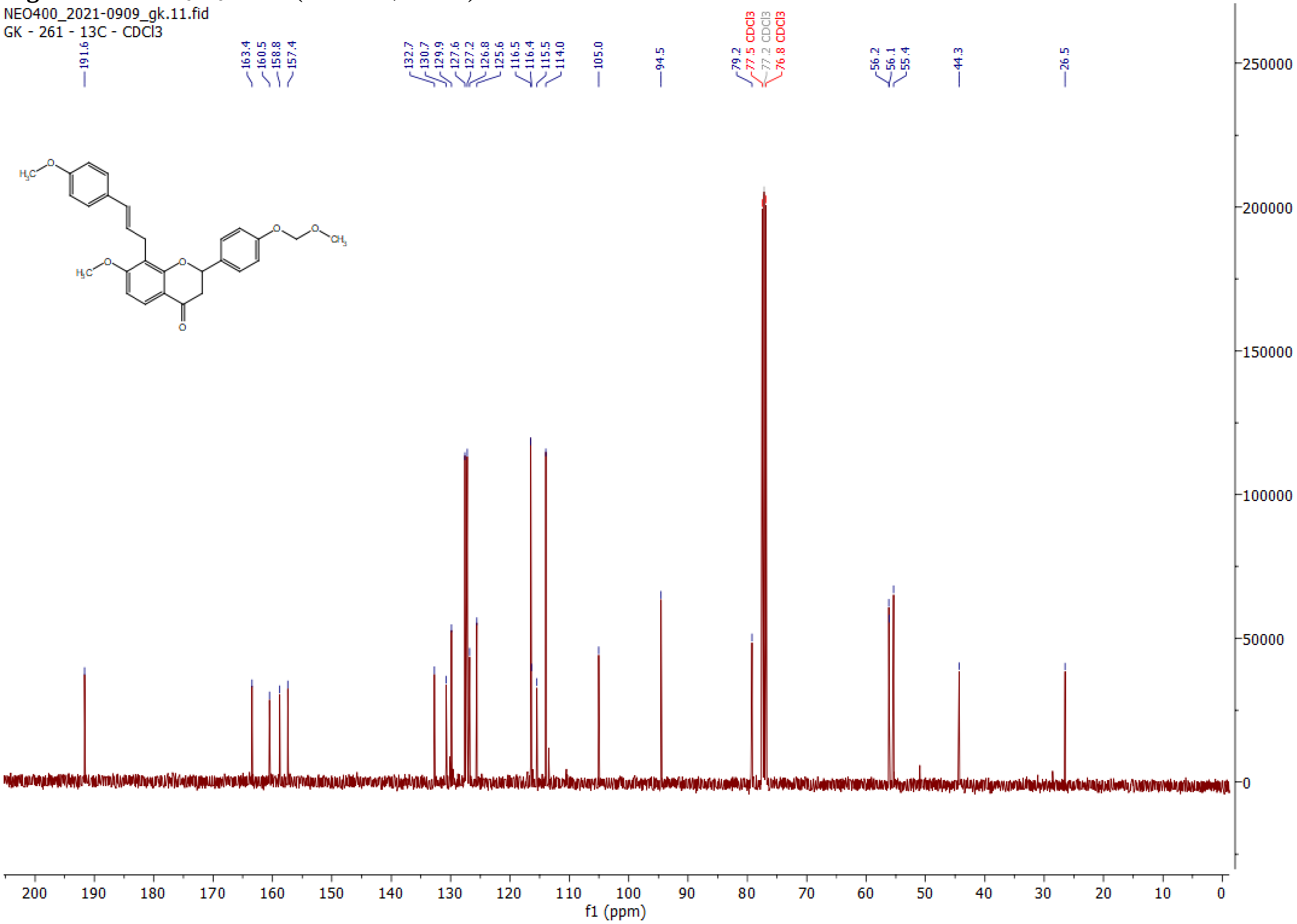

Figure S166: HSQC (400/100 MHz, $CDCl_3$) of **16bf**

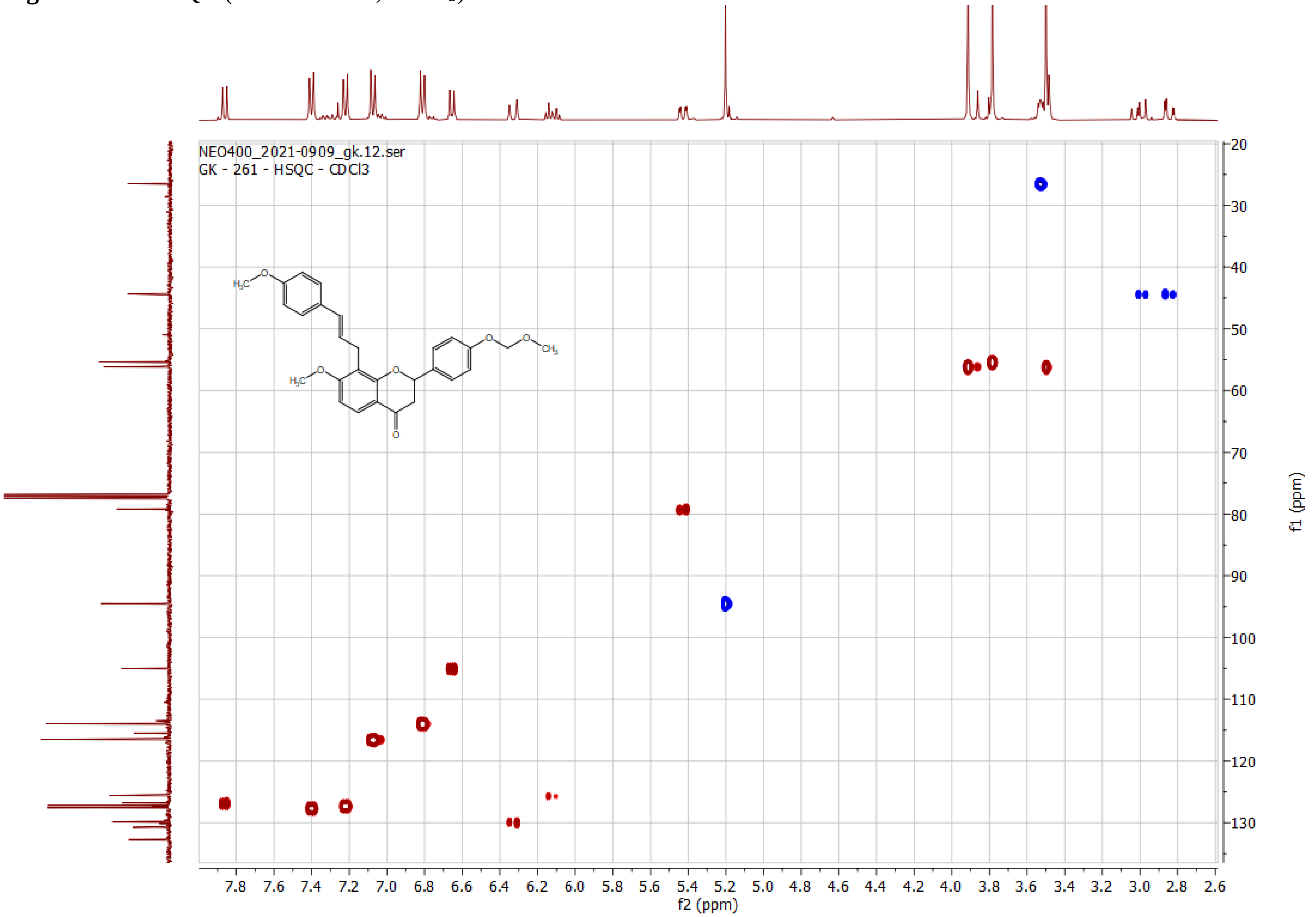

Figure S167: HMBC (400/100 MHz, $CDCl_3$) of **16bf**

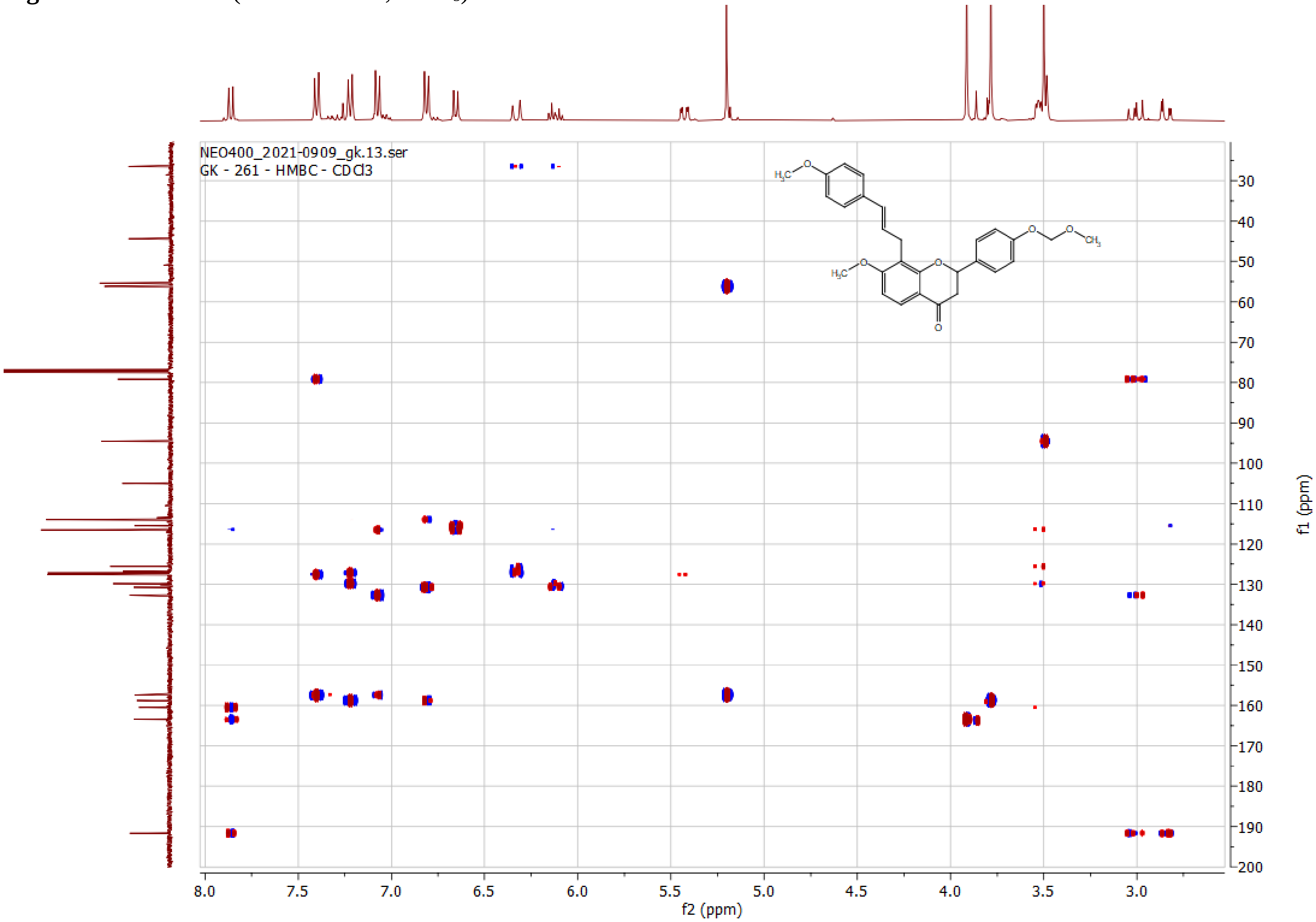

Figure S168: ^{1}H NMR (400 MHz, $CDCl_3$) of **16cf**

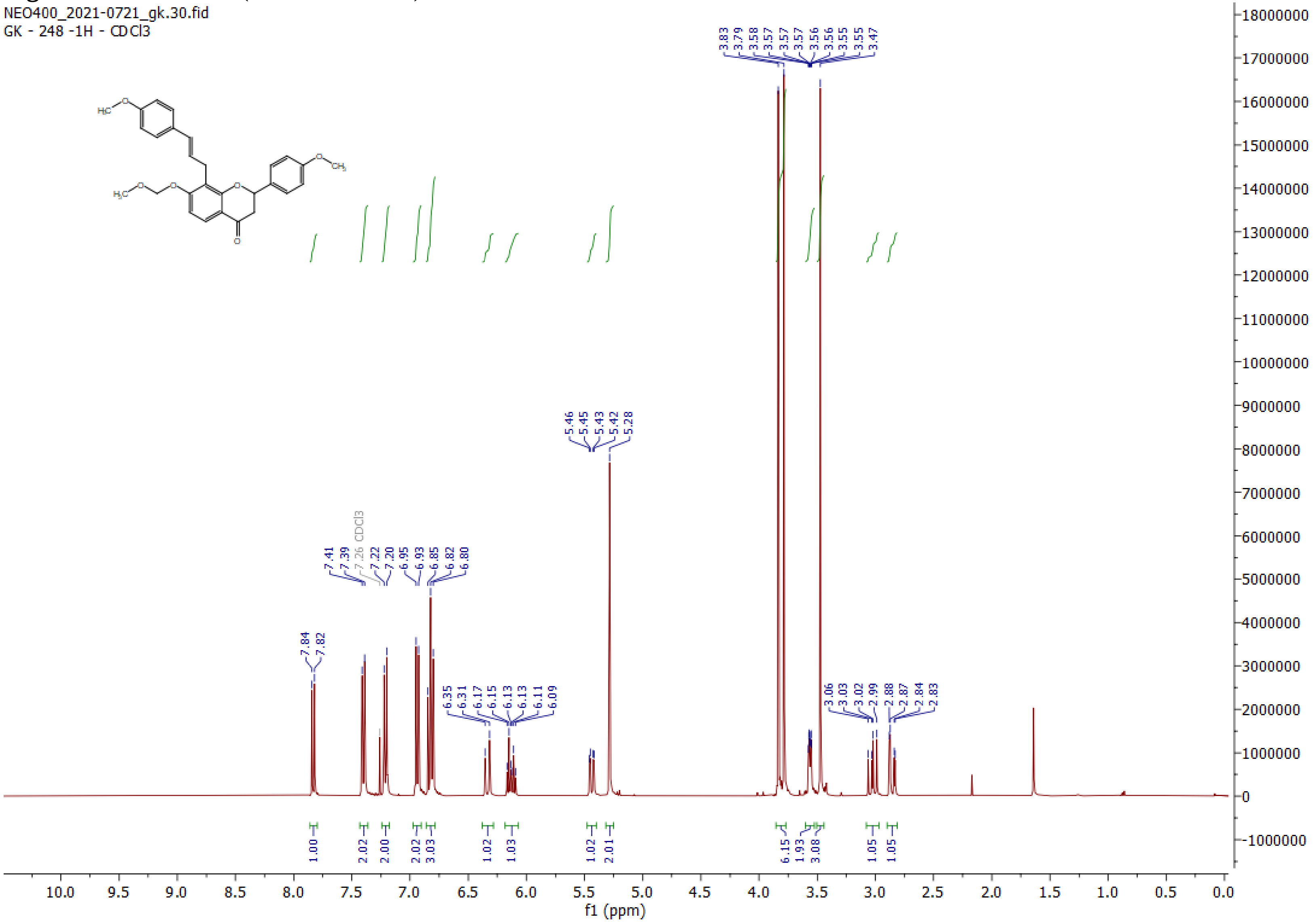

Figure S169: $^{13}C\{^{1}H\}$ NMR (100 MHz, $CDCl_3$) of **16cf**

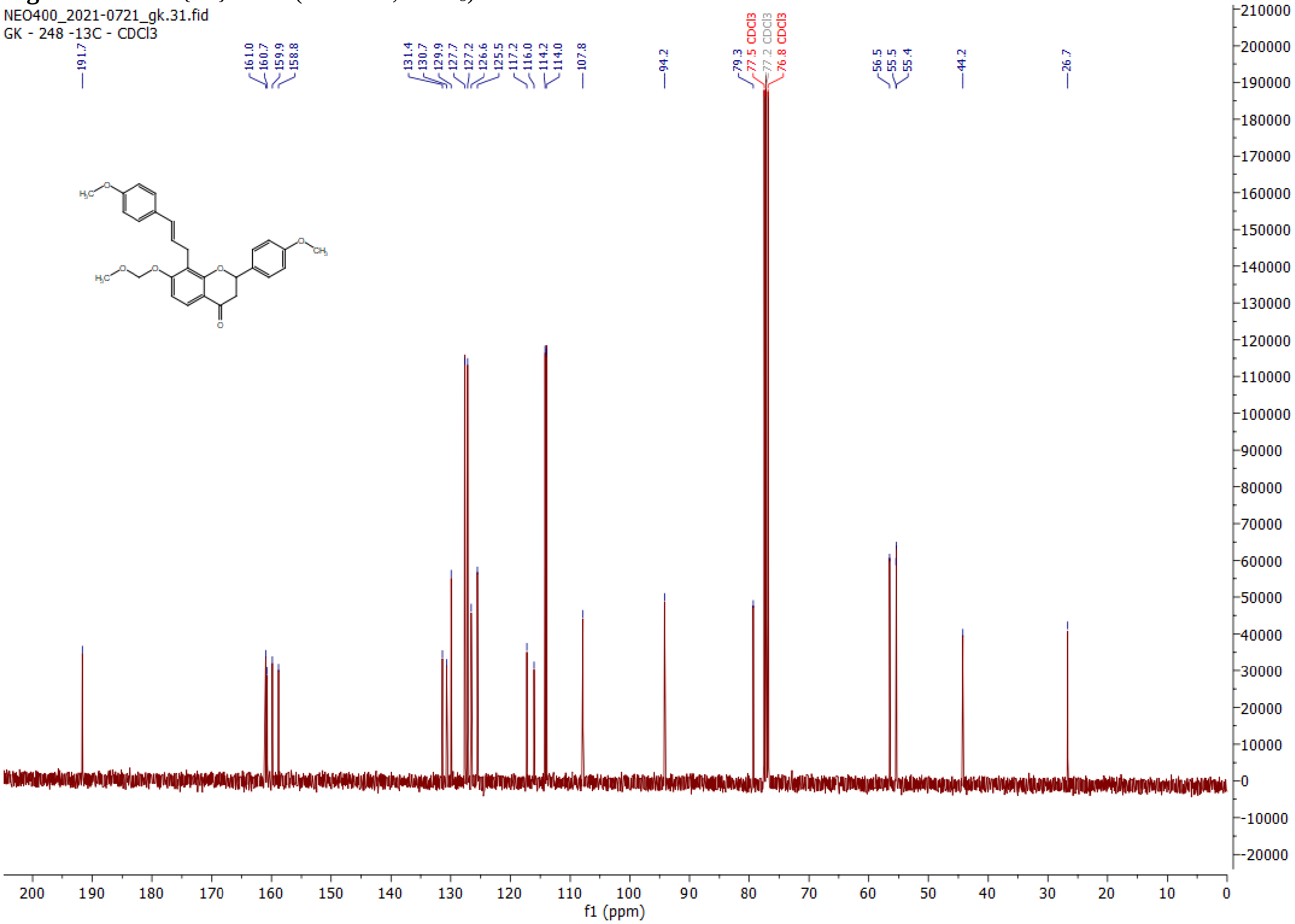

Figure S170: HSQC (400/100 MHz, $CDCl_3$) of **16cf**

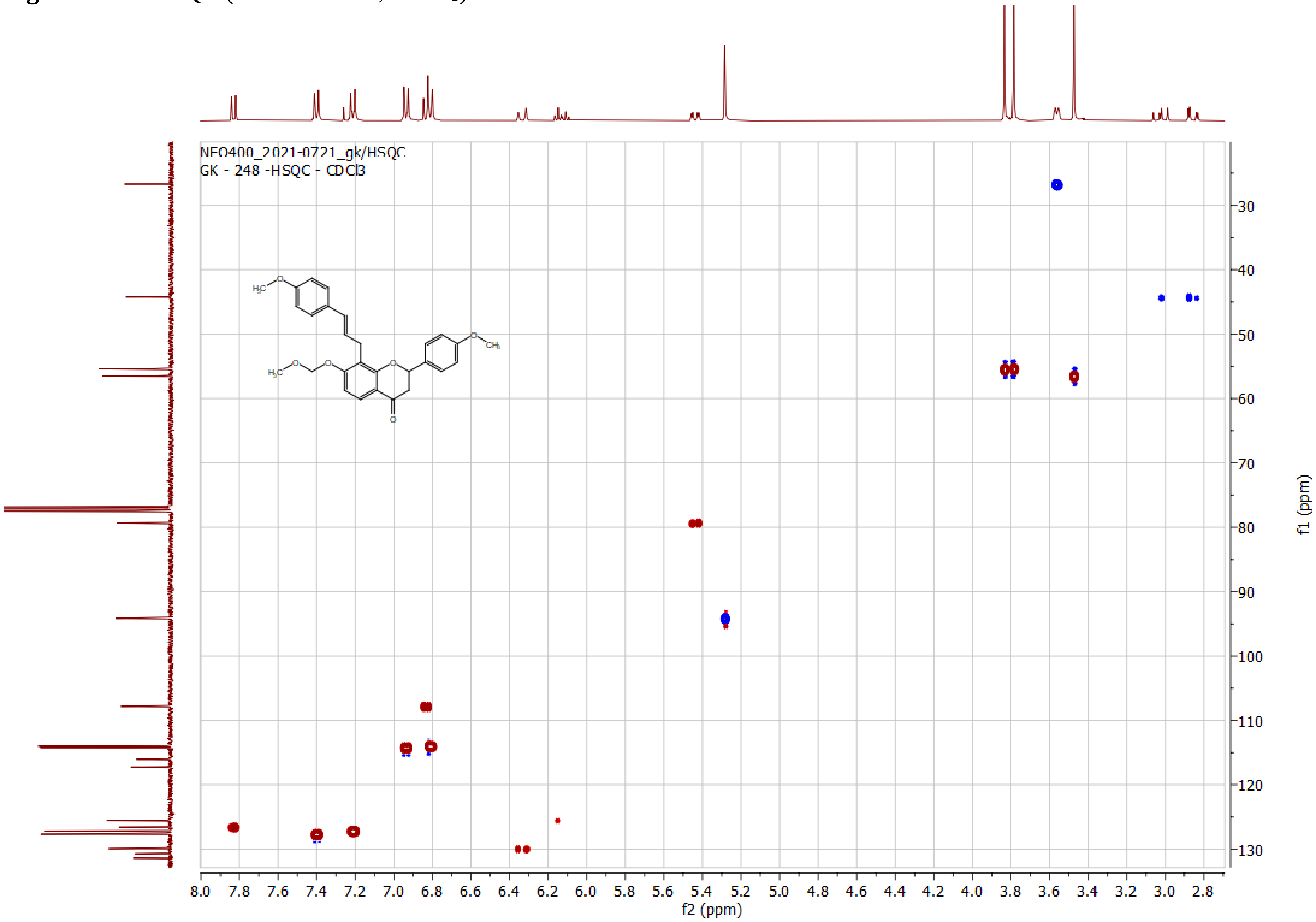

Figure S171: HMBC (400/100 MHz, $CDCl_3$) of **16cf**

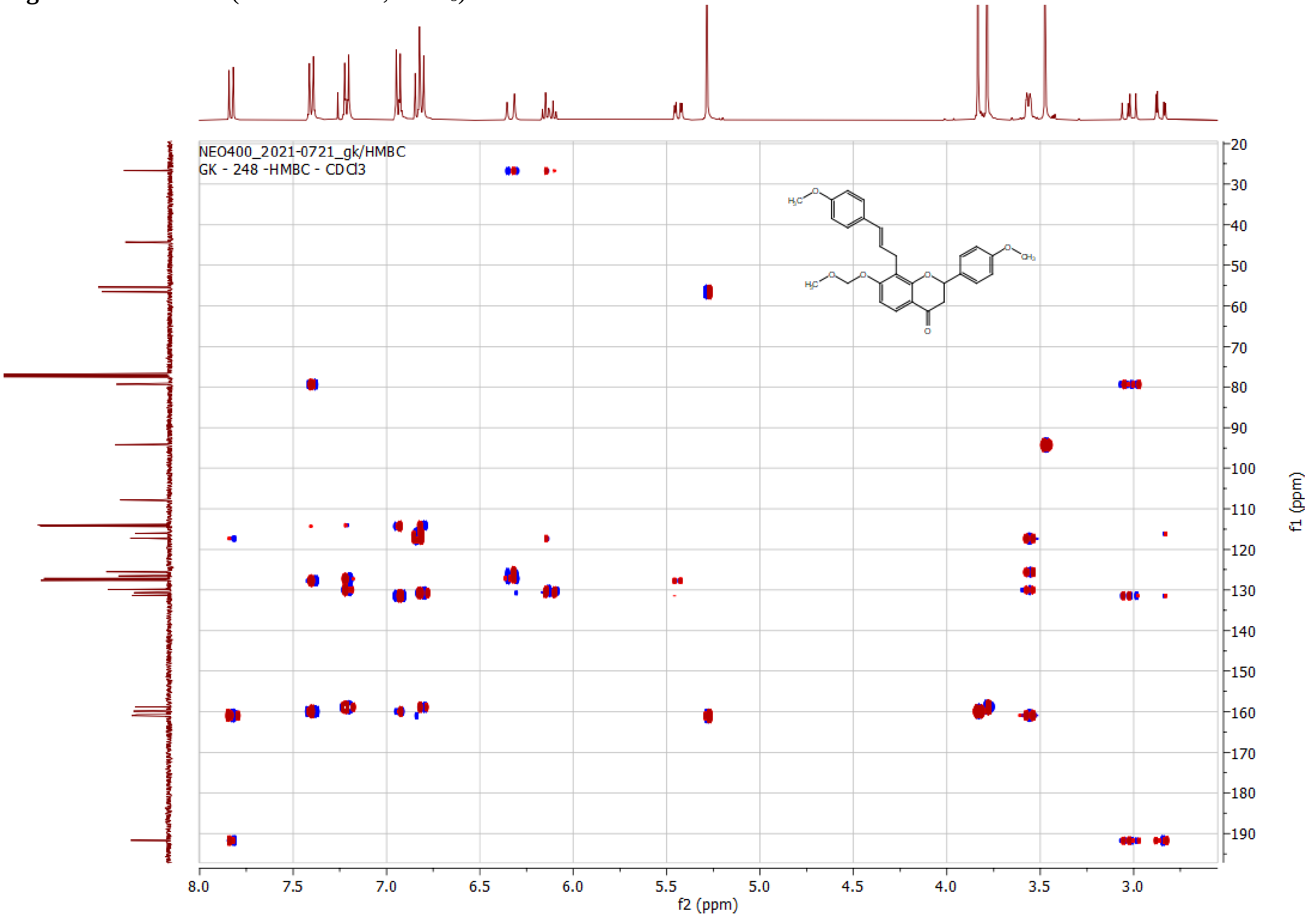

Figure S172: NMR-Signal assignment and selected HMBC-couplings for compound **17af**

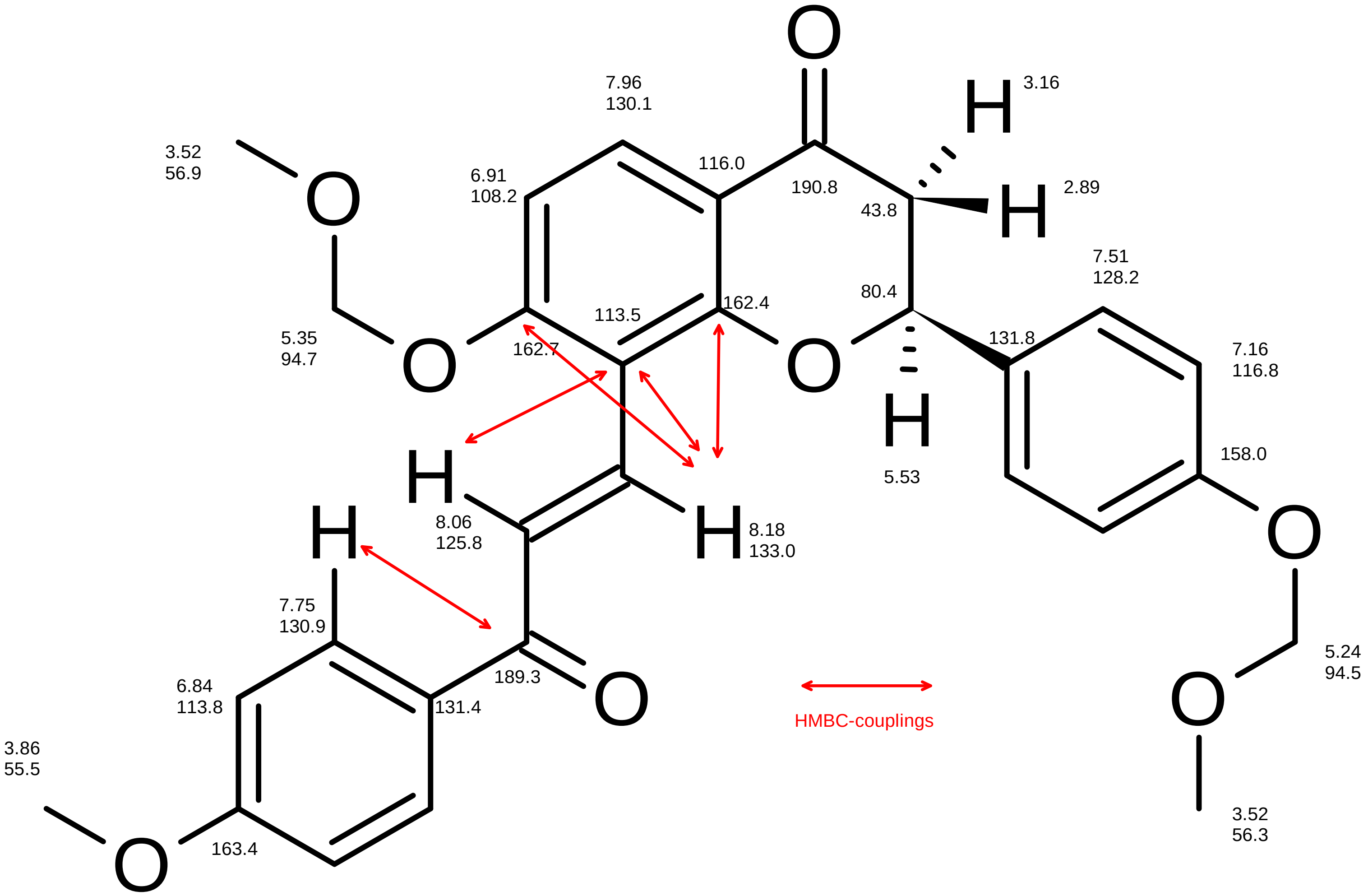

Figure S173: ^{1}H NMR (400 MHz, $CDCl_3$) of **17af**

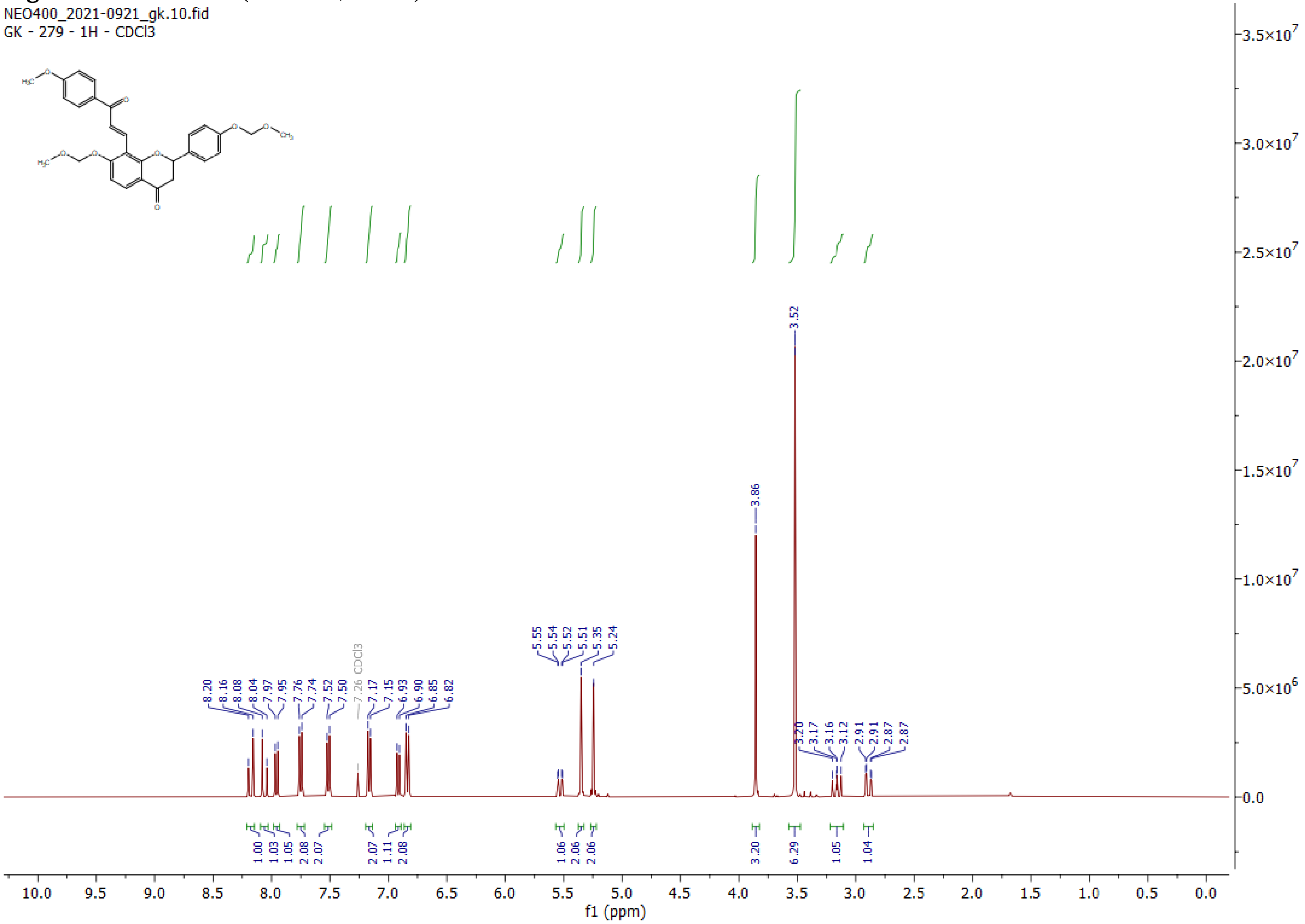

Figure S174: $^{13}C\{^{1}H\}$ NMR (100 MHz, $CDCl_3$) of **17af**

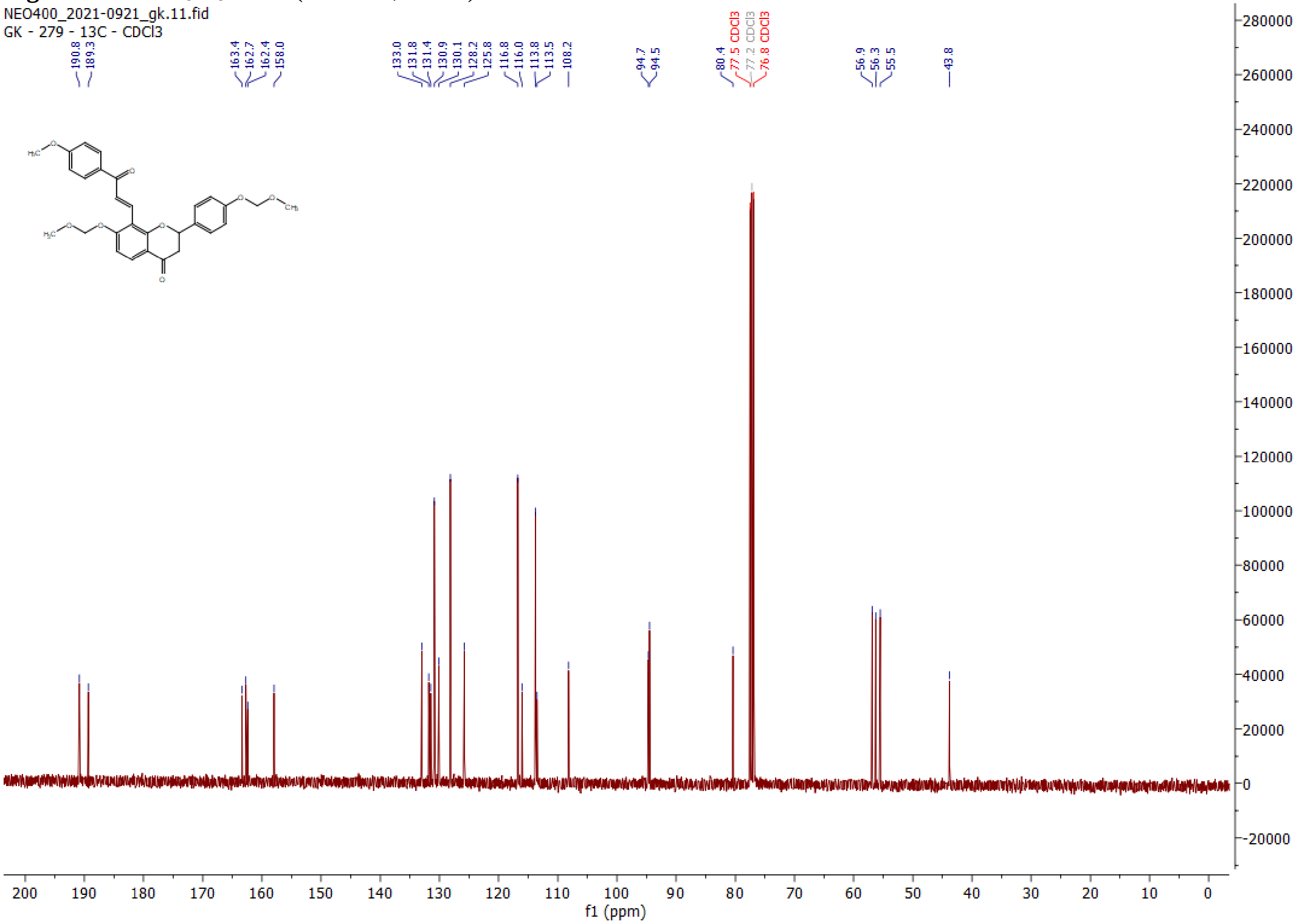

Figure S175: HSQC (400/100 MHz, $CDCl_3$) of **17af**

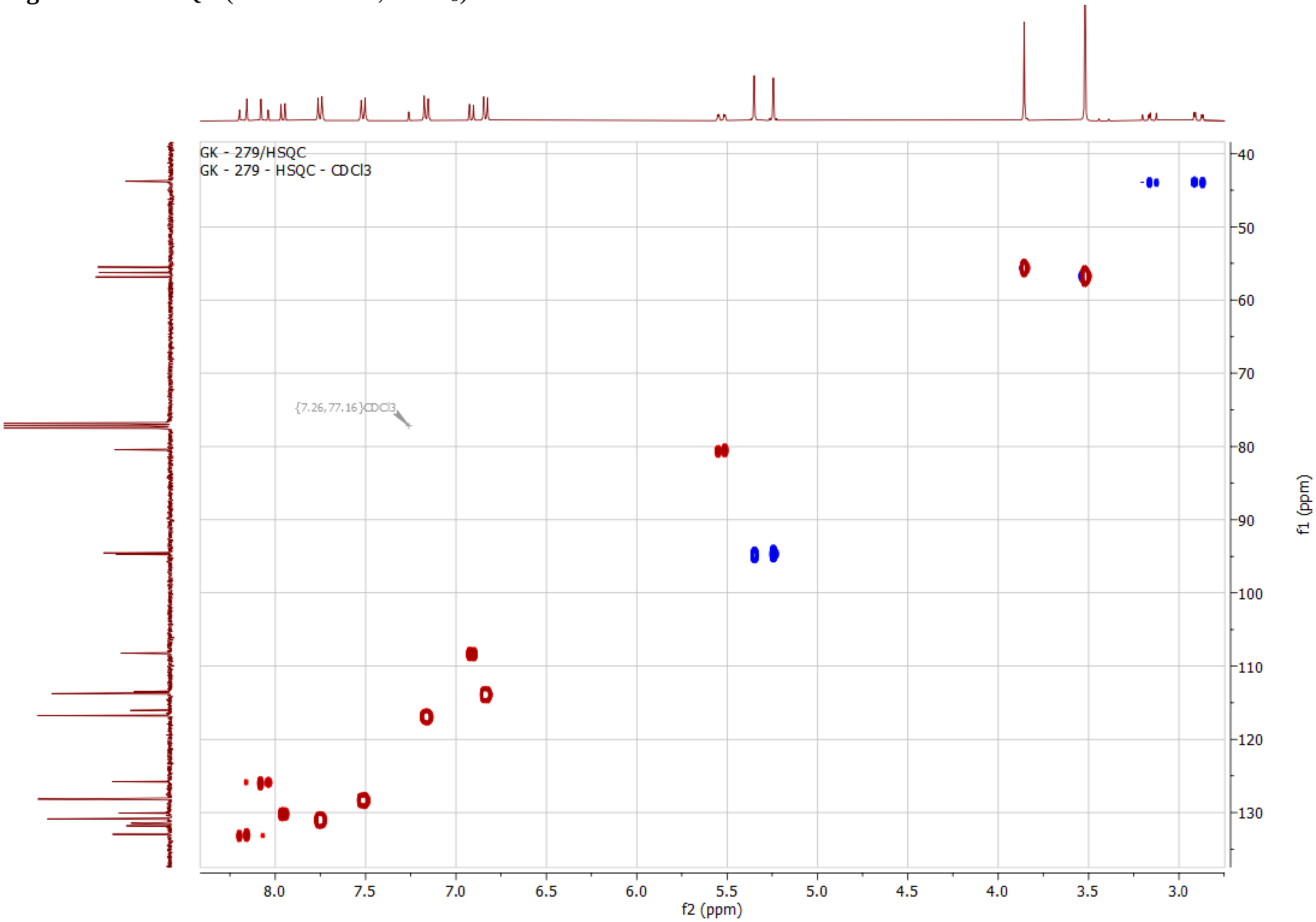

Figure S176: HMBC (400/100 MHz, $CDCl_3$) of **17af**

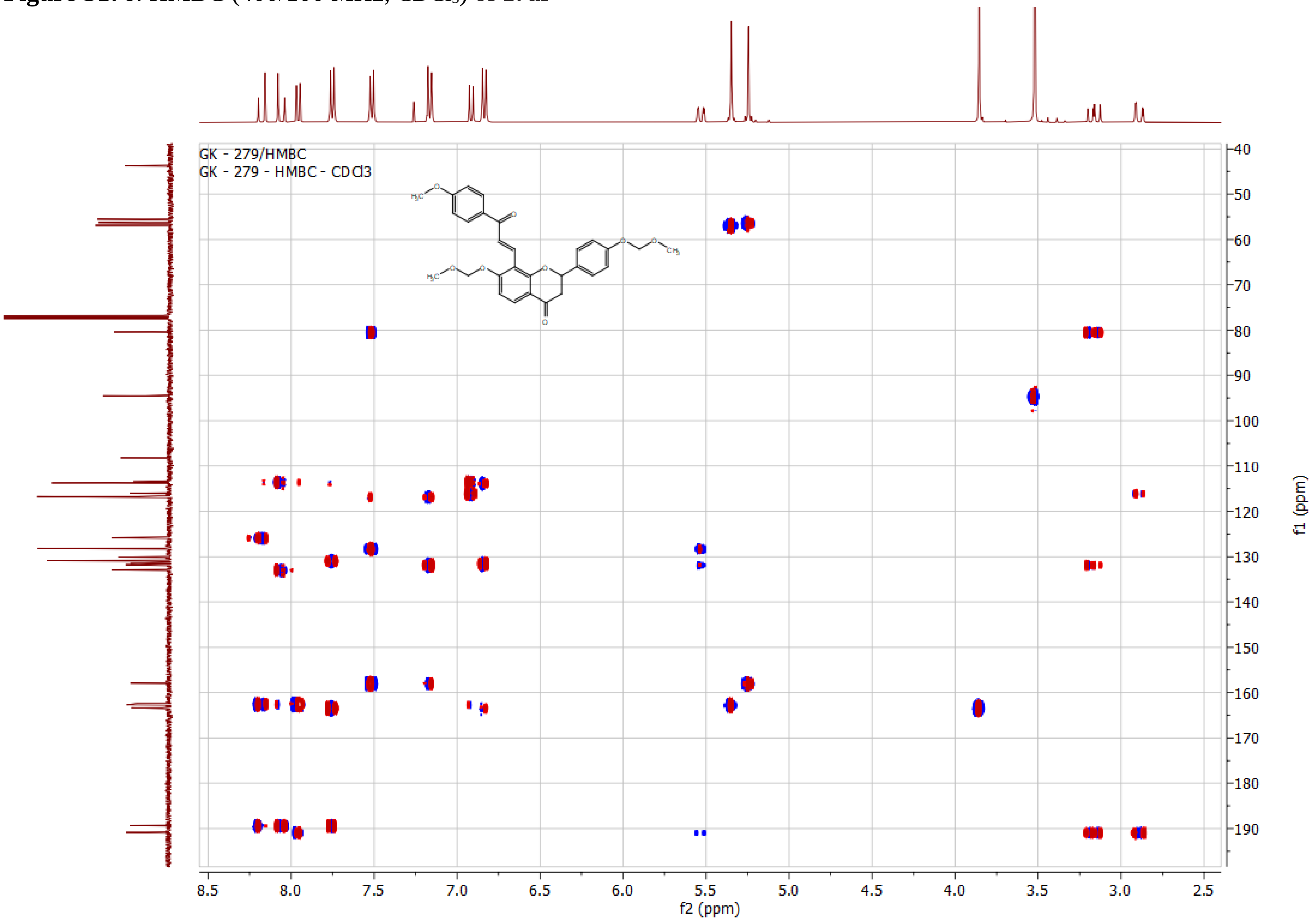

Figure S177: ^{1}H NMR (400 MHz, $CDCl_3$) of **17bf**

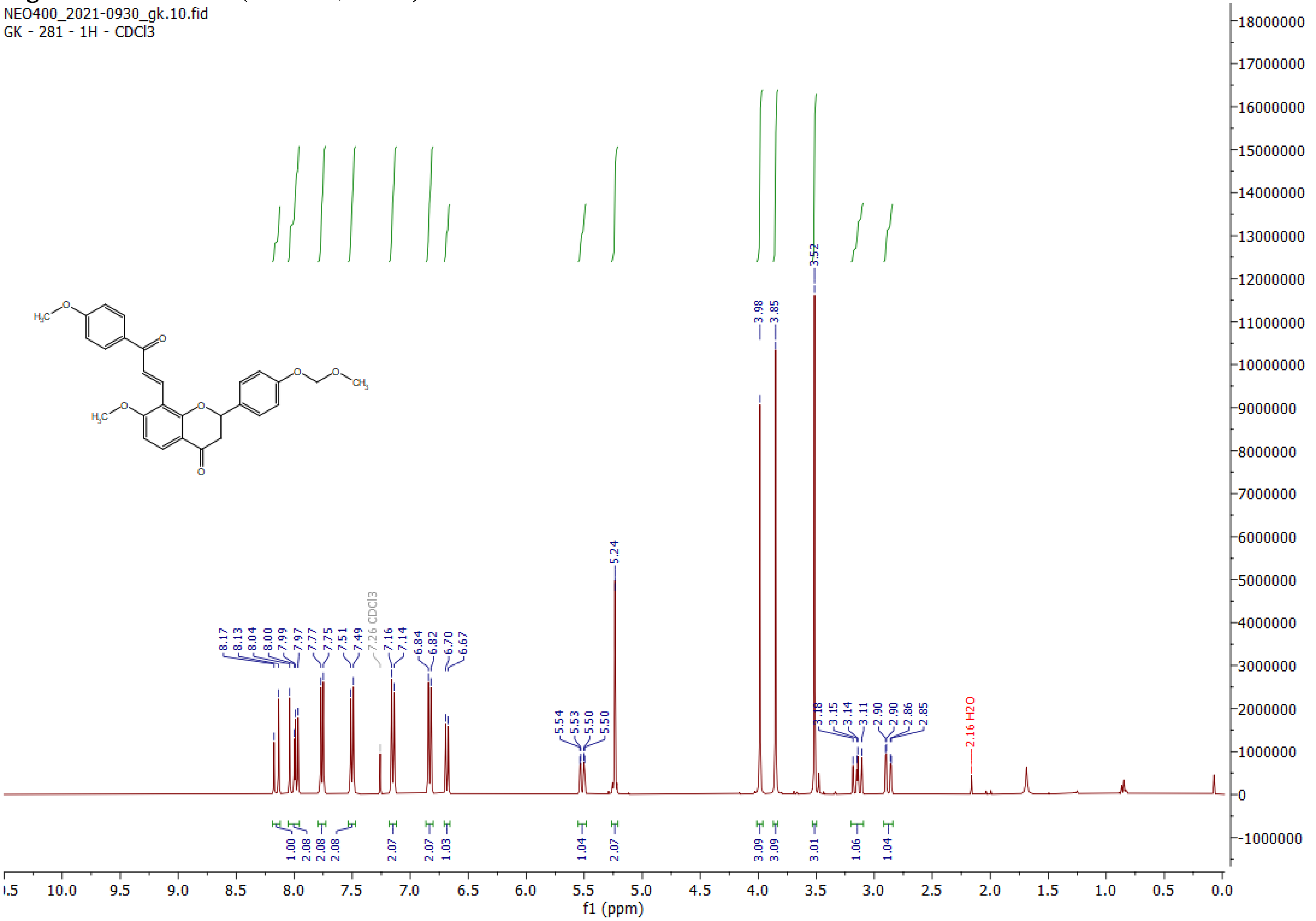

Figure S178: $^{13}C\{^{1}H\}$ NMR (100 MHz, $CDCl_3$) of **17bf**

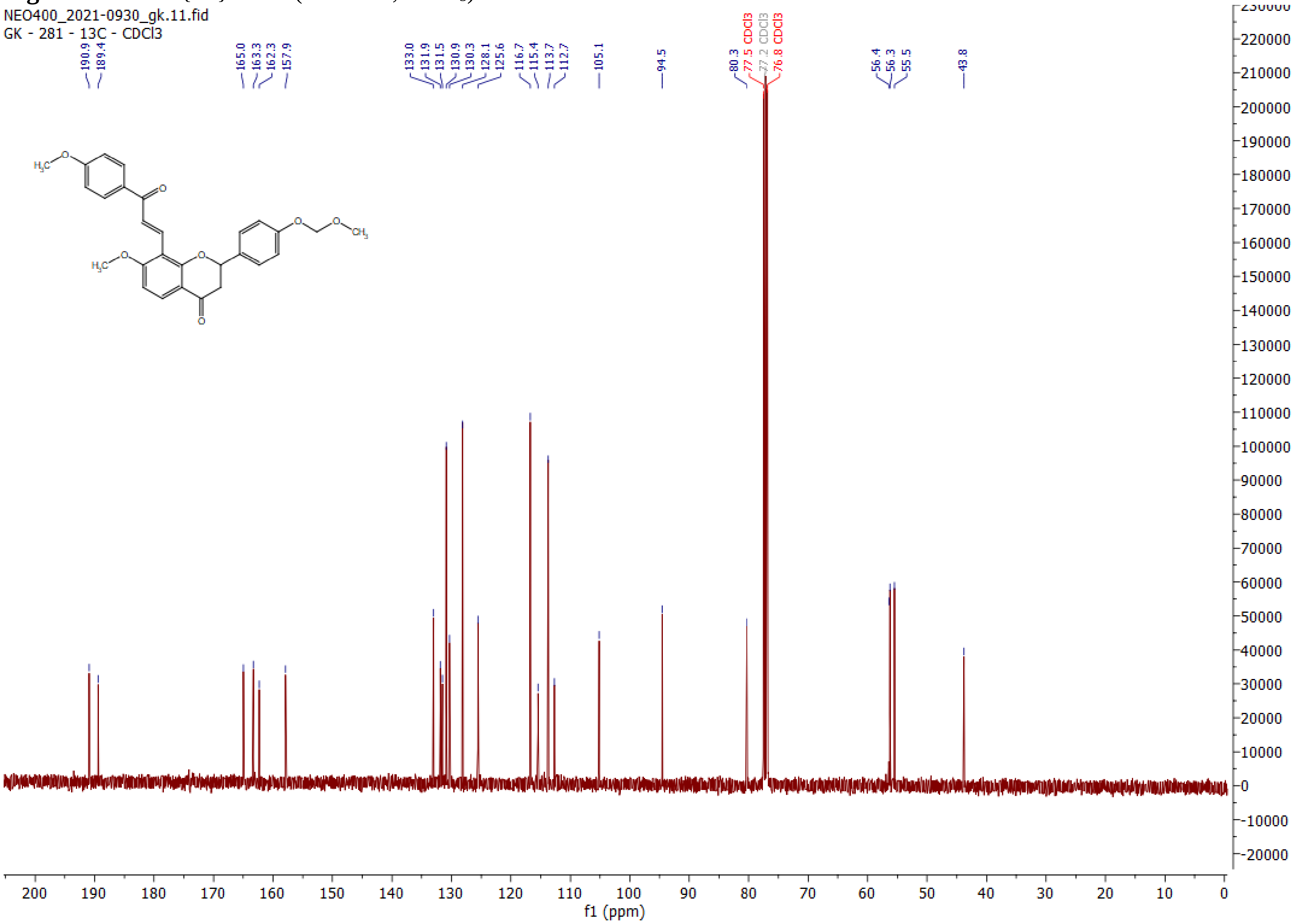

Figure S179: HSQC (400/100 MHz, $CDCl_3$) of **17bf**

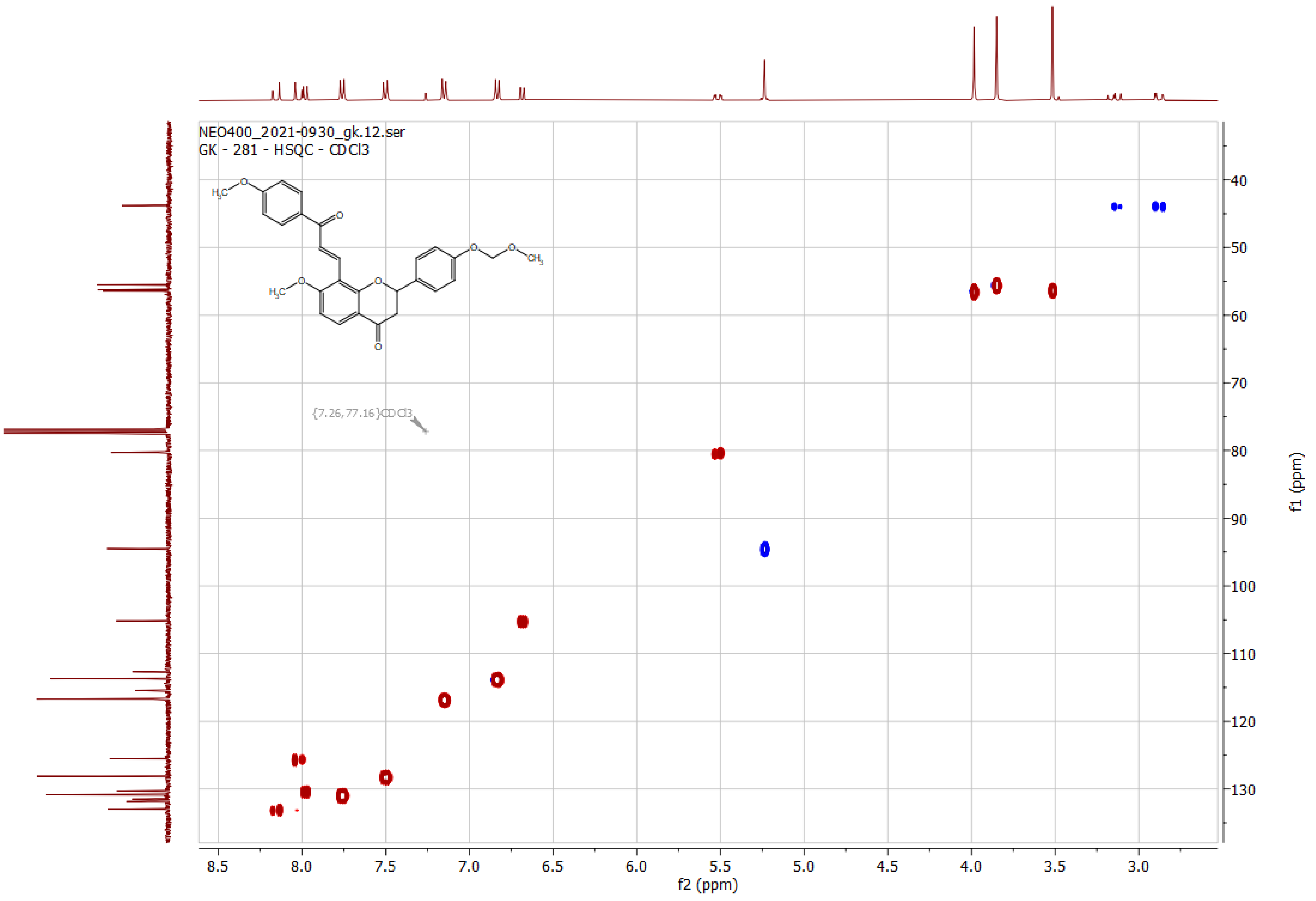

Figure S180: HMBC (400/100 MHz, $CDCl_3$) of **17bf**

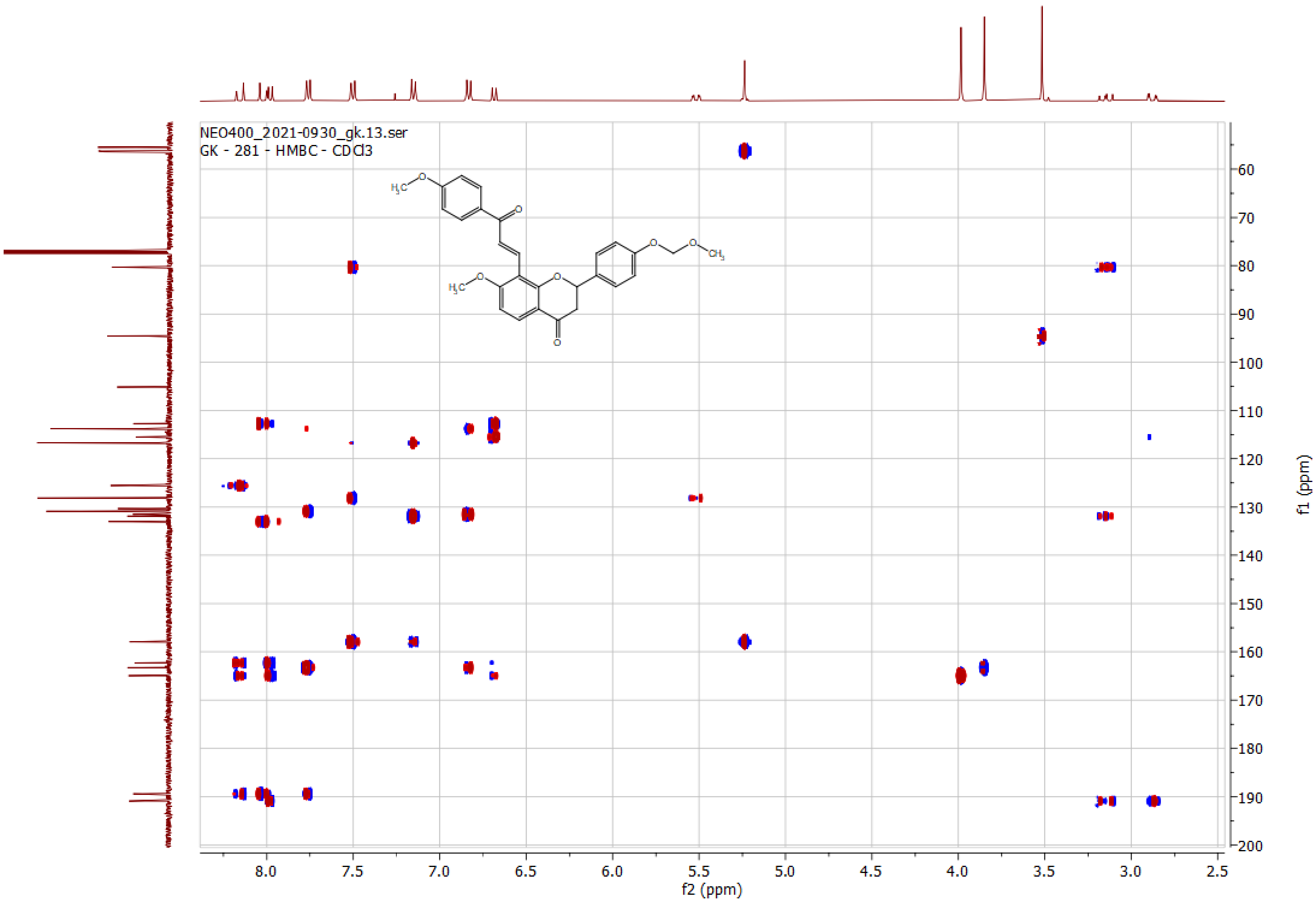

Figure S181: ^{1}H NMR (400 MHz, $CDCl_3$) of **17cf**

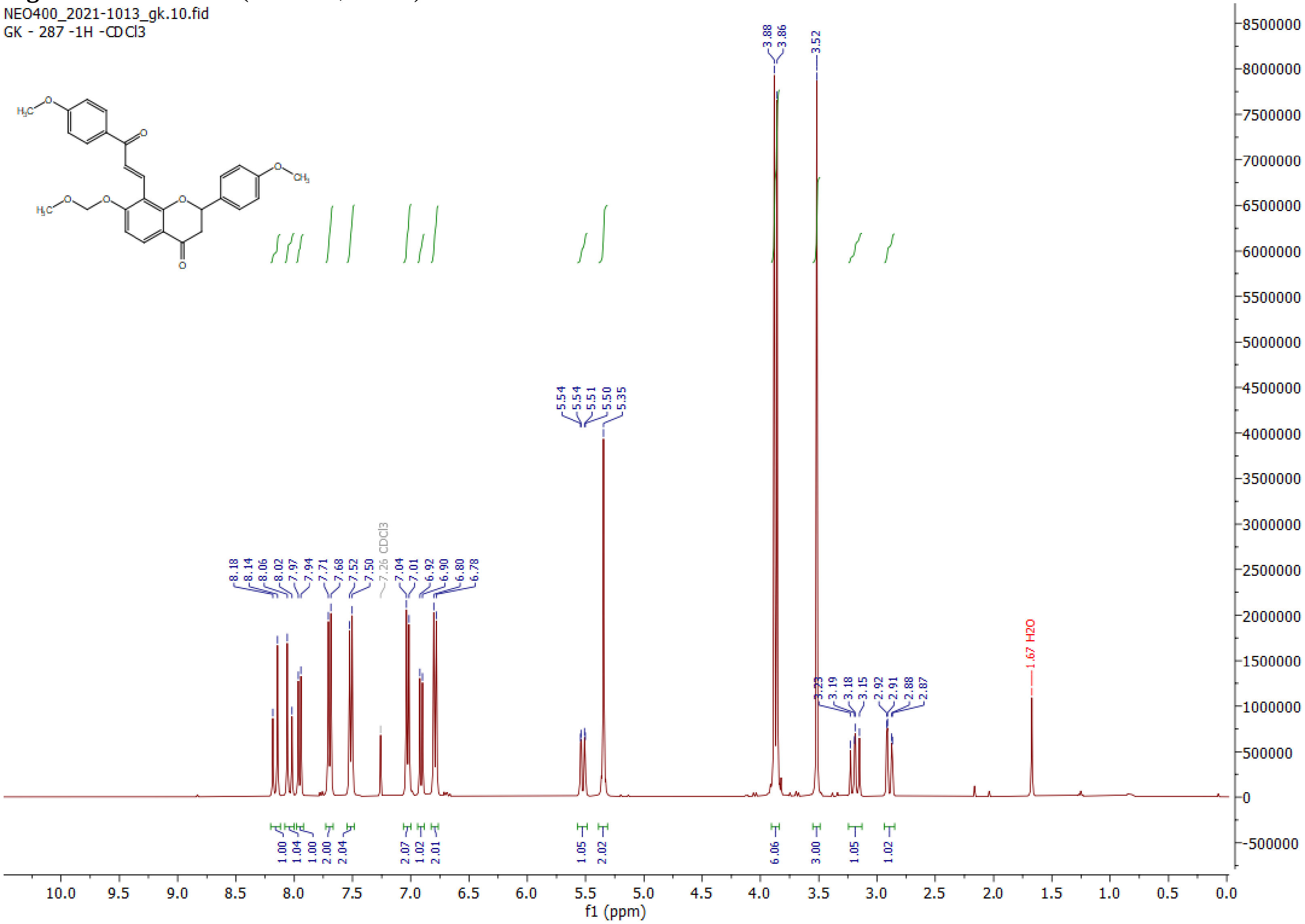

Figure S182: $^{13}C\{^1H\}$ NMR (100 MHz, $CDCl_3$) of **17cf**

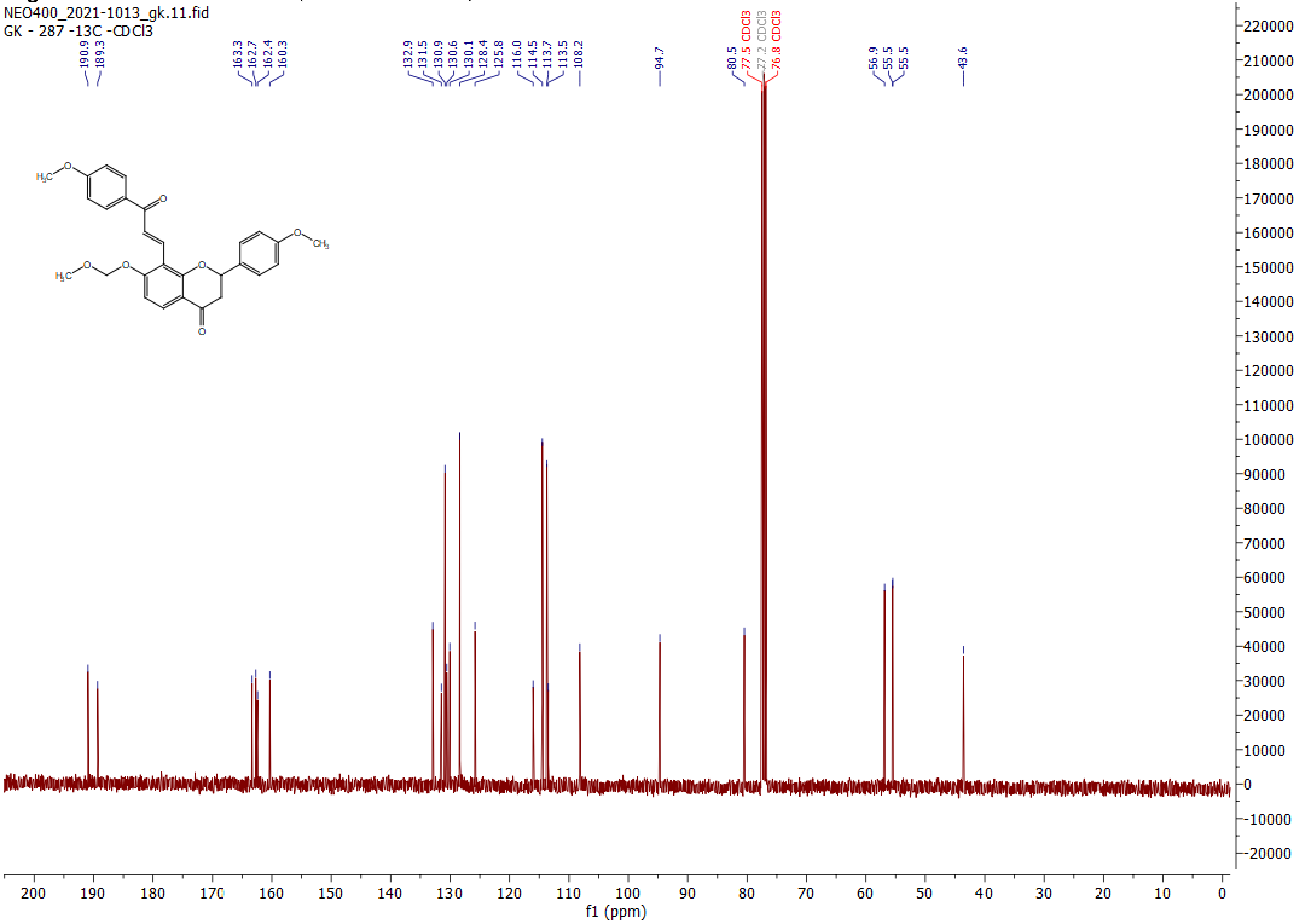

Figure S183: HSQC (400/100 MHz, $CDCl_3$) of **17cf**

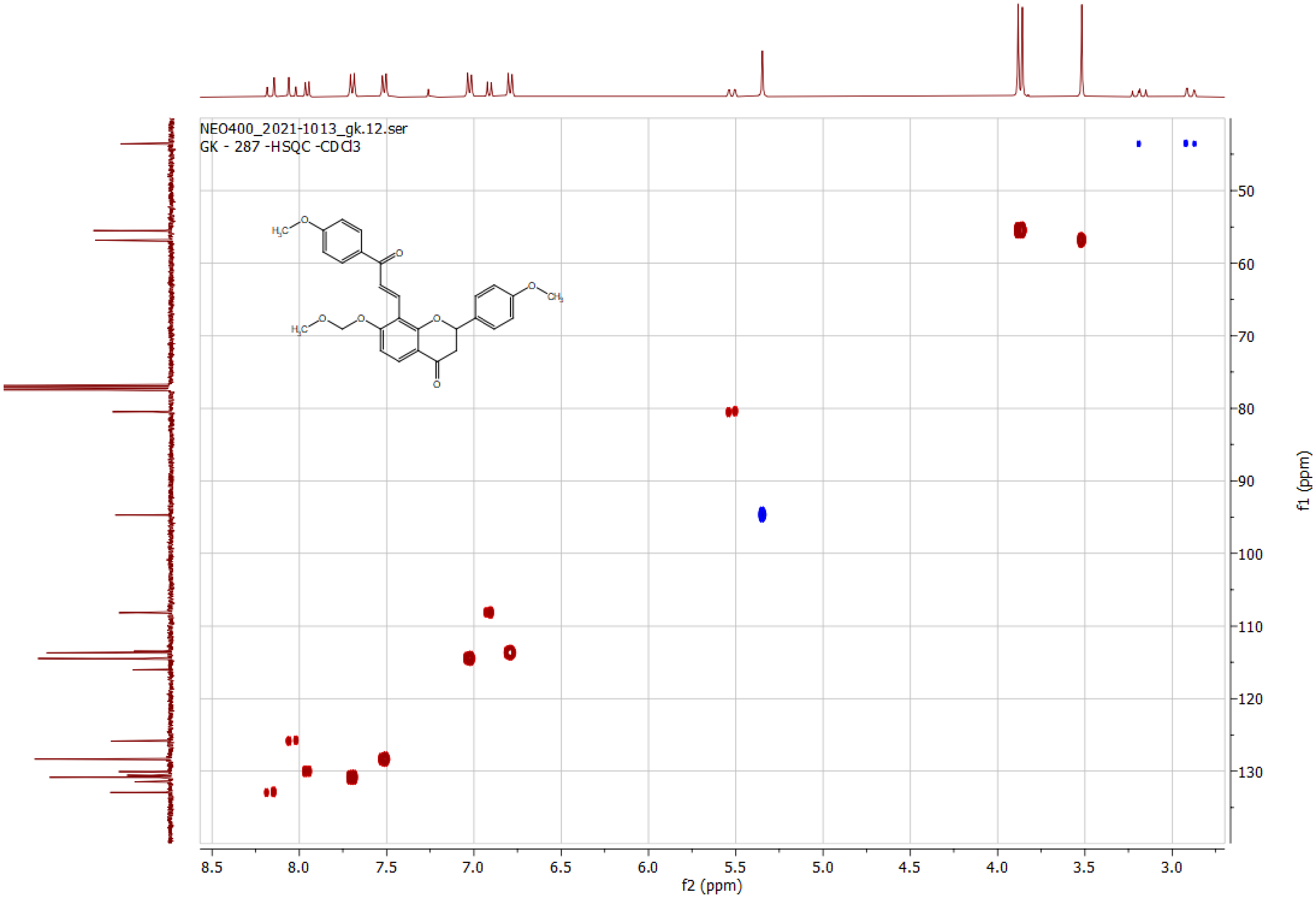

Figure S184: HMBC (400/100 MHz, $CDCl_3$) of **17cf**

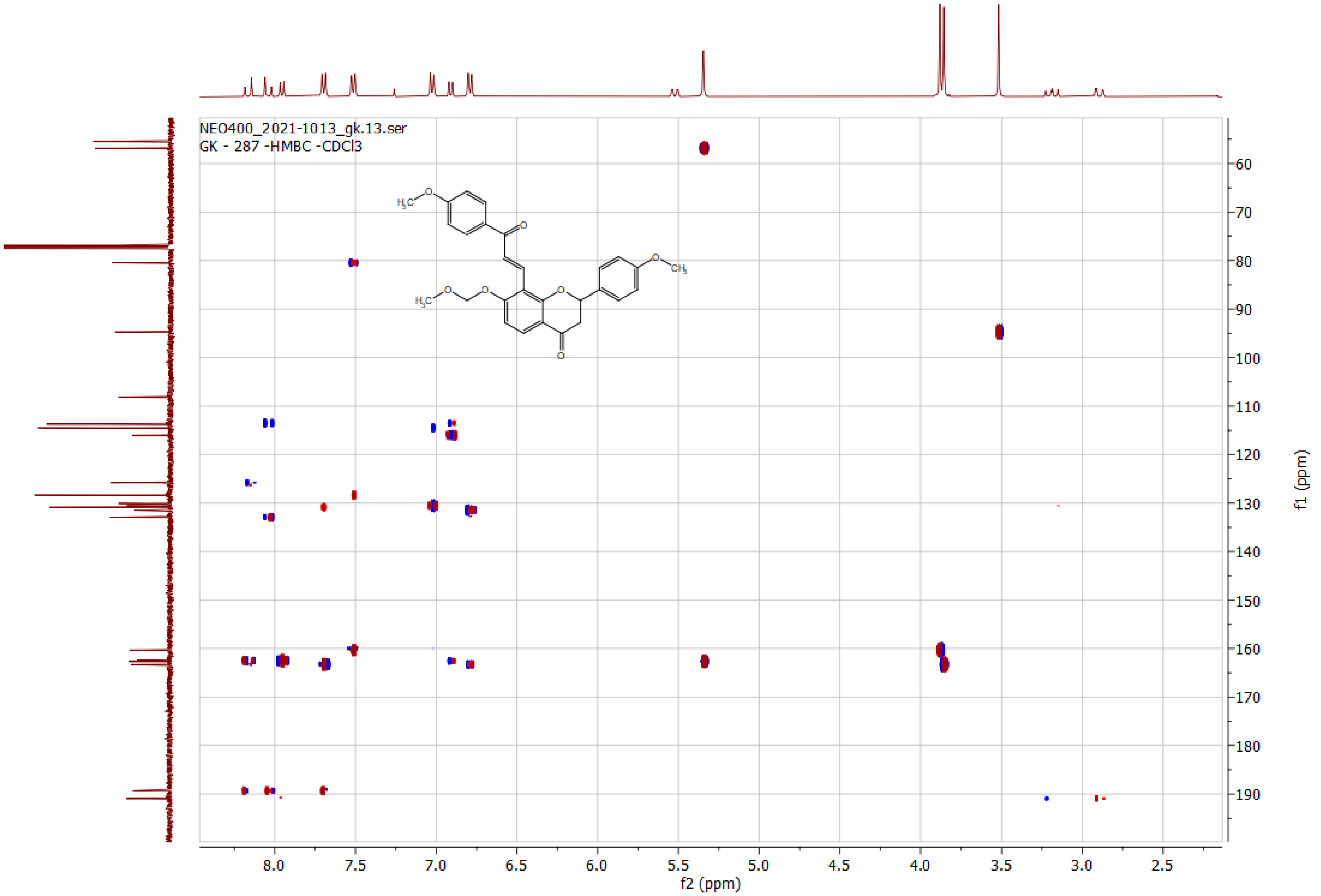

Figure S185: ^{1}H NMR (400 MHz, $CDCl_3$) of **18af**

NEO400_2021-1021_gk.10.fid
GK - 288 -1H - DMSO

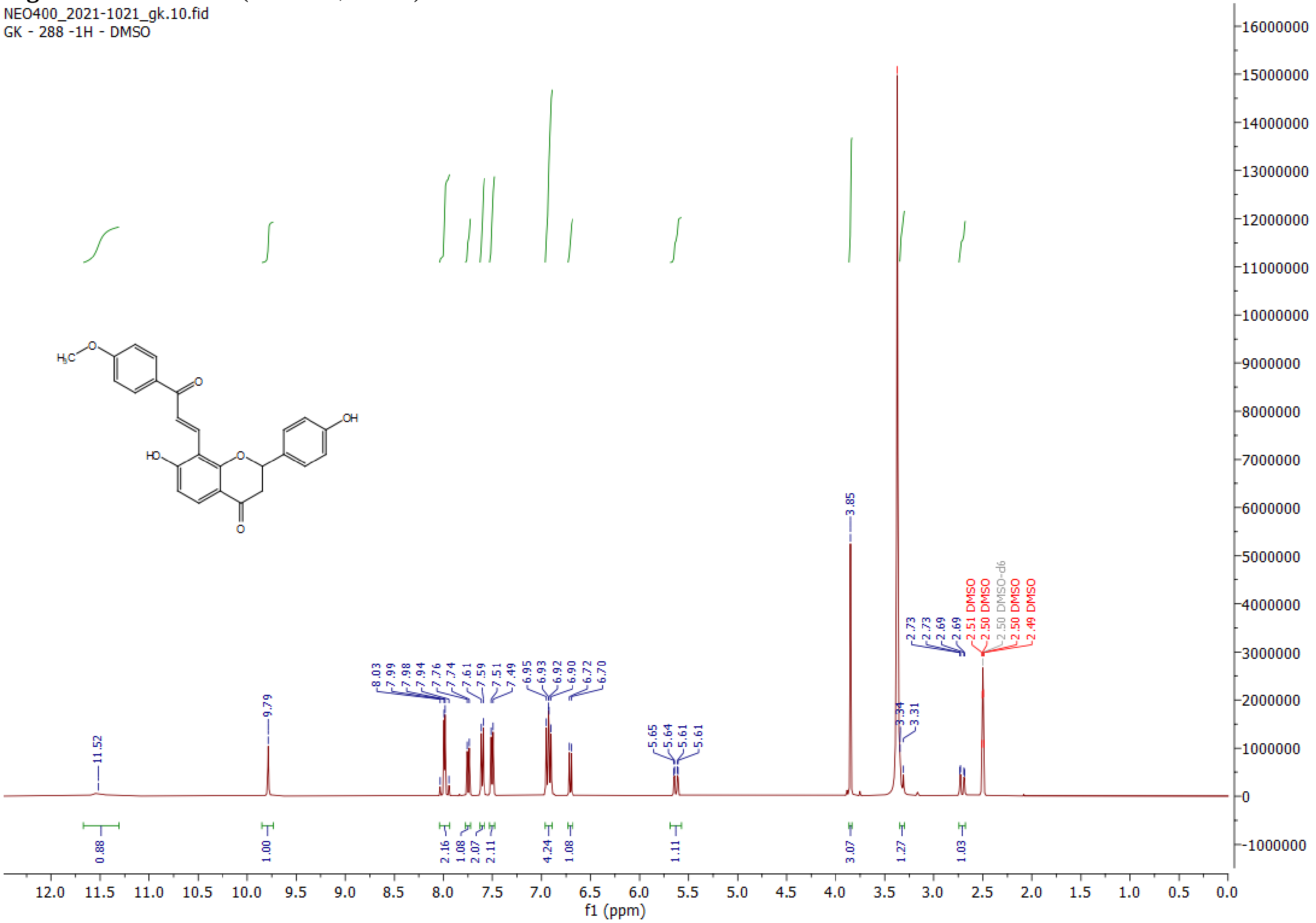

Figure S186: $^{13}C\{^{1}H\}$ NMR (100 MHz, $CDCl_3$) of **18af**

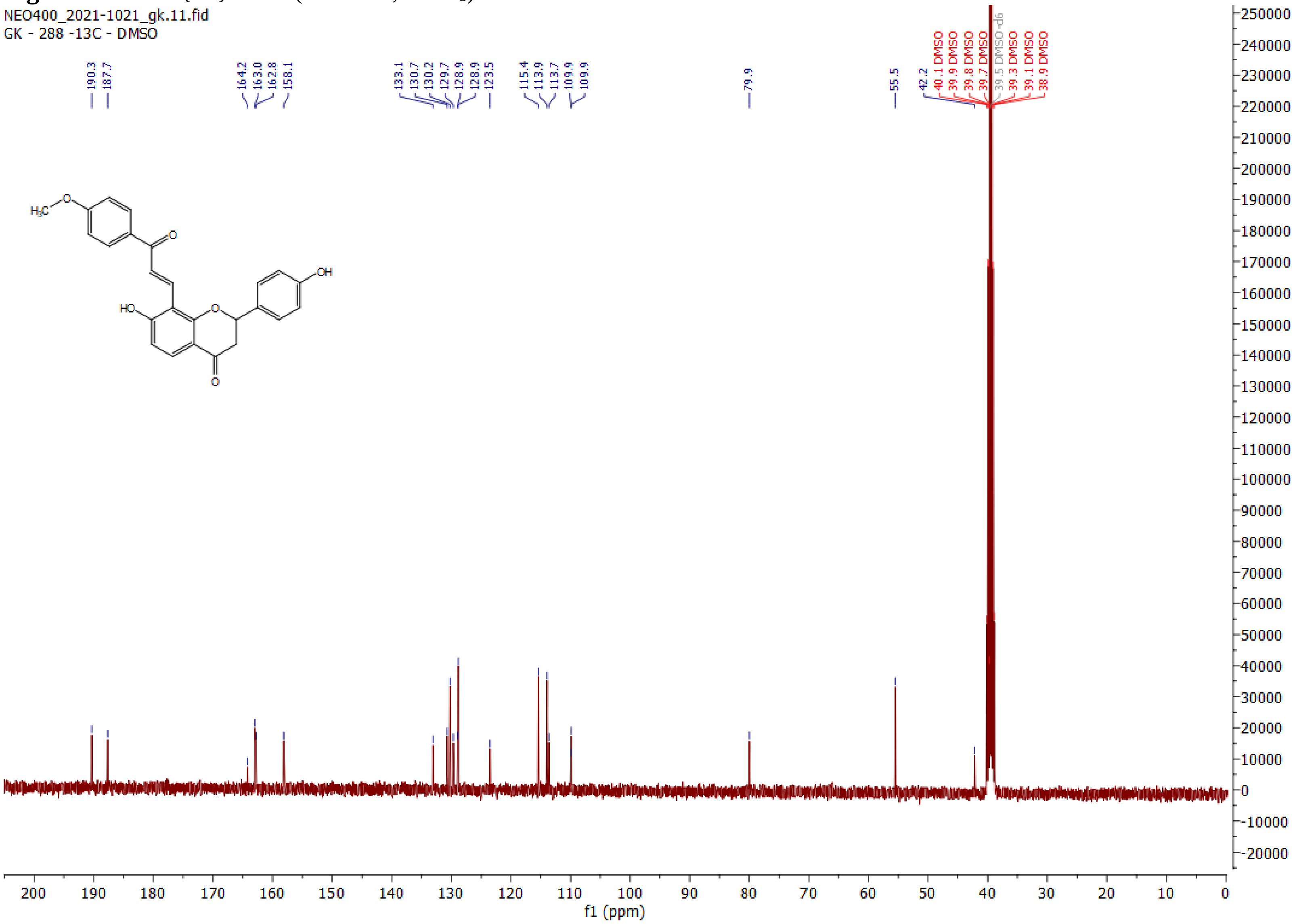

Figure S187: ^{1}H NMR (400 MHz, $CDCl_3$) of **18bf**

NEO400_2021-1018_gk.10.fid
GK - 289 -1H - DMSO

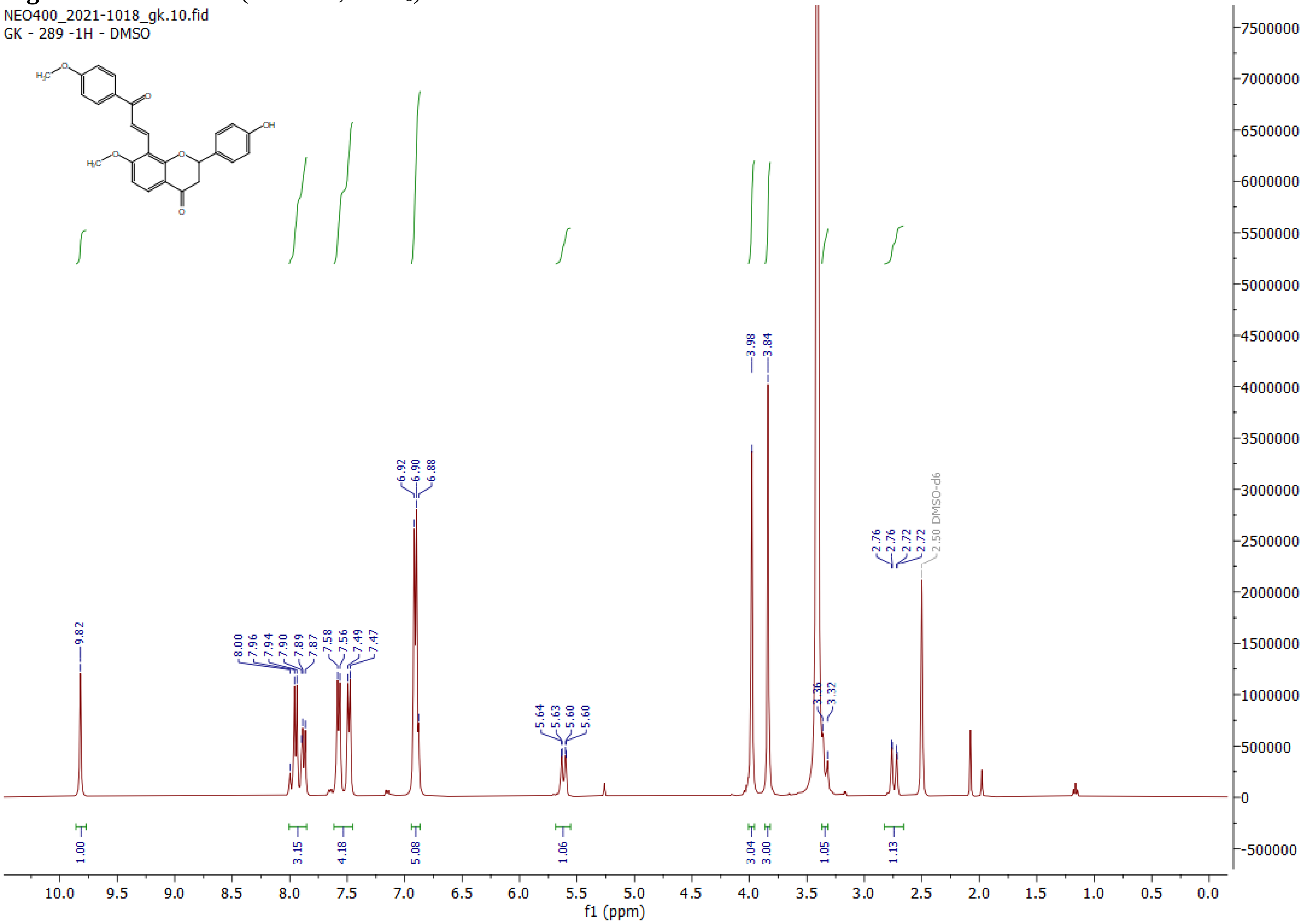

Figure S188: $^{13}C\{^{1}H\}$ NMR (100 MHz, $CDCl_3$) of **18bf**

NEO400_2021-1018_gk.11.fid
GK - 289 -13C - DMSO

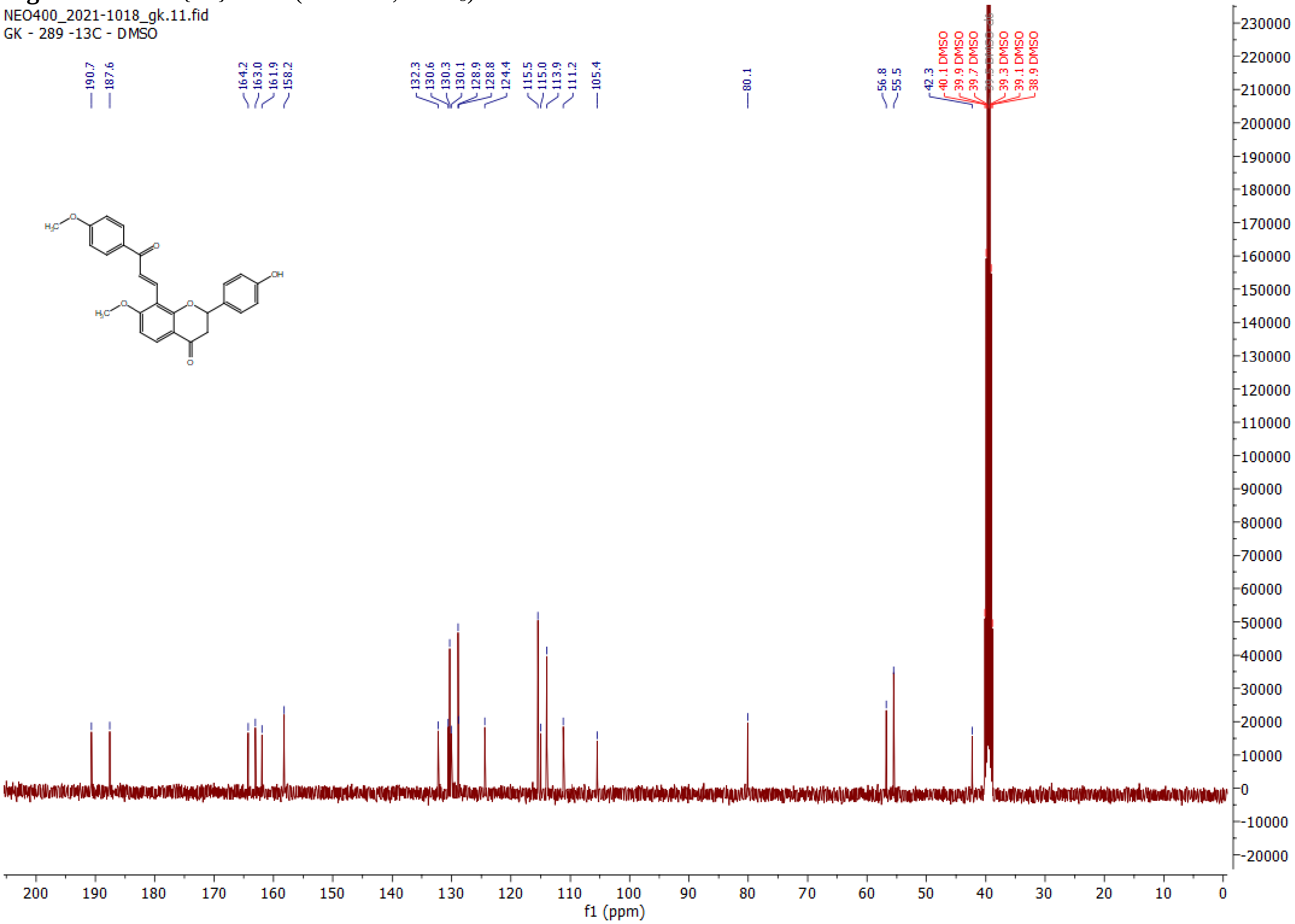

Figure S189: ^{1}H NMR (400 MHz, $CDCl_3$) of **18cf**

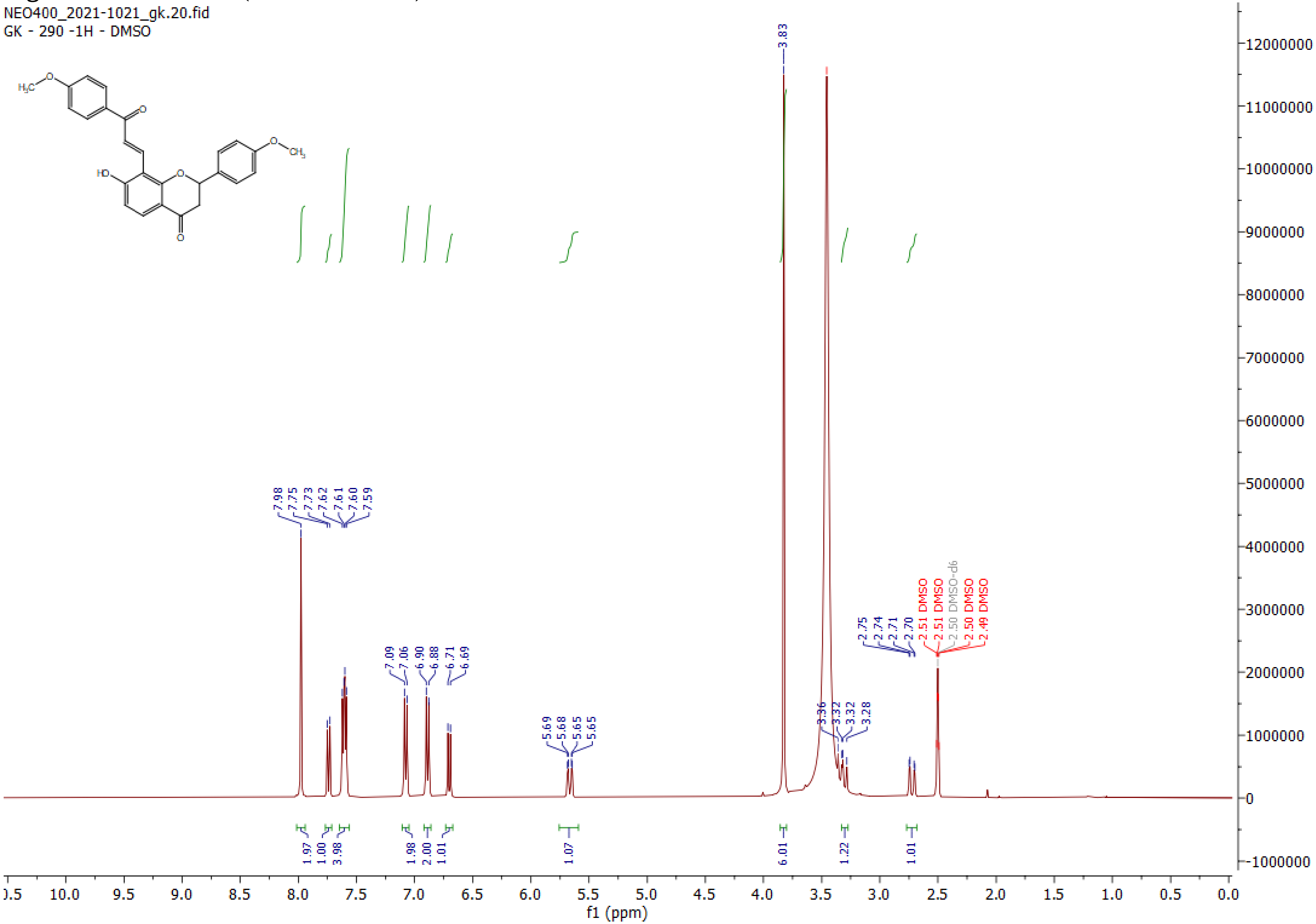

Figure S190: $^{13}C\{^1H\}$ NMR (100 MHz, $CDCl_3$) of **18cf**

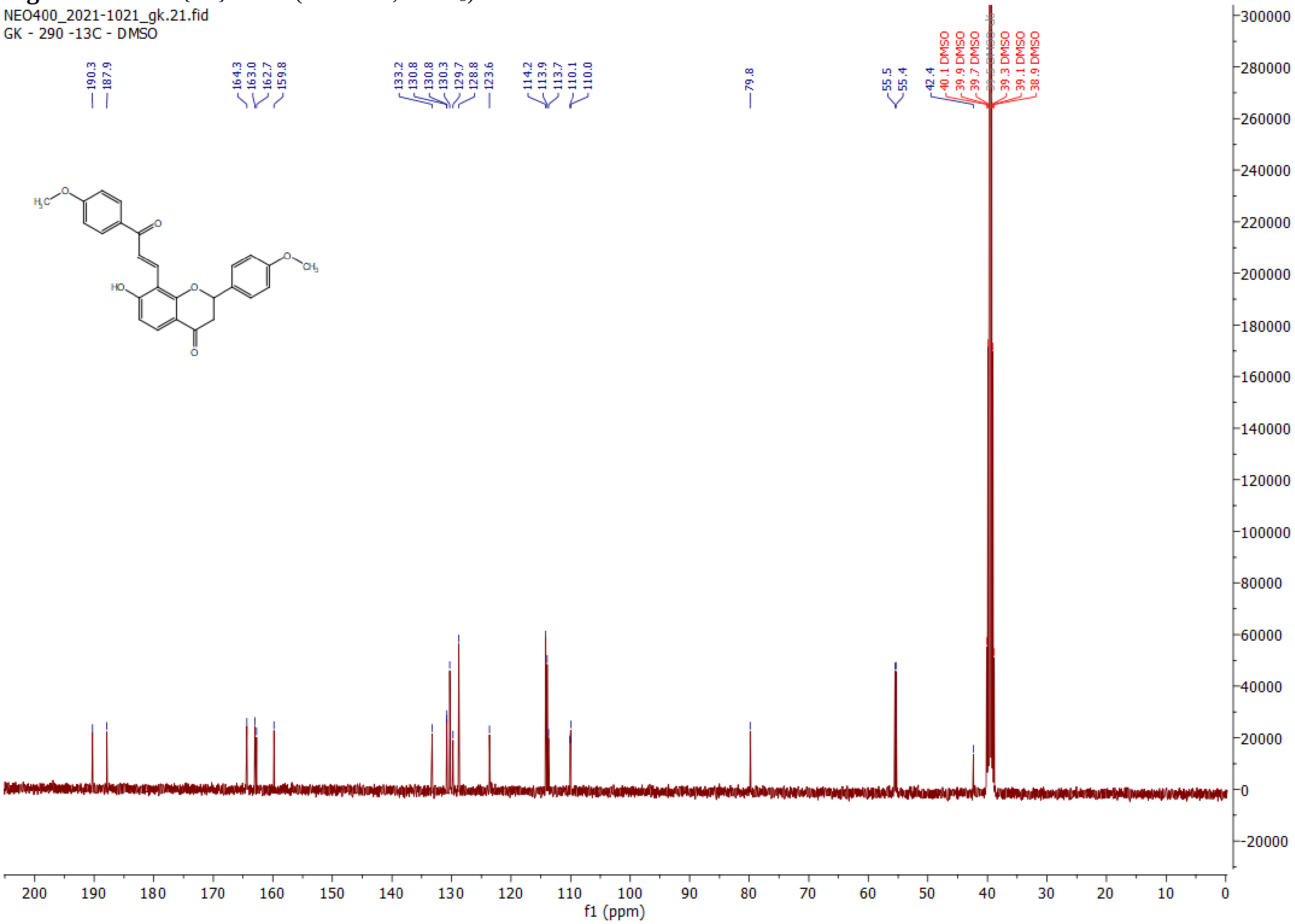

Figure S191: NMR-Signal assignment and selected HMBC-couplings for compound **20**

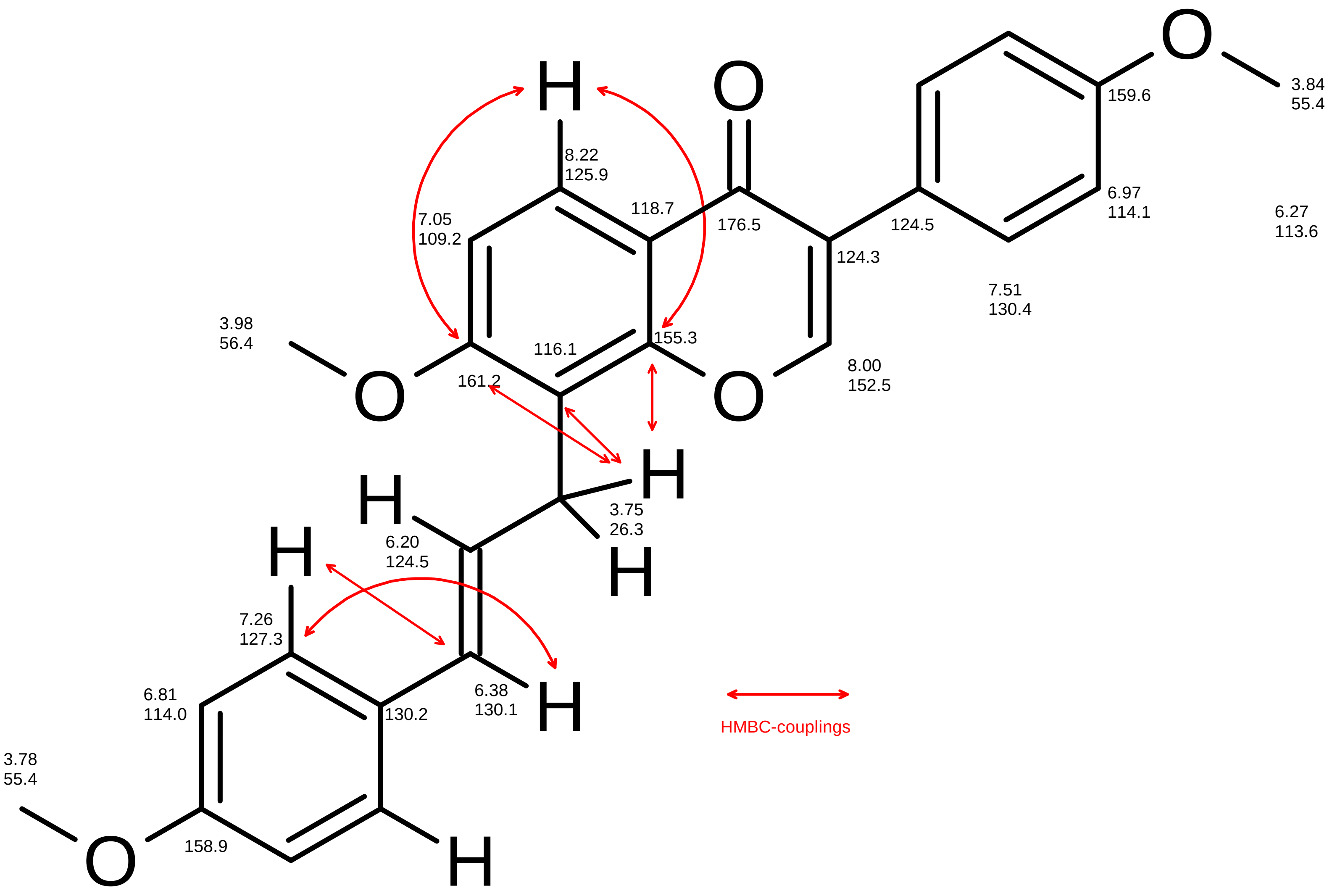

Figure S192: ^{1}H NMR (400 MHz, $CDCl_3$) of **20**

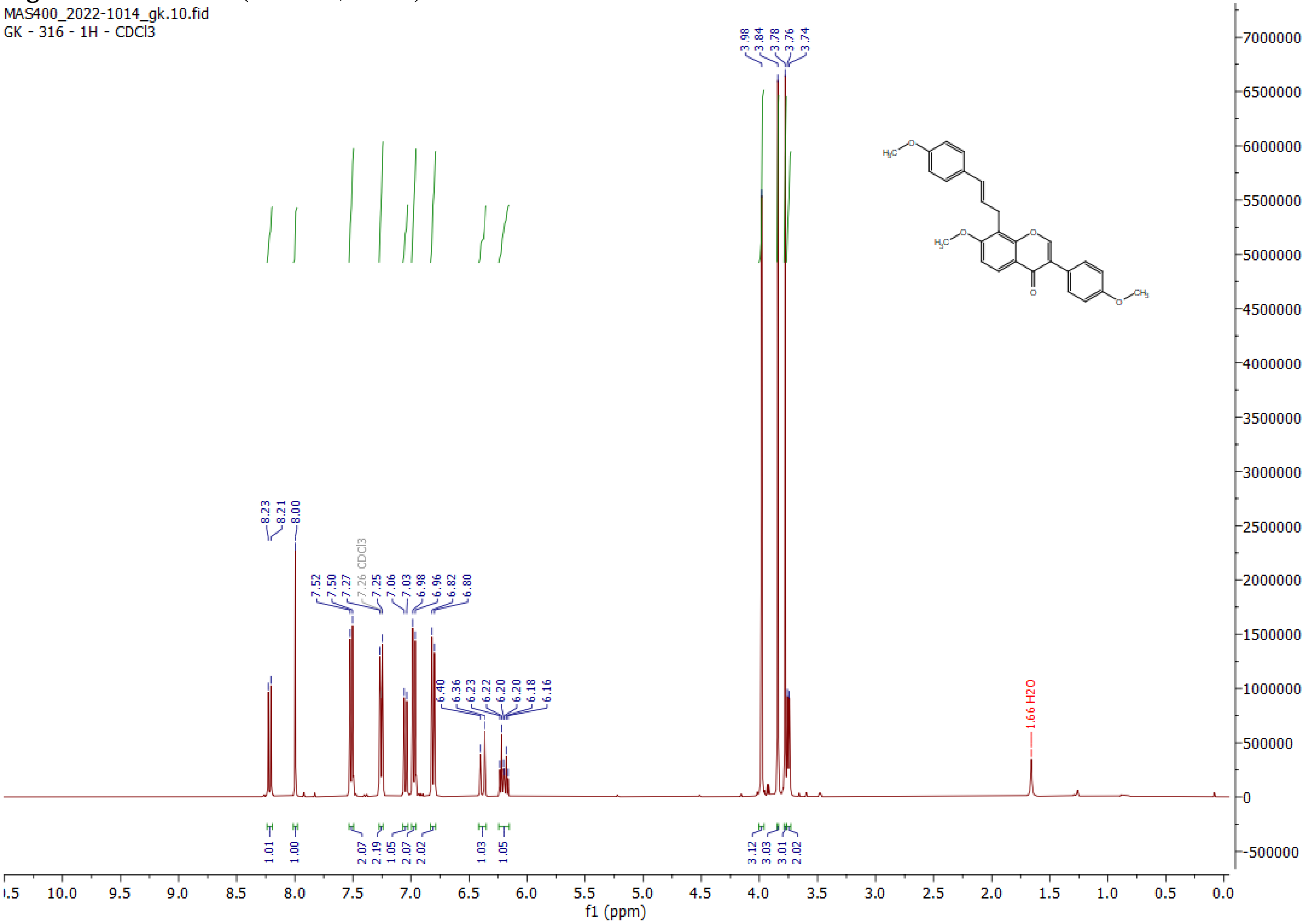

Figure S193: $^{13}C\{^1H\}$ NMR (100 MHz, $CDCl_3$) of **20**

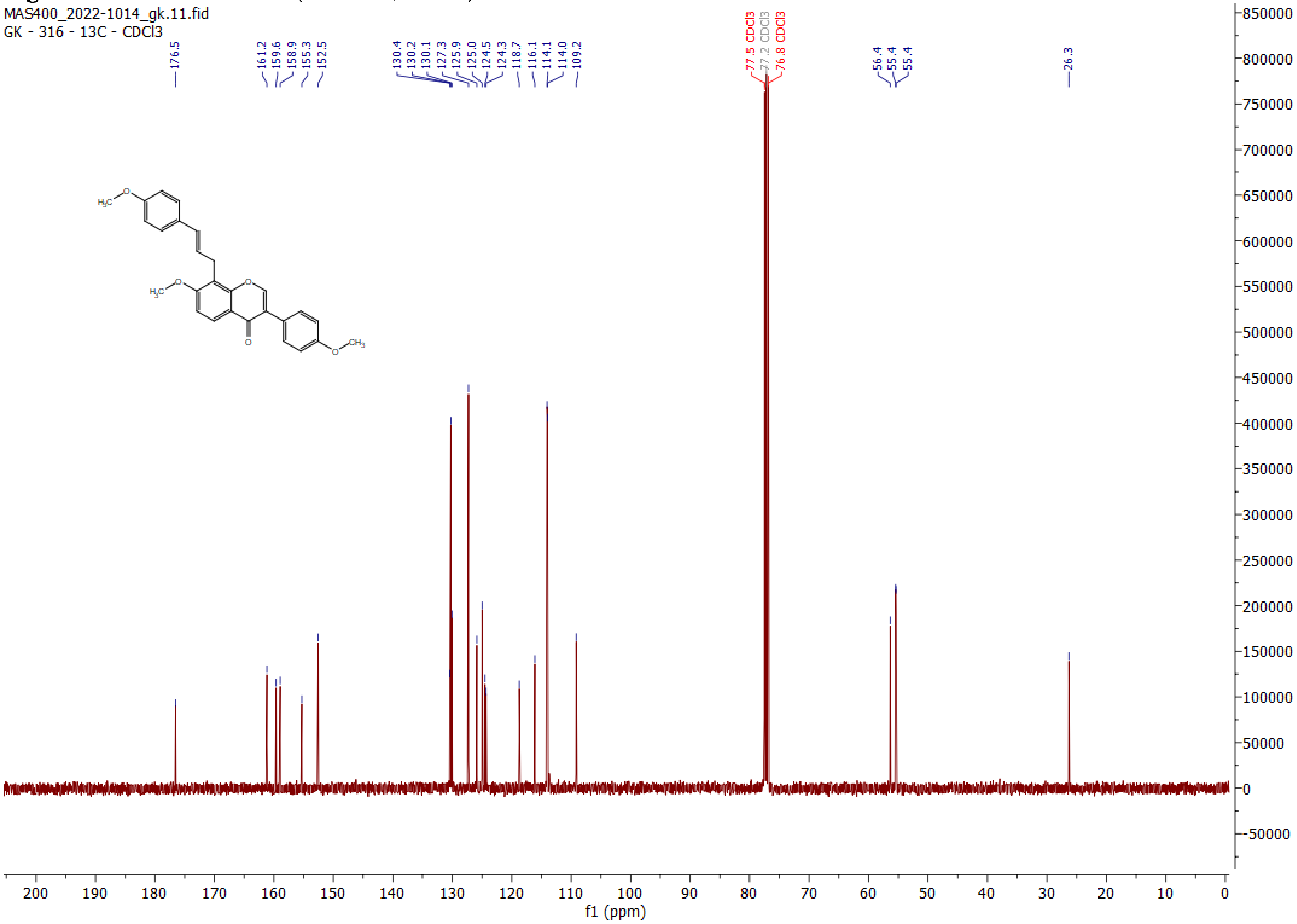

Figure S194: HSQC (400/100 MHz, $CDCl_3$) of **20**

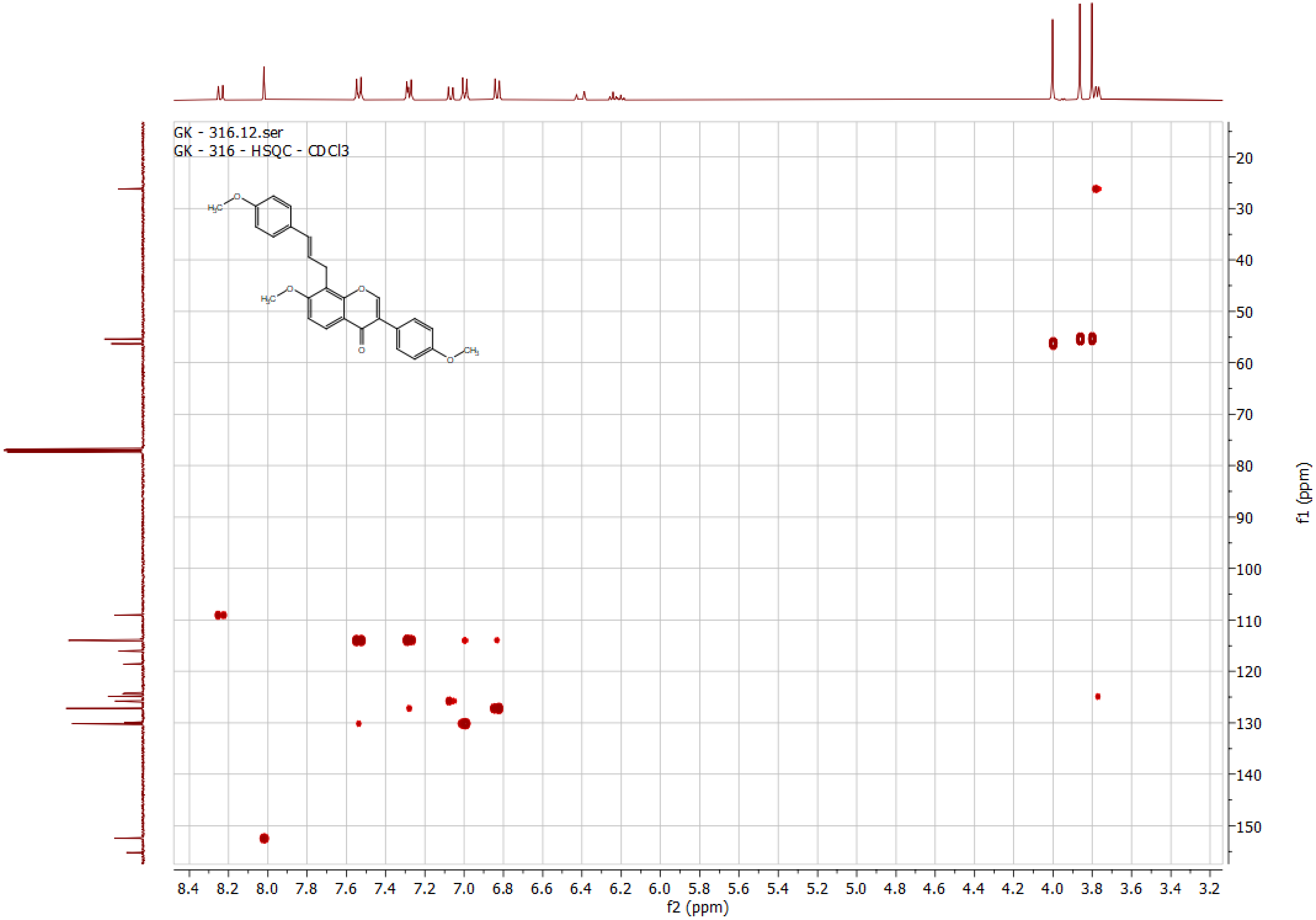

Figure S195: HMBC (400/100 MHz, $CDCl_3$) of **20**

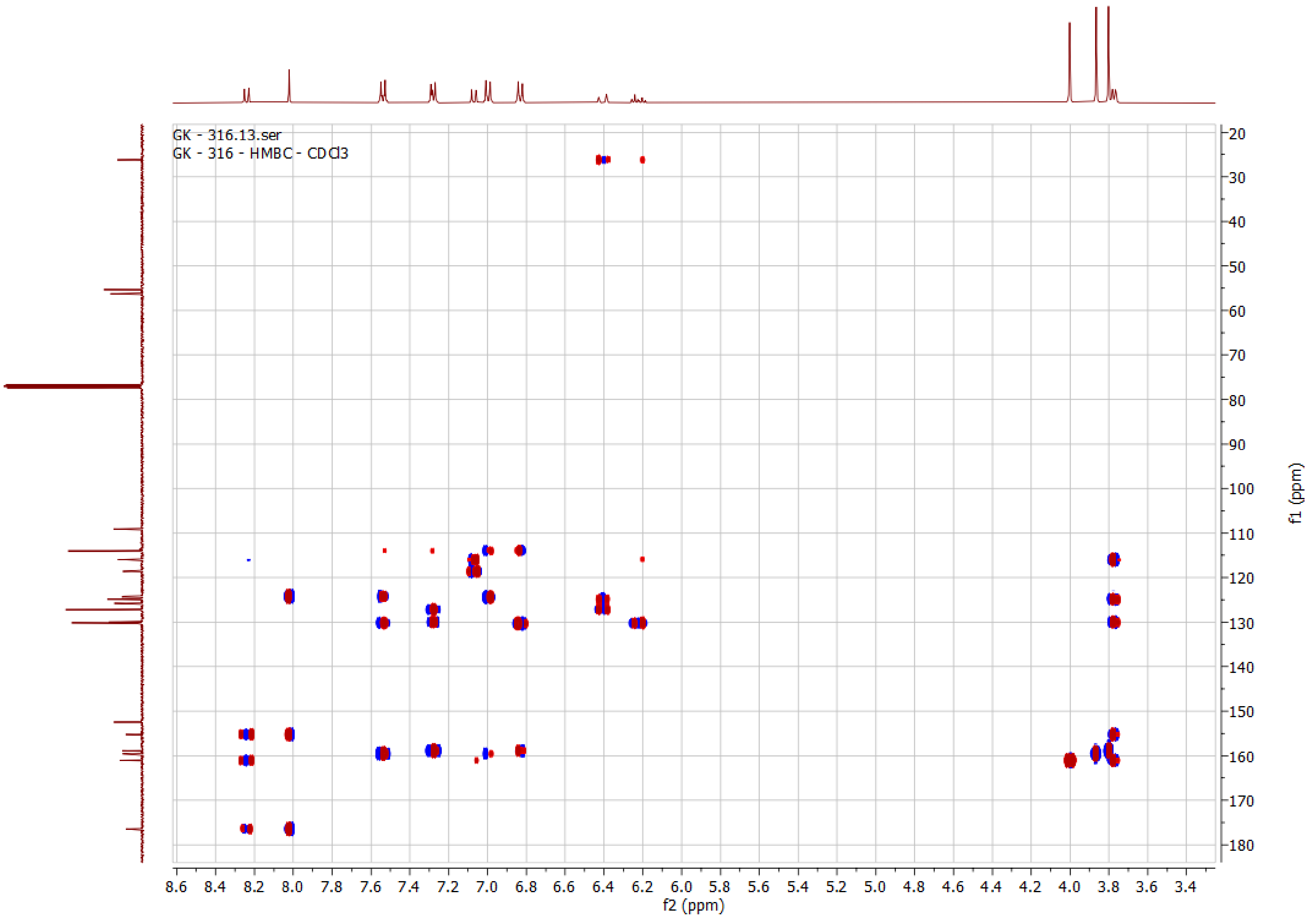

Figure S196: NMR-Signal assignment and selected HMBC-couplings for compound **21**

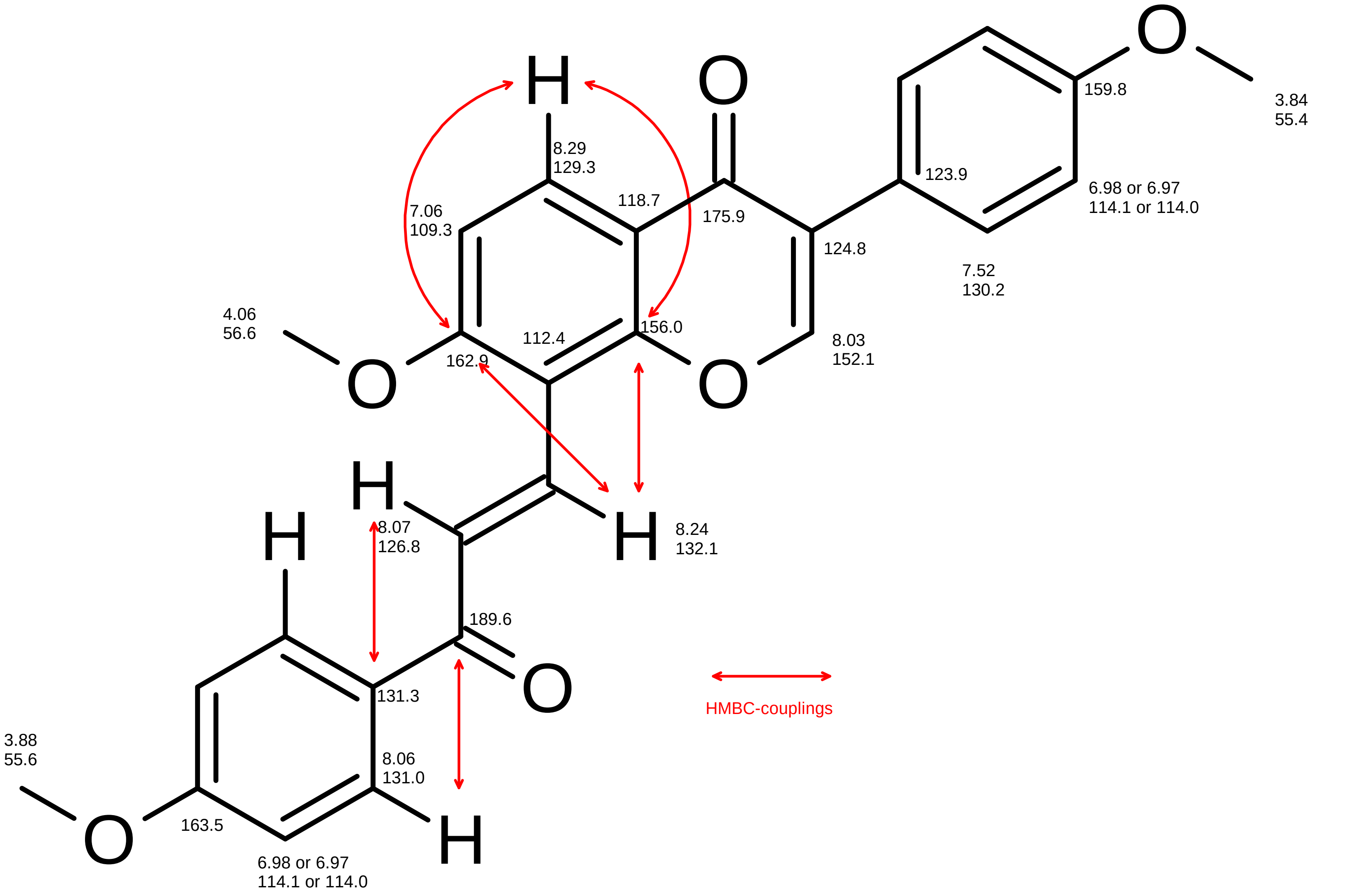

Figure S197: ^{1}H NMR (400 MHz, $CDCl_3$) of **21**

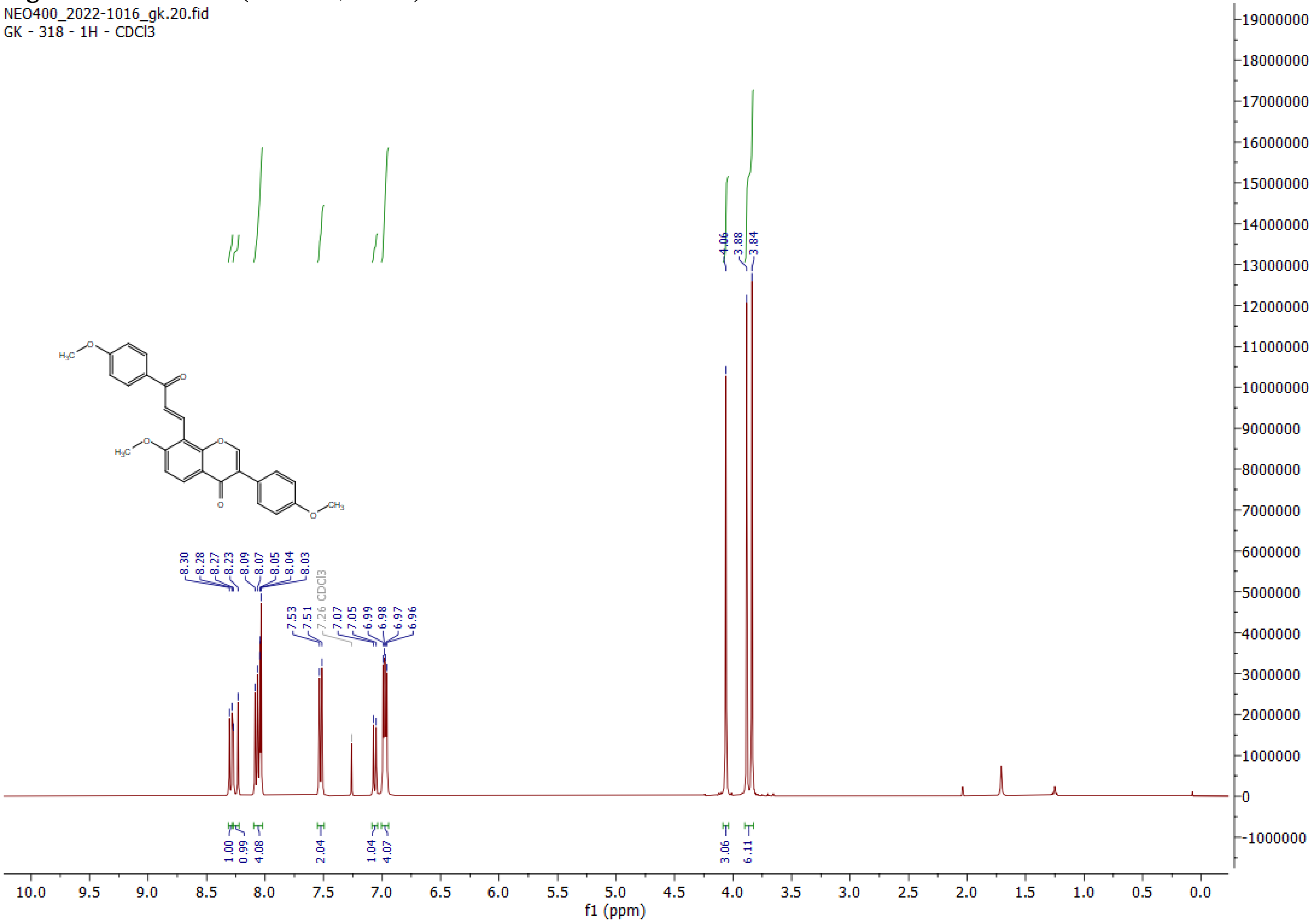

Figure S198: $^{13}C\{^1H\}$ NMR (100 MHz, $CDCl_3$) of **21**

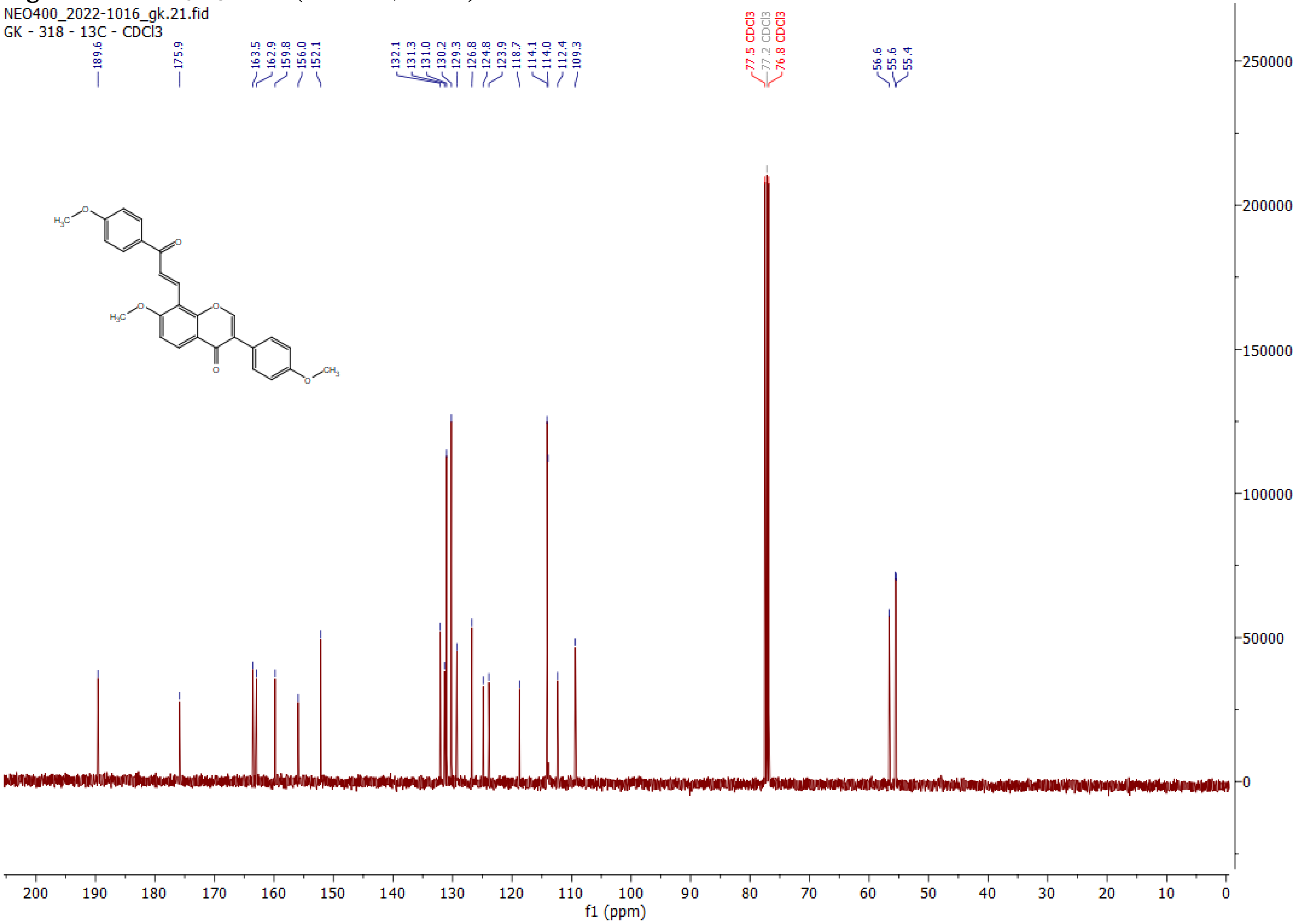

Figure S199: HSQC (400/100 MHz, $CDCl_3$) of **21**

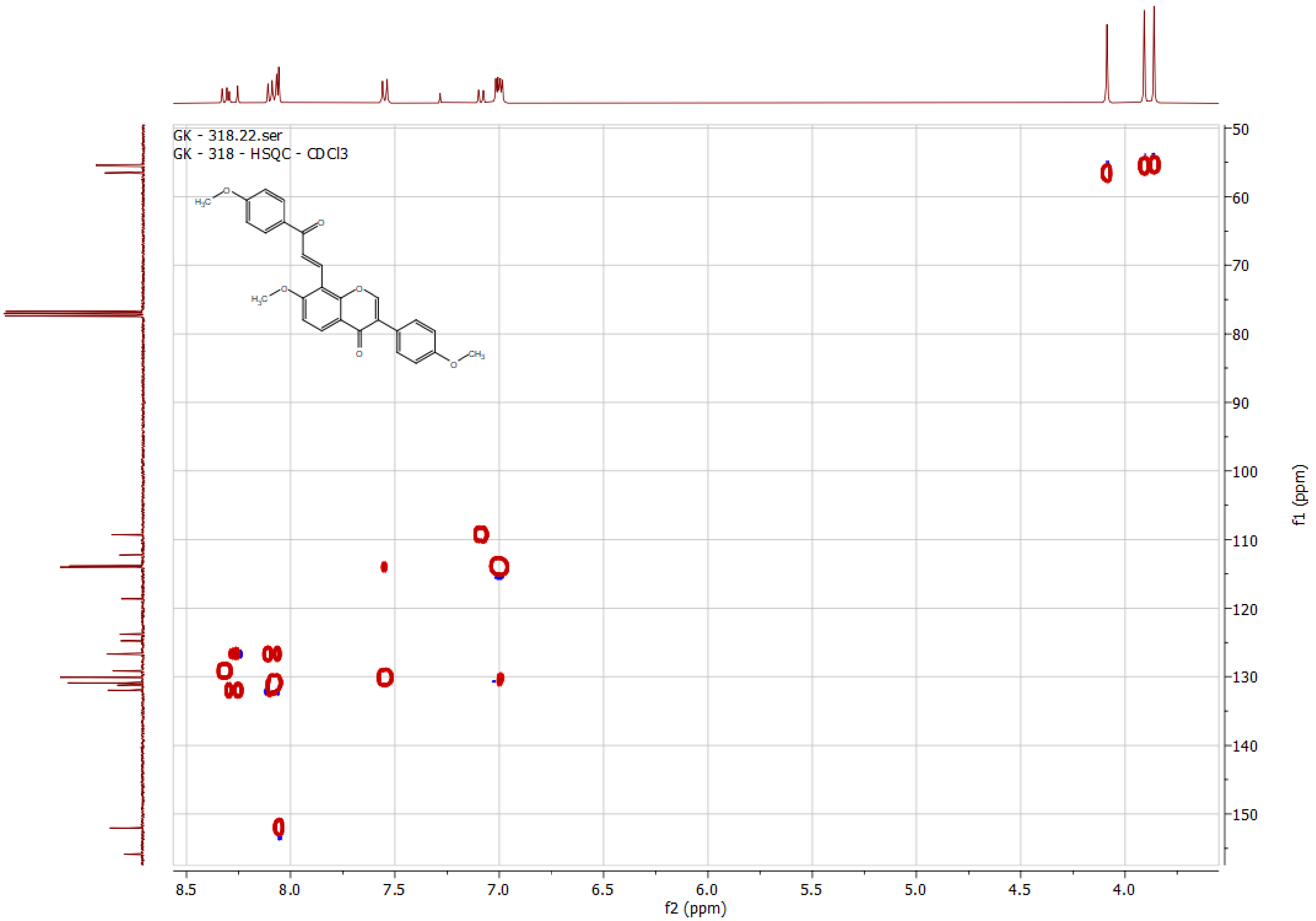

Figure S200: HMBC (400/100 MHz, $CDCl_3$) of **21**

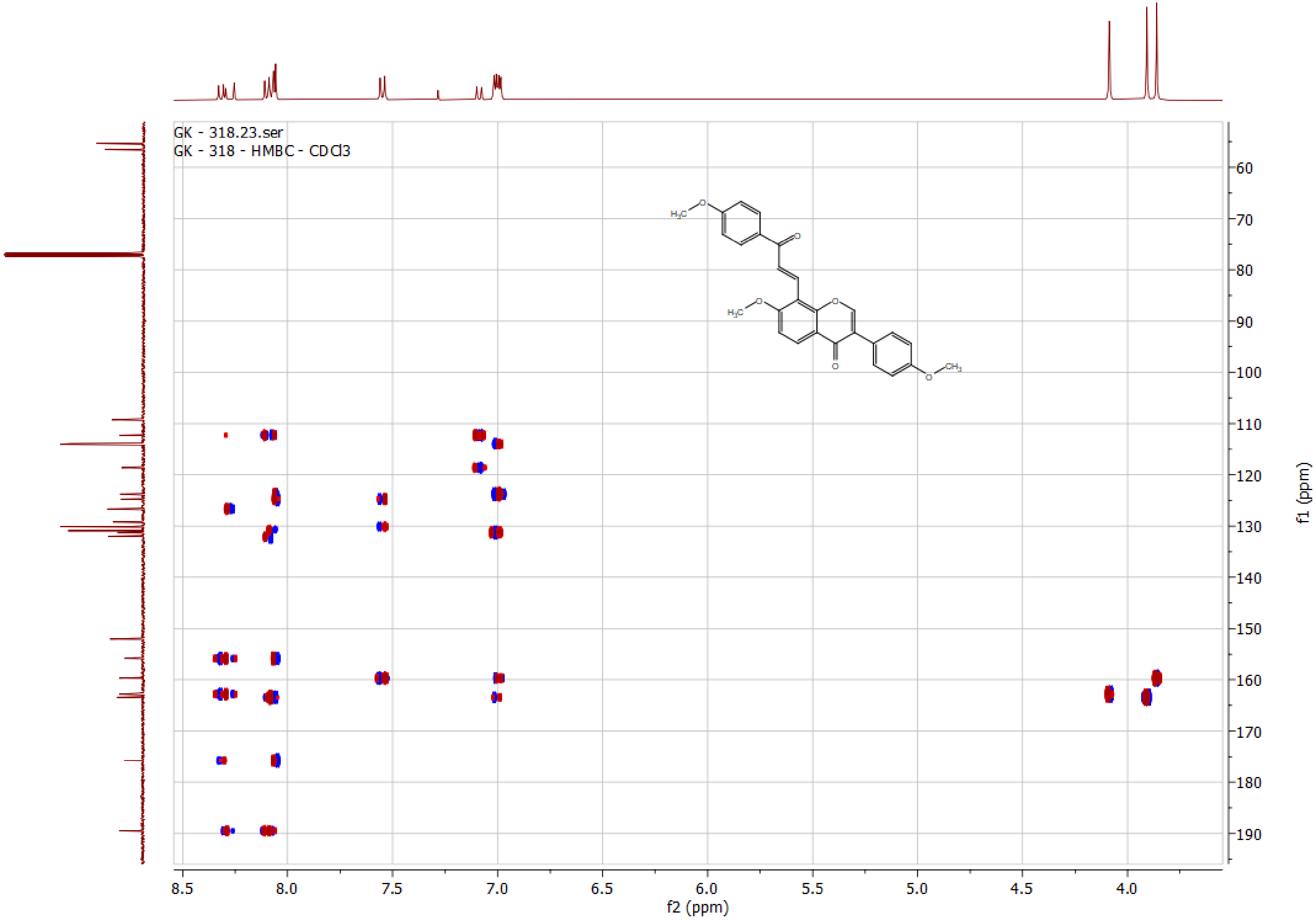

Figure S201: NMR-Signal assignment and selected HMBC-couplings for compound **23**

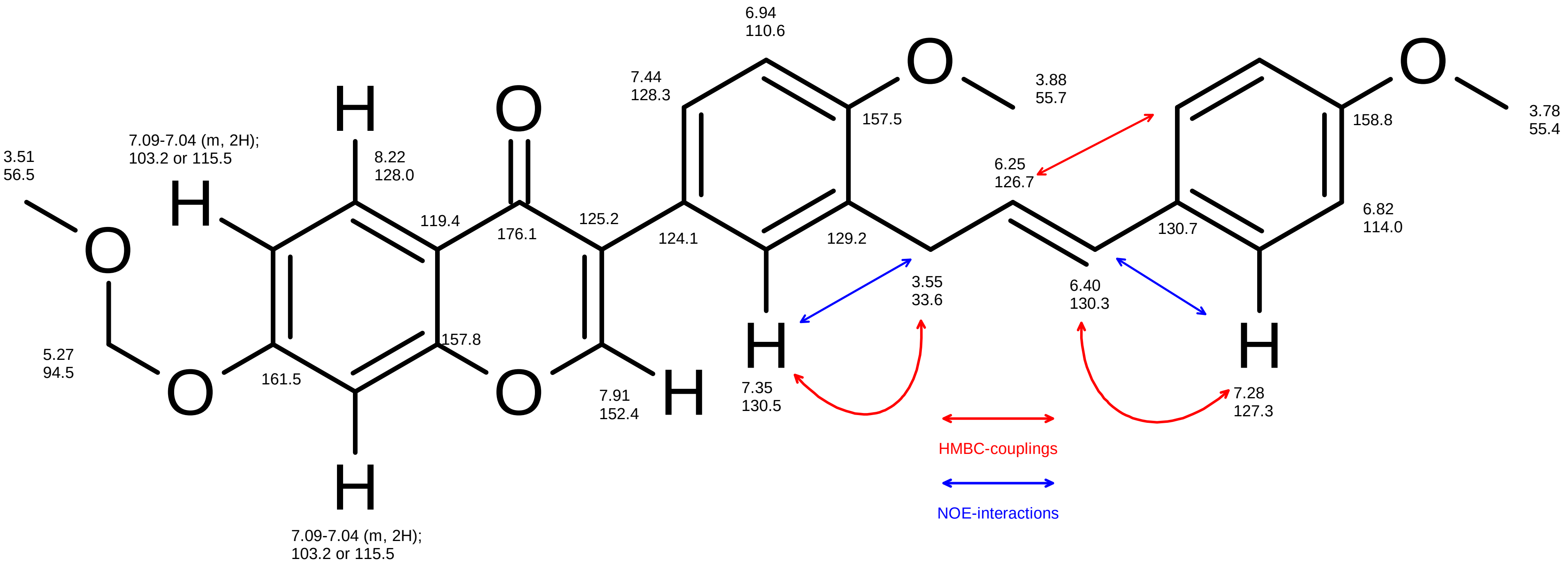

Figure S202: ^{1}H NMR (400 MHz, $CDCl_3$) of **23**

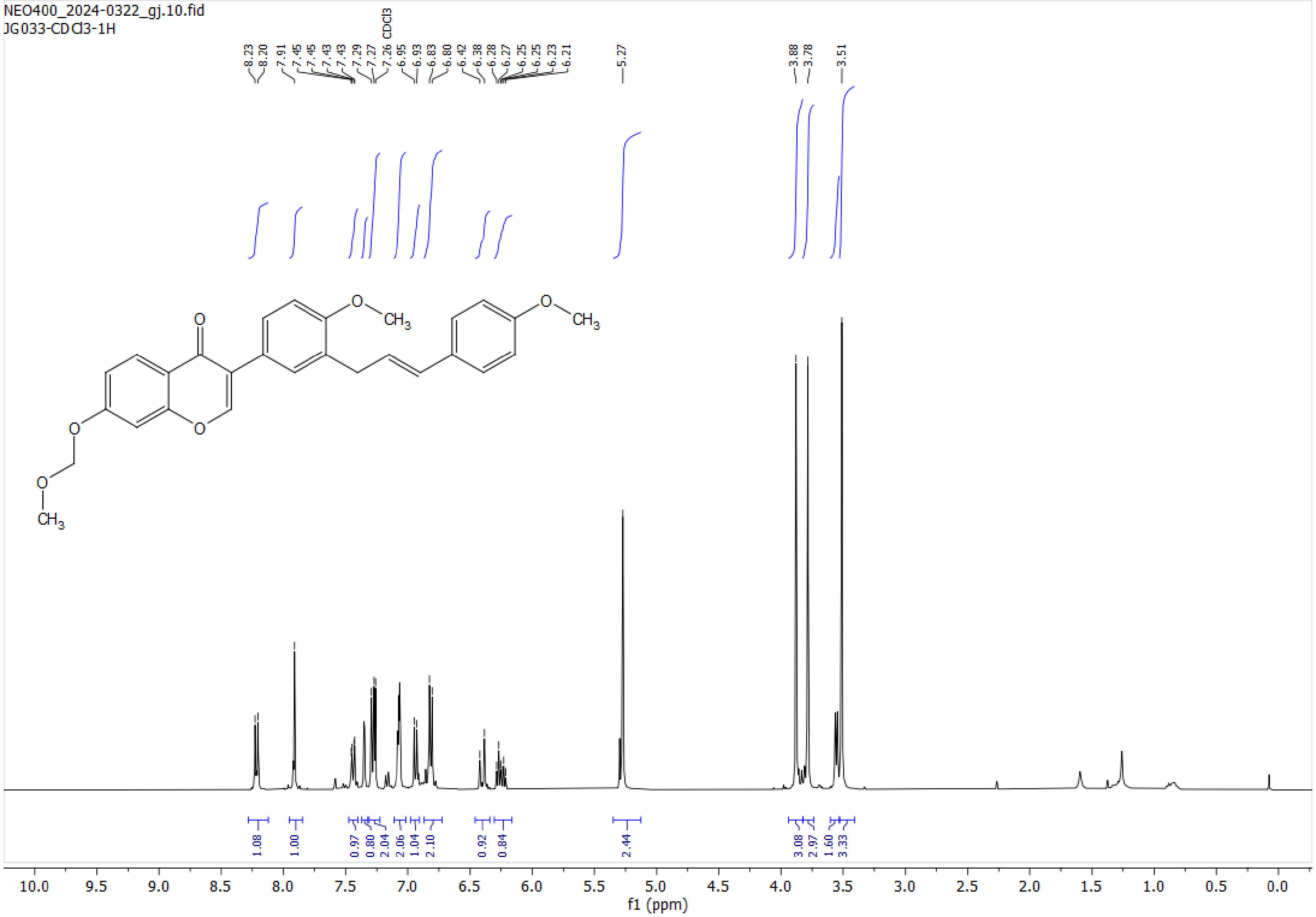

Figure S203: $^{13}C\{^{1}H\}$ NMR (100 MHz, $CDCl_3$) of **23**

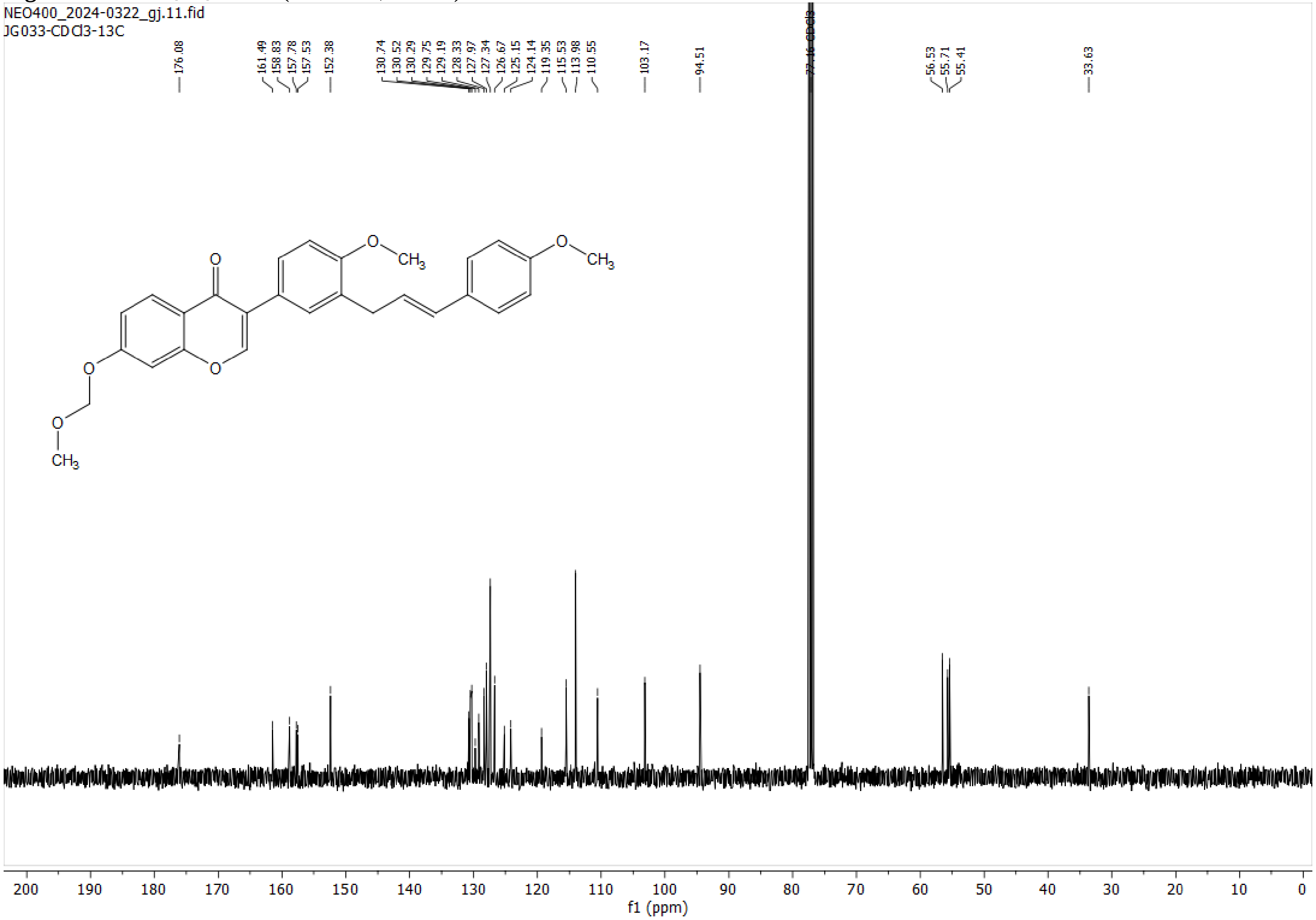

Figure S204: COSY (400 MHz, $CDCl_3$) of **23**

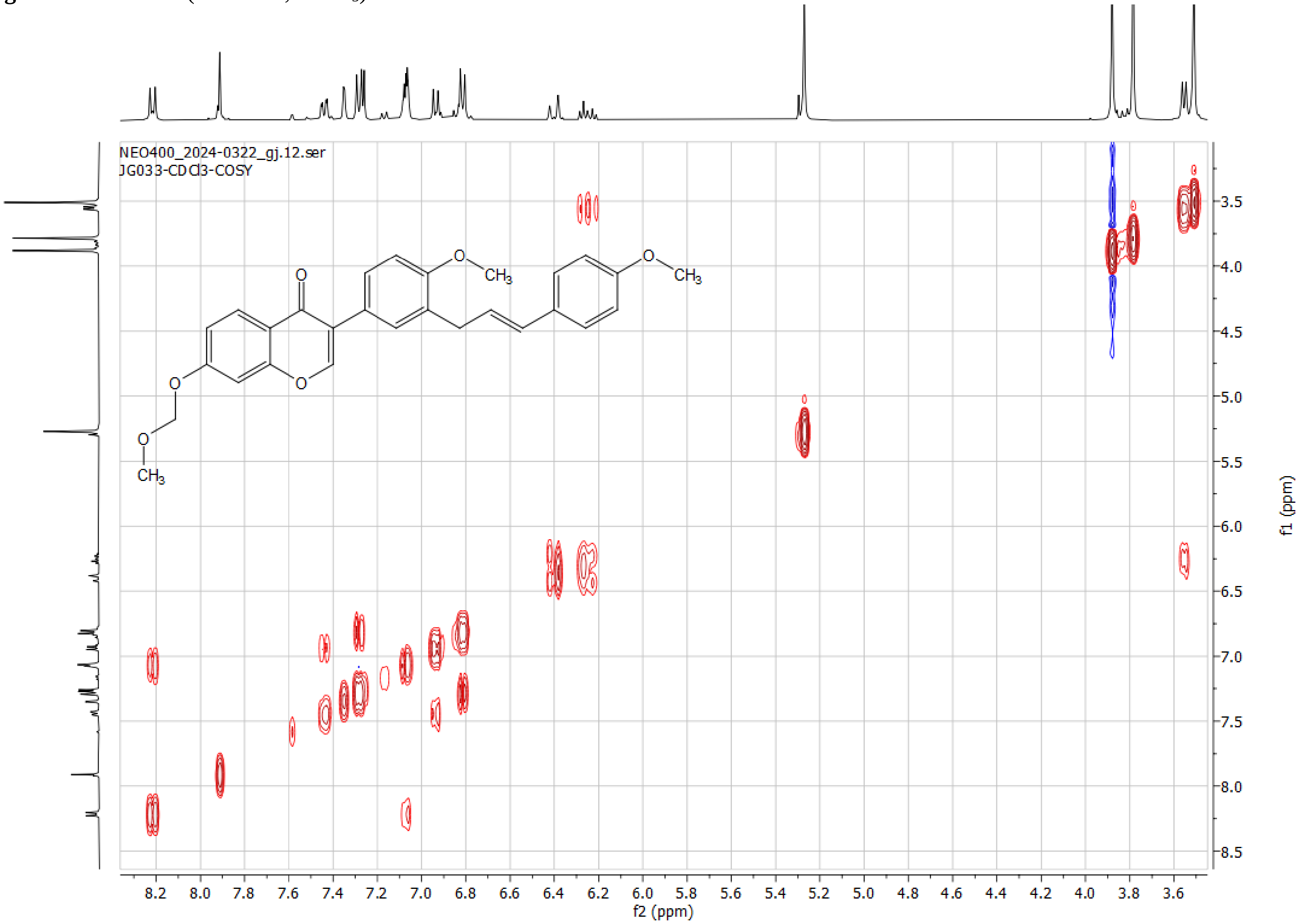

Figure S205: HSQC (400/100 MHz, $CDCl_3$) of **23**

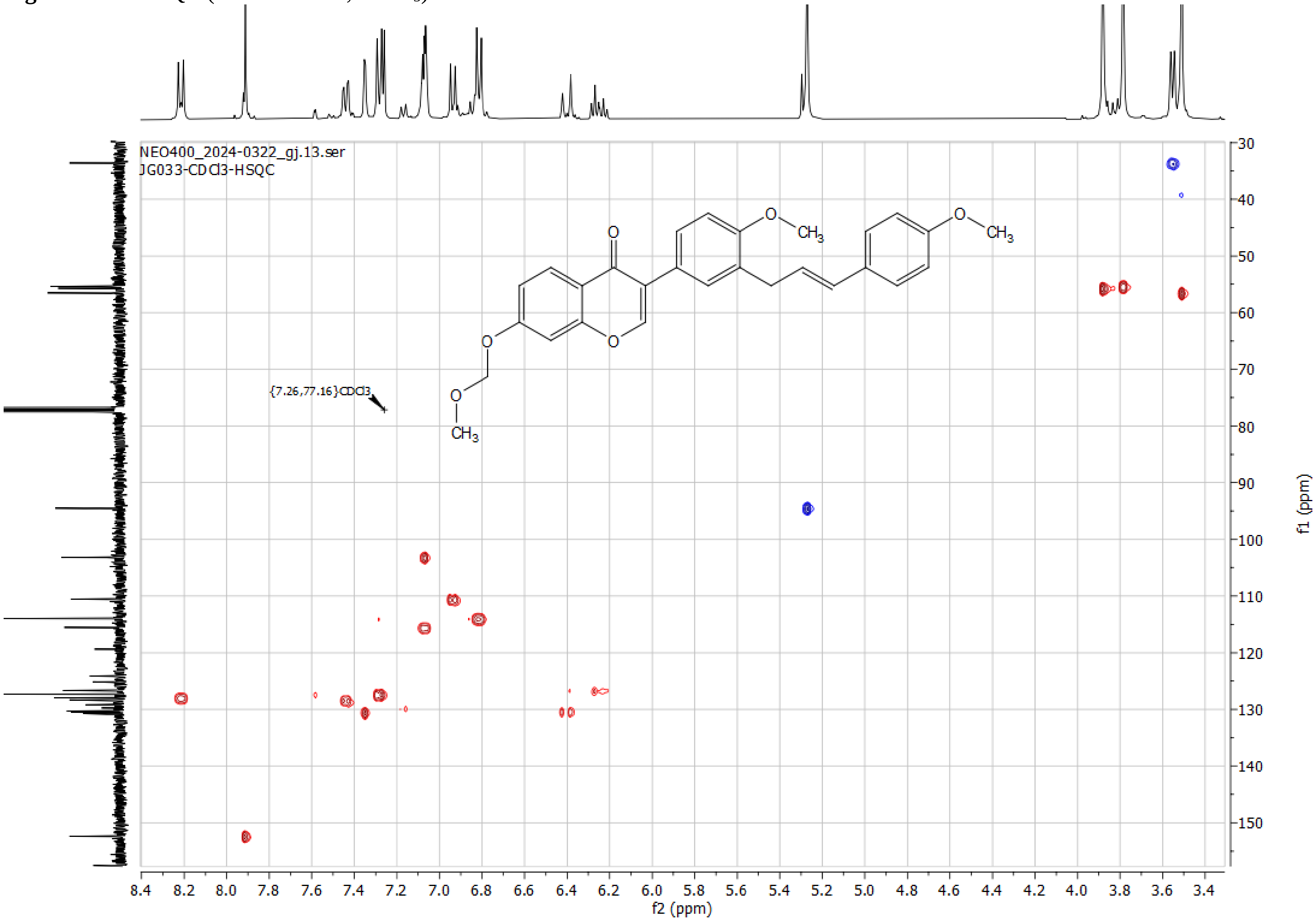

Figure S206: HMBC (400/100 MHz, $CDCl_3$) of **23**

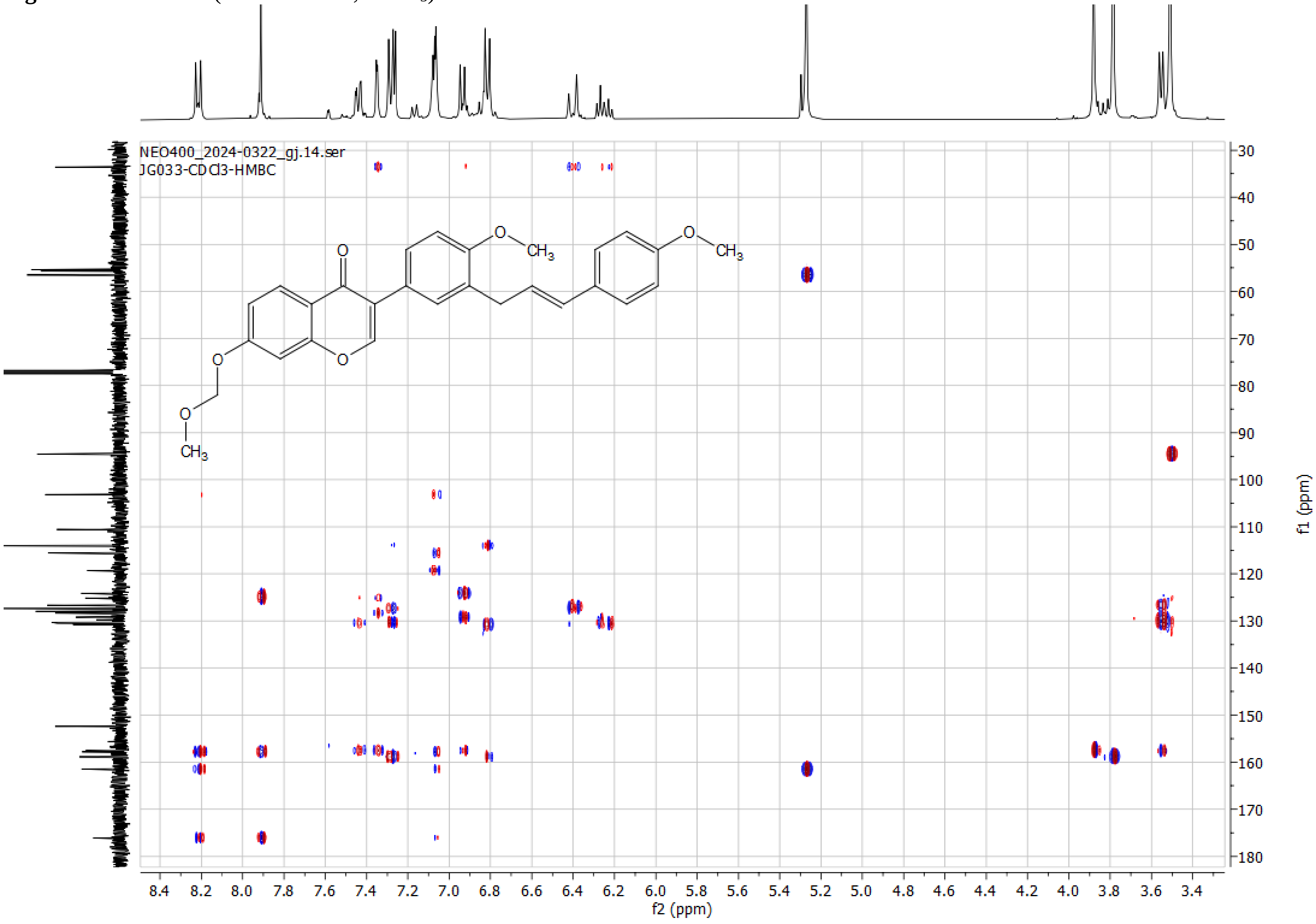

Figure S207: NOESY (400 MHz, $CDCl_3$) of **23**

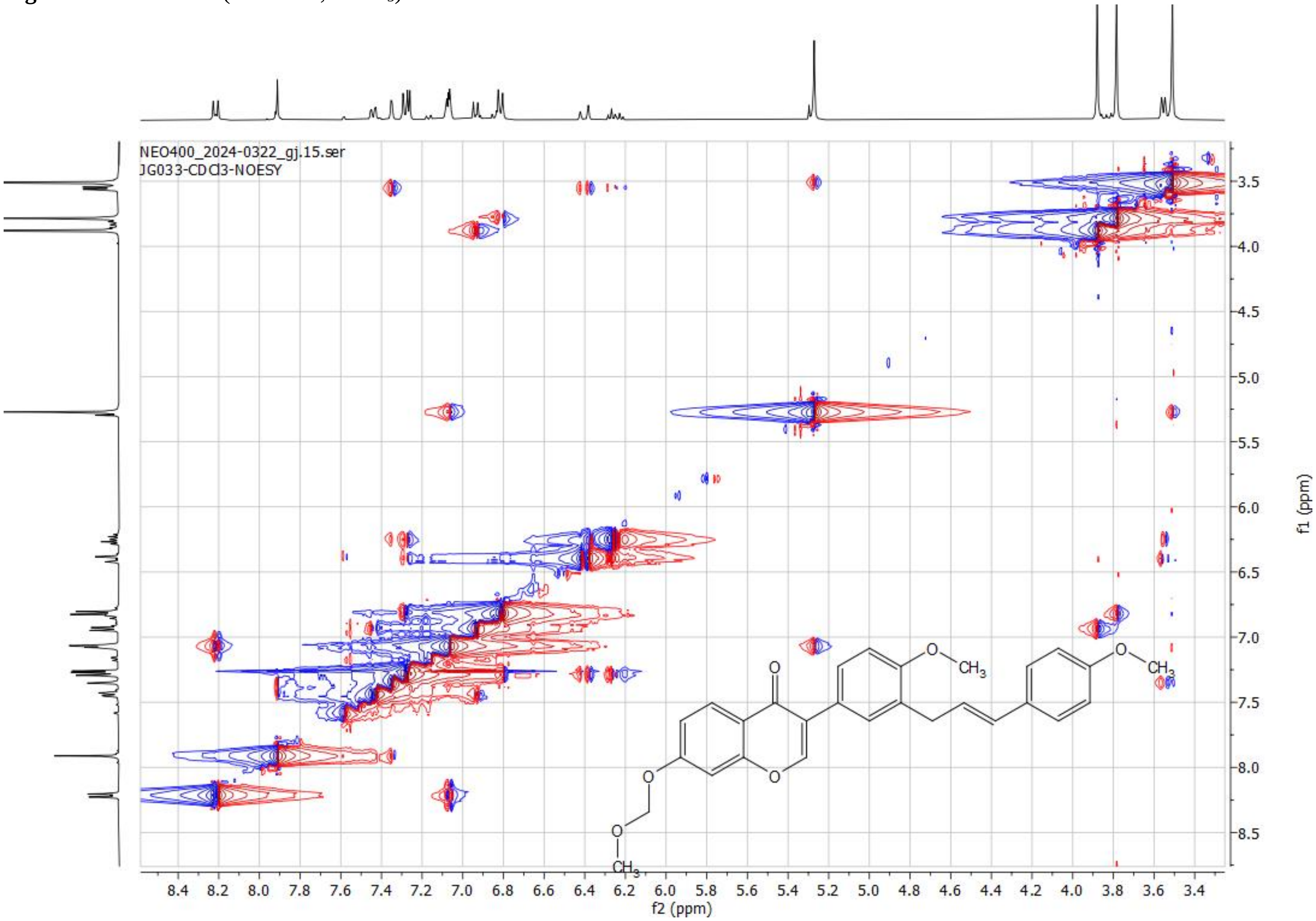

D References

1. STOE & Cie GmbH (2018) X-Area. software package for collecting single-crystal data on STOE area-detector diffractometers, for image processing, for the correction and scaling of reflection intensities and for outlier rejection. STOE & Cie GmbH, Darmstadt.
2. Sheldrick, G. *Acta Cryst. C* **2015**, *C71*, 3-8.
3. Sheldrick, G. *Acta Cryst. A* **2008**, *A64*, 112-122.
4. Kratzert, D. FinalCif. Version V113; online available at: https://dkratzert.de/finalcif.html (last accession date: 2023-03-09).
5. Bandenburg, K. and H. Putz (2020) Crystal Impact Diamond. Diamond - Crystal and Molecular Structure Visualization, Bonn, Germany.
6. Amin, S.; Hecht, S. S.; LaVoie, E.; Hoffmann, D. *J. Med. Chem.* **1979**, *22*, 1336-1340.
7. Sasiambarrena, L. D.; Cánepa, A. S.; Luna, C. N.; Bravo, R. D. *Tetrahedron Lett.* **2015**, *56*, 2054-2058.
8. Terzic, V.; Pousse, G.; Méallet-Renault, R.; Grellier, P.; Dubois, J. *J. Org. Chem.* **2019**, *84*, 8542-8551.
9. Liu, C.-F.; Wang, H.; Martin, R. T.; Zhao, H.; Gutierrez, O.; Koh, M. J. *Nat. Catal.* **2021**, *4*, 674-683.
